# Thimet Oligopeptidase (EC 3.4.24.15) Key Functions Suggested by Knockout Mice Phenotype Characterization

**DOI:** 10.3390/biom9080382

**Published:** 2019-08-19

**Authors:** Nilton B. dos Santos, Roseane D. Franco, Rosana Camarini, Carolina D. Munhoz, Rosangela A. S. Eichler, Mayara C. F. Gewehr, Patricia Reckziegel, Ricardo P. Llanos, Camila S. Dale, Victoria R. O. da Silva, Vanessa F. Borges, Braulio H. F. Lima, Fernando Q. Cunha, Bruna Visniauskas, Jair R. Chagas, Sergio Tufik, Fernanda F. Peres, Vanessa C. Abilio, Jorge C. Florio, Leo K. Iwai, Vanessa Rioli, Benedito C. Presoto, Alessander O. Guimaraes, Joao B. Pesquero, Michael Bader, Leandro M. Castro, Emer S. Ferro

**Affiliations:** 1 Department of Pharmacology, Biomedical Sciences Institute, University of São Paulo (USP), São Paulo, SP 05508-000, Brazil; 2 Department of Pharmacology, Federal University of São Paulo (UNIFESP), São Paulo, SP 04023-062, Brazil; 3 Department of Anatomy, Biomedical Sciences Institute, University of São Paulo (USP), São Paulo, SP 05508-000, Brazil; 4 Department of Pharmacology, Faculty of Medicine of Ribeirao Preto, University of São Paulo, Ribeirão Preto, SP 14049-900, Brazil; 5 Department of Psychobiology, Federal University of São Paulo (UNIFESP), São Paulo 04023-062, Brazil; 6 Department of Pathology, Veterinarian Medical School, University of São Paulo (USP), São Paulo, SP 05508-000, Brazil; 7 Special Laboratory of Applied Toxinology (LETA), Center of Toxins, Immune Response and Cell Signaling (CETICS), Butantan Institute, São Paulo 05503-900, Brazil; 8 Pharmacology Laboratory, Butantan Institute, São Paulo 05503-900, Brazil; 9 Department of Biophysics, Federal University of São Paulo (UNIFESP), São Paulo, SP 04023-062, Brazil; 10 Max-Delbrück-Center for Molecular Medicine, D-13125 Berlin, Germany; 11 Charité - Universitätsmedizin Berlin, 13353 Berlin, Germany; 12 Berlin Institute of Health (BIH), 10117 Berlin, Germany; 13 DZHK (German Center for Cardiovascular Research), Partner Site Berlin, 10785 Berlin, Germany; 14 Institute for Biology, University of Lübeck, 23538 Lübeck, Germany; 15 Biosciences Institute, São Paulo State University (UNESP), São Vicente, SP 11330-900, Brazil

**Keywords:** THOP1, neurodegeneration, inflammation, sepsis, MHC-I, peptidome

## Abstract

Thimet oligopeptidase (THOP1) is thought to be involved in neuropeptide metabolism, antigen presentation, neurodegeneration, and cancer. Herein, the generation of THOP1 C57BL/6 knockout mice (THOP1^−/−^) is described showing that they are viable, have estrus cycle, fertility, and a number of puppies per litter similar to C57BL/6 wild type mice (WT). In specific brain regions, THOP1^-/-^ exhibit altered mRNA expression of proteasome beta5, serotonin 5HT2a receptor and dopamine D2 receptor, but not of neurolysin (NLN). Peptidomic analysis identifies differences in intracellular peptide ratios between THOP1^-/-^ and WT mice, which may affect normal cellular functioning. In an experimental model of multiple sclerosis THOP1^-/-^ mice present worse clinical behavior scores compared to WT mice, corroborating its possible involvement in neurodegenerative diseases. THOP1^-/-^ mice also exhibit better survival and improved behavior in a sepsis model, but also a greater peripheral pain sensitivity measured in the hot plate test after bradykinin administration in the paw. THOP1^-/-^ mice show depressive-like behavior, as well as attention and memory retention deficits. Altogether, these results reveal a role of THOP1 on specific behaviors, immune-stimulated neurodegeneration, and infection-induced inflammation.

## 1. Introduction

Thimet oligopeptidase (EC 3.4.24.15; EP24.15, THOP1) was initially identified as a neuropeptide-inactivating enzyme in rat brain homogenates [1,2,3,4]. The majority of well-characterized THOP1 substrates are neuropeptides [5], including bradykinin [6,7], neurotensin [8,9,10], opioid peptides [1,11,12], angiotensin [13], and gonadotrophin-releasing hormone (GnRH) [14,15,16]. Studies have suggested that THOP1 could be secreted or associated to the external surface of the plasma membrane to function as a neuropeptide-degrading enzyme [17,18,19]. THOP1 secretion from cultured cells has been shown to occur through an unconventional secretory pathway, and is facilitated by interaction with 14-3-3 epsilon and/or calmodulin [20,21]. Phosphorylation of THOP1 at Ser_644_ regulates its secretion and interaction with 14-3-3 epsilon [21], as well as its catalytic activity toward GnRH [22]. THOP1 has substrate size restriction and most of its substrates, products, or competitive inhibitors are peptides containing 9–12 amino acids [23,24,25]. THOP1 substrate size restriction was better understood after solving its crystal structure showing that the catalytic center is located at the bottom of a deep and narrow channel [26]. THOP1 belongs to M3 family of zinc-dependent endopeptidases [2,26,27,28,29]. THOP1′s maximal enzymatic activity is maintained by partial S-glutathionylation, a mechanism that apparently triggers protein oligomerization to dimeric and trimeric catalytically inactive complexes [30,31,32]. THOP1 is ubiquitously expressed in mammalian cells and tissues, and in rodents its specific activity is highest in brain, endocrine tissues, bone marrow and immune system [33]. In rat brain, THOP1’s cellular and subcellular distribution seems to be predominant in the nucleus (>70%), as determined by immunohistochemistry and electron microscopic immunogold labeling [34,35]. The amount of THOP1 found in the nucleus is inversely correlated with that found in the cytosol and associated to the cytosolic face of organelles, suggesting that the enzyme could be mobilized from one intracellular compartment to the other [34]. Therefore, the predominant intracellular localization of THOP1 suggests that extracellular neuropeptide and hormone degradation may not be its main biological role [25,34,35,36,37]. Accordingly, THOP1 was shown to participate in antigen presentation by major histocompatibility class I (MHC I) molecules acting downstream of the proteasome [25,36,37,38].

A substrate-capture assay using a catalytically inactive form of THOP1 uncovered novel natural peptides derived from intracellular proteins, suggesting a broader function for this oligopeptidase than previously anticipated [39,40,41,42]. This initial substrate-capture assay later allowed the finding of a large pool of intracellular peptides in different species [43,44,45,46,47], some of which were characterized either as substrates or products of THOP1 [23]. Indeed, THOP1 inhibition by siRNA affected the relative levels of intracellular peptides in HEK293 cells and, in parallel, increased the signal transduction triggered by isoproterenol [48]. On the other hand, THOP1 overexpression in both CHO-S and HEK293 cells reduced signal transduction of angiotensin II and isoproterenol [49], suggestion that THOP1 plays a role in GPCR signaling. THOP1 overexpression in primary cortical neurons was neuroprotective against amyloid-beta-mediated toxicity, while RNAi knockdown made neurons more vulnerable to amyloid peptide toxicity [50]. THOP1 has been associated to retinal neurodegeneration, which is an early event in the pathogenesis of diabetic retinopathy [51]. THOP1 was also found to be one of the most overexpressed genes related to epigenetic interactions in lung adenocarcinoma of poor prognosis [52].

The present study aimed to generate and perform an initial phenotype characterization of THOP1 C57BL/6 knockout mice (THOP1^-/-^). THOP1^-/-^ mice were viable and displayed normal external phenotype and fertility. The litters had normal number of animals. THOP^-/-^ mice could not be visually distinguished from WT littermates. Overall, the results presented here link THOP1 to brain disorders such as depression, attention and memory retention deficits, in addition to immune-stimulated neurodegeneration and infection induced inflammation.

## 2. Results

### 2.1. THOP1^-/-^ Generation

**Figure 1:**
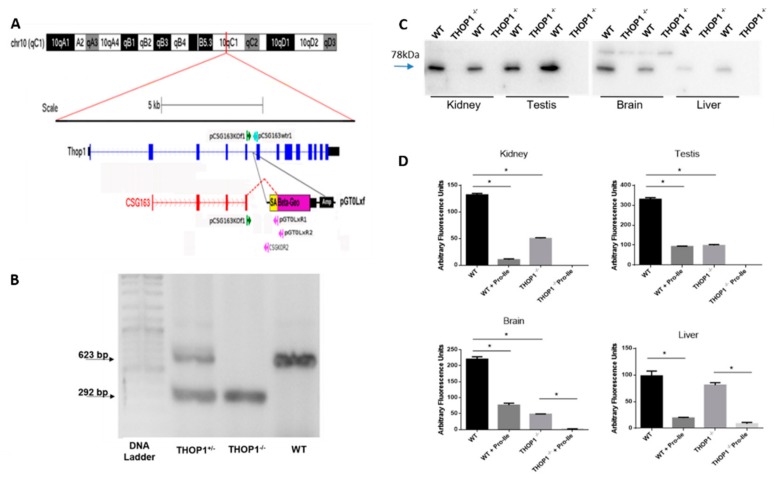
Generation and characterization of THOP1 knockout mouse. (**A**) representation of THOP1 gene structure and the location of the CSG163 line gene trap insertion. The position of the THOP1 locus on a schematic representation of the mouse chromosome 10 is shown as a red vertical line (chr10-qC1). The THOP1 exons are represented as blue blocks connected by arrowed lines, blue blocks indicating the coding and black blocks the untranslated regions. The gene trap 5′RACE cDNA sequence for the CSG163 strain is represented as red blocks connected at the 3′ end by dashed red lines to the Beta-Geo exon just downstream of the mouse En2 intron/exon splicing acceptor site (SA) region of the indicated gene trap vector pGT0Lxf. The forward and reverse primers are indicated and represented respectively as green and light blue or pink arrows; (**B**) typical results obtained by genotyping polymerase chain reaction (PCR) for THOP1^+/-^, THOP1^-/-^ or WT mice (bp: base pairs); (**C**) Western blots show the presence of a single 78 KDa band corresponding to THOP1 in the different organs investigated in WT mice. Note the lack of this 78 kDa band in THOP1^-/-^ mice in all organs investigated. The differences in Western blot band intensities correspond to the specific expression of THOP1 in the different tissues (highest in testis and kidneys. and lower in the liver); 60 µg of protein from crude 10.000× *g* supernatant was applied in each lane. The anti-THOP1 antiserum was previously described [34,35]. (**D**) THOP1 enzymatic activity was determined in different tissue homogenates from either THOP1^-/-^ or WT mice using the quenched fluorescence substrate Abz-GGFLRRVNH2-EDDnp (QFS) in the presence or absence of NLN inhibitor Pro-Ile (5 mM). Experiments were conducted in triplicates that varied less than 5% among each other and results are presented as mean ± S.E.M. Statistical significance was determined by one-way ANOVA test. Tukey’s post hoc. * *p* < 0.05; *n* = 3 per group.

To generate the THOP1^-/-^ mice, we used embryonic stem cells with a gene-trap cassette inserted into intron 5 of the THOP1 gene on chromosome 10 (Figure 1A), which was confirmed by genotyping of the resulting mice (Figure 1B). To obtain a pure genetic background, we bred F1 (129/OlaHsd/C57BL/6 background) heterozygous THOP1-deficient animals to the C57BL/6 mouse line for at least ten consecutive generations (Supplementary information Trap THOP1 knockout generation). Tail DNA samples were extracted and sent to the Charles River Laboratory (Genetic Testing Services, Wilmington, MA, USA) for the complete mapping of microsatellite markers. It was confirmed that these animals had a genetic background of 99.25%, on average, of the C57BL/6 line (data not shown). Mice were genotyped before experimentation. THOP^-/-^ mice showed a 292 bp electrophoresis band, heterozygous animals displayed two bands (292 and 623 bp), and WT animals presented a 623 bp band (Figure 1B).

THOP1^-/-^ mice were totally viable and presented no deviations from normal Mendelian distributions following intercrossing of the either heterozygote or homozygote animals. THOP1^-/-^ mice could not be visually distinguished from WT C57BL/6 littermates and had normal external appearance and fertility. Homozygous males and females were both fertile and the litter size was usually 7–8 puppies. The reproductive abilities and estrous cycle of THOP1^-/-^ and WT mice were similar, suggesting that GnRH and luteinizing hormone (LH) metabolism was not significantly compromised in THOP1^-/-^ mice.

THOP1 protein expression was evaluated by Western blot and showed highest levels in testis and kidneys followed by brain, and lower expression in liver (Figure 1C). A complete lack of immunoreactivity was observed in tissue homogenates obtained from THOP1^-/-^ compared to WT mice (Figure 1C). THOP1 enzymatic activity was determined in crude tissue homogenates, using the quenched fluorescence substrate Abz-GGFLRRVNH2-EDDnp (QFS), in the presence or absence of the NLN specific inhibitor Pro-Ile (5 mM). In WT mice, Pro-Ile inhibited different percentages of QFS degradation (Figure 1D). NLN inhibition was highest in testis (220 AFU from a total of 320 AFU; 68%), followed by brain (145 AFU from a total of 220 AFU; 65%), kidneys (110 AFU from a total of 140 AFU; 78%), and liver (80 AFU from a total of 100 AFU; 80%). These data suggest that in the absence of THOP1, NLN is the major QFS degrading activity in mice. In THOP1^-/-^ mice, Pro-Ile completely inhibited the remaining QFS degrading activity in brain, testis and liver, supporting the specificity of this fluorescent assay to determine THOP1 and NLN activities in these tissues. The lowest THOP1 specific activity was observed in liver, which is in agreement with the Western blot data (Figure 1C,D).

**Figure 2:**
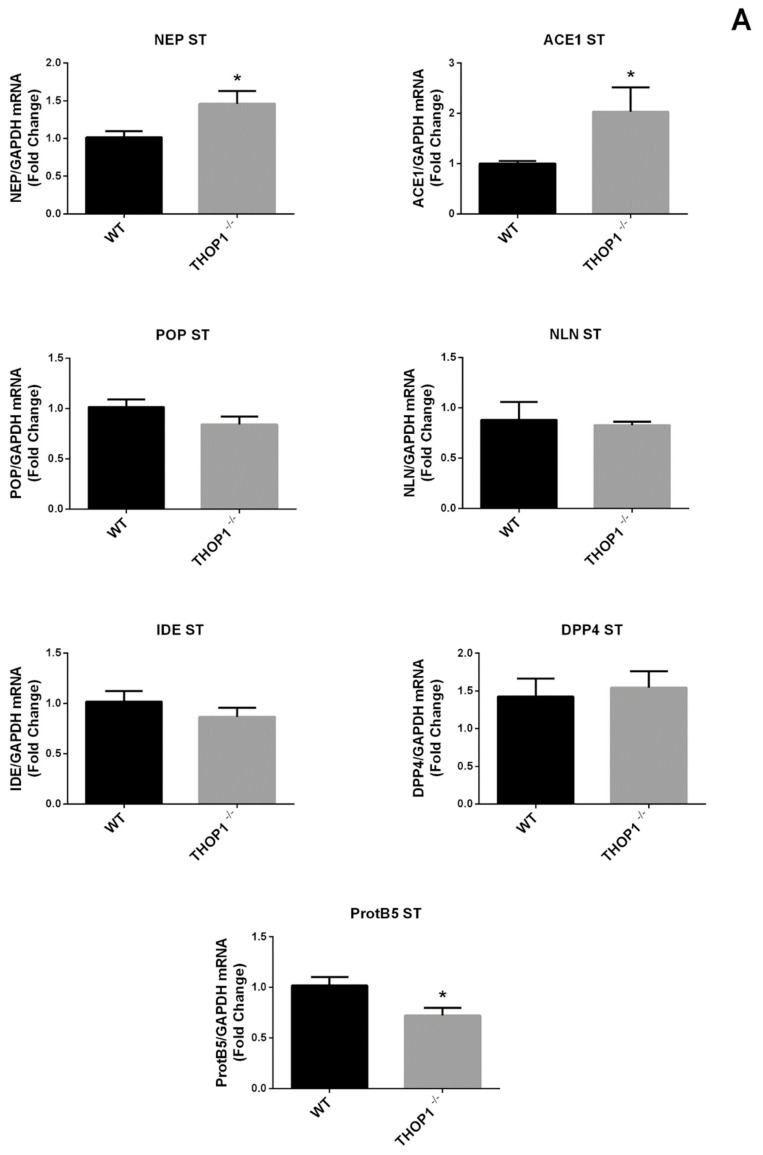

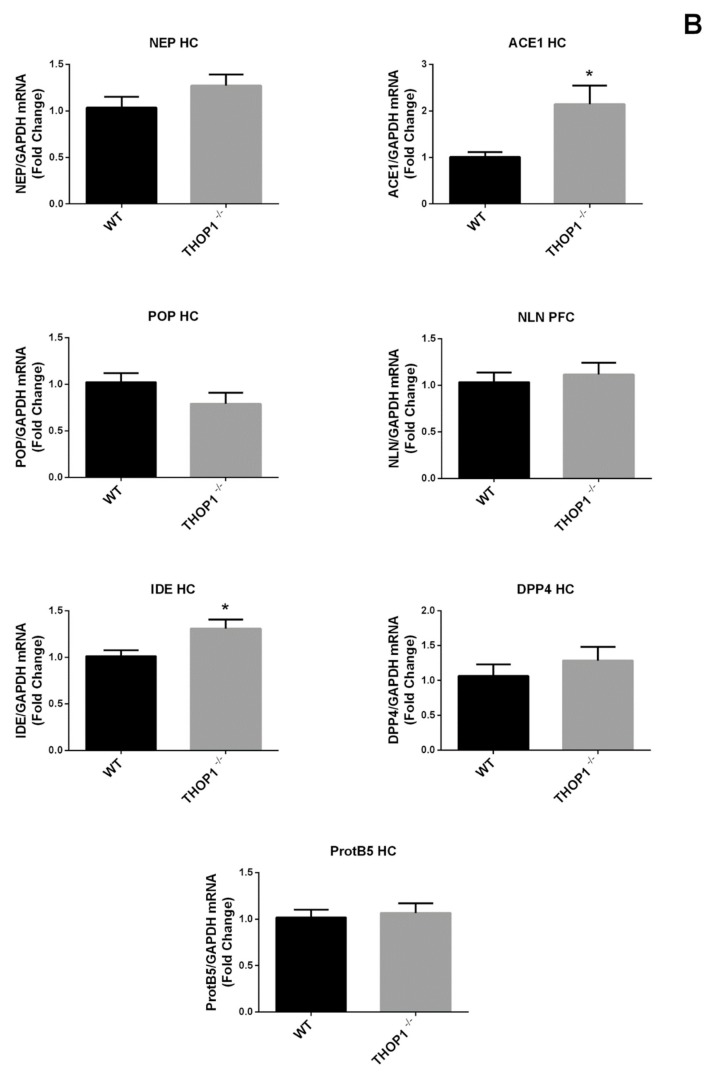

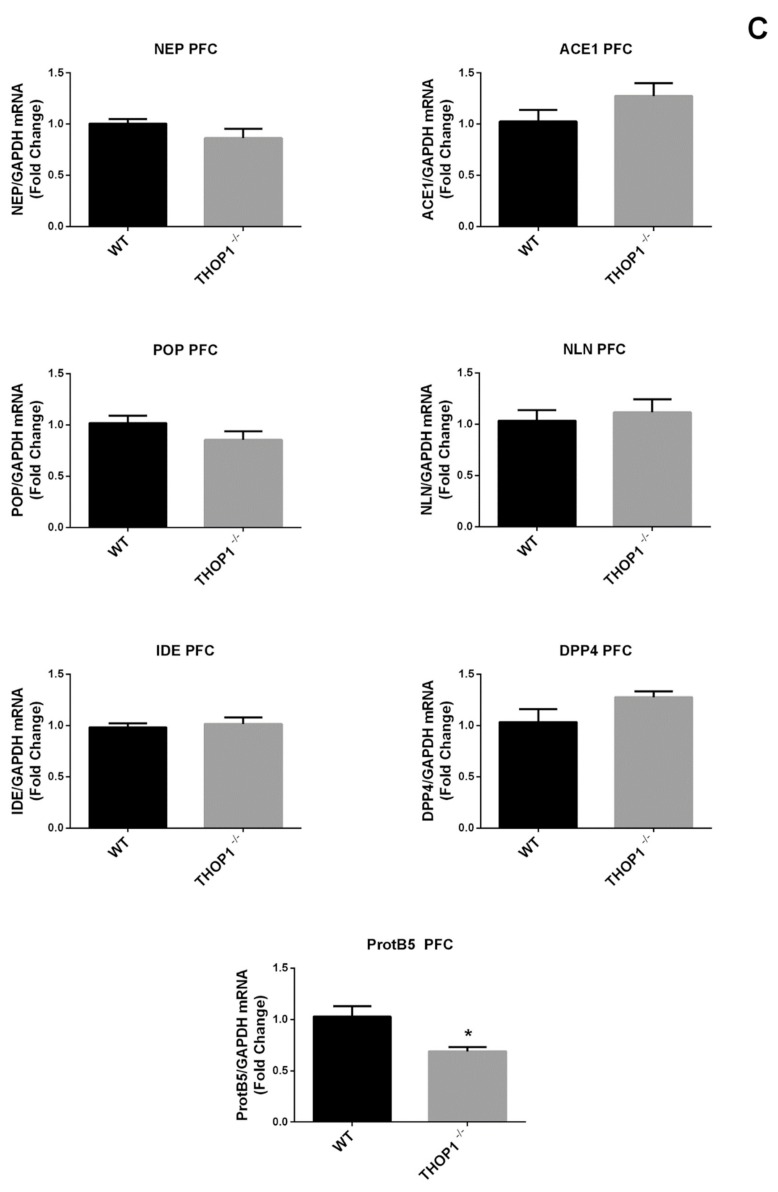
Gene expression of peptidases and proteasome beta5 subunit in different areas of mouse brain. qRT-PCR was used to investigate the mRNA levels for specific peptidases or proteasome beta5-subunit (ProtB5): (**A**) striatum (ST), (**B**) hippocampus (HC), and (**C**) prefrontal cortex (PFC). Neprilysin (NEP), angiotensin converting enzyme 1 (ACE1), prolyl-oligopeptidase (POP), insulin degrading enzyme (IDE), dipeptidyl peptidase 4 (DPP4), or neurolysin (NLN). Results are expressed as mean ± S.E.M. Statistical significance was determined by Student’s *t*-test. * *p* < 0.05. *n* = 6–9.

Next, the mRNA expression of NLN and several other peptidases and the proteasome beta5-subunit (ProtB5) were evaluated in striatum (ST), hippocampus (HC), and prefrontal cortex (PFC) of WT and THOP1^-/-^ brains using quantitative real-time (qRT) PCR (qRT-PCR) (Figure 2). mRNA levels of proteasome beta5-subunit (ProtB5) was reduced both in ST and PFC, and remained unaltered in the HC (Figure 2). An increase in mRNA levels of neprilysin (NEP) and angiotensin converting enzyme 1 (ACE1) was observed in ST from THOP1^-/-^ compared to WT mice, whereas no changes were found for prolyl-oligopeptidase (POP), neurolysin (Nln), insulin degrading enzyme (IDE), and dipeptidyl peptidase 4 (DPP4) mRNA expressions between THOP1^-/-^ and WT mice (Figure 2A). In HC from THOP1^-/-^ mice, mRNA levels of ACE1 and IDE increased, whereas no alterations were observed for NEP, POP, NLN, or DDP4 (Figure 2C). Taken altogether, these data suggest that THOP1 suppression slightly affects mRNA levels of several peptidases and ProtB5 in specific areas of mouse brain.

### 2.2. Global Gene Expression Analyses

Affymetrix^®^ microarrays were used to evaluate global mRNA expression in prefrontal cortex, hippocampus, and striatum of THOP1^-/-^ compared to WT mice (Supplementary Information Microarray data). Among the immediately adjacent genes to THOP1 on chromosome 10, accessory protein 6 receptor and pseudogene EFF2 were not differentially expressed in comparisons with genes from the same region, showing that the gene trap insertion appears to have not influenced physically close genes (Supplementary Information Microarray data). The assessments of global mRNA levels in WT and THOP1^-/-^ mice shows that only the thymine DNA glycosylase (TDG) gene was differentially expressed (false discovery rate with adjusted *p*-value/Q-value cut-off of 0.05) in the evaluated areas of THOP1^-/-^ brain. The other differentially expressed genes were six reporter control probe sets of the microarray, and mitochondrial encoded tRNA cysteine in prefrontal cortex. However, excluding outliers [53], the hippocampus samples showed 395 differentially expressed genes, including TDG and the same six reporter controls probe sets previously found (Supplementary Information Microarray data).

### 2.3. Peptidomics

Semiquantitative peptidome analyses using dimethylation-isotopic labeling and electron-spray mass spectrometry were used to determine possible differences in the relative levels of peptides in PFC, ST and/or HC of THOP1^-/-^ compared to WT mice (Supplementary data shows further MS and MS/MS data). The 66 distinctive peptides identified were derived from 25 proteins with main subcellular localization in cytosol (C; 66%), mitochondria (M; 16%), secretory pathway (V; 13%), plasma membrane/cytosol (PM/C; 5%), or nucleus (N; 3%) (Table 1). In PFC, five out of 36 peptides identified in THOP1^-/-^ were reduced in their levels by more than 50% compared to WT mice, and only two peptides were increased more than 100% (Table 1). In ST, none of the 38 peptides appeared reduced whereas only one peptide (SANSNPAMAPRE) was increased more than 100% in THOP1^-/-^ compared to WT (Table 1). In HC, none of the 45 peptides identified were reduced, whereas four were increased more than 100% in THOP1^-/-^ compared to WT (Table 1). Thus, from the 63 peptides identified here, four peptides, all found in PFC, could be considered products of THOP1, while seven peptides found in PFC (2), ST (1), or HC (4) could be considered substrates of THOP1. It is to note that in some cases the same peptides (VEKVDELKKKYGI and KQATVGDVNTDRPGLLDL) are decreased in PFC, unchanged in ST and increased in HC (Table 1). Intracellular peptides levels have been shown to present greater variations among samples compared to neuropeptides [45], which may explain the large inter sample variations observed for most of the peptides identified (Table 1). THOP1 gene ablation affected the expression of ProtB5 and additional peptidases, which may have influenced the levels of at least some peptides. Moreover, the present data suggest that only 26% of the peptides identified in at least two samples were present in all three investigated areas, suggesting that similarly to neuropeptides some intracellular peptides could be specific to certain areas of mouse brain.

### 2.4. Complete Blood Count and Coagulation Analysis

Complete blood count of THOP1^-/-^ females before immunization showed increased percentage of granulocytes and monocytes and decreased percentage of lymphocytes compared to WT mice (Table 2), suggesting intrinsic autoimmune or blood disorders of THOP1^-/-^ animals. Thrombocytopenia was also observed in THOP1^-/-^ compared to WT mice (Table 2), which could contribute to prolonged bleeding of these animals empirically observed following small surgical procedures (i.e., small tail/ear cuts for genotyping). Blood coagulation was further investigated using the nonactivated thromboelastometry (NATEM) test [55]. Results showed that the parameters clotting time, clotting formation time, maximum clotting firmness, and maximum lysis were all similar comparing THOP1^-/-^ and WT (data not shown). The prolonged bleeding of THOP1^-/-^ was not correlated to a decreased blood coagulation time, which suggest that further investigations are necessary to uncover the possible mechanism behind altered thrombus formation in THOP1^-/-^. Additional blood parameters including hemoglobin concentration, white blood cells, red blood cells, red cell distribution width, mean corpuscular volume and mean corpuscular hemoglobin concentration were similar (Table 2).

### 2.5. Autoimmune Encephalomyelitis Neurodegeneration Model

**Figure 3:**
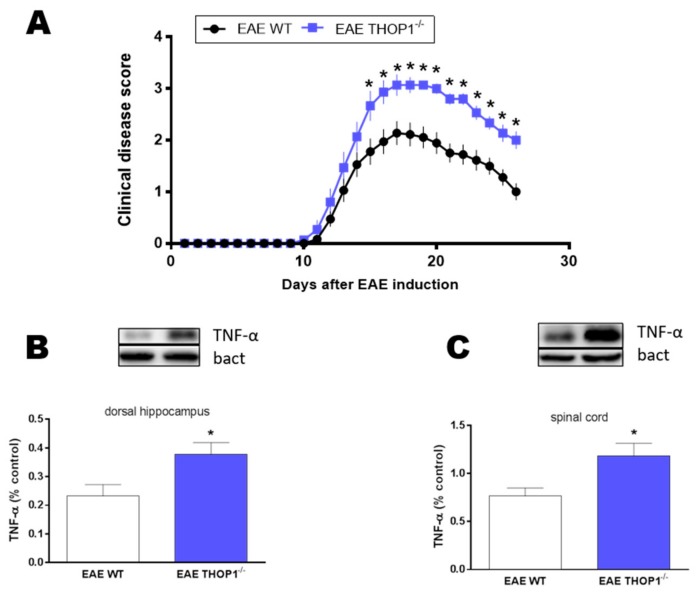
Neurodegeneration induced by EAE and inflammatory profiles. (**A**) Evaluation of the clinical score over time of WT C57BL/6 female mice and THOP1^-/-^ immunized with MOG^35-55^ emulsified with complete Freund’s adjuvant (CFA). Results are expressed as mean ± S.E.M. Statistical significance was determined by two-way ANOVA. Tukey post hoc: * *p* < 0.05. EAE WT *n* = 18 and EAE THOP^-/-^ *n* = 15. Western blotting for TNF-α in dorsal hippocampus (**B**) or spinal cord (**C**) at 26th dpi; blots (**B,C**) were cropped (indicated by boxes) from the same membranes that were successively exposed to distinctive antibodies as indicated in Experimental Procedures (Supplemental Information—TNF-α Western blots). Results are expressed as mean ± S.E.M. Statistical significance was determined by Mann–Whitney test: * *p* < 0.05. EAE WT *n* = 6. EAE THOP1^-/-^ *n* = 6. bact: beta-actin.

Autoimmune encephalomyelitis (EAE) is the most common animal model for multiple sclerosis as they share many clinical and pathophysiological features [56,57]. Following immunization with MOG35-55 peptide, THOP1^-/-^ mice showed poor clinical score from 17th-26th day postimmunization (dpi) in EAE, compared to WT mice (Figure 3). EAE symptoms of THOP1^-/-^ were associated with increased tumoral necrosis factor alpha (TNF-α) levels in the dorsal hippocampus and spinal cord at the 26th dpi (Figure 3). Altogether, these data supports that THOP1 may be involved in neurodegeneration [50,51] and autoimmunity [36,38].

### 2.6. Sepsis

**Figure 4:**
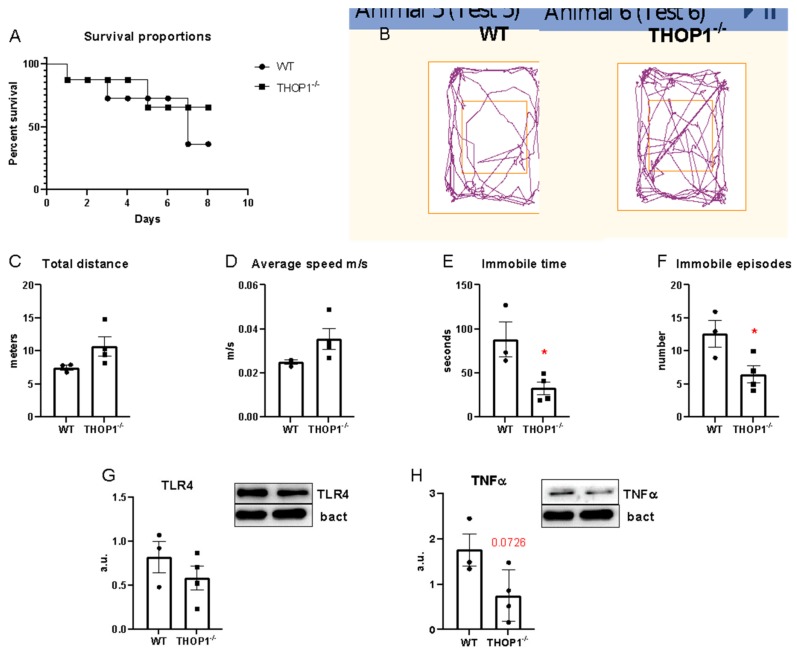
Survival curves and behavior analysis of mice undergoing sepsis. (**A**) THOP^-/-^ and WT mice survivors during 7 days after sepsis induction. Next, survived animals of each group were submitted to Open Field test and Western blot analysis of dorsal hippocampus. Results were expressed as % survival during 7 days (*n* = 6). (**B**) Distance tracking in open field task. (**C**) Total distance performed in Open field. (**D**) Average speed in open field (**E**) total immobility time and (**F**) total immobility episodes. (**G**) TLR4 and (**H**) TNF-α expression in total protein extract of the dorsal hippocampus. Behavior tests and Western blots were performed at the 8th day after sepsis induction. Results are expressed as mean ± S.E.M. Statistical significance was determined by Student’s *t*-test: * *p* < 0.05 vs. WT. WT. *n* = 3; THOP1^-/-^. *n* = 4.

Animals were subjected to cecal ligation and puncture (CLP) sepsis protocol with a 21-gauge needle. All sham-operated animals survived for at least 7 days after surgery (data not shown). WT animals that received 2 punctures showed 50% mortality (3 from 6), whereas THOP1^-/-^ animals showed a 30% mortality (2 from 6) up to 7 days after the surgery (Figure 4). Animals that survived from sepsis (3 WT and 4 THOP1^-/-^) were subjected to behavior tests in an open field arena at the 8th day. WT animals showed an average speed and total traveled distance smaller than THOP1^-/-^ animals (Figure 4). On the other hand, WT animals demonstrated greater immobility time than THOP1^-/-^ animals (Figure 4). These data suggest that THOP1^-/-^ improved the performance of animals subjected to sepsis and it could be a potential drug target.

### 2.7. Behavior Analysis

**Figure 5:**
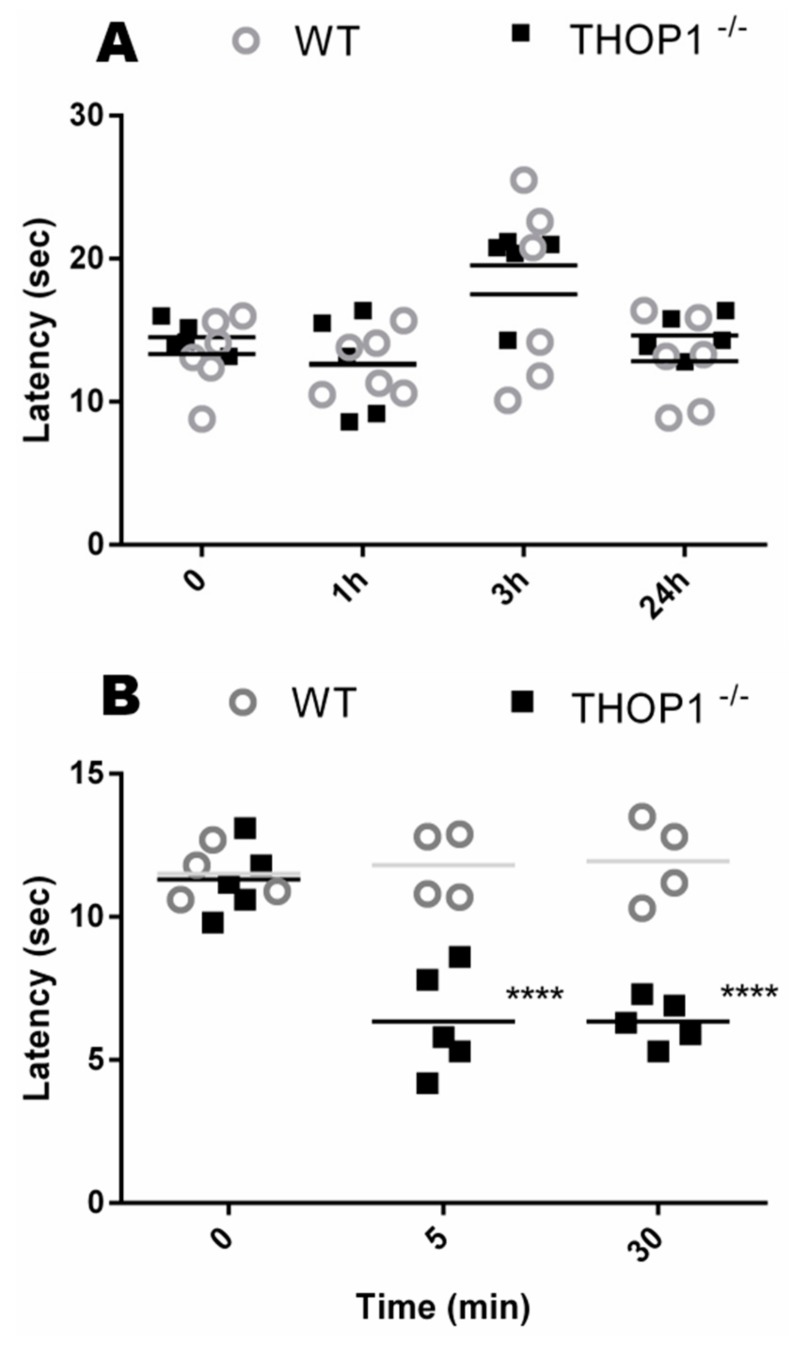
Hot plate test. **(A)** Regular basal nociceptive response of THOP1^-/-^ or WT litter mates, evaluated by the hot plate test at different time points. Note that THOP1^-/-^ latency to nociceptive stimulus is similar to WT at this determined dose of BK (0.1 µM; 40 µL). Data are expressed as mean ± SEM of 4–6 animals per group. Data were analyzed by two-way ANOVA followed by Bonferroni’s multiple comparison test. (**B)** Nociceptive response was evaluated after Bk was injected intraplantar (0.1 µM; 40 µL). Note that THOP1^-/-^ latency to nociceptive stimulus is significantly lower than that of WT. Data presented as mean ± SEM. Data were analyzed by two-way ANOVA followed by Bonferroni’s multiple comparison test. **** *p* < 0.001 compared to either control (0 min) or to WT (after 5 or 30 min of Bk intraplantar administration).

In the hot plate test, WT and THOP1^-/-^ mice demonstrated similar basal nociceptive sensibility (Figure 5). However, following intraplantar injection of bradykinin, the latency of paw withdrawal response of THOP1^-/-^ was significantly lower than that of WT mice (Figure 5). Together, these data indicate that THOP1 is a rate-limiting peptidase for bradykinin inactivation in peripheral tissues [58].

**Figure 6:**
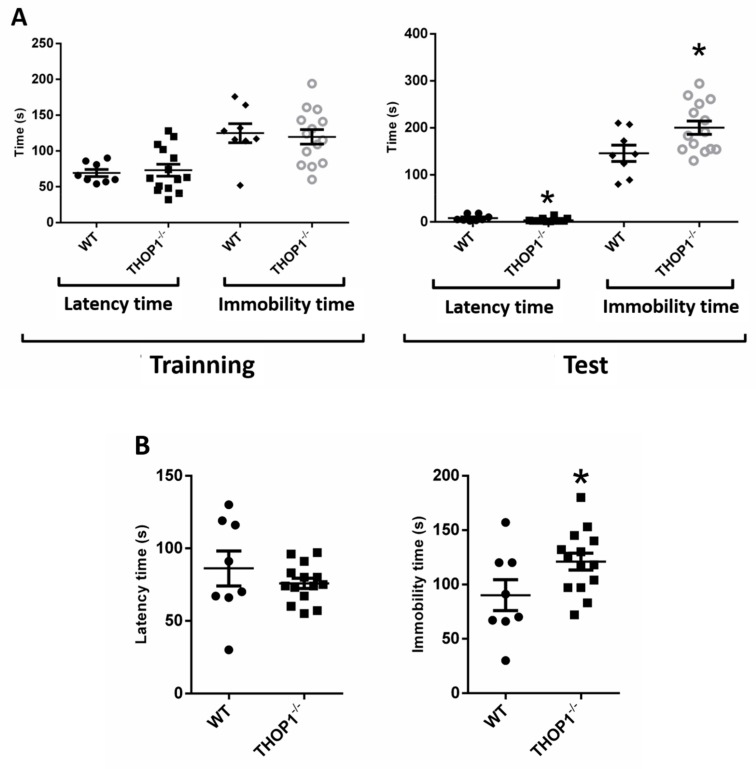
Depression-like behavior analysis using the forced swim (FST) and tail suspension (TST) tests. Panel (**A**): In the FST, Training corresponds to the first day of tests and Test corresponds to the second day of tests. Latency time to the first immobility episode and total period (in seconds) of animal immobility during the 5 min of forced swimming task duration. In both parameters, THOP1^-/-^ mice present a mild (*p* < 0.05) depressive-like behavior compared with WT mice. Panel (**B**): TST were conducted as detailed in Experimental procedures. THOP1^-/-^ have similar latency and higher immobility time compared to WT. Results are expressed as mean ± S.E.M. Statistical significance was determined by Student’s *t*-test: * *p* ≤ 0.05 vs. WT. WT. *n* = 8; THOP1^-/-^. *n* = 14.

No depression-like behavior was observed for THOP1^-/-^ compared to WT mice during the first day of forced swimming test (FST) training. In the second day of FST experiments, a mild depression-like behavior was observed for THOP1^-/-^ mice, which exhibited slightly greater latency time to the first immobility episode as well as greater immobility periods during the 5 min of the FST experiments than WT control group (Figure 6A). To further evaluate this depressive-like phenotype of THOP1^-/-^ additional tests were conducted using the tail suspension test (TST). The latency period of time for both WT and THOP1^-/-^ were similar in the TST, whereas THOP1^-/-^ mice hung by the tail developed an immobile posture that last longer than WT mice (Figure 6B). Additional experiments should be conducted in the future with both THOP1^-/-^ and WT, male and females, to deeply investigate the chronic and acute effects of antidepressives such as fluoxetine and imipramine on this phenotype.

**Figure 7:**
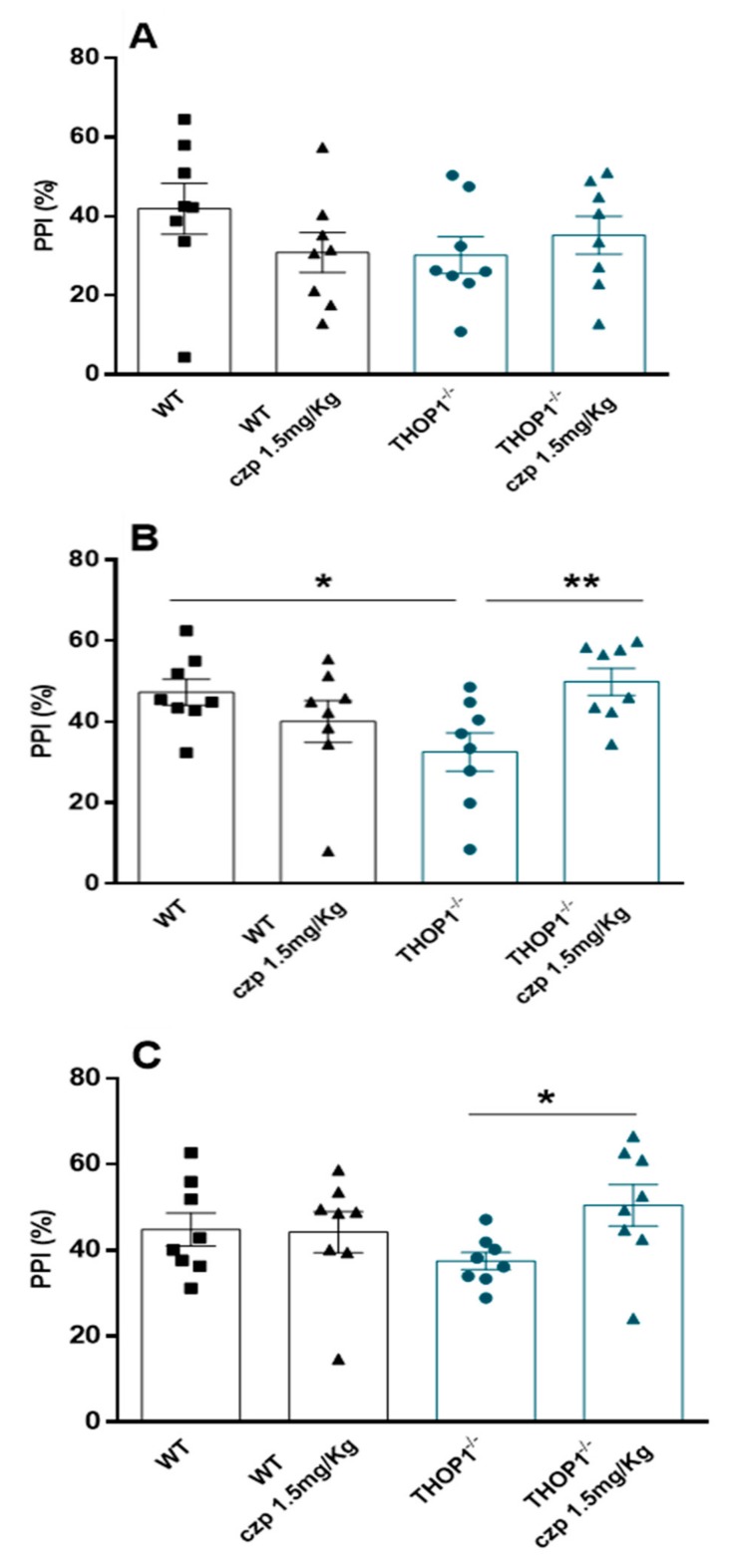
Prepulse inhibition of startle test. Percentage of prepulse inhibition of startle for each prepulse (PP) intensity: 75 dB (**A**), 80 dB (**B**), and 85 dB (**C**). Note that clozapine (czp) induced a significant increase in PPI response of THOP1^-/-^ at both 80 and 85 dB (B,C), whereas it did not modify the response of WT mice (A–C). Results are expressed as mean ± S.E.M. Statistical significance was determined by one-way ANOVA test. Tukey’s post hoc: * *p* < 0.05; ** *p* < 0.01). *n* = 8. per group.

The prepulse inhibition of startle reflex test (PPI) is a paradigm that assesses the functioning of sensorimotor gating. Deficits in PPI indicate impaired information processing and are seen in psychiatric disorders, such as attention deficit hyperactivity disorder (ADHD) and schizophrenia. Compared to WT, THOP1^-/-^ animals displayed impaired PPI (%) after presentation of 80 dB prepulse, but not after the prepulses of 75 and 85 dB (Figure 7). Administration of clozapine restored THOP1^-/-^ animals’ PPI deficit after the prepulse of 80 dB and increased their PPI response after the prepulse of 85 dB. These pharmacological responses suggest that disruption of THOP1 expression in mice induces a behavioral phenotype that resembles those seen in psychiatric disorders involving attention deficits (Figure 7B,C).

**Figure 8:**
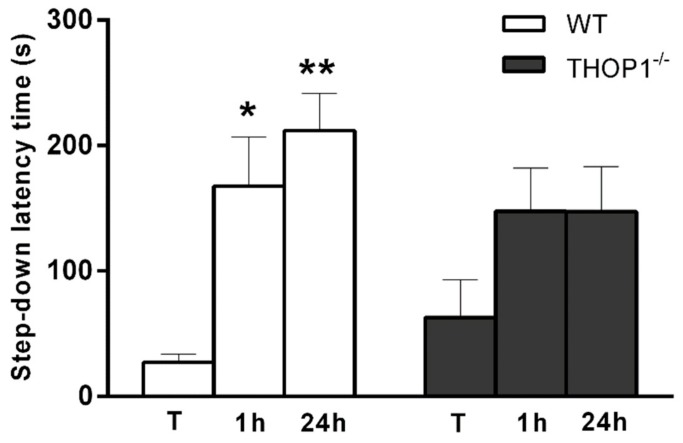
Step-down passive avoidance task: in this task, the time the animal takes to step-off from the platform (step-off latency) was determined. The longer the mouse stays on the platform. the greater was the latency for step-off. indicating that the mouse learned the passive avoidance task. WT showed higher latency to step-down during tests as compared to training. THOP^-/-^ showed an impairment of memory retention at training-tests. Results are expressed as mean ± S.E.M. Statistical significance was determined by two-way ANOVA test. Sidak’s post hoc: * *p* < 0.05 between WT training (T) and WT 1 h test; ** *p* < 0.01 between WT training (T) and WT 24 h test; No statistically significant differences were observed for the THOP1^-/-^ mice between training and 1 h or 24 h tests. WT. n = 10; THOP1^-/-^. *n* = 9.

Another feature presented by the THOP1^-/-^ mice was that they presented lower latency to step-down when compared to WT mice during the training-tests. These data suggest that THOP1^-/-^ animals have an impairment in their memory retention process (Figure 8); the longer the mice stay on the platform, the greater the latency for step-off, indicating that the mouse learned the passive avoidance task.

Further behavior analyses showed no alterations on locomotor activity with either THOP1^-/-^ or WT animals, which spend similar time in different zones of the open field apparatus with similar rearing behavior/amount of time crossing the central square/instances of grooming (Supplementary Figure S1). The spontaneous activity of THOP1^-/-^ and WT animals during the 24 h period was similar, except in the interval from 5 p.m. to 10 p.m. where THOP1^-/-^ have smaller spontaneous activity than WT animals (data not shown). The elevated plus maze task suggested no signs of altered anxiety behavior of THOP1^-/-^ compared to WT mice (Supplementary Figure S2). In Barnes maze and novel object recognition tasks also no alterations of THOP1^-/-^ in learning/cognitive performance were observed comparing to WT (Supplementary Figure S3).

### 2.8. Neurotransmitter Levels and Turnover

**Figure 9:**
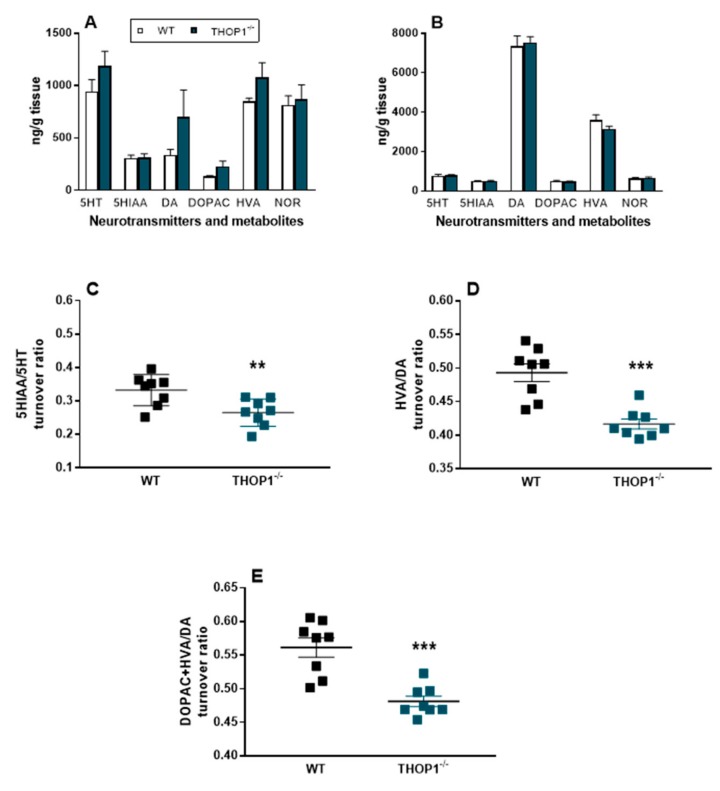
Neurotransmitters and their metabolites levels in two different brain regions of WT and THOP1^-/-^ mice. No differences were observed among the neurotransmitters and their metabolites in the PFC (**A**) or ST (**B**) from WT mice compared to THOP1^-/-^ mice. However, the turnover ratio of 5HIAA/5HT was significantly lower in PFC of THOP1^-/-^ mice (**C**). Similarly, the turnover ratios of HVA/DA (**D**) and DOPAC+HVA/DA (**E**) are lower in ST of THOP1^-/-^ mice compared to WT mice. Results are expressed as mean ± S.E.M. Statistical significance was determined by Student’s *t*-test: ** *p* < 0.01; *** *p* < 0.001 vs. WT. *n* = 8.

Similar concentrations of dopamine (DA), serotonin (5HT), noradrenaline (NOR), and the metabolites 3,4-dihydroxyphenylacetic acid (DOPAC), homovanillic acid (HVA), and 5-hydroxyindoleacetic acid 5 (5HIAA) were observed in PFC (Figure 9A) and ST (Figure 9B) of THOP1^-/-^ compared to WT (Figure 8). In addition, neurotransmitter turnover ratios were analyzed showing that the relationship of 5HIAA/5HT was significantly lower in PFC of THOP1^-/-^ compared to WT mice (Figure 9). Similarly, the turnover ratios of HVA/DA and DOPAC+HVA/DA were lower in ST of THOP1^-/-^ mice compared to WT mice.

### 2.9. mRNA Levels of 5HT 2a and DA D2 Receptors

**Figure 10:**
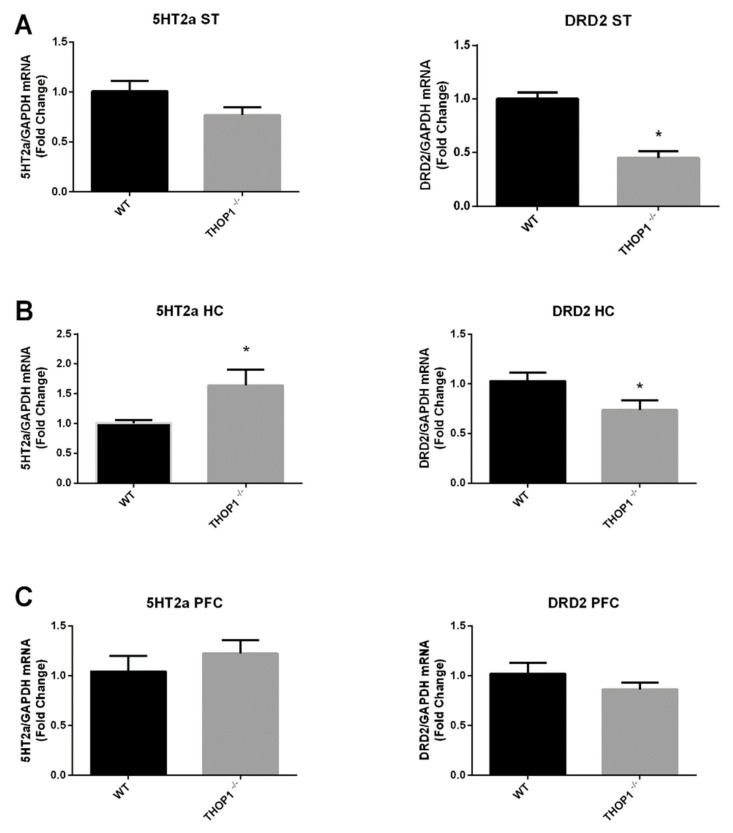
Gene expression of 5HT2a and DRD2 in different areas of mice brain. THOP1^-/-^ mice showed upregulation of 5HT2a gene expression in ST (**A**), HC (**B**), and PFC (**C**); whereas, in the same brain areas, DRD2 mRNA showed no changes (A–C). Results are expressed as mean ± S.E.M. Statistical significance was determined by Student’s *t*-test. * *p* < 0.05. *n* = 6–9.

Next, qRT-PCR was used to evaluate the mRNA levels of 5HT 2a receptor (5-HT2a) and DA D2 receptor (DRD2), showing differential regulation in specific areas of THOP1^-/-^ brain compared to WT (Figure 10). 5HT2a mRNA was increased in HC and DRD2 reduced in ST and HC (Figure 10A,B). In PFC, neither 5HT2a nor DRD2 mRNA were altered comparing THOP1^-/-^ to WT (Figure 10C). Together, these data suggest that at least some behavior differences observed between THOP1^-/-^ and WT mice could involve alterations in serotonin and dopamine neurotransmission.

## 3. Discussion

In this study, we successfully generated a model of THOP1 knockout mice that will contribute to a better understanding of the biological functions of this oligopeptidase. The present data also strengthen the involvement of THOP1 in neurodegeneration, peripheral bradykinin inactivation, as well as generation and degradation of intracellular peptides. However, the molecular mechanisms behind the phenotype differences seen in THOP1^-/-^ are still to be fully elucidated. Altogether, these findings provide better insights about THOP1′s biological role and highlights its potential as drug target for the development of novel therapies.

THOP1^-/-^ mice were viable and have normal external appearance, estrous cycle, and fertility. Their litters had a normal number of animals, which cannot be visually distinguished from WT littermates. GnRH is critical for pubertal development and maintenance of reproductive competence, with normal estrous cycle being produced by a series of hormonal signals that starts with the release of GnRH from the hypothalamus [59,60]. After being secreted, GnRH can be degraded in the hypothalamus and the anterior pituitary gland by two endopeptidases—THOP1 and POP—acting in a stepwise manner [61]. A possible role of brain THOP1 in the regulation of GnRH effects was suggested, for example, since intracerebroventricular administration of *N*-[1-(RS)-carboxy-3-phenylpropyl]-Ala-Ala-Phe-p-aminobenzoate (cFP-AAF-pAB), a specific inhibitor of THOP1, resulted in increased gonadotropin secretion and increased recovery of intracerebroventricular-administered GnRH [4,14]. The direct correlation between THOP1 and in vivo GnRH metabolism has not been established here due to technical difficulties for GnRH and luteinizing hormone (LH) quantification in samples of portal hypophyseal and plasma from mice; these limitations have also been previously reported [59,60]. However, the lack of changes in the estrous cycle together with a normal reproductive function and litters of normal numbers, as well as normal expression of POP in all brain regions investigated herein, are strong evidences that both GnRH and LH levels are in a regular physiological range in THOP1^-/-^. Inhibition of ACE1 by cFP-AAF-pAB cleavage product *N*-[1(*R*,*S*)-carboxy-3-phenylpropyl]-Ala-Ala [62] could be an additional reason for these apparently contradictory results. Cleavage of cFP-AAF-pAB at the Ala-Phe bond by NEP produces *N*-[1(*R*,*S*)-carboxy-3-phenylpropyl]-Ala-Ala, which is a potent inhibitor of ACE1 [62]. Therefore, after in vivo administration THOP1 specific inhibitor cFP-AAF-pAB could be cleaved by NEP inhibiting ACE1, which is a peptidase that can affect GnRH stability and reproduction [62,63,64]. Here we have also observed that POP mRNA expression was normal in ST, HC and PFC, which could be an additional explanation that THOP1^-/-^ have normal reproductive functions. POP is well known as a key enzyme for GnRH metabolism [61] and pulsatility [60], and could metabolize GnRH. Future investigations could be performed to further investigate the possible involvement of THOP1 in fine molecular tuning mechanisms related to sexual organ development, maturation and puberty onset.

THOP1 was also shown herein to regulate mRNA levels of ProtB5, NEP, ACE1, and IDE in ST and HC, but not in the PFC of mouse brain. THOP1 is mainly localized inside the nucleus in brain neurons, where it could be directly contributing to peptide metabolism, and indirectly to transcriptional regulation. Previous studies have demonstrated that peptides are potential ligands with high affinities for hairpin RNAs. As a result, these peptides serve as inhibitors of viral replication [65,66,67]. The intracellular peptide VGSELIQKY, corresponding to amino acids 251–259 of the human 19S ATPase regulatory subunit 4, stimulates the expression of the immunoproteasome beta 5i subunit increasing the proliferation of CD8+ T cells [68]. ACE1 mRNA expression was seen to increase in ST and HC of THOP1^-/-^ mice, and the ACE1 gene insertion/deletion polymorphisms has been shown to play a role in susceptibility to schizophrenia and also in its depressive symptom severity in a Han Chinese population [69]. Global gene expression analysis suggested that the gene trap had no influence in adjacent genes. Excluding outliers, hippocampus samples showed 395 differentially expressed genes, including TDG and the six reporter controls probe sets. Therefore, through a yet unknown mechanism it is possible that THOP1 could be acting at the transcriptional and/or post-transcriptional levels to regulate mRNA levels, which then affect some of the phenotypes characterized here.

THOP1 suppression affected the levels of specific peptides in particular regions of the central nervous system (CNS), including ST, HC, and PFC. Previous observations suggested that peptidases are not substrate-specific [70]. Thus, a great diversity of peptidases and proteases expressed in the brain may compensate for the lack of THOP1. In the PFC, none of the peptidases investigated were differentially expressed, which corroborates the largest variation in peptide levels observed in this CNS region of THOP1^-/-^ brain. In the ST no peptides were altered in THOP1^-/-^, whereas the mRNA levels increased for both ACE1 and NEP that share many substrates with THOP1 [40,71]. Therefore, an increase of ACE1 and NEP in ST and HC of THOP1^-/-^ may have compensatory effects on the metabolism of peptides. However, no differences were observed herein in NLN mRNA expression. Previous studies using NLN knockout mice suggested a slight increase in 31 peptides that could represent substrates of NLN, and a slightly decrease of six peptides that may represent products of NLN [54]. Therefore, whereas changes observed herein in the peptidome cannot be associated to NLN, four peptides (among 65; ASKGLGSDLDSSLASL, KGLGSDLDSSLASL, NDFASAVRILEV, and ADKVPKTAENF), identified here, were identical to those previously found in NLN knockout mice brain [54]. Among these four peptides, a slight reduction was observed for peptides ASKGLGSDLDSSLASL and KGLGSDLDSSLASL, derived from clathrin coat assembly protein AP180, and NDFASAVRILEV, derived from cytochrome c oxidase subunit 5A, only in the PFC of THOP1^-/-^. None of these four peptides was altered in NLN knockout mice brain [54]. These results suggest that NLN and THOP1 have distinctive substrates in mouse brain, which is corroborated by their distinctive subcellular localization in neurons, with THOP1 mainly localized in the nucleus and NLN mainly present in dendrites [34,35]. Moreover, proteasome inhibition has been shown to affect the levels of most intracellular peptides both in human cell lines and yeast [47,72,73], suggesting that the reduced expression of ProtB5 observed herein could be a biological response to compensate for the lack of THOP1. THOP1 downregulation affects peptide levels and the expression of ProtB5 and additional peptidases, supporting its role in brain intracellular peptide metabolism. However, further studies are yet necessary to clarify the possible molecular mechanisms linking gene expression of peptidases and ProtB5 and changes in intracellular peptides.

THOP1 gene suppression strongly affected the clinical performance of mice in experimental EAE: an animal model of multiple sclerosis, a chronic neuroinflammatory demyelinating disorder of the CNS with a marked neurodegenerative component initiated by CD4+ T cells [74,75]. During EAE, MHC I-restricted myelin basic protein is presented by oligodendrocytes and cross-presented by Tip-dendritic cells (DCs) to CD4+ T cells. These observations suggest that in EAE CD4+ T cell-mediated CNS autoimmunity leads to determinant spreading to myelin-specific CD8+ T cells, which can directly recognize oligodendrocytes [75]. THOP1 was previously suggested to function in antigen presentation through MHC I-associated antigens [25,36,38]. Many of the THOP1 substrates identified by these approaches are 9–11 amino acids in length, supporting the proposal that THOP1 can function in the degradation of peptides that could be used for antigen presentation. However, THOP1 also converts some peptides into products that are 8–10 amino acids, thus contributing to the formation of peptides for antigen presentation [23]. Therefore, depending on the antigenic peptide, THOP1 can either degrade or generate epitopes for MHC-I presentation. Indeed, stable retroviral silencing of endogenous THOP1 by RNA interference induced a striking, long-term increase in surface MHC I [37], whereas overexpression of THOP1 caused a marked reduction in the levels of H-2Kb and HLA-A3 molecules on COS7 cell surface [76]. THOP1 was required, either together with nardilysin or alone, for the generation of a tumor-specific CTL epitope from PRAME, an immunodominant CTL epitope from Epstein–Barr virus protein EBNA3C, and a clinically important epitope from the melanoma protein MART-1 [38]. Here, THOP1^-/-^ have worse clinical scores in EAE from 17th-26th dpi compared to WT, which was associated to increased TNF-α levels in the dorsal hippocampus and spinal cord at the 26th dpi. These data suggest that THOP1 is directly or indirectly contributing for the degradation of EAE antigenic epitopes protecting mice against neuroimmune-induced degeneration. Several studies have shown that CD8^+^ T cells, activated by MHC I from DCs and oligodendrocytes, potentiate CD4^+^ T cell-mediated EAE, contributing to inflammation, demyelination, and tissue damage in the CNS [57,75,77]. Overexpression of THOP1 had little or no effect on the presentation to a specific T cell hybridoma of a class II-presented peptides generated from ovalbumin [76], suggesting that further studies are still necessary to investigate the role of THOP1 in antigen cross-presentation. Together, these data successfully shown that THOP1 plays a key function in autoimmune-induced neurodegeneration in experimental EAE.

THOP1^-/-^ showed increased percentages of granulocytes and monocytes and a decreased percentage of lymphocytes in blood, which could also contribute for the differences seen in their immune and inflammatory response. THOP1 has been implicated in rheumatoid arthritis (RA), another common autoimmune disease manifesting as chronic inflammation of the joints and characterized by a significant genetic contribution [78]. The downregulation of THOP1 in whole blood and peripheral blood mononuclear cells from patients with RA could result in abnormal antigen presentation, which might contribute to the pathogenesis of RA [78]. These data indicate that THOP is also important in inflammatory diseases. Here, a possible role of THOP1 in sepsis, another inflammatory condition with a high epidemiological burden, was identified. Sepsis is a life-threatening organ dysfunction caused by a deregulated host response to infection which is often accompanied by an intense systemic inflammatory response [79,80]. Sepsis signs and symptoms are quite varied and the organ dysfunction severity can be assessed according to the sequential organ failure assessment [81]. With high incidence and mortality in the early phase of syndrome, a wide range of long-term problems can be associated with sepsis [82]. Nearly half of sepsis survivors have at least three symptoms among insomnia, loss of cognitive function, nightmares, depression, fatigue, and loss of self-esteem [83]. Inflammatory and anti-inflammatory responses and innate and adaptive immune systems are each equally important and represent potential targets for immune therapy to improve sepsis outcomes [81,84,85]. Here, we demonstrated that THOP1^-/-^ mice have slightly better survival and behavior performance seven days after polymicrobial sepsis induction, with slightly lower expression of TLR4 and TNF-α in dorsal hippocampus. These data suggest that THOP1 may be a new therapeutic target for treating sepsis and inflammatory diseases.

THOP1 has also been associated with opioid peptide metabolism and the generation of both Leu- and Met-enkephalins in the murine brain [1,3,27,86,87]. In the hot plate test WT and THOP1^-/-^ mice present similar behavior, suggesting that THOP1 alone is not a key enzyme for the metabolism of opioid peptides in mouse CNS. Enhanced NEP mRNA expression levels were observed in ST and NEP may replace THOP1 in the metabolism of opioid peptides under THOP1 suppression, particularly in that area of the CNS. THOP1^-/-^ mice showed higher sensitivity to the intraplantar administration of bradykinin, and under these conditions their peripheral nociceptive response in the hot plate test was much faster than that of WT mice. These observations indicate that THOP1 is a rate-limiting peptidase for bradykinin inactivation in the periphery. These latter data corroborates previous suggestions that THOP1 plays a key function inactivating bradykinin in the circulation [21], and suggests THOP1 as a pharmacological target for the treatment of certain types of pain.

THOP1^-/-^ mice presented a mild (*p* = 0.05) depression-like behavior in both FST and TST. Further experiments have to be conducted to investigate the pharmacological effects of antidepressants on THOP1^-/-^ depressive-like behavior. THOP1^-/-^ presented deficit of attention and memory retention phenotypes, and lower turnover ratio of 5HIAA/5HT in the PFC and of HVA/DA and DOPAC+HVA/DA in the ST. In the ST and HC of THOP1^-/-^ the mRNA levels for dopamine DRD2 receptors were lower compared to WT. High mRNA levels of serotonin 5HT2a receptors mRNA expression in the HC were observed for THOP1^-/-^ compared to WT. It is well documented that serotonin and dopamine play important functions in neuronal communication and in many psychiatric disorders, including depression and schizophrenia that can affect attention and cognition [88,89]. THOP1 and NLN are oligopeptidases with great similarities in structure [90] and substrate-specificity [25]. Here, NLN mRNA levels were not seen to be altered in any of the brain regions evaluated in THOP1^-/-^. THOP1 and NLN have not been previously associated to the best of our knowledge with a depressive behavior or a deficit of attention and memory retention. The role of NLN in other pathophysiological conditions has been documented lately but still remains unclear [9]. NLN and myosin C gene expression have been shown to be reduced in patients with schizophrenia [91]. These results suggest that THOP1^-/-^ causes unbalance of dopamine and serotonin neurotransmitter turnover and also affect the mRNA expression of their respective receptors. Thus, it is possible that each of these individual changes could be contributing to the overall changes in behavior phenotype alterations observed in THOP1^-/-^ mice.

In conclusion, the present report corroborates the previously anticipated biological significance of THOP1 in neurodegeneration, inflammatory diseases, and peptide metabolism. A role of THOP1 in the regulation of specific mRNA levels, depression-like behaviors, and deficit of attention and memory retention were first suggested herein. Therefore, THOP1 knockout mice reported here for the first time can be a new tool to investigate new drugs for neurodegenerative and inflammatory diseases as well as psychiatric disorders such as depression and deficit of attention.

## 4. Experimental Procedures

### 4.1. Animals

Animals were kept with free access to food and water in a room with controlled temperature (22–23 °C) and in 12 h light/dark cycles with lights on at 6:00 a.m. All experiments were conducted using THOP1^-/-^ or WT, males or females, 4–16 weeks old, raised in our local animal facility at Biomedical Science Institute, Pharmacology Department Unit 2, University of São Paulo, SP, Brazil. The animals were maintained and used in accordance with the guidelines of the National Council for Control of Animal Experiments (CONCEA), following international norms of animal care and maintenance. For each behavioral task, we used a different cohort of animals (naïve) to avoid unreliable results. Thus, we hereby state that all experimental protocols were previously approved by University of São Paulo Ethics Committee Councils from Biomedical Science Institute (approval number for mice experimentation ICB/USP Nº 70/2015).

### 4.2. Generation of THOP1^-/-^

THOP1^-/-^ gene-trap knockout mouse strain was generated by C57BL/6 blastocyst micro-injection of genetically modified embryonic stem cells (CSG163, 129ola) obtained from Baygenomics through the International Gene-Trap Consortium. Briefly, the genetically modified THOP1 allele of the CSG163 strain has the gene-trap vector pGT0Lxf inserted at the intron 5 of the THOP1 gene localized on chromosome 10. These data were confirmed by sequencing 5′RACE cDNA from the original CSG163 ES cell lines used to generate this mouse strain. After transmission of the knockout allele from chimera to F1 generation, THOP1^-/-^ mice were obtained from heterozygous breeding and the line was further maintained with the mixed background by breeding +/− with +/− animals. To obtain such mice with a pure genetic background, we bred F1 (129/OlaHsd/C57BL/6 background) heterozygous THOP1-deficient animals to the inbred C57BL/6 mouse line (Charles River) for at least 10 generations before using them for experimental investigations.

### 4.3. Genotyping

Genotyping was conducted as previously described [92]. Briefly, DNA samples were extracted from the tail of the mice in 50 μL of lysis buffer (100 mM TrisHCl, 5 mM EDTA, 0.2% SDS, 200 mM NaCl) and 8 μg proteinase K, incubated at 55 °C under 300 rpm for 6 h. Subsequently, the mixture was incubated at 95 °C for 10 min without stirring. After this step, 500 μL of buffer containing EDTA (1 mM), Tris-HCl (10 mM, pH 7.5), and 15 μg Rnase A (Purelink Rnase, Invitrogen, Pleasanton, CA, USA) was added and incubated for another 10 min at 37 °C. The genomic DNA samples were obtained and stored at −20 °C before the use. PCRs were conducted using the Amplitaq Gold 360 Master Mix (Applied Biosystems, Foster City, CA, USA) and 2 μL of the genomic DNA samples obtained as described above. The following oligonucleotide sequences were used; pCSGf2 (5′GAGTCGGGACCTTGGAGC3′), pCSGKOR2 (5′TTAAC TATGCGGCATCAGAGC3′), and pCSGwtR4 (5′CACCAGGGAATGAGCCAC3′). The PCR cycles used were 95 °C for 3 min (1×), 95 °C for 15 s, 60 °C for 15 s (40×), 72 °C for 30 s, 72 °C for 7 min (1×), and 4 °C until the end of the reaction. The PCR products were applied to 1.5% agarose gels containing 20 μL of Safe DNA developer (Sybr Safe 10,000×, Thermo Fisher, Waltham, MA, USA), analyzed and digitally stored.

### 4.4. Enzymatic Activity

The enzymatic activity of THOP1 and NLN were determined in triplicates using a continuous assay with a quenched fluorescent substrate ((QFS) 7-met [93] hoxycoumarin-4-acetyl-P-l-G-P-dK-(2,4-dinitrophenyl) as previously described [5]. Pro-Ile (5 mM) was used as a specific inhibitor of NLN, as previously described [21]. The results were expressed as arbitrary units of fluorescence (UAF) per minute normalized by protein concentration.

### 4.5. Blood Count Analysis and Coagulation

Blood aliquots (20 µL) were taken from the tail and diluted in 1 mL of phosphate buffer saline for total blood cell counting with an automatic cell counter (BC2800 Vet Analyzer, BioBrasil, São Paulo, SP, Brazil) [94]. The coagulation tests were performed using a computerized ROTEM^®^ four-channel system (Pentapharm, Munich, Germany), according to the manufacturer’s instructions for the standard nonactivated ROTEM^®^ method (NATEM) during 1 h. Whole blood samples were manually collected into syringes containing 1:10 (v/v) 3.2% trisodium citrate from mice (*n* = 3–4) anesthetized with isoflurane at 2–3% in medicinal oxygen by venous puncture of peripheral blood. Parameters such as clotting time (CT), clot formation time (CFT), alpha angle, maximum clot firmness (MCF), A05 to A30, and maximum lysis (ML) were determined automatically according to previous descriptions [55].

### 4.6. EAE Induction

EAE was induced as previously described [95], with some modifications. Briefly, 24 h before immunization 20 µL of blood was collected from tail and diluted in 1 mL of phosphate buffer saline, pH 7.5, and immediately read in a BC2800 vet analyzer (BioBrasil, São Paulo, SP, Brazil) to quantify white blood cells. On the day of immunization (day 0), WT females were subcutaneously injected with 150 µg of MOG35-55 peptide (Proteimax Biotechnology LTD, São Paulo, SP, Brazil), emulsified in artificial spinal fluid (v/v) with 400 µg heat-killed *Mycobacterium tuberculosis*. Also, 200 ng of *Bordetella pertussis* toxin was administered intraperitoneally (I.P.) twice, at 0 and 48 h postimmunization. The animals were evaluated daily based on clinical scores: 0: without disease; 0.5: loss of tail tonus; 1: hind limb weakness; 2: one hind limb paralyzed; 3: complete hind limbs paralysis; 4: hind limbs paralyzed, weakness in forelimbs; 5: tetraplegia or death. After euthanasia by decapitation on the 26th dpi, dorsal hippocampus and spinal cord were briefly dissected and immediately stored at −80 °C for further analysis.

### 4.7. Sepsis Induction

For all experiments, male C57BL/6 mice (weight, 18–20 g) were used. The animals were housed in cages in temperature-controlled rooms and received water and food ad libitum. Sepsis was induced through CLP as described elsewhere [80,96]. Briefly, animals were anesthetized with isoflurane; the abdominal region shaved and disinfected with iodinated alcohol. Then a laparotomy in the linea alba was performed and cecum exposed and ligated. To induce mild sepsis, one through-and-through puncture with a 21 G needle were performed and animals sutured in two layers. After the procedure, animals where hydrated with 1 mL of 0.9% saline s.c. and kept in warm light until full recovery of the surgery. Sham animals were anesthetized, operated, cecum exposed and repositioned, sutured, hydrated and kept in warm light. During the first 12 h after CLP, the mortality was checked each hour. After that, animals were checked every 12 h for a total of 7 days.

### 4.8. Western Blot Assays

Western blots were conducted as previously described with some modifications [97]. Briefly, electrophoresis was performed using a 10% polyacrylamide gel and the Mini-Protean^®^ Tetra Cell apparatus (BioRad Laboratories, Inc., Hercules, CA, USA). The proteins from 10,000× *g* supernatant were combined with an equal or a quarter part of the supernatant volume with Laemmli’s buffer (BioRad Laboratories, Inc. USA; complemented with 5% mercaptoethanol) and boiled at 95 °C for 5 min. Protein samples (60 µg/lane) were size-separated in 10% SDS-PAGE gel (90 V), and then blotted onto Immobilon^®^ PVDF membrane (EMD Millipore Corporation, Temecula, CA, USA). Ponceau S method was used to ensure equal protein loading on the immunoblot. Blots were blocked with 5% nonfat milk or bovine serum albumin (BSA), diluted in TBS-T buffer (50 mM Tris-HCL, 150 mM NaCl, 0.1% Tween 20, pH 7.5), for 1 h at room temperature and subsequently incubated overnight at 4 °C with specific anti-THOP1 antiserum (1:2000; Proteimax Biotechnology LTDA, São Paulo, SP, Brazil), anti-TNF-α (1:1000; Cell Signaling Technologies, Danvers, MA, USA) and anti-TLR4 (1:1000; Santa Cruz Biotechnology, Dallas, TX, USA) as previously described [34,35]. After incubation with the primary antibody, the membrane was then probed with a secondary antibody conjugated to horseradish peroxidase (dilution of 1:2000; Cell Signaling Technologies, Danvers, MA, USA), for 2 h at room temperature and developed by ECL-Immobilon^®^ reagent (EMD Millipore Corporation, Temecula, CA, USA). Chemiluminescent bands were recorded using ChemiDoc MP Imaging System Detection System and Image Lab software (BioRad Laboratories, Inc., USA).

### 4.9. Quantitative Real-Time PCR (qRT-PCR)

To determine specific mRNA expression in prefrontal cortex, hippocampus, hypothalamus, and striatum were dissected as indicated [98] and quantitative real-time PCR (qRT-PCR) were conducted [99]. Animals were decapitated under inhalant anesthesia with isoflurane and tissues were removed according to coordinates previously described, snap-frozen in liquid nitrogen, and stored in −80 °C until use. Samples were homogenized, and total RNA was isolated using TRIzol^®^. The total RNA was cleaned in a RNeasy mini kit (Qiagen, Germantown, MD, USA), and the RNA integrity was assessed by electrophoresis on 1% agarose gels. cDNA was synthesized from 2 μg of total RNA with High Capacity cDNA Reverse Transcription Kit (Thermo Fisher, Waltham, MA, USA) using random hexamer nucleotides. Standard curves for all primers were calculated to determine their amplification efficiencies. qRT-PCRs were performed on an ABI Prism 7900 (Applied Biosystems, Foster City, CA, USA) sequence detection system with 20 ng of cDNA, 100 nM primers and Fast SYBR™ Green Master Mix (Thermo Fisher Scientific). We analyzed the results from qRT-PCR assays using Pfaffl equation [100]. We calculated the Ct= (Ct of the target gene in WT group − Ct of the target gene in THOP-/- group)/ (Ct of housekeeping gene in WT group − Ct housekeeping gene in THOP-/- group) and, since PCR product is produced exponentially, we transformed the Ct variation ration into fold change using the formula 2^−Ct^. Glyceraldehyde-3-phosphate dehydrogenase (GAPDH) was used as reference gene and we set control group (WT) as 1 in order to show the proportional variation of THOP^-/-^ group compared to WT group. Besides GAPDH, we have also used hypoxanthine guanine phosphoribosyl transferase (HPRT) and ribosomal protein L19 (RPL19) as housekeeping genes, since HPRT and RPL19 have shown some variation in mRNA levels among THOP1^-/-^ and WT (data not shown), GAPDH was what best fulfill housekeeping feature. Primer sequences used herein were as shown below (Table 3).

### 4.10. Global Gene Expression Analysis by Affymetrix GeneChip Mouse Gene 2.0 ST Array Platform

Microarray analyses were conducted to compare gene expression of WT and THOP1^-/-^ using mice Affymetrix (Thermo Fisher Scientific, USA) GeneChip Mouse Gene 2.0 ST Array platform, as previously described [101], and Supplementary Information Microarray Data. Briefly, animals were euthanized by decapitation and the brain was rapidly removed from the skull, and multiple brain regions were dissected (hippocampus, hypothalamus, striatum, and prefrontal cortex) with the aid of an atlas of mouse anatomy. The dissected tissues were frozen with the aid of dry ice and stored at −80 °C until the time of extraction of the RNAs. RNA extraction was performed using the TRIzol^®^ reagent (Thermo Fisher, USA) according to the protocol available from the manufacturer. The homogenizer’s metal stand and stem were previously cleaned with a mixture of 10% Extran^®^ (Merck Millipore, Burlington, MA, USA), 70% ethanol, 0.1 M hydrochloric acid, and RNase. Next, 400 μL of TRIzol^®^ was added to the tube containing the still-frozen tissue, and homogenization was continued until the tissue fragment completely disappeared. At that time, a further 500 μL of TRIzol was simultaneously added in all tubes which were left at room temperature for 5 min when 200μL of chloroform was added and the vortex homogenized for 15 s, followed by standing at environment temperature for 3 min. The samples were then centrifuged at 4 °C for 15 min at 12,000× *g*, when separation occurred in 3 distinct phases. The RNAs were precipitated for 10 min after addition of 500 μL of pure isopropanol at room temperature, followed by centrifugation for 10 min at 4°C at 12,000× *g*. The supernatant was carefully removed and the pellet rinsed with 75% ethanol followed by a further centrifugation step for 5 min 4 °C at 10,000× *g*. The excess ethanol was carefully removed and the RNAs left dehydrating at room temperature when 75 μL of diethyl pyrocarbonate (DEPC)-containing water was added and allowed to incubate for 10 min at 60 °C, and then on ice at approximately 20 min when the samples were stored in a freezer at −80 °C until the moment of use. The integrity of the RNAs was first evaluated by agarose gel electrophoresis observing the presence of bands for 28S and 18S ribosomal RNA. In order to further guarantee the integrity of the samples, the integrity of the RNAs was monitored by capillary electrophoresis (Bioanalyser Agilent, Santa Clara, CA, USA). The purification of messenger RNAs (mRNAs) was performed by the silica method, using the Qiagen kit (RNeasy; Qiagen, Germantown, MD, USA). Subsequently, the samples were prepared for hybridization according to the manufacturer’s guidelines using the Ambion kit (Affymetrix^®^ GeneChip^®^ Whole Transcript Expression Arrays; Affymetrix; Thermo Fisher, Waltham, MA, USA). Samples were hybridized to GeneChip^®^ and subjected to final wash of the arrays as instructed by the manufacturer (Affymetrix; Thermo Fisher, Waltham, MA, USA). Data were collected and analyzed using specific software (TAU GC Bioinformatics, SP, Brazil; Microarray Supporting Information). Differential gene expression analyses were performed in specific areas of the CNS such as, hippocampus, hypothalamus, striatum and prefrontal cortex. For microarray data analyses we used LIMMA method, considering differences from WT/THOP1^-/-^ that were ≥1 and *p* ≤ 0.001.

### 4.11. Peptide Extraction

Peptide extracts were prepared from WT or THOP1 mice as previously described [102]. Briefly, mice were sacrificed by decapitation and the head was immediately irradiated in a conventional microwave oven for 8 s (1500 watts, ELECTROLUX) to inactivate protein and peptide degradation. The brains were rapidly removed from the skull and the hippocampus, striatum and cortex areas were collected separately. For each sample, structures from four animals were used. The samples were sonicated twice for 20 s at 1 pulse/s in 1 mL ice-cold water using an ultrasonic processor (Fisher Scientific, Lenexa, KS, USA). The homogenates were incubated at 80 °C in water bath for 20 min and then cooled on ice and acidified with 0.1 M HCl to a final concentration of 10 mM HCl. The homogenates were centrifuged at 13,000 g for 40 min at 4 °C and the supernatants were collected and filtered through Millipore membrane (MCWO 10000—Amicon Ultra, EMD Millipore Corporation. Billerica, MA, U.S.A.) to remove proteins larger than 10 kDa. The flow-through was applied to C18-like Oasis columns (Waters, Milford, CT, USA), eluted with 100% methanol/0.15% trifluoroacetic acid. The peptide extracts were resuspended in 100 μL of deionized water and kept at −80 °C before used.

### 4.12. Peptide Quantification

The peptide concentration was determined using fluorescamine, at pH 6.8, as previously described [103]. Briefly, 2.5 μL of each sample was mixed with 25 μL of 0.2 M phosphate buffer (pH 6.8) and 12.5 μL of a 0.3 mg/mL fluorescamine solution in acetone. After vortexing for 1 min, 110 μL of water was added and fluorescence was measured with a SpectraMax M2e plate reader (Molecular Devices, San Jose, CA, USA) at an excitation wavelength of 370 nm and an emission wavelength of 480 nm. The peptide 5A (LTLRTKL), of known composition and concentration, was used as the standard reference for determining the peptide concentration [93].

### 4.13. Dimethyl Isotopic Labeling for Peptide Semi-Quantitative Analyses

Peptide samples were labeled as previously described [45,93,104]. The labeling method employed is based on the dimethylation of amine and formaldehyde groups in the presence of cyanoborohydride. Here, two isotopic forms were used and the final product of these reactions adds 28 or 32 Da to the final mass of peptides at each available (lysine or N-terminal) labeling site, which can be observed in the MS spectrum. The labeling schemes for each brain are summarized in Supplementary Information—MS Data. Briefly, 5 μg of peptide extract was diluted in 100 μL TEAB buffer to a final concentration of 100 mM. In the hood, 4 μL of the different isotopic forms of the formaldehydes were added at a concentration of 4% according to the desired labeling scheme. Then, 4 μL of 0.6 M NaBH 3 CN was added and the samples incubated for 3 h at room temperature. After this time, the reaction was quenched with 16 µL of 1% ammonia. Finally, 8 µL formic acid was added and the two differentially labeled samples were pooled, desalted using C18 columns (OASIS; Waters, Milford, MA, USA), and eluted with 100% acetonitrile containing 0.15% trifluoroacetic acid (TFA). The samples were dried in a vacuum centrifuge and stored at −20 °C.

### 4.14. Liquid Chromatography (LC) and Mass Spectrometry (MS)

LC-MS analyses were performed by online liquid chromatography in an Easy-nLC II nanoHPLC system coupled to an LTQ-Orbitrap Velos (Thermo Fisher Scientific, Bremen, Germany) through a nanoelectrospray ion source. Separation was carried out in a column (ID 360 μm OD × 100 micron) packed in-house with 5-µm Jupiter^®^ C-18 beads (Phenomenex, Torrance, CA, USA). Peptides were eluted with a linear gradient of 5 to 45% acetonitrile, in 0.1% formic acid, for 90 min at 200 nL/min. The data were automatically acquired after the generation of multiple peptides protonated by the ESI (electrospray ionization), followed by the dissociation MS/MS (Top 10) by the collision with nitrogen (CID) in an intensity of 10 to 30 eV, 2.3 kV. The injection time on the ion-trap is fixed at 100 ms and the FT-MS injection with a resolution of 1000 ms, 30,000 at m/z 300–1800. Fragmentation scanning was performed with a minimum of 5000 counts and a dynamic exclusion in 70 s. The RAW extension files were analyzed using the Mascot softwares (using the SwissProt database) and Xcalibur. The identified peptides were selected according to criteria that should appear in at least 2 replicates of each region and analyzed condition.

### 4.15. MS/MS Data Analyses

To identify peptides, MS/MS data were analyzed using the Mascot search engine (Matrix Science Ltd., London, UK). Briefly, the raw files generated by the mass spectrometer were converted to text format files (mgf) by the Mascot Distiller program version 2.1.1 (Matrix Science Ltd., UK) and submitted to the Mascot Search Database searched included mouse SwissProt. No cleavage site was specified. Modifications included the reductive dimethyl labels, N-terminal protein acetylation and methionine oxidation. Mascot searches were followed by manual interpretation to eliminate false positives. Several criteria were used to accept or decline the peptides that were identified by MASCOT: (1) the majority (>80%) of the major MS/MS fragment ions matched predicted *a*, *b*, or *y* ions; (2) a minimum of five fragments ions matched *b* or *y* ions; and (3) the number of tags incorporated into the peptide matched the number of free amines (N terminus and side chains of Lys). Quantification was performed by measuring the ratio of peak intensity for the various Dimethyl labeled peptides pairs in the MS spectra. For these analyses, the monoisotopic peak and the peaks containing one and two atoms of ^13^C were used. Multiple scans of the MS spectra were combined prior to quantification.

### 4.16. Animal Behavior Tests

The behavioral tests used (hot plate, open field, forced swimming, high cross maze, object recognition, passive avoidance, Barnes labyrinth, and prepulse inhibition) were used to evaluate phenotypic changes in THOP1^-/-^ compared to WT mice, as previously described [105,106,107,108,109,110,111,112,113,114,115,116,117,118].

### 4.17. Hot Plate

In the hot plate test [119], mice were placed on a metal surface kept at 50 °C. The time interval between placement and licking of both anterior feet was recorded as response latency. Animals were at different time points, and a 30 s cutoff was used to minimize tissue damage. Results were analyzed by comparing the difference between pre- and post-treatment/experiment by two-way ANOVA followed by Bonferroni’s multiple comparison test.

### 4.18. Forced Swim Test

This test is aimed to evaluate depression symptoms in animals and is divided into 2 phases: training and testing. In the training, the animals were individually placed in a cylindrical tube (25 cm high) containing water up to 16 cm high, under a temperature of 25 °C, for 15 min. After 24 h, the test was performed, where the animals were submitted to the same training conditions, but for 5 min. The animals were filmed and evaluated during both training and test phases [108]. During the test, the latency time that each animal took to become immobile for the first time, as well as the time of immobility of the animal in a period of 5 min was evaluated.

### 4.19. Tail Suspension Test

Tail suspension testing was conducted as previously described [109,110]. The tail suspension test is based on the observation that a mouse suspended by the tail shows alternate periods of agitation and immobility The mouse, acoustically and visually isolated, was hung on the hook by an adhesive tape placed 20  mm from the extremity of its tail and it was kept 150 mm away from the nearest object. Both the latency and immobility time were manually registered by an observer who was previously unaware of the mice phenotype. Each mouse (*n* = 8–14) was used only once for each experimental session. Like the FST, the TST is based on the observation that rodents (almost always mice) after initial escape-oriented movements, develop an immobile posture when placed in an inescapable stressful situation. In the case of the tail suspension test the stressful situation involves the hemodynamic stress of being hung in an uncontrollable fashion by their tail whereas in the forced swim test mice were placed in a cylinder filled with water [111].

### 4.20. Passive Avoidance Test (Step-Down)

Step-down tests were conducted as previously described [119]. Briefly, the apparatus consists of a rectangular metal box (40 × 30 cm), one of the walls being acrylic and transparent, with the floor surface formed by steel rods. In one corner of the box there is a leakage area (200 mm × 75 mm). During the test, a low-power shock is emitted, so that the entire surface of the ground is reached, except for only the rectangular platform. During 5 min of training, the mice were placed on the platform. The animals that came down from the platform and touched, with their four legs, the ground possessing the steel rods, received an electric shock at the power of 0.6 mA for 2 s, and were removed from the apparatus. The maximum training time was 300 s. After 1 h, in order to analyze short-term memory, the animals were tested with the same procedure, but without electric shock. The latency time of the descent of the animal from the platform to the ground was counted, with a maximum time of 300 s. The test was repeated after 24 h for long-term memory evaluation. After each test, the apparatus was cleaned with 70% ethanol.

### 4.21. Prepulse Inhibition Test (PPI)

PPI test is a widely used model for detecting sensorimotor gating deficits in humans and animals, similar to those observed in schizophrenia [120,121]. This paradigm consists on a reduction of the startle reaction to a stimulus when it is immediately preceded by a low-intensity stimulus called prepulse. The PPI deficit is one of the most used paradigms in animal models of schizophrenia, since there is a similarity of the phenomenon in different species, which favors its use in translational approaches [122]. Before the start of the test, a calibration of the platforms was performed to ensure an equivalence in sensitivity throughout the experiment in all 4 boxes for acoustic startle response evaluation. This calibration was done by adjusting the gain using a standard weight (50 g) appropriate for the mouse. The animals were placed in a containment cage (80 × 90 × 255 mm) suspended inside a PVC structure connected by four screws to the platform with a stabilizer, which captures the startling response of the animal. The cage and platform are enclosed in a ventilated soundproof enclosure (760 × 485 × 705 mm). The startle reaction of the mice generates a pressure on the stabilizer and analog signals are amplified, scanned and analyzed by software of the SS measurement system (Insight^®^, São Paulo, Brazil). This system also controls other test parameters (acoustic stimulus intensity, interval between stimuli). Two loudspeakers were used as sources of sound stimuli. The test consists of placing the animals in the startle response measurement boxes with a sound seal in the presence of a constant background sound of 65 decibels (dB). After 5 initial min of habituation, the session is started. During the session, three types of stimuli are presented, pseudorandomly distributed in intervals of 20 s in average: (i) 20 pulses (P) of 120 dB for 40 milliseconds (ms) (capable of producing a startle response), (ii) 10 presentations of each prepulse intensity (PP) (75, 80 and 85 dB by 20 ms) 100 ms before the pulse, and (iii) 10 nonstimulus presentations. At the beginning of each session, 10 pulses (120 dB per 40 ms) are presented for the habituation of the animals to this stimulus. This series is not considered for PPI calculations. The percentage of prepulse startle inhibition (PPI) induced by each of the three prepulse intensities was calculated as PPI = [100 − 100 (PP)/(P)].

### 4.22. Open Field

Open field testing is one of the most widely used tests in animal behavior studies [123]. Besides its use for global locomotor activity, the test also allows for measuring exploratory activity and anxiety. The test is performed in an acrylic box in which the floor surface is divided into 9 virtual quadrants of equal size. During the test, the number of quadrants taken by the animal, number of rearing, number of grooming movements, and the number of times the animal traverses the central quadrant [115]. For preliminary investigations of general locomotor activity, a 5 min test is sufficient to evaluate gross abnormalities in locomotion; therefore, in this context, the animals submitted to the test were observed during a period of 5 min. The test was performed under adequate lighting conditions, standard light, according to the circadian cycle of the animal. After the test, between the use of one animal and another, the apparatus was cleaned with 70% ethanol, in order to remove odors that can serve as olfactory “tracks”.

### 4.23. Elevated Plus Maze

This test aims to evaluate the behavior of anxiety and is often used in the phenotypic characterization of knockout and transgenic animals. The apparatus for carrying out the test consists of 2 closed arms and 2 open arms (30 × 5 cm) crossed in the middle, perpendicular to each other, and a central area (5 × 5 cm) raised 1 m from the ground. The mice had access to all arms and were able to move freely between them [124]. The animals were placed individually in the central quadrant, facing the open arm, and were free to explore the environment for 5 min. The time of residence in the different compartments, the number of entries in the closed and open arms, as well as the number of dives (head dipping) were analyzed. After the tests the apparatus was cleaned with 70% ethanol before receiving the next animal.

### 4.24. Barnes Maze

The Barnes maze test resembles both the Morris water maze and radial maze, but it takes advantage of the superior rodent abilities to find and escape through small holes. In addition, the Barnes maze presents advantages for behavioral phenotyping. In contrast to the T maze and radial maze, food restriction is not necessary. In comparison with the Morris water maze, the stress component is lower on dry land, as in the Barnes labyrinth [125,126].

The rationale of this test is to evaluate learning and spatial memory. The apparatus consists of a circular platform (92 cm in diameter) elevated 105 cm above the ground, with 20 equally spaced holes of 5 cm in diameter and 7.5 cm between the holes. A small black box of acrylic (leakage box) of 10 × 5 × 7 cm was placed under one of the holes. The view from the center of the apparatus is the same for all holes, so the escape box is not directly seen. Different spatial reference cues were placed on the wall, close to the apparatus, in the behavior room and were not modified during the study.

The test was divided into 3 phases: (i) Adjustment period: when the animal was placed in the center of the apparatus, covered by a white square box (15 cm high), after 10 s, a metronome and the light directly on the apparatus were connected, and the animal was gently guided to the target hole, which contained the escape box. Once the animal entered the box, the metronome and light were turned off, and the animal remained in the escape box for 2 min. (ii) Period of spatial acquisition: this occurred between days 1 to 4, in which the training was performed, and each animal was placed in the center of the apparatus where all the procedures described in the adaptation period were performed. However, at this stage, the animals were not guided to the target hole, but were able to explore the maze freely. The session was ended when the animal found the target hole and entered the escape box. When the animal found the escape box, the light and the metronome were turned off, and the animal was left in the box for 1 min in the dark. If the animal remained exploring the maze after 3 min, it was led to the escape box and left for 1 min. (iii) Barnes maze test: applied on the 5th day, 24 h after the last training. The same procedure was carried out, except that the target hole was closed. After 90 s, the test was performed in order to analyze if the animal remembered where the escape box was located. On the day of the test, 4 test sessions were performed, and the final result was the mean of the last four tests.

During training, the number of total errors (number of wrong holes visited) and latency time (time acquired to find the target hole) were counted as a learning measure. The error was standardized when an animal “dipped” its head into a hole that did not contain the escape box. Several consecutive head “dips” in the same hole were considered as only 1 error. The task is based on the innate preference of rodents for dark and closed spaces as opposed to open areas. Mice are presumed to learn the location of a “leak-hole” using spatial reference points that are fixed relative to the labyrinth (extra-labyrinth tracks) or in the labyrinth itself [126].

### 4.25. Novel Object Recognition Test

The novel object recognition task is very useful to study short-term memory, intermediate-term memory, and long-term memory, through manipulation of the retention interval, i.e., the amount of time animals must retain memory of the sample objects presented during the familiarization phase before the test phase, when one of the familiar objects is replaced by a novel one [127]. The apparatus consists of a simple box with milky white walls, and objects used were made of metal or glass, which facilitated cleaning between sessions with different animals. The test was divided into 3 phases: (i) Habituation phase: before starting the training, the animals were exposed to the box (without the objects) for 10 min, in order to habituate the animal to the apparatus. (ii) Exposure phase: the animals, in turn, were placed in the box with 2 identical objects (A and B) arranged 15 cm away from the wall of the apparatus. The animals were observed for 10 min and then removed and returned to their respective cages. (iii) Test phase: one object already known (B) is replaced by a new object (C) 1 h and 24 h after the training. The animals were observed for 5 min. The exploratory activity was defined as the time the animal spent smelling, licking, touching the new object with the nose or front paws or even when the animal returned the snout to the object at a radius less than or equal to 1 cm. After the tests the apparatus was cleaned with 70% ethanol before receiving the next animal minimizing possible olfactory cues.

### 4.26. Quantitation of Neurotransmitters and Their Metabolites in Specific Areas of Mice Brain

Mice were euthanized and the brain was rapidly dissected on ice. ST, HC, and PFC were isolated, weighed, and rapidly stored in liquid nitrogen. For neurochemical analyses tissues were homogenized in 0.1 M perchloric acid by manual sonication, centrifuged at 10,000 rpm for 20 min to remove the supernatant, and stored at −80 °C until the determination of monoamine levels. The levels of neurotransmitters and their metabolites (dopamine (DA), 3,4-dihydroxyphenylacetic acid (DOPAC) and homovanillic acid (HVA), and serotonin (5HT), 5-hydroxyindoleacetic acid (5HIAA), and noradrenaline (NOR) were measured by reversed-phase high-performance liquid chromatography (HPLC) using a system (model 6A, Shimadzu, Kyoto, Japan) with an electrochemical detector, as described elsewhere [128,129]. Briefly, 20 μL samples were loaded into a sample injector, and the mobile phase was delivered at a constant rate of 1.2 mL/min. The runtime for each sample was 15 min, and the concentrations of all neurotransmitters and their metabolites were expressed as nanograms per gram of tissue (ng/g).

### 4.27. Statistical Analysis

All results are expressed as the means ± standard error of the mean (SEM). The statistical comparisons were performed using Student’s *t*-test and/or analysis of variance (ANOVA) and/or two-way ANOVA followed by post hoc Tukey’s test. Probability less than 0.05 was considered as statistically significant (*p* < 0.05). Data were statistically analyzed with GraphPad Prism software (GraphPad Software Inc, San Diego, CA, USA).

## Figures and Tables

**Table 1:** Relative quantification of peptides from prefrontal cortex (PFC), striatum (ST), or hippocampus (HC).

Protein Name	Peptide Sequence		Average Ratio	Average Ratio	Average Ratio
		Location	THOP1/WT PFC	THOP1/WT ST	THOP1/WT HC
			±SE (n)	±SE (n)	±SE (n)
Acyl-CoA-binding protein	ATVGDVNTDRPGLLDL	C		1.04 ± 0.32 (3)	**1.80 ± 0.66 (3)**
Acyl-CoA-binding protein	GDVNTDRPGLLDL	C			**1.90 ± 0.58 (3)**
Acyl-CoA-binding protein	KQATVGDVNTDRPGLLDL 2+	C		1.11 ± 0.26 (3)	**2.50 ± 1.17 (3)**
Acyl-CoA-binding protein	KQATVGDVNTDRPGLLDL 3+	C	**0.46 ± 0.12 (3)**	1.03 ± 0.27 (3)	**2.20 ± 0.96 (3)**
Acyl-CoA-binding protein	SHFKQATVGDVNTDRPGLLDL	C		1.36 ± 0.39 (3)	
Acyl-CoA-binding protein	TVGDVNTDRPGLLDL	C		1.07 ± 0.31 (3)	**2.60 ± 1.33 (3)**
Acyl-CoA-binding protein	VEKVDELKKKYGI 2+	C	**0.4 ± 0.01 (2)**		**2.3 ± 0.90 (3)**
Acyl-CoA-binding protein	VEKVDELKKKYGI 3+	C	**0.4 ± 0.10 (2)**	0.91 ± 0.14 (3)	**2.07 ± 0.73 (3)**
ATP synthase-coupling factor 6	KFDDPKFEVIDKPQS	M			1.12 ± 0.26 (2)
Cerebellin-4	AANSKVAFSAVRSTN	V	1.12 ± 0.11 (3)		
Cerebellin-4	SKVAFSAVRSTN	V			0.89 ± 0.21 (3)
Clathrin coat assembly protein AP180	ASKGLGSDLDSSLAS (1.52 ± 0.12)	M/C	**0.53 ± 0.11 (3)**	1.08 ± 0.12 (3)	1.05 ± 0.04 (3)
Clathrin coat assembly protein AP180	KGLGSDLDSSLASL (1.54 ± 0.16)	M/C	**0.56 ± 0.14 (3)**		
Clathrin coat assembly protein AP180	SKGLGSDLDSSLASL	M/C	**0.55 ± 0.12 (3)**		
Clathrin coat assembly protein AP180	SPSPTPATQSPKKPPAKDPLADLNIKDFL	M/C	**0.51 ± 0.12 (3)**	1.17 ± 0.18 (3)	0.99 ± 0.06 (3)
Cysteine and glycine-rich protein 1	GQGAGALVHSE	N		1.08 ± 0.02 (3)	**2.17 ± 0.64 (3)**
Cytochrome b-c1 complex subunit Rieske	ARSGPFAPVLSAT	M	0.81 ± 0.19 (3)		
Cytochrome b-c1 complex subunit Rieske	GLNVPASVRF	M	0.94 ± 0.21 (3)		
Cytochrome b-c1 complex subunit Rieske	SGQAAARPLVA	M	0.86 ± 0.21 (3)		1.01 ± 0.10 (3)
Cytochrome b-c1 complex subunit Rieske	SGQAAARPLVATV	M	0.75 ± 0.14 (3)	1.25 ± 0.20 (3)	0.94 ± 0.08 (3)
Cytochrome b-c1 complex subunit Rieske	TVGLNVPASVRF	M	0.95 ± 0.17 (3)	1.17 ± 0.16 (3)	0.98 ± 0.13 (3)
Cytochrome c oxidase subunit 5A	GISTPEELGLDKV	M	**0.49 ± 0.06 (3)**	1.05 ± 0.19 (3)	**1.51 ± 0.34 (3)**
Cytochrome c oxidase subunit 5A	NDFASAVRILEV (1.43 ± 0.62)	M	**0.36 ± 0.12 (2)**		**1.70 ± 0.38 (3)**
Cytochrome c oxidase subunit 5A	RPTLNELGISTPEELGLDKV	M	**0.42 ± 0.12 (3)**	1.20 ± 0.25 (3)	**1.53 ± 0.37 (3)**
Cytochrome c oxidase subunit 6A1	FHNPHVNPLPTGYEDE	M		1.09 ± 0.13 (3)	1.12 ± 0.14 (3)
Cytochrome c oxidase subunit 6A1	HNPHVNPLPTGYEDE	M	0.86 ± 0.13 (3)	1.13 ± 0.36 (3)	1.30 ± 0.20 (3)
Cytochrome c oxidase subunit 6A1	NPHVNPLPTGYEDE	M			1.07 ± 0.18 (3)
Gamma-enolase	AGNSDLILPVPAFNVINGGSHAGNKL	C			0.76 ± 0.20 (3)
Glyceraldehyde-3-phosphate dehyd.	AFRVPTPNVSVVDL	C/N		**1.51 ± 0.09 (3)**	0.99 ± 0.09 (3)
LIM zinc-binding domain-containing Nebulette	TQVVSDAAYKGVQPHVV	C	**0.59 ± 0.06 (3)**		0.93 ± 0.22 (3)
Macrophage migration inhibitory factor	AQATGKPAQYIA	C	0.92 ± 0.12 (3)		
Macrophage migration inhibitory factor	AQATGKPAQYIA	C			1.17 ± 0.02 (2)
Macrophage migration inhibitory factor	AQATGKPAQYIAVHVVPDQL 2+	C	0.65 ± 0.03 (3)		0.87 ± 0.07 (3)
Macrophage migration inhibitory factor	AQATGKPAQYIAVHVVPDQL 3+	C	**0.59 ± 0.12 (3)**	1.17 ± 0.07 (3)	0.75 ± 0.15 (3)
Microtubule-associated protein 2	AEDVTAALAKQGL	C			0.87 ± 0.06 (3)
Microtubule-associated protein tau	ADEVSASLAKQGL	C	0.78 ± 0.24 (3)	1.17 ± 0.16 (3)	0.95 ± 0.12 (3)
Neurogranin	GRKGPGPGGPGGAGGARGGAGGGPSGD	C	**0.60 ± 0.10 (3)**	0.84 ± 0.03 (3)	1.08 ± 0.30 (3)
Neurogranin	KGPGPGGPGGAGGARGGAGGGPSGD 2+	C	**0.55 ± 0.20 (3)**	0.64 ± 0.02 (3)	**1.58 ± 0.37 (3)**
Neurogranin	KGPGPGGPGGAGGARGGAGGGPSGD 3+	C		0.78 ± 0.01 (3)	**1.63 ± 0.37 (3)**
Neurogranin	RKGPGPGGPGGAGGARGGAGGGPSGD	C	0.94 ± 0.20 (3)	1.28 ± 0.55 (3)	**1.57 ± 0.38 (3)**
Peptidyl-prolyl cis-trans isomerase A	ADDEPLGRVSF	C	0.85 ± 0.14 (3)	1.10 ± 0.02 (3)	
Peptidyl-prolyl cis-trans isomerase A	ADKVPKTAENF (1.59 ± 0.21)	C	0.71 ± 0.14 (3)		
Peptidyl-prolyl cis-trans isomerase A	ADKVPKTAENFR	C		1.36 ± 0.30 (2)	
Peptidyl-prolyl cis-trans isomerase A	ADKVPKTAENFRAL	C	0.77 ± 0.08 (3)	1.26 ± 0.20 (3)	
Peptidyl-prolyl cis-trans isomerase A	EDENFILKHTGPGILSM	C	0.80 ± 0.10 (3)		
Peptidyl-prolyl cis-trans isomerase A	ELFADKVPKTAENF	C		1.19 ± 0.23 (2)	
Peptidyl-prolyl cis-trans isomerase A	ELFADKVPKTAENFRAL	C	0.98 ± 0.35 (3)		
Peptidyl-prolyl cis-trans isomerase A	ITADDEPLGRVSF	C	0.66 ± 0.16 (3)		0.88 ± 0.13 (3)
Peptidyl-prolyl cis-trans isomerase A	LFADKVPKTAENFRAL	C	0.93 ± 0.15 (3)		
Peptidyl-prolyl cis-trans isomerase A	FDITADDEPLGRVSF	C			1.03 ± 0.22 (3)
Peptidyl-prolyl cis-trans isomerase A	LFADKVPKTAENF	C			0.96 ± 0.22 (3)
Peptidyl-prolyl cis-trans isomerase FKBP1A	VFDVELLKLE	C	0.84 ± 0.13 (3)		1.03 ± 0.22 (3)
Phosphatidylethanolamine-binding protein 1	AGVTVDELGKVL	C		1.11 ± 0.24 (2)	
Phosphatidylethanolamine-binding protein 1	AGVTVDELGKVLTPTQV	C		1.40 ± 0.04 (3)	0.81 ± 0.11 (3)
Phosphatidylethanolamine-binding protein 1	DDYVPKLYEQLSGK	C	0.67 ± 0.08 (3)	1.11 ± 0.17 (3)	0.79 ± 0.06 (3)
Phosphatidylethanolamine-binding protein 1	DGLDPGKLYTL	C			1.06 ± 0.20 (3)
Phosphatidylethanolamine-binding protein 1	GLDPGKLYTL	C			0.77 ± 0.15 (3)
Phosphatidylethanolamine-binding protein 1	GVTVDELGKVLTPTQV	C		1.22 ± 0.20 (3)	
Phosphatidylethanolamine-binding protein 1	KGNDISSGTVL	C			1.10 ± 0.12 (3)
Phosphatidylethanolamine-binding protein 1	KGNDISSGTVLSDYVGSGPPSGTGL	C		1.38 ± 0.04 (2)	
Proenkephalin-A	SPQLEDEAKELQ	V		0.69 ± 0.25 (3)	0.83 ± 0.20 (3)
ProSAAS	ASAPLVETSTPLRL	V		1.20 ± 0.22 (2)	
ProSAAS	SLSAASAPLVETSTPLRL	V	0.92 ± 0.08 (3)	1.20 ± 0.32 (3)	0.84 ± 0.16 (2)
Secretogranin-1	LLDEGHYPVRESPIDTA	V		**1.71 ± 0.15 (3)**	
Secretogranin-1	SFARAPQLDL	V		1.33 ± 0.33 (3)	0.85 ± 0.31 (3)
Somatostatin	ANSNPAMAPRE (methionine oxidation)	V			**1.73 ± 0.13 (3)**
Somatostatin	SANSNPAMAPRE	V	**1.65 ± 0.13 (3)**	**2.03 ± 0.65 (3)**	1.10 ± 0.31 (3)
Somatostatin	SANSNPAMAPRE (methionine oxidation)	V	**2.03 ± 0.47 (2)**	**1.84 ± 0.52 (3)**	
Synapsin-1	AGGPPHPQLNKS	C		0.97 ± 0.29 (3)	
Tubulin beta-2A chain	SGPFGQIFRPDNF	C		1.07 ± 0.22 (3)	0.80 ± 0.02 (3)
Tubulin beta-3 chain	DDEESEAQGPK	C	**2.23 ± 0.57 (2)**		**1.60 ± 0.24 (2)**

Peptides shaded in gray were previously found in Nln knockout mice (NLN^-/-^); the number in parenthesis indicate the relative levels of the peptide in whole brain extracts from NLN^-/-^ compared to WT mice [54]. C. cytosol; M. mitochondria; N. nucleus; V. vesicles. Numbers in bold indicate changes (bigger than 1.5 or smaller than 0.5) in relative peptides level from WT and THOP1^−/−^.

**Table 2:** Complete blood count for wild type (WT) and THOP1^-/-^ animals.

	WT	THOP^-/-^
Granulocytes (%)	21.02 ± 0.63	24.22 ± 0.55 **
Monocytes (%)	6.62 ± 0.17	7.47 ± 0.27 *
Lymphocytes (%)	72.46 ± 0.62	68.32 ± 0.80 **
Platelets 10^9^/L	1509.00 ± 73.12	1159.00 ± 108.50 *
Hemoglobin g/L	140.20 ± 1.74	135.30 ± 3.57
WBC 10^9^/L	10.09 ± 1.00	8.64 ± 0.71
RBC 10^12^/L	9.62 ± 0.10	9.25 ± 0.28
RDW	12.80 ± 0.21	13.12 ± 0.29
MCV fL	46.78 ± 0.23	47.37 ± 0.26
MCHC g/L	311.22 ± 0.89	308.50 ± 1.54

White blood cells (WBC). Red blood cells (RBC). Red cell distribution width (RDW); Mean corpuscular volume (MCV); Mean corpuscular hemoglobin concentration (MCHC). *. *p* ≤ 0.05; **. *p* ≤ 0.001.

**Table 3:** Primer sequences.

Gene	Sequence	Amplicon (bp)	Accession Number
Neurolysin (Nln)	Fwd: CACCCTATCACAGACGAGCT Rev: GGGACTGGTCAACTTTGCTC	106	NM_029447
Neprilysin (NEP)	Fwd: CCTGAACTTTGCCCAGGTGT Rev: GCGGCAATGAAAGGCATCTG	148	NM_001289462.1
Angiotensin I Converting Enzyme 1 (ACE1)	Fwd: ACCCTAGGACCTGCCAATCT Rev: CGTGAGGAAGCCAGGATGTT	164	NM_207624.5
Prolyl oligopeptidase (POP)	Fwd: GGGTGCTCCGACACTAAACA Rev: GACGGGTACTGGATGTCGTC	98	NM_011156.3
Insulin Degrading enzyme (IDE)	Fwd: GTCCATGTTCTTGCCAGGGA Rev: TTCACGAGGGGAAACAGTGG	161	NM_031156.3
Dipeptidyl peptidase 4 (DPP4)	Fwd: GACGGCAGAGGAAGTGGTT Rev: CGCTTGCTATCCACAAATCCC	134	NM_010074.3
Proteasome subunit beta 5 (ProtB5)	Fwd: CCAAACTGCTCGCTAACATGG Rev: GTTCCCCTCGCTGTCTACG	119	NM_011186.1
5-Hydroxytryptamine (serotonin) receptor 2A (5-HT2A)	Fwd: GTTTCCTTGTCATGCCCGTG Rev: CCAGGTAAATCCAGACGGCA	93	NM_172812.2
Dopamine D2receptor (DRD2)	Fwd: ATGGGAGTTTCCCAGTGAACA Rev: ATGGGGCTATACCGGGTCC	115	NM_010077.2
Hypoxanthine guanine phosphoribosyl transferase (HPRT)	Fwd: TGCTGACCTGCTGGATTACA Rev: TTTATGTCCCCCGTTGACTGA	120	NM_013556.2
Ribosomal protein L19 (RPL19)	Fwd: CAATGCCAACTCCCGTCA Rev: GTGTTTTTCCGGCAACGAG	102	NM_009078.2
Glyceraldehyde-3-phosphatedehydrogenase (GAPDH)	Fwd: GTGCAGTGCCAGCCTCGTCC Rev: CAGGCGCCCAATACGGCCAA	75	BC085275.1

## Supplementary Materials

The following are available online at https://www.mdpi.com/2218-273X/9/8/382/s1. Figure S1: Open field task; Figure S2: Elevated plus maze task; Figure S3: Novel object recognition task. Microarray data; Individual MS identification and quantitation; MS supplementary information; TNF-α crude Western blots; THOP1^-/-^ generation.
