# Supplementary material for: Thimet Oligopeptidase (EC 3.4.24.15) Key Functions Suggested by Knockout Mice Phenotype Characterization
Source: Biomolecules. 2019 Aug 19;9(8):382. doi: 10.3390/biom9080382 (PMC6722639; doi:10.3390/biom9080382)
Supplement: Supplementary file 1 [file biomolecules-09-00382-s001.zip › Microarray data Supp.pdf]

Thimet oligopeptidase (EC 3.4.24.15) key functions suggested by knockout mice phenotype characterization

Dos Santos, N.B.[1#], Franco, R.D.[1#], Camarini, R.[1], Munhoz, C.D.[1], Eichler, R.A.S.[1], Gewehr, M.C.F. [1], Reckziegel, P.[1], Llanos, R.P.[1], Dale, C.S. [2], Oliveira, V.R. [2], Borges, V.F.[3], Lima, B.H.F.[3], Cunha, F.Q.[3], Visniauskas, B.[4], Chagas, J.R.[4], Tufik, S.[4]., Peres, F.F.[5], Abilio, V.C.[5], Florio, J.C.[6], Iwai, L.K.[7], Rioli, V.[7], Prezoto, B.C.[8], Guimaraes, A.O.[9], Pesquero, J.B.[9], Bader, M. [10,11,12,13,14], Castro, L.M.[15], and Ferro, E.S.[1*]

Department of [1]Pharmacology and [2]Anatomy, Biomedical Sciences Institute, and [6]Department of Pathology, Veterinarian Medical School, University of São Paulo (USP), São Paulo, 05508-000; [3]*Department of Pharmacology*, *Faculty* of *Medicine* of *Ribeirao Preto, University of São Paulo, Ribeirão Preto, SP,* 14049-900; Departments of [4]Psychobiology, [5]Pharmacology and [9]Biophysics, Federal University of São Paulo (UNIFESP), São Paulo, 04023-062; [7]Special Laboratory of Applied Toxinology (LETA), Center of Toxins, Immune Response and Cell Signaling (CETICS), [8]Pharmacology Department, Butantan Institute, 05503-000, São Paulo;[10]Max-Delbrück-Center for Molecular Medicine, D-13125, Berlin, Germany, [11]Charité - Universitätsmedizin Berlin, [12]Berlin Institute of Health (BIH), Berlin, Germany, [13]DZHK (German Center for Cardiovascular Research), Partner Site Berlin, Berlin, Germany, [14]Institute for Biology, University of Lübeck, Lübeck, Germany, [15]Biosciences Institute, São Paulo State University (UNESP), São Vicente, 11330-900; SP, Brazil.

Running Title: THOP1 knockout mice

***To whom correspondence should be addressed:** Emer S. Ferro, PhD, Professor, Av. Prof. Lineu Prestes 1524, Sala 317, São Paulo, SP, 05508-000, Brazil, Phone: +55-11-3091-7310, Fax: +55-11-3091-7322, e-mail: *eferro@usp.br*

#These two authors contributed equally

Keywords: THOP1; neurodegeneration; inflammation; sepsis; MHC-I; peptidome.

Supplementary information for Microarray data

Experimental design

A differential expression analysis was conducted on 30 samples from 3 regions of mouse CNS. Samples were either wild-type (WT) or thimet oligopeptidase 1 (EC 3.4.25.15; THOP1) knockout for the gene ($THOP1^{-/-}$). Only thymine DNA glycosylase (TDG) gene was found differentially expressed between WT and $THOP1^{-/-}$ mice for all regions with a FDR of approximately 0.001. A second round of analysis leaving out outlier samples produced similar results from pre-frontal cortex (PFC) and striatum (ST) samples. However, 395 differentially expressed genes were identified from hippocampus (HC) samples.

Wild-type (WT, n =5) and $THOP1^{-/-}$ (KO, n =5) individuals had RNA extracted for 3 CNS regions: prefrontal cortex (PFC), striatum (EST) and hippocampus (HC). The microarray experiments were performed with 30 individual mRNA samples submitted to Affymetrix (Thermo Fisher Scientific) GeneChip Mouse Gene 2.0 ST Array platform, generating 41.345 probe sets readings, of which 33.793 are the main ones and 7.552 are control probe sets. A second analysis was also executed for the set of samples without outliers, as described further ahead in the samples correlations analysis. Analysis were run with R (v3.4.0) statistical enviroment packages including BioConductor project packages. The Affymetrix microarray analysis routines, quality control, background removal and normalization were done using the protocols of the Oligo (v1.38.0) package. Differential expression testing, as well as rendering related figures, were conducted with the help of the Limma (v3.30.13) package. Hierarchical clustering and heatmap redering was executed using the PoiClaClu (v1.0.2) and Pheatmap (v1.0.8) packages. Part of the color scales used in illustrations was done with the help of the RColorBrewer (v1.1-2) package. Differentially expressed genes were submitted to the Panther DB tools (pantherdb.org) v12 for classification and overrepresentation tests (release 20170413) of Gene Ontology classes (GO), Panther protein classes, metabolic pathways and reactome pathways (v58). StringDB (v10.5) evidence cutoff was 0.9 (highest confidence) and MCL clustering inflation parameter was set at 6.

Quality control

The intensity amplitude from these samples showed good consistency among all replicates and experiments.

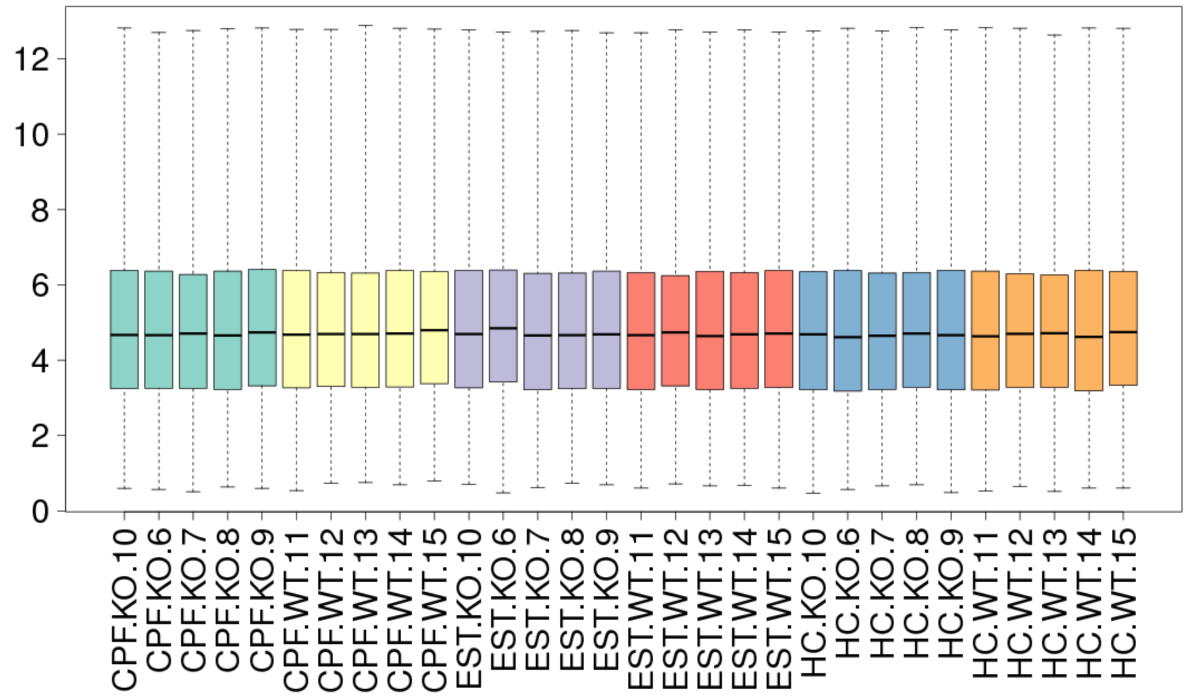

Figure 1 - Probe intensity readings distributions among samples. Intensities represented in logarithmic scale after background removal and normalization.

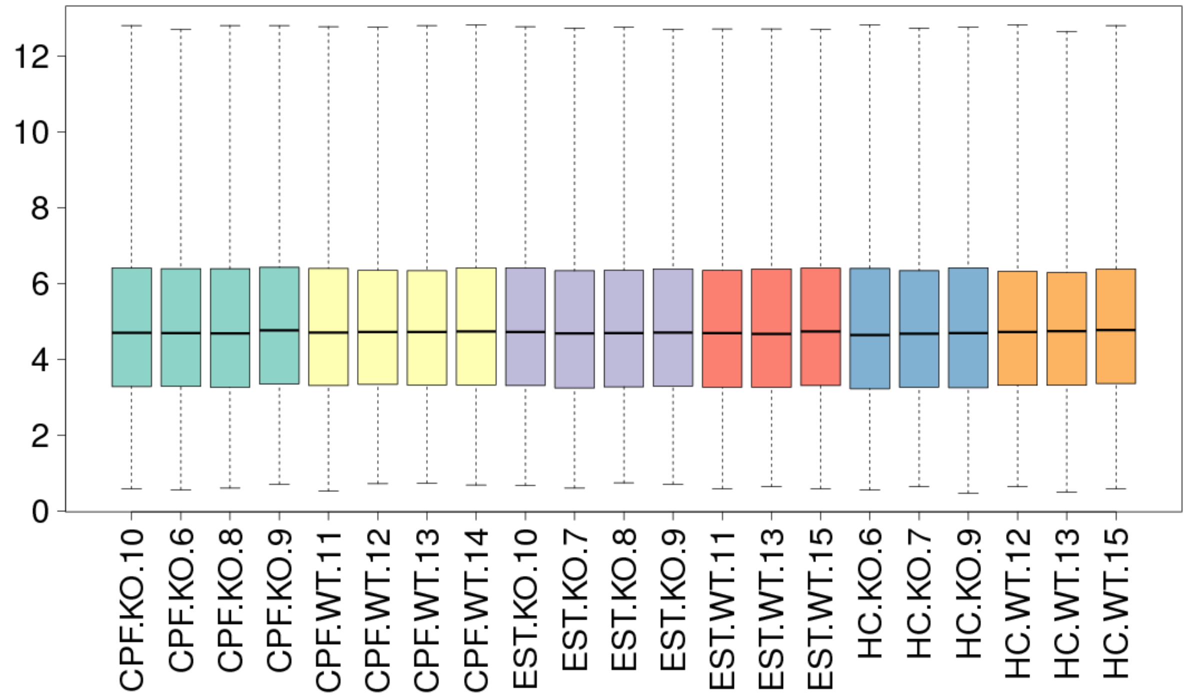

Figure 2 - Probe intensity readings distributions among samples <u>without outliers</u>. Intensities represented in logarithmic scale after background removal and normalization.

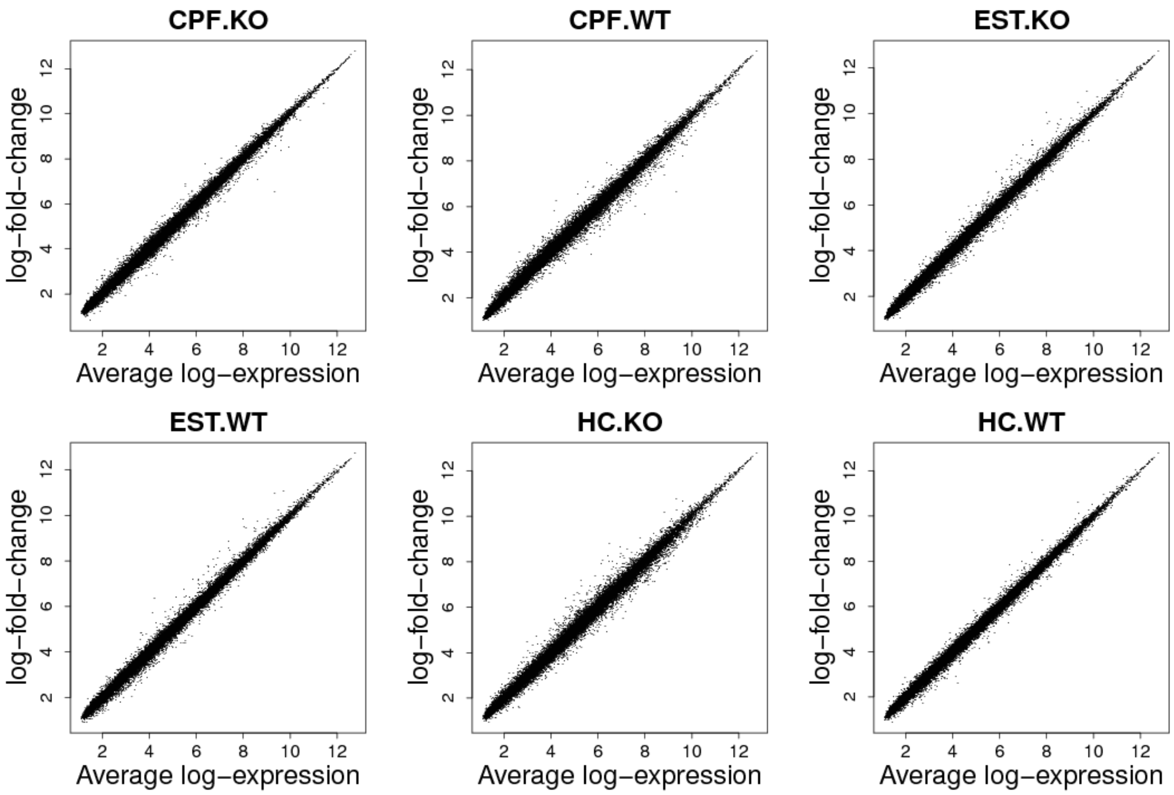

Figure 3 - Intensity readings variation for each phenotype as a correlation of expression values. Values calculated by the average of replicates of each phenotype, excluding the replicated being analysed. Log-fold-change as it relates to the average.

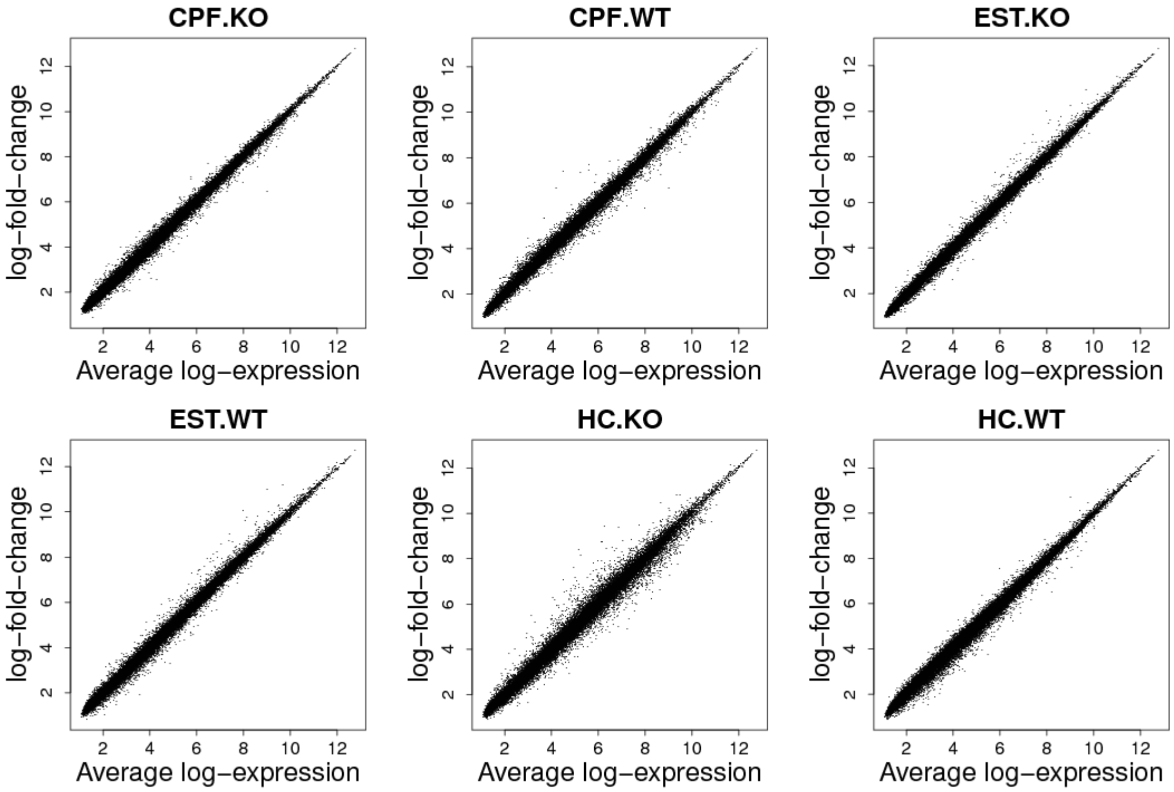

Figure 4 - Intensity readings variation for each phenotype as a correlation of expression values without outlier samples. Values calculated with the average of replicates of each phenotype, excluding the replicated being analyse, with log-fold-change as it relates to this average.

Samples correlation

Using the normalized expression data, in the form of the adjusted linear model coefficients, a sample distance matrix was constructed and a *Principal Components Analysis* (PCA) was executed. The matrix was used to render a heatmap with clustering. This analysis allowed us to asses that some of the samples, especially from the HC, did not conform to the behavior of the majority of replicates in some of the treatments (note that in Figure 3a the expression behavior of the replicates of treatments is not uniform, especially for HC, and so we decided to remove the outliers). For these samples, a second concomitant analysis was performed, excluding samples placed far from the immediate radius of the majority of replicates of each phenotype. That yielded a reduction of at least 1 replicate per phenotype, 2 in the case of the HC.

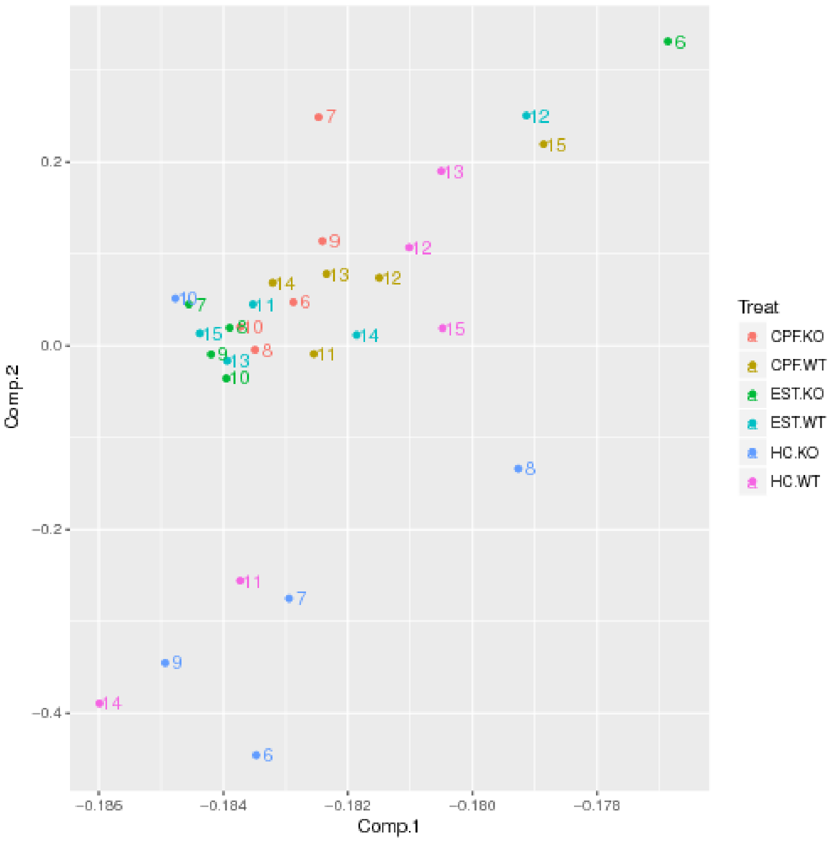

Figure 5 - Principal components analysis of samples. Each phenotype and CNS region represented in color and the subject number noted next to the point it is represented by. Only the 2 components with the greatest accumulated variance are shown.

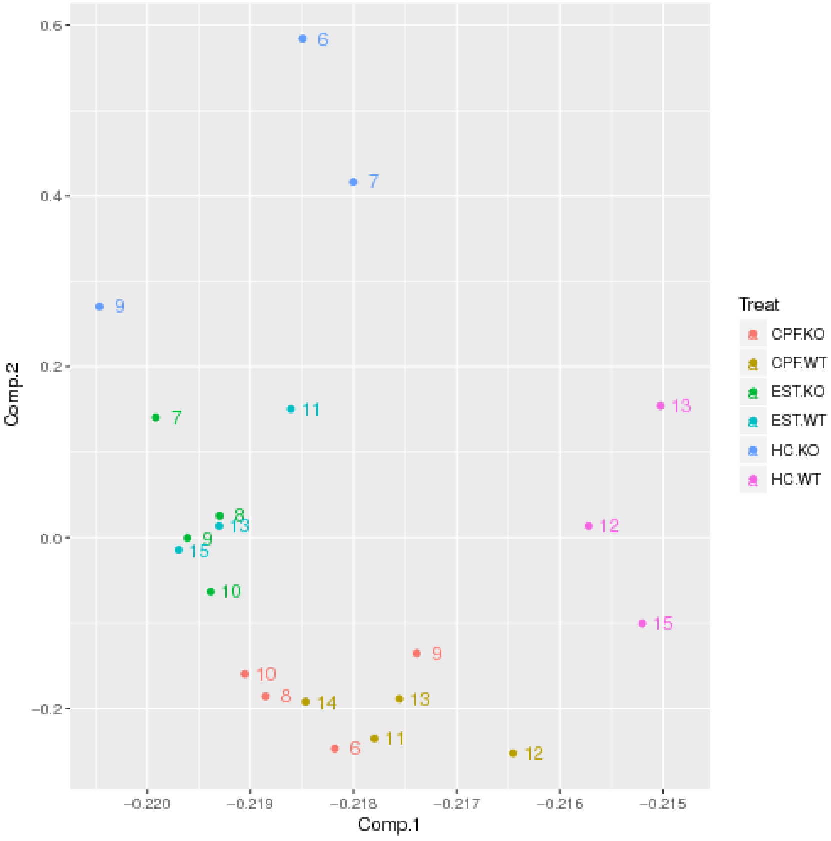

Figure 6 - Principal components analysis of samples <u>without outliers</u>. Each phenotype and CNS region represented in color and the subject number noted next to the point it is represented by. Only the 2 components with the greatest accumulated variance are shown.

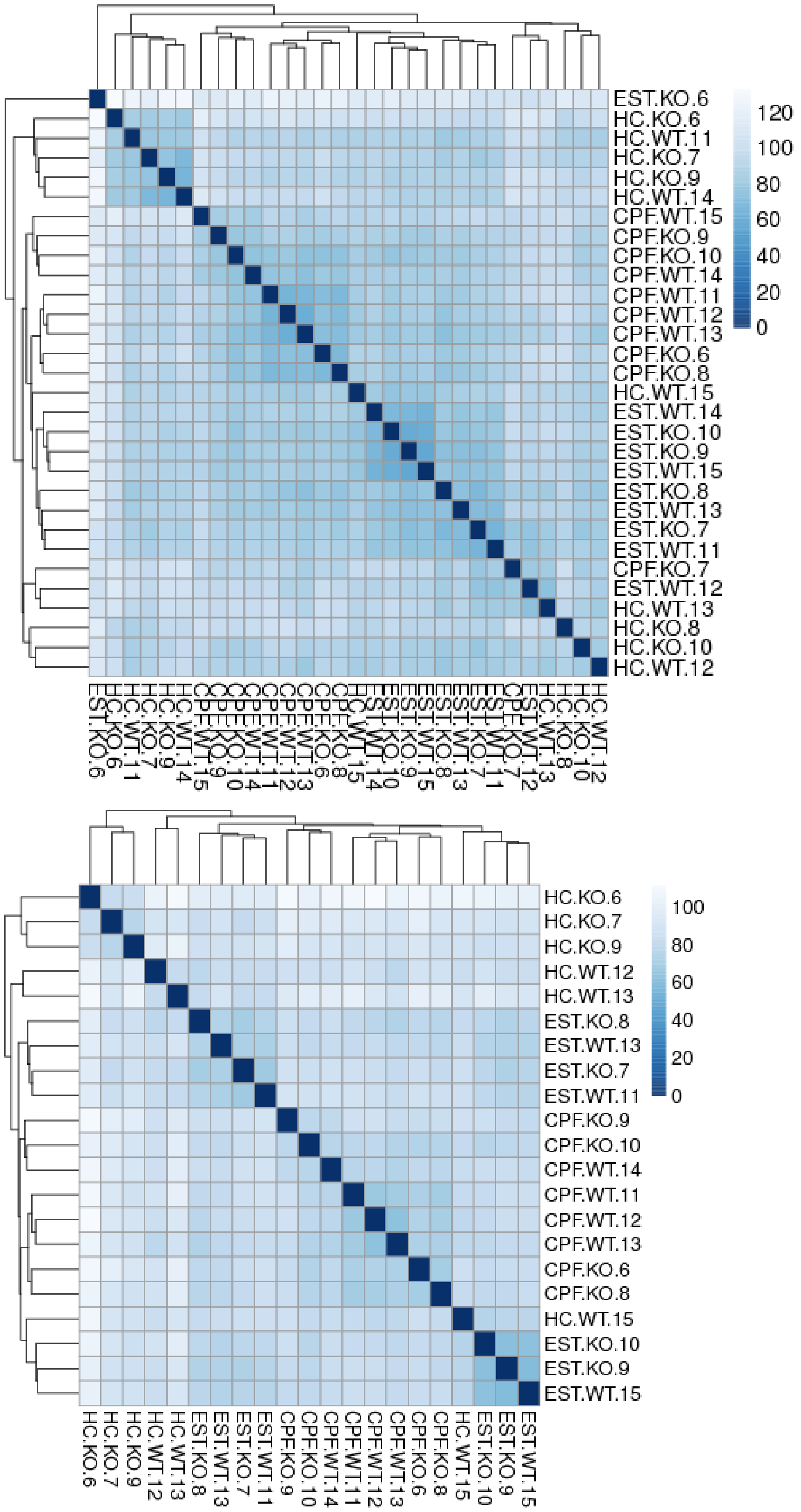

Figure 7 - Sample distance based heatmap and clustering. The distance reflects the expression measurements for each sample. Lower distances, of more related samples, are in darker blue and higher, less related samples, in lighter shades. The accompanying dendrogram is a representation of the hierarchical clustering on distances procedure. The bottom heatmap and clustering figure is from <u>samples without outliers.</u>

Differential expression (DE) testing

For these procedures three phenotype comparisons were used, only those between the same CNS region and only WT versus THOP1$^{-/-}$ (KO): CPF.WT-CPF.KO, EST.WT-EST.KO, HC.WT-HC.KO.

Using a false discovery rate (adjusted P-value; Q-value) cut-off of 0.05 only the TDP gene was found to be differentially expressed (DE) in any sample set scopes of CPF and EST. The only other DEs found were 6 reporter control probe sets of the microarray and a mitochondrially encoded tRNA cysteine in the CPF comparison. That was not the case of HC samples. With the full sample set HC.WT-HC.KO yielded the same result, only TDP was DE. However, excluding outlier samples it yielded 395 DEs, including TDP and having the same 6 reporters controls DE.

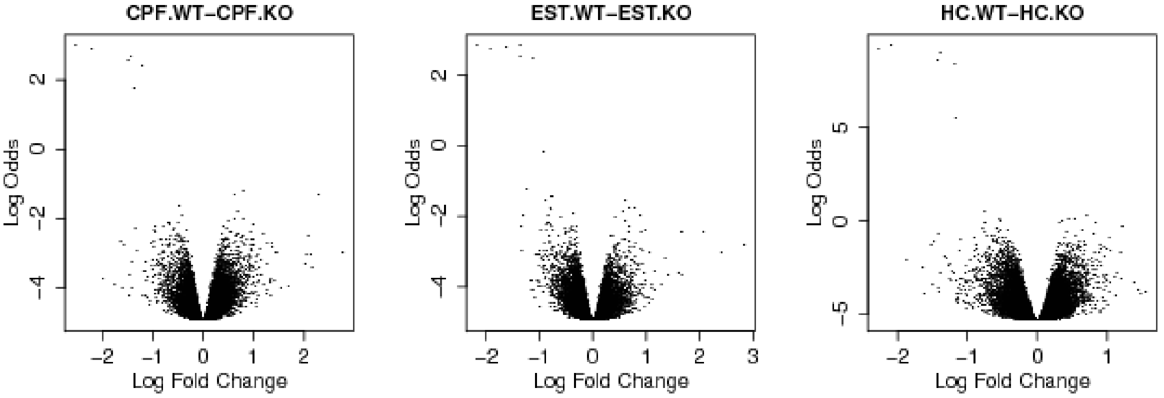

Figure 8 - Correlation of DE probability by expression change rate in comparisons. Higher Log Odds represent higher DE probability.

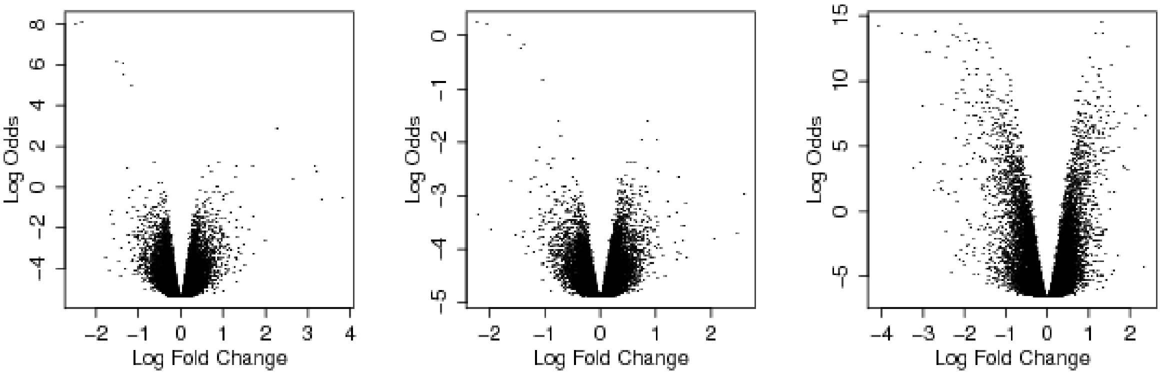

Figure 9 - Correlation of DE probability by expression change rate in comparisons without outlier samples. Higher Log Odds represent higher DE probability.

From the HC DEs, the top 5 genes were, in order of decreasing Q-value, an uncharacterized LOC101056001 locus, Pcbd1 - pterin 4 alpha carbinolamine dehydratase/dimerization cofactor of hepatocyte nuclear factor 1 alpha (TCF1) 1, Glra1 - glycine receptor - alpha 1 subunit, RAB37 - member RAS oncogene family and Dlgap2 - discs large (Drosophila) homolog-associated protein 2.

DE classification

Among the gene ontology categories of the HC DEs without outlier samples, as found through the Panther GO tool, those found with highest frequency were: cellular process in the Biological Process set, binding in the molecular function set and cell part in the Cellular Component set.

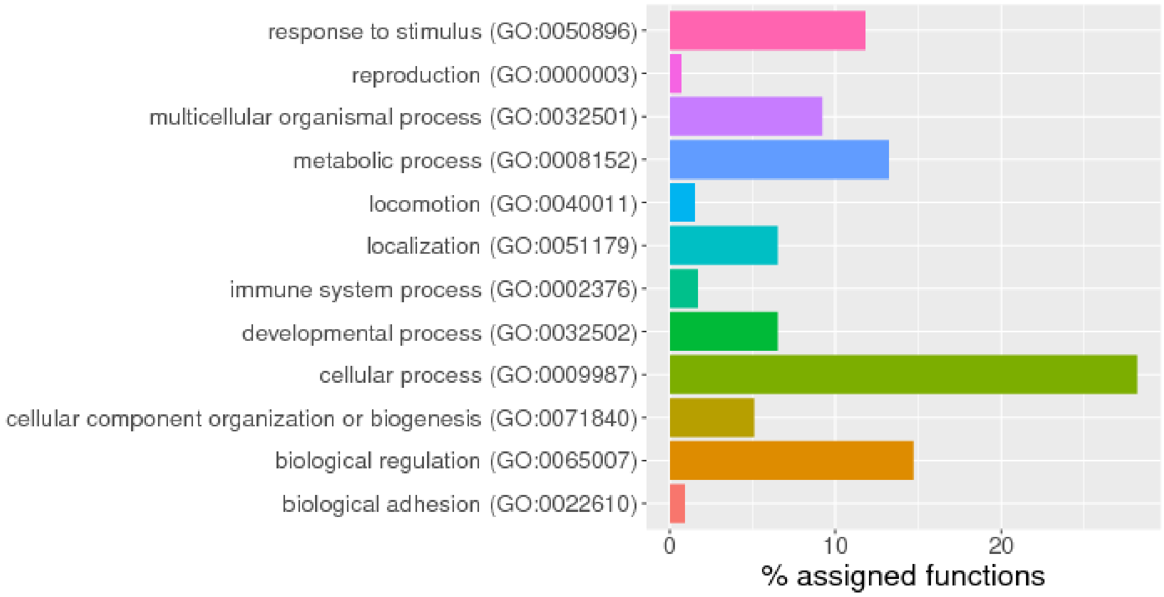

Figure10 - Biological Process categories found among the DE genes through PantherDB GO.

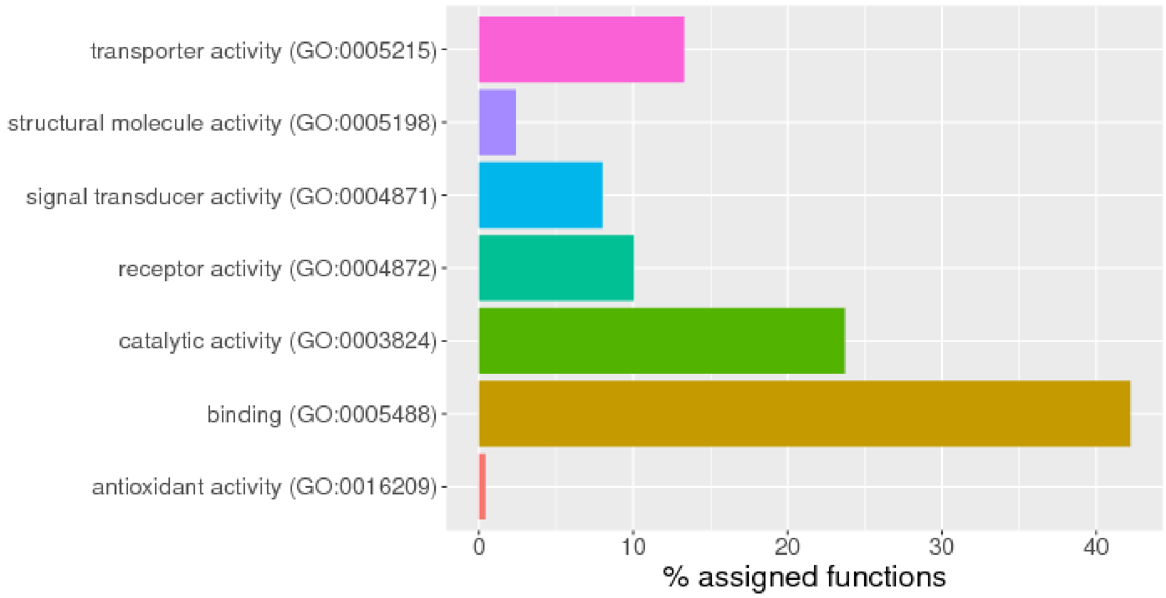

Figure 11 - Molecular function categories found among the DE genes through PantherDB GO.

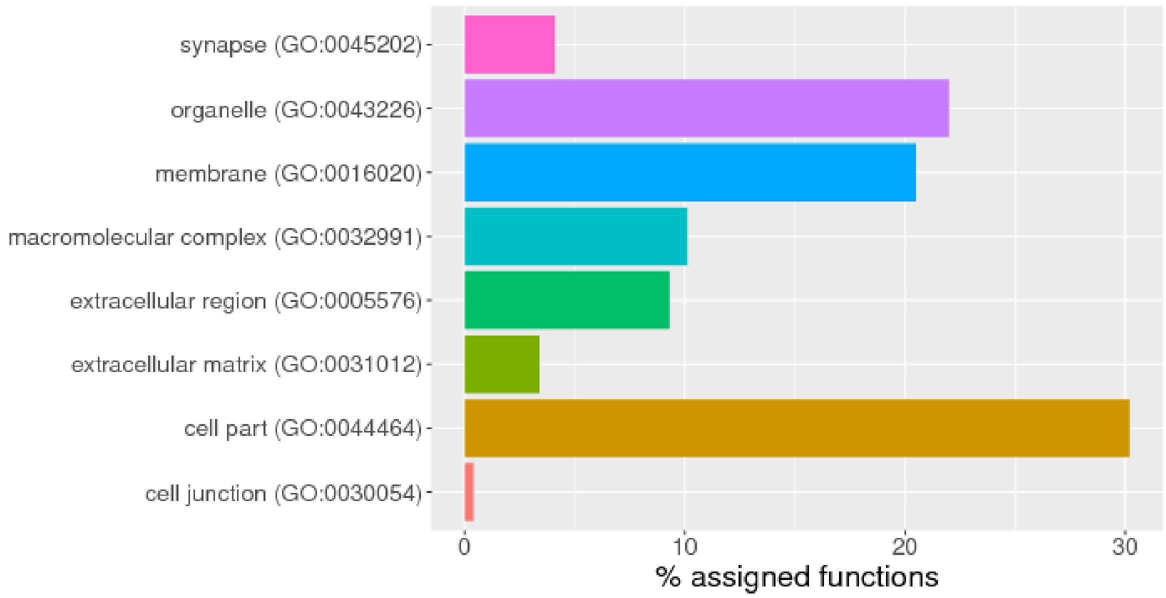

Figure 12 - Cellular Component categories found among the DE genes through PantherDB GO.

By the classification of proteins, the categories of chaperone, hydrolase, membrane trafficking protein and, mainly, oxy-uterase were found.

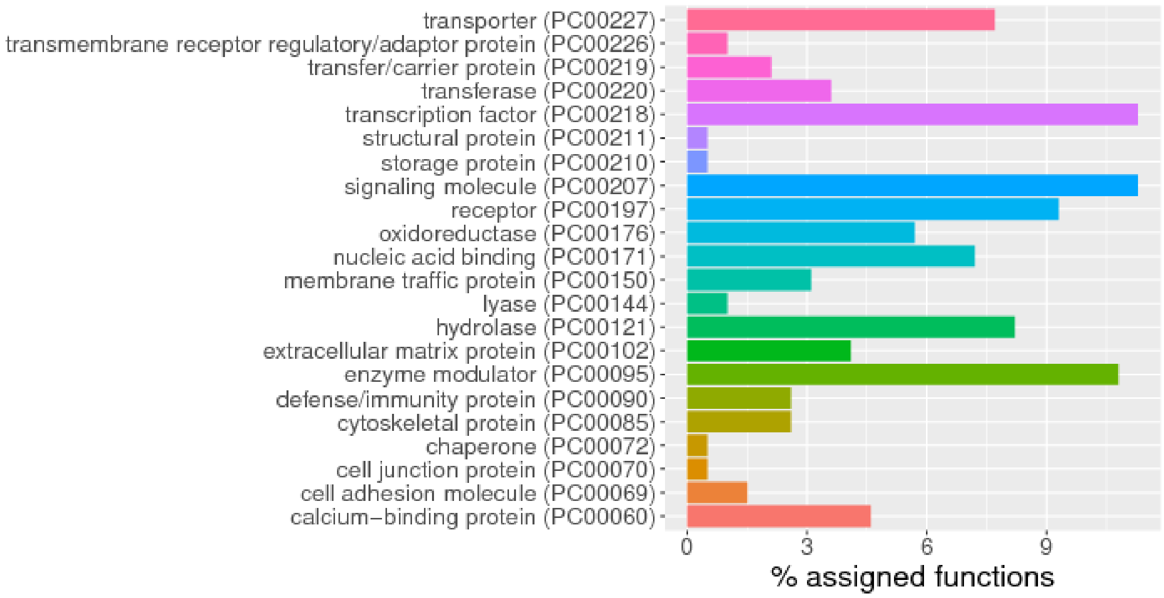

Figure 13 - Panther protein categories found among the DE genes through PantherDB.

Table 1 - Pathways categories found among the DE genes through PantherDB

Axon guidance mediated by netrin (P00009)
Beta3 adrenergic receptor signaling pathway (P04379)
Axon guidance mediated by Slit/Robo (P00008)
Metabotropic glutamate receptor group III pathway (P00039)
Beta2 adrenergic receptor signaling pathway (P04378)
Beta1 adrenergic receptor signaling pathway (P04377)
Apoptosis signaling pathway (P00006)
5HT4 type receptor mediated signaling pathway (P04376)
Angiogenesis (P00005)
Ionotropic glutamate receptor pathway (P00037)
Purine metabolism (P02769)
5HT3 type receptor mediated signaling pathway (P04375)
Alzheimer disease-presenilin pathway (P00004)
5HT2 type receptor mediated signaling pathway (P04374)
Alzheimer disease-amyloid secretase pathway (P00003)
5HT1 type receptor mediated signaling pathway (P04373)
Alpha adrenergic receptor signaling pathway (P00002)
5-Hydroxytryptamine degredation (P04372)
Adrenaline and noradrenaline biosynthesis (P00001)
5-Hydroxytryptamine biosynthesis (P04371)
Inflammation mediated by chemokine and cytokine signaling pathway (P00031)
Nicotine pharmacodynamics pathway (P06587)
Synaptic vesicle trafficking (P05734)
Huntington disease (P00029)
Heterotrimeric G-protein signaling pathway-Gq alpha and Go alpha mediated pathway (P00027)
Heterotrimeric G-protein signaling pathway-Gi alpha and Gs alpha mediated pathway (P00026)
Wnt signaling pathway (P00057)
Vitamin D metabolism and pathway (P04396)
VEGF signaling pathway (P00056)
Xanthine and guanine salvage pathway (P02788)
Thyrotropin-releasing hormone receptor signaling pathway (P04394)
Toll receptor signaling pathway (P00054)
T cell activation (P00053)
FGF signaling pathway (P00021)
TGF-beta signaling pathway (P00052)
Oxytocin receptor mediated signaling pathway (P04391)
Endothelin signaling pathway (P00019)
EGF receptor signaling pathway (P00018)
p38 MAPK pathway (P05918)
Parkinson disease (P00049)
Opioid proopiomelanocortin pathway (P05917)
PDGF signaling pathway (P00047)
Opioid prodynorphin pathway (P05916)
Oxidative stress response (P00046)
Opioid proenkephalin pathway (P05915)
Histamine H1 receptor mediated signaling pathway (P04385)
Notch signaling pathway (P00045)
Nicotinic acetylcholine receptor signaling pathway (P00044)
Cadherin signaling pathway (P00012)
Muscarinic acetylcholine receptor 2 and 4 signaling pathway (P00043)
Blood coagulation (P00011)
Dopamine receptor mediated signaling pathway (P05912)
Muscarinic acetylcholine receptor 1 and 3 signaling pathway (P00042)
B cell activation (P00010)
Angiotensin II-stimulated signaling through G proteins and beta-arrestin (P05911)
Metabotropic glutamate receptor group I pathway (P00041)
Cortocotropin releasing factor receptor signaling pathway (P04380)
Metabotropic glutamate receptor group II pathway (P00040)
CCKR signaling map (P06959)

Gonadotropin-releasing hormone receptor pathway (P06664)

An overrepresentation test was also executed with the PantherDB tools, revealing categories that have been enriched or diminished in relation to what is expected of a random sampling of the genome at a FDR lower than 0.05. The highest fold enrichment or reduction found per category set were, in order: neuromuscular synaptic transmission and sensory perception of smell in the Biological Process set, proteinaceous extracellular matrix and no reductions in the Cellular Component set, neuropeptide hormone activity and no reduction in the Molecular Function set, GABA receptor and no reduction in the PANTHER Protein Classes, 5HT3 type receptor mediated signaling pathway and no reduction in the PANTHER Pathways and Na+/Cl- dependent neurotransmitter transporters and no reduction in the Reactome pathways.

Table 2 - PantherDB set categories relevantly enriched or diminished among the DE genes

	HC.WT-HC.KO.DE	fold Enrichment	FDR
PANTHER GO-Slim Biological Process			
sensory perception of smell (GO:0007608)	0	< 0.01	2.08E-03
sensory perception of chemical stimulus (GO:0007606)	0	< 0.01	1.09E-03
sensory perception (GO:0007600)	3	.24	2.24E-02
cellular process (GO:0009987)	165	1.32	3.22E-04
regulation of biological process (GO:0050789)	70	1.48	1.08E-02
biological regulation (GO:0065007)	86	1.54	8.98E-04
multicellular organismal process (GO:0032501)	54	1.66	4.19E-03
signal transduction (GO:0007165)	69	1.67	8.47E-04
single-multicellular organism process (GO:0044707)	54	1.67	4.28E-03
developmental process (GO:0032502)	38	1.76	1.29E-02
system process (GO:0003008)	38	1.79	8.65E-03
neurological system process (GO:0050877)	37	1.86	6.37E-03
cell communication (GO:0007154)	88	1.88	3.09E-07
anion transport (GO:0006820)	10	2.85	3.34E-02
mesoderm development (GO:0007498)	11	2.91	2.16E-02
ectoderm development (GO:0007398)	9	3.03	3.29E-02
response to endogenous stimulus (GO:0009719)	10	3.06	2.21E-02
ion transport (GO:0006811)	20	3.58	8.60E-05
cell-cell signaling (GO:0007267)	31	3.69	1.41E-07
cell growth (GO:0016049)	6	4.29	3.22E-02
amino acid transport (GO:0006865)	6	5.00	2.00E-02
synaptic transmission (GO:0007268)	29	5.18	5.39E-10
neurotransmitter secretion (GO:0007269)	7	5.21	8.20E-03
behavior (GO:0007610)	5	5.74	2.32E-02
neuron-neuron synaptic transmission (GO:0007270)	10	7.87	8.37E-05
synaptic vesicle exocytosis (GO:0016079)	7	8.45	9.64E-04
neuromuscular synaptic transmission (GO:0007274)	6	8.94	2.17E-03
PANTHER GO-Slim Cellular Component			
proteinaceous extracellular matrix (GO:0005578)	7	7.78	7.38E-04
axon (GO:0030424)	4	6.36	2.68E-02
synapse (GO:0045202)	11	5.92	1.68E-04
neuron projection (GO:0043005)	18	5.02	3.69E-06
extracellular matrix (GO:0031012)	9	4.47	2.60E-03
dendrite (GO:0030425)	6	3.85	3.10E-02
cytoplasmic membrane-bounded vesicle (GO:0016023)	10	3.63	4.54E-03
cell projection (GO:0042995)	19	3.44	1.20E-04
integral to membrane (GO:0016021)	32	2.15	8.59E-04
extracellular region (GO:0005576)	25	1.98	8.64E-03
plasma membrane (GO:0005886)	47	1.90	5.27E-04
extracellular space (GO:0005615)	20	1.88	3.59E-02
membrane (GO:0016020)	55	1.62	3.93E-03
PANTHER GO-Slim Molecular Function			
neuropeptide hormone activity (GO:0005184)	4	23.34	1.31E-03
neurotransmitter transporter activity (GO:0005326)	6	16.80	1.95E-04
voltage-gated potassium channel activity (GO:0005249)	9	10.00	4.87E-05
voltage-gated ion channel activity (GO:0005244)	9	7.08	2.80E-04
hormone activity (GO:0005179)	5	5.65	3.75E-02

ligand-gated ion channel activity (GO:0015276)	10	5.34	7.76E-04
amino acid transmembrane transporter activity (GO:0015171)	5	5.15	4.72E-02
ion channel activity (GO:0005216)	19	4.03	5.85E-05
lipase activity (GO:0016298)	7	3.92	3.71E-02
sequence-specific DNA binding RNA polymerase II transcription factor activity (GO:0000981)	10	3.06	3.48E-02
transmembrane transporter activity (GO:0022857)	32	2.71	1.16E-04
receptor binding (GO:0005102)	33	2.44	1.59E-04
transporter activity (GO:0005215)	33	2.41	1.62E-04
protein binding (GO:0005515)	64	1.60	3.26E-03
binding (GO:0005488)	105	1.40	3.33E-03
PANTHER Protein Class			
acetylcholine receptor (PC00037)	6	10.00	1.19E-02
GABA receptor (PC00023)	6	10.00	5.97E-03
signaling molecule (PC00207)	22	2.35	2.30E-02
PANTHER Pathways			
5HT3 type receptor mediated signaling pathway (P04375)	3	14.00	2.25E-02
Adrenaline and noradrenaline biosynthesis (P00001)	6	13.55	6.51E-04
Alpha adrenergic receptor signaling pathway (P00002)	4	12.18	9.50E-03
Muscarinic acetylcholine receptor 1 and 3 signaling pathway (P00042)	10	11.67	8.16E-06
Nicotine pharmacodynamics pathway (P06587)	5	10.00	5.49E-03
Synaptic vesicle trafficking (P05734)	4	10.00	1.38E-02
5HT2 type receptor mediated signaling pathway (P04374)	9	9.55	8.82E-05
Thyrotropin-releasing hormone receptor signaling pathway (P04394)	7	8.17	1.77E-03
Histamine H1 receptor mediated signaling pathway (P04385)	5	8.14	9.12E-03
Oxytocin receptor mediated signaling pathway (P04391)	6	7.37	5.25E-03
Dopamine receptor mediated signaling pathway (P05912)	5	6.36	1.95E-02
Muscarinic acetylcholine receptor 2 and 4 signaling pathway (P00043)	5	5.74	2.42E-02
Nicotinic acetylcholine receptor signaling pathway (P00044)	7	5.10	9.52E-03
CCKR signaling map (P06959)	10	4.38	4.22E-03
Heterotrimeric G-protein signaling pathway-Gq alpha and Go alpha mediated pathway (P00027)	7	4.08	2.37E-02
Reactome pathways			
Na+/Cl- dependent neurotransmitter transporters (R-MMU-442660)	5	18.42	4.62E-03
Synthesis of IP3 and IP4 in the cytosol (R-MMU-1855204)	5	14.00	1.08E-02
Voltage gated Potassium channels (R-MMU-1296072)	7	11.67	2.68E-03
Inositol phosphate metabolism (R-MMU-1483249)	6	9.55	1.18E-02
Neurotransmitter Release Cycle (R-MMU-112310)	6	8.94	1.35E-02
Potassium Channels (R-MMU-1296071)	9	6.50	4.17E-03
G alpha (q) signalling events (R-MMU-416476)	15	6.33	3.49E-05
Sphingolipid metabolism (R-MMU-428157)	7	6.13	2.67E-02
Neuronal System (R-MMU-112316)	23	5.24	5.46E-07
Transmission across Chemical Synapses (R-MMU-112315)	13	4.69	3.09E-03
Peptide ligand-binding receptors (R-MMU-375276)	10	3.91	3.94E-02
Class A/1 (Rhodopsin-like receptors) (R-MMU-373076)	14	3.44	1.45E-02
Gastrin-CREB signalling pathway via PKC and MAPK (R-MMU-881907)	18	3.33	4.45E-03
Platelet activation, signaling and aggregation (R-MMU-76002)	12	3.29	4.37E-02
GPCR ligand binding (R-MMU-500792)	16	2.94	2.24E-02

StringDB is an interactome network constructing tool that compiles evidence from several databases and the literature. Among the StringDB THOP1 interacting genes none has been found DE. As for the chromosome 10 genomic neighbors of THOP1 , a receptor accessory protein 6 (REEP6) and a EEF2 pseudogene, none have been found DE as well. That is, the THOP1 *knockout* assay does not seem to have directly influenced close by genes. A StringDB network was also constructed for the DE, which yielded 54 genes been included in 19 clusters, of which only 31 in 5 clusters have 3 or more genes. That is, 31 genes in 5 clusters seem to form interactome modules altered by the mutation of THOP1 in the HC. An enrichment analysis of the StringDB included genes yielded the following top relevant

category sets: regulation of multicellular organismal process in the Biological Process set, synapse part in Celullar Component set, ligand-gated ion channel activity in the Molecular Function set, Potassium channel voltage-dependent EAG/ELK/ERG in the InterPro classification and Calcium signaling pathway in KEGG pathways set.

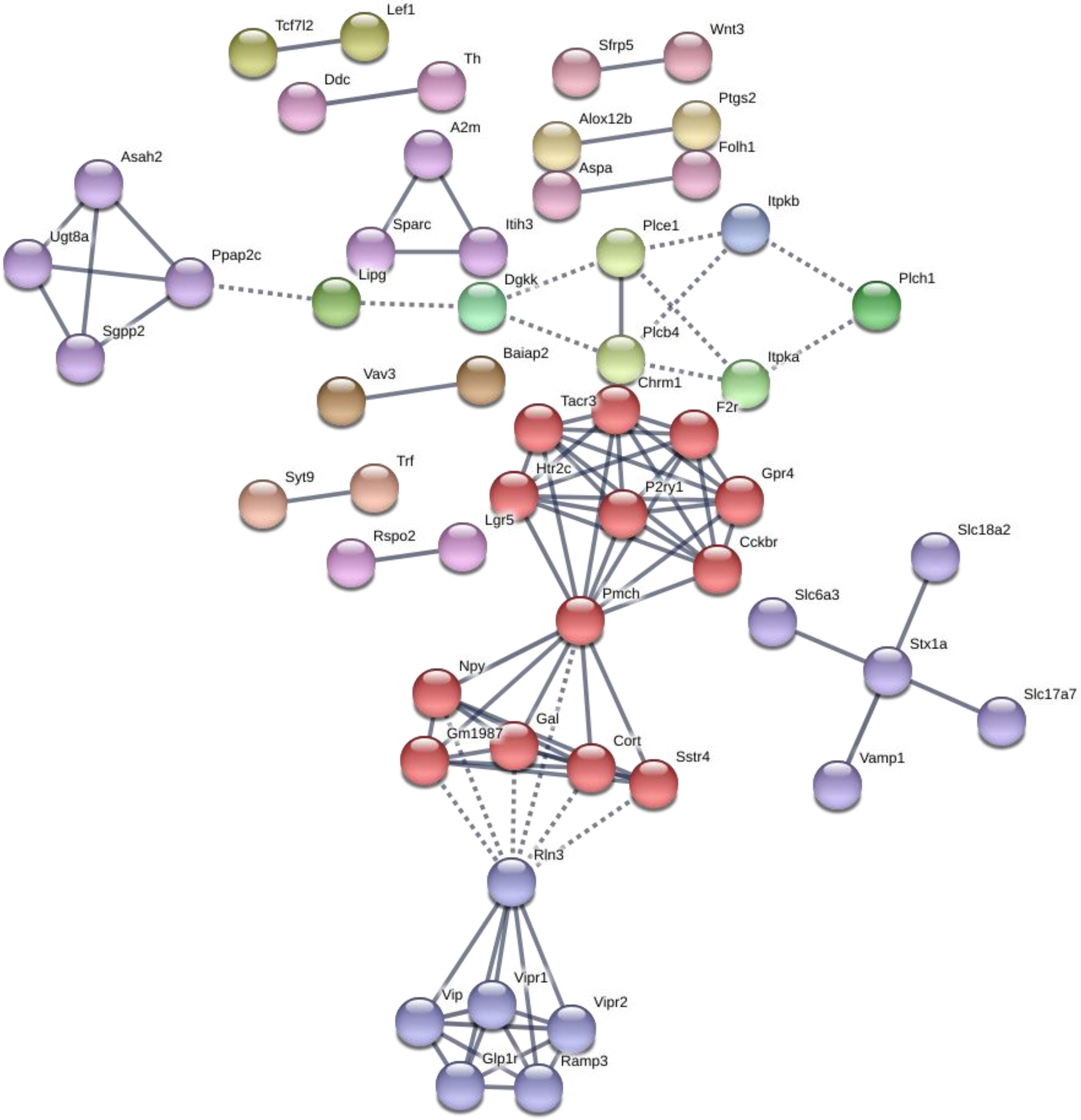

Figure 14 - StringDB DE interacting genes network. Stronger bonds between nodes correspond to stronger evidence of interaction. Same color nodes belong to more likely interacting genes, as per the MCL clustering method.

Table 3 - Gene categories enriched in the StringDB network interacting DE genes

#ID	pathway description	observed gene count	false discovery rate
Celular Component			
GO.0044456	synapse part	33	7.38e-11
GO.0043005	neuron projection	39	2.48e-09
GO.0045202	synapse	34	2.48e-09
GO.0044459	plasma membrane part	61	4.89e-09
GO.0097458	neuron part	44	4.89e-09
GO.0097060	synaptic membrane	20	2.97e-08
GO.0045211	postsynaptic membrane	18	5.42e-08
GO.0036477	somatodendritic compartment	28	7.75e-07
GO.0071944	cell periphery	90	9.27e-07
GO.0098794	postsynapse	21	9.27e-07
GO.0098590	plasma membrane region	33	1.7e-06
GO.0005886	plasma membrane	87	2.75e-06
GO.0031226	intrinsic component of plasma membrane	35	4.75e-06
GO.0098589	membrane region	36	4.75e-06
GO.0031224	intrinsic component of membrane	102	6.69e-06
GO.0043025	neuronal cell body	20	9.26e-06
GO.0044425	membrane part	114	9.62e-06
GO.0005887	integral component of plasma membrane	33	1.08e-05
GO.0042995	cell projection	47	1.66e-05
GO.0016021	integral component of membrane	98	1.7e-05
GO.0044297	cell body	21	1.7e-05
GO.0030425	dendrite	19	0.000164
GO.0030054	cell junction	34	0.000167
GO.0030424	axon	17	0.00035
GO.0098805	whole membrane	45	0.000369
GO.0030672	synaptic vesicle membrane	7	0.000487
GO.0014069	postsynaptic density	11	0.0023
GO.1902495	transmembrane transporter complex	13	0.00242
GO.0008021	synaptic vesicle	9	0.00256
GO.0034702	ion channel complex	12	0.00347
GO.0016020	membrane	129	0.00467
GO.0032589	neuron projection membrane	5	0.00467
GO.0098793	presynapse	9	0.00546
GO.0032590	dendrite membrane	4	0.00963
GO.0044463	cell projection part	24	0.00963
GO.0005575	cellular_component	214	0.0108
GO.0009986	cell surface	21	0.0275
GO.0044433	cytoplasmic vesicle part	14	0.0275
Molecular Function			
GO.0015276	ligand-gated ion channel activity	11	0.00167
GO.0022836	gated channel activity	16	0.00167
GO.0004871	signal transducer activity	35	0.00196
GO.0005230	extracellular ligand-gated ion channel activity	8	0.00196
GO.0043565	sequence-specific DNA binding	29	0.00206
GO.0046873	metal ion transmembrane transporter activity	17	0.00206
GO.0003674	molecular_function	179	0.00247
GO.0015075	ion transmembrane transporter activity	24	0.00247
GO.0015267	channel activity	17	0.00247
GO.0022891	substrate-specific transmembrane transporter activity	25	0.00247
GO.0022892	substrate-specific transporter activity	28	0.00247
GO.0038023	signaling receptor activity	29	0.00247
GO.0005216	ion channel activity	16	0.00292
GO.0000976	transcription regulatory region sequence-specific DNA binding	21	0.00373
GO.0022857	transmembrane transporter activity	26	0.00373
GO.0005215	transporter activity	30	0.00414
GO.0060089	molecular transducer activity	35	0.00424
GO.0005488	binding	154	0.00431
GO.0000977	RNA polymerase II regulatory region sequence-specific DNA binding	19	0.00578
GO.0001228	transcriptional activator activity, RNA polymerase II transcription regulatory region sequence-specific binding	15	0.00578
GO.0005231	excitatory extracellular ligand-gated ion channel activity	6	0.00578
GO.0000982	transcription factor activity, RNA polymerase II core promoter proximal region sequence-specific binding	15	0.00599
GO.0005515	protein binding	90	0.00599
GO.0044212	transcription regulatory region DNA binding	23	0.00599
GO.0004872	receptor activity	29	0.00935
GO.0001077	transcriptional activator activity, RNA polymerase II core promoter proximal region sequence-specific binding	12	0.00977
GO.0005249	voltage-gated potassium channel activity	7	0.00977
GO.0008324	cation transmembrane transporter activity	18	0.0189
GO.0001046	core promoter sequence-specific DNA binding	7	0.0219
GO.0022890	inorganic cation transmembrane transporter activity	16	0.0224
GO.0015370	solute:sodium symporter activity	5	0.0236
GO.0000978	RNA polymerase II core promoter proximal region sequence-specific DNA binding	13	0.0261
GO.0022843	voltage-gated cation channel activity	8	0.0356
GO.0005179	hormone activity	5	0.0395
GO.0015077	monovalent inorganic cation	12	0.0408

	transmembrane transporter activity		
GO.0005261	cation channel activity	11	0.0452
GO.0004699	calcium-independent protein kinase C activity	2	0.0483
GO.0004999	vasoactive intestinal polypeptide receptor activity	2	0.0483
GO.0015081	sodium ion transmembrane transporter activity	7	0.0483
InterPro			
IPR003938	Potassium channel, voltage-dependent, EAG/ELK/ERG	4	0.0146
KEGG			
4020	Calcium signaling pathway	14	5.18e-05
4080	Neuroactive ligand-receptor interaction	17	5.79e-05
5033	Nicotine addiction	6	0.00162
5031	Amphetamine addiction	7	0.00163
4725	Cholinergic synapse	8	0.00831
4723	Retrograde endocannabinoid signaling	7	0.0219
5030	Cocaine addiction	5	0.0219
5034	Alcoholism	8	0.0265
562	Inositol phosphate metabolism	5	0.0437
4721	Synaptic vesicle cycle	5	0.0437
Biological Process			
GO.0051239	regulation of multicellular organismal process	74	5.91e-10
GO.0007610	behavior	32	3.61e-09
GO.0065008	regulation of biological quality	76	3.61e-09
GO.0007399	nervous system development	61	1.06e-08
GO.0022008	neurogenesis	49	3.46e-08
GO.0044708	single-organism behavior	26	6.09e-08
GO.0007154	cell communication	91	7.46e-08
GO.0007267	cell-cell signaling	29	7.46e-08
GO.0044700	single organism signaling	89	7.46e-08
GO.0048699	generation of neurons	46	7.46e-08
GO.0051240	positive regulation of multicellular organismal process	48	7.46e-08
GO.0032501	multicellular organismal process	115	7.91e-08
GO.0044707	single-multicellular organism process	112	1.09e-07
GO.0007268	synaptic transmission	21	1.06e-06
GO.0051094	positive regulation of developmental process	41	1.1e-06
GO.0007165	signal transduction	81	2.11e-06
GO.0044763	single-organism cellular process	165	2.11e-06
GO.0044767	single-organism developmental process	102	3.52e-06
GO.0048167	regulation of synaptic plasticity	13	3.92e-06
GO.0050896	response to stimulus	114	4.37e-06
GO.0050793	regulation of developmental process	58	5.23e-06
GO.0051960	regulation of nervous system development	30	5.23e-06
GO.2000026	regulation of multicellular organismal development	48	5.23e-06
GO.0032879	regulation of localization	58	5.33e-06
GO.0051716	cellular response to stimulus	98	5.91e-06
GO.0030154	cell differentiation	76	6.18e-06
GO.0050803	regulation of synapse structure or activity	16	8.34e-06
GO.0007186	G-protein coupled receptor signaling pathway	31	9.91e-06
GO.0003008	system process	40	9.97e-06
GO.0007275	multicellular organismal development	91	9.97e-06
GO.0050804	modulation of synaptic transmission	17	1.05e-05
GO.0048518	positive regulation of biological process	100	1.82e-05
GO.0045597	positive regulation of cell differentiation	31	2.28e-05
GO.0048731	system development	81	2.72e-05
GO.0030001	metal ion transport	24	3.11e-05
GO.0050767	regulation of neurogenesis	26	3.14e-05
GO.0030182	neuron differentiation	31	3.79e-05
GO.0050890	cognition	16	3.84e-05
GO.0051962	positive regulation of nervous system development	21	4.25e-05
GO.0007611	learning or memory	15	4.51e-05
GO.0048522	positive regulation of cellular process	89	4.56e-05
GO.0050794	regulation of cellular process	143	5.18e-05
GO.0043269	regulation of ion transport	24	5.45e-05
GO.0060284	regulation of cell development	29	6.49e-05
GO.0048468	cell development	44	7.07e-05
GO.0065007	biological regulation	153	7.33e-05
GO.0010975	regulation of neuron projection development	18	0.000112
GO.0044057	regulation of system process	19	0.000113
GO.0051241	negative regulation of multicellular organismal process	32	0.000113
GO.0051049	regulation of transport	44	0.000126
GO.0031344	regulation of cell projection organization	21	0.000128
GO.0007218	neuropeptide signaling pathway	10	0.000129
GO.0022603	regulation of anatomical structure morphogenesis	30	0.000132
GO.0048856	anatomical structure development	88	0.000134
GO.0044699	single-organism process	168	0.000136
GO.0009605	response to external stimulus	43	0.000183
GO.0050769	positive regulation of neurogenesis	18	0.000201
GO.0045664	regulation of neuron differentiation	21	0.000307
GO.0010720	positive regulation of cell development	20	0.000344
GO.0045595	regulation of cell differentiation	41	0.000344
GO.0010646	regulation of cell communication	59	0.000375
GO.0016477	cell migration	25	0.000419
GO.0050789	regulation of biological process	144	0.000561
GO.0007417	central nervous system development	28	0.000664
GO.0023051	regulation of signaling	56	0.000667
GO.0044765	single-organism transport	56	0.000667
GO.0048666	neuron development	24	0.000772
GO.0050877	neurological system process	28	0.000772
GO.0007274	neuromuscular synaptic transmission	5	0.000911
GO.0001503	ossification	14	0.000957
GO.0009725	response to hormone	25	0.00122
GO.0007626	locomotory behavior	13	0.00126
GO.0006811	ion transport	31	0.00135
GO.0007423	sensory organ development	21	0.00137
GO.0055082	cellular chemical homeostasis	19	0.00162
GO.0050773	regulation of dendrite development	9	0.00177
GO.0048513	organ development	61	0.00179
GO.0090596	sensory organ morphogenesis	14	0.00192
GO.0051179	localization	79	0.00198
GO.0006812	cation transport	24	0.00206
GO.0051674	localization of cell	25	0.00206
GO.0048598	embryonic morphogenesis	22	0.00216
GO.0048562	embryonic organ morphogenesis	15	0.00223
GO.0010769	regulation of cell morphogenesis involved in	14	0.00248

	differentiation		
GO.1902578	single-organism localization	57	0.00248
GO.0034220	ion transmembrane transport	24	0.00252
GO.0048545	response to steroid hormone	17	0.00258
GO.0050801	ion homeostasis	19	0.00268
GO.0055080	cation homeostasis	18	0.00279
GO.0072507	divalent inorganic cation homeostasis	14	0.00279
GO.0030003	cellular cation homeostasis	16	0.00291
GO.0048878	chemical homeostasis	25	0.00291
GO.0060998	regulation of dendritic spine development	6	0.0032
GO.0001964	startle response	5	0.0035
GO.0030900	forebrain development	16	0.0037
GO.0042592	homeostatic process	33	0.0037
GO.0009719	response to endogenous stimulus	32	0.00372
GO.0098771	inorganic ion homeostasis	18	0.00397
GO.0030534	adult behavior	10	0.00404
GO.0055074	calcium ion homeostasis	13	0.00409
GO.0098660	inorganic ion transmembrane transport	19	0.00409
GO.0009891	positive regulation of biosynthetic process	42	0.00436
GO.0072503	cellular divalent inorganic cation homeostasis	13	0.00456
GO.0060322	head development	23	0.00478
GO.0010035	response to inorganic substance	16	0.00508
GO.0045665	negative regulation of neuron differentiation	10	0.00508
GO.0007420	brain development	22	0.0055
GO.0055065	metal ion homeostasis	16	0.0055
GO.0009887	organ morphogenesis	27	0.00565
GO.0010038	response to metal ion	13	0.00574
GO.0031328	positive regulation of cellular biosynthetic process	41	0.00579
GO.0014070	response to organic cyclic compound	24	0.00605
GO.0021543	pallium development	10	0.00605
GO.0007612	learning	9	0.00658
GO.0051480	cytosolic calcium ion homeostasis	10	0.00734
GO.0048583	regulation of response to stimulus	59	0.00762
GO.0022604	regulation of cell morphogenesis	17	0.00832
GO.0010243	response to organonitrogen compound	21	0.00844
GO.0006875	cellular metal ion homeostasis	14	0.00854
GO.0042472	inner ear morphogenesis	8	0.00874
GO.0000160	phosphorelay signal transduction system	3	0.00915
GO.0006874	cellular calcium ion homeostasis	12	0.00915
GO.0007204	positive regulation of cytosolic calcium ion concentration	9	0.00915
GO.0008284	positive regulation of cell proliferation	23	0.00915
GO.0021902	commitment of neuronal cell to specific neuron type in forebrain	3	0.00915
GO.0045666	positive regulation of neuron differentiation	13	0.00915
GO.0051592	response to calcium ion	8	0.0102
GO.0055085	transmembrane transport	26	0.0109
GO.1903530	regulation of secretion by cell	19	0.0109
GO.0051046	regulation of secretion	20	0.0111
GO.0051050	positive regulation of transport	24	0.0113
GO.0007200	phospholipase C-activating G-protein coupled receptor signaling pathway	6	0.0118
GO.0021879	forebrain neuron differentiation	6	0.0118
GO.0040011	locomotion	27	0.0118
GO.0023056	positive regulation of signaling	33	0.0121
GO.0031644	regulation of neurological system process	6	0.0127
GO.0098655	cation transmembrane transport	18	0.0147
GO.1903522	regulation of blood circulation	10	0.0159
GO.0034765	regulation of ion transmembrane transport	14	0.0164
GO.0007631	feeding behavior	7	0.0177
GO.0009888	tissue development	38	0.0177
GO.0015842	synaptic vesicle amine transport	2	0.0177
GO.0033076	isoquinoline alkaloid metabolic process	2	0.0177
GO.0035759	mesangial cell-matrix adhesion	2	0.0177
GO.0051602	response to electrical stimulus	5	0.0177
GO.0052314	phytoalexin metabolic process	2	0.0177
GO.0010721	negative regulation of cell development	12	0.0177
GO.0006366	transcription from RNA polymerase II promoter	17	0.0182
GO.0042127	regulation of cell proliferation	33	0.0184
GO.0048839	inner ear development	10	0.0184
GO.0060384	innervation	4	0.0186
GO.0043627	response to estrogen	10	0.0190
GO.0006836	neurotransmitter transport	8	0.0192
GO.0033993	response to lipid	22	0.0195
GO.0051961	negative regulation of nervous system development	11	0.0195
GO.0042220	response to cocaine	5	0.0204
GO.0046903	secretion	16	0.0205
GO.0051173	positive regulation of nitrogen compound metabolic process	39	0.0205
GO.0060429	epithelium development	27	0.0213
GO.0032355	response to estradiol	8	0.0217
GO.0042471	ear morphogenesis	8	0.0217
GO.0042493	response to drug	15	0.0224
GO.0010647	positive regulation of cell communication	34	0.0225
GO.0051047	positive regulation of secretion	13	0.0229
GO.0048664	neuron fate determination	3	0.0230
GO.0060341	regulation of cellular localization	28	0.0230
GO.0021872	forebrain generation of neurons	6	0.0231
GO.0031214	biomineral tissue development	6	0.0231
GO.0048814	regulation of dendrite morphogenesis	6	0.0231
GO.0048519	negative regulation of biological process	74	0.0232
GO.1901700	response to oxygen-containing compound	29	0.0245
GO.0010976	positive regulation of neuron projection development	10	0.0247
GO.0021761	limbic system development	7	0.0248
GO.0021766	hippocampus development	6	0.0265
GO.0043270	positive regulation of ion transport	10	0.0274
GO.0006928	movement of cell or subcellular component	27	0.0289
GO.0048568	embryonic organ development	16	0.0290
GO.0098662	inorganic cation transmembrane transport	15	0.0304
GO.0009893	positive regulation of metabolic process	62	0.0306
GO.0031345	negative regulation of cell	7	0.0306

	projection organization		
GO.1903532	positive regulation of secretion by cell	12	0.0309
GO.0006935	chemotaxis	14	0.0324
GO.0060326	cell chemotaxis	8	0.0324
GO.0001667	ameboidal-type cell migration	8	0.0336
GO.0060563	neuroepithelial cell differentiation	6	0.0336
GO.0061001	regulation of dendritic spine morphogenesis	4	0.0338
GO.0032835	glomerulus development	5	0.0354
GO.0042416	dopamine biosynthetic process	3	0.0359
GO.0009966	regulation of signal transduction	44	0.0363
GO.0031325	positive regulation of cellular metabolic process	53	0.0368
GO.0031914	negative regulation of synaptic plasticity	2	0.0371
GO.0046684	response to pyrethroid	2	0.0371
GO.0071864	positive regulation of cell proliferation in bone marrow	2	0.0371
GO.0071895	odontoblast differentiation	2	0.0371
GO.1903524	positive regulation of blood circulation	6	0.0371
GO.0021953	central nervous system neuron differentiation	9	0.0385
GO.0043583	ear development	10	0.0385
GO.0035725	sodium ion transmembrane transport	7	0.0393
GO.0035295	tube development	19	0.0396
GO.0006810	transport	58	0.0408
GO.0010770	positive regulation of cell morphogenesis involved in differentiation	8	0.0432
GO.0030902	hindbrain development	8	0.0432
GO.0001654	eye development	13	0.0446
GO.0032940	secretion by cell	13	0.0446
GO.0031175	neuron projection development	16	0.0450
GO.0051128	regulation of cellular component organization	43	0.0450
GO.0007166	cell surface receptor signaling pathway	33	0.0456
GO.0032350	regulation of hormone metabolic process	4	0.0456
GO.0050808	synapse organization	7	0.0467
GO.0043065	positive regulation of apoptotic process	16	0.0472
GO.0008217	regulation of blood pressure	8	0.0476
GO.0045935	positive regulation of nucleobase-containing compound metabolic process	36	0.0476
GO.0071805	potassium ion transmembrane transport	7	0.0483
GO.0010557	positive regulation of macromolecule biosynthetic process	35	0.0495
