# Supplementary material for: Thimet Oligopeptidase (EC 3.4.24.15) Key Functions Suggested by Knockout Mice Phenotype Characterization
Source: Biomolecules. 2019 Aug 19;9(8):382. doi: 10.3390/biom9080382 (PMC6722639; doi:10.3390/biom9080382)
Supplement: Supplementary file 1 [file biomolecules-09-00382-s001.zip › Supp - Individual MS identification and quantitation.pdf]

Thimet oligopeptidase (EC 3.4.24.15) key functions suggested by knockout mice phenotype characterization

Dos Santos, N.B.[1#], Franco, R.D.[1#], Camarini, R.[1], Munhoz, C.D.[1], Eichler, R.A.S.[1], Gewehr, M.C.F. [1], Reckziegel, P.[1], Llanos, R.P.[1], Dale, C.S. [2], Oliveira, V.R. [2], Borges, V.F.[3], Lima, B.H.F.[3], Cunha, F.Q.[3], Visniauskas, B.[4], Chagas, J.R.[4], Tufik, S.[4]., Peres, F.F.[5], Abilio, V.C.[5], Florio, J.C.[6], Iwai, L.K.[7], Rioli, V.[7], Prezoto, B.C.[8], Guimaraes, A.O.[9], Pesquero, J.B.[9], Bader, M. [10,11,12,13,14], Castro, L.M.[15], and Ferro, E.S.[1*]

Department of [1]Pharmacology and [2]Anatomy, Biomedical Sciences Institute, and [6]Department of Pathology, Veterinarian Medical School, University of São Paulo (USP), São Paulo, 05508-000; [3]Department of Pharmacology, Faculty of Medicine of Ribeirao Preto, University of São Paulo, Ribeirão Preto, SP, 14049-900; Departments of [4]Psychobiology, [5]Pharmacology and [9]Biophysics, Federal University of São Paulo (UNIFESP), São Paulo, 04023-062; [7]Special Laboratory of Applied Toxinology (LETA), Center of Toxins, Immune Response and Cell Signaling (CETICS), [8]Pharmacology Department, Butantan Institute, 05503-000, São Paulo;[10]Max-Delbrück-Center for Molecular Medicine, D-13125, Berlin, Germany, [11]Charité - Universitätsmedizin Berlin, [12]Berlin Institute of Health (BIH), Berlin, Germany, [13]DZHK (German Center for Cardiovascular Research), Partner Site Berlin, Berlin, Germany, [14]Institute for Biology, University of Lübeck, Lübeck, Germany, [15]Biosciences Institute, São Paulo State University (UNESP), São Vicente, 11330-900; SP, Brazil.

Running Title: THOP1 knockout mice

***To whom correspondence should be addressed:** Emer S. Ferro, PhD, Professor, Av. Prof. Lineu Prestes 1524, Sala 317, São Paulo, SP, 05508-000, Brazil, Phone: +55-11-3091-7310, Fax: +55-11-3091-7322, e-mail: *eferro@usp.br*

#These two authors contributed equally

Keywords: THOP1; neurodegeneration; inflammation; sepsis; MHC-I; peptidome.

Peptides individual MS identification and quantitation

Pre frontal cortex

Protein name	Peptide sequence	Elution time Run1	Elution time Run2	Elution time Run 3	m/z Light	m/z Heavy	Z	diff		T	m/z 1	mz/ 2	average m/z	SD	Observed mass	Theoretical mass	ppm	Peptide sequence	Ratio KO1/WT1	Ratio KO2/WT2	RATIO KO3/WT3	Average ratio	SE	n
Acyl-CoA-binding protein	VEKVDELKKKYGI	34,1	nd	40,5	845,0340	855,097	2	20	5,0	5	1547,8955	1548,028	1547,96175	0,09369	1547,9618	1.547,8996	40	VEKVDELKKKYGI	0,40	nd	0,39	0,40	0,005	2
Acyl-CoA-binding protein	VEKVDELKKKYGI	34,1	nd	40,5	563,6916	570,4003	3	20	5,0	5	1547,8943	1548,0269	1547,9606	0,09376	1547,9606	1.547,8996	39	VEKVDELKKKYGI	0,50	nd	0,30	0,40	0,100	2
Acyl-CoA-binding protein	KQATVGDVNTDRPGLLDL	50,2	57,0	55,4	656,6995	659,3848	3	8	2,0	2	1911,0119	1911,0704	1911,04115	0,04137	1911,0412	1.911,0134	15	KQATVGDVNTDRPGLLDL	0,66	0,46	0,25	0,46	0,118	3
Acyl-CoA-binding protein	TVGDVNTDRPGLLDL	59,6	63,5	62,1	806,9342	808,9464	2	4	1,0	1	1583,8211	1583,8468	1583,83395	0,01817	1583,8340	1.583,8228	7	TVGDVNTDRPGLLDL	0,98	0,62	0,32	0,64	0,191	3
Cerebellin-4	AANSKVAFSAVRSTN	25,7	33,1	31,9	789,9358	793,9625	2	8	2,0	2	1521,793	1521,849	1521,821	0,0396	1521,8210	1.521,7972	16	AANSKVAFSAVRSTN	1	1,33	1,02	1,12	0,107	3
Clathrin coat assembly protein AP180	ASKGLGSDLDSSLASL	61,1	63,5	63,5	788,9265	792,9523	2	8	2,0	2	1519,7744	1519,8286	1519,8015	0,03833	1519,8015	1.519,7802	14	ASKGLGSDLDSSLASL	0,75	0,42	0,42	0,53	0,110	3
Clathrin coat assembly protein AP180	SKGLGSDLDSSLASL	60,7	63,2	63,9	753,4092	757,4341	2	8	2,0	2	1448,7398	1448,7922	1448,766	0,03705	1448,7660	1.448,7431	16	SKGLGSDLDSSLASL	0,78	0,38	0,50	0,55	0,120	3
Clathrin coat assembly protein AP180	SPSPTPATQSPKKPPAKDPLADLNIKDFL	65,2	69,1	67,3	643,5716	647,5959	4	16	4,0	4	2458,1292	2458,2316	3072,6568	0,07241	3072,6568	3.072,6568	0	SPSPTPATQSPKKPPAKDPLADLNIKDFL	0,75	0,41	0,36	0,51	0,123	3
Clathrin coat assembly protein AP180	KGLGSDLDSSLASL	59,8	63,0	62,5	709,8929	713,9183	2	8	2,0	2	1361,7072	1361,7606	1361,7339	0,03776	1361,7339	1.361,7111	17	KGLGSDLDSSLASL	0,83	0,40	0,44	0,56	0,137	3
Cytochrome b-c1 complex subunit Rieske, mitochondrial	ARSGPFAPVLSAT	47,4	54,4	53,1	651,3831	653,3367	2	4	1,0	1	1272,7189	1272,6274	1272,67315	0,0647	1272,6732	1.272,6899	-13	ARSGPFAPVLSAT	0,95	1,05	0,44	0,81	0,189	3
Cytochrome b-c1 complex subunit Rieske, mitochondrial	SGQAAARPLVATV	33,0	40,3	39,5	634,8712	636,8837	2	4	1,0	1	1239,6951	1239,7214	1239,70825	0,0186	1239,7083	1.239,7008	6	SGQAAARPLVATV	0,88	0,91	0,46	0,75	0,144	3
Cytochrome b-c1 complex subunit Rieske, mitochondrial	GLNVPASVRF	51,8	56,3	57,1	544,3185	546,3308	2	4	1,0	1	1058,5897	1058,6156	1058,60265	0,01831	1058,6027	1.058,5946	8	GLNVPASVRF	0,88	1,33	0,63	0,94	0,206	3
Cytochrome b-c1 complex subunit Rieske, mitochondrial	TVGLNVPASVRF	58,6	61,2	60,6	644,3771	646,3301	2	4	1,0	1	1258,7069	1258,6142	1258,66055	0,06555	1258,6606	1.258,7106	-40	TVGLNVPASVRF	1,00	1,21	0,63	0,95	0,171	3
Cytochrome b-c1 complex subunit Rieske, mitochondrial	SGQAAARPLVA	23,7	31,4	29,6	534,8123	536,8249	2	4	1,0	1	1039,5773	1039,6038	1039,59055	0,01874	1039,5906	1.039,5847	6	SGQAAARPLVA	0,84	1,22	0,51	0,86	0,207	3
Cytochrome c oxidase subunit 5A, mitochondrial	NDFASAVRILEV	nd	75,0	72,0	681,3769	683,3907	2	4	1,0	1	1332,7065	1332,7354	1332,72095	0,02044	1332,7210	1.332,7110	7	NDFASAVRILEV	nd	0,47	0,24	0,36	0,115	2
Cytochrome c oxidase subunit 5A, mitochondrial	RPTLNELGISTPEELGLDKV	64,2	66,0	65,9	746,4207	749,1039	3	8	2,0	2	2180,1755	2180,2277	2180,2016	0,03691	2180,2016	2.180,1761	12	RPTLNELGISTPEELGLDKV	0,66	0,33	0,27	0,42	0,121	3
Cytochrome c oxidase subunit 5A, mitochondrial	GISTPEELGLDKV	53,9	67,0	57,6	707,3982	711,423	2	8	2,0	2	1356,7178	1356,77	1356,7439	0,03691	1356,7439	1.356,7209	17	GISTPEELGLDKV	0,54	0,55	0,38	0,49	0,055	3
Cytochrome c oxidase subunit 6A1, mitochondrial	HNPHVNPLPTGYEDE	32,1	38,6	37,7	873,9091	875,921	2	4	1,0	1	1717,7709	1717,796	1717,78345	0,01775	1717,7835	1.717,7769	4	HNPHVNPLPTGYEDE	1	0,97	0,61	0,86	0,127	3
LIM zinc-binding domain-containing Nebulette	TQVVSDAAYKGVQPHVV	37,8	44,0	43,4	618,6769	621,3605	3	8	2,0	2	1796,9441	1796,9975	1796,9708	0,03776	1796,9708	1.797,0492	-44	TQVVSDAAYKGVQPHVV	0,67	0,64	0,48	0,59	0,059	3
Macrophage migration inhibitory factor	AQATGKPAQYIAVHVVPDQL	54,5	57,4	56,2	721,4068	724,0897	3	8	2,0	2	2105,1338	2105,1851	2105,15945	0,03627	2105,1595	2.105,1342	12	AQATGKPAQYIAVHVVPDQL	0,80	0,40	0,57	0,59	0,115	3
Macrophage migration inhibitory factor	AQATGKPAQYIAVHVVPDQL	54,5	57,4	56,2	1081,6061	1085,6301	2	8	2,0	2	2105,1336	2105,1842	2105,1589	0,03578	2105,1589	2.105,1342	12	AQATGKPAQYIAVHVVPDQL	0,71	0,61	0,62	0,65	0,032	3
Macrophage migration inhibitory factor	AQATGKPAQYIA	26,4	33,4	32,2	637,8604	641,8852	2	8	2,0	2	1217,6422	1217,6944	1217,6683	0,03691	1217,6683	1.217,6477	17	AQATGKPAQYIA	1,11	0,93	0,71	0,92	0,116	3
Microtubule-associated protein tau	ADEVSASLAKQGL	40,8	46,8	46,7	672,8729	676,8983	2	8	2,0	2	1287,6672	1287,7206	1287,6939	0,03776	1287,6939	1.287,6743	15	ADEVSASLAKQGL	1,245	0,59	0,50	0,78	0,235	3
Neurogranin	GRKGPGPGGPGGAGGARGGAGGGPSGD	16,3	22,9	21,3	725,3692	728,0518	3	8	2,0	2	2117,021	2117,0714	2117,0184	0,03564	2117,0184	2.117,0184	0	GRKGPGPGGPGGAGGARGGAGGGPSGD	0,79	0,56	0,45	0,60	0,100	3
Neurogranin	KGPGPGGPGGAGGARGGAGGGPSGD	18,0	23,8	22,6	980,9863	985,0108	2	8	2,0	2	1903,894	1903,9456	1903,9198	0,03649	1903,9198	1.903,8958	13	KGPGPGGPGGAGGARGGAGGGPSGD	0,90	0,53	0,22	0,55	0,197	3
Neurogranin	RKGPGPGGPGGAGGARGGAGGGPSGD	16,2	22,8	21,2	706,3615	709,0445	3	8	2,0	2	2059,9979	2060,0495	2060,0237	0,03649	2060,0237	2.059,9969	13	RKGPGPGGPGGAGGARGGAGGGPSGD	0,71	1,34	0,77	0,94	0,200	3
Peptidyl-prolyl cis-trans isomerase A	EDENFILKHTGPGILSM	63,6	66,5	64,8	979,0121	983,0385	2	8	2,0	2	1899,9456	1900,001	1899,9733	0,03917	1899,9733	1.899,9473	14	EDENFILKHTGPGILSM	0,73	1,00	0,66	0,80	0,104	3
Peptidyl-prolyl cis-trans isomerase A	ELFADKVPKTAENFRAL	51,9	55,2	55,2	678,3856	682,3581	3	12	3,0	3	1948,0389	1947,9603	1947,9996	0,05558	1947,9996	1.948,0491	-25	ELFADKVPKTAENFRAL	0,52	0,75	1,68	0,98	0,355	3
Peptidyl-prolyl cis-trans isomerase A	LFADKVPKTAENFRAL	44,3	50,9	49,3	635,3727	639,3986	3	12	3,0	3	1819,0002	1819,0818	1819,041	0,0577	1819,0410	1.819,0065	19	LFADKVPKTAENFRAL	1,16	0,98	0,65	0,93	0,149	3
Peptidyl-prolyl cis-trans isomerase A	ADKVPKTAENFRAL	31,3	39,0	38,4	548,6557	552,6805	3	12	3,0	3	1558,8492	1558,9275	1558,88835	0,05537	1558,8884	1.558,8540	22	ADKVPKTAENFRAL	0,90	0,77	0,63	0,77	0,079	3
Peptidyl-prolyl cis-trans isomerase A	ITADDEPLGRVSF	55,5	60,5	59,1	724,3787	726,3914	2	4	1,0	1	1418,7101	1418,7368	1418,72345	0,01888	1418,7235	1.418,7114	8	ITADDEPLGRVSF	0,98	0,50	0,50	0,66	0,160	3
Peptidyl-prolyl cis-trans isomerase A	ADKVPKTAENF	25,9	33,0	32,3	652,3679	658,4055	2	12	3,0	3	1218,6259	1218,705	1218,66545	0,05593	1218,6655	1.218,6317	28	ADKVPKTAENF	0,75	0,93	0,45	0,71	0,140	3
Peptidyl-prolyl cis-trans isomerase A	ADDEPLGRVSF	47,5	54,7	54,3	617,3111	619,3237	2	4	1,0	1	1204,5749	1204,6014	1204,58815	0,01874	1204,5882	1.204,5797	7	ADDEPLGRVSF	1	0,98	0,58	0,85	0,137	3
Peptidyl-prolyl cis-trans isomerase FKBP1A	VFDVELLKLE	71,0	72,2	72,4	630,8781	634,9038	2	8	2,0	2	1203,6776	1203,7316	1203,7046	0,03818	1203,7046	1.203,6824	18	VFDVELLKLE	0,77	1,10	0,65	0,84	0,135	3
Phosphatidylethanolamine-binding protein 1	DDYVPKLYEQLSGK	56,6	60,2	59,6	580,3149	584,3407	3	12	3,0	3	1653,8268	1653,9081	1653,86745	0,05749	1653,8675	1.653,8323	21	DDYVPKLYEQLSGK	0,59	0,83	0,60	0,67	0,078	3
ProSAAS	SLSAASAPLVETSTPLRL	64,7	66,4	65,9	921,0261	923,039	2	4	1,0	1	1812,0049	1812,032	1812,01845	0,01916	1812,0185	1.812,0066	7	SLSAASAPLVETSTPLRL	0,75	1,00	1,00	0,92	0,083	3
Somatostatin	SANSNPAMAPRE	20,8	26,8	26,1	636,8049	638,8176	2	4	1,0	1	1243,5625	1243,5892	1243,57585	0,01888	1243,5759	1.243,5688	6	SANSNPAMAPRE	1,46	1,90	1,60	1,65	0,130	3
Somatostatin	SANSNPAMAPRE (methionine oxidation)	nd	23,4	22,9	644,8035	646,816	2	4	1,0	1	1259,5597	1259,586	1259,57285	0,0186	1259,5729	1.259,5637	7	SANSNPAMAPRE (OXI)	nd	2,50	1,56	2,03	0,470	2
Tubulin beta-3 chain	DDEESEAQGPK	nd	21,9	21,2	630,7849	634,8098	2	8	2,0	2	1203,4912	1203,5436	1203,5174	0,03705	1203,5174	1.203,4964	17	DDEESEAQGPK	nd	2,80	1,66	2,23	0,570	2

Abbreviations: z=charge; m/z=mass charge ratio; T=number of tags; nd= not detected

Peptides individual MS identification and quantitation

Striatum

Protein name	Peptide sequence	Elution time Run1	Elution time Run2	Elution time Run 3	m/z Light	m/ z Heavy	Z	diff		T	m/z 1	mz/ 2	average m/z	SD	Observed mass	Theoretical mass	ppm	Peptide sequence	Ratio KO1/WT1	Ratio KO2/WT2	RATIO KO3/WT3	Average ratio KO/WT	SE	n
Acyl-CoA-binding protein	ATVGDVNTDRPGLLDL	60,9	61,0	64,6	842,4515	844,4658	2	4	1,0	1	1654,8557	1654,8856	1654,87065	0,02114	1654,8707	1.654,8599	6	ATVGDVNTDRPGLLDL	0,87	1,66	0,60	1,04	0,318	3
Acyl-CoA-binding protein	KQATVGDVNTDRPGLLDL	51,8	52,5	57,5	984,5448	988,5694	2	8	2,0	2	1911,011	1911,0628	1911,0369	0,03663	1911,0369	1.911,0134	12	KQATVGDVNTDRPGLLDL	1,00	1,60	0,72	1,11	0,260	3
Acyl-CoA-binding protein	KQATVGDVNTDRPGLLDL	51,8	52,3	57,5	656,6985	659,3814	3	8	2,0	2	1911,0089	1911,0602	1911,03455	0,03627	1911,0346	1.911,0134	11	KQATVGDVNTDRPGLLDL	0,95	1,52	0,61	1,03	0,265	3
Acyl-CoA-binding protein	SHFKQATVGDVNTDRPGLLDL	50,9	51,1	56,5	1170,1259	1174,1481	2	8	2,0	2	2282,1732	2282,2202	2282,1967	0,03323	2282,1967	2.282,1728	10	SHFKQATVGDVNTDRPGLLDL	0,94	2,15	1,00	1,36	0,394	3
Acyl-CoA-binding protein	TVGDVNTDRPGLLDL	59,7	60,0	63,8	806,9318	808,944	2	4	1,0	1	1583,8163	1583,842	1583,82915	0,01817	1583,8292	1.583,8228	4	TVGDVNTDRPGLLDL	0,86	1,68	0,68	1,07	0,308	3
Acyl-CoA-binding protein	VEKVDELKKKYGI	36,6	36,4	41,3	563,6907	570,3989	3	20	5,0	5	1547,8916	1548,0227	1547,95715	0,0927	1547,9572	1.547,8996	37	VEKVDELKKKYGI	1,16	0,90	0,66	0,91	0,144	3
Clathrin coat assembly protein AP180	ASKGLGSDLDSSLASL	61,0	61,0	64,2	788,9254	792,9509	2	8	2,0	2	1519,7722	1519,8258	1519,799	0,0379	1519,7990	1.519,7802	12	ASKGLGSDLDSSLASL	1,10	1,28	0,87	1,08	0,119	3
Clathrin coat assembly protein AP180	SPSPTPATQSPKKPPAKDPLADLNIKDFL	67,8	67,7	68,7	804,2106	809,2419	4	20	5,0	5	3072,6539	3072,7856	3072,71975	0,09313	3072,7198	3.072,6568	20	SPSPTPATQSPKKPPAKDPLADLNIKDFL	1,00	1,53	0,97	1,17	0,182	3
Cysteine and glycine-rich protein 1	GQGAGALVHSE	23,8	23,5	27,9	527,2706	529,2832	2	4	1,0	1	1024,4939	1024,5204	1024,50715	0,01874	1024,5072	1.024,5010	6	GQGAGALVHSE	1,05	1,10	1,10	1,08	0,017	3
Cytochrome b-c1 complex subunit Rieske, mitochondrial	SGQAAARPLVATV	35,8	35,7	40,2	634,8714	636,8836	2	4	1,0	1	1239,6955	1239,7212	1239,70835	0,01817	1239,7084	1.239,7008	6	SGQAAARPLVATV	1,00	1,10	1,65	1,25	0,202	3
Cytochrome b-c1 complex subunit Rieske, mitochondrial	TVGLNVPASVRF	58,8	59,2	60,7	644,3762	646,3892	2	4	1,0	1	1258,7051	1258,7324	1258,71875	0,0193	1258,7188	1.258,7106	6	TVGLNVPASVRF	1,48	1,05	0,98	1,17	0,156	3
Cytochrome c oxidase subunit 5A, mitochondrial	GISTPEELGLDKV	54,5	54,8	58,2	707,3970	711,4219	2	8	2,0	2	1356,7154	1356,7678	1356,7416	0,03705	1356,7416	1.356,7209	15	GISTPEELGLDKV	1,00	1,40	0,75	1,05	0,189	3
Cytochrome c oxidase subunit 5A, mitochondrial	RPTLNELGISTPEELGLDKV	64,9	65,2	66,0	746,4198	749,1033	3	8	2,0	2	2180,1728	2180,2259	2180,19935	0,03755	2180,1994	2.180,1761	11	RPTLNELGISTPEELGLDKV	1,25	1,60	0,75	1,20	0,247	3
Cytochrome c oxidase subunit 6A1, mitochondrial	FHNPHVNPLPTGYEDE	39,3	39,1	43,4	947,4431	949,4562	2	4	1,0	1	1864,8389	1864,8664	1864,85265	0,01945	1864,8527	1.864,8453	4	FHNPHVNPLPTGYEDE	1,06	1,33	0,87	1,09	0,133	3
Cytochrome c oxidase subunit 6A1, mitochondrial	HNPHVNPLPTGYEDE	33,8	34,0	38,1	873,9086	875,921	2	4	1,0	1	1717,7699	1717,796	1717,78295	0,01846	1717,7830	1.717,7769	4	HNPHVNPLPTGYEDE	1,14	1,75	0,50	1,13	0,361	3
Glyceraldehyde-3-phosphate dehydrogenase	AFRVPTPNVSVVDL	68,3	68,1	69,1	771,4396	773,4476	2	4	1,0	1	1512,8319	1512,8492	1512,84055	0,01223	1512,8406	1.512,8373	2	AFRVPTPNVSVVDL	1,33	1,60	1,60	1,51	0,090	3
Macrophage migration inhibitory factor	AQATGKPAQYIAVHVVPDQL	54,3	54,6	57,1	721,4048	724,0881	3	8	2,0	2	2105,1278	2105,1803	2105,15405	0,03712	2105,1541	2.105,1342	9	AQATGKPAQYIAVHVVPDQL	1,10	1,10	1,32	1,17	0,073	3
Microtubule-associated protein tau	ADEVSASLAKQGL	42,3	42,4	47,5	672,8734	676,8988	2	8	2,0	2	1287,6682	1287,7216	1287,6949	0,03776	1287,6949	1.287,6743	16	ADEVSASLAKQGL	1,00	1,02	1,50	1,17	0,163	3
Neurogranin	GRKGPGPGGPGGAGGARGGAGGGPSGD	17,8	17,1	21,9	544,2773	546,2896	4	8	2,0	2	2117,0146	2117,0664	2117,0405	0,03663	2117,0405	2.117,0184	10	GRKGPGPGGPGGAGGARGGAGGGPSGD	0,89	0,78	0,85	0,84	0,032	3
Neurogranin	KGPGPGGPGGAGGARGGAGGGPSGD	19,2	18,6	23,1	654,3266	657,0099	3	8	2,0	2	1903,8932	1903,9457	1903,91945	0,03712	1903,9195	1.903,8958	12	KGPGPGGPGGAGGARGGAGGGPSGD	0,78	0,77	0,79	0,78	0,006	3
Neurogranin	KGPGPGGPGGAGGARGGAGGGPSGD	18,9	18,9	23,1	980,9834	985,008	2	8	2,0	2	1903,8882	1903,94	1903,9141	0,03663	1903,9141	1.903,8958	10	KGPGPGGPGGAGGARGGAGGGPSGD	0,62	0,69	0,62	0,64	0,023	3
Neurogranin	RKGPGPGGPGGAGGARGGAGGGPSGD	17,3	16,9	21,8	706,3609	709,044	3	8	2,0	2	2059,9961	2060,048	2060,02205	0,0367	2060,0221	2.059,9969	12	RKGPGPGGPGGAGGARGGAGGGPSGD	1,16	2,30	0,39	1,28	0,555	3
Peptidyl-prolyl cis-trans isomerase A	ADDEPLGRVSF	49,1	49,5	55,2	617,3094	619,3221	2	4	1,0	1	1204,5715	1204,5982	1204,58485	0,01888	1204,5849	1.204,5797	4	ADDEPLGRVSF	1,08	1,08	1,15	1,10	0,023	3
Peptidyl-prolyl cis-trans isomerase A	ADKVPKTAENFRAL	33,7	33,5	38,2	822,4785	828,5163	2	12	3,0	3	1558,8471	1558,9266	1558,88685	0,05621	1558,8869	1.558,8540	21	ADKVPKTAENFRAL	1,10	1,02	1,65	1,26	0,198	3
Peptidyl-prolyl cis-trans isomerase A	ADKVPKTAENFR	23,8	23,7	nd	487,2813	491,3062	3	12	3,0	3	1374,726	1374,8046	1374,7653	0,05558	1374,7653	1.374,7328	24	ADKVPKTAENFR	1,06	1,66	nd	1,36	0,300	2
Peptidyl-prolyl cis-trans isomerase A	ELFADKVPKTAENF	48,3	48,7	nd	846,9666	853,0028	2	12	3,0	3	1607,8233	1607,8996	1607,86145	0,05395	1607,8615	1.607,8268	22	ELFADKVPKTAENF	0,96	1,42	nd	1,19	0,230	2
Phosphatidylethanolamine-binding protein 1	KGNDISSGTVLSDYVGSGPPSGTGL	61,4	61,6	nd	1211,1600	1215,1393	2	8	2,0	2	2364,2414	2364,2026	2364,222	0,02744	2364,2220	2.364,1518	30	KGNDISSGTVLSDYVGSGPPSGTGL	1,33	1,42	nd	1,38	0,045	2
Phosphatidylethanolamine-binding protein 1	AGVTVDELGKVL	60,5	60,9	nd	628,8796	632,9054	2	8	2,0	2	1199,6806	1199,7348	1199,7077	0,03833	1199,7077	1199,6834	20	AGVTVDELGKVL	0,87	1,35	nd	1,11	0,240	2
Phosphatidylethanolamine-binding protein 1	GVTVDELGKVLTPTQV	64,5	64,9	66,4	856,5344	860,5231	2	8	2,0	2	1654,9902	1654,9702	1654,9802	0,01414	1654,9802	1.654,9214	36	GVTVDELGKVLTPTQV	0,83	1,50	1,33	1,22	0,201	3
Phosphatidylethanolamine-binding protein 1	DDYVPKLYEQLSGK	56,9	57,2	60,5	580,3149	584,3407	3	12	3,0	3	1653,8268	1653,9081	1653,86745	0,05749	1653,8675	1.653,8323	21	DDYVPKLYEQLSGK	0,85	1,42	1,06	1,11	0,166	3
Phosphatidylethanolamine-binding protein 1	AGVTVDELGKVLTPTQV	65,6	65,7	67,0	892,0162	896,0409	2	8	2,0	2	1725,9538	1726,0058	1725,9798	0,03677	1725,9798	1.725,9585	12	AGVTVDELGKVLTPTQV	1,33	1,40	1,46	1,40	0,038	3
Proenkephalin-A	SPQLEDEAKELQ	38,5	38,4	44,2	721,8737	725,8987	2	8	2,0	2	1385,6688	1385,7214	1385,6951	0,03719	1385,6951	1.385,6747	15	SPQLEDEAKELQ	1,10	0,25	0,71	0,69	0,246	3
ProSAAS	ASAPLVETSTPLRL	57,8	58,5	nd	741,9335	743,9453	2	4	1,0	1	1453,8197	1453,8446	1453,83215	0,01761	1453,8322	1.453,8213	7	ASAPLVETSTPLRL	1,42	0,99	nd	1,20	0,216	2
ProSAAS	SLSAASAPLVETSTPLRL	66,0	66,0	67,1	921,0241	923,0366	2	4	1,0	1	1812,0009	1812,0272	1812,01405	0,0186	1812,0141	1.812,0066	4	SLSAASAPLVETSTPLRL	1,43	1,60	0,58	1,20	0,316	3
Secretogranin-1	SFARAPQLDL	51,6	52,5	57,2	573,3200	575,3326	2	4	1,0	1	1116,5927	1116,6192	1116,60595	0,01874	1116,6060	1.116,6000	5	SFARAPQLDL	2,00	1,00	1,00	1,33	0,333	3
Secretogranin-1	LLDEGHYPVRESPIDTA	41,6	41,5	46,2	970,4936	972,5087	2	4	1,0	1	1910,9399	1910,9714	1910,95565	0,02227	1910,9557	1.910,9447	6	LLDEGHYPVRESPIDTA	1,52	1,60	2,00	1,71	0,148	3
Somatostatin	SANSNPAMAPRE	21,8	21,4	25,7	636,8052	638,8178	2	4	1,0	1	1243,5631	1243,5896	1243,57635	0,01874	1243,5764	1.243,5688	6	SANSNPAMAPRE	2,85	0,75	2,50	2,03	0,650	3
Somatostatin	SANSNPAMAPRE (methionine oxidation)	18,2	17,9	22,8	644,8033	646,8157	2	4	1,0	1	1259,5593	1259,5854	1259,57235	0,01846	1259,5724	1.259,5637	7	SANSNPAMAPRE (methionine oxidation)	2,66	0,87	2,00	1,84	0,523	3
Synapsin-1	AGGPPHPQLNKS	22,4	22,1	nd	629,8502	633,8749	2	8	2,0	2	1201,6218	1201,6738	1201,6478	0,03677	1201,6478	1.201,6276	17	AGGPPHPQLNKS	1,25	0,68	nd	0,97	0,285	2
Tubulin beta-2A chain	SGPFGQIFRPDNF	67,5	67,4	69,7	755,3795	757,3921	2	4	1,0	1	1480,7117	1480,7382	1480,72495	0,01874	1480,7250	1.480,7172	5	SGPFGQIFRPDNF	0,97	0,75	1,48	1,07	0,216	3

Peptides individual MS identification and quantitation

Hippocampus

Protein name	Peptide sequence	Elution time Run1	Elution time Run2	Elution time Run 3	m/z Light	m/z Heavy	Z	diff		T	m/z 1	mz/ 2	average m/z	SD	Observed mass	Theoretical mass	ppm	Peptide sequence	Ratio KO1/WT1	Ratio KO2/WT2	RATIO KO3/WT3	Average ratio	SE	n
Acyl-CoA-binding protein	ATVGDVNTDRPGLLDL	60,3	57,6	64,2	842,4547	844,4669	2	4	1,0	1	1654,8621	1654,8878	1654,87495	0,01817264	1654,8750	1.654,8599	9	ATVGDVNTDRPGLLDL	0,72	1,66	3,00	1,79	0,662	3
Acyl-CoA-binding protein	GDVNTDRPGLLDL	53,2	51,8	61,1	706,8754	708,8877	2	4	1,0	1	1383,7035	1383,7294	1383,71645	0,01831407	1383,7165	1.383,7067	7	GDVNTDRPGLLDL	1,07	1,55	3,01	1,88	0,583	3
Acyl-CoA-binding protein	KQATVGDVNTDRPGLLDL	48,9	47,4	56,9	984,5476	988,5691	2	8	2,0	2	1911,0166	1911,0622	1911,0394	0,03224407	1911,0394	1.911,0134	14	KQATVGDVNTDRPGLLDL	0,77	2,00	4,72	2,49	1,168	3
Acyl-CoA-binding protein	KQATVGDVNTDRPGLLDL	48,9	47,4	56,9	656,6997	659,381	3	8	2,0	2	1911,0125	1911,059	1911,03575	0,03288047	1911,0358	1.911,0134	12	KQATVGDVNTDRPGLLDL	0,75	1,76	4,00	2,17	0,961	3
Acyl-CoA-binding protein	TVGDVNTDRPGLLDL	58,8	56,4	63,7	806,9332	808,9454	2	4	1,0	1	1583,8191	1583,8448	1583,83195	0,01817264	1583,8320	1.583,8228	6	TVGDVNTDRPGLLDL	0,85	1,82	5,25	2,64	1,335	3
Acyl-CoA-binding protein	VEKVDELKKKYGI	34,4	34,4	41,6	563,6919	570,4003	3	20	5,0	5	1547,8952	1548,0269	1547,96105	0,09312596	1547,9611	1.547,8996	40	VEKVDELKKKYGI	1,00	1,75	4,00	2,25	0,901	3
Acyl-CoA-binding protein	VEKVDELKKKYGI	34,3	34,5	41,6	845,0334	855,0981	2	20	5,0	5	1547,8943	1548,0302	1547,96225	0,09609581	1547,9623	1.547,8996	40	VEKVDELKKKYGI	0,87	1,93	3,40	2,07	0,734	3
ATP synthase-coupling factor 6, mitochondrial	KFDDPKFEVIDKPQS	38,0	37,5	nd	953,0265	961,0734	2	16	4,0	4	1791,9118	1792,0108	1791,9613	0,07000357	1791,9613	1791,9116	28	KFDDPKFEVIDKPQS	0,87	1,38	nd	1,12	0,255	2
Cerebellin-4	SKVAFSAVRSTN	25,0	24,1	29,6	441,5870	444,2707	3	8	2,0	2	1265,6744	1265,7281	1265,70125	0,03797163	1265,7013	1.265,6801	17	SKVAFSAVRSTN	1,33	0,65	0,70	0,89	0,216	3
Clathrin coat assembly protein AP180	ASKGLGSDLDSSLASL	60,8	58,5	64,2	788,9260	792,9506	2	8	2,0	2	1519,7734	1519,8252	1519,7993	0,03662813	1519,7993	1.519,7802	13	ASKGLGSDLDSSLASL	1,11	1,07	0,97	1,05	0,042	3
Clathrin coat assembly protein AP180	SPSPTPATQSPKKPPAKDPLADLNIKDFL	66,0	62,2	68,7	804,2125	809,244	4	20	5,0	5	3072,6615	3072,794	3072,72775	0,09369165	3072,7278	3.072,6568	23	SPSPTPATQSPKKPPAKDPLADLNIKDFL	1,12	0,93	0,92	0,99	0,065	3
Cysteine and glycine-rich protein 1	GQGAGALVHSE	23,2	22,6	27,9	527,2711	529,2838	2	4	1,0	1	1024,4949	1024,5216	1024,50825	0,01887975	1024,50825	1024,501	7	GQGAGALVHSE	0,90	3,00	2,60	2,17	0,644	3
Cytochrome b-c1 complex subunit Rieske, mitochondrial	SGQAAARPLVA	24,8	24,5	29,1	534,8131	536,8254	2	4	1,0	1	1039,5789	1039,6048	1039,59185	0,01831407	1039,5919	1.039,5847	7	SGQAAARPLVA	1,08	0,80	1,14	1,01	0,103	3
Cytochrome b-c1 complex subunit Rieske, mitochondrial	SGQAAARPLVATV	33,6	33,4	40,2	634,8714	636,8836	2	4	1,0	1	1239,6955	1239,7212	1239,70835	0,01817264	1239,7084	1.239,7008	6	SGQAAARPLVATV	0,99	0,78	1,05	0,94	0,083	3
Cytochrome b-c1 complex subunit Rieske, mitochondrial	TVGLNVPASVRF	57,3	54,7	60,7	644,3773	646,3898	2	4	1,0	1	1258,7073	1258,7336	1258,72045	0,01859691	1258,7205	1.258,7106	8	TVGLNVPASVRF	1,15	0,71	1,07	0,98	0,136	3
Cytochrome c oxidase subunit 5A, mitochondrial	GISTPEELGLDKV	51,9	50,5	58,2	707,3984	711,4236	2	8	2,0	2	1356,7182	1356,7712	1356,7447	0,03747666	1356,7447	1.356,7209	18	GISTPEELGLDKV	0,84	1,77	1,93	1,51	0,339	3
Cytochrome c oxidase subunit 5A, mitochondrial	NDFASAVRILEV	69,8	68,2	72,5	681,3779	683,3907	2	4	1,0	1	1332,7085	1332,7354	1332,72195	0,01902117	1332,7220	1.332,7110	8	NDFASAVRILEV	0,95	2,00	2,15	1,70	0,376	3
Cytochrome c oxidase subunit 5A, mitochondrial	RPTLNELGISTPEELGLDKV	64,6	62,5	66,1	746,4225	749,1057	3	8	2,0	2	2180,1809	2180,2331	2180,207	0,03691097	2180,2070	2.180,1761	14	RPTLNELGISTPEELGLDKV	0,79	1,80	2,00	1,53	0,374	3
Cytochrome c oxidase subunit 6A1, mitochondrial	FHNPHVNPLPTGYEDE	36,9	37,4	44,1	947,4456	949,4577	2	4	1,0	1	1864,8439	1864,8694	1864,85665	0,01803122	1864,8567	1.864,8453	6	FHNPHVNPLPTGYEDE	1,00	0,96	1,40	1,12	0,139	3
Cytochrome c oxidase subunit 6A1, mitochondrial	HNPHVNPLPTGYEDE	32,6	32,6	37,4	873,9091	875,922	2	4	1,0	1	1717,7709	1717,798	1717,78445	0,01916259	1717,7845	1.717,7769	4	HNPHVNPLPTGYEDE	0,98	1,27	1,66	1,30	0,198	3
Cytochrome c oxidase subunit 6A1, mitochondrial	NPHVNPLPTGYEDE	37,2	36,9	43,4	805,3803	807,3929	2	4	1,0	1	1580,7133	1580,7398	1580,72655	0,01873833	1580,7266	1.580,7180	5	NPHVNPLPTGYEDE	1,11	0,74	1,36	1,07	0,180	3
Gamma-enolase	AGNSDLILPVPAFNVINGGSHAGNKL	71,7	69,5	72,8	877,8140	880,4963	3	8	2,0	2	2574,3554	2574,4049	2574,38015	0,03500179	2574,3802	2.574,3627	7	AGNSDLILPVPAFNVINGGSHAGNKL	1,11	0,42	0,74	0,76	0,198	3
Glyceraldehyde-3-phosphate dehydrogenase	AFRVPTPNVSVVDL	66,9	66,1	69,1	771,4396	773,4497	2	4	1,0	1	1512,8319	1512,8534	1512,84265	0,0152028	1512,8427	1.512,8373	4	AFRVPTPNVSVVDL	0,83	1,14	1,00	0,99	0,088	3
LIM zinc-binding domain-containing Nebulette	TQVVSDAAYKGVQPHVV	38,1	37,6	44,6	927,5132	931,5369	2	8	2,0	2	1796,9478	1796,9978	1796,9728	0,03535534	1796,9728	1.796,9494	13	TQVVSDAAYKGVQPHVV	1,30	0,53	0,95	0,93	0,223	3
Macrophage migration inhibitory factor	AQATGKPAQYIA	27,1	26,7	nd	637,8605	641,8856	2	8	2,0	2	1217,6424	1217,6952	1217,6688	0,03733524	1217,6688	1.217,6477	17	AQATGKPAQYIA	1,15	1,18	nd	1,17	0,017	2
Macrophage migration inhibitory factor	AQATGKPAQYIAVHVVPDQL	50,9	49,3	57,3	1081,6071	1.085,6313	2	8	2,0	2	2105,1356	2105,1866	2105,1611	0,03606245	2105,1611	2.105,1342	13	AQATGKPAQYIAVHVVPDQL	1,02	0,71	0,51	0,75	0,150	3
Macrophage migration inhibitory factor	AQATGKPAQYIAVHVVPDQL	50,9	49,9	57,3	721,4066	724,0896	3	8	2,0	2	2105,1332	2105,1848	2105,159	0,03648671	2105,1590	2.105,1342	12	AQATGKPAQYIAVHVVPDQL	0,98	0,75	0,88	0,87	0,068	3
Microtubule-associated protein 2	AEDVTAALAKQGL	48,1	47,4	54,4	671,8840	675,9103	2	8	2,0	2	1285,6894	1285,7446	1285,717	0,03903229	1285,7170	1.285,6951	17	AEDVTAALAKQGL	0,99	0,78	0,86	0,87	0,060	3
Microtubule-associated protein tau	ADEVSASLAKQGL	41,1	40,4	47,5	672,8743	676,8996	2	8	2,0	2	1287,67	1287,7232	1287,6966	0,03761808	1287,6966	1.287,6743	17	ADEVSASLAKQGL	1,12	0,71	1,03	0,95	0,122	3
Neurogranin	GRKGPGPGGPGGAGGARGGAGGGPSGD	16,4	16,1	21,4	725,3685	728,0512	3	8	2,0	2	2117,0189	2117,0696	2117,04425	0,03585031	2117,0443	2.117,0184	12	GRKGPGPGGPGGAGGARGGAGGGPSGD	0,85	1,68	0,71	1,08	0,301	3
Neurogranin	KGPGPGGPGGAGGARGGAGGGPSGD	18,7	18,0	22,9	980,9864	985,0126	2	8	2,0	2	1903,8942	1903,9492	1903,9217	0,03889087	1903,9217	1.903,8958	14	KGPGPGGPGGAGGARGGAGGGPSGD	0,86	2,08	1,81	1,58	0,370	3
Neurogranin	KGPGPGGPGGAGGARGGAGGGPSGD	18,7	17,9	22,9	654,3271	657,0103	3	8	2,0	2	1903,8947	1903,9469	1903,9208	0,03691097	1903,9208	1.903,8958	13	KGPGPGGPGGAGGARGGAGGGPSGD	0,97	2,27	1,66	1,63	0,374	3
Neurogranin	RKGPGPGGPGGAGGARGGAGGGPSGD	17,0	16,1	21,7	706,3608	709,044	3	8	2,0	2	2059,9958	2060,0480	2060,0219	0,03691097	2060,0219	2.059,9969	12	RKGPGPGGPGGAGGARGGAGGGPSGD	1,23	1,16	2,33	1,57	0,379	3
Peptidyl-prolyl cis-trans isomerase A	FDITADDEPLGRVSF	67,4	67,4	71,0	855,4278	857,4405	2	4	1,0	1	1680,8083	1680,835	1680,82165	0,01887975	1680,8217	1.680,8068	9	FDITADDEPLGRVSF	1,10	1,37	0,61	1,03	0,221	3
Peptidyl-prolyl cis-trans isomerase A	ITADDEPLGRVSF	53,7	52,0	59,8	724,3763	726,3896	2	4	1,0	1	1418,7053	1418,7332	1418,71925	0,01972828	1418,7193	1.418,7114	6	ITADDEPLGRVSF	1,04	0,62	0,98	0,88	0,130	3
Peptidyl-prolyl cis-trans isomerase A	LFADKVPKTAENF	40,4	39,4	47,1	521,9648	525,9901	3	12	3,0	3	1478,7765	1478,8563	1478,8164	0,05642712	1478,8164	1.478,7842	22	LFADKVPKTAENF	1,36	0,59	0,92	0,96	0,223	3
Peptidyl-prolyl cis-trans isomerase FKBP1A	VFDVELLKLE	70,3	68,5	nd	630,8798	634,905	2	8	2,0	2	1203,681	1203,7338	1203,7074	0,03733524	1203,7074	1.203,6824	21	VFDVELLKLE	0,82	1,25	nd	1,03	0,216	2
Phosphatidylethanolamine-binding protein 1	KGNDISSGTVL	31,3	31,3	37,5	573,8236	577,8486	2	8	2,0	2	1089,5686	1089,6212	1089,5949	0,03719382	1089,5949	1.089,5739	19	KGNDISSGTVL	1,14	0,87	1,28	1,10	0,120	3
Phosphatidylethanolamine-binding protein 1	GLDPGKLYTL	53,8	52,0	60,6	566,8365	570,8616	2	8	2,0	2	1075,5944	1075,6472	1075,6208	0,03733524	1075,6208	1.075,5986	21	GLDPGKLYTL	1,00	0,50	0,80	0,77	0,145	3
Phosphatidylethanolamine-binding protein 1	DGLDPGKLYTL	59,3	57,3	63,5	624,3498	628,3755	2	8	2,0	2	1190,621	1190,675	1190,648	0,03818377	1190,6480	1.190,6256	19	DGLDPGKLYTL	0,75	1,00	1,42	1,06	0,195	3
Phosphatidylethanolamine-binding protein 1	DDYVPKLYEQLSGK	53,5	53,6	60,5	580,3162	584,3405	3	12	3,0	3	1653,8307	1653,9075	1653,8691	0,0543058	1653,8691	1.653,8323	22	DDYVPKLYEQLSGK	0,85	0,68	0,85	0,79	0,057	3
Phosphatidylethanolamine-binding protein 1	AGVTVDELGKVLTPTQV	64,9	63,6	67,0	892,0186	896,0447	2	8	2,0	2	1725,9586	1726,0134	1725,986	0,03874945	1725,9860	1.725,9585	16	AGVTVDELGKVLTPTQV	0,95	0,60	0,88	0,81	0,107	3
Proenkephalin-A	SPQLEDEAKELQ	36,9	36,8	44,2	721,8748	725,8996	2	8	2,0	2	1385,671	1385,7232	1385,6971	0,03691097	1385,6971	1.385,6747	16	SPQLEDEAKELQ	1,04	0,43	1,00	0,83	0,196	3
ProSAAS	SLSAASAPLVETSTPLRL	64,7	62,4	nd	921,0273	923,039	2	4	1,0	1	1812,0073	1812,032	1812,01965	0,01746554	1812,0197	1.812,0066	7	SLSAASAPLVETSTPLRL	1,00	0,68	nd	0,84	0,161	2
Secretogranin-1	SFARAPQLDL	48,4	47,6	57,2	573,3215	575,3341	2	4	1,0	1	1116,5957	1116,6222	1116,60895	0,01873833	1116,6090	1.116,6000	8	SFARAPQLDL	1,46	0,46	0,62	0,85	0,310	3
Somatostatin	ANSNPAMAPRE (oxi)	18,1	17,5	22,0	601,2894	603,2997	2	4	1,0	1	1172,5315	1172,5534	1172,54245	0,01548564	1172,5425	1.172,5317	9	ANSNPAMAPRE (methionine oxidation)	1,85	1,46	1,87	1,73	0,133	3
Somatostatin	SANSNPAMAPRE	21,2	20,8	25,6	636,8058	638,8181	2	4	1,0	1	1243,5643	1243,5902	1243,57725	0,01831407	1243,5773	1.243,5688	7	SANSNPAMAPRE	1,60	0,53	1,17	1,10	0,311	3
Tubulin beta-2A chain	SGPFGQIFRPDNF	66,1	65,2	69,7	755,3773	757,3903	2	4	1,0	1	1480,7073	1480,7346	1480,72095	0,01930402	1480,7210	1.480,7172	3	SGPFGQIFRPDNF	0,83	0,82	0,76	0,80	0,022	3
Tubulin beta-3 chain	DDEESEAQGPK	17,3	nd	21,1	630,7856	634,8118	2	8	2,0	2	1203,4926	1203,5476	1203,5201	0,03889087	1203,5201	1.203,4964	20	DDEESEAQGPK	1,36	nd	1,84	1,60	0,240	2
