# Supplementary material for: Thimet Oligopeptidase (EC 3.4.24.15) Key Functions Suggested by Knockout Mice Phenotype Characterization
Source: Biomolecules. 2019 Aug 19;9(8):382. doi: 10.3390/biom9080382 (PMC6722639; doi:10.3390/biom9080382)

Thimet oligopeptidase (EC 3.4.24.15) key functions suggested by knockout mice phenotype characterization

Dos Santos, N.B.[1#], Franco, R.D.[1#], Camarini, R.[1], Munhoz, C.D.[1], Eichler, R.A.S.[1], Gewehr, M.C.F. [1], Reckziegel, P.[1], Llanos, R.P.[1], Dale, C.S. [2], Oliveira, V.R. [2], Borges, V.F.[3], Lima, B.H.F.[3], Cunha, F.Q.[3], Visniauskas, B.[4], Chagas, J.R.[4], Tufik, S.[4]., Peres, F.F.[5], Abilio, V.C.[5], Florio, J.C.[6], Iwai, L.K.[7], Rioli, V.[7], Prezoto, B.C.[8], Guimaraes, A.O.[9], Pesquero, J.B.[9], Bader, M. [10,11,12,13,14], Castro, L.M.[15], and Ferro, E.S.[1*]

Department of [1]Pharmacology and [2]Anatomy, Biomedical Sciences Institute, and [6]Department of Pathology, Veterinarian Medical School, University of São Paulo (USP), São Paulo, 05508-000; [3]Department of Pharmacology, Faculty of Medicine of Ribeirao Preto, University of São Paulo, Ribeirão Preto, SP, 14049-900; Departments of [4]Psychobiology, [5]Pharmacology and [9]Biophysics, Federal University of São Paulo (UNIFESP), São Paulo, 04023-062; [7]Special Laboratory of Applied Toxinology (LETA), Center of Toxins, Immune Response and Cell Signaling (CETICS), [8]Pharmacology Department, Butantan Institute, 05503-000, São Paulo;[10]Max-Delbrück-Center for Molecular Medicine, D-13125, Berlin, Germany, [11]Charité - Universitätsmedizin Berlin, [12]Berlin Institute of Health (BIH), Berlin, Germany, [13]DZHK (German Center for Cardiovascular Research), Partner Site Berlin, Berlin, Germany, [14]Institute for Biology, University of Lübeck, Lübeck, Germany, [15]Biosciences Institute, São Paulo State University (UNESP), São Vicente, 11330-900; SP, Brazil.

Running Title: THOP1 knockout mice

***To whom correspondence should be addressed:** Emer S. Ferro, PhD, Professor, Av. Prof. Lineu Prestes 1524, Sala 317, São Paulo, SP, 05508-000, Brazil, Phone: +55-11-3091-7310, Fax: +55-11-3091-7322, e-mail: *eferro@usp.br*

#These two authors contributed equally

Keywords: THOP1; neurodegeneration; inflammation; sepsis; MHC-I; peptidome.

Organization of MS supplementary information

1. Peptide Identification (Mascot search results).....................pages 02-136
2. Peptide Quantitation
2.1 Diagram of Dimethyl labeling..page 137
2.2 MS spectra from cortex peptides......................................pages 138-178
2.3 MS spectra from striatum peptides...................................pages 179-219
2.4 MS spectra from hippocampus peptides..........................pages 220-268

[redacted] Mascot Search Results

Peptide View

MS/MS Fragmentation of ATVGDVNTDRPGLLDL
Found in ACBP_MOUSE in SwissProt, Acyl-CoA-binding protein OS=Mus musculus GN=Dbi PE=1 SV=2

Match to Query 6508: 1682.897688 from(842.456120,2+) intensity(13946.9990) scans(9669) rawscans(sn9669) rtinseconds(3650.2103) index(7078)
Title: 7079: Scan 9669 (rt=3650.21) [C:\Users\Administrador\Desktop\AMOSTRAS NO ORBITRAP LEANDRO RODAR NOVAMENTE\CORTEX\cortex_01.raw]
Data file cortex_01.temp.mgf

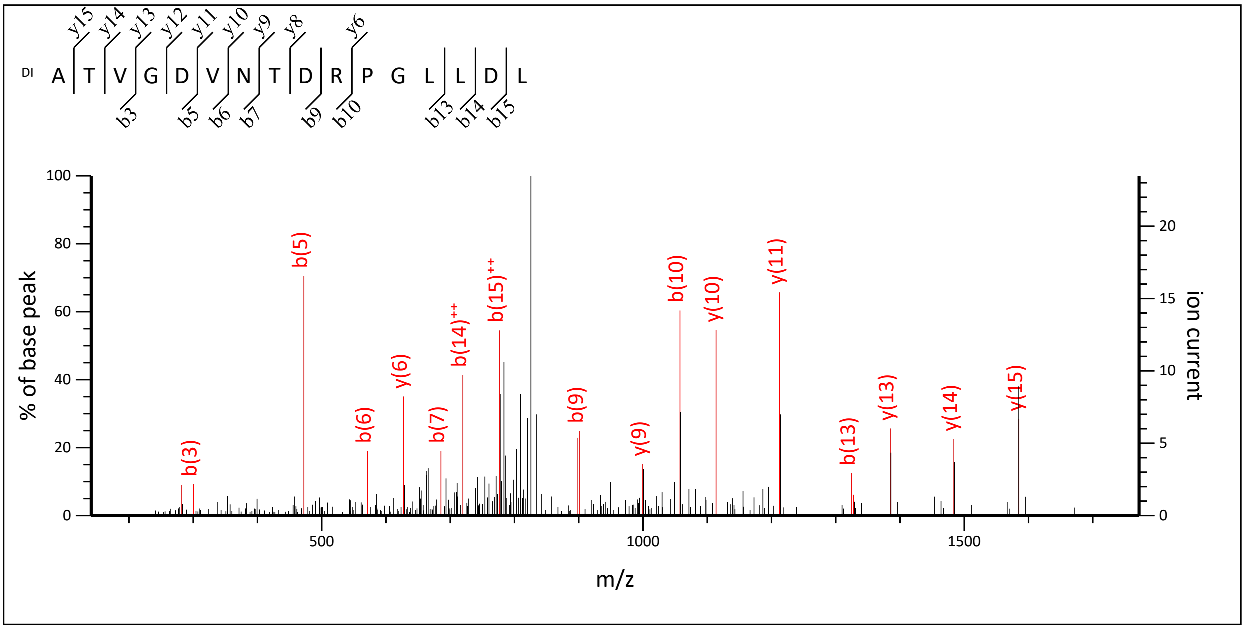

141.23 to 1772.07

Label all possible matches ○ Label matches used for scoring ◉

Monoisotopic mass of neutral peptide Mr(calc): 1682.8839
Variable modifications:
N-term : Dimethyl (N-term)
Ions Score: 65 **Expect:** 0.01
Matches : 19/152 fragment ions using 29 most intense peaks (help)

#	b	b++	b*	b*++	b0	b0++	Seq.	y	y++	y*	y*++	y0	y0++	#
1	100.0757	50.5415					A							16
2	201.1234	101.0653			183.1128	92.0600	T	1584.8228	792.9150	1567.7962	784.4018	1566.8122	783.9097	15
3	300.1918	150.5995			282.1812	141.5942	V	1483.7751	742.3912	1466.7485	733.8779	1465.7645	733.3859	14
4	357.2132	179.1103			339.2027	170.1050	G	1384.7067	692.8570	1367.6801	684.3437	1366.6961	683.8517	13
5	472.2402	236.6237			454.2296	227.6185	D	1327.6852	664.3462	1310.6587	655.8330	1309.6747	655.3410	12
6	571.3086	286.1579			553.2980	277.1527	V	1212.6583	606.8328	1195.6317	598.3195	1194.6477	597.8275	11
7	685.3515	343.1794	668.3250	334.6661	667.3410	334.1741	N	1113.5899	557.2986	1096.5633	548.7853	1095.5793	548.2933	10
8	786.3992	393.7032	769.3727	385.1900	768.3886	384.6980	T	999.5469	500.2771	982.5204	491.7638	981.5364	491.2718	9
9	901.4262	451.2167	884.3996	442.7034	883.4156	442.2114	D	898.4993	449.7533	881.4727	441.2400	880.4887	440.7480	8
10	1057.5273	529.2673	1040.5007	520.7540	1039.5167	520.2620	R	783.4723	392.2398	766.4458	383.7265	765.4617	383.2345	7
11	1154.5800	577.7937	1137.5535	569.2804	1136.5695	568.7884	P	627.3712	314.1892			609.3606	305.1840	6
12	1211.6015	606.3044	1194.5749	597.7911	1193.5909	597.2991	G	530.3184	265.6629			512.3079	256.6576	5
13	1324.6856	662.8464	1307.6590	654.3331	1306.6750	653.8411	L	473.2970	237.1521			455.2864	228.1468	4
14	1437.7696	719.3884	1420.7431	710.8752	1419.7591	710.3832	L	360.2129	180.6101			342.2023	171.6048	3
15	1552.7966	776.9019	1535.7700	768.3886	1534.7860	767.8966	D	247.1288	124.0681			229.1183	115.0628	2
16							L	132.1019	66.5546					1

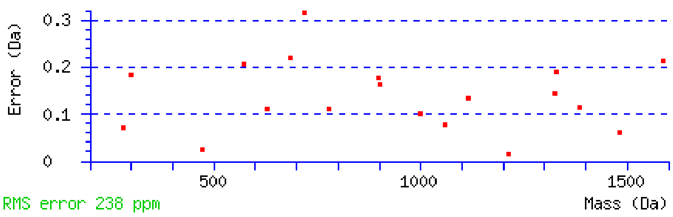

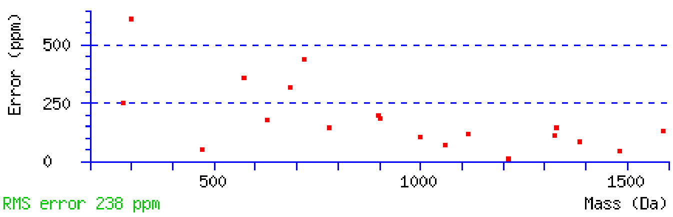

NCBI **BLAST** search of ATVGDVNTDRPGLLDL
(Parameters: blastp, nr protein database, expect=20000, no filter, PAM30)
Other BLAST web gateways

All matches to this query

Score	Mr(calc)	Delta	Sequence
64.9	1682.8839	0.0138	ATVGDVNTDRPGLLDL
18.8	1682.9931	-0.0954	ATVPETGRKDTVVKV
14.8	1682.9310	-0.0333	KVTAMIPLGHMGDPE
12.9	1682.9203	-0.0226	DKNGKLEDEVLPSR
12.2	1682.9138	-0.0161	ATVVAAVDMARVREPA
10.6	1682.6485	0.2492	CGCGNRGMQGNLDTCC
10.6	1682.8927	0.0050	ATVTQMHFLPGQGRL
10.4	1682.8566	0.0411	PLGGDHFAGGGDLASAPL
10.3	1682.8841	0.0135	SGTTTSGGGGRTRREAP
10.1	1682.9167	-0.0190	LAMERDGSSGGVIRLA

Mascot: http://www.matrixscience.com/

ATVGDVNTDRPGLLDL

[redacted] Mascot Search Results

Peptide View

MS/MS Fragmentation of **GDVNTDRPGLLDL**
Found in **ACBP_MOUSE** in **SwissProt**, Acyl-CoA-binding protein OS=Mus musculus GN=Dbi PE=1 SV=2

Match to Query 4471: 1411.736668 from(706.875610,2+) intensity(36356.2890) scans(8478) rawscans(sn8478) rtinseconds(3197.2789) index(6025)
Title: 6026: Scan 8478 (rt=3197.28) [C:\Users\Administrador\Desktop\AMOSTRAS NO ORBITRAP LEANDRO RODAR NOVAMENTE\HIPOCAMPO\hipocampo_01.raw]
Data file hipocampo_01.temp.mgf

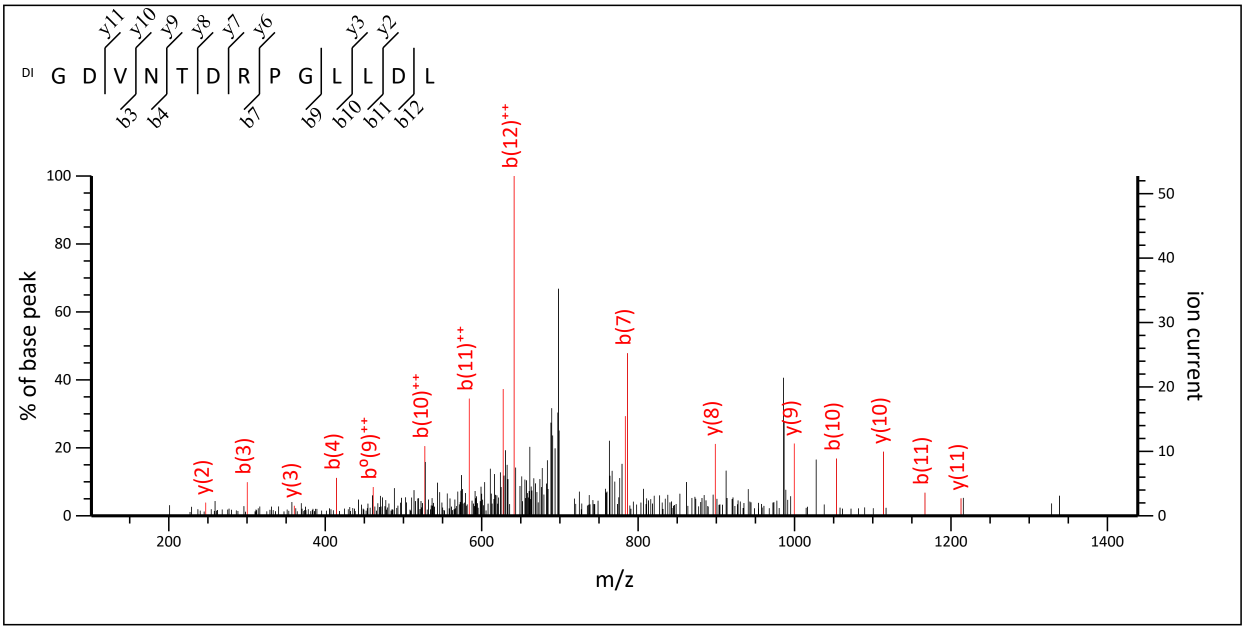

101.07 to 1438.8

Label all possible matches ○ Label matches used for scoring ◉

Monoisotopic mass of neutral peptide Mr(calc): 1411.7307
Variable modifications:
N-term : Dimethyl (N-term)
Ions Score: 47 **Expect:** 0.67
Matches : 17/122 fragment ions using 34 most intense peaks (help)

#	b	b^{++}	b*	b^{*++}	b^0	b^{0++}	Seq.	y	y^{++}	y*	y^{*++}	y^0	y^{0++}	#
1	86.0600	43.5337					G							13
2	201.0870	101.0471			183.0764	92.0418	D	1327.6852	664.3462	1310.6587	655.8330	1309.6747	655.3410	12
3	**300.1554**	150.5813			282.1448	141.5761	V	***1212.6583***	606.8328	1195.6317	598.3195	1194.6477	597.8275	11
4	**414.1983**	207.6028	397.1718	199.0895	396.1878	198.5975	N	***1113.5899***	557.2986	1096.5633	548.7853	1095.5793	548.2933	10
5	515.2460	258.1266	498.2195	249.6134	497.2354	249.1214	T	***999.5469***	500.2771	982.5204	491.7638	981.5364	491.2718	9
6	630.2729	315.6401	613.2464	307.1268	612.2624	306.6348	D	***898.4993***	449.7533	881.4727	441.2400	880.4887	440.7480	8
7	**786.3741**	393.6907	769.3475	385.1774	768.3635	384.6854	R	***783.4723***	392.2398	766.4458	383.7265	765.4618	383.2345	7
8	883.4268	442.2170	866.4003	433.7038	865.4163	433.2118	P	***627.3712***	314.1892			609.3606	305.1840	6
9	940.4483	470.7278	923.4217	462.2145	922.4377	461.7225	G	530.3184	265.6629			512.3079	256.6576	5
10	**1053.5323**	**527.2698**	1036.5058	518.7565	1035.5218	518.2645	L	473.2970	237.1521			455.2864	228.1468	4
11	**1166.6164**	**583.8118**	1149.5899	575.2986	1148.6058	574.8066	L	***360.2129***	180.6101			342.2023	171.6048	3
12	1281.6434	**641.3253**	1264.6168	632.8120	1263.6328	632.3200	D	***247.1288***	124.0681			229.1183	115.0628	2
13							L	132.1019	66.5546					1

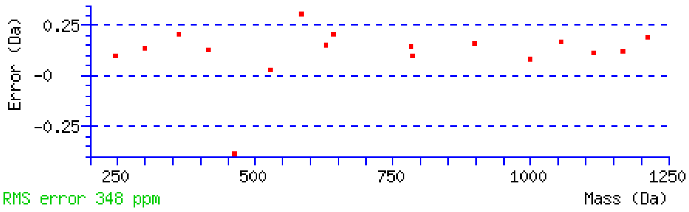

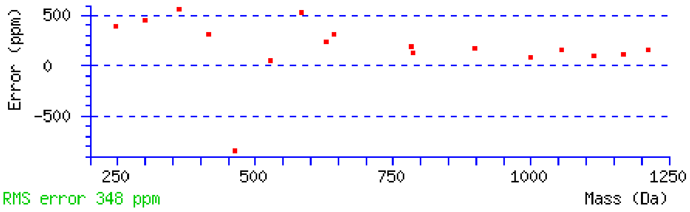

NCBI **BLAST** search of GDVNTDRPGLLDL
(Parameters: blastp, nr protein database, expect=20000, no filter, PAM30)
Other BLAST web gateways

All matches to this query

Score	Mr(calc)	Delta	Sequence
46.6	1411.7307	0.0060	GDVNTDRPGLLDL
21.5	1411.7895	-0.0529	RDVAGGLRSLAQAA
19.7	1411.8203	-0.0836	FPYKNLPIEDL
19.7	1411.8333	-0.0966	ICRKDLLARNL
19.7	1411.8973	-0.1606	KREADLVQLNL
17.9	1411.7537	-0.0170	SFFIGDSGIPLEV
16.2	1411.8224	-0.0857	SLASLPALASQPAL
15.9	1411.7307	0.0059	GTGLGLGGGSPGASTPV
15.8	1411.5761	0.1605	CPAGFHGVHCENN
15.5	1411.9112	-0.1745	LDEKLLNIIDL

Mascot: http://www.matrixscience.com/

[redacted] Mascot Search Results

Peptide View

MS/MS Fragmentation of **KQATVGDVNTDRPGLLDL**
Found in **ACBP_MOUSE** in **SwissProt**, Acyl-CoA-binding protein OS=Mus musculus GN=Dbi PE=1 SV=2

Match to Query 9016: 1975.121802 from(659.381210,3+) intensity(76778.1090) scans(11111) rawscans(sn11111) rtinseconds(3418.9909) index(6401)
Title: 6402: Scan 11111 (rt=3418.99) [C:\Users\Administrador\Desktop\AMOSTRAS NO ORBITRAP LEANDRO RODAR NOVAMENTE\HIPOCAMPO\hipocampo_04.raw]
Data file hipocampo_04.temp.mgf

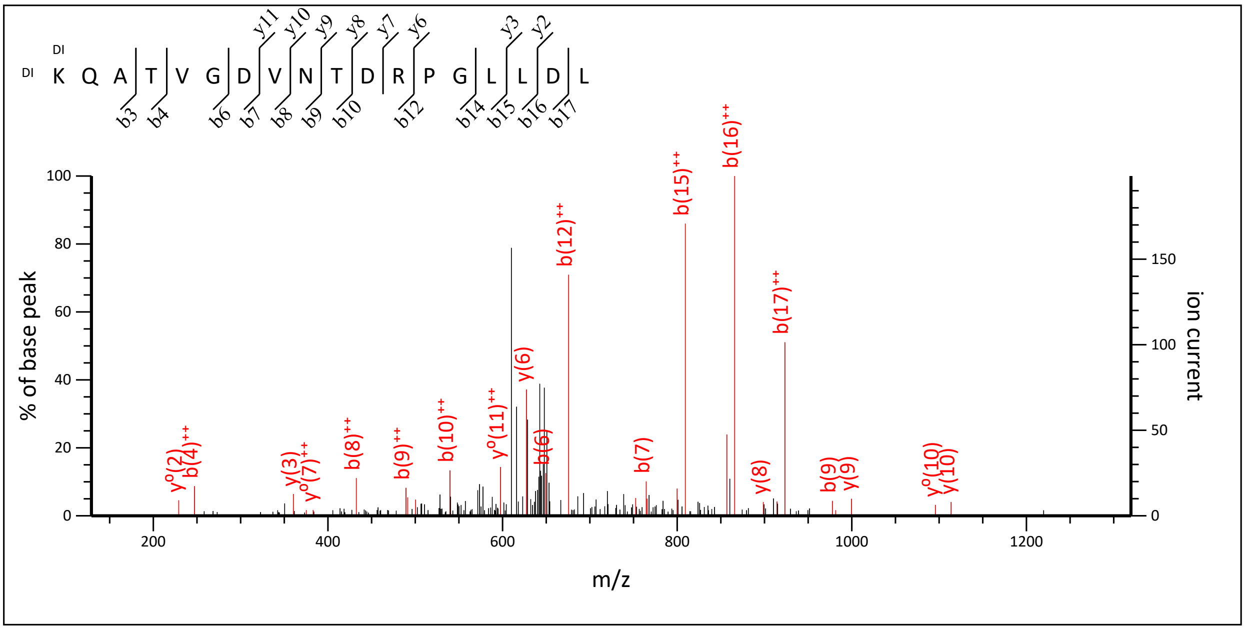

129.1 to 1319.73

Label all possible matches ○ Label matches used for scoring ◉

Monoisotopic mass of neutral peptide Mr(calc): 1975.1190
Variable modifications:
N-term : Dimethyl:2H(4) (N-term)
K1 : Dimethyl:2H(4) (K)
Ions Score: 57 **Expect:** 0.059
Matches : 36/184 fragment ions using 58 most intense peaks (help)

#	b	b^{++}	b*	b^{*++}	b^0	b^{0++}	Seq.	y	y^{++}	y*	y^{*++}	y^0	y^{0++}	#
1	193.2151	97.1112	176.1885	88.5979			K							18
2	321.2736	161.1405	304.2471	152.6272			Q	1783.9185	892.4629	1766.8919	883.9496	1765.9079	883.4576	17
3	392.3107	196.6590	375.2842	188.1457			A	1655.8599	828.4336	1638.8333	819.9203	1637.8493	819.4283	16
4	493.3584	247.1829	476.3319	238.6696	475.3479	238.1776	T	1584.8228	792.9150	1567.7962	784.4018	1566.8122	783.9097	15
5	592.4268	296.7171	575.4003	288.2038	574.4163	287.7118	V	1483.7751	742.3912	1466.7485	733.8779	1465.7645	733.3859	14
6	649.4483	325.2278	632.4218	316.7145	631.4377	316.2225	G	1384.7067	692.8570	1367.6801	684.3437	1366.6961	683.8517	13
7	764.4752	382.7413	747.4487	374.2280	746.4647	373.7360	D	1327.6852	664.3462	1310.6587	655.8330	1309.6747	655.3410	12
8	863.5437	432.2755	846.5171	423.7622	845.5331	423.2702	V	1212.6583	606.8328	1195.6317	598.3195	1194.6477	597.8275	11
9	977.5866	489.2969	960.5600	480.7837	959.5760	480.2916	N	1113.5899	557.2986	1096.5633	548.7853	1095.5793	548.2933	10
10	1078.6343	539.8208	1061.6077	531.3075	1060.6237	530.8155	T	999.5469	500.2771	982.5204	491.7638	981.5364	491.2718	9
11	1193.6612	597.3342	1176.6347	588.8210	1175.6506	588.3290	D	898.4993	449.7533	881.4727	441.2400	880.4887	440.7480	8
12	1349.7623	675.3848	1332.7358	666.8715	1331.7518	666.3795	R	783.4723	392.2398	766.4458	383.7265	765.4617	383.2345	7
13	1446.8151	723.9112	1429.7885	715.3979	1428.8045	714.9059	P	627.3712	314.1892			609.3606	305.1840	6
14	1503.8365	752.4219	1486.8100	743.9086	1485.8260	743.4166	G	530.3184	265.6629			512.3079	256.6576	5
15	1616.9206	808.9639	1599.8941	800.4507	1598.9100	799.9587	L	473.2970	237.1521			455.2864	228.1468	4
16	1730.0047	865.5060	1712.9781	856.9927	1711.9941	856.5007	L	360.2129	180.6101			342.2023	171.6048	3
17	1845.0316	923.0194	1828.0051	914.5062	1827.0211	914.0142	D	247.1288	124.0681			229.1183	115.0628	2
18							L	132.1019	66.5546					1

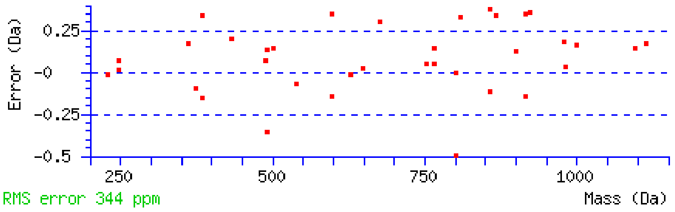

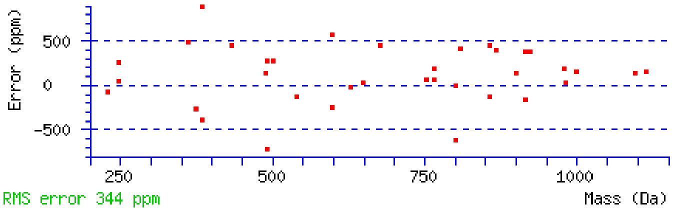

NCBI **BLAST** search of KQATVGDVNTDRPGLLDL
(Parameters: blastp, nr protein database, expect=20000, no filter, PAM30)
Other BLAST web gateways

All matches to this query

Score	Mr(calc)	Delta	Sequence
57.4	1975.1190	0.0028	KQATVGDVNTDRPGLLDL
35.0	1974.9839	0.1379	RFQPTYPYVQHEIDL
31.1	1974.9750	0.1468	YTMTNGGSINSSTHLLDL
23.7	1975.1367	-0.0149	FAKVFVDAREIKQNAR
22.0	1974.8663	0.2555	CDLISDMGNLVHATNSEE
22.0	1975.3664	-0.2446	TLAISAKGIADKLLLLDL
21.2	1975.1106	0.0112	CAQQQLLTRVLHFILD
21.1	1975.1171	0.0047	PRDIFRPLDILSDLDL
21.1	1975.0900	0.0319	TAIHEVMEQQTISIAKA
20.9	1975.2070	-0.0852	PLTAFANLRKLERLDI

Mascot: http://www.matrixscience.com/

[redacted] Mascot Search Results

Peptide View

MS/MS Fragmentation of **KQATVGDVNTDRPGLLDL**
Found in **ACBP_MOUSE** in **SwissProt**, Acyl-CoA-binding protein OS=Mus musculus GN=Dbi PE=1 SV=2

Match to Query 7527: 1967.069428 from(984.541990,2+) intensity(29598.1250) scans(7731) rawscans(sn7731) rtinseconds(2935.691) index(5420)
Title: 5421: Scan 7731 (rt=2935.69) [C:\Users\Administrador\Desktop\AMOSTRAS NO ORBITRAP LEANDRO RODAR NOVAMENTE\HIPOCAMPO\hipocampo_01.raw]
Data file hipocampo_01.temp.mgf

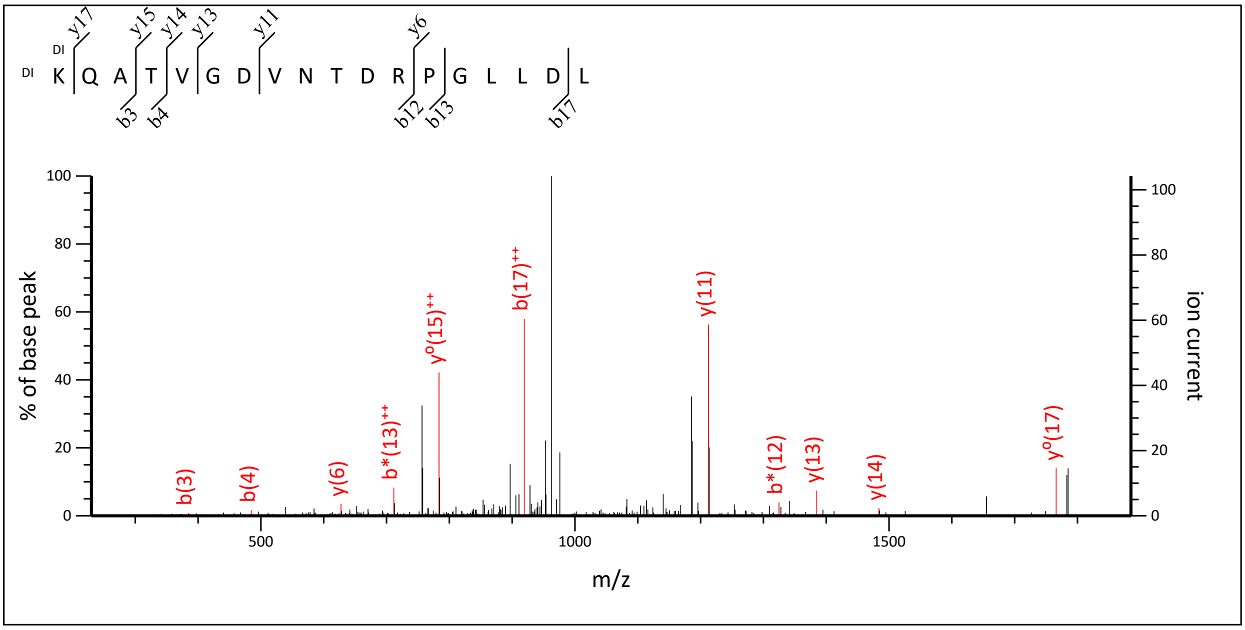

230.2 to 1885.14

Label all possible matches ○ Label matches used for scoring ◉

Monoisotopic mass of neutral peptide Mr(calc): 1967.0688
Variable modifications:
N-term : Dimethyl (N-term)
K1 : Dimethyl (K)
Ions Score: 35 **Expect:** 10
Matches : 13/184 fragment ions using 14 most intense peaks (help)

#	b	b^{++}	b*	b^{*++}	b^0	b^{0++}	Seq.	y	y^{++}	y*	y^{*++}	y^0	y^{0++}	#
1	185.1648	93.0861	168.1383	84.5728			K							18
2	313.2234	157.1153	296.1969	148.6021			Q	1783.9185	892.4629	1766.8919	883.9496	1765.9079	883.4576	17
3	**384.2605**	192.6339	367.2340	184.1206			A	1655.8599	828.4336	1638.8333	819.9203	1637.8493	819.4283	16
4	**485.3082**	243.1577	468.2817	234.6445	467.2976	234.1525	T	1584.8228	792.9150	1567.7962	784.4018	1566.8122	783.9097	15
5	584.3766	292.6920	567.3501	284.1787	566.3661	283.6867	V	***1483.7751***	742.3912	1466.7485	733.8779	1465.7645	733.3859	14
6	641.3981	321.2027	624.3715	312.6894	623.3875	312.1974	G	***1384.7067***	692.8570	1367.6801	684.3437	1366.6961	683.8517	13
7	756.4250	378.7162	739.3985	370.2029	738.4145	369.7109	D	1327.6852	664.3462	1310.6587	655.8330	1309.6747	655.3410	12
8	855.4934	428.2504	838.4669	419.7371	837.4829	419.2451	V	***1212.6583***	606.8328	1195.6317	598.3195	1194.6477	597.8275	11
9	969.5364	**485.2718**	952.5098	476.7585	951.5258	476.2665	N	1113.5899	557.2986	1096.5633	548.7853	1095.5793	548.2933	10
10	1070.5841	535.7957	1053.5575	527.2824	1052.5735	526.7904	T	999.5469	500.2771	982.5204	491.7638	981.5364	491.2718	9
11	1185.6110	593.3091	1168.5844	584.7959	1167.6004	584.3039	D	898.4993	449.7533	881.4727	441.2400	880.4887	440.7480	8
12	1341.7121	671.3597	1324.6856	662.8464	1323.7015	662.3544	R	***783.4723***	392.2398	766.4458	383.7265	765.4617	383.2345	7
13	1438.7649	719.8861	1421.7383	711.3728	1420.7543	710.8808	P	***627.3712***	314.1892			609.3606	305.1840	6
14	1495.7863	748.3968	1478.7598	739.8835	1477.7758	739.3915	G	530.3184	265.6629			512.3079	256.6576	5
15	1608.8704	804.9388	1591.8438	796.4256	1590.8598	795.9336	L	473.2970	237.1521			455.2864	228.1468	4
16	1721.9545	861.4809	1704.9279	852.9676	1703.9439	852.4756	L	360.2129	180.6101			342.2023	171.6048	3
17	1836.9814	**918.9943**	1819.9549	910.4811	1818.9708	909.9891	D	247.1288	124.0681			229.1183	115.0628	2
18							L	132.1019	66.5546					1

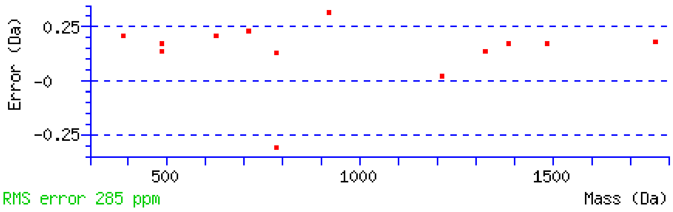

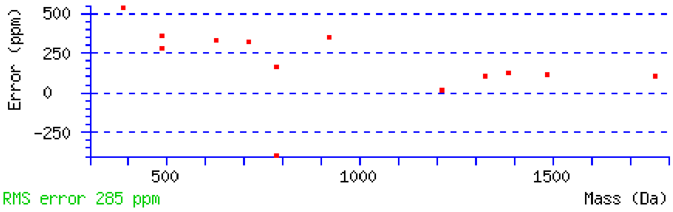

NCBI **BLAST** search of KQATVGDVNTDRPGLLDL
(Parameters: blastp, nr protein database, expect=20000, no filter, PAM30)
Other BLAST web gateways

All matches to this query

Score	Mr(calc)	Delta	Sequence
34.9	1967.0688	0.0007	KQATVGDVNTDRPGLLDL
19.9	1966.9133	0.1561	RGGSGGSGAGPGTTGAAGEFAFA
13.0	1967.0826	-0.0132	EFADSLGVPFLETSAKNA
13.0	1967.2403	-0.1709	EVVLMKVLGGCKLLLRL
12.7	1967.1237	-0.0543	REKLCESLGLRVADLPL
12.0	1967.0404	0.0291	EIIAELRYDLPALYNF
11.8	1967.2281	-0.1587	SEAKILFLLQLLTDHI
11.4	1967.0517	0.0178	TIISIASNVAGHPLVWDF
11.4	1966.9021	0.1674	ISNNGATGTWLYRNESD
11.2	1967.1826	-0.1131	LKGETRLNCIRIARKA

Mascot: http://www.matrixscience.com/

[redacted] Mascot Search Results

Peptide View

MS/MS Fragmentation of **SHFKQATVGDVNTDRPGLLDL**
Found in **ACBP_MOUSE** in **SwissProt**, Acyl-CoA-binding protein OS=Mus musculus GN=Dbi PE=1 SV=2

Match to Query 9570: 2346.289062 from(783.103630,3+) intensity(14550.9800) scans(7669) rawscans(sn7669) rtinseconds(2938.3815) index(5403)
Title: 5404: Scan 7669 (rt=2938.38) [C:\Users\Administrador\Desktop\AMOSTRAS NO ORBITRAP LEANDRO RODAR NOVAMENTE\CORTEX\cortex_01.raw]
Data file cortex_01.temp.mgf

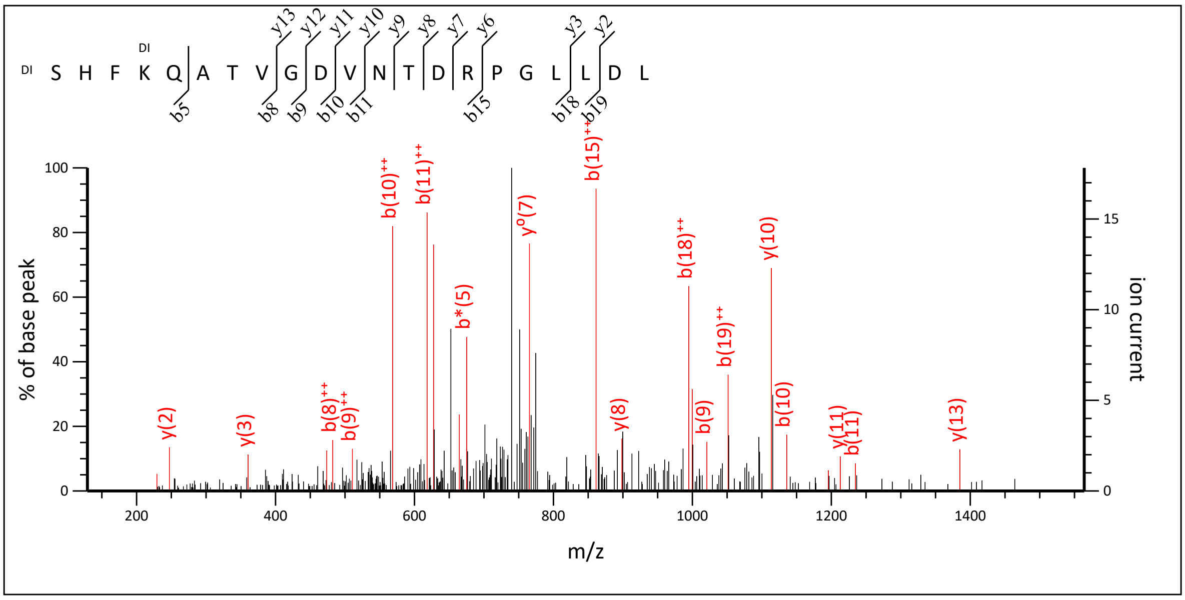

129.2 to 1564.02

Label all possible matches ○ Label matches used for scoring ◉

```
Monoisotopic mass of neutral peptide Mr(calc): 2346.2784
Variable modifications:
N-term : Dimethyl:2H(4) (N-term)
K4     : Dimethyl:2H(4) (K)
Ions Score: 52  Expect: 0.2
Matches : 27/220 fragment ions using 37 most intense peaks   (help)
```

#	b	b^{++}	b*	b^{*++}	b^0	b^{0++}	Seq.	y	y^{++}	y*	y^{*++}	y^0	y^{0++}	#
1	120.0957	60.5515			102.0851	51.5462	S							21
2	257.1546	129.0809			239.1441	120.0757	H	2228.1972	1114.6022	2211.1706	1106.0889	2210.1866	1105.5969	20
3	404.2230	202.6152			386.2125	193.6099	F	2091.1383	1046.0728	2074.1117	1037.5595	2073.1277	1037.0675	19
4	564.3744	282.6908	547.3479	274.1776	546.3638	273.6856	K	1944.0698	972.5386	1927.0433	964.0253	1926.0593	963.5333	18
5	692.4330	346.7201	675.4064	338.2069	674.4224	337.7148	Q	1783.9185	892.4629	1766.8919	883.9496	1765.9079	883.4576	17
6	763.4701	382.2387	746.4435	373.7254	745.4595	373.2334	A	1655.8599	828.4336	1638.8333	819.9203	1637.8493	819.4283	16
7	864.5178	432.7625	847.4912	424.2493	846.5072	423.7572	T	1584.8228	792.9150	1567.7962	784.4018	1566.8122	783.9097	15
8	963.5862	***482.2967***	946.5596	473.7835	945.5756	473.2915	V	1483.7751	742.3912	1466.7486	733.8779	1465.7645	733.3859	14
9	**1020.6077**	***510.8075***	1003.5811	502.2942	1002.5971	501.8022	G	***1384.7067***	692.8570	1367.6801	684.3437	1366.6961	683.8517	13
10	**1135.6346**	***568.3209***	1118.6081	559.8077	1117.6240	559.3157	D	1327.6852	664.3462	1310.6587	655.8330	1309.6747	655.3410	12
11	**1234.7030**	***617.8551***	1217.6765	609.3419	1216.6924	608.8499	V	***1212.6583***	606.8328	1195.6317	598.3195	1194.6477	597.8275	11
12	1348.7459	***674.8766***	1331.7194	666.3633	1330.7354	665.8713	N	***1113.5899***	557.2986	1096.5633	548.7853	1095.5793	548.2933	10
13	1449.7936	725.4004	1432.7671	716.8872	1431.7831	716.3952	T	***999.5469***	500.2771	982.5204	491.7638	981.5364	491.2718	9
14	1564.8206	782.9139	1547.7940	774.4006	1546.8100	773.9086	D	***898.4993***	449.7533	881.4727	441.2400	880.4887	440.7480	8
15	1720.9217	***860.9645***	1703.8951	852.4512	1702.9111	851.9592	R	783.4723	392.2398	766.4458	383.7265	765.4617	383.2345	7
16	1817.9744	909.4909	1800.9479	900.9776	1799.9639	900.4856	P	***627.3712***	314.1892			609.3606	305.1840	6
17	1874.9959	938.0016	1857.9694	929.4883	1856.9853	928.9963	G	530.3184	265.6629			512.3079	256.6576	5
18	1988.0800	***994.5436***	1971.0534	986.0303	1970.0694	985.5383	L	***473.2970***	237.1521			455.2864	228.1468	4
19	2101.1640	***1051.0857***	2084.1375	1042.5724	2083.1535	1042.0804	L	***360.2129***	180.6101			342.2023	171.6048	3
20	2216.1910	1108.5991	2199.1644	1100.0858	2198.1804	1099.5938	D	***247.1288***	124.0681			229.1183	115.0628	2
21							L	132.1019	66.5546					1

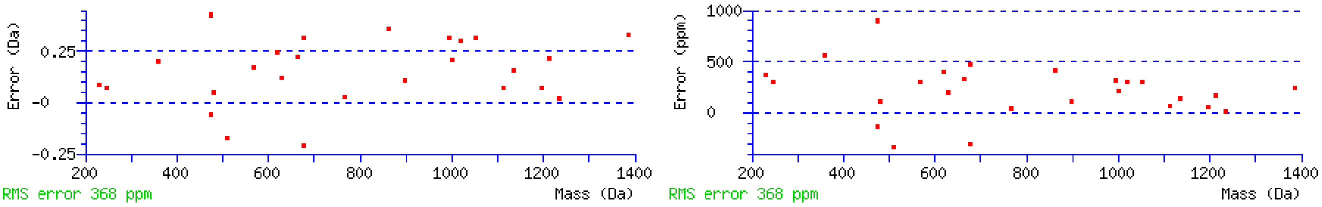

NCBI **BLAST** search of SHFKQATVGDVNTDRPGLLDL
(Parameters: blastp, nr protein database, expect=20000, no filter, PAM30)
Other BLAST web gateways

All matches to this query

Score	Mr(calc)	Delta	Sequence
52.4	2346.2784	0.0107	SHFKQATVGDVNTDRPGLLDL
19.7	2346.2040	0.0851	MVVQVVIGEQRGEGVFMAARC
18.2	2346.1301	0.1590	FDVLSSSEGGVATGTFSLEAMK
17.2	2346.2563	0.0328	LLRNMVGAGEVDEDLEVETK
16.7	2346.3370	-0.0480	ATKKFRRSVLLSVDECEEV
16.4	2346.5192	-0.2302	LLPKVLVQATVFARMAPEQK
16.3	2346.2907	-0.0016	GTIHIQVTSEVLEQRIVQQV
16.2	2346.1604	0.1286	YQVHDRNATVDKLEDFINN
15.9	2346.0798	0.2093	CGNFDSVPGDDVNEHIASSKIS
15.1	2346.2919	-0.0028	EPTPGLSREVIRFLLEQTVM

Mascot: http://www.matrixscience.com/

[redacted] Mascot Search Results

Peptide View

MS/MS Fragmentation of **TVGDVNTDRPGLLDL**
Found in **ACBP_MOUSE** in **SwissProt**, Acyl-CoA-binding protein OS=Mus musculus GN=Dbi PE=1 SV=2

Match to Query 5594: 1611.851988 from(806.933270,2+) intensity(92331.3520) scans(9395) rawscans(sn9395) rtinseconds(3525.0802) index(6752)
Title: 6753: Scan 9395 (rt=3525.08) [C:\Users\Administrador\Desktop\AMOSTRAS NO ORBITRAP LEANDRO RODAR NOVAMENTE\HIPOCAMPO\hipocampo_01.raw]
Data file hipocampo_01.temp.mgf

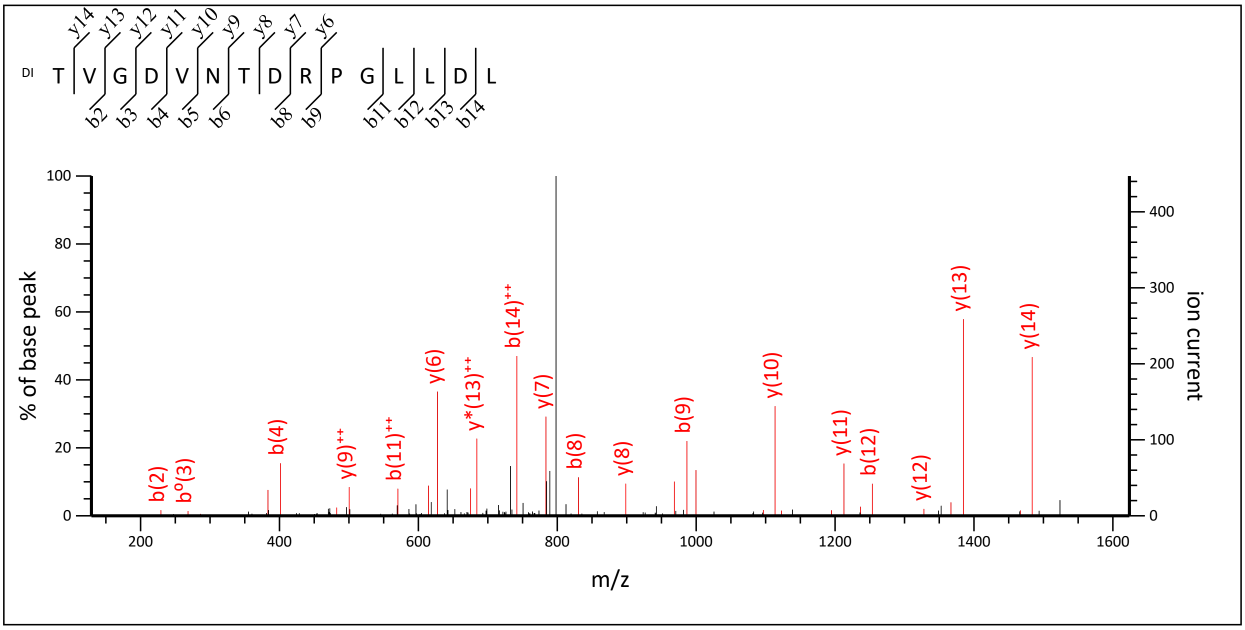

129.21 to 1623.82

Label all possible matches ○ Label matches used for scoring ◉

```
Monoisotopic mass of neutral peptide Mr(calc): 1611.8468
Variable modifications:
N-term : Dimethyl (N-term)
Ions Score: 85  Expect: 0.0001
Matches : 40/144 fragment ions using 39 most intense peaks   (help)
```

#	b	b^{++}	b*	b^{*++}	b^0	b^{0++}	Seq.	y	y^{++}	y*	y^{*++}	y^0	y^{0++}	#
1	130.0863	65.5468			112.0757	56.5415	T							15
2	229.1547	115.0810			211.1441	106.0757	V	1483.7751	742.3912	1466.7485	733.8779	1465.7645	733.3859	14
3	286.1761	143.5917			268.1656	134.5864	G	1384.7067	692.8570	1367.6801	684.3437	1366.6961	683.8517	13
4	401.2031	201.1052			383.1925	192.0999	D	1327.6852	664.3462	1310.6587	655.8330	1309.6747	655.3410	12
5	500.2715	250.6394			482.2609	241.6341	V	1212.6583	606.8328	1195.6317	598.3195	1194.6477	597.8275	11
6	614.3144	307.6608	597.2879	299.1476	596.3039	298.6556	N	1113.5899	557.2986	1096.5633	548.7853	1095.5793	548.2933	10
7	715.3621	358.1847	698.3355	349.6714	697.3515	349.1794	T	999.5469	500.2771	982.5204	491.7638	981.5364	491.2718	9
8	830.3890	415.6982	813.3625	407.1849	812.3785	406.6929	D	898.4993	449.7533	881.4727	441.2400	880.4887	440.7480	8
9	986.4901	493.7487	969.4636	485.2354	968.4796	484.7434	R	783.4723	392.2398	766.4458	383.7265	765.4617	383.2345	7
10	1083.5429	542.2751	1066.5164	533.7618	1065.5323	533.2698	P	627.3712	314.1892			609.3606	305.1840	6
11	1140.5644	570.7858	1123.5378	562.2726	1122.5538	561.7805	G	530.3184	265.6629			512.3079	256.6576	5
12	1253.6484	627.3279	1236.6219	618.8146	1235.6379	618.3226	L	473.2970	237.1521			455.2864	228.1468	4
13	1366.7325	683.8699	1349.7060	675.3566	1348.7219	674.8646	L	360.2129	180.6101			342.2023	171.6048	3
14	1481.7594	741.3834	1464.7329	732.8701	1463.7489	732.3781	D	247.1288	124.0681			229.1183	115.0628	2
15							L	132.1019	66.5546					1

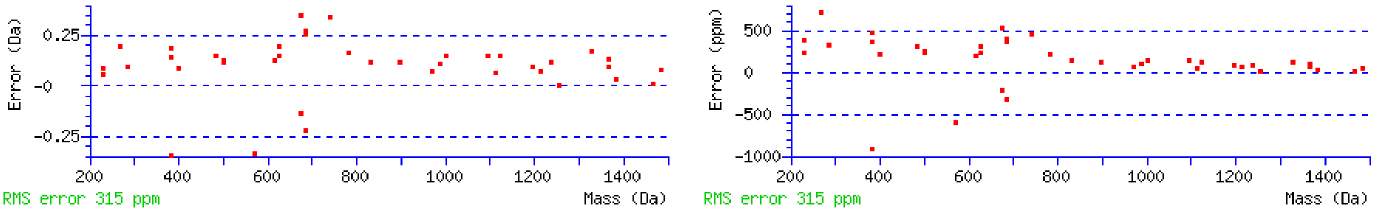

NCBI **BLAST** search of TVGDVNTDRPGLLDL
(Parameters: blastp, nr protein database, expect=20000, no filter, PAM30)
Other BLAST web gateways

All matches to this query

Score	Mr(calc)	Delta	Sequence
84.9	1611.8468	0.0052	TVGDVNTDRPGLLDL
29.9	1611.8719	-0.0200	LSISASKSVPGEVPEG
29.6	1611.7678	0.0842	GEQQPDTRPEDLPG
28.2	1611.8658	-0.0138	PVTPTSQSVPSSPSIP
28.2	1611.9147	-0.0627	RHLTSGNWEVIRI
25.4	1611.7992	0.0528	TLQGQELSTEPDGPL
25.3	1611.9406	-0.0887	KEAVPSVAPSGLSGGGA
25.2	1611.9573	-0.1053	SLRELGVWRSLLR
25.1	1611.9058	-0.0538	VLCGLIWASAALASLP
24.9	1611.7127	0.1393	EQDVQLYCDFPDI

Mascot: http://www.matrixscience.com/

[redacted] Mascot Search Results

Peptide View

MS/MS Fragmentation of **VEKVDELKKKYGI**
Found in **ACBP_MOUSE** in **SwissProt**, Acyl-CoA-binding protein OS=Mus musculus GN=Dbi PE=1 SV=2

Match to Query 6057: 1688.053872 from(563.691900,3+) intensity(158413.2800) scans(5190) rawscans(sn5190) rtinseconds(2058.7076) index(3181)
Title: 3182: Scan 5190 (rt=2058.71) [C:\Users\Administrador\Desktop\AMOSTRAS NO ORBITRAP LEANDRO RODAR NOVAMENTE\HIPOCAMPO\hipocampo_01.raw]
Data file hipocampo_01.temp.mgf

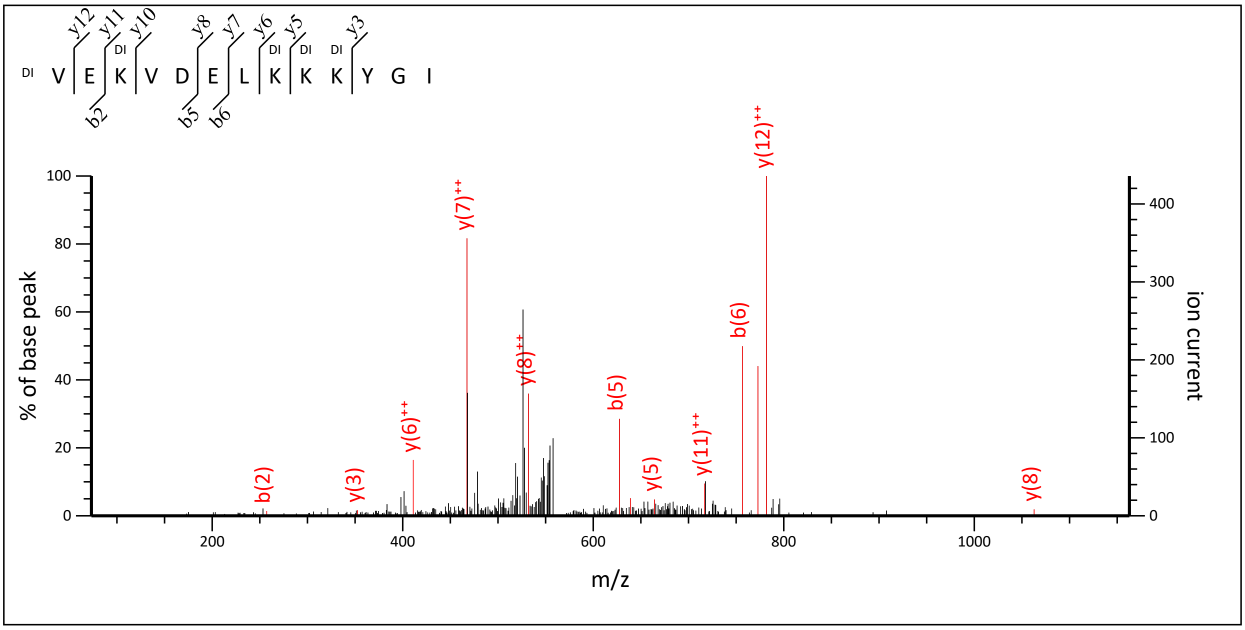

73.23 to 1162.83

Label all possible matches ○ Label matches used for scoring ◉

```
Monoisotopic mass of neutral peptide Mr(calc): 1688.0488
Variable modifications:
N-term : Dimethyl (N-term)
K3     : Dimethyl (K)
K8     : Dimethyl (K)
K9     : Dimethyl (K)
K10    : Dimethyl (K)
Ions Score: 23  Expect: 1.4e+002
Matches : 14/118 fragment ions using 31 most intense peaks   (help)
```

#	b	b^{++}	b*	b^{*++}	b^0	b^{0++}	Seq.	y	y^{++}	y*	y^{*++}	y^0	y^{0++}	#
1	128.1070	64.5571					V							13
2	**257.1496**	129.0784			239.1390	120.0731	E	1561.9564	***781.4818***	1544.9298	772.9685	1543.9458	772.4765	12
3	413.2758	207.1416	396.2493	198.6283	395.2653	198.1363	K	1432.9138	***716.9605***	1415.8872	708.4472	1414.9032	707.9552	11
4	512.3443	256.6758	495.3177	248.1625	494.3337	247.6705	V	1276.7875	***638.8974***	1259.7610	630.3841	1258.7769	629.8921	10
5	**627.3712**	314.1892	610.3447	305.6760	609.3606	305.1840	D	1177.7191	589.3632	1160.6925	580.8499	1159.7085	580.3579	9
6	**756.4138**	378.7105	739.3872	370.1973	738.4032	369.7053	E	***1062.6921***	***531.8497***	1045.6656	523.3364	1044.6816	522.8444	8
7	869.4979	435.2526	852.4713	426.7393	851.4873	426.2473	L	933.6496	***467.3284***	916.6230	458.8151			7
8	1025.6241	513.3157	1008.5976	504.8024	1007.6136	504.3104	K	820.5655	***410.7864***	803.5389	402.2731			6
9	1181.7504	591.3788	1164.7238	582.8656	1163.7398	582.3735	K	***664.4392***	332.7233	647.4127	324.2100			5
10	1337.8766	669.4420	1320.8501	660.9287	1319.8661	660.4367	K	508.3130	254.6601	491.2864	246.1468			4
11	1500.9400	750.9736	1483.9134	742.4604	1482.9294	741.9683	Y	***352.1867***	176.5970					3
12	1557.9614	779.4844	1540.9349	770.9711	1539.9509	770.4791	G	189.1234	95.0653					2
13							I	132.1019	66.5546					1

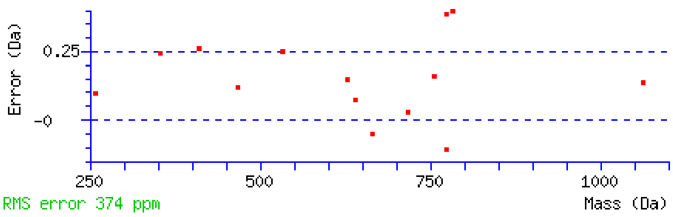

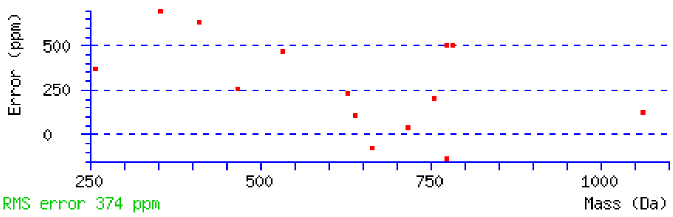

NCBI **BLAST** search of VEKVDELKKKYGI
(Parameters: blastp, nr protein database, expect=20000, no filter, PAM30)
Other BLAST web gateways

All matches to this query

Score	Mr(calc)	Delta	Sequence
23.4	1688.0488	0.0051	VEKVDELKKKYGI
18.9	1687.8927	0.1612	GERLTQCREVLLTT
17.2	1687.8570	0.1969	FVFPRESETDHVK
16.9	1687.8933	0.1605	GSELGLELVFVWNR
16.4	1687.9422	0.1117	TQKSYSSSETLKAY
15.7	1687.9719	0.0820	LSAYEQKLQTVHAS
15.4	1687.8967	0.1572	ALLSGREMVGPTLPGY
15.4	1687.8516	0.2023	KSVLTAGGVSGEELDE
15.1	1688.0310	0.0228	VELKIVGEKACKIF
14.2	1687.7956	0.2582	GELRTHVSRCQVCM

Mascot: http://www.matrixscience.com/

[redacted] Mascot Search Results

Peptide View

MS/MS Fragmentation of **VEKVDELKKKYGI**
Found in **ACBP_MOUSE** in **SwissProt**, Acyl-CoA-binding protein OS=Mus musculus GN=Dbi PE=1 SV=2

Match to Query 6057: 1688.053872 from(563.691900,3+) intensity(158413.2800) scans(5190) rawscans(sn5190) rtinseconds(2058.7076) index(3181)
Title: 3182: Scan 5190 (rt=2058.71) [C:\Users\Administrador\Desktop\AMOSTRAS NO ORBITRAP LEANDRO RODAR NOVAMENTE\HIPOCAMPO\hipocampo_01.raw]
Data file hipocampo_01.temp.mgf

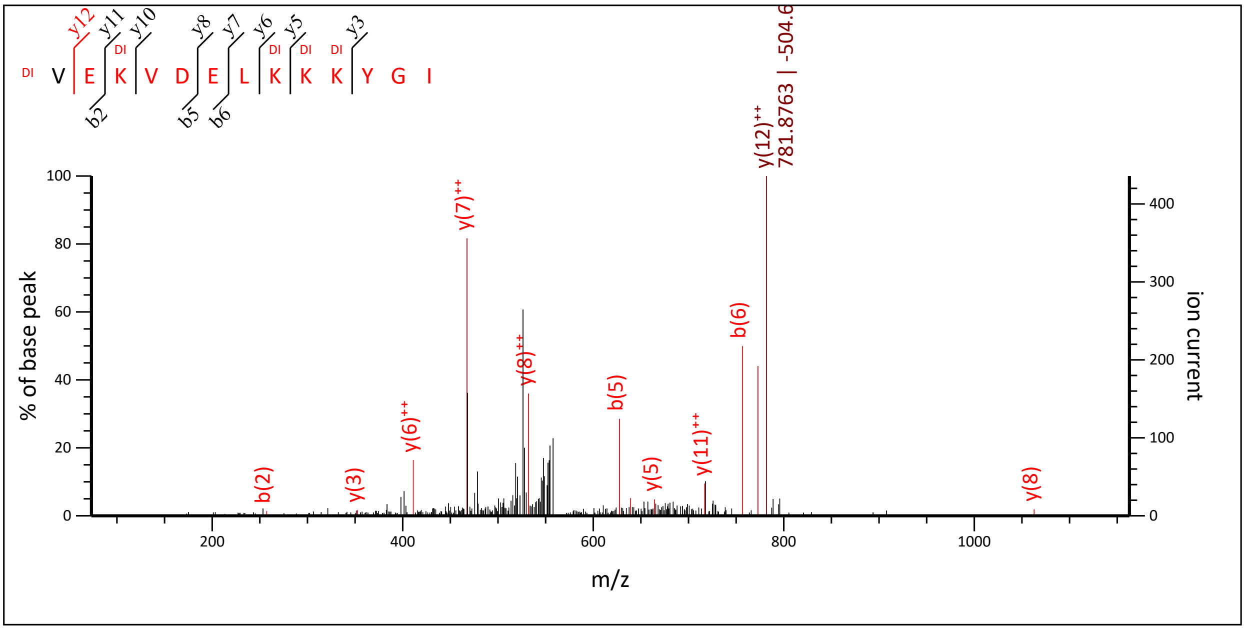

73.23 to 1162.83

Label all possible matches ○ Label matches used for scoring ◉

Monoisotopic mass of neutral peptide Mr(calc): 1688.0488
Variable modifications:
N-term : Dimethyl (N-term)
K3 : Dimethyl (K)
K8 : Dimethyl (K)
K9 : Dimethyl (K)
K10 : Dimethyl (K)
Ions Score: 23 **Expect:** 1.4e+002
Matches : 14/118 fragment ions using 31 most intense peaks (help)

#	b	b^{++}	b*	b^{*++}	b^0	b^{0++}	Seq.	y	y^{++}	y*	y^{*++}	y^0	y^{0++}	#
1	128.1070	64.5571					V							13
2	**257.1496**	129.0784			239.1390	120.0731	E	1561.9564	**781.4818**	1544.9298	772.9685	1543.9458	772.4765	12
3	413.2758	207.1416	396.2493	198.6283	395.2653	198.1363	K	1432.9138	**716.9605**	1415.8872	708.4472	1414.9032	707.9552	11
4	512.3443	256.6758	495.3177	248.1625	494.3337	247.6705	V	1276.7875	**638.8974**	1259.7610	630.3841	1258.7769	629.8921	10
5	**627.3712**	314.1892	610.3447	305.6760	609.3606	305.1840	D	1177.7191	589.3632	1160.6925	580.8499	1159.7085	580.3579	9
6	**756.4138**	378.7105	739.3872	370.1973	738.4032	369.7053	E	**1062.6921**	**531.8497**	1045.6656	523.3364	1044.6816	522.8444	8
7	869.4979	435.2526	852.4713	426.7393	851.4873	426.2473	L	933.6496	**467.3284**	916.6230	458.8151			7
8	1025.6241	513.3157	1008.5976	504.8024	1007.6136	504.3104	K	820.5655	**410.7864**	803.5389	402.2731			6
9	1181.7504	591.3788	1164.7238	582.8656	1163.7398	582.3735	K	**664.4392**	332.7233	647.4127	324.2100			5
10	1337.8766	669.4420	1320.8501	660.9287	1319.8661	660.4367	K	508.3130	254.6601	491.2864	246.1468			4
11	1500.9400	750.9736	1483.9134	742.4604	1482.9294	741.9683	Y	**352.1867**	176.5970					3
12	1557.9614	779.4844	1540.9349	770.9711	1539.9509	770.4791	G	189.1234	95.0653					2
13							I	132.1019	66.5546					1

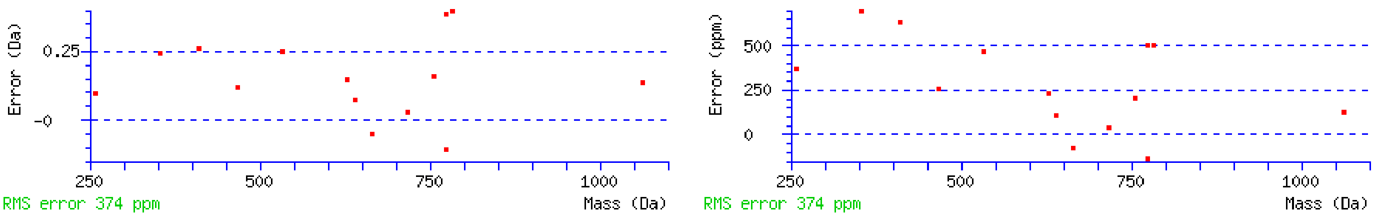

NCBI **BLAST** search of VEKVDELKKKYGI
(Parameters: blastp, nr protein database, expect=20000, no filter, PAM30)
Other BLAST web gateways

All matches to this query

Score	Mr(calc)	Delta	Sequence
23.4	1688.0488	0.0051	VEKVDELKKKYGI
18.9	1687.8927	0.1612	GERLTQCREVLLTT
17.2	1687.8570	0.1969	FVFPRESETDHVK
16.9	1687.8933	0.1605	GSELGLELVFVWNR
16.4	1687.9422	0.1117	TQKSYSSSETLKAY
15.7	1687.9719	0.0820	LSAYEQKLQTVHAS
15.4	1687.8967	0.1572	ALLSGREMVGPTLPGY
15.4	1687.8516	0.2023	KSVLTAGGVSGEELDE
15.1	1688.0310	0.0228	VELKIVGEKACKIF
14.2	1687.7956	0.2582	GELRTHVSRCQVCM

Mascot: http://www.matrixscience.com/

[redacted] Mascot Search Results

Peptide View

MS/MS Fragmentation of **KFDDPKFEVIDKPQS**
Found in **ATP5J_MOUSE** in **SwissProt**, ATP synthase-coupling factor 6, mitochondrial OS=Mus musculus GN=Atp5j PE=1 SV=1

Match to Query 7204: 1904.038388 from(953.026470,2+) intensity(32767.4570) scans(5862) rawscans(sn5862) rtinseconds(2278.4824) index(3776)
Title: 3777: Scan 5862 (rt=2278.48) [C:\Users\Administrador\Desktop\AMOSTRAS NO ORBITRAP LEANDRO RODAR NOVAMENTE\HIPOCAMPO\hipocampo_01.raw]
Data file hipocampo_01.temp.mgf

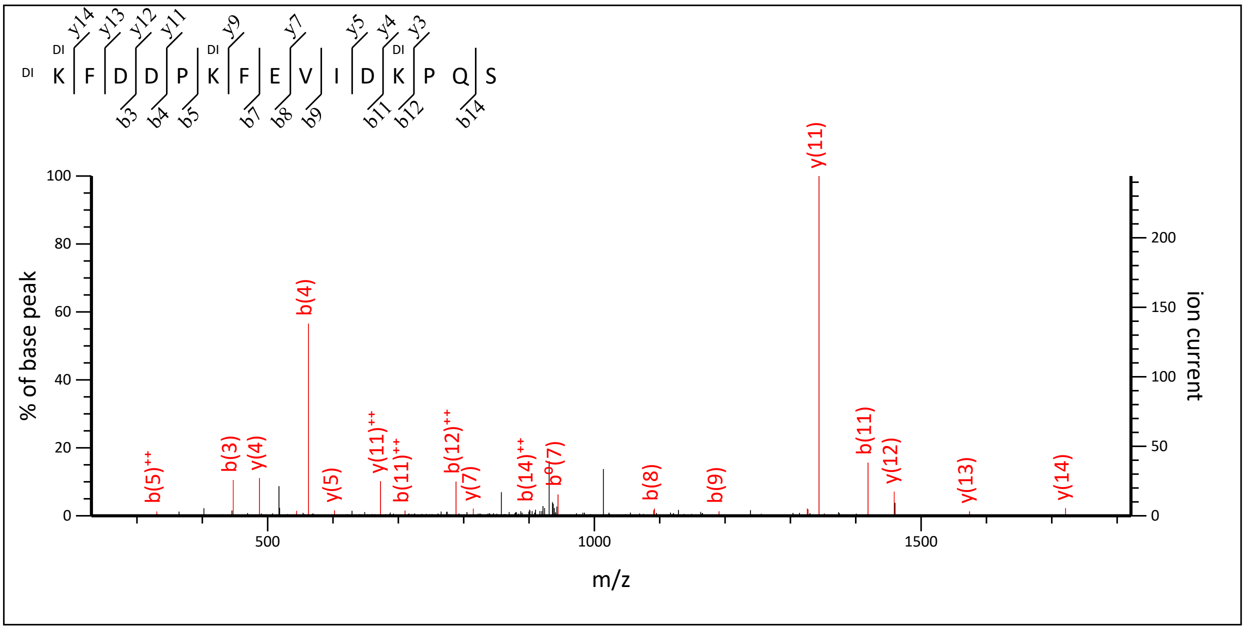

230.31 to 1821.15

Label all possible matches ○ Label matches used for scoring ◉

Monoisotopic mass of neutral peptide Mr(calc): 1904.0295
Variable modifications:
N-term : Dimethyl (N-term)
K1 : Dimethyl (K)
K6 : Dimethyl (K)
K12 : Dimethyl (K)
Ions Score: 45 **Expect:** 1
Matches : 23/162 fragment ions using 48 most intense peaks (help)

#	b	b^{++}	b*	b^{*++}	b^0	b^{0++}	Seq.	y	y^{++}	y*	y^{*++}	y^0	y^{0++}	#
1	185.1648	93.0861	168.1383	84.5728			K							15
2	332.2333	166.6203	315.2067	158.1070			F	1720.8792	860.9433	1703.8527	852.4300	1702.8687	851.9380	14
3	447.2602	224.1337	430.2336	215.6205	429.2496	215.1285	D	1573.8108	787.4090	1556.7843	778.8958	1555.8003	778.4038	13
4	562.2871	281.6472	545.2606	273.1339	544.2766	272.6419	D	1458.7839	729.8956	1441.7573	721.3823	1440.7733	720.8903	12
5	659.3399	330.1736	642.3134	321.6603	641.3293	321.1683	P	1343.7569	672.3821	1326.7304	663.8688	1325.7464	663.3768	11
6	815.4662	408.2367	798.4396	399.7234	797.4556	399.2314	K	1246.7042	623.8557	1229.6776	615.3424	1228.6936	614.8504	10
7	962.5346	481.7709	945.5080	473.2577	944.5240	472.7656	F	1090.5779	545.7926	1073.5514	537.2793	1072.5673	536.7873	9
8	1091.5772	546.2922	1074.5506	537.7790	1073.5666	537.2869	E	943.5095	472.2584	926.4829	463.7451	925.4989	463.2531	8
9	1190.6456	595.8264	1173.6190	587.3132	1172.6350	586.8211	V	814.4669	407.7371	797.4403	399.2238	796.4563	398.7318	7
10	1303.7297	652.3685	1286.7031	643.8552	1285.7191	643.3632	I	715.3985	358.2029	698.3719	349.6896	697.3879	349.1976	6
11	1418.7566	709.8819	1401.7300	701.3687	1400.7460	700.8767	D	602.3144	301.6608	585.2879	293.1476	584.3039	292.6556	5
12	1574.8829	787.9451	1557.8563	779.4318	1556.8723	778.9398	K	487.2875	244.1474	470.2609	235.6341	469.2769	235.1421	4
13	1671.9356	836.4714	1654.9091	827.9582	1653.9251	827.4662	P	331.1612	166.0842	314.1347	157.5710	313.1506	157.0790	3
14	1799.9942	900.5007	1782.9677	891.9875	1781.9836	891.4955	Q	234.1084	117.5579	217.0819	109.0446	216.0979	108.5526	2
15							S	106.0499	53.5286			88.0393	44.5233	1

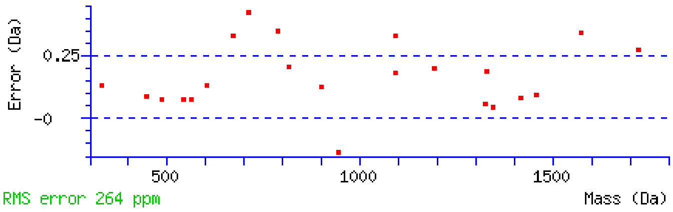

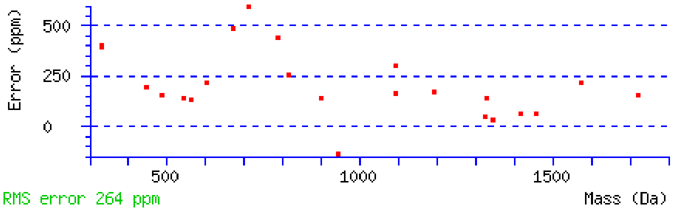

NCBI **BLAST** search of KFDDPKFEVIDKPQS
(Parameters: blastp, nr protein database, expect=20000, no filter, PAM30)
Other BLAST web gateways

All matches to this query

Score	Mr(calc)	Delta	Sequence
44.9	1904.0295	0.0088	KFDDPKFEVIDKPQS
19.1	1903.9836	0.0548	DPVSMPGSSRAVSDRRGV
16.5	1903.9877	0.0506	THHVLRSTDWDRRR
16.4	1903.9965	0.0419	MKDDPITLFVALSPQGT
16.2	1903.8736	0.1648	NFDFDQMYPVALVML
16.1	1904.0903	-0.0520	MKDDPITLFVALSPQGT
15.2	1903.9164	0.1220	GGDFDAKQSLVGDIGDVN
14.2	1904.0458	-0.0074	FTSPPPRDFLLDIARQ
14.1	1903.8847	0.1537	TLPFQGNATNRDHSSMT
14.0	1904.0511	-0.0127	MFDGSVLAAYGNVIVVTL

Mascot: http://www.matrixscience.com/

[redacted] Mascot Search Results

Peptide View

MS/MS Fragmentation of **AANSKVAFSAVRSTN**
Found in **CBLN4_MOUSE** in **SwissProt**, Cerebellin-4 OS=Mus musculus GN=Cbln4 PE=1 SV=1

Match to Query 5849: 1577.853468 from(789.934010,2+) intensity(26784.0980) scans(3621) rawscans(sn3621) rtinseconds(1555.4991) index(1881)
Title: 1882: Scan 3621 (rt=1555.5) [C:\Users\Administrador\Desktop\AMOSTRAS NO ORBITRAP LEANDRO RODAR NOVAMENTE\CORTEX\cortex_01.raw]
Data file cortex_01.temp.mgf

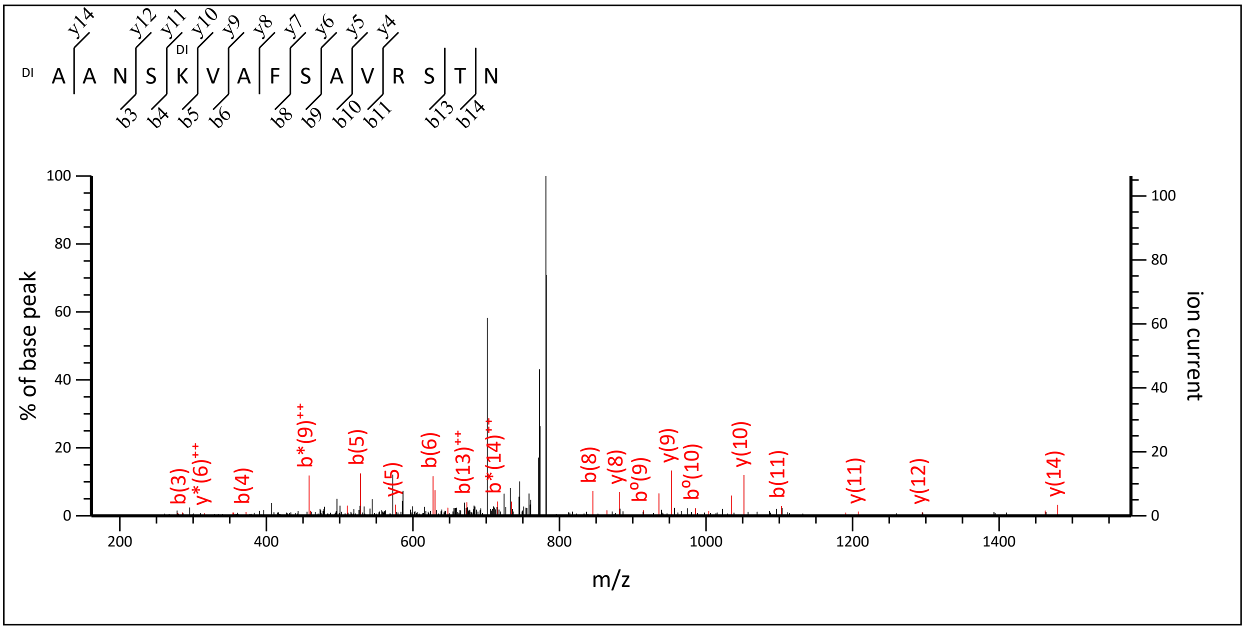

161.21 to 1579.93

Label all possible matches ○ Label matches used for scoring ◉

```
Monoisotopic mass of neutral peptide Mr(calc): 1577.8525
Variable modifications:
N-term : Dimethyl (N-term)
K5     : Dimethyl (K)
Ions Score: 28  Expect: 50
Matches : 36/156 fragment ions using 100 most intense peaks   (help)
```

#	b	b^{++}	b*	b^{*++}	b^0	b^{0++}	Seq.	y	y^{++}	y*	y^{*++}	y^0	y^{0++}	#
1	100.0757	50.5415					A							15
2	171.1128	86.0600					A	1479.7914	740.3993	1462.7649	731.8861	1461.7809	731.3941	14
3	285.1557	143.0815	268.1292	134.5682			N	1408.7543	704.8808	1391.7278	696.3675	1390.7437	695.8755	13
4	372.1878	186.5975	355.1612	178.0842	354.1772	177.5922	S	1294.7114	647.8593	1277.6848	639.3461	1276.7008	638.8540	12
5	528.3140	264.6606	511.2875	256.1474	510.3035	255.6554	K	1207.6793	604.3433	1190.6528	595.8300	1189.6688	595.3380	11
6	627.3824	314.1949	610.3559	305.6816	609.3719	305.1896	V	1051.5531	526.2802	1034.5265	517.7669	1033.5425	517.2749	10
7	698.4195	349.7134	681.3930	341.2001	680.4090	340.7081	A	952.4847	476.7460	935.4581	468.2327	934.4741	467.7407	9
8	845.4880	423.2476	828.4614	414.7343	827.4774	414.2423	F	881.4476	441.2274	864.4210	432.7141	863.4370	432.2221	8
9	932.5200	466.7636	915.4934	458.2504	914.5094	457.7584	S	734.3791	367.6932	717.3526	359.1799	716.3686	358.6879	7
10	1003.5571	502.2822	986.5306	493.7689	985.5465	493.2769	A	647.3471	324.1772	630.3206	315.6639	629.3365	315.1719	6
11	1102.6255	551.8164	1085.5990	543.3031	1084.6150	542.8111	V	576.3100	288.6586	559.2835	280.1454	558.2994	279.6534	5
12	1258.7266	629.8670	1241.7001	621.3537	1240.7161	620.8617	R	477.2416	239.1244	460.2150	230.6112	459.2310	230.1191	4
13	1345.7587	673.3830	1328.7321	664.8697	1327.7481	664.3777	S	321.1405	161.0739	304.1139	152.5606	303.1299	152.0686	3
14	1446.8063	723.9068	1429.7798	715.3935	1428.7958	714.9015	T	234.1084	117.5579	217.0819	109.0446	216.0979	108.5526	2
15							N	133.0608	67.0340	116.0342	58.5207			1

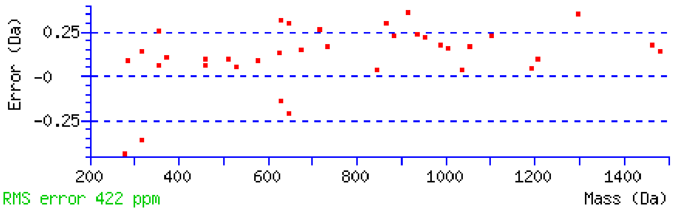

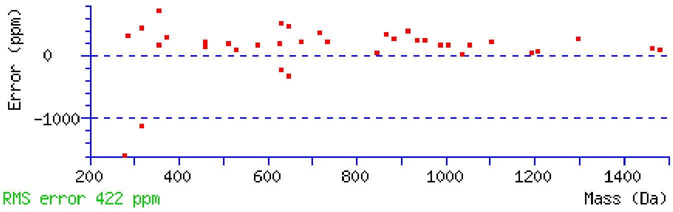

NCBI **BLAST** search of AANSKVAFSAVRSTN
(Parameters: blastp, nr protein database, expect=20000, no filter, PAM30)
Other BLAST web gateways

All matches to this query

Score	Mr(calc)	Delta	Sequence
27.9	1577.8525	0.0009	AANSKVAFSAVRSTN
17.5	1577.8314	0.0220	VTTRVWNRTFAAS
16.4	1577.7435	0.1099	ASLYVAMNGEGYLY
14.5	1577.7825	0.0710	VDEGGVFESLKAET
13.9	1577.9323	-0.0788	GRHVVAVLPEIDPV
13.6	1577.9273	-0.0738	LISIKANCIDSTASA
13.5	1577.9365	-0.0830	ASREHLKRSLALAA
13.4	1577.8049	0.0486	EDVPSQPPENVRAL
13.0	1577.9029	-0.0494	KVEDVVETLLQLH
12.9	1577.8161	0.0373	LTENPQVAQRANAP

Mascot: http://www.matrixscience.com/

Mascot Search Results

Peptide View

MS/MS Fragmentation of **SKVAFSAVRSTN**
Found in **CBLN4_MOUSE** in **SwissProt**, Cerebellin-4 OS=Mus musculus GN=Cbln4 PE=1 SV=1

Match to Query 3962: 1321.739472 from(441.587100,3+) intensity(89601.6020) scans(3471) rawscans(sn3471) rtinseconds(1502.291) index(1720)
Title: 1721: Scan 3471 (rt=1502.29) [C:\Users\Administrador\Desktop\AMOSTRAS NO ORBITRAP LEANDRO RODAR NOVAMENTE\HIPOCAMPO\hipocampo_01.raw]
Data file hipocampo_01.temp.mgf

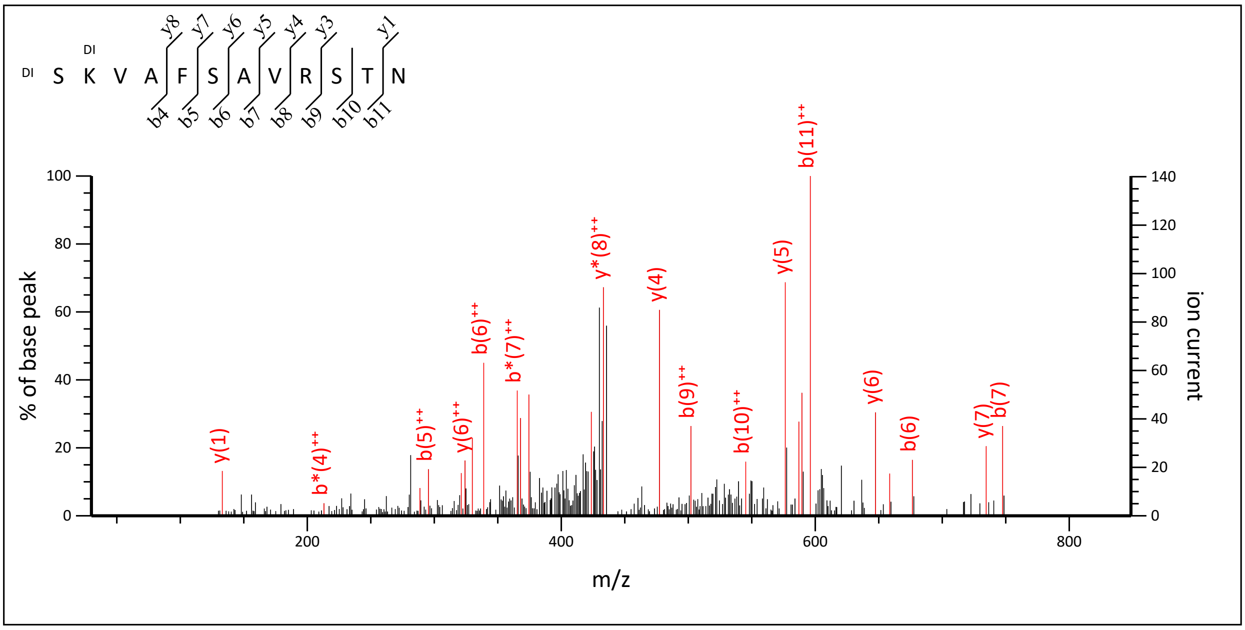

30.01 to 848.6

Label all possible matches ○ Label matches used for scoring ◉

Monoisotopic mass of neutral peptide Mr(calc): 1321.7354
Variable modifications:
N-term : Dimethyl (N-term)
K2 : Dimethyl (K)
Ions Score: 44 **Expect:** 1.3
Matches : 31/128 fragment ions using 41 most intense peaks (help)

#	b	b++	b*	b*++	b0	b0++	Seq.	y	y++	y*	y*++	y0	y0++	#
1	116.0706	58.5389			98.0600	49.5337	S							12
2	272.1969	136.6021	255.1703	128.0888	254.1863	127.5968	K	1207.6793	604.3433	1190.6528	595.8300	1189.6688	595.3380	11
3	371.2653	186.1363	354.2387	177.6230	353.2547	177.1310	V	1051.5531	526.2802	1034.5265	517.7669	1033.5425	517.2749	10
4	442.3024	221.6548	425.2758	213.1416	424.2918	212.6496	A	952.4847	476.7460	935.4581	468.2327	934.4741	467.7407	9
5	589.3708	295.1890	572.3443	286.6758	571.3602	286.1838	F	881.4476	441.2274	864.4210	432.7141	863.4370	432.2221	8
6	676.4028	338.7051	659.3763	330.1918	658.3923	329.6998	S	734.3791	367.6932	717.3526	359.1799	716.3686	358.6879	7
7	747.4400	374.2236	730.4134	365.7103	729.4294	365.2183	A	647.3471	324.1772	630.3206	315.6639	629.3365	315.1719	6
8	846.5084	423.7578	829.4818	415.2445	828.4978	414.7525	V	576.3100	288.6586	559.2835	280.1454	558.2994	279.6534	5
9	1002.6095	501.8084	985.5829	493.2951	984.5989	492.8031	R	477.2416	239.1244	460.2150	230.6112	459.2310	230.1191	4
10	1089.6415	545.3244	1072.6150	536.8111	1071.6309	536.3191	S	321.1405	161.0739	304.1139	152.5606	303.1299	152.0686	3
11	1190.6892	595.8482	1173.6626	587.3350	1172.6786	586.8429	T	234.1084	117.5579	217.0819	109.0446	216.0979	108.5526	2
12							N	133.0608	67.0340	116.0342	58.5207			1

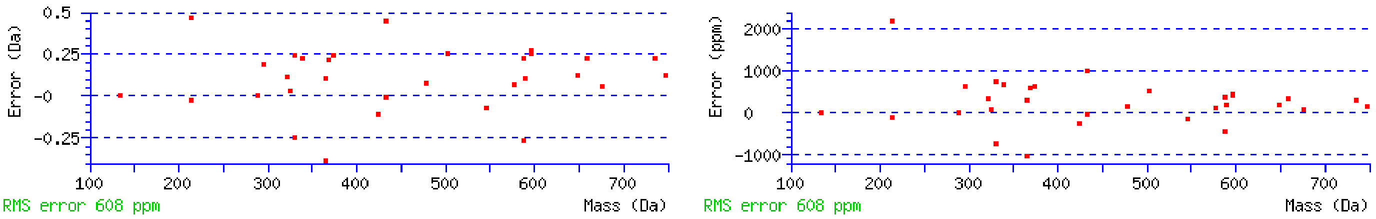

NCBI **BLAST** search of SKVAFSAVRSTN
(Parameters: blastp, nr protein database, expect=20000, no filter, PAM30)
Other BLAST web gateways

All matches to this query

Score	Mr(calc)	Delta	Sequence
43.7	1321.7354	0.0041	SKVAFSAVRSTN
32.1	1321.6700	0.0695	RQFSSALIDCL
28.0	1321.6849	0.0545	CQAALGASEASGLL
25.8	1321.6048	0.1347	REDENASAADVN
25.6	1321.6664	0.0731	VETTATGQQVTN
25.6	1321.7486	-0.0091	GVTMKGTQDNSL
25.4	1321.6486	0.0909	GLLSMANSGPSTN
24.3	1321.7242	0.0153	FVTLSATNAKTN
23.9	1321.7153	0.0242	QSSQLANHIRH
23.2	1321.7704	-0.0309	KDPYTGEQISL

Mascot: http://www.matrixscience.com/

[redacted] Mascot Search Results

Peptide View

MS/MS Fragmentation of **ASKGLGSDLDSSLASL**
Found in **AP180_MOUSE** in **SwissProt**, Clathrin coat assembly protein AP180 OS=Mus musculus GN=Snap91 PE=1 SV=1

Match to Query 5405: 1575.841268 from(788.927910,2+) intensity(73989.8200) scans(9747) rawscans(sn9747) rtinseconds(3652.6788) index(7038)
Title: 7039: Scan 9747 (rt=3652.68) [C:\Users\Administrador\Desktop\AMOSTRAS NO ORBITRAP LEANDRO RODAR NOVAMENTE\HIPOCAMPO\hipocampo_01.raw]
Data file hipocampo_01.temp.mgf

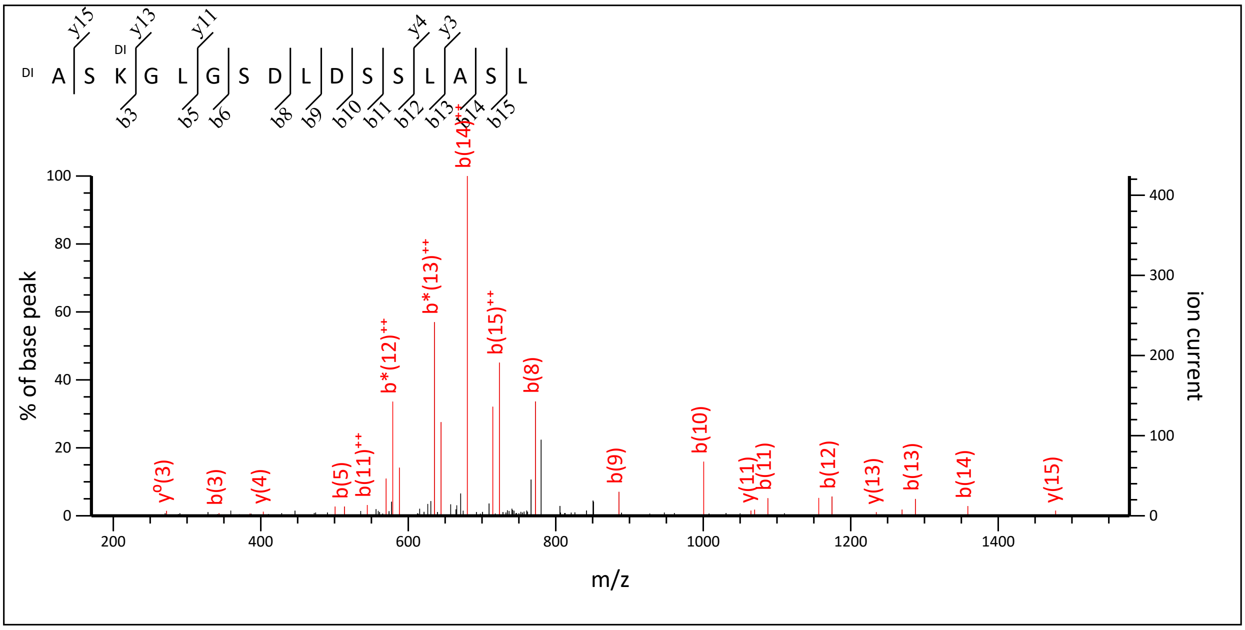

170.33 to 1577.92

Label all possible matches ○ Label matches used for scoring ◉

Monoisotopic mass of neutral peptide Mr(calc): 1575.8356
Variable modifications:
N-term : Dimethyl (N-term)
K3 : Dimethyl (K)
Ions Score: 77 **Expect:** 0.00063
Matches : 32/146 fragment ions using 41 most intense peaks (help)

#	b	b^{++}	b*	b^{*++}	b^0	b^{0++}	Seq.	y	y^{++}	y*	y^{*++}	y^0	y^{0++}	#
1	100.0757	50.5415					A							16
2	187.1077	94.0575			169.0972	85.0522	S	1477.7744	739.3909	1460.7479	730.8776	1459.7639	730.3856	15
3	343.2340	172.1206	326.2074	163.6074	325.2234	163.1153	K	1390.7424	695.8748	1373.7159	687.3616	1372.7318	686.8696	14
4	400.2554	200.6314	383.2289	192.1181	382.2449	191.6261	G	1234.6161	617.8117			1216.6056	608.8064	13
5	513.3395	257.1734	496.3130	248.6601	495.3289	248.1681	L	1177.5947	589.3010			1159.5841	580.2957	12
6	570.3610	285.6841	553.3344	277.1709	552.3504	276.6788	G	1064.5106	532.7589			1046.5000	523.7537	11
7	657.3930	329.2001	640.3665	320.6869	639.3824	320.1949	S	1007.4891	504.2482			989.4786	495.2429	10
8	772.4199	386.7136	755.3934	378.2003	754.4094	377.7083	D	920.4571	460.7322			902.4466	451.7269	9
9	885.5040	443.2556	868.4775	434.7424	867.4934	434.2504	L	805.4302	403.2187			787.4196	394.2134	8
10	1000.5310	500.7691	983.5044	492.2558	982.5204	491.7638	D	692.3461	346.6767			674.3355	337.6714	7
11	1087.5630	544.2851	1070.5364	535.7719	1069.5524	535.2798	S	577.3192	289.1632			559.3086	280.1579	6
12	1174.5950	587.8011	1157.5685	579.2879	1156.5844	578.7959	S	490.2871	245.6472			472.2766	236.6419	5
13	1287.6791	644.3432	1270.6525	635.8299	1269.6685	635.3379	L	403.2551	202.1312			385.2445	193.1259	4
14	1358.7162	679.8617	1341.6896	671.3485	1340.7056	670.8564	A	290.1710	145.5892			272.1605	136.5839	3
15	1445.7482	723.3777	1428.7217	714.8645	1427.7376	714.3725	S	219.1339	110.0706			201.1234	101.0653	2
16							L	132.1019	66.5546					1

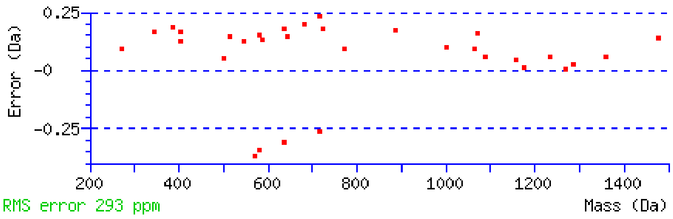

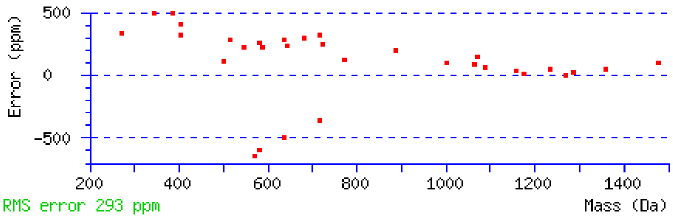

NCBI **BLAST** search of ASKGLGSDLDSSLASL
(Parameters: blastp, nr protein database, expect=20000, no filter, PAM30)
Other BLAST web gateways

All matches to this query

Score	Mr(calc)	Delta	Sequence
76.9	1575.8356	0.0057	ASKGLGSDLDSSLASL
33.2	1575.8615	-0.0203	LAVMLAALMSSLASI
31.9	1575.7613	0.0800	AHRMDSLDSRDSI
29.7	1575.8615	-0.0203	LAVMLAALMSSLASI
28.0	1575.9021	-0.0609	VSALSSAGGTENLILL
27.7	1575.9323	-0.0911	SVALSPTPTKSTPNI
27.2	1575.7330	0.1083	AGSLGPWGPMSSGPW
26.7	1575.7450	0.0962	NMLSSDVAQLSDPGV
25.8	1576.1168	-0.2755	KPISVESSSKKVK
25.8	1575.9134	-0.0721	LPLVSGRSASSSLASL

Mascot: http://www.matrixscience.com/

[redacted] Mascot Search Results

Peptide View

MS/MS Fragmentation of **KGLGSDLDSSLASL**
Found in **AP180_MOUSE** in **SwissProt**, Clathrin coat assembly protein AP180 OS=Mus musculus GN=Snap91 PE=1 SV=1

Match to Query 4749: 1417.773008 from(709.893780,2+) intensity(33636.4020) scans(9499) rawscans(sn9499) rtinseconds(3590.1007) index(6933)
Title: 6934: Scan 9499 (rt=3590.1) [C:\Users\Administrador\Desktop\AMOSTRAS NO ORBITRAP LEANDRO RODAR NOVAMENTE\CORTEX\cortex_01.raw]
Data file cortex_01.temp.mgf

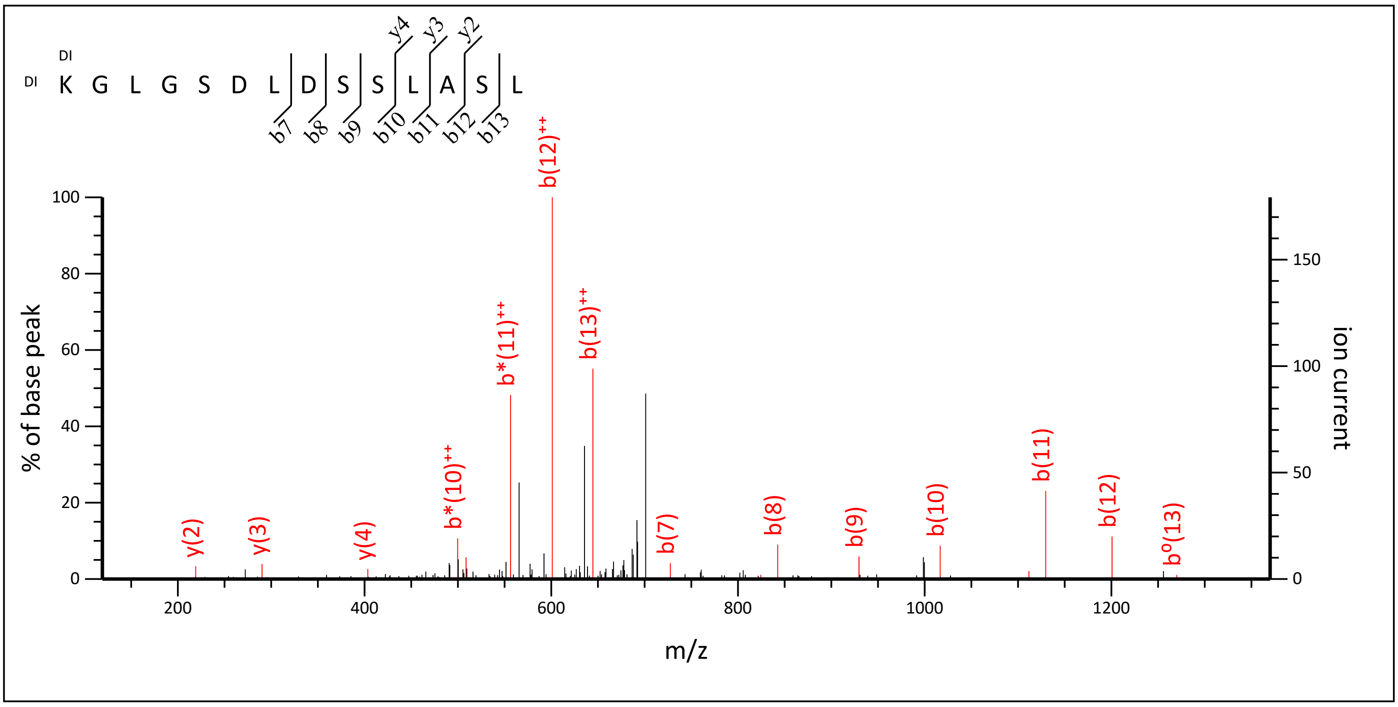

119.14 to 1370.03

Label all possible matches Label matches used for scoring

```
Monoisotopic mass of neutral peptide Mr(calc): 1417.7664
Variable modifications:
N-term : Dimethyl (N-term)
K1     : Dimethyl (K)
Ions Score: 57  Expect: 0.065
Matches : 21/120 fragment ions using 22 most intense peaks   (help)
```

#	b	b++	b*	b*++	b0	b0++	Seq.	y	y++	y*++	y0	y0++	#
1	185.1648	93.0861	168.1383	84.5728			K						14
2	242.1863	121.5968	225.1598	113.0835			G	1234.6161	617.8117		1216.6056	608.8064	13
3	355.2704	178.1388	338.2438	169.6255			L	1177.5947	589.3010		1159.5841	580.2957	12
4	412.2918	206.6496	395.2653	198.1363			G	1064.5106	532.7589		1046.5000	523.7537	11
5	***499.3239***	250.1656	482.2973	241.6523	481.3133	241.1603	S	1007.4891	504.2482		989.4786	495.2429	10
6	614.3508	307.6790	597.3243	299.1658	596.3402	298.6738	D	920.4571	460.7322		902.4466	451.7269	9
7	***727.4349***	364.2211	710.4083	355.7078	709.4243	355.2158	L	805.4302	403.2187		787.4196	394.2134	8
8	***842.4618***	421.7345	825.4353	413.2213	824.4512	412.7293	D	692.3461	346.6767		674.3355	337.6714	7
9	***929.4938***	465.2506	912.4673	456.7373	911.4833	456.2453	S	577.3192	289.1632		559.3086	280.1579	6
10	***1016.5259***	**508.7666**	999.4993	500.2533	998.5153	499.7613	S	490.2871	245.6472		472.2766	236.6419	5
11	***1129.6099***	565.3086	1112.5834	556.7953	1111.5994	556.3033	L	**403.2551**	202.1312		385.2445	193.1259	4
12	***1200.6470***	**600.8272**	1183.6205	592.3139	1182.6365	591.8219	A	**290.1710**	145.5892		272.1605	136.5839	3

**13**	1287.6791	**644.3432**	1270.6525	635.8299	1269.6685	635.3379	**S**	**219.1339**	110.0706		201.1234	101.0653	**2**
**14**							**L**	132.1019	66.5546				**1**

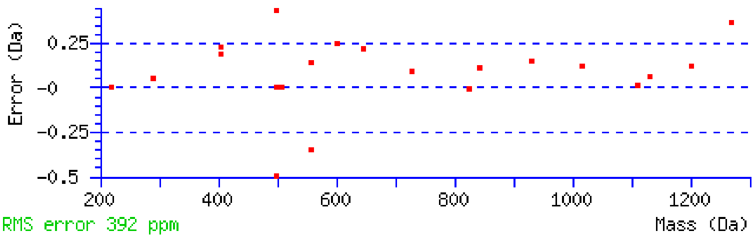

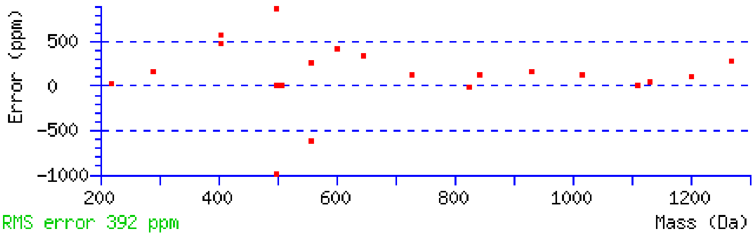

NCBI **BLAST** search of KGLGSDLDSSLASL
(Parameters: blastp, nr protein database, expect=20000, no filter, PAM30)
Other BLAST web gateways

All matches to this query

Score	Mr(calc)	Delta	Sequence
56.7	1417.7664	0.0066	KGLGSDLDSSLASL
46.6	1417.7010	0.0720	MSLPDLDSSLASI
40.7	1417.7706	0.0024	VNHADSRASLASL
36.8	1417.6962	0.0768	WALPGGSREPGEM
36.1	1417.7920	-0.0190	LMDPVDGPILASL
36.0	1417.7463	0.0267	GQRHETTSSIASL
35.8	1417.8788	-0.1058	AKLQMTEAALALS
35.4	1417.7061	0.0670	AEHMSDLSALASI
35.2	1417.8079	-0.0349	GEALLDSGVRVTAV
34.0	1417.7238	0.0492	GSAISPDLLVDSNG

Mascot: http://www.matrixscience.com/

[redacted] Mascot Search Results

Peptide View

MS/MS Fragmentation of SKGLGSDLDSSLASL
Found in AP180_MOUSE in SwissProt, Clathrin coat assembly protein AP180 OS=Mus musculus GN=Snap91 PE=1 SV=1

Match to Query 5450: 1512.856708 from(757.435630,2+) intensity(167512.9800) scans(9658) rawscans(sn9658) rtinseconds(3646.6755) index(7067)
Title: 7068: Scan 9658 (rt=3646.68) [C:\Users\Administrador\Desktop\AMOSTRAS NO ORBITRAP LEANDRO RODAR NOVAMENTE\CORTEX\cortex_01.raw]
Data file cortex_01.temp.mgf

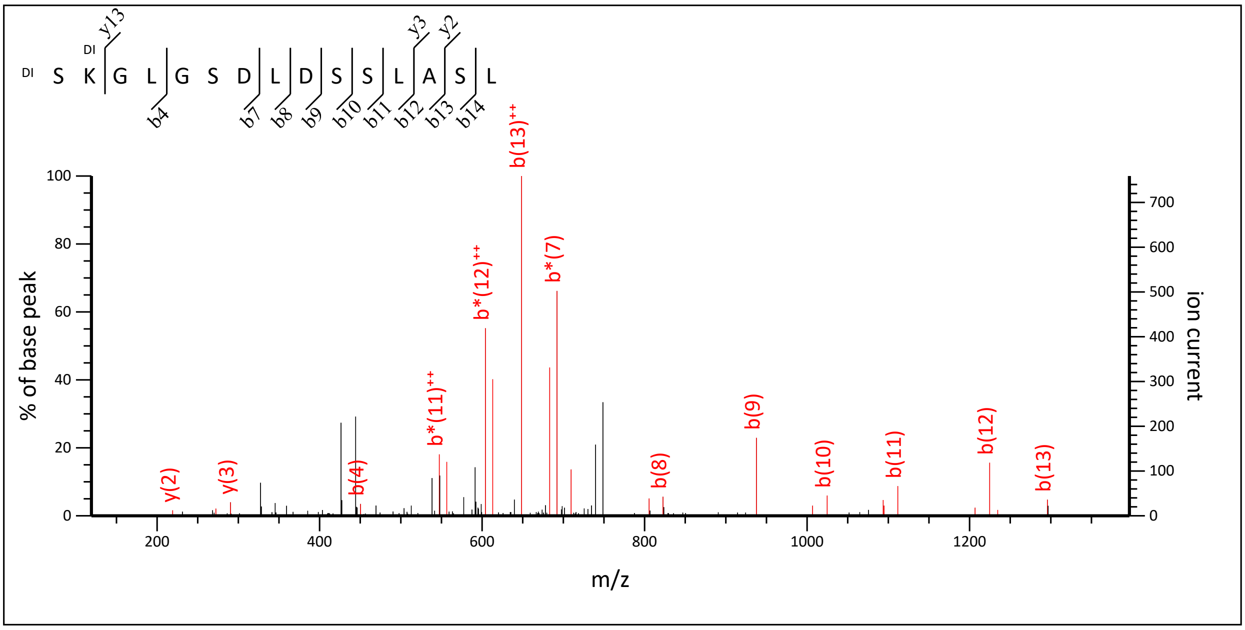

119.17 to 1396.72

Label all possible matches ○ Label matches used for scoring ◉

Monoisotopic mass of neutral peptide Mr(calc): 1512.8487
Variable modifications:
N-term : Dimethyl:2H(4) (N-term)
K2 : Dimethyl:2H(4) (K)
Ions Score: 51 **Expect:** 0.25
Matches : 30/138 fragment ions using 40 most intense peaks (help)

#	b	b++	b*	b*++	b0	b0++	Seq.	y	y++	y*	y*++	y0	y0++	#
1	120.0957	60.5515			102.0851	51.5462	S							15
2	280.2471	140.6272	263.2205	132.1139	262.2365	131.6219	K	1394.7675	697.8874	1377.7410	689.3741	1376.7569	688.8821	14
3	337.2685	169.1379	320.2420	160.6246	319.2580	160.1326	G	1234.6161	617.8117			1216.6056	608.8064	13
4	450.3526	225.6799	433.3261	217.1667	432.3420	216.6747	L	1177.5947	589.3010			1159.5841	580.2957	12
5	507.3741	254.1907	490.3475	245.6774	489.3635	245.1854	G	1064.5106	532.7589			1046.5000	523.7537	11
6	594.4061	297.7067	577.3796	289.1934	576.3955	288.7014	S	1007.4891	504.2482			989.4786	495.2429	10
7	709.4330	355.2202	692.4065	346.7069	691.4225	346.2149	D	920.4571	460.7322			902.4466	451.7269	9
8	822.5171	411.7622	805.4906	403.2489	804.5065	402.7569	L	805.4302	403.2187			787.4196	394.2134	8
9	937.5441	469.2757	920.5175	460.7624	919.5335	460.2704	D	692.3461	346.6767			674.3355	337.6714	7
10	1024.5761	512.7917	1007.5495	504.2784	1006.5655	503.7864	S	577.3192	289.1632			559.3086	280.1579	6
11	1111.6081	556.3077	1094.5816	547.7944	1093.5975	547.3024	S	490.2871	245.6472			472.2766	236.6419	5
12	1224.6922	612.8497	1207.6656	604.3364	1206.6816	603.8444	L	403.2551	202.1312			385.2445	193.1259	4
13	1295.7293	648.3683	1278.7027	639.8550	1277.7187	639.3630	A	290.1710	145.5892			272.1605	136.5839	3
14	1382.7613	691.8843	1365.7348	683.3710	1364.7507	682.8790	S	219.1339	110.0706			201.1234	101.0653	2
15							L	132.1019	66.5546					1

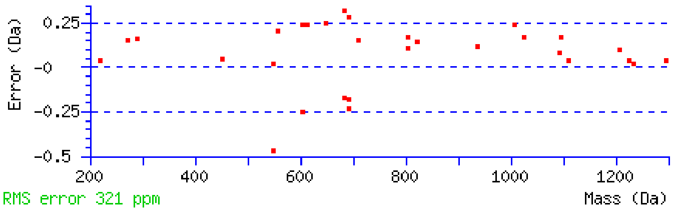

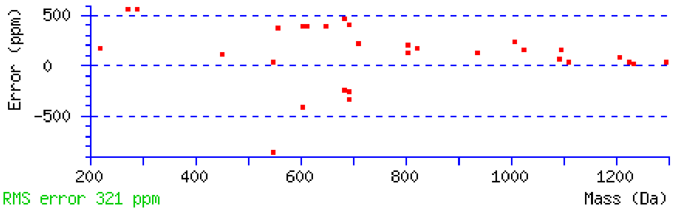

NCBI **BLAST** search of SKGLGSDLDSSLASL
(Parameters: blastp, nr protein database, expect=20000, no filter, PAM30)
Other BLAST web gateways

All matches to this query

Score	Mr(calc)	Delta	Sequence
50.8	1512.8487	0.0080	SKGLGSDLDSSLASL
39.4	1512.8293	0.0274	GGPHTKMGGSRDSL
35.7	1512.9160	-0.0593	MTKPLVHSSLALS
31.8	1513.0396	-0.1829	NFLTAIKKVIASL
29.1	1512.7821	0.0746	QSLSSSSTATGTTVI
28.8	1512.9037	-0.0469	VASLLNSMKSIASL
28.3	1512.6878	0.1689	NCDSSLNRTYAIS
27.7	1512.8286	0.0281	EPELLTALSISASL
27.3	1512.8562	0.0005	VSALAQDTHRATLV
26.8	1512.8309	0.0258	SQLSMKASLENSL

Mascot: http://www.matrixscience.com/

[redacted] Mascot Search Results

Peptide View

MS/MS Fragmentation of **SPSPTPATQSPKKPPAKDPLADLNIKDFL**
Found in **AP180_MOUSE** in **SwissProt**, Clathrin coat assembly protein AP180 OS=Mus musculus GN=Snap91 PE=1 SV=1

Match to Query 10492: 3232.944496 from(809.243400,4+) intensity(189922.9200) scans(10602) rawscans(sn10602) rtinseconds(3964.6895) index(7798)
Title: 7799: Scan 10602 (rt=3964.69) [C:\Users\Administrador\Desktop\AMOSTRAS NO ORBITRAP LEANDRO RODAR NOVAMENTE\HIPOCAMPO\hipocampo_01.raw]
Data file hipocampo_01.temp.mgf

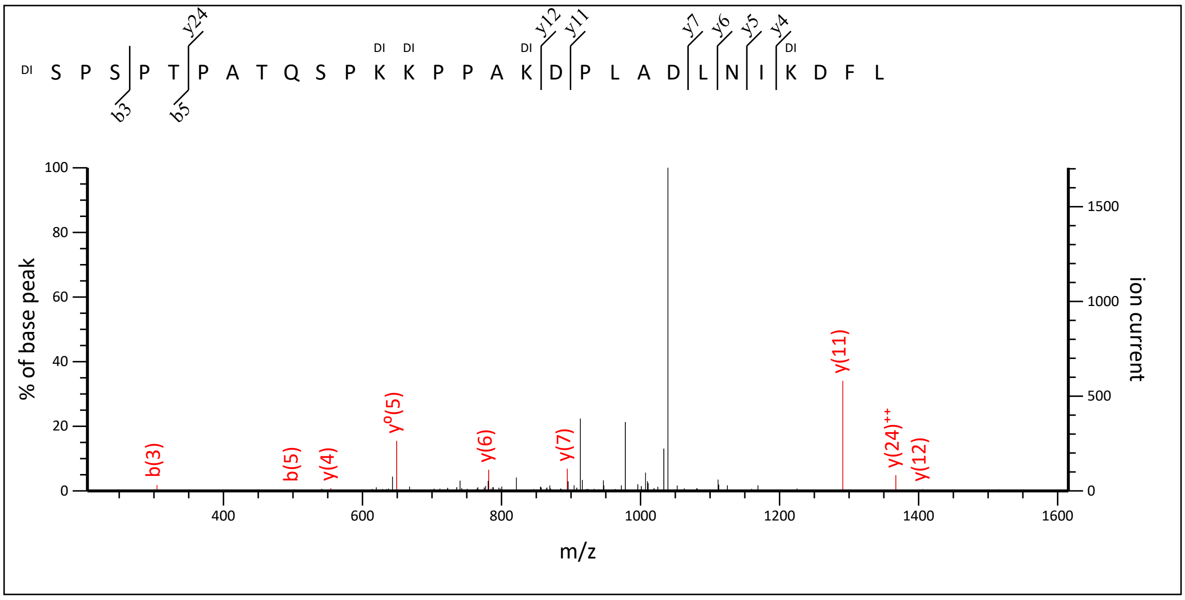

204.27 to 1615.4

Label all possible matches ○ Label matches used for scoring ◉

Monoisotopic mass of neutral peptide Mr(calc): 3232.9316
Variable modifications:
N-term : Dimethyl:2H(4) (N-term)
K12 : Dimethyl:2H(4) (K)
K13 : Dimethyl:2H(4) (K)
K17 : Dimethyl:2H(4) (K)
K26 : Dimethyl:2H(4) (K)
Ions Score: 20 **Expect:** 3.3e+002
Matches : 9/310 fragment ions using 13 most intense peaks (help)

#	b	b++	b*	b*++	b0	b0++	Seq.	y	y++	y*	y*++	y0	y0++	#
1	120.0957	60.5515			102.0851	51.5462	S							29
2	217.1485	109.0779			199.1379	100.0726	P	3114.8504	1557.9289	3097.8239	1549.4156	3096.8399	1548.9236	28
3	**304.1805**	152.5939			286.1699	143.5886	S	3017.7977	1509.4025	3000.7711	1500.8892	2999.7871	1500.3972	27
4	401.2333	201.1203			383.2227	192.1150	P	2930.7656	1465.8865	2913.7391	1457.3732	2912.7551	1456.8812	26
5	**502.2809**	251.6441			484.2704	242.6388	T	2833.7129	1417.3601	2816.6863	1408.8468	2815.7023	1408.3548	25
6	599.3337	300.1705			581.3231	291.1652	P	2732.6652	1366.8362	2715.6386	1358.3230	2714.6546	1357.8310	24
7	670.3708	335.6891			652.3603	326.6838	A	2635.6124	1318.3099	2618.5859	1309.7966	2617.6019	1309.3046	23
8	771.4185	386.2129			753.4079	377.2076	T	2564.5753	1282.7913	2547.5488	1274.2780	2546.5648	1273.7860	22
9	899.4771	450.2422	882.4505	441.7289	881.4665	441.2369	Q	2463.5276	1232.2675	2446.5011	1223.7542	2445.5171	1223.2622	21
10	986.5091	493.7582	969.4826	485.2449	968.4985	484.7529	S	2335.4691	1168.2382	2318.4425	1159.7249	2317.4585	1159.2329	20
11	1083.5619	542.2846	1066.5353	533.7713	1065.5513	533.2793	P	2248.4370	1124.7222	2231.4105	1116.2089	2230.4265	1115.7169	19
12	1243.7132	622.3603	1226.6867	613.8470	1225.7027	613.3550	K	2151.3843	1076.1958	2134.3577	1067.6825	2133.3737	1067.1905	18
13	1403.8646	702.4359	1386.8381	693.9227	1385.8540	693.4307	K	1991.2329	996.1201	1974.2063	987.6068	1973.2223	987.1148	17
14	1500.9174	750.9623	1483.8908	742.4491	1482.9068	741.9570	P	1831.0815	916.0444	1814.0550	907.5311	1813.0710	907.0391	16
15	1597.9701	799.4887	1580.9436	790.9754	1579.9596	790.4834	P	1734.0288	867.5180	1717.0022	859.0047	1716.0182	858.5127	15
16	1669.0073	835.0073	1651.9807	826.4940	1650.9967	826.0020	A	1636.9760	818.9916	1619.9495	810.4784	1618.9654	809.9864	14
17	1829.1586	915.0830	1812.1321	906.5697	1811.1481	906.0777	K	1565.9389	783.4731	1548.9123	774.9598	1547.9283	774.4678	13
18	1944.1856	972.5964	1927.1590	964.0831	1926.1750	963.5911	D	***1405.7875***	703.3974	1388.7610	694.8841	1387.7770	694.3921	12
19	2041.2383	1021.1228	2024.2118	1012.6095	2023.2278	1012.1175	P	***1290.7606***	645.8839	1273.7340	637.3706	1272.7500	636.8786	11

20	2154.3224	1077.6648	2137.2958	1069.1516	2136.3118	1068.6596	L	1193.7078	597.3575	1176.6813	588.8443	1175.6972	588.3523	10
21	2225.3595	1113.1834	2208.3330	1104.6701	2207.3489	1104.1781	A	1080.6237	540.8155	1063.5972	532.3022	1062.6132	531.8102	9
22	2340.3865	1170.6969	2323.3599	1162.1836	2322.3759	1161.6916	D	1009.5866	505.2970	992.5601	496.7837	991.5761	496.2917	8
23	2453.4705	1227.2389	2436.4440	1218.7256	2435.4600	1218.2336	L	***894.5597***	447.7835	877.5331	439.2702	876.5491	438.7782	7
24	2567.5134	1284.2604	2550.4869	1275.7471	2549.5029	1275.2551	N	***781.4756***	391.2414	764.4491	382.7282	763.4651	382.2362	6
25	2680.5975	1340.8024	2663.5710	1332.2891	2662.5869	1331.7971	I	667.4327	334.2200	650.4061	325.7067	649.4221	325.2147	5
26	2840.7489	1420.8781	2823.7223	1412.3648	2822.7383	1411.8728	K	***554.3486***	277.6780	537.3221	269.1647	536.3381	268.6727	4
27	2955.7758	1478.3915	2938.7493	1469.8783	2937.7653	1469.3863	D	394.1973	197.6023			376.1867	188.5970	3
28	3102.8442	1551.9258	3085.8177	1543.4125	3084.8337	1542.9205	F	279.1703	140.0888					2
29							L	132.1019	66.5546					1

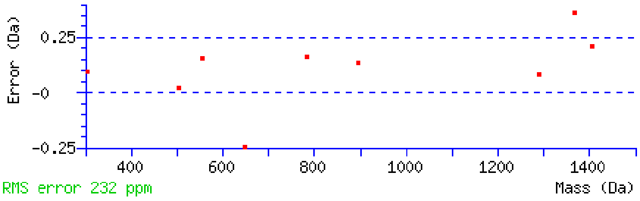

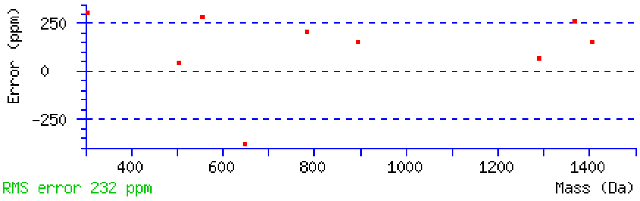

NCBI **BLAST** search of SPSPTPATQSPKKPPAKDPLADLNIKDFL
(Parameters: blastp, nr protein database, expect=20000, no filter, PAM30)
Other BLAST web gateways

All matches to this query

Score	Mr(calc)	Delta	Sequence
20.5	3232.9316	0.0129	SPSPTPATQSPKKPPAKDPLADLNIKDFL
18.7	3232.6049	0.3396	FLRFIPTYTSRLAIISQSGQCQFCEPTG
14.3	3232.6132	0.3313	LMAIAHGSGLPLMMIVFGEMTDKFVDNTG
13.9	3232.6975	0.2470	FLTDGASAMLIMSEDRALAMGYKPKAYL
13.7	3232.6431	0.3014	DKDIVMLQTGVSMMDPNHFLMIMLSRF
13.6	3232.7254	0.2190	SYLITTPCYSDAFAKLLESGDLSMNSIK
13.6	3232.5079	0.4366	NIDSDSILMSILDMSLHQQMGSDRDLQS
13.4	3232.7629	0.1816	QSLEYLINDIRPPCIKEQMLGKGYETV
12.7	3232.7576	0.1869	CPNPKFINRSMATKGLLLPSRRSLASFS
11.8	3232.6950	0.2495	PAHPTPFYIDDILGRGPAAPTPTPTLPSPNS

Mascot: http://www.matrixscience.com/

[redacted] Mascot Search Results

Peptide View

MS/MS Fragmentation of **ARSGPFAPVLSAT**
Found in **UCRI_MOUSE** in **SwissProt**, Cytochrome b-c1 complex subunit Rieske, mitochondrial OS=Mus musculus GN=Uqcrfs1 PE=1 SV=1

Match to Query 3942: 1300.719408 from(651.366980,2+) intensity(53800.7110) scans(7400) rawscans(sn7400) rtinseconds(2841.3764) index(5181)
Title: 5182: Scan 7400 (rt=2841.38) [C:\Users\Administrador\Desktop\AMOSTRAS NO ORBITRAP LEANDRO RODAR NOVAMENTE\CORTEX\cortex_01.raw]
Data file cortex_01.temp.mgf

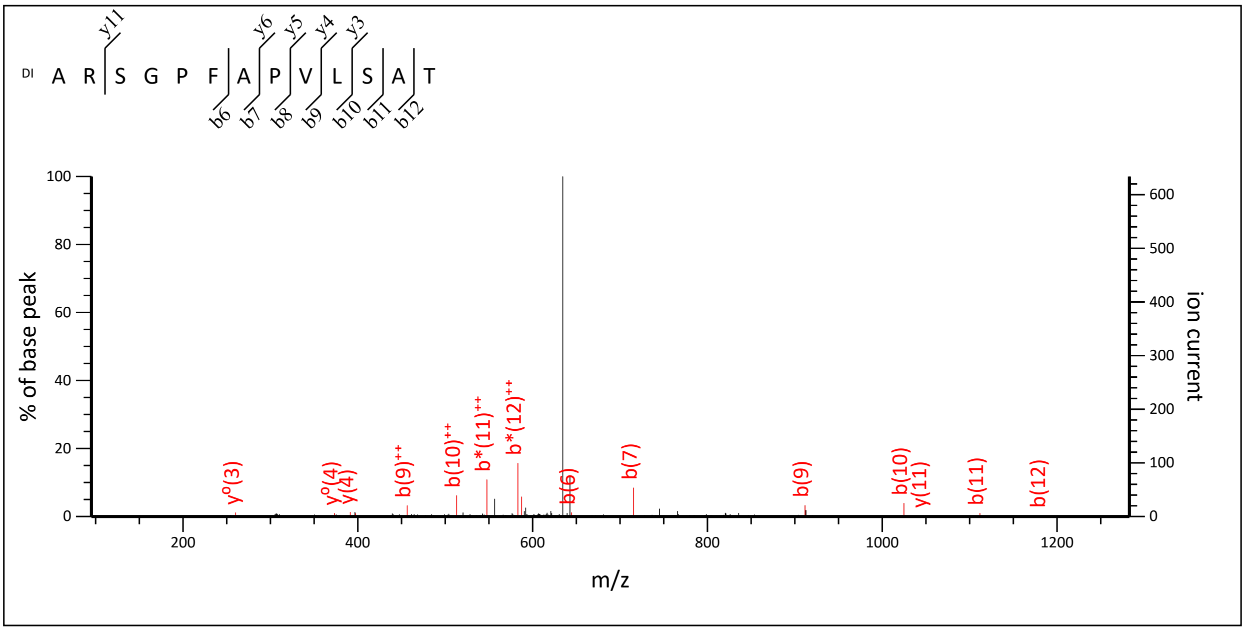

95.19 to 1282.8

Label all possible matches ○ Label matches used for scoring ◉

Monoisotopic mass of neutral peptide Mr(calc): 1300.7139
Variable modifications:
N-term : Dimethyl (N-term)
Ions Score: 24 **Expect:** 1.2e+002
Matches : 19/116 fragment ions using 37 most intense peaks (help)

#	b	b++	b*	b*++	b0	b0++	Seq.	y	y++	y*	y*++	y0	y0++	#
1	100.0757	50.5415					A							13
2	256.1768	128.5920	239.1503	120.0788			R	1202.6528	601.8300	1185.6263	593.3168	1184.6422	592.8248	12
3	343.2088	172.1081	326.1823	163.5948	325.1983	163.1028	S	**1046.5517**	523.7795			1028.5411	514.7742	11
4	400.2303	200.6188	383.2037	192.1055	382.2197	191.6135	G	959.5197	480.2635			941.5091	471.2582	10
5	497.2831	249.1452	480.2565	240.6319	479.2725	240.1399	P	902.4982	451.7527			884.4876	442.7475	9
6	*644.3515*	322.6794	627.3249	314.1661	626.3409	313.6741	F	805.4454	403.2264			787.4349	394.2211	8
7	*715.3886*	358.1979	698.3620	349.6847	697.3780	349.1926	A	658.3770	329.6921			640.3665	320.6869	7
8	812.4413	406.7243	795.4148	398.2110	794.4308	397.7190	P	**587.3399**	294.1736			569.3293	285.1683	6
9	*911.5098*	**456.2585**	894.4832	447.7452	893.4992	447.2532	V	490.2871	245.6472			472.2766	236.6419	5
10	*1024.5938*	**512.8006**	1007.5673	504.2873	1006.5833	503.7953	L	**391.2187**	196.1130			373.2082	187.1077	4
11	*1111.6259*	556.3166	1094.5993	547.8033	1093.6153	547.3113	S	278.1347	139.5710			260.1241	130.5657	3
12	*1182.6630*	591.8351	1165.6364	583.3218	1164.6524	582.8298	A	191.1026	96.0550			173.0921	87.0497	2
13							T	120.0655	60.5364			102.0550	51.5311	1

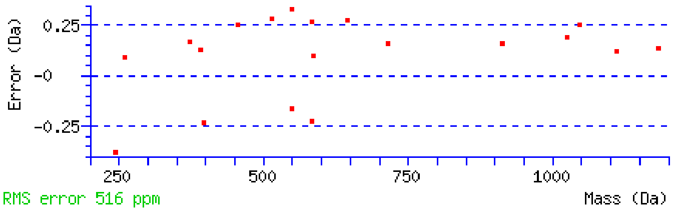

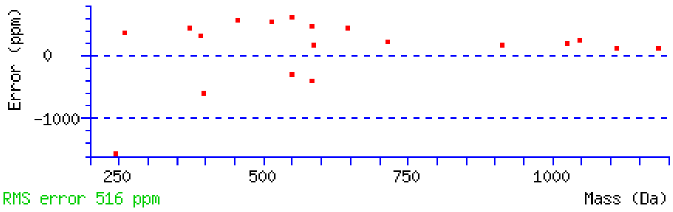

NCBI **BLAST** search of ARSGPFAPVLSAT
(Parameters: blastp, nr protein database, expect=20000, no filter, PAM30)
Other BLAST web gateways

All matches to this query

Score	Mr(calc)	Delta	Sequence
32.7	1300.8016	-0.0822	INITPLARLSAT
32.6	1300.8380	-0.1186	SGTLRLVLGLLQ
24.4	1300.7602	-0.0408	IGDKGVSKLSAT
24.1	1300.7523	-0.0329	TYLGMSKISAT
23.9	1300.7139	0.0055	ARSGPFAPVLSAT
23.6	1300.5964	0.1230	HSSEDYNLSTA
22.9	1300.7420	-0.0226	GALAQPVVPAEAT
20.1	1300.6489	0.0705	NSDYFIPISAT
19.8	1300.7391	-0.0197	KYGTHLLISAT
19.3	1300.6053	0.1141	CSPVQSMRSSAT

Mascot: http://www.matrixscience.com/

[redacted] Mascot Search Results

Peptide View

MS/MS Fragmentation of **GLNVPASVRF**
Found in **UCRI_MOUSE** in **SwissProt**, Cytochrome b-c1 complex subunit Rieske, mitochondrial OS=Mus musculus GN=Uqcrfs1 PE=1 SV=1

Match to Query 2441: 1086.620988 from(544.317770,2+) intensity(154132.7200) scans(8139) rawscans(sn8139) rtinseconds(3109.3608) index(5788)
Title: 5789: Scan 8139 (rt=3109.36) [C:\Users\Administrador\Desktop\AMOSTRAS NO ORBITRAP LEANDRO RODAR NOVAMENTE\CORTEX\cortex_01.raw]
Data file cortex_01.temp.mgf

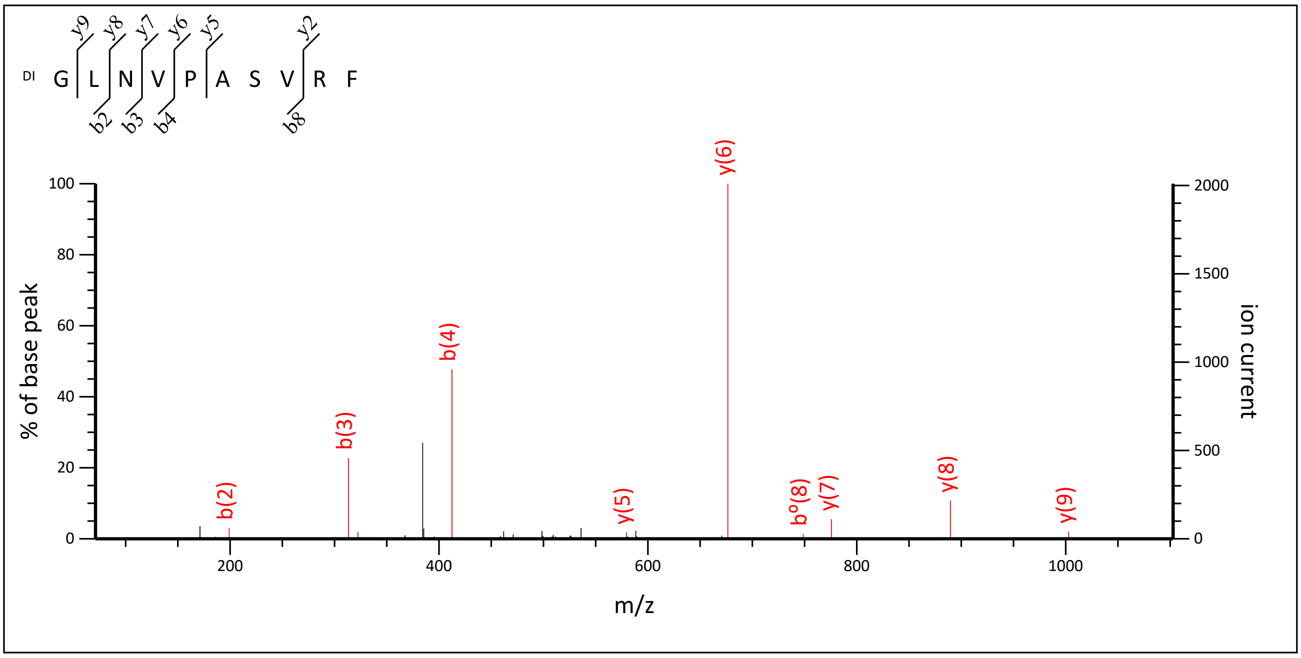

71.14 to 1102.81

Label all possible matches ○ Label matches used for scoring ◉

```
Monoisotopic mass of neutral peptide Mr(calc): 1086.6186
Variable modifications:
N-term : Dimethyl (N-term)
Ions Score: 48  Expect: 0.51
Matches : 10/84 fragment ions using 15 most intense peaks   (help)
```

#	b	b^{++}	b*	b^{*++}	b^0	b^{0++}	Seq.	y	y^{++}	y*	y^{*++}	y^0	y^{0++}	#
1	86.0600	43.5337					G							10
2	**199.1441**	100.0757					L	***1002.5731***	501.7902	985.5465	493.2769	984.5625	492.7849	9
3	**313.1870**	157.0972	296.1605	148.5839			N	***889.4890***	445.2482	872.4625	436.7349	871.4785	436.2429	8
4	**412.2554**	206.6314	395.2289	198.1181			V	***775.4461***	388.2267	758.4196	379.7134	757.4355	379.2214	7
5	509.3082	255.1577	492.2817	246.6445			P	***676.3777***	338.6925	659.3511	330.1792	658.3671	329.6872	6
6	580.3453	290.6763	563.3188	282.1630			A	***579.3249***	290.1661	562.2984	281.6528	561.3144	281.1608	5
7	667.3774	334.1923	650.3508	325.6790	649.3668	325.1870	S	508.2878	254.6475	491.2613	246.1343	490.2772	245.6423	4
8	766.4458	383.7265	749.4192	375.2132	748.4352	374.7212	V	421.2558	211.1315	404.2292	202.6183			3
9	922.5469	461.7771	905.5203	453.2638	904.5363	452.7718	R	***322.1874***	161.5973	305.1608	153.0840			2
10							F	166.0863	83.5468					1

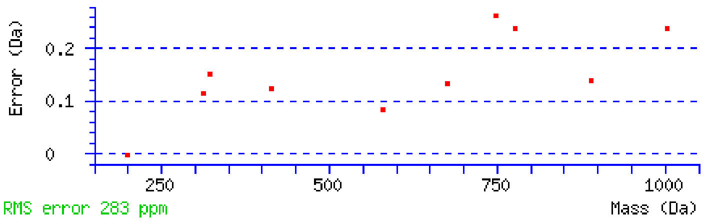

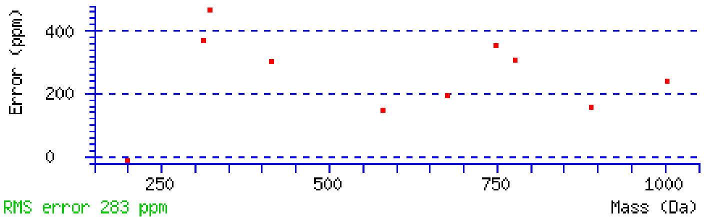

NCBI **BLAST** search of GLNVPASVRF
(Parameters: blastp, nr protein database, expect=20000, no filter, PAM30)
Other BLAST web gateways

All matches to this query

Score	Mr(calc)	Delta	Sequence
47.6	1086.6186	0.0024	GLNVPASVRF
27.5	1086.6107	0.0103	GLGGVGLAVIMG
27.5	1086.6107	0.0103	GLGGVGLSVIMG
24.8	1086.5380	0.0830	QNVGTPVMVG
22.3	1086.5305	0.0904	GLAAAPGGSGAGSS
22.0	1086.5896	0.0314	GIKGPAGMPGF
21.3	1086.5822	0.0388	QNVPRYLQ
21.0	1086.5669	0.0540	QRVPGLSSDT
19.6	1086.5557	0.0653	QNVADSVEK
19.6	1086.5532	0.0678	QVHLGAVFM

Mascot: http://www.matrixscience.com/

[redacted] Mascot Search Results

Peptide View

MS/MS Fragmentation of **SGQAAARPLVA**
Found in **UCRI_MOUSE** in **SwissProt**, Cytochrome b-c1 complex subunit Rieske, mitochondrial OS=Mus musculus GN=Uqcrfs1 PE=1 SV=1

Match to Query 2316: 1071.635948 from(536.825250,2+) intensity(150756.6900) scans(3397) rawscans(sn3397) rtinseconds(1478.1366) index(1654)
Title: 1655: Scan 3397 (rt=1478.14) [C:\Users\Administrador\Desktop\AMOSTRAS NO ORBITRAP LEANDRO RODAR NOVAMENTE\HIPOCAMPO\hipocampo_01.raw]
Data file hipocampo_01.temp.mgf

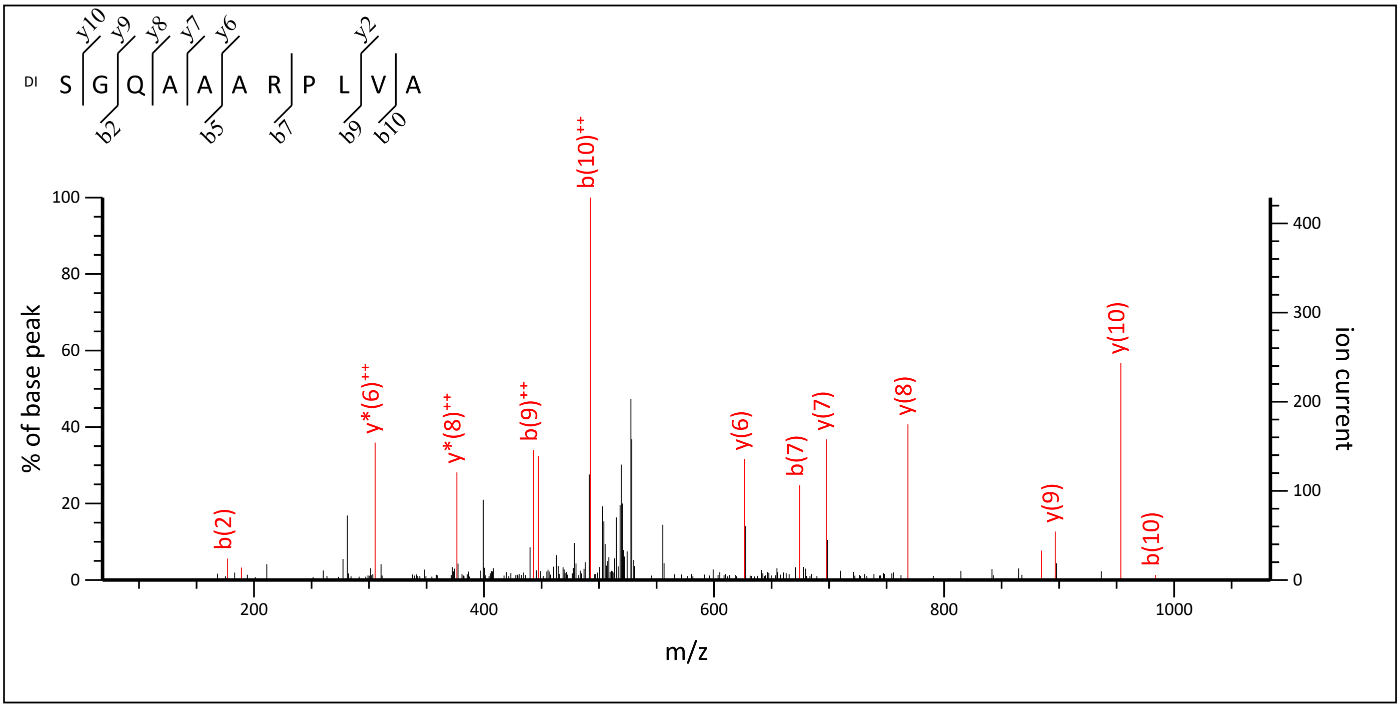

68.29 to 1083.72

Label all possible matches   Label matches used for scoring

Monoisotopic mass of neutral peptide Mr(calc): 1071.6338
Variable modifications:
N-term : Dimethyl:2H(4) (N-term)
Ions Score: 51 **Expect:** 0.21
Matches : 18/88 fragment ions using 25 most intense peaks (help)

#	b	b++	b*	b*++	b0	b0++	Seq.	y	y++	y*	y*++	#
1	120.0957	60.5515			102.0851	51.5462	S					11
2	177.1172	89.0622			159.1066	80.0569	G	953.5527	477.2800	936.5261	468.7667	10
3	305.1758	153.0915	288.1492	144.5782	287.1652	144.0862	Q	896.5312	448.7693	879.5047	440.2560	9
4	376.2129	188.6101	359.1863	180.0968	358.2023	179.6048	A	768.4727	384.7400	751.4461	376.2267	8
5	447.2500	224.1286	430.2234	215.6154	429.2394	215.1233	A	697.4355	349.2214	680.4090	340.7081	7
6	518.2871	259.6472	501.2605	251.1339	500.2765	250.6419	A	626.3984	313.7028	609.3719	305.1896	6
7	674.3882	337.6977	657.3617	329.1845	656.3776	328.6925	R	555.3613	278.1843	538.3348	269.6710	5
8	771.4410	386.2241	754.4144	377.7108	753.4304	377.2188	P	399.2602	200.1337			4
9	884.5250	442.7662	867.4985	434.2529	866.5145	433.7609	L	302.2074	151.6074			3
10	983.5934	492.3004	966.5669	483.7871	965.5829	483.2951	V	189.1234	95.0653			2
11							A	90.0550	45.5311			1

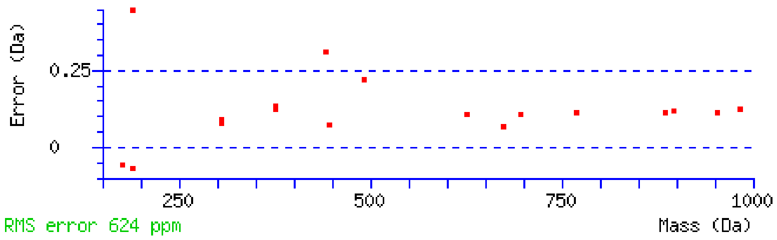

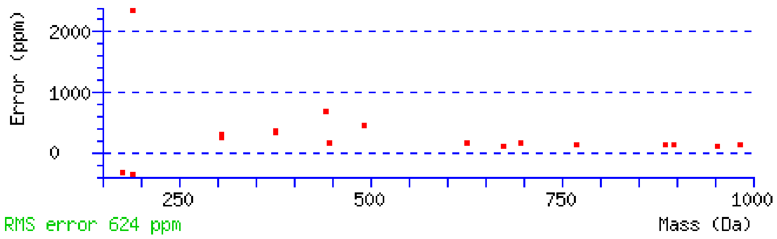

NCBI **BLAST** search of SGQAAARPLVA
(Parameters: blastp, nr protein database, expect=20000, no filter, PAM30)
Other BLAST web gateways

All matches to this query

Score	Mr(calc)	Delta	Sequence
51.5	1071.6338	0.0021	SGQAAARPLVA
31.0	1071.5812	0.0548	DILLTAAQGT
30.2	1071.4509	0.1851	FDNYSAQSA
27.2	1071.6338	0.0021	GGLLRPAAQSA
26.1	1071.5461	0.0898	HYRIGAQGT
25.2	1071.7226	-0.0867	KLLQAAAGASA
25.2	1071.5993	0.0366	YLIGTAAGASA
24.1	1071.5825	0.0535	IYALHRNSA
23.7	1071.6338	0.0021	ARAVPVAGAASA
23.5	1071.5862	0.0497	GVGAVPAAAAGSA

Mascot: http://www.matrixscience.com/

[redacted] Mascot Search Results

Peptide View

MS/MS Fragmentation of **SGQAAARPLVATV**
Found in **UCRI_MOUSE** in **SwissProt**, Cytochrome b-c1 complex subunit Rieske, mitochondrial OS=Mus musculus GN=Uqcrfs1 PE=1 SV=1

Match to Query 3605: 1271.753708 from(636.884130,2+) intensity(369882.6500) scans(5065-5081) rawscans(sn5065:sn5081) rtinseconds(2018.9559-2024.242) index(3073)
Title: 3074: Sum of 2 scans in range 5065 (rt=2018.96) to 5081 (rt=2024.24) [C:\Users\Administrador\Desktop\AMOSTRAS NO ORBITRAP LEANDRO RODAR NOVAMENTE\HIPOCAMPO\hipocampo_01.raw]
Data file hipocampo_01.temp.mgf

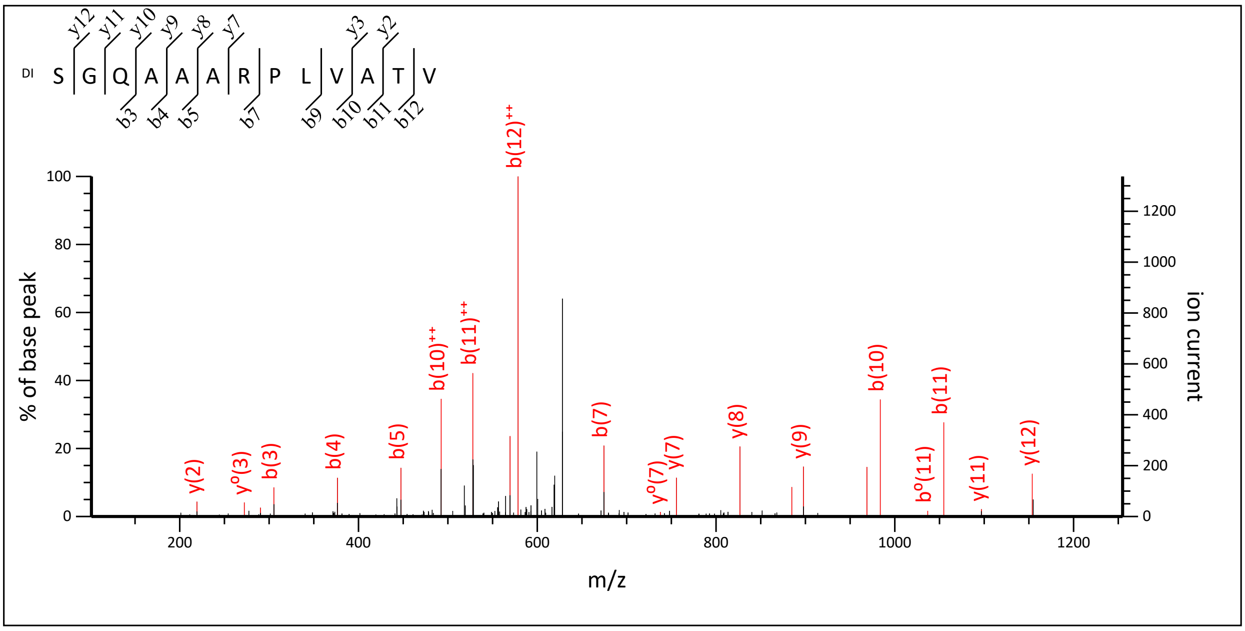

101.21 to 1254.85

Label all possible matches ○ Label matches used for scoring ◉

Monoisotopic mass of neutral peptide Mr(calc): 1271.7499
Variable modifications:
N-term : Dimethyl:2H(4) (N-term)
Ions Score: 83 **Expect:** 0.00014
Matches : 23/126 fragment ions using 29 most intense peaks (help)

#	b	b^{++}	b*	b*++	b^0	b^{0++}	Seq.	y	y^{++}	y*	y*++	y^0	y^{0++}	#
1	120.0957	60.5515			102.0851	51.5462	S							13
2	177.1172	89.0622			159.1066	80.0569	G	***1153.6688***	577.3380	1136.6422	568.8248	1135.6582	568.3327	12
3	***305.1758***	153.0915	288.1492	144.5782	287.1652	144.0862	Q	***1096.6473***	548.8273	1079.6208	540.3140	1078.6368	539.8220	11
4	***376.2129***	188.6101	359.1863	180.0968	358.2023	179.6048	A	***968.5887***	484.7980	951.5622	476.2847	950.5782	475.7927	10
5	***447.2500***	224.1286	430.2234	215.6154	429.2394	215.1233	A	***897.5516***	449.2795	880.5251	440.7662	879.5411	440.2742	9
6	518.2871	259.6472	501.2605	251.1339	500.2765	250.6419	A	***826.5145***	413.7609	809.4880	405.2476	808.5040	404.7556	8
7	***674.3882***	337.6977	657.3617	329.1845	656.3776	328.6925	R	***755.4774***	378.2423	738.4509	369.7291	737.4668	369.2371	7
8	771.4410	386.2241	754.4144	377.7108	753.4304	377.2188	P	599.3763	300.1918			581.3657	291.1865	6
9	***884.5250***	442.7662	867.4985	434.2529	866.5145	433.7609	L	502.3235	251.6654			484.3130	242.6601	5
10	***983.5934***	**492.3004**	966.5669	483.7871	965.5829	483.2951	V	389.2395	195.1234			371.2289	186.1181	4
11	***1054.6306***	**527.8189**	1037.6040	519.3056	1036.6200	518.8136	A	***290.1710***	145.5892			272.1605	136.5839	3
12	1155.6782	**578.3428**	1138.6517	569.8295	1137.6677	569.3375	T	***219.1339***	110.0706			201.1234	101.0653	2
13							V	118.0863	59.5468					1

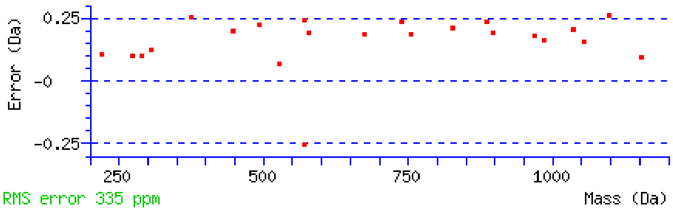

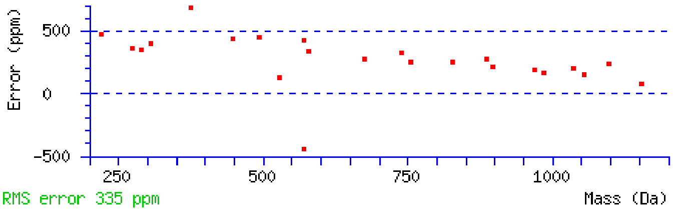

NCBI **BLAST** search of SGQAAARPLVATV
(Parameters: blastp, nr protein database, expect=20000, no filter, PAM30)
Other BLAST web gateways

All matches to this query

Score	Mr(calc)	Delta	Sequence
83.2	1271.7499	0.0038	SGQAAARPLVATV
31.2	1271.6510	0.1027	PHLANGYTTAVT
27.4	1271.6408	0.1129	QELQHSAAAQSA
27.3	1271.5414	0.2123	FEESCMEVATV
26.4	1271.6209	0.1328	MSTSGAAAAAAAGGT
25.9	1271.7247	0.0290	SVQEKTMVLGT
25.1	1271.6469	0.1068	TAAQAAAAAAAAGGTA
24.8	1271.7159	0.0378	VTMINAIPVASL
24.2	1271.8135	-0.0598	EAKRREVLSA
24.1	1271.7903	-0.0366	WSVLRLPVALS

Mascot: http://www.matrixscience.com/

[redacted] Mascot Search Results

Peptide View

MS/MS Fragmentation of TVGLNVPASVRF
Found in UCRI_MOUSE in SwissProt, Cytochrome b-c1 complex subunit Rieske, mitochondrial OS=Mus musculus GN=Uqcrfs1 PE=1 SV=1

Match to Query 3711: 1286.737948 from(644.376250,2+) intensity(47278.7850) scans(9142) rawscans(sn9142) rtinseconds(3433.9542) index(6546)
Title: 6547: Scan 9142 (rt=3433.95) [C:\Users\Administrador\Desktop\AMOSTRAS NO ORBITRAP LEANDRO RODAR NOVAMENTE\HIPOCAMPO\hipocampo_01.raw]
Data file hipocampo_01.temp.mgf

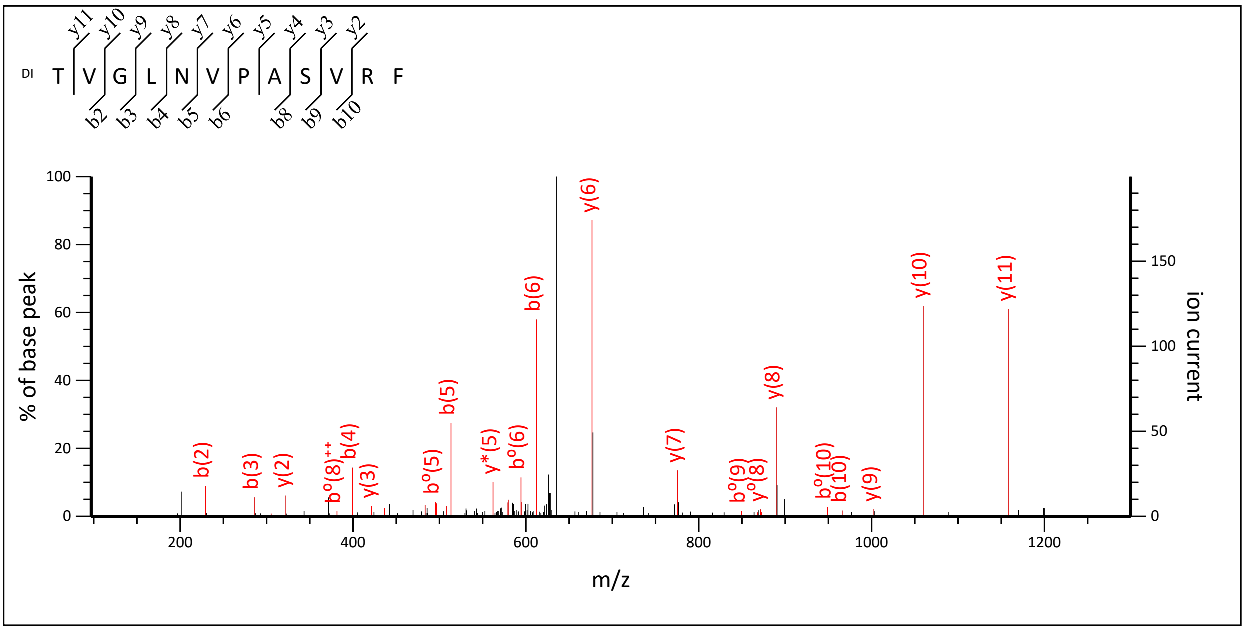

97.07 to 1299.82

Label all possible matches ○ Label matches used for scoring ◉

Monoisotopic mass of neutral peptide Mr(calc): 1286.7347
Variable modifications:
N-term : Dimethyl (N-term)
Ions Score: 55 **Expect:** 0.088
Matches : 34/116 fragment ions using 71 most intense peaks (help)

#	b	b^{++}	b*	b^{*++}	b^0	b^{0++}	Seq.	y	y^{++}	y*	y^{*++}	y^0	y^{0++}	#
1	130.0863	65.5468			112.0757	56.5415	T							12
2	229.1547	115.0810			211.1441	106.0757	V	1158.6630	579.8351	1141.6364	571.3218	1140.6524	570.8298	11
3	286.1761	143.5917			268.1656	134.5864	G	1059.5946	530.3009	1042.5680	521.7876	1041.5840	521.2956	10
4	399.2602	200.1337			381.2496	191.1285	L	1002.5731	501.7902	985.5465	493.2769	984.5625	492.7849	9
5	513.3031	257.1552	496.2766	248.6419	495.2926	248.1499	N	889.4890	445.2482	872.4625	436.7349	871.4785	436.2429	8
6	612.3715	306.6894	595.3450	298.1761	594.3610	297.6841	V	775.4461	388.2267	758.4196	379.7134	757.4355	379.2214	7
7	709.4243	355.2158	692.3978	346.7025	691.4137	346.2105	P	676.3777	338.6925	659.3511	330.1792	658.3671	329.6872	6
8	780.4614	390.7343	763.4349	382.2211	762.4509	381.7291	A	579.3249	290.1661	562.2984	281.6528	561.3144	281.1608	5
9	867.4934	434.2504	850.4669	425.7371	849.4829	425.2451	S	508.2878	254.6475	491.2613	246.1343	490.2772	245.6423	4
10	966.5619	483.7846	949.5353	475.2713	948.5513	474.7793	V	421.2558	211.1315	404.2292	202.6183			3
11	1122.6630	561.8351	1105.6364	553.3218	1104.6524	552.8298	R	322.1874	161.5973	305.1608	153.0840			2
12							F	166.0863	83.5468					1

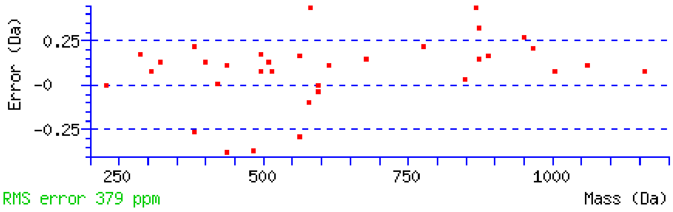

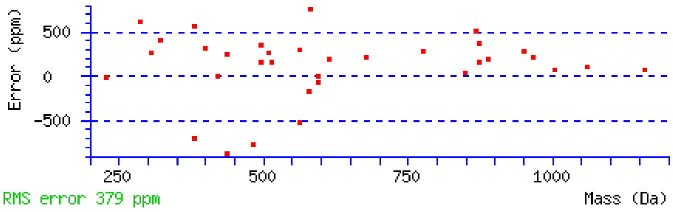

NCBI **BLAST** search of TVGLNVPASVRF
(Parameters: blastp, nr protein database, expect=20000, no filter, PAM30)
Other BLAST web gateways

All matches to this query

Score	Mr(calc)	Delta	Sequence
55.3	1286.7347	0.0033	TVGLNVPASVRF
23.7	1286.8351	-0.0972	PVVSLSKNGKY
22.4	1286.8761	-0.1382	KLQHPVARGLP
21.9	1286.7235	0.0145	GISQVVFVSNPI
21.9	1286.5965	0.1415	SAGARVFEYMA
19.8	1286.7995	-0.0616	KSCVWVNGLPL
19.3	1286.7347	0.0033	KTLNFDGRLPA
19.3	1286.7950	-0.0571	LPRNKEPWIA
18.6	1286.7234	0.0145	VSAQKNYLGPI
18.5	1286.7598	-0.0219	TVKQGQTLFIP

Mascot: http://www.matrixscience.com/

[redacted] Mascot Search Results

Peptide View

MS/MS Fragmentation of **GISTPEELGLDKV**
Found in **COX5A_MOUSE** in **SwissProt**, Cytochrome c oxidase subunit 5A, mitochondrial OS=Mus musculus GN=Cox5a PE=1 SV=2

Match to Query 4478: 1412.778728 from(707.396640,2+) intensity(151348.6600) scans(8237) rawscans(sn8237) rtinseconds(3114.5524) index(5837)
Title: 5838: Scan 8237 (rt=3114.55) [C:\Users\Administrador\Desktop\AMOSTRAS NO ORBITRAP LEANDRO RODAR NOVAMENTE\HIPOCAMPO\hipocampo_01.raw]
Data file hipocampo_01.temp.mgf

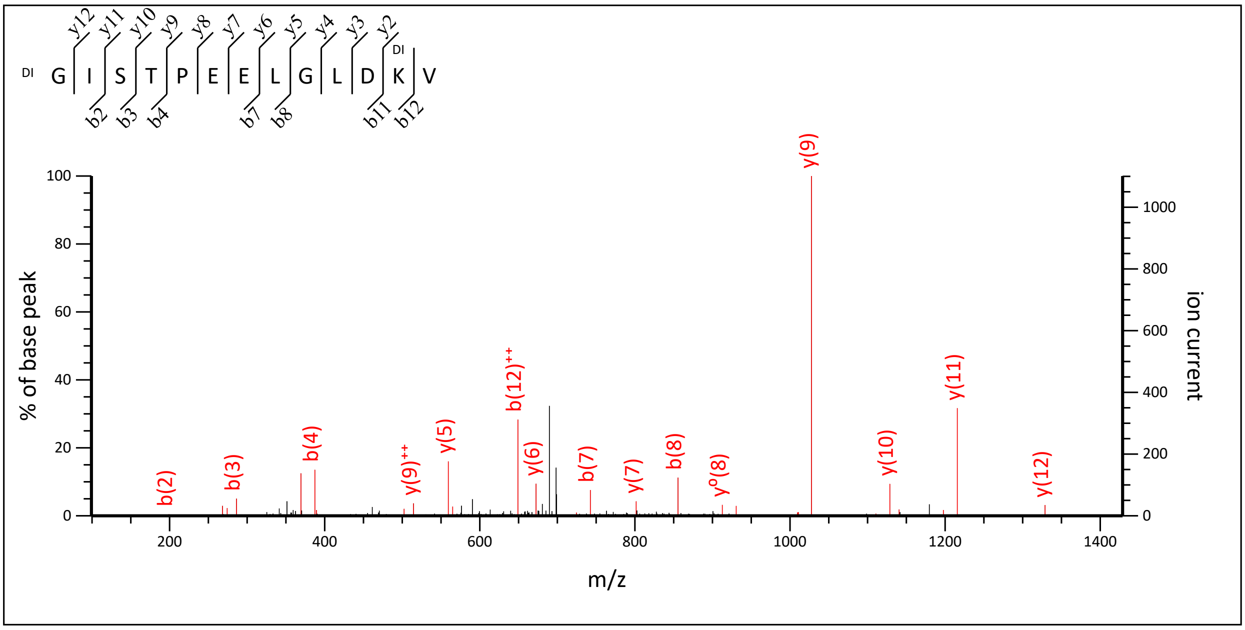

99.21 to 1428.86

Label all possible matches ○ Label matches used for scoring ◉

```
Monoisotopic mass of neutral peptide Mr(calc): 1412.7763
Variable modifications:
N-term : Dimethyl (N-term)
K12    : Dimethyl (K)
Ions Score: 66  Expect: 0.007
Matches : 29/112 fragment ions using 60 most intense peaks   (help)
```

#	b	b++	b*	b*++	b0	b0++	Seq.	y	y++	y*	y*++	y0	y0++	#
1	86.0600	43.5337					G							13
2	199.1441	100.0757					I	1328.7308	664.8690	1311.7042	656.3558	1310.7202	655.8637	12
3	286.1761	143.5917			268.1656	134.5864	S	1215.6467	608.3270	1198.6202	599.8137	1197.6361	599.3217	11
4	387.2238	194.1155			369.2132	185.1103	T	1128.6147	564.8110	1111.5881	556.2977	1110.6041	555.8057	10
5	484.2766	242.6419			466.2660	233.6366	P	1027.5670	514.2871	1010.5405	505.7739	1009.5564	505.2819	9
6	613.3192	307.1632			595.3086	298.1579	E	930.5142	465.7608	913.4877	457.2475	912.5037	456.7555	8
7	742.3618	371.6845			724.3512	362.6792	E	801.4716	401.2395	784.4451	392.7262	783.4611	392.2342	7
8	855.4458	428.2266			837.4353	419.2213	L	672.4291	336.7182	655.4025	328.2049	654.4185	327.7129	6
9	912.4673	456.7373			894.4567	447.7320	G	559.3450	280.1761	542.3184	271.6629	541.3344	271.1709	5
10	1025.5514	513.2793			1007.5408	504.2740	L	502.3235	251.6654	485.2970	243.1521	484.3130	242.6601	4
11	1140.5783	570.7928			1122.5677	561.7875	D	389.2395	195.1234	372.2129	186.6101	371.2289	186.1181	3
12	1296.7046	648.8559	1279.6780	640.3426	1278.6940	639.8506	K	274.2125	137.6099	257.1860	129.0966			2
13							V	118.0863	59.5468					1

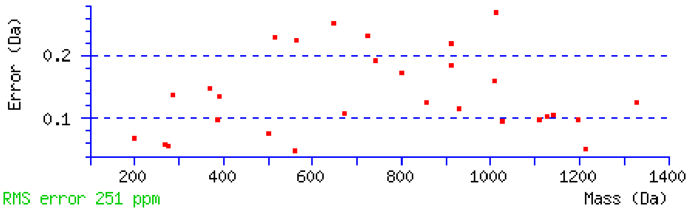

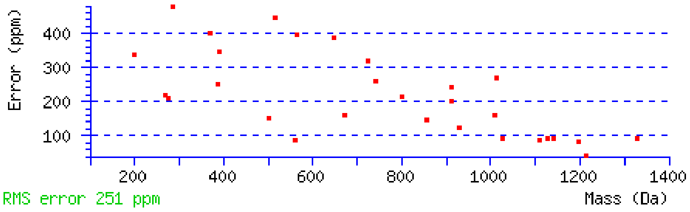

NCBI **BLAST** search of GISTPEELGLDKV
(Parameters: blastp, nr protein database, expect=20000, no filter, PAM30)
Other BLAST web gateways

All matches to this query

Score	Mr(calc)	Delta	Sequence
66.4	1412.7763	0.0025	GISTPEELGLDKV
32.0	1412.8429	-0.0641	SPGLDGLLDLLLGV
30.3	1412.8279	-0.0492	AVSTPAGLITPIVF
25.7	1412.7925	-0.0138	RPTPVNSATAALPS
25.0	1412.7623	0.0164	LGTSGRAALGSVEPA
24.9	1412.8239	-0.0451	GLSGLLKLDLSQN
24.1	1412.7875	-0.0088	QSGSEGKVKPDK
24.0	1412.6857	0.0930	GIECQEELPAPSL
23.6	1412.6969	0.0818	QETLELIQHMQ
23.6	1412.7299	0.0488	TNTATLLRYYQ

Mascot: http://www.matrixscience.com/

Mascot Search Results

Peptide View

MS/MS Fragmentation of NDFASAVRILEV
Found in COX5A_MOUSE in SwissProt, Cytochrome c oxidase subunit 5A, mitochondrial OS=Mus musculus GN=Cox5a PE=1 SV=2

Match to Query 4207: 1360.736888 from(681.375720,2+) intensity(31020.1310) scans(11202) rawscans(sn11202) rtinseconds(4184.7178) index(8301)
Title: 8302: Scan 11202 (rt=4184.72) [C:\Users\Administrador\Desktop\AMOSTRAS NO ORBITRAP LEANDRO RODAR NOVAMENTE\HIPOCAMPO\hipocampo_01.raw]
Data file hipocampo_01.temp.mgf

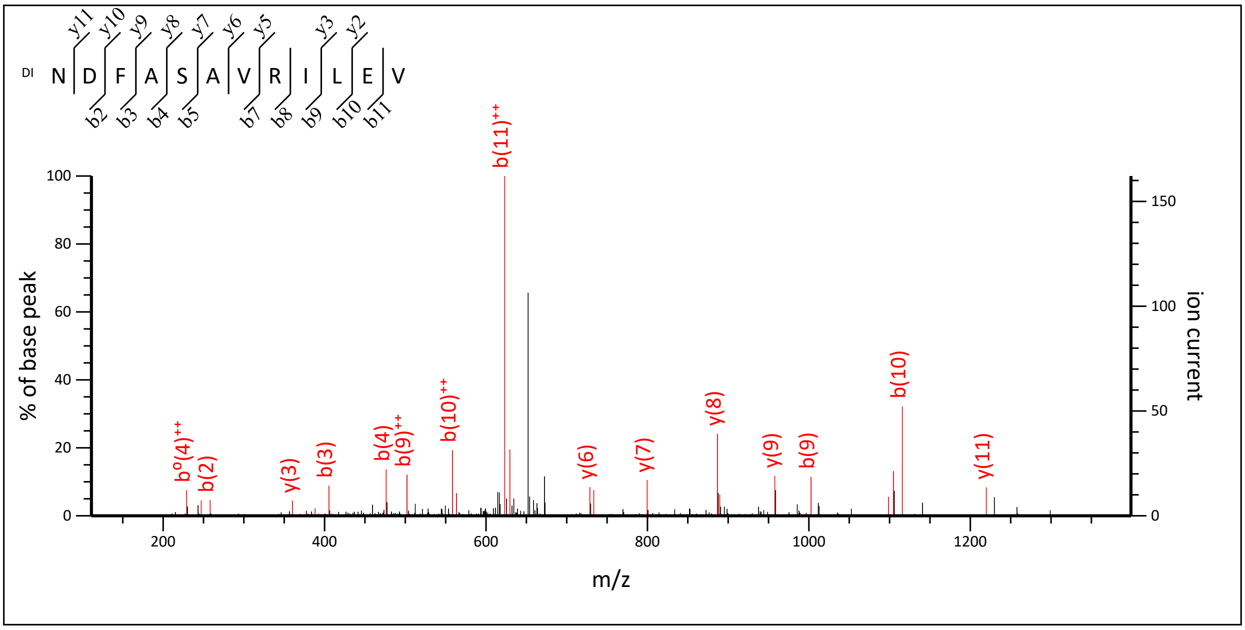

111.14 to 1399.02

Label all possible matches ○ Label matches used for scoring ◉

Monoisotopic mass of neutral peptide Mr(calc): 1360.7350
Variable modifications:
N-term : Dimethyl (N-term)
Ions Score: 70 **Expect:** 0.0027
Matches : 24/120 fragment ions using 33 most intense peaks (help)

#	b	b^{++}	b*	b^{*++}	b^0	b^{0++}	Seq.	y	y^{++}	y*	y^{*++}	y^0	y^{0++}	#
1	143.0815	72.0444	126.0550	63.5311			N							12
2	258.1084	129.5579	241.0819	121.0446	240.0979	120.5526	D	1219.6681	610.3377	1202.6416	601.8244	1201.6575	601.3324	11
3	405.1769	203.0921	388.1503	194.5788	387.1663	194.0868	F	1104.6412	552.8242	1087.6146	544.3109	1086.6306	543.8189	10
4	476.2140	238.6106	459.1874	230.0974	458.2034	229.6053	A	957.5728	479.2900	940.5462	470.7767	939.5622	470.2847	9
5	563.2460	282.1266	546.2195	273.6134	545.2354	273.1214	S	886.5356	443.7715	869.5091	435.2582	868.5251	434.7662	8
6	634.2831	317.6452	617.2566	309.1319	616.2726	308.6399	A	799.5036	400.2554	782.4771	391.7422	781.4931	391.2502	7
7	733.3515	367.1794	716.3250	358.6661	715.3410	358.1741	V	728.4665	364.7369	711.4400	356.2236	710.4559	355.7316	6
8	889.4526	445.2300	872.4261	436.7167	871.4421	436.2247	R	629.3981	315.2027	612.3715	306.6894	611.3875	306.1974	5
9	1002.5367	501.7720	985.5102	493.2587	984.5261	492.7667	I	473.2970	237.1521			455.2864	228.1468	4
10	1115.6208	558.3140	1098.5942	549.8007	1097.6102	549.3087	L	360.2129	180.6101			342.2023	171.6048	3
11	1244.6634	622.8353	1227.6368	614.3220	1226.6528	613.8300	E	247.1288	124.0681			229.1183	115.0628	2
12							V	118.0863	59.5468					1

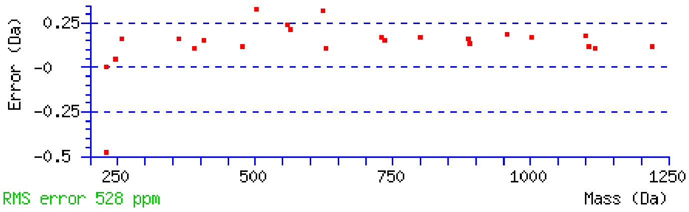

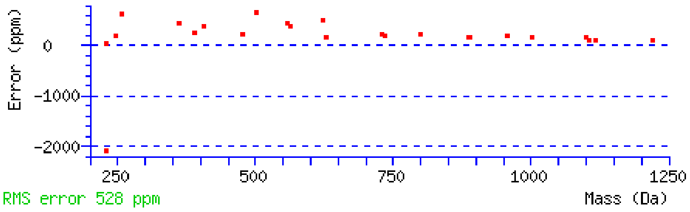

NCBI **BLAST** search of NDFASAVRILEV
(Parameters: blastp, nr protein database, expect=20000, no filter, PAM30)
Other BLAST web gateways

All matches to this query

Score	Mr(calc)	Delta	Sequence
70.5	1360.7350	0.0018	NDFASAVRILEV
31.9	1360.8417	-0.1048	GPETVLQSAIKL
27.8	1360.5825	0.1544	NMMASIQSFSAM
26.2	1360.7322	0.0047	ATLAPTTRAMAPA
26.1	1360.8417	-0.1048	KDNIGVAVVLEV
23.8	1360.7380	-0.0011	VINAAPGAQGGTEV
23.8	1360.6519	0.0849	NPGPMVVFATPGM
23.0	1360.6408	0.0960	SQDASSRPDPEV
22.6	1360.8166	-0.0797	APGVQKALGAGTEV
22.4	1360.6097	0.1272	TLDEMDSAASDL

Mascot: http://www.matrixscience.com/

[redacted] Mascot Search Results

Peptide View

MS/MS Fragmentation of **RPTLNELGISTPEELGLDKV**
Found in **COX5A_MOUSE** in **SwissProt**, Cytochrome c oxidase subunit 5A, mitochondrial OS=Mus musculus GN=Cox5a PE=1 SV=2

Match to Query 8645: 2244.298632 from(749.106820,3+) intensity(31536.9790) scans(10331) rawscans(sn10331) rtinseconds(3866.6548) index(7546)
Title: 7547: Scan 10331 (rt=3866.65) [C:\Users\Administrador\Desktop\AMOSTRAS NO ORBITRAP LEANDRO RODAR NOVAMENTE\HIPOCAMPO\hipocampo_01.raw]
Data file hipocampo_01.temp.mgf

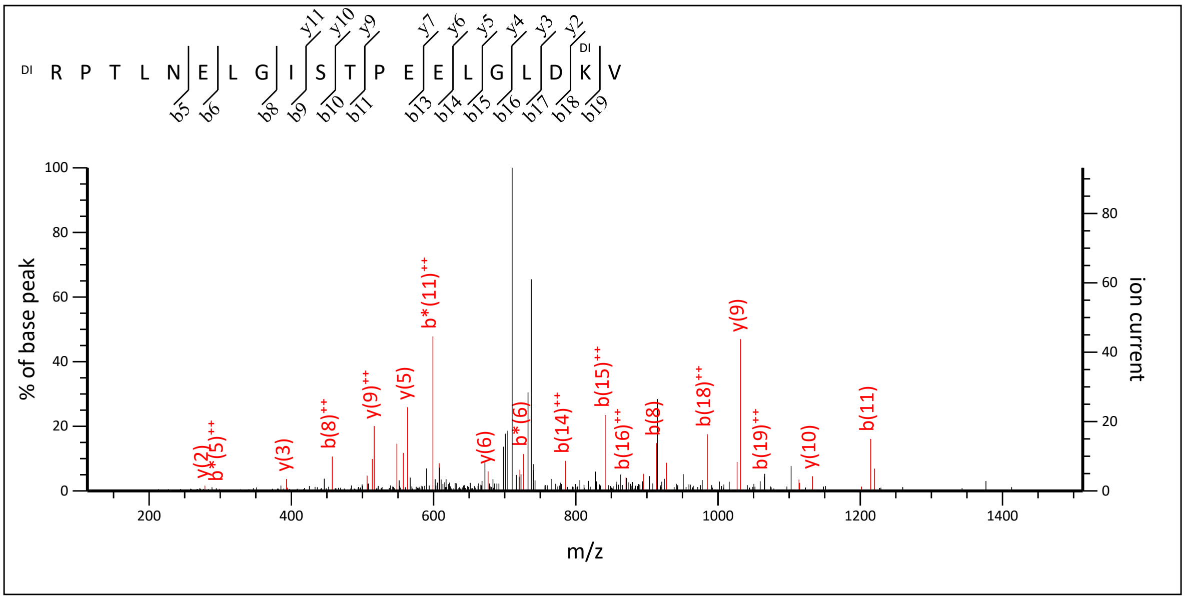

113.22 to 1512.86

Label all possible matches ○ Label matches used for scoring ◉

```
Monoisotopic mass of neutral peptide Mr(calc): 2244.2817
Variable modifications:
N-term : Dimethyl:2H(4) (N-term)
K19    : Dimethyl:2H(4) (K)
Ions Score: 42  Expect: 2.3
Matches : 38/218 fragment ions using 78 most intense peaks   (help)
```

#	b	b^{++}	b*	b^{*++}	b^0	b^{0++}	Seq.	y	y^{++}	y*	y^{*++}	y^0	y^{0++}	#
1	189.1648	95.0860	172.1382	86.5728			R							20
2	286.2176	143.6124	269.1910	135.0991			P	2057.1314	1029.0694	2040.1049	1020.5561	2039.1209	1020.0641	19
3	387.2652	194.1363	370.2387	185.6230	369.2547	185.1310	T	1960.0787	980.5430	1943.0521	972.0297	1942.0681	971.5377	18
4	500.3493	250.6783	483.3228	242.1650	482.3387	241.6730	L	1859.0310	930.0191	1842.0044	921.5059	1841.0204	921.0139	17
5	614.3922	307.6998	597.3657	299.1865	596.3817	298.6945	N	1745.9469	873.4771	1728.9204	864.9638	1727.9364	864.4718	16
6	743.4348	372.2210	726.4083	363.7078	725.4243	363.2158	E	1631.9040	816.4556	1614.8775	807.9424	1613.8934	807.4504	15
7	856.5189	428.7631	839.4923	420.2498	838.5083	419.7578	L	1502.8614	751.9343	1485.8349	743.4211	1484.8508	742.9291	14
8	**913.5403**	***457.2738***	896.5138	448.7605	895.5298	448.2685	G	1389.7773	695.3923	1372.7508	686.8790	1371.7668	686.3870	13
9	**1026.6244**	***513.8158***	1009.5979	505.3026	1008.6138	504.8106	I	1332.7559	666.8816	1315.7293	658.3683	1314.7453	657.8763	12
10	**1113.6564**	***557.3319***	1096.6299	548.8186	1095.6459	548.3266	S	**1219.6718**	610.3395	1202.6453	601.8263	1201.6613	601.3343	11
11	**1214.7041**	***607.8557***	1197.6776	599.3424	1196.6936	598.8504	T	**1132.6398**	566.8235	1115.6132	558.3103	1114.6292	557.8183	10
12	1311.7569	656.3821	1294.7303	647.8688	1293.7463	647.3768	P	**1031.5921**	516.2997	1014.5656	507.7864	1013.5815	507.2944	9
13	1440.7995	***720.9034***	1423.7729	712.3901	1422.7889	711.8981	E	934.5393	467.7733	917.5128	459.2600	916.5288	458.7680	8
14	1569.8421	***785.4247***	1552.8155	776.9114	1551.8315	776.4194	E	805.4968	403.2520	788.4702	394.7387	787.4862	394.2467	7
15	1682.9261	***841.9667***	1665.8996	833.4534	1664.9156	832.9614	L	**676.4542**	338.7307	659.4276	330.2174	658.4436	329.7254	6
16	1739.9476	***870.4774***	1722.9210	861.9642	1721.9370	861.4722	G	**563.3701**	282.1887	546.3435	273.6754	545.3595	273.1834	5
17	1853.0317	***927.0195***	1836.0051	918.5062	1835.0211	918.0142	L	**506.3486**	253.6780	489.3221	245.1647	488.3381	244.6727	4
18	1968.0586	***984.5329***	1951.0321	976.0197	1950.0480	975.5277	D	**393.2646**	197.1359	376.2380	188.6226	375.2540	188.1306	3
19	2128.2100	***1064.6086***	2111.1834	1056.0954	2110.1994	1055.6033	K	**278.2376**	139.6225	261.2111	131.1092			2
20							V	118.0863	59.5468					1

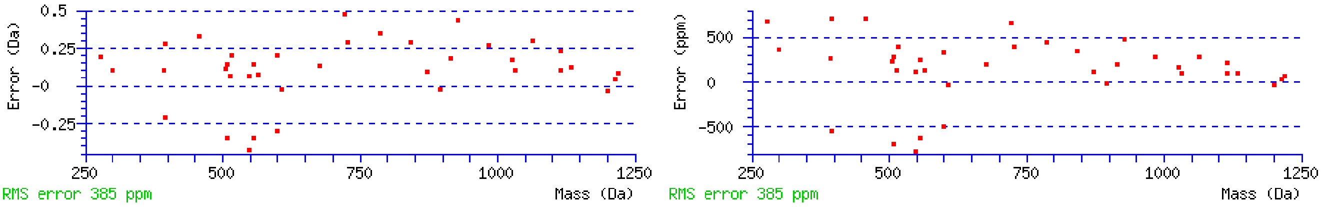

NCBI **BLAST** search of RPTLNELGISTPEELGLDKV
(Parameters: blastp, nr protein database, expect=20000, no filter, PAM30)
Other BLAST web gateways

All matches to this query

Score	Mr(calc)	Delta	Sequence
41.6	2244.2817	0.0170	RPTLNELGISTPEELGLDKV
18.0	2244.3293	-0.0307	LSAKGQLTNIVSPTAATTPRI
17.3	2243.9274	0.3712	MMESHSSNSNGTEMVSQPSH
15.6	2244.1007	0.1979	AENGHQMAAETAIQRPNVPH
15.1	2244.1517	0.1469	ARGFGLWEGALYEIGGLFGTG
14.6	2244.1462	0.1524	EDTMYPLIALVVIFFGMSL
14.1	2244.2676	0.0310	SLDATPSPETTSLQTKKMTI
13.6	2244.0580	0.2406	VCPGIAADSPSSTSDSPQNKEV
12.8	2244.0220	0.2766	LMSPMTSGEMSMPLMKTTPS
12.4	2244.0775	0.2211	LVHGLTLVGTASNPCGVMMQM

Mascot: http://www.matrixscience.com/

[redacted] Mascot Search Results

Peptide View

MS/MS Fragmentation of **FHNPHVNPLPTGYEDE**
Found in **CX6A1_MOUSE** in **SwissProt**, Cytochrome c oxidase subunit 6A1, mitochondrial OS=Mus musculus GN=Cox6a1 PE=1 SV=2

Match to Query 7128: 1892.874028 from(947.444290,2+) intensity(60708.2460) scans(5788) rawscans(sn5788) rtinseconds(2254.0558) index(3711)
Title: 3712: Scan 5788 (rt=2254.06) [C:\Users\Administrador\Desktop\AMOSTRAS NO ORBITRAP LEANDRO RODAR NOVAMENTE\HIPOCAMPO\hipocampo_01.raw]
Data file hipocampo_01.temp.mgf

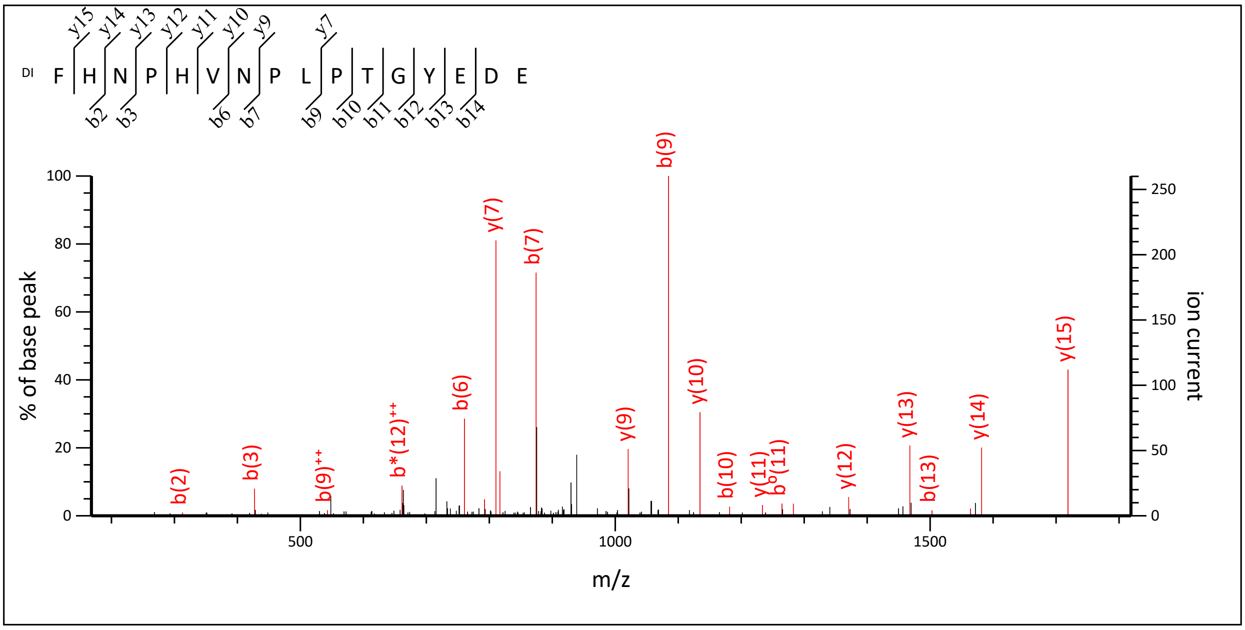

168.2 to 1818.91

Label all possible matches ○ Label matches used for scoring ◉

```
Monoisotopic mass of neutral peptide Mr(calc): 1892.8693
Variable modifications:
N-term : Dimethyl (N-term)
Ions Score: 55  Expect: 0.11
Matches : 25/138 fragment ions using 42 most intense peaks   (help)
```

#	b	b^{++}	b*	b^{*++}	b^0	b^{0++}	Seq.	y	y^{++}	y*	y^{*++}	y^0	y^{0++}	#
1	176.1070	88.5571					F							16
2	*313.1659*	157.0866					H	*1718.7769*	859.8921	1701.7503	851.3788	1700.7663	850.8868	15
3	*427.2088*	214.1081	410.1823	205.5948			N	*1581.7180*	791.3626	1564.6914	782.8494	1563.7074	782.3573	14
4	524.2616	262.6344	507.2350	254.1212			P	*1467.6751*	734.3412	1450.6485	725.8279	1449.6645	725.3359	13
5	*661.3205*	331.1639	644.2940	322.6506			H	*1370.6223*	685.8148	1353.5957	677.3015	1352.6117	676.8095	12
6	*760.3889*	380.6981	743.3624	372.1848			V	*1233.5634*	617.2853	1216.5368	608.7721	1215.5528	608.2800	11
7	*874.4318*	437.7196	857.4053	429.2063			N	*1134.4950*	567.7511	1117.4684	559.2378	1116.4844	558.7458	10
8	971.4846	486.2459	954.4581	477.7327			P	*1020.4520*	510.7297			1002.4415	501.7244	9
9	*1084.5687*	**542.7880**	1067.5421	534.2747			L	923.3993	462.2033			905.3887	453.1980	8
10	*1181.6214*	591.3144	1164.5949	582.8011			P	*810.3152*	405.6612			792.3046	396.6560	7
11	*1282.6691*	641.8382	1265.6426	633.3249	1264.6586	632.8329	T	713.2624	357.1349			695.2519	348.1296	6
12	1339.6906	670.3489	1322.6640	661.8357	1321.6800	661.3436	G	612.2148	306.6110			594.2042	297.6057	5
13	*1502.7539*	751.8806	1485.7274	743.3673	1484.7433	742.8753	Y	555.1933	278.1003			537.1827	269.0950	4
14	1631.7965	**816.4019**	1614.7700	807.8886	1613.7859	807.3966	E	392.1300	196.5686			374.1194	187.5633	3
15	1746.8234	**873.9154**	1729.7969	865.4021	1728.8129	864.9101	D	263.0874	132.0473			245.0768	123.0420	2
16							E	148.0604	74.5339			130.0499	65.5286	1

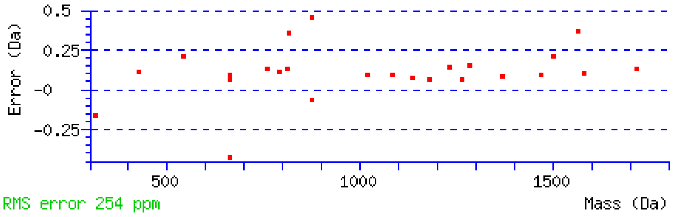

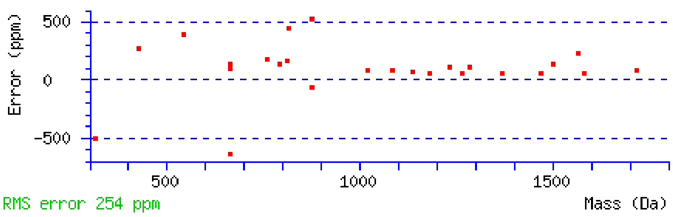

NCBI **BLAST** search of FHNPHVNPLPTGYEDE
(Parameters: blastp, nr protein database, expect=20000, no filter, PAM30)
Other BLAST web gateways

All matches to this query

Score	Mr(calc)	Delta	Sequence
54.7	1892.8693	0.0047	FHNPHVNPLPTGYEDE
16.7	1893.0683	-0.1943	LREIQELGQLIQAVQQ
15.7	1892.8102	0.0638	NEFYGLNFTCGGSNTSM
15.1	1892.9576	-0.0835	TEREIVNDFREKMM
14.9	1892.7746	0.0994	NLMFGDYMNPDLEGDD
14.6	1892.8549	0.0191	EATHCTPLPAQHFQMP
14.1	1893.1335	-0.2594	HVVLRRINNVHVKMD
13.7	1892.7246	0.1494	SEDEDEPDMKCDDMM
13.5	1893.1222	-0.2482	KSMHLGRLPSRPPLPGS
13.5	1892.7828	0.0912	AGALAQDEYEEDSSDEE

Mascot: http://www.matrixscience.com/

[redacted] Mascot Search Results

Peptide View

MS/MS Fragmentation of **HNPHVNPLPTGYEDE**
Found in **CX6A1_MOUSE** in **SwissProt**, Cytochrome c oxidase subunit 6A1, mitochondrial OS=Mus musculus GN=Cox6a1 PE=1 SV=2

Match to Query 6391: 1745.804808 from(873.909680,2+) intensity(84828.5230) scans(4888) rawscans(sn4888) rtinseconds(1960.3945) index(2914)
Title: 2915: Scan 4888 (rt=1960.39) [C:\Users\Administrador\Desktop\AMOSTRAS NO ORBITRAP LEANDRO RODAR NOVAMENTE\HIPOCAMPO\hipocampo_01.raw]
Data file hipocampo_01.temp.mgf

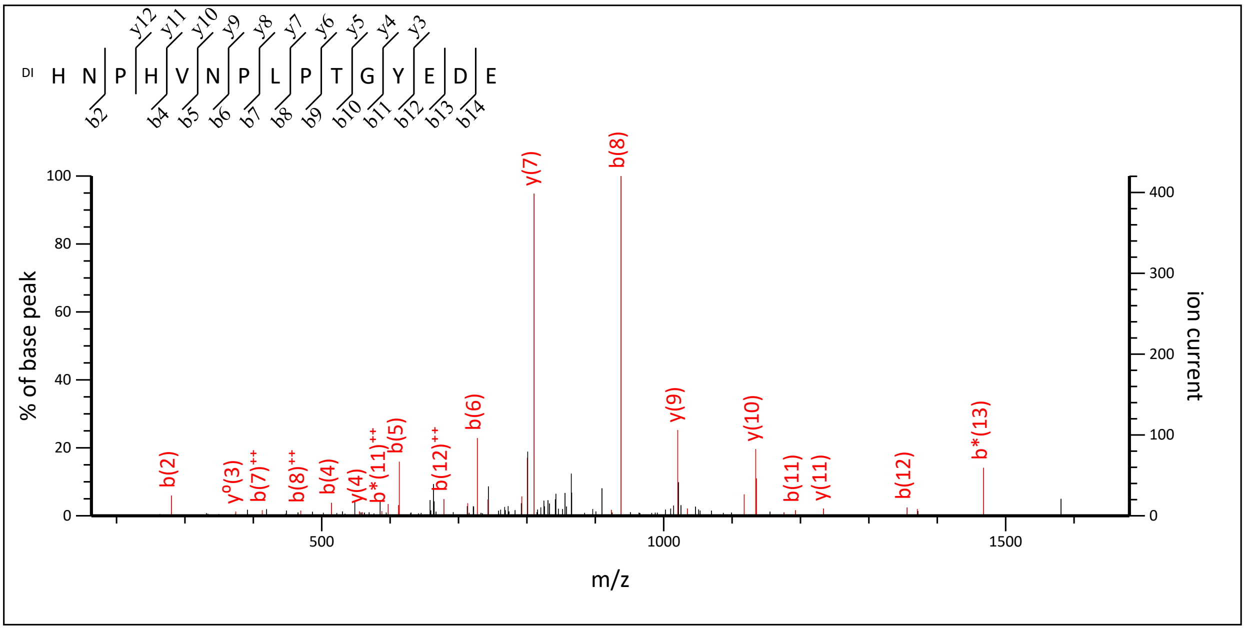

163.27 to 1680.94

Label all possible matches ○ Label matches used for scoring ◉

Monoisotopic mass of neutral peptide Mr(calc): 1745.8009
Variable modifications:
N-term : Dimethyl (N-term)
Ions Score: 61 **Expect:** 0.023
Matches : 38/130 fragment ions using 69 most intense peaks (help)

#	b	b^{++}	b^{*}	b^{*++}	b^{0}	b^{0++}	Seq.	y	y^{++}	y^{*}	y^{*++}	y^{0}	y^{0++}	#
1	166.0975	83.5524					H							15
2	280.1404	140.5738	263.1139	132.0606			N	1581.7180	791.3626	1564.6914	782.8494	1563.7074	782.3573	14
3	377.1932	189.1002	360.1666	180.5870			P	1467.6751	734.3412	1450.6485	725.8279	1449.6645	725.3359	13
4	514.2521	257.6297	497.2255	249.1164			H	1370.6223	685.8148	1353.5957	677.3015	1352.6117	676.8095	12
5	613.3205	307.1639	596.2940	298.6506			V	1233.5634	617.2853	1216.5368	608.7721	1215.5528	608.2800	11
6	727.3634	364.1854	710.3369	355.6721			N	1134.4950	567.7511	1117.4684	559.2378	1116.4844	558.7458	10
7	824.4162	412.7117	807.3896	404.1985			P	1020.4520	510.7297			1002.4415	501.7244	9
8	937.5003	469.2538	920.4737	460.7405			L	923.3993	462.2033			905.3887	453.1980	8
9	1034.5530	517.7802	1017.5265	509.2669			P	810.3152	405.6612			792.3046	396.6560	7
10	1135.6007	568.3040	1118.5742	559.7907	1117.5901	559.2987	T	713.2624	357.1349			695.2519	348.1296	6
11	1192.6222	596.8147	1175.5956	588.3014	1174.6116	587.8094	G	612.2148	306.6110			594.2042	297.6057	5
12	1355.6855	678.3464	1338.6589	669.8331	1337.6749	669.3411	Y	555.1933	278.1003			537.1827	269.0950	4
13	1484.7281	742.8677	1467.7015	734.3544	1466.7175	733.8624	E	392.1300	196.5686			374.1194	187.5633	3
14	1599.7550	800.3812	1582.7285	791.8679	1581.7445	791.3759	D	263.0874	132.0473			245.0768	123.0420	2
15							E	148.0604	74.5339			130.0499	65.5286	1

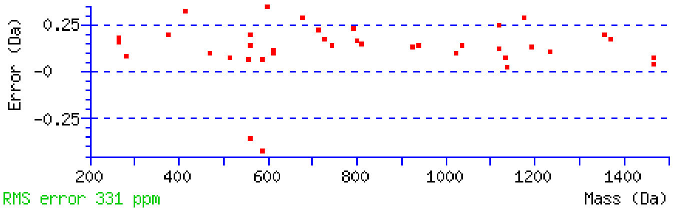

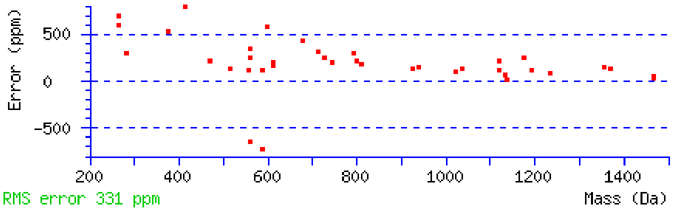

NCBI **BLAST** search of HNPHVNPLPTGYEDE
(Parameters: blastp, nr protein database, expect=20000, no filter, PAM30)
Other BLAST web gateways

All matches to this query

Score	Mr(calc)	Delta	Sequence
61.4	1745.8009	0.0039	HNPHVNPLPTGYEDE
20.5	1745.9098	-0.1050	IGLHGGRPLQDMVGMGG
17.4	1745.8723	-0.0675	KTGIDNPIFDTEEGI
15.8	1745.9272	-0.1224	QAKYGDRHAMKQSD
14.8	1746.0254	-0.2206	SHRMFDSTAKSFPK
14.2	1745.8680	-0.0632	IFEVGKNGGSSCPECR
14.1	1745.7171	0.0877	DGMHQNLPPMERDM
13.8	1745.8266	-0.0218	TMSEAELPLGFSRMN
13.8	1745.9109	-0.1061	LDASDEFLKERVMN
13.5	1745.8869	-0.0821	GKQLTRLSLMEEDE

Mascot: http://www.matrixscience.com/

Mascot Search Results

Peptide View

MS/MS Fragmentation of **NPHVNPLPTGYEDE**
Found in **CX6A1_MOUSE** in **SwissProt**, Cytochrome c oxidase subunit 6A1, mitochondrial OS=Mus musculus GN=Cox6a1 PE=1 SV=2

Match to Query 5578: 1608.746608 from(805.380580,2+) intensity(42224.8050) scans(5737) rawscans(sn5737) rtinseconds(2237.191) index(3665)
Title: 3666: Scan 5737 (rt=2237.19) [C:\Users\Administrador\Desktop\AMOSTRAS NO ORBITRAP LEANDRO RODAR NOVAMENTE\HIPOCAMPO\hipocampo_01.raw]
Data file hipocampo_01.temp.mgf

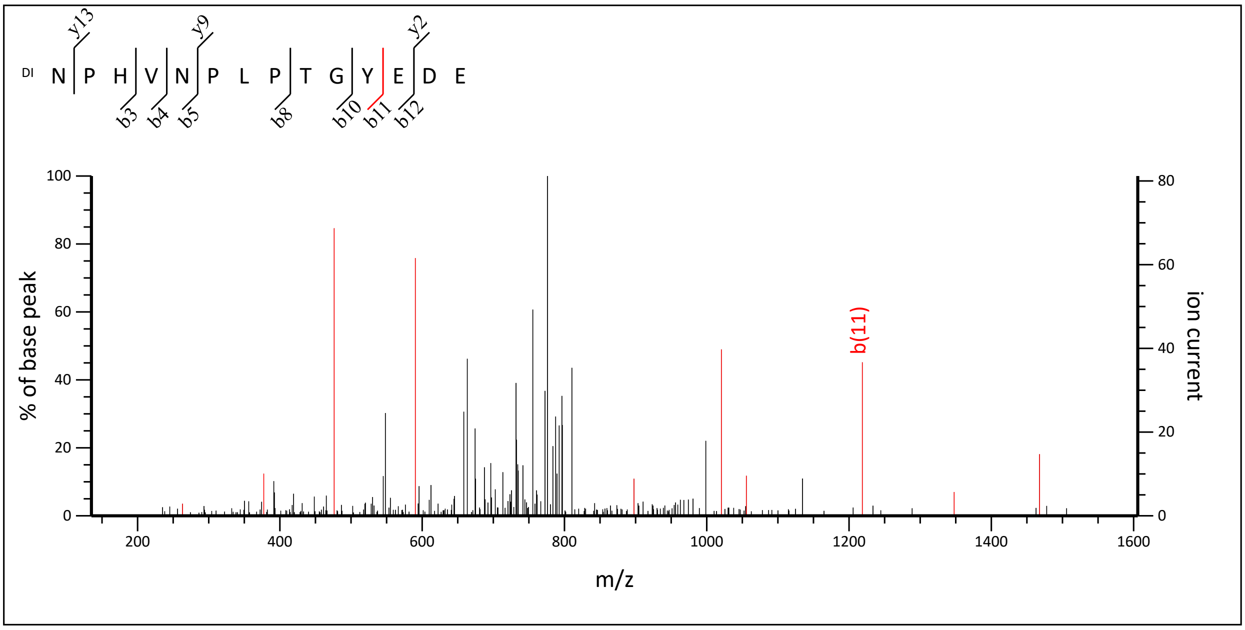

135.11 to 1605.82

Label all possible matches ○ Label matches used for scoring ◉

```
Monoisotopic mass of neutral peptide Mr(calc): 1608.7420
Variable modifications:
N-term : Dimethyl (N-term)
Ions Score: 53  Expect: 0.17
Matches : 10/122 fragment ions using 13 most intense peaks   (help)
```

#	b	b^{++}	b*	b^{*++}	b^0	b^{0++}	Seq.	y	y^{++}	y*	y^{*++}	y^0	y^{0++}	#
1	143.0815	72.0444	126.0550	63.5311			N							14
2	240.1343	120.5708	223.1077	112.0575			P	**1467.6751**	734.3412	1450.6485	725.8279	1449.6645	725.3359	13
3	***377.1932***	189.1002	360.1666	180.5870			H	1370.6223	685.8148	1353.5957	677.3015	1352.6117	676.8095	12
4	***476.2616***	238.6344	459.2350	230.1212			V	1233.5634	617.2853	1216.5368	608.7721	1215.5528	608.2800	11
5	***590.3045***	295.6559	573.2780	287.1426			N	1134.4950	567.7511	1117.4684	559.2378	1116.4844	558.7458	10
6	687.3573	344.1823	670.3307	335.6690			P	**1020.4520**	510.7297			1002.4415	501.7244	9
7	800.4413	400.7243	783.4148	392.2110			L	923.3993	462.2033			905.3887	453.1980	8
8	***897.4941***	449.2507	880.4676	440.7374			P	810.3152	405.6612			792.3046	396.6560	7
9	998.5418	499.7745	981.5152	491.2613	980.5312	490.7693	T	713.2624	357.1349			695.2519	348.1296	6
10	***1055.5633***	528.2853	1038.5367	519.7720	1037.5527	519.2800	G	612.2148	306.6110			594.2042	297.6057	5
11	***1218.6266***	609.8169	1201.6000	601.3037	1200.6160	600.8116	Y	555.1933	278.1003			537.1827	269.0950	4
12	***1347.6692***	674.3382	1330.6426	665.8250	1329.6586	665.3329	E	392.1300	196.5686			374.1194	187.5633	3
13	1462.6961	731.8517	1445.6696	723.3384	1444.6856	722.8464	D	**263.0874**	132.0473			245.0768	123.0420	2
14							E	148.0604	74.5339			130.0499	65.5286	1

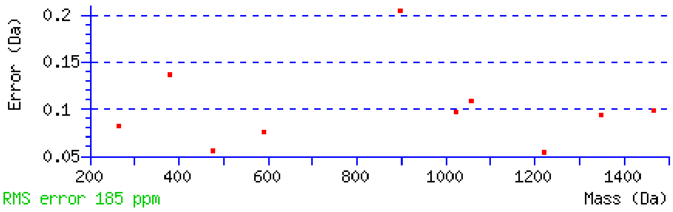

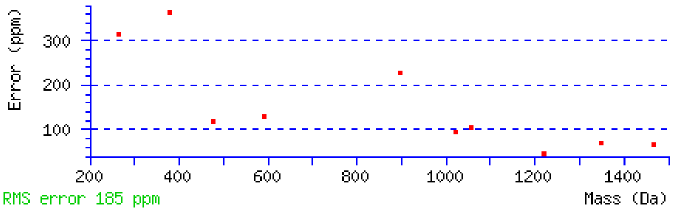

NCBI **BLAST** search of NPHVNPLPTGYEDE
(Parameters: blastp, nr protein database, expect=20000, no filter, PAM30)
Other BLAST web gateways

All matches to this query

Score	Mr(calc)	Delta	Sequence
52.7	1608.7420	0.0046	NPHVNPLPTGYEDE
21.8	1608.8388	-0.0922	LIERGASLEEVNDE
20.9	1608.8275	-0.0809	ALERDEVETLIDE
19.1	1608.7885	-0.0419	EPGGRGGGASTRVEED
18.9	1608.7197	0.0269	SMDAPVMIMNGKDE
18.0	1608.8589	-0.1123	EVSSIFPLTLGEPF
17.3	1608.8235	-0.0769	NTSVSSKSTDPHFP
17.3	1608.5923	0.1543	TSCSYPSEDSSEDE
16.1	1608.9483	-0.2017	PPTLRETKPVNMP
13.9	1608.9169	-0.1703	LLEYRAGLVAGHGFA

Mascot: http://www.matrixscience.com/

Mascot Search Results

Peptide View

MS/MS Fragmentation of **AGNSDLILPVPAFNVINGGSHAGNKL**
Found in **ENOG_MOUSE** in **SwissProt**, Gamma-enolase OS=Mus musculus GN=Eno2 PE=1 SV=2

Match to Query 9603: 2630.429982 from(877.817270,3+) intensity(22324.3420) scans(11509) rawscans(sn11509) rtinseconds(4300.4458) index(8562)
Title: 8563: Scan 11509 (rt=4300.45) [C:\Users\Administrador\Desktop\AMOSTRAS NO ORBITRAP LEANDRO RODAR NOVAMENTE\HIPOCAMPO\hipocampo_01.raw]
Data file hipocampo_01.temp.mgf

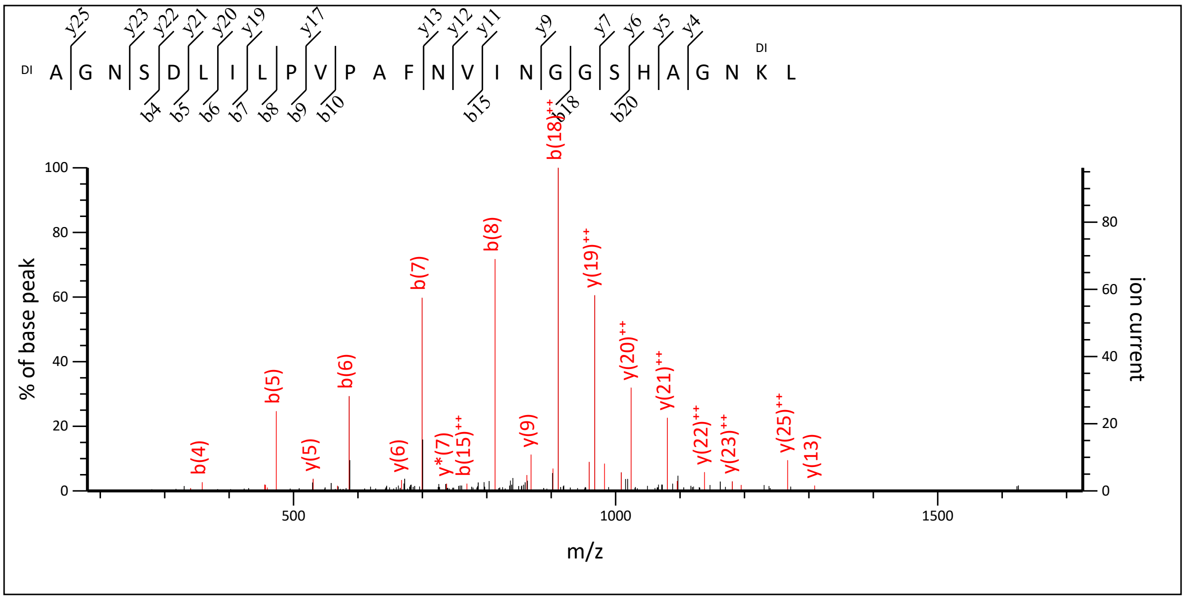

180.03 to 1725.12

Label all possible matches ○ Label matches used for scoring ◉

```
Monoisotopic mass of neutral peptide Mr(calc): 2630.4180
Variable modifications:
N-term : Dimethyl (N-term)
K25    : Dimethyl (K)
Ions Score: 81  Expect: 0.00026
Matches : 34/276 fragment ions using 43 most intense peaks   (help)
```

#	b	b^{++}	b*	b^{*++}	b^0	b^{0++}	Seq.	y	y^{++}	y*	y^{*++}	y^0	y^{0++}	#
1	100.0757	50.5415					A							26
2	157.0972	79.0522					G	2532.3569	***1266.6821***	2515.3303	1258.1688	2514.3463	1257.6768	25
3	271.1401	136.0737	254.1135	127.5604			N	2475.3354	1238.1714	2458.3089	1229.6581	2457.3249	1229.1661	24
4	**358.1721**	179.5897	341.1456	171.0764	340.1615	170.5844	S	2361.2925	***1181.1499***	2344.2660	1172.6366	2343.2819	1172.1446	23
5	**473.1991**	237.1032	456.1725	228.5899	455.1885	228.0979	D	2274.2605	***1137.6339***	2257.2339	1129.1206	2256.2499	1128.6286	22
6	**586.2831**	293.6452	569.2566	285.1319	568.2726	284.6399	L	2159.2335	***1080.1204***	2142.2070	1071.6071	2141.2230	1071.1151	21
7	**699.3672**	350.1872	682.3406	341.6740	681.3566	341.1819	I	2046.1495	***1023.5784***	2029.1229	1015.0651	2028.1389	1014.5731	20
8	**812.4512**	406.7293	795.4247	398.2160	794.4407	397.7240	L	1933.0654	***967.0363***	1916.0389	958.5231	1915.0548	958.0311	19
9	909.5040	**455.2556**	892.4775	446.7424	891.4934	446.2504	P	1819.9813	***910.4943***	1802.9548	901.9810	1801.9708	901.4890	18
10	**1008.5724**	504.7898	991.5459	496.2766	990.5619	495.7846	V	1722.9286	***861.9679***	1705.9020	853.4547	1704.9180	852.9626	17
11	1105.6252	553.3162	1088.5986	544.8030	1087.6146	544.3109	P	1623.8602	***812.4337***	1606.8336	803.9204	1605.8496	803.4284	16
12	1176.6623	588.8348	1159.6358	580.3215	1158.6517	579.8295	A	1526.8074	763.9073	1509.7809	755.3941	1508.7968	754.9021	15
13	1323.7307	662.3690	1306.7042	653.8557	1305.7201	653.3637	F	1455.7703	728.3888	1438.7437	719.8755	1437.7597	719.3835	14
14	1437.7736	719.3905	1420.7471	710.8772	1419.7631	710.3852	N	***1308.7019***	654.8546	1291.6753	646.3413	1290.6913	645.8493	13
15	1536.8421	**768.9247**	1519.8155	760.4114	1518.8315	759.9194	V	***1194.6589***	597.8331	1177.6324	589.3198	1176.6484	588.8278	12
16	1649.9261	825.4667	1632.8996	816.9534	1631.9156	816.4614	I	***1095.5905***	548.2989	1078.5640	539.7856	1077.5800	539.2936	11
17	1763.9690	882.4882	1746.9425	873.9749	1745.9585	873.4829	N	***982.5065***	491.7569	965.4799	483.2436	964.4959	482.7516	10
18	1820.9905	**910.9989**	1803.9640	902.4856	1802.9799	901.9936	G	***868.4635***	434.7354	851.4370	426.2221	850.4530	425.7301	9
19	1878.0120	939.5096	1860.9854	930.9964	1860.0014	930.5043	G	811.4421	406.2247	794.4155	397.7114	793.4315	397.2194	8
20	1965.0440	**983.0256**	1948.0175	974.5124	1947.0334	974.0204	S	754.4206	377.7139	737.3941	369.2007	736.4100	368.7087	7
21	2102.1029	1051.5551	2085.0764	1043.0418	2084.0923	1042.5498	H	***667.3886***	334.1979	650.3620	325.6847			6

22	2173.1400	1087.0737	2156.1135	1078.5604	2155.1295	1078.0684	A	***530.3297***	265.6685	513.3031	257.1552			5
23	2230.1615	1115.5844	2213.1349	1107.0711	2212.1509	1106.5791	G	***459.2926***	230.1499	442.2660	221.6366			4
24	2344.2044	1172.6058	2327.1779	1164.0926	2326.1939	1163.6006	N	402.2711	201.6392	385.2445	193.1259			3
25	2500.3307	1250.6690	2483.3041	1242.1557	2482.3201	1241.6637	K	288.2282	144.6177	271.2016	136.1044			2
26							L	132.1019	66.5546					1

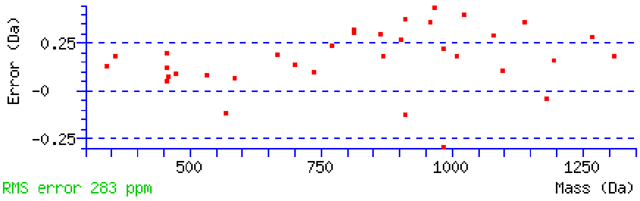

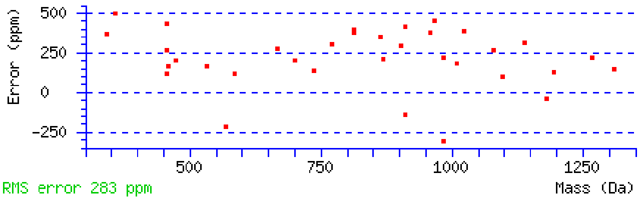

NCBI **BLAST** search of AGNSDLILPVPAFNVINGGSHAGNKL
(Parameters: blastp, nr protein database, expect=20000, no filter, PAM30)
Other BLAST web gateways

All matches to this query

Score	Mr(calc)	Delta	Sequence
81.3	2630.4180	0.0120	AGNSDLILPVPAFNVINGGSHAGNKL
37.0	2630.4796	-0.0496	STSLLGQKKPGYHAPVALLNDIPQ
27.7	2630.5195	-0.0895	MLLALILSMAVPTIIGLSLWKEF
27.7	2630.6287	-0.1987	PVTFIILLNIIFLVITLCKMVK
26.4	2630.4427	-0.0127	AIEMLDGHLYLLLDMGSGTIKIK
25.6	2630.3262	0.1038	MNVTVEGPTSILQNYQLILHTSE
25.3	2630.4359	-0.0059	GTYDIFYTAAKPGTYVIYVRFGG
24.0	2630.3151	0.1148	APPGALNLGLSSNNSTLSVAGAMGSHGG
23.9	2630.5411	-0.1111	RQLQQLVLDPGFLGLEPLLDLLL
23.1	2630.5197	-0.0897	LTVLIPPEETRIDGGPVILLQAGTP

Mascot: http://www.matrixscience.com/

[redacted] Mascot Search Results

Peptide View

MS/MS Fragmentation of AFRVPTPNVSVVDL
Found in G3P_MOUSE in SwissProt, Glyceraldehyde-3-phosphate dehydrogenase OS=Mus musculus GN=Gapdh PE=1 SV=2

Match to Query 5231: 1540.873148 from(771.443850,2+) intensity(23204.3850) scans(10746) rawscans(sn10746) rtinseconds(4016.6452) index(7908)
Title: 7909: Scan 10746 (rt=4016.65) [C:\Users\Administrador\Desktop\AMOSTRAS NO ORBITRAP LEANDRO RODAR NOVAMENTE\HIPOCAMPO\hipocampo_01.raw]
Data file hipocampo_01.temp.mgf

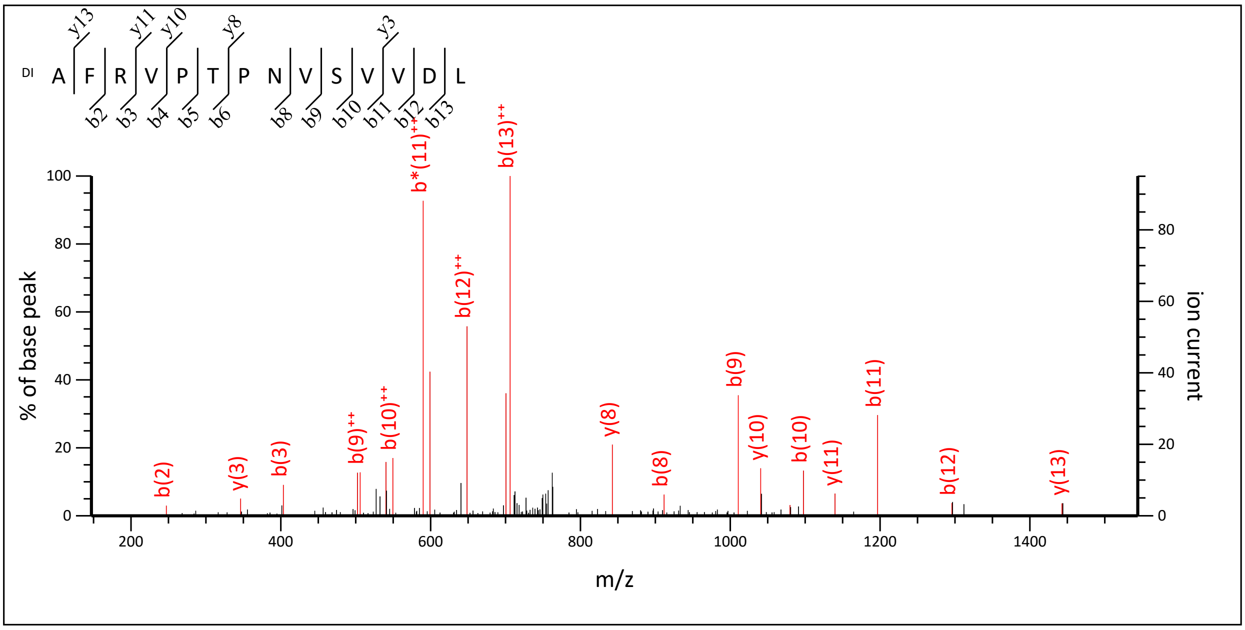

147.15 to 1544.02

Label all possible matches ○ Label matches used for scoring ◉

Monoisotopic mass of neutral peptide Mr(calc): 1540.8613
Variable modifications:
N-term : Dimethyl (N-term)
Ions Score: 79 **Expect:** 0.00041
Matches : 28/128 fragment ions using 34 most intense peaks (help)

#	b	b^{++}	b*	b^{*++}	b^0	b^{0++}	Seq.	y	y^{++}	y*	y^{*++}	y^0	y^{0++}	#
1	100.0757	50.5415					A							14
2	247.1441	124.0757					F	1442.8002	721.9037	1425.7736	713.3905	1424.7896	712.8985	13
3	403.2452	202.1262	386.2187	193.6130			R	1295.7318	648.3695	1278.7052	639.8563	1277.7212	639.3642	12
4	502.3136	251.6605	485.2871	243.1472			V	1139.6307	570.3190	1122.6041	561.8057	1121.6201	561.3137	11
5	599.3664	300.1868	582.3398	291.6736			P	1040.5623	520.7848	1023.5357	512.2715	1022.5517	511.7795	10
6	700.4141	350.7107	683.3875	342.1974	682.4035	341.7054	T	943.5095	472.2584	926.4829	463.7451	925.4989	463.2531	9
7	797.4668	399.2371	780.4403	390.7238	779.4563	390.2318	P	842.4618	421.7345	825.4353	413.2213	824.4512	412.7293	8
8	911.5098	456.2585	894.4832	447.7452	893.4992	447.2532	N	745.4090	373.2082	728.3825	364.6949	727.3985	364.2029	7
9	1010.5782	505.7927	993.5516	497.2795	992.5676	496.7874	V	631.3661	316.1867			613.3556	307.1814	6
10	1097.6102	549.3087	1080.5837	540.7955	1079.5996	540.3035	S	532.2977	266.6525			514.2871	257.6472	5
11	1196.6786	598.8429	1179.6521	590.3297	1178.6681	589.8377	V	445.2657	223.1365			427.2551	214.1312	4
12	1295.7470	648.3772	1278.7205	639.8639	1277.7365	639.3719	V	346.1973	173.6023			328.1867	164.5970	3
13	1410.7740	705.8906	1393.7474	697.3774	1392.7634	696.8853	D	247.1288	124.0681			229.1183	115.0628	2
14							L	132.1019	66.5546					1

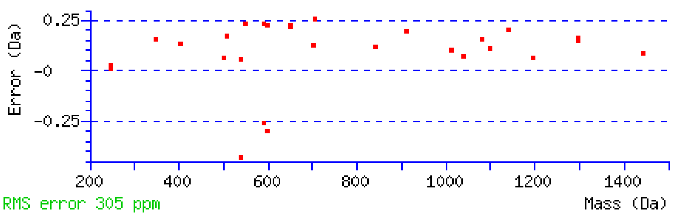

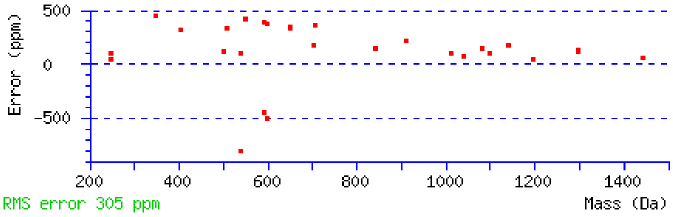

NCBI **BLAST** search of AFRVPTPNVSVVDL
(Parameters: blastp, nr protein database, expect=20000, no filter, PAM30)
Other BLAST web gateways

All matches to this query

Score	Mr(calc)	Delta	Sequence
78.8	1540.8613	0.0118	AFRVPTPNVSVVDL
30.7	1540.9287	-0.0555	SIVTGGEGKPGVVDL
30.4	1540.8763	-0.0031	QLSVPLGQLREEV
25.4	1540.7973	0.0758	DLHHAKDSEGVDI
24.4	1540.7820	0.0911	QFVSLQTQHPRM
24.1	1540.8622	0.0109	ALKTQGSSMSVVDL
23.8	1540.6939	0.1793	MLDMAENEGVVDI
23.8	1540.7415	0.1317	MLDMAEREGVVDI
23.7	1540.9127	-0.0395	ILSVVDLNPRGVDL
23.0	1540.9127	-0.0395	LTIHQSAQVSVLLT

Mascot: http://www.matrixscience.com/

[redacted] Mascot Search Results

Peptide View

MS/MS Fragmentation of **TQVVSDAAYKGVQPHVV**
Found in **LNEBL_MOUSE** in **SwissProt**, LIM zinc-binding domain-containing Nebulette OS=Mus musculus GN=Nebl PE=1 SV=1

Match to Query 6921: 1853.007548 from(927.511050,2+) intensity(29419.5640) scans(5889) rawscans(sn5889) rtinseconds(2287.5838) index(3802)
Title: 3803: Scan 5889 (rt=2287.58) [C:\Users\Administrador\Desktop\AMOSTRAS NO ORBITRAP LEANDRO RODAR NOVAMENTE\HIPOCAMPO\hipocampo_01.raw]
Data file hipocampo_01.temp.mgf

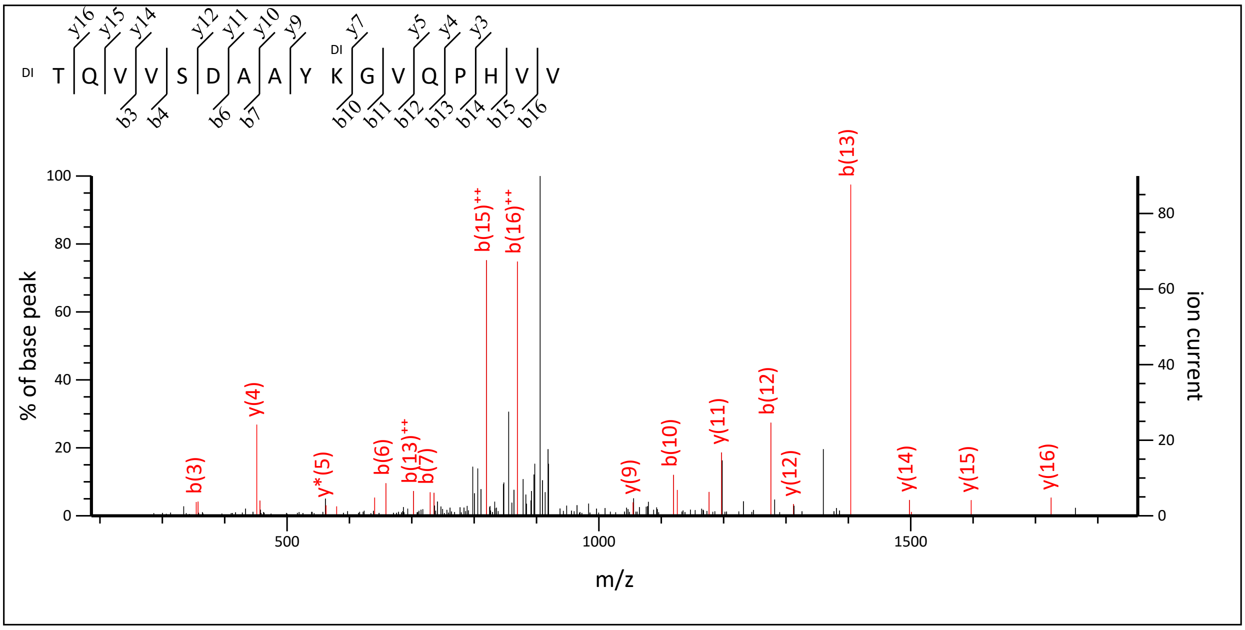

186.31 to 1864.04

Label all possible matches ○ Label matches used for scoring ◉

```
Monoisotopic mass of neutral peptide Mr(calc): 1853.0047
Variable modifications:
N-term : Dimethyl (N-term)
K10    : Dimethyl (K)
Ions Score: 83  Expect: 0.00018
Matches : 26/160 fragment ions using 39 most intense peaks   (help)
```

#	b	b++	b*	b*++	b0	b0++	Seq.	y	y++	y*	y*++	y0	y0++	#
1	130.0863	65.5468			112.0757	56.5415	T							17
2	258.1448	129.5761	241.1183	121.0628	240.1343	120.5708	Q	***1724.9330***	862.9701	1707.9064	854.4569	1706.9224	853.9649	16
3	***357.2132***	179.1103	340.1867	170.5970	339.2027	170.1050	V	***1596.8744***	798.9408	1579.8479	790.4276	1578.8639	789.9356	15
4	***456.2817***	228.6445	439.2551	220.1312	438.2711	219.6392	V	***1497.8060***	749.4066	1480.7795	740.8934	1479.7954	740.4014	14
5	543.3137	272.1605	526.2871	263.6472	525.3031	263.1552	S	1398.7376	699.8724	1381.7110	691.3592	1380.7270	690.8672	13
6	***658.3406***	329.6740	641.3141	321.1607	640.3301	320.6687	D	***1311.7056***	656.3564	1294.6790	647.8431	1293.6950	647.3511	12
7	***729.3777***	365.1925	712.3512	356.6792	711.3672	356.1872	A	***1196.6786***	598.8429	1179.6521	590.3297			11
8	800.4149	400.7111	783.3883	392.1978	782.4043	391.7058	A	***1125.6415***	563.3244	1108.6150	554.8111			10
9	963.4782	482.2427	946.4516	473.7295	945.4676	473.2375	Y	***1054.6044***	527.8058	1037.5778	519.2926			9
10	***1119.6045***	560.3059	1102.5779	551.7926	1101.5939	551.3006	K	891.5411	446.2742	874.5145	437.7609			8
11	***1176.6259***	588.8166	1159.5994	580.3033	1158.6154	579.8113	G	***735.4148***	368.2110	718.3883	359.6978			7
12	***1275.6943***	638.3508	1258.6678	629.8375	1257.6838	629.3455	V	678.3933	339.7003	661.3668	331.1870			6
13	***1403.7529***	**702.3801**	1386.7264	693.8668	1385.7423	693.3748	Q	***579.3249***	290.1661	562.2984	281.6528			5
14	***1500.8057***	750.9065	1483.7791	742.3932	1482.7951	741.9012	P	***451.2663***	226.1368					4
15	1637.8646	**819.4359**	1620.8380	810.9227	1619.8540	810.4306	H	***354.2136***	177.6104					3
16	1736.9330	**868.9701**	1719.9064	860.4569	1718.9224	859.9649	V	217.1547	109.0810					2
17							V	118.0863	59.5468					1

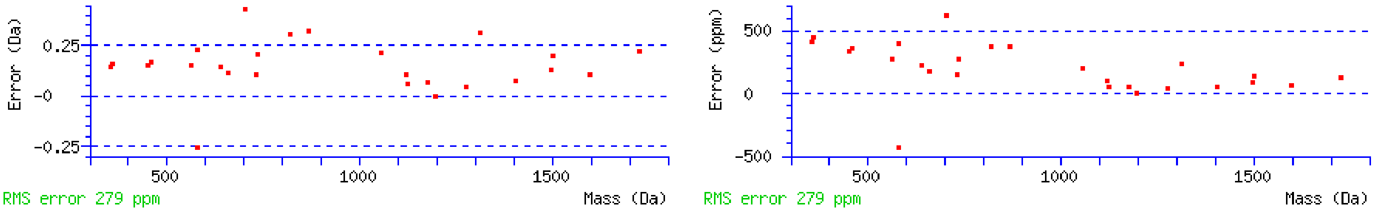

NCBI **BLAST** search of TQVVSDAAYKGVQPHVV
(Parameters: blastp, nr protein database, expect=20000, no filter, PAM30)
Other BLAST web gateways

All matches to this query

Score	Mr(calc)	Delta	Sequence
82.6	1853.0047	0.0028	TQVVSDAAYKGVQPHVV
17.8	1852.9472	0.0603	QTVFTIHPSHVQPAYT
17.5	1852.8426	0.1649	AGPVGEDMFHWQATIM
16.4	1853.0168	-0.0093	MDTADGVPVNSRVSTKI
15.0	1852.9117	0.0959	VPPFFACSRQPCPHVV
13.8	1852.8535	0.1540	YCRFHKCLSVGMSHN
12.2	1852.9291	0.0785	LAHPASSNLDPSMQGLPA
11.4	1853.0633	-0.0558	LSVRSVFFGVFQSFVV
11.3	1852.9046	0.1029	PSHPPPPYPFGSQQASQ
11.3	1852.9829	0.0247	LGGMRTPAVKAPQERE

Mascot: http://www.matrixscience.com/

[redacted] Mascot Search Results

Peptide View

MS/MS Fragmentation of AQATGKPAQYIA
Found in MIF_MOUSE in SwissProt, Macrophage migration inhibitory factor OS=Mus musculus GN=Mif PE=1 SV=2

Match to Query 3619: 1273.707348 from(637.860950,2+) intensity(114164.7300) scans(3846) rawscans(sn3846) rtinseconds(1624.2138) index(2033)
Title: 2034: Scan 3846 (rt=1624.21) [C:\Users\Administrador\Desktop\AMOSTRAS NO ORBITRAP LEANDRO RODAR NOVAMENTE\HIPOCAMPO\hipocampo_01.raw]
Data file hipocampo_01.temp.mgf

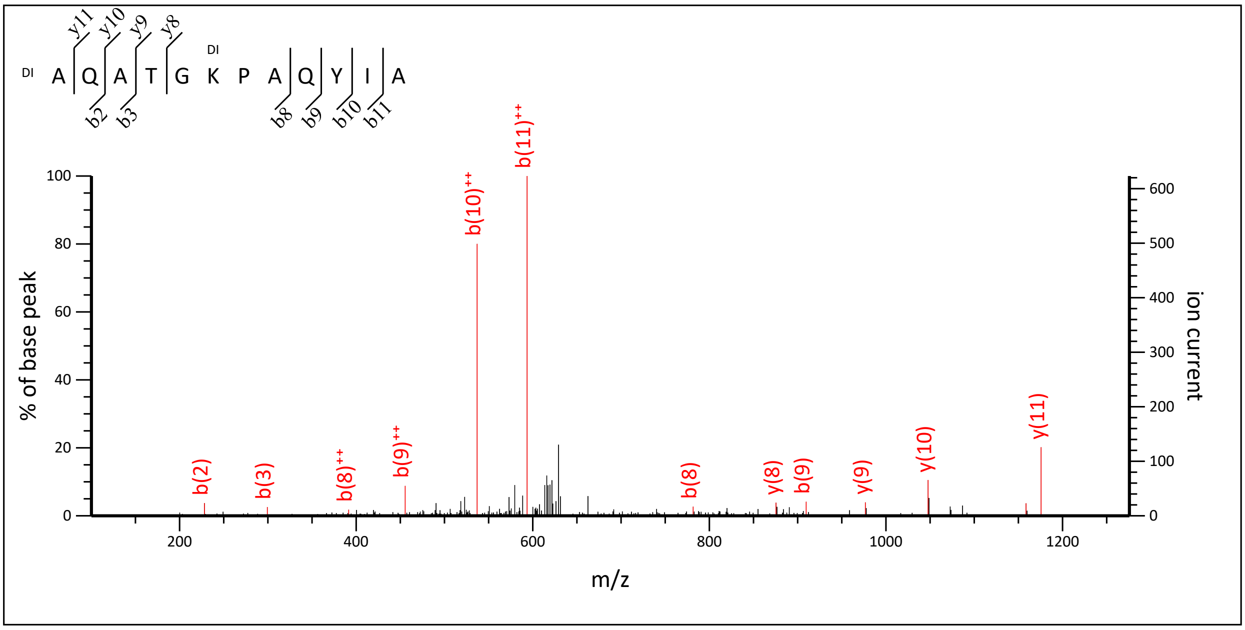

100.15 to 1275.74

Label all possible matches ○ Label matches used for scoring ◉

```
Monoisotopic mass of neutral peptide Mr(calc): 1273.7030
Variable modifications:
N-term : Dimethyl (N-term)
K6     : Dimethyl (K)
Ions Score: 39  Expect: 3.4
Matches : 13/102 fragment ions using 20 most intense peaks   (help)
```

#	b	b^{++}	b^{*}	b^{*++}	b^{0}	b^{0++}	Seq.	y	y^{++}	y^{*}	y^{*++}	y^{0}	y^{0++}	#
1	100.0757	50.5415					A							12
2	228.1343	114.5708	211.1077	106.0575			Q	1175.6419	588.3246	1158.6154	579.8113	1157.6313	579.3193	11
3	299.1714	150.0893	282.1448	141.5761			A	1047.5833	524.2953	1030.5568	515.7820	1029.5728	515.2900	10
4	400.2191	200.6132	383.1925	192.0999	382.2085	191.6079	T	976.5462	488.7767	959.5197	480.2635	958.5356	479.7715	9
5	457.2405	229.1239	440.2140	220.6106	439.2300	220.1186	G	875.4985	438.2529	858.4720	429.7396			8
6	613.3668	307.1870	596.3402	298.6738	595.3562	298.1817	K	818.4771	409.7422	801.4505	401.2289			7
7	710.4196	355.7134	693.3930	347.2001	692.4090	346.7081	P	662.3508	331.6790	645.3243	323.1658			6
8	781.4567	391.2320	764.4301	382.7187	763.4461	382.2267	A	565.2980	283.1527	548.2715	274.6394			5
9	909.5152	455.2613	892.4887	446.7480	891.5047	446.2560	Q	494.2609	247.6341	477.2344	239.1208			4
10	1072.5786	536.7929	1055.5520	528.2797	1054.5680	527.7876	Y	366.2023	183.6048					3
11	1185.6626	593.3350	1168.6361	584.8217	1167.6521	584.3297	I	203.1390	102.0731					2
12							A	90.0550	45.5311					1

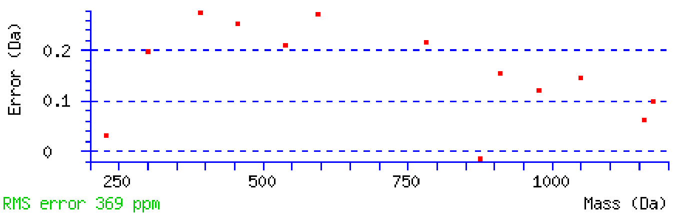

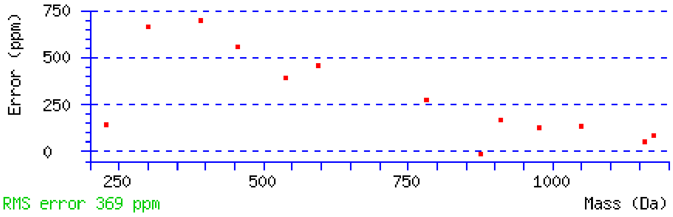

NCBI **BLAST** search of AQATGKPAQYIA
(Parameters: blastp, nr protein database, expect=20000, no filter, PAM30)
Other BLAST web gateways

All matches to this query

Score	Mr(calc)	Delta	Sequence
39.5	1273.7030	0.0043	AQATGKPAQYIA
24.1	1273.6952	0.0122	LKDLKGQMEA
23.4	1273.5860	0.1213	EGLGAPEVGAEMA
22.1	1273.5319	0.1755	AYCQSTMLDTN
18.9	1273.6455	0.0618	GAAQYYVAGHLP
17.5	1273.5972	0.1101	AGAAKCPGSEGSSP
17.2	1273.7646	-0.0572	EAILKGLQSIF
16.3	1273.8009	-0.0936	SGILGLSLFKLA
15.9	1273.5826	0.1247	DLETDHQYLA
15.6	1273.5972	0.1101	SSNHTVMIQET

Mascot: http://www.matrixscience.com/

[redacted] Mascot Search Results

Peptide View

MS/MS Fragmentation of **AQATGKPAQYIAVHVVPDQLMTF**
Found in **MIF_MOUSE** in **SwissProt**, Macrophage migration inhibitory factor OS=Mus musculus GN=Mif PE=1 SV=2

Match to Query 9456: 2556.356622 from(853.126150,3+) intensity(25264.7190) scans(9989) rawscans(sn9989) rtinseconds(3740.7446) index(7248)
Title: 7249: Scan 9989 (rt=3740.74) [C:\Users\Administrador\Desktop\AMOSTRAS NO ORBITRAP LEANDRO RODAR NOVAMENTE\HIPOCAMPO\hipocampo_01.raw]
Data file hipocampo_01.temp.mgf

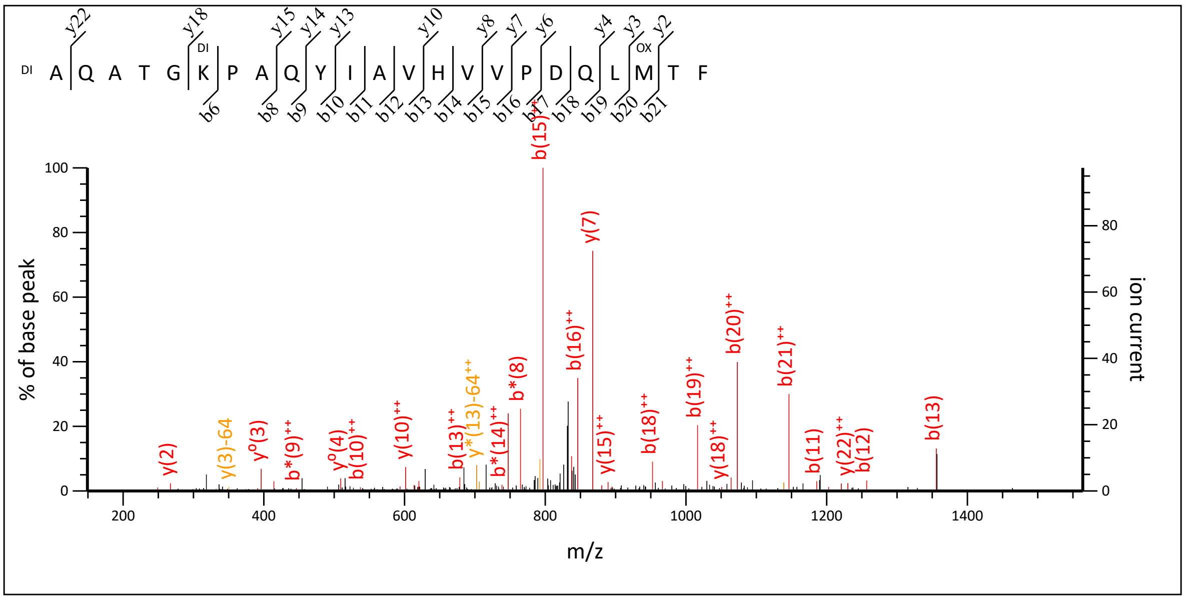

149.24 to 1563.96

Label all possible matches ○ Label matches used for scoring ◉

```
Monoisotopic mass of neutral peptide Mr(calc): 2556.3410
Variable modifications:
N-term : Dimethyl (N-term)
K6     : Dimethyl (K)
M21    : Oxidation (M), with neutral losses 0.0000(shown in table), 63.9983
Ions Score: 45  Expect: 1.1
Matches : 55/374 fragment ions using 96 most intense peaks   (help)
```

#	b	b++	b*	b*++	b0	b0++	Seq.	y	y++	y*	y*++	y0	y0++	#
1	100.0757	50.5415					A							23
2	228.1343	114.5708	211.1077	106.0575			Q	2458.2799	**1229.6436**	2441.2533	1221.1303	2440.2693	1220.6383	22
3	299.1714	150.0893	282.1448	141.5761			A	2330.2213	1165.6143	2313.1948	1157.1010	2312.2108	1156.6090	21
4	400.2191	200.6132	383.1925	192.0999	382.2085	191.6079	T	2259.1842	1130.0957	2242.1577	1121.5825	2241.1736	1121.0905	20
5	457.2405	229.1239	440.2140	220.6106	439.2300	220.1186	G	2158.1365	1079.5719	2141.1100	1071.0586	2140.1260	1070.5666	19
6	**613.3668**	307.1870	596.3402	298.6738	595.3562	298.1817	K	2101.1151	**1051.0612**	2084.0885	1042.5479	2083.1045	1042.0559	18
7	710.4196	355.7134	693.3930	347.2001	692.4090	346.7081	P	1944.9888	972.9980	1927.9623	964.4848	1926.9782	963.9928	17
8	781.4567	***391.2320***	764.4301	382.7187	763.4461	382.2267	A	1847.9360	924.4717	1830.9095	915.9584	1829.9255	915.4664	16
9	909.5152	455.2613	892.4887	446.7480	891.5047	446.2560	Q	1776.8989	**888.9531**	1759.8724	880.4398	1758.8884	879.9478	15
10	**1072.5786**	***536.7929***	1055.5520	528.2797	1054.5680	527.7876	Y	1648.8403	824.9238	1631.8138	816.4105	1630.8298	815.9185	14
11	**1185.6626**	***593.3350***	1168.6361	584.8217	1167.6521	584.3297	I	1485.7770	743.3921	1468.7505	734.8789	1467.7665	734.3869	13
12	**1256.6998**	628.8535	1239.6732	620.3402	1238.6892	619.8482	A	1372.6930	686.8501	1355.6664	678.3368	1354.6824	677.8448	12
13	**1355.7682**	***678.3877***	1338.7416	669.8744	1337.7576	669.3824	V	1301.6558	651.3316	1284.6293	642.8183	1283.6453	642.3263	11
14	1492.8271	***746.9172***	1475.8005	738.4039	1474.8165	737.9119	H	**1202.5874**	**601.7973**	1185.5609	593.2841	1184.5769	592.7921	10
15	1591.8955	***796.4514***	1574.8689	787.9381	1573.8849	787.4461	V	1065.5285	533.2679	1048.5020	524.7546	1047.5179	524.2626	9
16	1690.9639	***845.9856***	1673.9374	837.4723	1672.9533	836.9803	V	**966.4601**	483.7337	949.4335	475.2204	948.4495	474.7284	8
17	1788.0167	***894.5120***	1770.9901	885.9987	1770.0061	885.5067	P	**867.3917**	434.1995	850.3651	425.6862	849.3811	425.1942	7
18	1903.0436	***952.0254***	1886.0171	943.5122	1885.0330	943.0202	D	770.3389	385.6731	753.3124	377.1598	752.3284	376.6678	6
19	2031.1022	***1016.0547***	2014.0756	1007.5415	2013.0916	1007.0495	Q	655.3120	328.1596	638.2854	319.6464	637.3014	319.1543	5
20	2144.1863	***1072.5968***	2127.1597	1064.0835	2126.1757	1063.5915	L	527.2534	264.1303			509.2428	255.1251	4
21	2291.2217	***1146.1145***	2274.1951	1137.6012	2273.2111	1137.1092	M	**414.1693**	207.5883			396.1588	198.5830	3

**22**	2392.2693	1196.6383	2375.2428	1188.1250	2374.2588	1187.6330	**T**	**267.1339**	134.0706			249.1234	125.0653	**2**
**23**							**F**	166.0863	83.5468					**1**

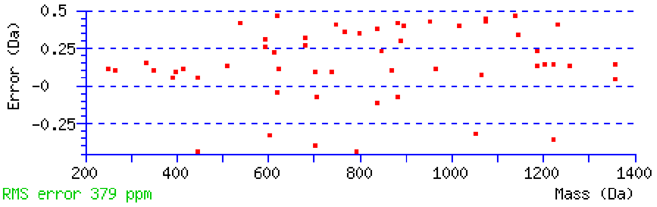

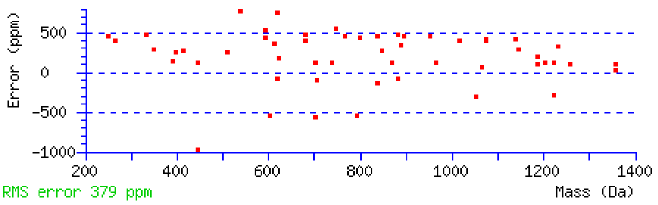

NCBI **BLAST** search of AQATGKPAQYIAVHVVPDQLMTF
(Parameters: blastp, nr protein database, expect=20000, no filter, PAM30)
Other BLAST web gateways

All matches to this query

Score	Mr(calc)	Delta	Sequence
45.0	2556.3410	0.0156	AQATGKPAQYIAVHVVPDQLMTF
14.7	2556.2868	0.0699	MSMIQALAMTVAEIPVFLYTTF
13.8	2556.2750	0.0816	VGVDRADMESLKMMASKPLEEH
13.4	2556.2572	0.0994	PAVMQEFANGRMSKAITSCIMAL
13.2	2556.4544	-0.0978	AVTRVHFPEALGHVALRLVPCPP
12.8	2556.4818	-0.1252	ITELPKSLFEGLFSLQLLLLNA
12.5	2556.2294	0.1273	FSMLRDEDGKPYSPSEYSLQQ
12.3	2556.4615	-0.1049	FSFLPTRALAALKCTCHHFKGI
12.1	2556.4133	-0.0567	TSFILLNLTRAGLGSSAEHFVFV
11.8	2556.5611	-0.2044	KMGVKKGDRVVGYLPNSAHAVE

Mascot: http://www.matrixscience.com/

[redacted] Mascot Search Results

Peptide View

MS/MS Fragmentation of **AQATGKPAQYIAVHVVPDQL**
Found in **MIF_MOUSE** in **SwissProt**, Macrophage migration inhibitory factor OS=Mus musculus GN=Mif PE=1 SV=2

Match to Query 8410: 2169.248648 from(1085.631600,2+) intensity(11146.7990) scans(8075) rawscans(sn8075) rtinseconds(3057.8712) index(5696)
Title: 5697: Scan 8075 (rt=3057.87) [C:\Users\Administrador\Desktop\AMOSTRAS NO ORBITRAP LEANDRO RODAR NOVAMENTE\HIPOCAMPO\hipocampo_01.raw]
Data file hipocampo_01.temp.mgf

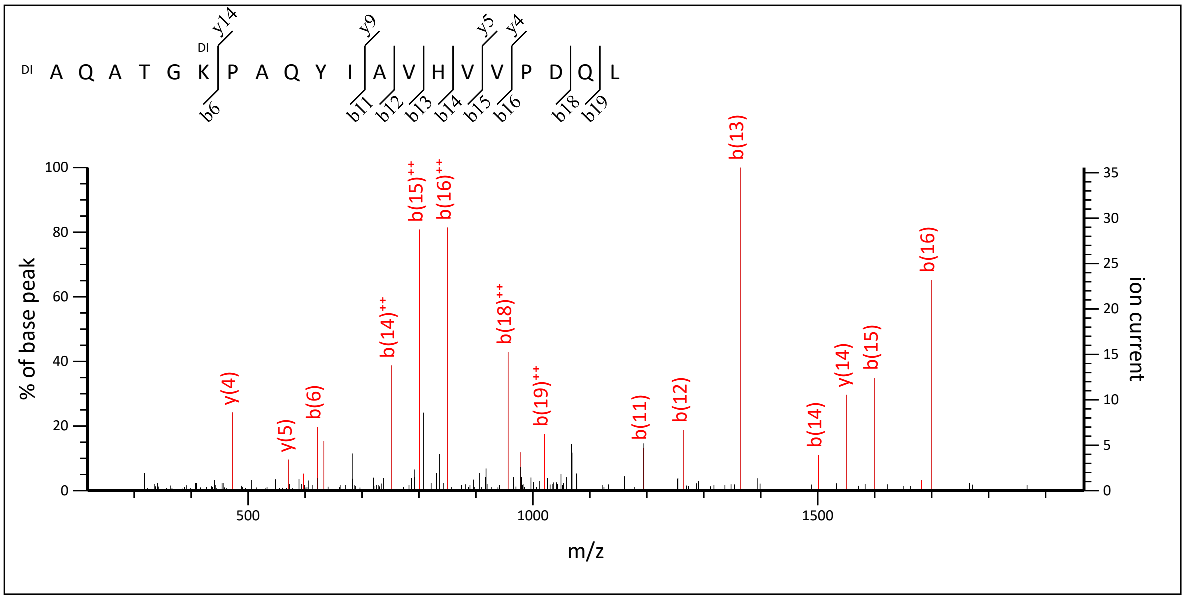

218.35 to 1967.4

Label all possible matches ○ Label matches used for scoring ●

Monoisotopic mass of neutral peptide Mr(calc): 2169.2398
Variable modifications:
N-term : Dimethyl:2H(4) (N-term)
K6 : Dimethyl:2H(4) (K)
Ions Score: 47 **Expect:** 0.62
Matches : 20/214 fragment ions using 31 most intense peaks (help)

#	b	b^{++}	b*	b^{*++}	b^0	b^{0++}	Seq.	y	y^{++}	y*	y^{*++}	y^0	y^{0++}	#
1	104.1008	52.5540					A							20
2	232.1594	116.5833	215.1328	108.0701			Q	2067.1535	1034.0804	2050.1270	1025.5671	2049.1429	1025.0751	19
3	303.1965	152.1019	286.1699	143.5886			A	1939.0949	970.0511	1922.0684	961.5378	1921.0844	961.0458	18
4	404.2442	202.6257	387.2176	194.1124	386.2336	193.6204	T	1868.0578	934.5325	1851.0313	926.0193	1850.0473	925.5273	17
5	461.2656	231.1365	444.2391	222.6232	443.2551	222.1312	G	1767.0101	884.0087	1749.9836	875.4954	1748.9996	875.0034	16
6	621.4170	311.2121	604.3905	302.6989	603.4064	302.2069	K	1709.9887	855.4980	1692.9621	846.9847	1691.9781	846.4927	15
7	718.4698	359.7385	701.4432	351.2252	700.4592	350.7332	P	1549.8373	775.4223	1532.8108	766.9090	1531.8267	766.4170	14
8	789.5069	395.2571	772.4803	386.7438	771.4963	386.2518	A	1452.7845	726.8959	1435.7580	718.3826	1434.7740	717.8906	13
9	917.5655	459.2864	900.5389	450.7731	899.5549	450.2811	Q	1381.7474	691.3774	1364.7209	682.8641	1363.7369	682.3721	12
10	1080.6288	540.8180	1063.6022	532.3048	1062.6182	531.8127	Y	1253.6889	627.3481	1236.6623	618.8348	1235.6783	618.3428	11
11	1193.7129	597.3601	1176.6863	588.8468	1175.7023	588.3548	I	1090.6255	545.8164	1073.5990	537.3031	1072.6150	536.8111	10
12	1264.7500	632.8786	1247.7234	624.3653	1246.7394	623.8733	A	977.5415	489.2744	960.5149	480.7611	959.5309	480.2691	9
13	1363.8184	682.4128	1346.7918	673.8996	1345.8078	673.4075	V	906.5043	453.7558	889.4778	445.2425	888.4938	444.7505	8
14	1500.8773	750.9423	1483.8507	742.4290	1482.8667	741.9370	H	807.4359	404.2216	790.4094	395.7083	789.4254	395.2163	7
15	1599.9457	800.4765	1582.9192	791.9632	1581.9351	791.4712	V	670.3770	335.6921	653.3505	327.1789	652.3665	326.6869	6
16	1699.0141	850.0107	1681.9876	841.4974	1681.0036	841.0054	V	571.3086	286.1579	554.2821	277.6447	553.2980	277.1527	5
17	1796.0669	898.5371	1779.0403	890.0238	1778.0563	889.5318	P	472.2402	236.6237	455.2136	228.1105	454.2296	227.6185	4
18	1911.0938	956.0506	1894.0673	947.5373	1893.0833	947.0453	D	375.1874	188.0974	358.1609	179.5841	357.1769	179.0921	3
19	2039.1524	1020.0798	2022.1259	1011.5666	2021.1418	1011.0746	Q	260.1605	130.5839	243.1339	122.0706			2
20							L	132.1019	66.5546					1

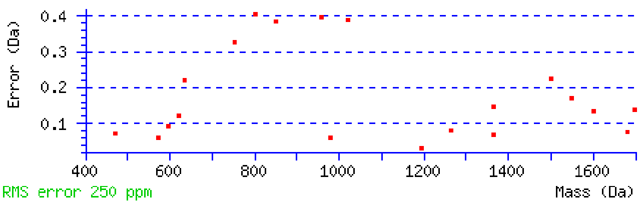

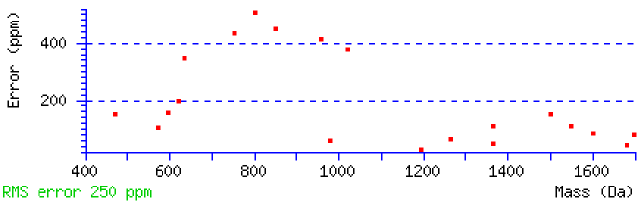

NCBI **BLAST** search of AQATGKPAQYIAVHVVPDQL
(Parameters: blastp, nr protein database, expect=20000, no filter, PAM30)
Other BLAST web gateways

All matches to this query

Score	Mr(calc)	Delta	Sequence
47.3	2169.2398	0.0089	AQATGKPAQYIAVHVVPDQL
24.4	2169.2386	0.0100	VGRVYRWMVDSRVALRI
18.9	2169.2020	0.0466	GQVLSTLKSEDVPYTAALTA
18.4	2169.2207	0.0280	GISGMVAATLTLPFDVVKTQ
17.7	2169.1777	0.0709	TIASAVNCPLYVVHVMSKSA
17.3	2169.2021	0.0465	VQIVVIVVVMMVMVVMITC
17.3	2169.2021	0.0465	VQIVVIVVVMMVMVVMITC
17.3	2169.2021	0.0465	VQIVVIVVVMMVMVVMITC
17.3	2169.2021	0.0465	VQIVVIVVVMMVMVVMITC
17.1	2169.1336	0.1150	RSMNHPTVALVRMVAPSPT

Mascot: http://www.matrixscience.com/

[redacted] Mascot Search Results

Peptide View

MS/MS Fragmentation of **AEDVTAALAKQGL**
Found in **MTAP2_MOUSE** in **SwissProt**, Microtubule-associated protein 2 OS=Mus musculus GN=Map2 PE=1 SV=2

Match to Query 4065: 1341.747968 from(671.881260,2+) intensity(63764.9730) scans(7590) rawscans(sn7590) rtinseconds(2884.8337) index(5309)
Title: 5310: Scan 7590 (rt=2884.83) [C:\Users\Administrador\Desktop\AMOSTRAS NO ORBITRAP LEANDRO RODAR NOVAMENTE\HIPOCAMPO\hipocampo_01.raw]
Data file hipocampo_01.temp.mgf

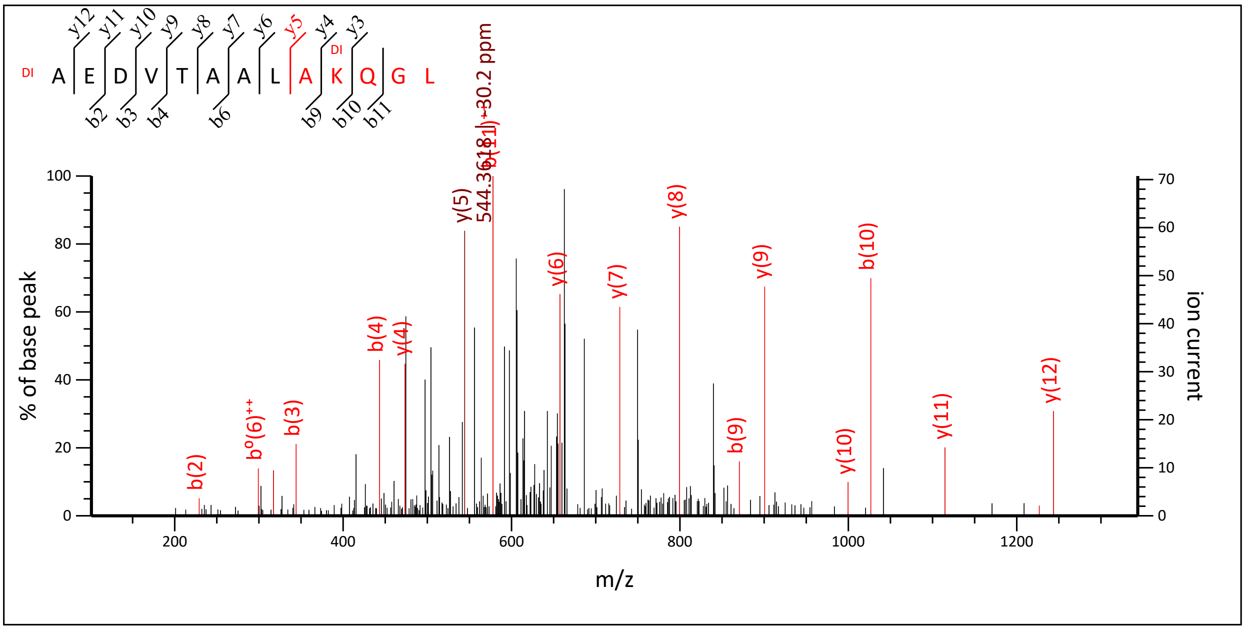

101.09 to 1343.7

Label all possible matches Label matches used for scoring

```
Monoisotopic mass of neutral peptide Mr(calc): 1341.7504
Variable modifications:
N-term : Dimethyl (N-term)
K10    : Dimethyl (K)
Ions Score: 69  Expect: 0.0034
Matches : 21/104 fragment ions using 32 most intense peaks   (help)
```

#	b	b^{++}	b*	b^{*++}	b^0	b^{0++}	Seq.	y	y^{++}	y*	y^{*++}	y^0	y^{0++}	#
1	100.0757	50.5415					A							13
2	**229.1183**	115.0628			211.1077	106.0575	E	***1243.6892***	622.3483	1226.6627	613.8350	1225.6787	613.3430	12
3	**344.1452**	172.5763			326.1347	163.5710	D	***1114.6467***	557.8270	1097.6201	549.3137	1096.6361	548.8217	11
4	**443.2136**	222.1105			425.2031	213.1052	V	***999.6197***	500.3135	982.5932	491.8002	981.6091	491.3082	10
5	**544.2613**	272.6343			526.2508	263.6290	T	***900.5513***	450.7793	883.5247	442.2660	882.5407	441.7740	9
6	615.2984	308.1529			597.2879	299.1476	A	***799.5036***	400.2554	782.4771	391.7422			8
7	686.3355	343.6714			668.3250	334.6661	A	***728.4665***	364.7369	711.4400	356.2236			7
8	**799.4196**	400.2134			781.4090	391.2082	L	***657.4294***	329.2183	640.4028	320.7051			6
9	**870.4567**	435.7320			852.4462	426.7267	A	***544.3453***	272.6763	527.3188	264.1630			5
10	**1026.5830**	513.7951	1009.5564	505.2819	1008.5724	504.7898	K	***473.3082***	237.1577	456.2817	228.6445			4
11	1154.6416	577.8244	1137.6150	569.3111	1136.6310	568.8191	Q	***317.1819***	159.0946	300.1554	150.5813			3
12	1211.6630	606.3352	1194.6365	597.8219	1193.6525	597.3299	G	189.1234	95.0653					2
13							L	132.1019	66.5546					1

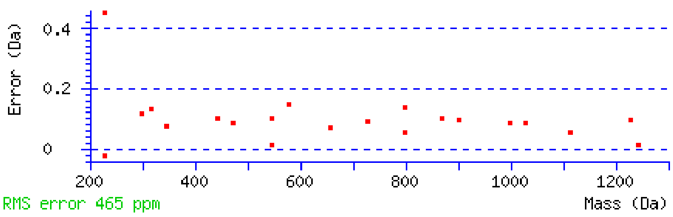

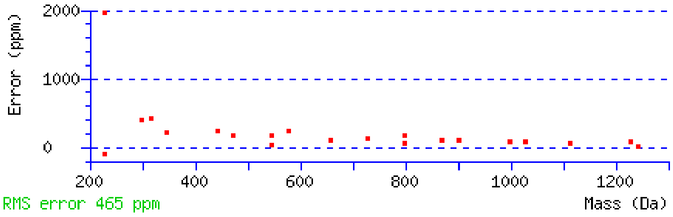

NCBI **BLAST** search of AEDVTAALAKQGL
(Parameters: blastp, nr protein database, expect=20000, no filter, PAM30)
Other BLAST web gateways

All matches to this query

Score	Mr(calc)	Delta	Sequence
69.5	1341.7504	-0.0024	AEDVTAALAKQGL
29.2	1341.6776	0.0704	AEDVTAPLVDER
23.3	1341.6776	0.0704	AEGQEGQATTIPI
22.9	1341.6599	0.0881	TGDNMGHVILLS
21.6	1341.8909	-0.1429	KTVTAAIKVMD
20.4	1341.8847	-0.1367	TTLSLVKTVVII
20.0	1341.7343	0.0137	LLWGSPAARGSPG
20.0	1341.7798	-0.0318	IPPEAAATGQRVP
20.0	1341.8785	-0.1305	LVVVLATAIALLD
19.6	1341.7027	0.0452	SVSESPAILPVSE

Mascot: http://www.matrixscience.com/

[redacted] Mascot Search Results

Peptide View

MS/MS Fragmentation of **GRKGPGPGGPGGAGGARGGAGGGPSGD**
Found in **NEUG_MOUSE** in **SwissProt**, Neurogranin OS=Mus musculus GN=Nrgn PE=1 SV=1

Match to Query 8425: 2173.080372 from(725.367400,3+) intensity(17060.7050) scans(2144) rawscans(sn2144) rtinseconds(1045.6394) index(527)
Title: 528: Scan 2144 (rt=1045.64) [C:\Users\Administrador\Desktop\AMOSTRAS NO ORBITRAP LEANDRO RODAR NOVAMENTE\HIPOCAMPO\hipocampo_01.raw]
Data file hipocampo_01.temp.mgf

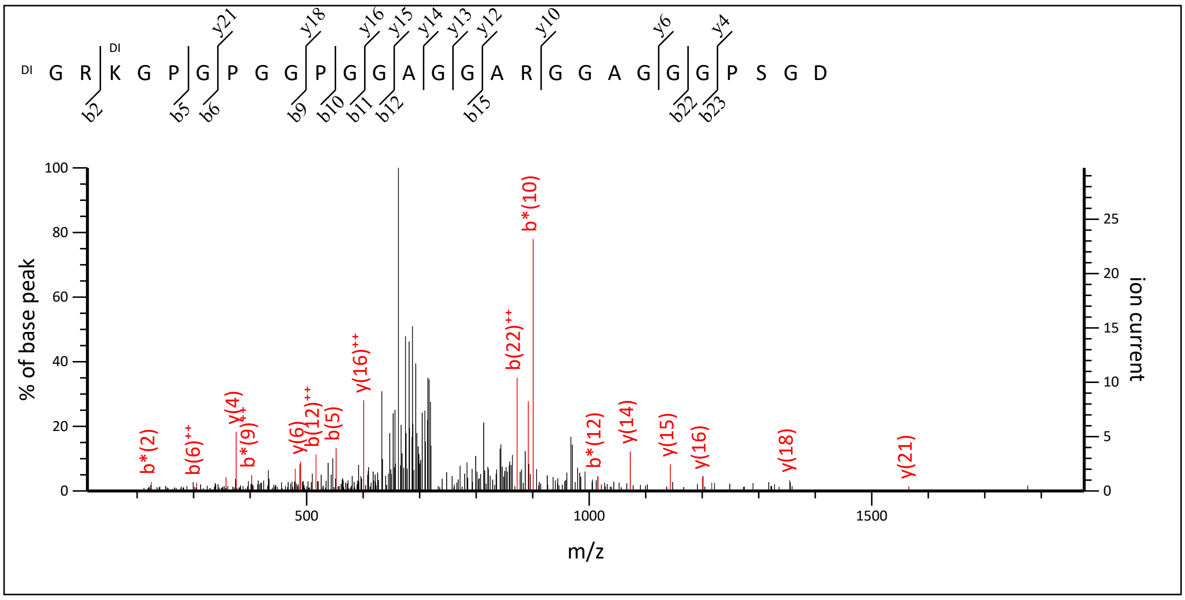

112.08 to 1875.96

Label all possible matches ○ Label matches used for scoring ◉

Monoisotopic mass of neutral peptide Mr(calc): 2173.0737
Variable modifications:
N-term : Dimethyl (N-term)
K3 : Dimethyl (K)
Ions Score: 33 **Expect:** 18
Matches : 25/242 fragment ions using 38 most intense peaks (help)

#	b	b++	b*	b*++	b0	b0++	Seq.	y	y++	y*	y*++	y0	y0++	#
1	86.0600	43.5337					G							27
2	242.1612	121.5842	225.1346	113.0709			R	2089.0282	1045.0177	2072.0016	1036.5045	2071.0176	1036.0125	26
3	398.2874	199.6473	381.2609	191.1341			K	1932.9271	966.9672	1915.9005	958.4539	1914.9165	957.9619	25
4	455.3089	228.1581	438.2823	219.6448			G	1776.8008	888.9040	1759.7743	880.3908	1758.7903	879.8988	24
5	552.3616	276.6845	535.3351	268.1712			P	1719.7794	860.3933	1702.7528	851.8800	1701.7688	851.3880	23
6	609.3831	305.1952	592.3566	296.6819			G	1622.7266	811.8669	1605.7000	803.3537	1604.7160	802.8616	22
7	706.4359	353.7216	689.4093	345.2083			P	1565.7051	783.3562	1548.6786	774.8429	1547.6946	774.3509	21
8	763.4573	382.2323	746.4308	373.7190			G	1468.6524	734.8298	1451.6258	726.3165	1450.6418	725.8245	20
9	820.4788	410.7430	803.4522	402.2298			G	1411.6309	706.3191	1394.6043	697.8058	1393.6203	697.3138	19
10	917.5316	459.2694	900.5050	450.7561			P	1354.6094	677.8084	1337.5829	669.2951	1336.5989	668.8031	18
11	974.5530	487.7802	957.5265	479.2669			G	1257.5567	629.2820	1240.5301	620.7687	1239.5461	620.2767	17
12	1031.5745	516.2909	1014.5479	507.7776			G	1200.5352	600.7712	1183.5087	592.2580	1182.5246	591.7660	16
13	1102.6116	551.8094	1085.5851	543.2962			A	1143.5137	572.2605	1126.4872	563.7472	1125.5032	563.2552	15
14	1159.6331	580.3202	1142.6065	571.8069			G	1072.4766	536.7420	1055.4501	528.2287	1054.4661	527.7367	14
15	1216.6545	608.8309	1199.6280	600.3176			G	1015.4552	508.2312	998.4286	499.7179	997.4446	499.2259	13
16	1287.6916	644.3495	1270.6651	635.8362			A	958.4337	479.7205	941.4071	471.2072	940.4231	470.7152	12
17	1443.7928	722.4000	1426.7662	713.8867			R	887.3966	444.2019	870.3700	435.6887	869.3860	435.1966	11
18	1500.8142	750.9107	1483.7877	742.3975			G	731.2955	366.1514			713.2849	357.1461	10
19	1557.8357	779.4215	1540.8091	770.9082			G	674.2740	337.6406			656.2634	328.6354	9
20	1628.8728	814.9400	1611.8463	806.4268			A	617.2525	309.1299			599.2420	300.1246	8
21	1685.8943	843.4508	1668.8677	834.9375			G	546.2154	273.6114			528.2049	264.6061	7
22	1742.9157	871.9615	1725.8892	863.4482			G	489.1940	245.1006			471.1834	236.0953	6

23	1799.9372	***900.4722***	1782.9106	891.9590			G	432.1725	216.5899			414.1619	207.5846	5
24	1896.9900	948.9986	1879.9634	940.4853			P	***375.1510***	188.0792			357.1405	179.0739	4
25	1984.0220	992.5146	1966.9954	984.0014	1966.0114	983.5093	S	278.0983	139.5528			260.0877	130.5475	3
26	2041.0434	1021.0254	2024.0169	1012.5121	2023.0329	1012.0201	G	191.0662	96.0368			173.0557	87.0315	2
27							D	134.0448	67.5260			116.0342	58.5207	1

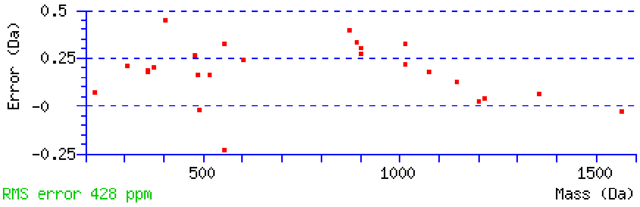

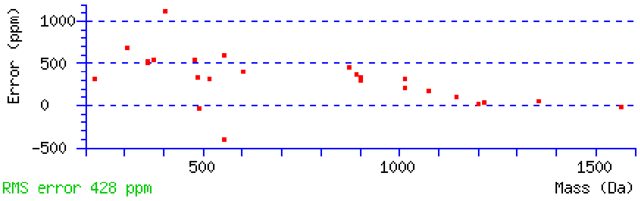

NCBI **BLAST** search of GRKGPGPGGPGGAGGARGGAGGGPSGD
(Parameters: blastp, nr protein database, expect=20000, no filter, PAM30)
Other BLAST web gateways

All matches to this query

Score	Mr(calc)	Delta	Sequence
32.7	2173.0737	0.0066	GRKGPGPGGPGGAGGARGGAGGGPSGD
20.0	2173.3276	-0.2472	PGLVGLVLSYALSLTGLLSGLV
15.5	2173.1793	-0.0990	VSRNWARAPLSPGVTTTLTD
14.4	2173.1052	-0.0248	QYEYGSGKKYGGPCGGRNC
13.3	2173.3988	-0.3184	LKMRISRLVPWLILASVV
12.1	2173.1565	-0.0762	TLRLSLWGMEALGTLGGQVQ
11.4	2173.0184	0.0620	DPSAPCFAPSTLLSPEASPCR
11.0	2173.2793	-0.1989	EIAELLRAHSEPGRSLGPK
10.9	2173.2371	-0.1568	KHLHTLIFNAARDLLINE
10.8	2173.1467	-0.0663	AADGTGVVGGGAVGGGLPKDGLQD

Mascot: http://www.matrixscience.com/

[redacted] Mascot Search Results

Peptide View

MS/MS Fragmentation of **KGPGPGGPGGAGGARGGAGGGPSGD**
Found in **NEUG_MOUSE** in **SwissProt**, Neurogranin OS=Mus musculus GN=Nrgn PE=1 SV=1

Match to Query 7492: 1959.958782 from(654.326870,3+) intensity(305028.6900) scans(2351) rawscans(sn2351) rtinseconds(1121.3576) index(719)
Title: 720: Scan 2351 (rt=1121.36) [C:\Users\Administrador\Desktop\AMOSTRAS NO ORBITRAP LEANDRO RODAR NOVAMENTE\HIPOCAMPO\hipocampo_01.raw]
Data file hipocampo_01.temp.mgf

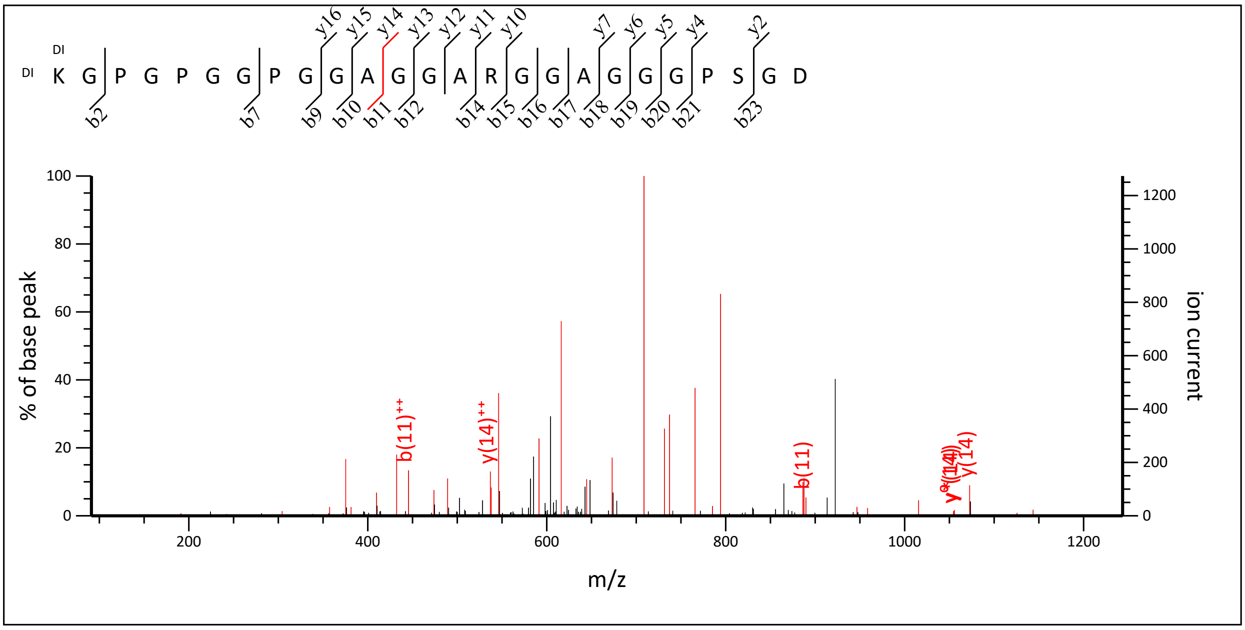

91.13 to 1243.58

Label all possible matches ○ Label matches used for scoring ◉

```
Monoisotopic mass of neutral peptide Mr(calc): 1959.9512
Variable modifications:
N-term : Dimethyl (N-term)
K1     : Dimethyl (K)
Ions Score: 106  Expect: 9.1e-007
Matches : 36/224 fragment ions using 46 most intense peaks   (help)
```

#	b	b++	b*	b*++	b0	b0++	Seq.	y	y++	y*	y*++	y0	y0++	#
1	185.1648	93.0861	168.1383	84.5728			K							25
2	**242.1863**	121.5968	225.1598	113.0835			G	1776.8008	888.9040	1759.7743	880.3908	1758.7903	879.8988	24
3	339.2391	170.1232	322.2125	161.6099			P	1719.7794	860.3933	1702.7528	851.8800	1701.7688	851.3880	23
4	396.2605	198.6339	379.2340	190.1206			G	1622.7266	811.8669	1605.7000	803.3537	1604.7160	802.8616	22
5	493.3133	247.1603	476.2867	238.6470			P	1565.7051	783.3562	1548.6786	774.8429	1547.6946	774.3509	21
6	550.3348	275.6710	533.3082	267.1577			G	1468.6524	734.8298	1451.6258	726.3165	1450.6418	725.8245	20
7	607.3562	***304.1817***	590.3297	295.6685			G	1411.6309	706.3191	1394.6043	697.8058	1393.6203	697.3138	19
8	704.4090	352.7081	687.3824	344.1949			P	1354.6094	677.8084	1337.5829	669.2951	1336.5989	668.8031	18
9	761.4305	***381.2189***	744.4039	372.7056			G	1257.5567	629.2820	1240.5301	620.7687	1239.5461	620.2767	17
10	818.4519	***409.7296***	801.4254	401.2163			G	1200.5352	600.7712	1183.5087	592.2580	1182.5246	591.7660	16
11	**889.4890**	***445.2482***	872.4625	436.7349			A	**1143.5137**	572.2605	1126.4872	563.7472	1125.5032	563.2552	15
12	**946.5105**	***473.7589***	929.4839	465.2456			G	**1072.4766**	536.7420	1055.4501	528.2287	1054.4661	527.7367	14
13	1003.5320	502.2696	986.5054	493.7563			G	**1015.4552**	508.2312	998.4286	499.7179	997.4446	499.2259	13
14	1074.5691	***537.7882***	1057.5425	529.2749			A	**958.4337**	479.7205	941.4071	471.2072	940.4231	470.7152	12
15	1230.6702	***615.8387***	1213.6436	607.3255			R	**887.3966**	444.2019	870.3700	435.6887	869.3860	435.1966	11
16	1287.6916	***644.3495***	1270.6651	635.8362			G	**731.2955**	366.1514			713.2849	357.1461	10
17	1344.7131	***672.8602***	1327.6866	664.3469			G	674.2740	337.6406			656.2634	328.6354	9
18	1415.7502	***708.3788***	1398.7237	699.8655			A	617.2525	309.1299			599.2420	300.1246	8

**19**	1472.7717	***736.8895***	1455.7451	728.3762			**G**	**546.2154**	273.6114			528.2049	264.6061	**7**
**20**	1529.7932	***765.4002***	1512.7666	756.8869			**G**	**489.1940**	245.1006			471.1834	236.0953	**6**
**21**	1586.8146	***793.9109***	1569.7881	785.3977			**G**	**432.1725**	216.5899			414.1619	207.5846	**5**
**22**	1683.8674	842.4373	1666.8408	833.9241			**P**	**375.1510**	188.0792			357.1405	179.0739	**4**
**23**	1770.8994	***885.9533***	1753.8729	877.4401	1752.8888	876.9481	**S**	278.0983	139.5528			260.0877	130.5475	**3**
**24**	1827.9209	914.4641	1810.8943	905.9508	1809.9103	905.4588	**G**	**191.0662**	96.0368			173.0557	87.0315	**2**
**25**							**D**	134.0448	67.5260			116.0342	58.5207	**1**

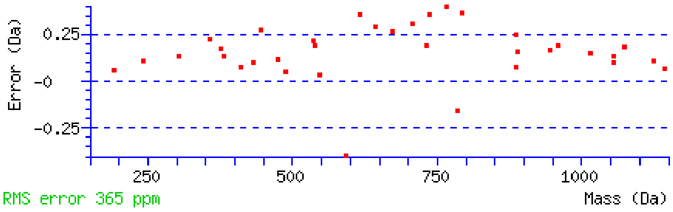

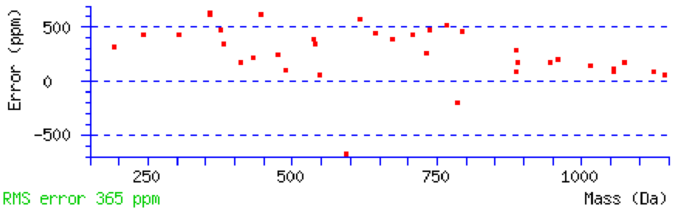

NCBI **BLAST** search of KGPGPGGPGGAGGARGGAGGGPSGD
(Parameters: blastp, nr protein database, expect=20000, no filter, PAM30)
Other BLAST web gateways

All matches to this query

Score	Mr(calc)	Delta	Sequence
105.5	1959.9512	0.0076	KGPGPGGPGGAGGARGGAGGGPSGD
30.4	1959.9185	0.0403	TAIGGGGGPGGGGSGAIDEGGGRD
29.3	1959.9212	0.0376	VLFLMMSGAPDAGGQGGGGGGG
25.2	1960.0757	-0.1169	WNGLLPDDTLQDLGLGK
25.0	1960.0036	-0.0448	KHLNNMQNLQNGGGEQV
24.1	1960.0030	-0.0442	LNDGNFDNFVADKDTVL
22.6	1960.0955	-0.1367	GVKYLIVLINKMDDPT
21.5	1960.0240	-0.0653	EENGPQKGGLEVADEALV
21.3	1960.0683	-0.1095	YRWRAWLLGDAVAGVTV
21.0	1959.8344	0.1243	GEGGGGGGGAANPAGGDSAVAGDE

Mascot: http://www.matrixscience.com/

[redacted] Mascot Search Results

Peptide View

MS/MS Fragmentation of **RKGPGPGGPGGAGGARGGAGGGPSGD**
Found in **NEUG_MOUSE** in **SwissProt**, Neurogranin OS=Mus musculus GN=Nrgn PE=1 SV=1

Match to Query 8201: 2116.063242 from(706.361690,3+) intensity(39999.9770) scans(2066) rawscans(sn2066) rtinseconds(1016.7325) index(457)
Title: 458: Scan 2066 (rt=1016.73) [C:\Users\Administrador\Desktop\AMOSTRAS NO ORBITRAP LEANDRO RODAR NOVAMENTE\HIPOCAMPO\hipocampo_01.raw]
Data file hipocampo_01.temp.mgf

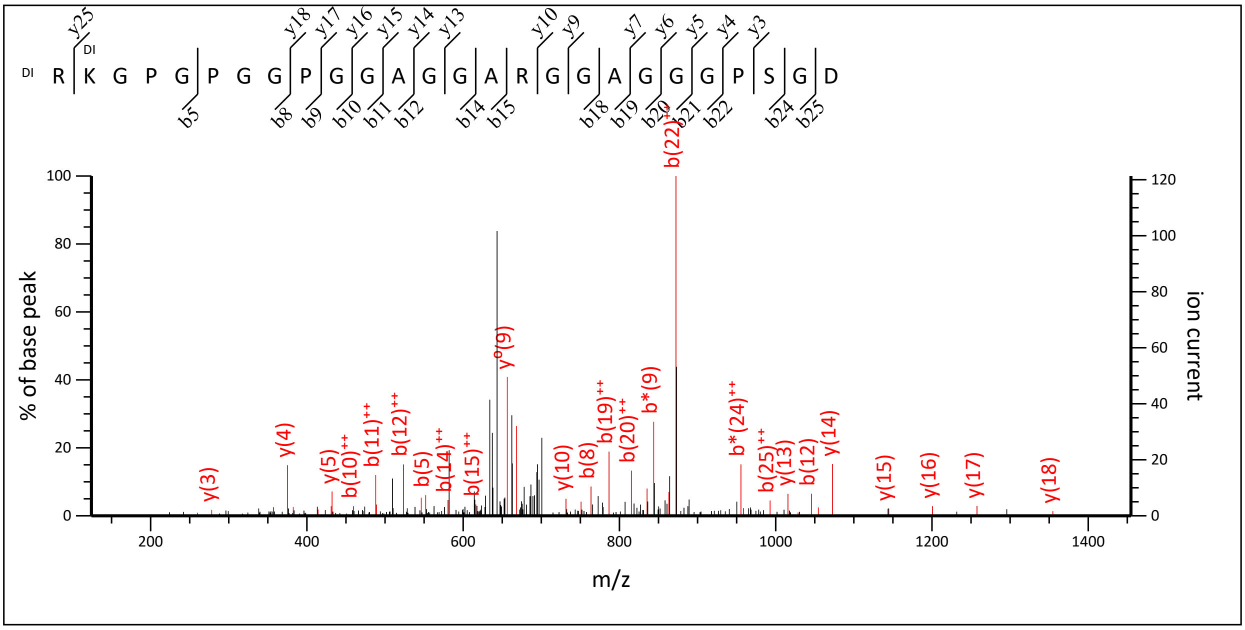

124.22 to 1454.67

Label all possible matches ○ Label matches used for scoring ◉

Monoisotopic mass of neutral peptide Mr(calc): 2116.0523
Variable modifications:
N-term : Dimethyl (N-term)
K2 : Dimethyl (K)
Ions Score: 82 **Expect:** 0.00022
Matches : 44/234 fragment ions using 68 most intense peaks (help)

#	b	b^{++}	b*	b^{*++}	b^0	b^{0++}	Seq.	y	y^{++}	y*	y^{*++}	y^0	y^{0++}	#
1	185.1397	93.0735	168.1131	84.5602			R							26
2	341.2659	171.1366	324.2394	162.6233			K	1932.9271	966.9672	1915.9005	958.4539	1914.9165	957.9619	25
3	398.2874	199.6473	381.2609	191.1341			G	1776.8008	888.9040	1759.7743	880.3908	1758.7903	879.8988	24
4	495.3402	248.1737	478.3136	239.6605			P	1719.7794	860.3933	1702.7528	851.8800	1701.7688	851.3880	23
5	**552.3616**	276.6845	535.3351	268.1712			G	1622.7266	811.8669	1605.7000	803.3537	1604.7160	802.8616	22
6	649.4144	325.2108	632.3879	316.6976			P	1565.7051	783.3562	1548.6786	774.8429	1547.6946	774.3509	21
7	706.4359	353.7216	689.4093	345.2083			G	1468.6524	734.8298	1451.6258	726.3165	1450.6418	725.8245	20
8	**763.4573**	***382.2323***	746.4308	373.7190			G	1411.6309	706.3191	1394.6043	697.8058	1393.6203	697.3138	19
9	860.5101	***430.7587***	843.4835	422.2454			P	***1354.6094***	677.8084	1337.5829	669.2951	1336.5989	668.8031	18
10	917.5316	***459.2694***	900.5050	450.7561			G	***1257.5567***	629.2820	1240.5301	620.7687	1239.5461	620.2767	17
11	974.5530	***487.7802***	957.5265	479.2669			G	***1200.5352***	600.7712	1183.5087	592.2580	1182.5246	591.7660	16
12	**1045.5901**	***523.2987***	1028.5636	514.7854			A	***1143.5137***	572.2605	1126.4872	563.7472	1125.5032	563.2552	15
13	1102.6116	***551.8094***	1085.5851	543.2962			G	***1072.4766***	536.7420	1055.4501	528.2287	1054.4661	527.7367	14
14	1159.6331	***580.3202***	1142.6065	571.8069			G	***1015.4552***	508.2312	998.4286	499.7179	997.4446	499.2259	13
15	1230.6702	***615.8387***	1213.6436	607.3255			A	***958.4337***	479.7205	941.4071	471.2072	940.4231	470.7152	12
16	1386.7713	693.8893	1369.7447	685.3760			R	887.3966	444.2019	870.3700	435.6887	869.3860	435.1966	11
17	1443.7928	722.4000	1426.7662	713.8867			G	***731.2955***	366.1514			713.2849	357.1461	10
18	1500.8142	***750.9107***	1483.7877	742.3975			G	674.2740	337.6406			656.2634	328.6354	9

**19**	1571.8513	***786.4293***	1554.8248	777.9160			**A**	617.2525	309.1299			599.2420	300.1246	**8**
**20**	1628.8728	***814.9400***	1611.8463	806.4268			**G**	***546.2154***	273.6114			528.2049	264.6061	**7**
**21**	1685.8943	***843.4508***	1668.8677	834.9375			**G**	***489.1940***	245.1006			471.1834	236.0953	**6**
**22**	1742.9157	***871.9615***	1725.8892	863.4482			**G**	***432.1725***	216.5899			414.1619	207.5846	**5**
**23**	1839.9685	920.4879	1822.9419	911.9746			**P**	***375.1510***	188.0792			357.1405	179.0739	**4**
**24**	1927.0005	964.0039	1909.9740	955.4906	1908.9900	954.9986	**S**	***278.0983***	139.5528			260.0877	130.5475	**3**
**25**	1984.0220	***992.5146***	1966.9954	984.0014	1966.0114	983.5093	**G**	191.0662	96.0368			173.0557	87.0315	**2**
**26**							**D**	134.0448	67.5260			116.0342	58.5207	**1**

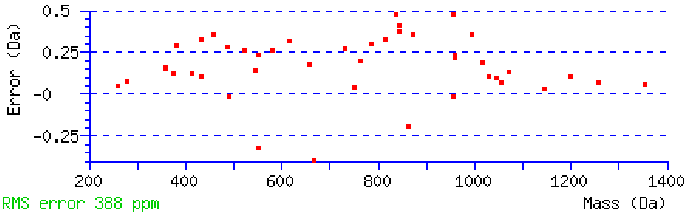

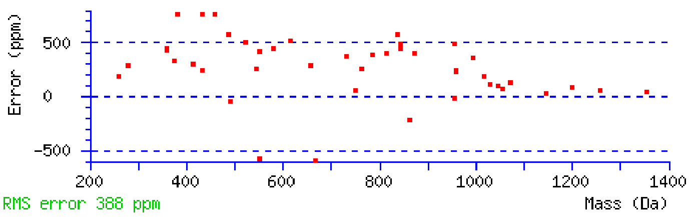

NCBI **BLAST** search of RKGPGPGGPGGAGGARGGAGGGPSGD
(Parameters: blastp, nr protein database, expect=20000, no filter, PAM30)
Other BLAST web gateways

All matches to this query

Score	Mr(calc)	Delta	Sequence
81.8	2116.0523	0.0110	RKGPGPGGPGGAGGARGGAGGGPSGD
19.6	2116.0739	-0.0106	GDYVQQHGGVSLVEQLLQD
18.7	2116.2255	-0.1623	GPKGSRGYIGLPGLFGLPGSD
17.6	2116.0358	0.0274	IGDGANDVSMIQVADIGIGVS
17.4	2115.9780	0.0852	LAGMSPGAGAMAGMSGSAGAAGVAG
16.6	2116.0722	-0.0089	VTEMKALINADELANDVAGA
16.0	2116.1503	-0.0870	FRGMTLLHLAAAQGYATLI
15.7	2116.1056	-0.0424	LSGFSTAMGALAGGFGALPDGLA
15.6	2115.9495	0.1137	GMKGVMEMMVALCGSEREA
14.6	2115.9495	0.1137	GMKGVMEMMVALCGSEREA

Mascot: http://www.matrixscience.com/

[redacted] Mascot Search Results

Peptide View

MS/MS Fragmentation of ADDEPLGRVSF
Found in PPIA_MOUSE in SwissProt, Peptidyl-prolyl cis-trans isomerase A OS=Mus musculus GN=Ppia PE=1 SV=2

Match to Query 3429: 1232.607428 from(617.310990,2+) intensity(48155.3950) scans(7430) rawscans(sn7430) rtinseconds(2852.3698) index(5207)
Title: 5208: Scan 7430 (rt=2852.37) [C:\Users\Administrador\Desktop\AMOSTRAS NO ORBITRAP LEANDRO RODAR NOVAMENTE\CORTEX\cortex_01.raw]
Data file cortex_01.temp.mgf

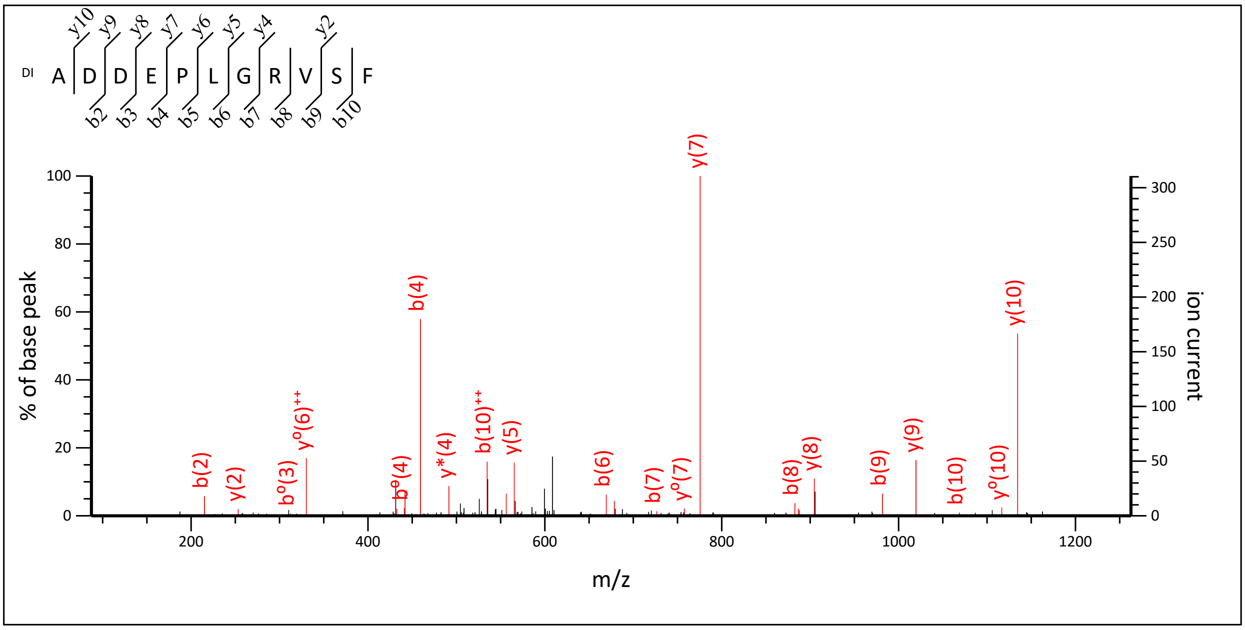

87.3 to 1262.73

Label all possible matches ○ Label matches used for scoring ◉

Monoisotopic mass of neutral peptide Mr(calc): 1232.6037
Variable modifications:
N-term : Dimethyl (N-term)
Ions Score: 60 **Expect:** 0.029
Matches : 28/96 fragment ions using 49 most intense peaks (help)

#	b	b^{++}	b^*	b^{*++}	b^0	b^{0++}	Seq.	y	y^{++}	y^*	y^{*++}	y^0	y^{0++}	#
1	100.0757	50.5415					A							11
2	215.1026	108.0550			197.0921	99.0497	D	1134.5426	567.7749	1117.5160	559.2617	1116.5320	558.7696	10
3	330.1296	165.5684			312.1190	156.5631	D	1019.5156	510.2615	1002.4891	501.7482	1001.5051	501.2562	9
4	459.1722	230.0897			441.1616	221.0844	E	904.4887	452.7480	887.4621	444.2347	886.4781	443.7427	8
5	556.2249	278.6161			538.2144	269.6108	P	775.4461	388.2267	758.4196	379.7134	757.4355	379.2214	7
6	669.3090	335.1581			651.2984	326.1529	L	678.3933	339.7003	661.3668	331.1870	660.3828	330.6950	6
7	726.3305	363.6689			708.3199	354.6636	G	565.3093	283.1583	548.2827	274.6450	547.2987	274.1530	5
8	882.4316	441.7194	865.4050	433.2061	864.4210	432.7141	R	508.2878	254.6475	491.2613	246.1343	490.2772	245.6423	4
9	981.5000	491.2536	964.4734	482.7404	963.4894	482.2483	V	352.1867	176.5970			334.1761	167.5917	3
10	1068.5320	534.7696	1051.5055	526.2564	1050.5214	525.7644	S	253.1183	127.0628			235.1077	118.0575	2
11							F	166.0863	83.5468					1

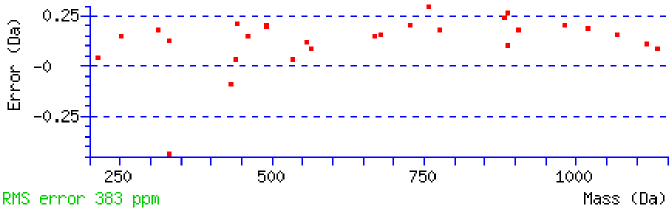

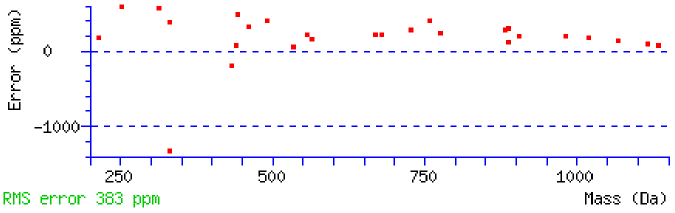

NCBI **BLAST** search of ADDEPLGRVSF
(Parameters: blastp, nr protein database, expect=20000, no filter, PAM30)
Other BLAST web gateways

All matches to this query

Score	Mr(calc)	Delta	Sequence
60.1	1232.6037	0.0037	ADDEPLGRVSF
26.9	1232.7832	-0.1757	SVVLKALEDPV
26.9	1232.5710	0.0364	PSLESDEDNPV
26.8	1232.5884	0.0190	ADDEKSEDRI
26.8	1232.6765	-0.0691	ADPKGHLETPI
26.6	1232.6111	-0.0037	ALVFPNSDPVM
25.9	1232.5781	0.0293	EGMICNVELPT
24.5	1232.7591	-0.1517	SKEPNLVVSF
24.1	1232.6435	-0.0360	KTMRSTTDPV
22.6	1232.5747	0.0327	WNEADMEVIV

Mascot: http://www.matrixscience.com/

[redacted] Mascot Search Results

Peptide View

MS/MS Fragmentation of **ADKVPKTAENF**
Found in **PPIA_MOUSE** in **SwissProt**, Peptidyl-prolyl cis-trans isomerase A OS=Mus musculus GN=Ppia PE=1 SV=2

Match to Query 3953: 1302.721248 from(652.367900,2+) intensity(502996.7200) scans(3630) rawscans(sn3630) rtinseconds(1558.8044) index(1888)
Title: 1889: Scan 3630 (rt=1558.8) [C:\Users\Administrador\Desktop\AMOSTRAS NO ORBITRAP LEANDRO RODAR NOVAMENTE\CORTEX\cortex_01.raw]
Data file cortex_01.temp.mgf

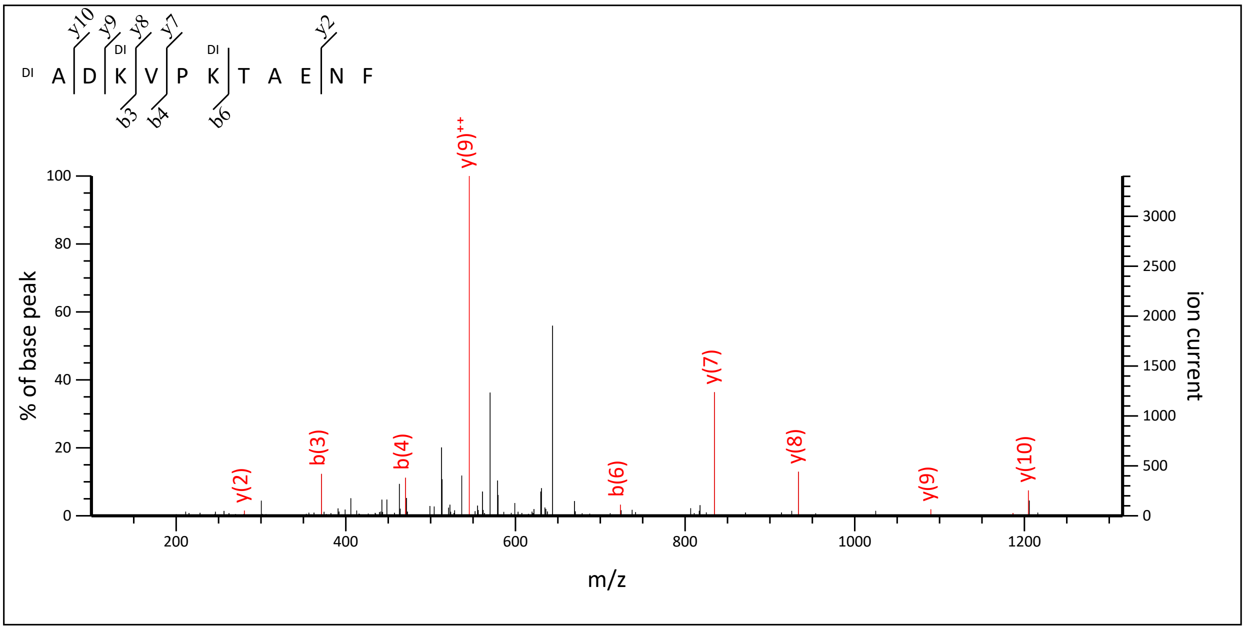

100.09 to 1315.79

Label all possible matches ○ Label matches used for scoring ◉

Monoisotopic mass of neutral peptide Mr(calc): 1302.7184
Variable modifications:
N-term : Dimethyl (N-term)
K3 : Dimethyl (K)
K6 : Dimethyl (K)
Ions Score: 40 **Expect:** 3.1
Matches : 10/108 fragment ions using 11 most intense peaks (help)

#	b	b^{++}	b*	b^{*++}	b^0	b^{0++}	Seq.	y	y^{++}	y*	y^{*++}	y^0	y^{0++}	#
1	100.0757	50.5415					A							11
2	215.1026	108.0550			197.0921	99.0497	D	***1204.6572***	602.8322	1187.6307	594.3190	1186.6467	593.8270	10
3	**371.2289**	186.1181	354.2023	177.6048	353.2183	177.1128	K	***1089.6303***	545.3188	1072.6037	536.8055	1071.6197	536.3135	9
4	**470.2973**	235.6523	453.2708	227.1390	452.2867	226.6470	V	***933.5040***	467.2556	916.4775	458.7424	915.4934	458.2504	8
5	567.3501	284.1787	550.3235	275.6654	549.3395	275.1734	P	***834.4356***	417.7214	817.4090	409.2082	816.4250	408.7162	7
6	**723.4763**	362.2418	706.4498	353.7285	705.4658	353.2365	K	737.3828	369.1951	720.3563	360.6818	719.3723	360.1898	6
7	824.5240	412.7656	807.4975	404.2524	806.5135	403.7604	T	581.2566	291.1319	564.2300	282.6186	563.2460	282.1266	5
8	895.5611	448.2842	878.5346	439.7709	877.5506	439.2789	A	480.2089	240.6081	463.1823	232.0948	462.1983	231.6028	4
9	1024.6037	512.8055	1007.5772	504.2922	1006.5932	503.8002	E	409.1718	205.0895	392.1452	196.5763	391.1612	196.0842	3
10	1138.6467	569.8270	1121.6201	561.3137	1120.6361	560.8217	N	***280.1292***	140.5682	263.1026	132.0550			2
11							F	166.0863	83.5468					1

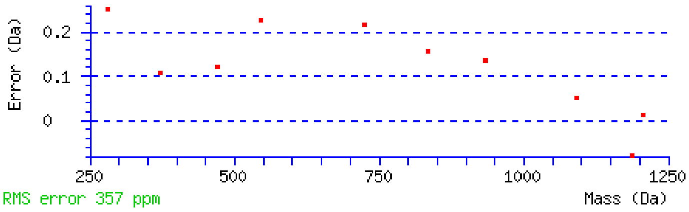

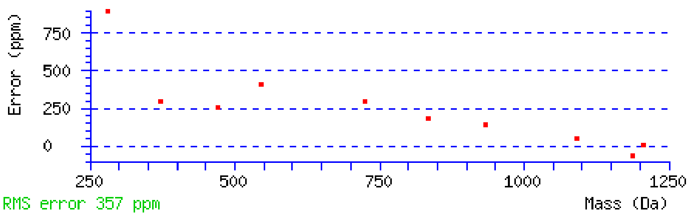

NCBI **BLAST** search of ADKVPKTAENF
(Parameters: blastp, nr protein database, expect=20000, no filter, PAM30)
Other BLAST web gateways

All matches to this query

Score	Mr(calc)	Delta	Sequence
39.8	1302.7184	0.0029	ADKVPKTAENF
33.6	1302.6905	0.0307	ADRRGLHVQSH
26.5	1302.7296	-0.0083	ADKVSSVRWIS
26.1	1302.7395	-0.0183	ADKVESGKTSVV
25.7	1302.6932	0.0280	DGGLVSNAPHIPV
24.1	1302.8163	-0.0950	ADKVLISILYI
22.6	1302.8639	-0.1427	AKVPEVPKKPV
21.7	1302.6213	0.1000	GWGCMGRLAAPV
21.7	1302.7953	-0.0741	HSIPLAHVRPV
21.7	1302.9367	-0.2154	KPKVVKVKPV

Mascot: http://www.matrixscience.com/

[redacted] Mascot Search Results

Peptide View

MS/MS Fragmentation of **ADKVPKTAENFR**
Found in **PPIA_MOUSE** in **SwissProt**, Peptidyl-prolyl cis-trans isomerase A OS=Mus musculus GN=Ppia PE=1 SV=2

Match to Query 5162: 1470.899548 from(736.457050,2+) intensity(51143.2770) scans(2908) rawscans(sn2908) rtinseconds(1323.3495) index(1267)
Title: 1268: Scan 2908 (rt=1323.35) [C:\Users\Administrador\Desktop\AMOSTRAS NO ORBITRAP LEANDRO RODAR NOVAMENTE\CORTEX\cortex_01.raw]
Data file cortex_01.temp.mgf

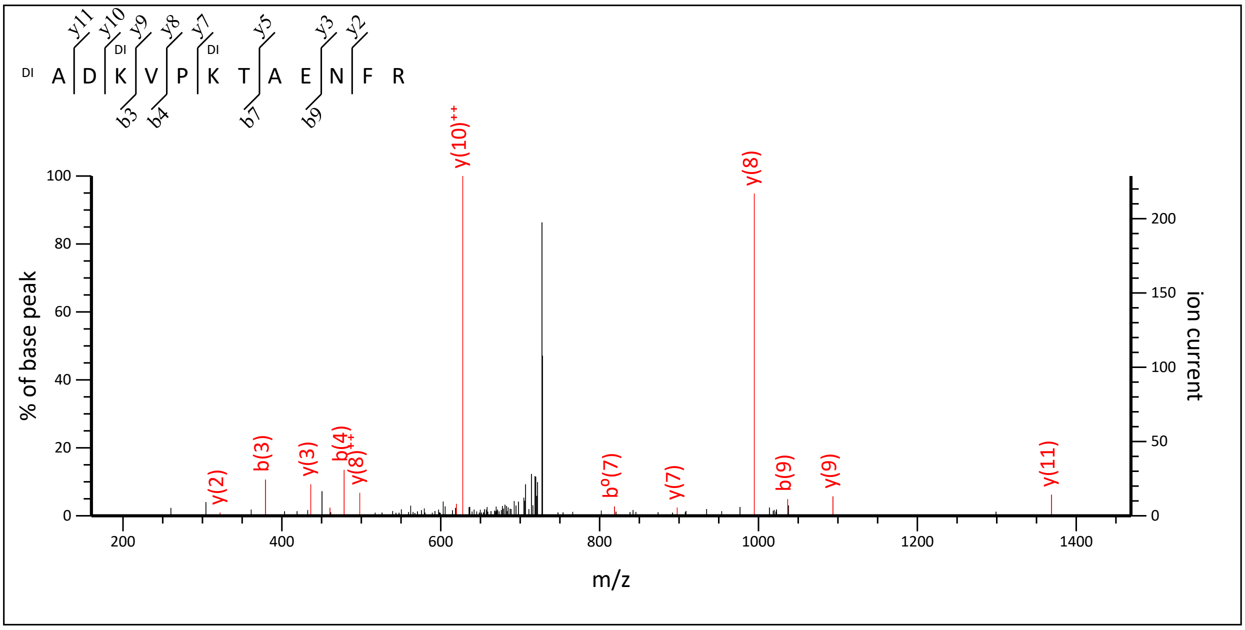

160.24 to 1468.9

Label all possible matches ○ Label matches used for scoring ◉

Monoisotopic mass of neutral peptide Mr(calc): 1470.8948
Variable modifications:
N-term : Dimethyl:2H(4) (N-term)
K3 : Dimethyl:2H(4) (K)
K6 : Dimethyl:2H(4) (K)
Ions Score: 31 **Expect:** 25
Matches : 14/120 fragment ions using 27 most intense peaks (help)

#	b	b++	b*	b*++	b0	b0++	Seq.	y	y++	y*	y*++	y0	y0++	#
1	104.1008	52.5540					A							12
2	219.1277	110.0675			201.1172	101.0622	D	1368.8085	684.9079	1351.7820	676.3946	1350.7980	675.9026	11
3	379.2791	190.1432	362.2526	181.6299	361.2685	181.1379	K	1253.7816	627.3944	1236.7550	618.8812	1235.7710	618.3892	10
4	478.3475	239.6774	461.3210	231.1641	460.3370	230.6721	V	1093.6302	547.3188	1076.6037	538.8055	1075.6197	538.3135	9
5	575.4003	288.2038	558.3737	279.6905	557.3897	279.1985	P	994.5618	497.7845	977.5353	489.2713	976.5512	488.7793	8
6	735.5517	368.2795	718.5251	359.7662	717.5411	359.2742	K	897.5090	449.2582	880.4825	440.7449	879.4985	440.2529	7
7	836.5993	418.8033	819.5728	410.2900	818.5888	409.7980	T	737.3577	369.1825	720.3311	360.6692	719.3471	360.1772	6
8	907.6365	454.3219	890.6099	445.8086	889.6259	445.3166	A	636.3100	318.6586	619.2835	310.1454	618.2994	309.6534	5
9	1036.6790	518.8432	1019.6525	510.3299	1018.6685	509.8379	E	565.2729	283.1401	548.2463	274.6268	547.2623	274.1348	4
10	1150.7220	575.8646	1133.6954	567.3513	1132.7114	566.8593	N	436.2303	218.6188	419.2037	210.1055			3
11	1297.7904	649.3988	1280.7638	640.8856	1279.7798	640.3935	F	322.1874	161.5973	305.1608	153.0840			2
12							R	175.1190	88.0631	158.0924	79.5498			1

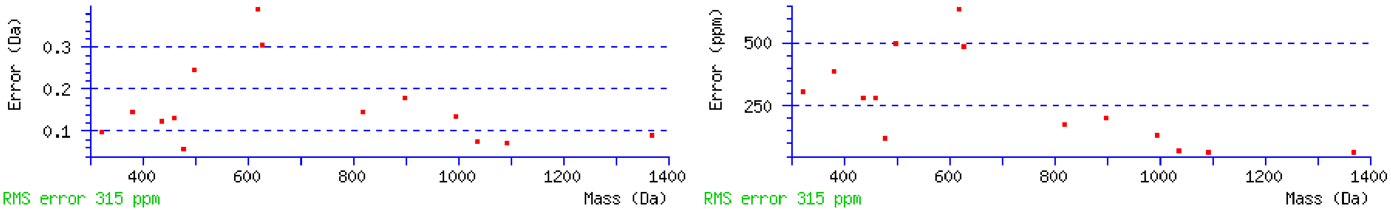

NCBI **BLAST** search of ADKVPKTAENFR
(Parameters: blastp, nr protein database, expect=20000, no filter, PAM30)
Other BLAST web gateways

All matches to this query

Score	Mr(calc)	Delta	Sequence
30.8	1470.8948	0.0048	ADKVPKTAENFR
23.8	1470.7363	0.1632	CSHLCLAVPVGGFV
21.3	1470.8208	0.0787	HRGVPKNIHFNP
20.2	1470.8475	0.0520	TQLVPPQPPISTSA
20.1	1470.8458	0.0537	AIFSLGFIIVGSIC
19.6	1470.7970	0.1026	EILLDSKPVSSW
19.1	1470.7719	0.1277	GPPGVPGLDGPKGEP
17.5	1470.9523	-0.0528	ATSSVADRKLVTK
17.3	1470.8322	0.0673	KSVAWSPSSQFLA
17.0	1470.7261	0.1735	AAFLLAMRNGCADA

Mascot: http://www.matrixscience.com/

[redacted] Mascot Search Results

Peptide View

MS/MS Fragmentation of ADKVPKTAENFRAL
Found in PPIA_MOUSE in SwissProt, Peptidyl-prolyl cis-trans isomerase A OS=Mus musculus GN=Ppia PE=1 SV=2

Match to Query 6243: 1642.945362 from(548.655730,3+) intensity(616415.2500) scans(4642) rawscans(sn4642) rtinseconds(1883.5018) index(2746)
Title: 2747: Scan 4642 (rt=1883.5) [C:\Users\Administrador\Desktop\AMOSTRAS NO ORBITRAP LEANDRO RODAR NOVAMENTE\CORTEX\cortex_01.raw]
Data file cortex_01.temp.mgf

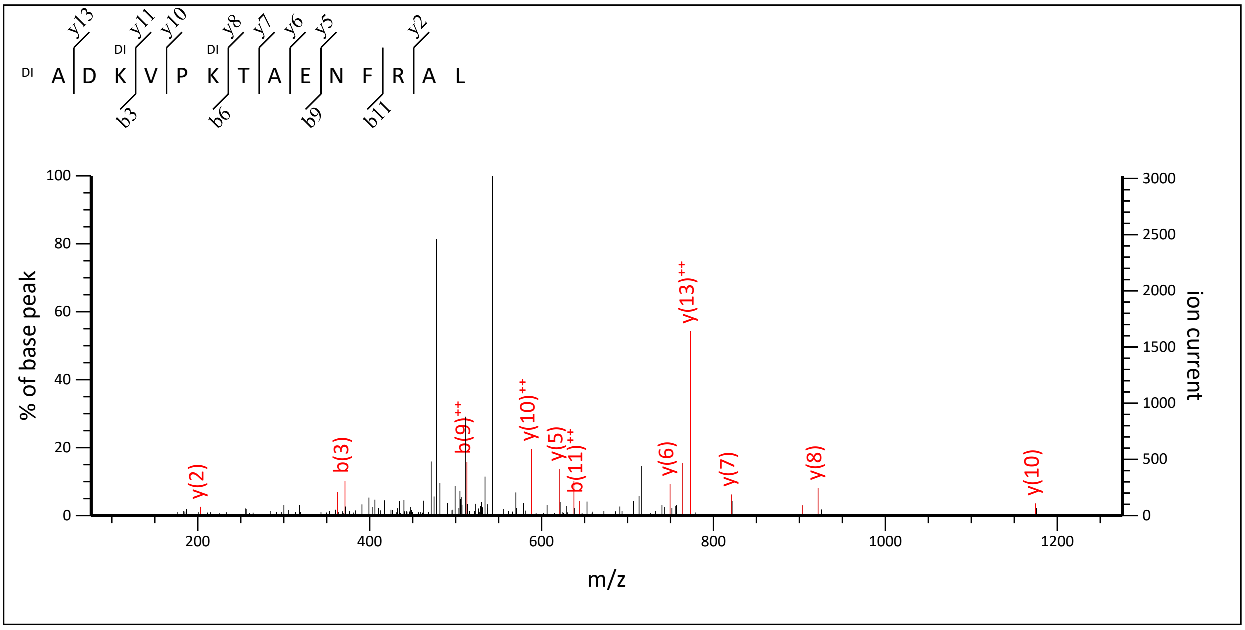

76.06 to 1275.66

Label all possible matches Label matches used for scoring

```
Monoisotopic mass of neutral peptide Mr(calc): 1642.9406
Variable modifications:
N-term : Dimethyl (N-term)
K3     : Dimethyl (K)
K6     : Dimethyl (K)
Ions Score: 22  Expect: 1.8e+002
Matches : 16/136 fragment ions using 32 most intense peaks   (help)
```

#	b	b^{++}	b*	b^{*++}	b^0	b^{0++}	Seq.	y	y^{++}	y*	y^{*++}	y^0	y^{0++}	#
1	100.0757	50.5415					A							14
2	215.1026	108.0550			197.0921	99.0497	D	1544.8795	**772.9434**	1527.8530	764.4301	1526.8689	763.9381	13
3	371.2289	186.1181	354.2023	177.6048	353.2183	177.1128	K	1429.8526	715.4299	1412.8260	706.9166	1411.8420	706.4246	12
4	470.2973	235.6523	453.2708	227.1390	452.2867	226.6470	V	1273.7263	**637.3668**	1256.6997	628.8535	1255.7157	628.3615	11
5	567.3501	284.1787	550.3235	275.6654	549.3395	275.1734	P	**1174.6579**	**587.8326**	1157.6313	579.3193	1156.6473	578.8273	10
6	723.4763	**362.2418**	706.4498	353.7285	705.4658	353.2365	K	1077.6051	539.3062	1060.5786	530.7929	1059.5946	530.3009	9
7	824.5240	412.7656	807.4975	404.2524	806.5135	403.7604	T	**921.4789**	461.2431	904.4523	452.7298	903.4683	452.2378	8
8	895.5611	448.2842	878.5346	439.7709	877.5506	439.2789	A	**820.4312**	410.7192	803.4046	402.2060	802.4206	401.7139	7
9	1024.6037	**512.8055**	1007.5772	504.2922	1006.5932	503.8002	E	**749.3941**	375.2007	732.3675	366.6874	731.3835	366.1954	6
10	1138.6467	569.8270	1121.6201	561.3137	1120.6361	560.8217	N	**620.3515**	310.6794	603.3249	302.1661			5
11	1285.7151	**643.3612**	1268.6885	634.8479	1267.7045	634.3559	F	506.3085	253.6579	489.2820	245.1446			4
12	1441.8162	721.4117	1424.7896	712.8985	1423.8056	712.4064	R	359.2401	180.1237	342.2136	171.6104			3
13	1512.8533	756.9303	1495.8267	748.4170	1494.8427	747.9250	A	**203.1390**	102.0731					2
14							L	132.1019	66.5546					1

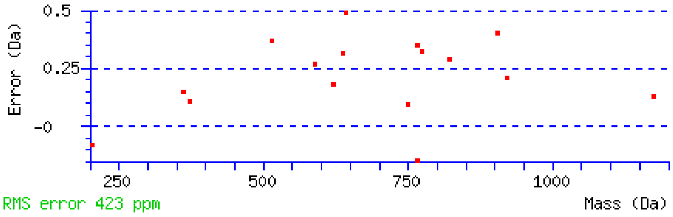

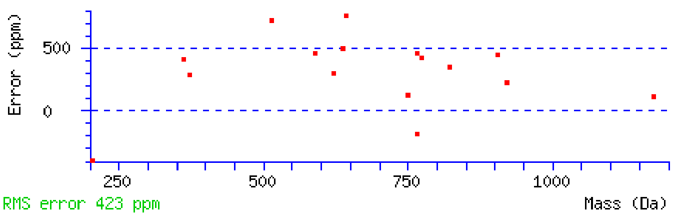

NCBI **BLAST** search of ADKVPKTAENFRAL
(Parameters: blastp, nr protein database, expect=20000, no filter, PAM30)
Other BLAST web gateways

All matches to this query

Score	Mr(calc)	Delta	Sequence
22.4	1642.9406	0.0047	ADKVPKTAENFRAL
15.5	1642.9082	0.0371	ANKNYYGAAKLIFS
13.1	1642.8501	0.0953	CAPANHTPALPSINPL
12.9	1642.7549	0.1905	EFGKYCDDIVNTAA
12.5	1642.8395	0.1058	FFFSLAAVNPNFTV
11.7	1642.7439	0.2015	DHLPGGESQGCPGQPS
11.4	1642.8463	0.0991	LFGYGVLDAGAMVKM
11.1	1642.8342	0.1112	PLTEVIEPLDFEDV
11.1	1642.8050	0.1404	ADPSAQSEGEKVADLA
10.9	1642.7303	0.2151	RMNMPISNEDMTV

Mascot: http://www.matrixscience.com/

[redacted] Mascot Search Results

Peptide View

MS/MS Fragmentation of **EDENFILKHTGPGILSM**
Found in **PPIA_MOUSE** in **SwissProt**, Peptidyl-prolyl cis-trans isomerase A OS=Mus musculus GN=Ppia PE=1 SV=2

Match to Query 8035: 1956.012148 from(979.013350,2+) intensity(38344.8750) scans(10101) rawscans(sn10101) rtinseconds(3802.4217) index(7458)
Title: 7459: Scan 10101 (rt=3802.42) [C:\Users\Administrador\Desktop\AMOSTRAS NO ORBITRAP LEANDRO RODAR NOVAMENTE\CORTEX\cortex_01.raw]
Data file cortex_01.temp.mgf

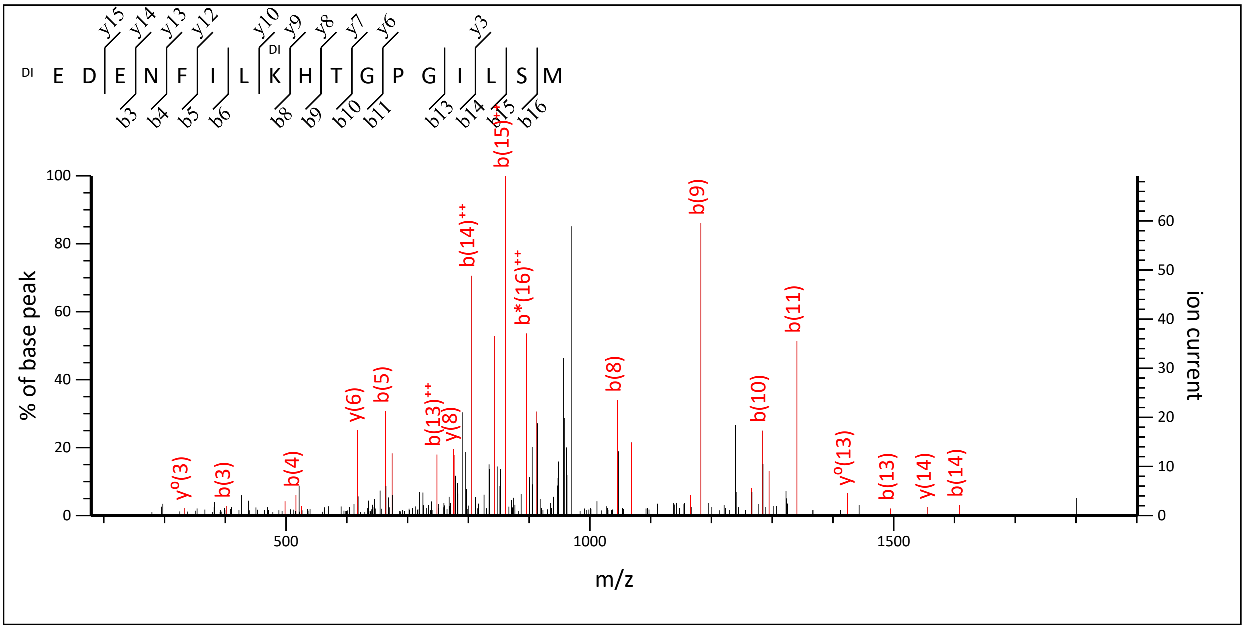

179.29 to 1901.21

Label all possible matches ○ Label matches used for scoring ◉

Monoisotopic mass of neutral peptide Mr(calc): 1956.0026
Variable modifications:
N-term : Dimethyl (N-term)
K8 : Dimethyl (K)
Ions Score: 57 **Expect:** 0.068
Matches : 30/166 fragment ions using 51 most intense peaks (help)

#	b	b^{++}	b*	b^{*++}	b^0	b^{0++}	Seq.	y	y^{++}	y*	y^{*++}	y^0	y^{0++}	#
1	158.0812	79.5442			140.0706	70.5389	E							17
2	273.1081	137.0577			255.0975	128.0524	D	1799.9360	900.4717	1782.9095	891.9584	1781.9255	891.4664	16
3	402.1507	201.5790			384.1401	192.5737	E	1684.9091	842.9582	1667.8825	834.4449	1666.8985	833.9529	15
4	516.1936	258.6005	499.1671	250.0872	498.1831	249.5952	N	1555.8665	778.4369	1538.8399	769.9236	1537.8559	769.4316	14
5	663.2620	332.1347	646.2355	323.6214	645.2515	323.1294	F	1441.8236	721.4154	1424.7970	712.9021	1423.8130	712.4101	13
6	776.3461	388.6767	759.3196	380.1634	758.3355	379.6714	I	1294.7552	647.8812	1277.7286	639.3679	1276.7446	638.8759	12
7	889.4302	445.2187	872.4036	436.7055	871.4196	436.2134	L	1181.6711	591.3392	1164.6445	582.8259	1163.6605	582.3339	11
8	1045.5564	523.2819	1028.5299	514.7686	1027.5459	514.2766	K	1068.5870	534.7972	1051.5605	526.2839	1050.5765	525.7919	10
9	1182.6153	591.8113	1165.5888	583.2980	1164.6048	582.8060	H	912.4608	456.7340			894.4502	447.7287	9
10	1283.6630	642.3352	1266.6365	633.8219	1265.6525	633.3299	T	775.4019	388.2046			757.3913	379.1993	8
11	1340.6845	670.8459	1323.6579	662.3326	1322.6739	661.8406	G	674.3542	337.6807			656.3436	328.6754	7
12	1437.7373	719.3723	1420.7107	710.8590	1419.7267	710.3670	P	617.3327	309.1700			599.3221	300.1647	6
13	1494.7587	747.8830	1477.7322	739.3697	1476.7482	738.8777	G	520.2799	260.6436			502.2694	251.6383	5
14	1607.8428	804.4250	1590.8162	795.9118	1589.8322	795.4197	I	463.2585	232.1329			445.2479	223.1276	4
15	1720.9268	860.9671	1703.9003	852.4538	1702.9163	851.9618	L	350.1744	175.5908			332.1639	166.5856	3
16	1807.9589	904.4831	1790.9323	895.9698	1789.9483	895.4778	S	237.0904	119.0488			219.0798	110.0435	2
17							M	150.0583	75.5328					1

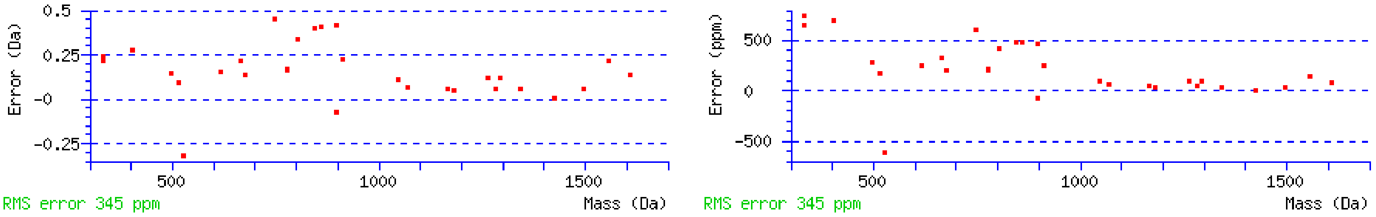

NCBI **BLAST** search of EDENFILKHTGPGILSM
(Parameters: blastp, nr protein database, expect=20000, no filter, PAM30)
Other BLAST web gateways

All matches to this query

Score	Mr(calc)	Delta	Sequence
56.8	1956.0026	0.0095	EDENFILKHTGPGILSM
20.0	1955.9375	0.0747	CDVNHQNRAGYSALMLA
18.3	1955.9741	0.0380	WNSGSLSSGVHTFPAVLQ
18.2	1956.0608	-0.0487	APLPAPGTSALFTFSPLTV
17.3	1955.8801	0.1321	DAREQEIRMGQMAMGGA
14.1	1956.1913	-0.1792	TVLLAPRQPQSLIVTER
13.3	1956.2739	-0.2618	PLPTATTGNVHTAKKISI
12.7	1955.8306	0.1816	LLEMDSFSSEDPRDTE
12.3	1956.0793	-0.0671	FINATTGIVTVNRPLDR
12.1	1955.9193	0.0928	QASGNMGTNPPTMTRVPGP

Mascot: http://www.matrixscience.com/

[redacted] Mascot Search Results

Peptide View

MS/MS Fragmentation of **ELFADKVPKTAENFRAL**
Found in **PPIA_MOUSE** in **SwissProt**, Peptidyl-prolyl cis-trans isomerase A OS=Mus musculus GN=Ppia PE=1 SV=2

Match to Query 8470: 2032.131582 from(678.384470,3+) intensity(105163.0900) scans(8156) rawscans(sn8156) rtinseconds(3115.0489) index(5804)
Title: 5805: Scan 8156 (rt=3115.05) [C:\Users\Administrador\Desktop\AMOSTRAS NO ORBITRAP LEANDRO RODAR NOVAMENTE\CORTEX\cortex_01.raw]
Data file cortex_01.temp.mgf

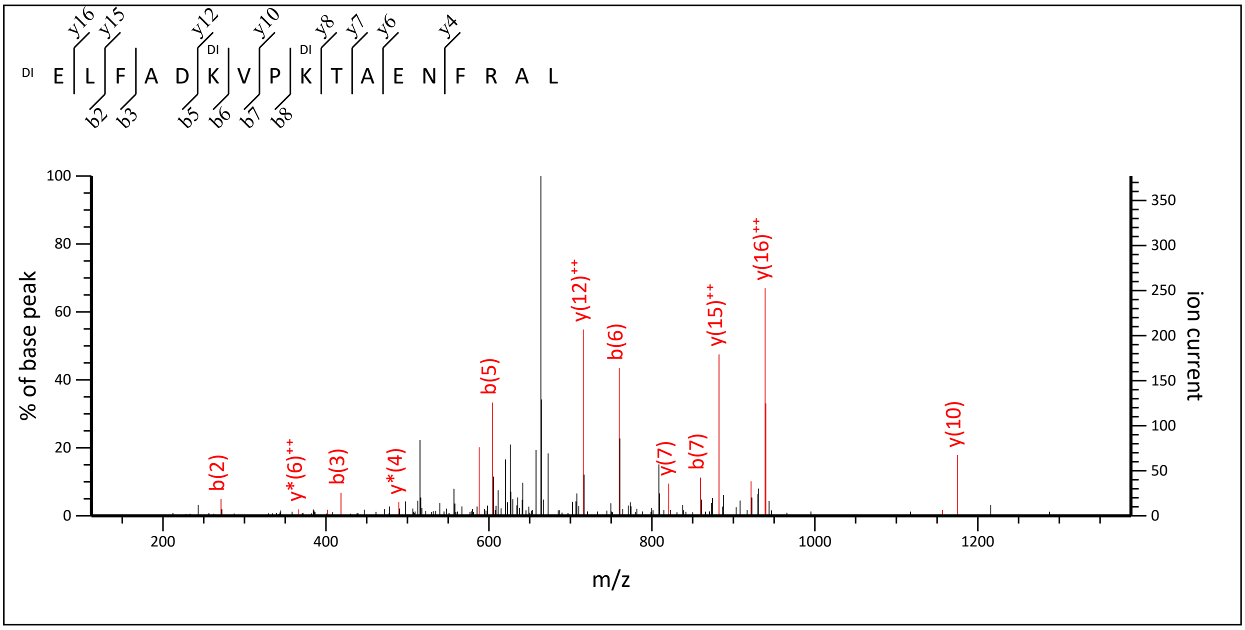

112.17 to 1387.89

Label all possible matches ○ Label matches used for scoring ◉

Monoisotopic mass of neutral peptide Mr(calc): 2032.1357
Variable modifications:
N-term : Dimethyl (N-term)
K6 : Dimethyl (K)
K9 : Dimethyl (K)
Ions Score: 29 **Expect:** 40
Matches : 21/168 fragment ions using 29 most intense peaks (help)

#	b	b++	b*	b*++	b0	b0++	Seq.	y	y++	y*	y*++	y0	y0++	#
1	158.0812	79.5442			140.0706	70.5389	E							17
2	271.1652	136.0863			253.1547	127.0810	L	1876.0691	938.5382	1859.0425	930.0249	1858.0585	929.5329	16
3	418.2336	209.6205			400.2231	200.6152	F	1762.9850	881.9962	1745.9585	873.4829	1744.9745	872.9909	15
4	489.2708	245.1390			471.2602	236.1337	A	1615.9166	808.4619	1598.8901	799.9487	1597.9061	799.4567	14
5	604.2977	302.6525			586.2871	293.6472	D	1544.8795	772.9434	1527.8530	764.4301	1526.8689	763.9381	13
6	760.4240	380.7156	743.3974	372.2023	742.4134	371.7103	K	1429.8526	715.4299	1412.8260	706.9166	1411.8420	706.4246	12
7	859.4924	430.2498	842.4658	421.7366	841.4818	421.2445	V	1273.7263	637.3668	1256.6997	628.8535	1255.7157	628.3615	11
8	956.5451	478.7762	939.5186	470.2629	938.5346	469.7709	P	1174.6579	587.8326	1157.6313	579.3193	1156.6473	578.8273	10
9	1112.6714	556.8393	1095.6449	548.3261	1094.6608	547.8341	K	1077.6051	539.3062	1060.5786	530.7929	1059.5946	530.3009	9
10	1213.7191	607.3632	1196.6925	598.8499	1195.7085	598.3579	T	921.4789	461.2431	904.4523	452.7298	903.4683	452.2378	8
11	1284.7562	642.8817	1267.7297	634.3685	1266.7456	633.8765	A	820.4312	410.7192	803.4046	402.2060	802.4206	401.7139	7
12	1413.7988	707.4030	1396.7722	698.8898	1395.7882	698.3978	E	749.3941	375.2007	732.3675	366.6874	731.3835	366.1954	6
13	1527.8417	764.4245	1510.8152	755.9112	1509.8312	755.4192	N	620.3515	310.6794	603.3249	302.1661			5
14	1674.9101	837.9587	1657.8836	829.4454	1656.8996	828.9534	F	506.3085	253.6579	489.2820	245.1446			4
15	1831.0112	916.0093	1813.9847	907.4960	1813.0007	907.0040	R	359.2401	180.1237	342.2136	171.6104			3
16	1902.0484	951.5278	1885.0218	943.0145	1884.0378	942.5225	A	203.1390	102.0731					2
17							L	132.1019	66.5546					1

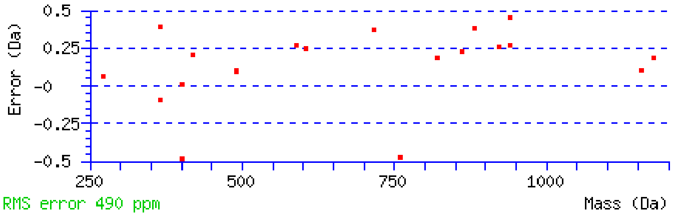

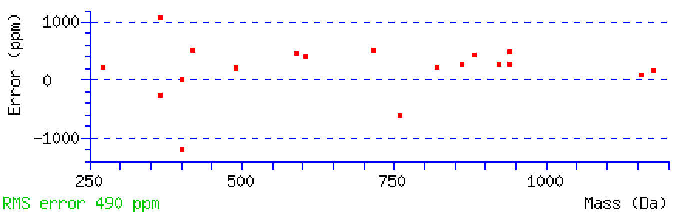

NCBI **BLAST** search of ELFADKVPKTAENFRAL
(Parameters: blastp, nr protein database, expect=20000, no filter, PAM30)
Other BLAST web gateways

All matches to this query

Score	Mr(calc)	Delta	Sequence
29.1	2032.1357	-0.0041	ELFADKVPKTAENFRAL
19.4	2032.1834	-0.0518	SLLHHGGFKVLQSLKGSV
17.6	2032.0561	0.0755	TQNADCVVVLDNTALNRI
17.0	2032.0486	0.0830	HSLLGPILSCVAVCEVIDH
17.0	2032.0661	0.0654	SVSVARSSGNSGGLGRISGIGS
16.6	2031.9935	0.1381	EDVENLIHVQIEAMIDH
15.7	2031.9281	0.2035	EALLYCNIPSVAEHSMEG
15.4	2032.0088	0.1228	GANVSFQCETKGNPPPAIF
14.9	2032.1066	0.0250	EWGKDTARRLGSTTVGSK
14.8	2032.0854	0.0462	SLPVHVRENLWEVAGRT

Mascot: http://www.matrixscience.com/

[redacted] Mascot Search Results

Peptide View

MS/MS Fragmentation of **ELFADKVPKTAENF**
Found in **PPIA_MOUSE** in **SwissProt**, Peptidyl-prolyl cis-trans isomerase A OS=Mus musculus GN=Ppia PE=1 SV=2

Match to Query 6637: 1703.953268 from(852.983910,2+) intensity(16920.4360) scans(7272) rawscans(sn7272) rtinseconds(2795.1865) index(5073)
Title: 5074: Scan 7272 (rt=2795.19) [C:\Users\Administrador\Desktop\AMOSTRAS NO ORBITRAP LEANDRO RODAR NOVAMENTE\CORTEX\cortex_01.raw]
Data file cortex_01.temp.mgf

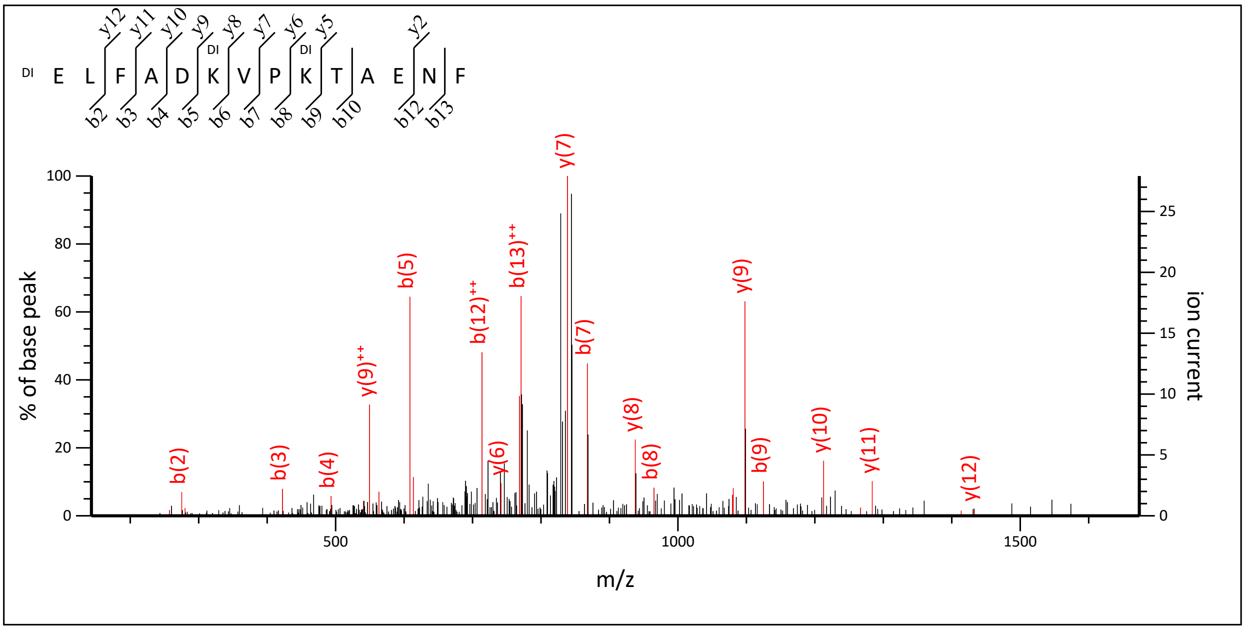

143.3 to 1673.93

Label all possible matches ○ Label matches used for scoring ◉

```
Monoisotopic mass of neutral peptide Mr(calc): 1703.9887
Variable modifications:
N-term : Dimethyl:2H(4) (N-term)
K6     : Dimethyl:2H(4) (K)
K9     : Dimethyl:2H(4) (K)
Ions Score: 34  Expect: 12
Matches : 29/140 fragment ions using 63 most intense peaks   (help)
```

#	b	b++	b*	b*++	b0	b0++	Seq.	y	y++	y*	y*++	y0	y0++	#
1	162.1063	81.5568			144.0957	72.5515	E							14
2	275.1903	138.0988			257.1798	129.0935	L	1543.8970	772.4521	1526.8705	763.9389	1525.8865	763.4469	13
3	422.2588	211.6330			404.2482	202.6277	F	1430.8130	715.9101	1413.7864	707.3968	1412.8024	706.9048	12
4	493.2959	247.1516			475.2853	238.1463	A	1283.7445	642.3759	1266.7180	633.8626	1265.7340	633.3706	11
5	608.3228	304.6650			590.3122	295.6598	D	1212.7074	606.8574	1195.6809	598.3441	1194.6969	597.8521	10
6	768.4742	384.7407	751.4476	376.2275	750.4636	375.7354	K	1097.6805	549.3439	1080.6539	540.8306	1079.6699	540.3386	9
7	867.5426	434.2749	850.5160	425.7617	849.5320	425.2697	V	937.5291	469.2682	920.5026	460.7549	919.5186	460.2629	8
8	964.5954	482.8013	947.5688	474.2880	946.5848	473.7960	P	838.4607	419.7340	821.4342	411.2207	820.4501	410.7287	7
9	1124.7467	562.8770	1107.7202	554.3637	1106.7362	553.8717	K	741.4079	371.2076	724.3814	362.6943	723.3974	362.2023	6
10	1225.7944	613.4008	1208.7679	604.8876	1207.7838	604.3956	T	581.2566	291.1319	564.2300	282.6186	563.2460	282.1266	5
11	1296.8315	648.9194	1279.8050	640.4061	1278.8210	639.9141	A	480.2089	240.6081	463.1823	232.0948	462.1983	231.6028	4
12	1425.8741	713.4407	1408.8476	704.9274	1407.8636	704.4354	E	409.1718	205.0895	392.1452	196.5763	391.1612	196.0842	3
13	1539.9170	770.4622	1522.8905	761.9489	1521.9065	761.4569	N	280.1292	140.5682	263.1026	132.0550			2
14							F	166.0863	83.5468					1

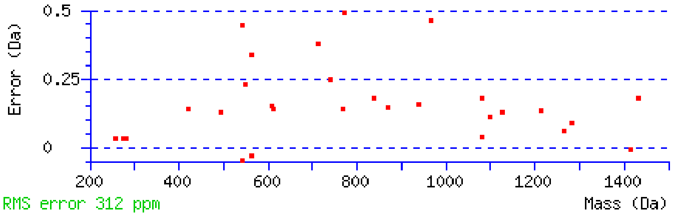

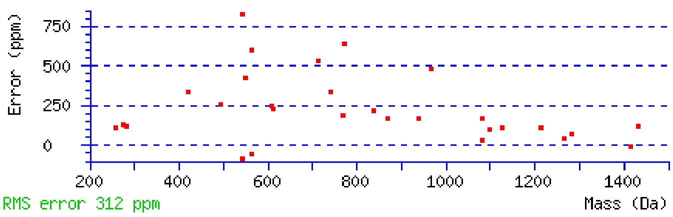

NCBI **BLAST** search of ELFADKVPKTAENF
(Parameters: blastp, nr protein database, expect=20000, no filter, PAM30)
Other BLAST web gateways

All matches to this query

Score	Mr(calc)	Delta	Sequence
34.2	1703.9887	-0.0355	ELFADKVPKTAENF
19.6	1703.8687	0.0846	GQDFQCRETGRCLK
17.5	1703.9375	0.0158	VITFDKGGTWEFLQ
17.4	1703.9959	-0.0427	EIAKEHDLPDAKSR
16.4	1703.8565	0.0968	SWSSQNVLMFMTLL
15.7	1703.9591	-0.0058	QGCISKDKVDTLIM
15.7	1703.8528	0.1005	DLQKMSDAAGDFVDI
15.0	1703.8930	0.0603	SIAATDHEPTDARKS
14.2	1703.9259	0.0274	SFIINLFVMAVFGQA
13.8	1703.8781	0.0752	IFSADRPNVQDQLQ

Mascot: http://www.matrixscience.com/

[redacted] Mascot Search Results

Peptide View

MS/MS Fragmentation of **ITADDEPLGRVSF**
Found in **PPIA_MOUSE** in **SwissProt**, Peptidyl-prolyl cis-trans isomerase A OS=Mus musculus GN=Ppia PE=1 SV=2

Match to Query 4721: 1450.766908 from(726.390730,2+) intensity(48205.5000) scans(8533) rawscans(sn8533) rtinseconds(3217.248) index(6069)
Title: 6070: Scan 8533 (rt=3217.25) [C:\Users\Administrador\Desktop\AMOSTRAS NO ORBITRAP LEANDRO RODAR NOVAMENTE\HIPOCAMPO\hipocampo_01.raw]
Data file hipocampo_01.temp.mgf

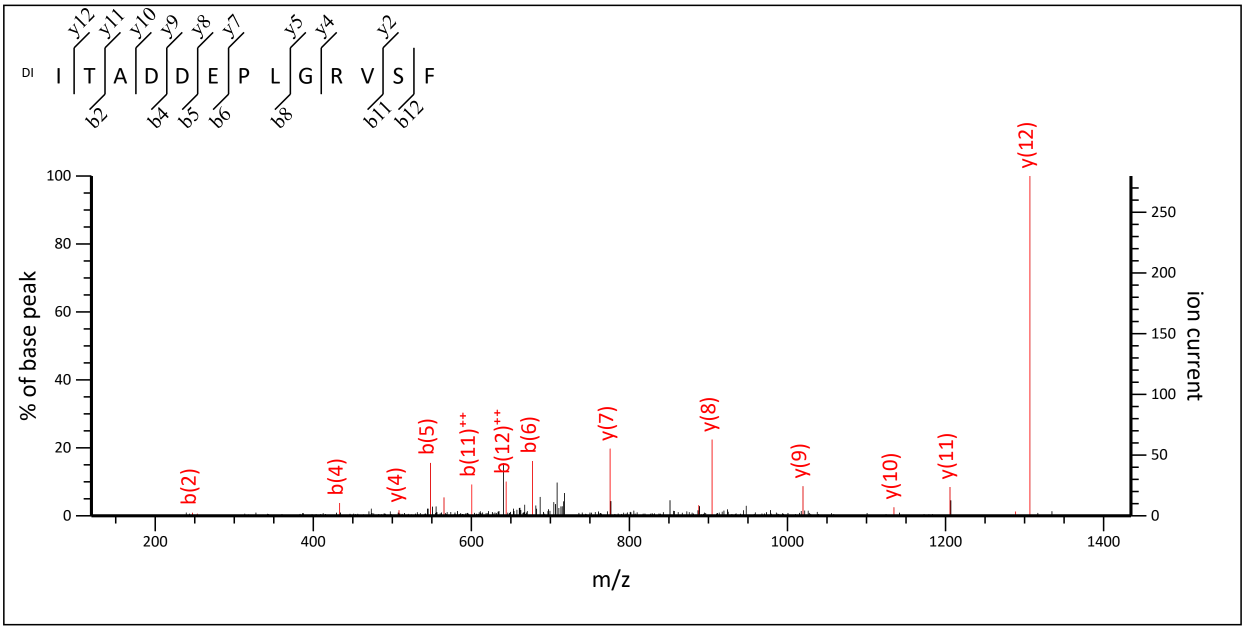

119.26 to 1434.61

Label all possible matches ○ Label matches used for scoring ◉

Monoisotopic mass of neutral peptide Mr(calc): 1450.7606
Variable modifications:
N-term : Dimethyl:2H(4) (N-term)
Ions Score: 55 **Expect:** 0.098
Matches : 19/116 fragment ions using 34 most intense peaks (help)

#	b	b^{++}	b*	b^{*++}	b^0	b^{0++}	Seq.	y	y^{++}	y*	y^{*++}	y^0	y^{0++}	#
1	146.1477	73.5775					I							13
2	**247.1954**	124.1014			229.1849	115.0961	T	***1306.6274***	653.8173	1289.6008	645.3040	1288.6168	644.8120	12
3	318.2325	159.6199			300.2220	150.6146	A	***1205.5797***	603.2935	1188.5531	594.7802	1187.5691	594.2882	11
4	**433.2595**	217.1334			415.2489	208.1281	D	***1134.5426***	567.7749	1117.5160	559.2617	1116.5320	558.7696	10
5	**548.2864**	274.6469			530.2759	265.6416	D	***1019.5156***	510.2615	1002.4891	501.7482	1001.5051	501.2562	9
6	**677.3290**	339.1681			659.3185	330.1629	E	***904.4887***	452.7480	887.4621	444.2347	886.4781	443.7427	8
7	774.3818	387.6945			756.3712	378.6892	P	***775.4461***	388.2267	758.4196	379.7134	757.4355	379.2214	7
8	**887.4658**	444.2366			869.4553	435.2313	L	678.3933	339.7003	661.3668	331.1870	660.3828	330.6950	6
9	944.4873	472.7473			926.4767	463.7420	G	***565.3093***	283.1583	548.2827	274.6450	547.2987	274.1530	5
10	1100.5884	550.7978	1083.5619	542.2846	1082.5779	541.7926	R	***508.2878***	254.6475	491.2613	246.1343	490.2772	245.6423	4
11	1199.6568	**600.3321**	1182.6303	591.8188	1181.6463	591.3268	V	352.1867	176.5970			334.1761	167.5917	3
12	1286.6889	**643.8481**	1269.6623	635.3348	1268.6783	634.8428	S	***253.1183***	127.0628			235.1077	118.0575	2
13							F	166.0863	83.5468					1

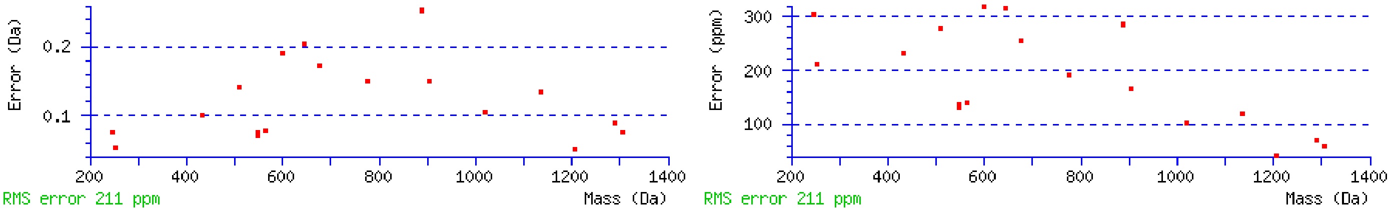

NCBI **BLAST** search of ITADDEPLGRVSF
(Parameters: blastp, nr protein database, expect=20000, no filter, PAM30)
Other BLAST web gateways

All matches to this query

Score	Mr(calc)	Delta	Sequence
54.9	1450.7606	0.0063	ITADDEPLGRVSF
29.0	1450.7672	-0.0003	MAGHDEAVLFVQV
28.4	1450.8316	-0.0647	GCQLIKFDSVEV
26.7	1450.7809	-0.0140	VIRTRAEDEGDL
25.3	1450.7250	0.0419	LMWNREDALMV
24.8	1450.7730	-0.0061	IFKANHPMDAEV
22.9	1450.8119	-0.0450	LEGKANGGLVDSEV
22.3	1450.7282	0.0387	LTYPPGGWDEVSV
21.1	1450.7765	-0.0096	AVRFRAMPRGNS
21.1	1450.8231	-0.0562	LAESDEAKVQRAA

Mascot: http://www.matrixscience.com/

[redacted] Mascot Search Results

Peptide View

MS/MS Fragmentation of **LFADKVPKTAENF**
Found in **PPIA_MOUSE** in **SwissProt**, Peptidyl-prolyl cis-trans isomerase A OS=Mus musculus GN=Ppia PE=1 SV=2

Match to Query 5336: 1562.875962 from(521.965930,3+) intensity(66458.7810) scans(6289) rawscans(sn6289) rtinseconds(2422.4935) index(4162)
Title: 4163: Scan 6289 (rt=2422.49) [C:\Users\Administrador\Desktop\AMOSTRAS NO ORBITRAP LEANDRO RODAR NOVAMENTE\HIPOCAMPO\hipocampo_01.raw]
Data file hipocampo_01.temp.mgf

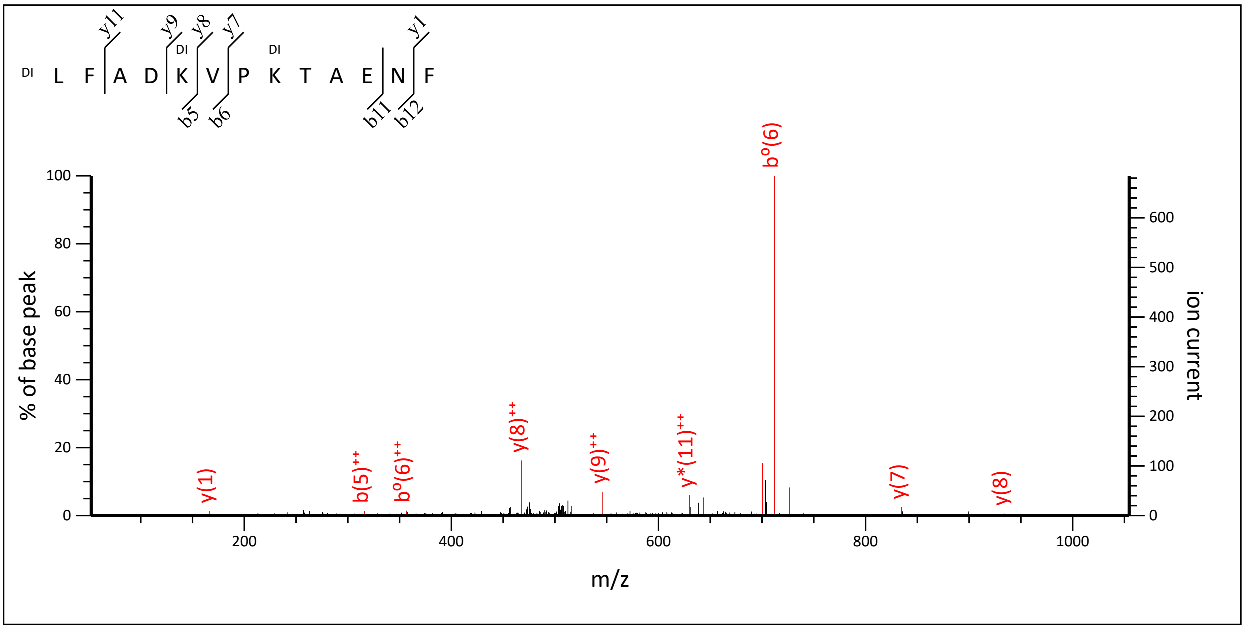

52.14 to 1054.58

Label all possible matches ○ Label matches used for scoring ◉

Monoisotopic mass of neutral peptide Mr(calc): 1562.8708
Variable modifications:
N-term : Dimethyl (N-term)
K5 : Dimethyl (K)
K8 : Dimethyl (K)
Ions Score: 20 **Expect:** 2.9e+002
Matches : 12/124 fragment ions using 17 most intense peaks (help)

#	b	b^{++}	b*	b^{*++}	b^0	b^{0++}	Seq.	y	y^{++}	y*	y^{*++}	y^0	y^{0++}	#
1	142.1226	71.5650					L							13
2	289.1911	145.0992					F	1422.7627	***711.8850***	1405.7362	703.3717	1404.7522	702.8797	12
3	360.2282	180.6177					A	1275.6943	638.3508	1258.6678	629.8375	1257.6838	629.3455	11
4	475.2551	238.1312			457.2445	229.1259	D	1204.6572	602.8322	1187.6307	594.3190	1186.6466	593.8270	10
5	631.3814	***316.1943***	614.3548	307.6811	613.3708	307.1890	K	1089.6303	***545.3188***	1072.6037	536.8055	1071.6197	536.3135	9
6	730.4498	365.7285	713.4232	357.2153	712.4392	356.7232	V	***933.5040***	***467.2556***	916.4775	458.7424	915.4934	458.2504	8
7	827.5026	414.2549	810.4760	405.7416	809.4920	405.2496	P	***834.4356***	417.7214	817.4090	409.2082	816.4250	408.7162	7
8	983.6288	492.3180	966.6023	483.8048	965.6182	483.3128	K	737.3828	369.1951	720.3563	360.6818	719.3723	360.1898	6
9	1084.6765	542.8419	1067.6499	534.3286	1066.6659	533.8366	T	581.2566	291.1319	564.2300	282.6186	563.2460	282.1266	5
10	1155.7136	578.3604	1138.6871	569.8472	1137.7030	569.3552	A	480.2089	240.6081	463.1823	232.0948	462.1983	231.6028	4
11	1284.7562	***642.8817***	1267.7297	634.3685	1266.7456	633.8765	E	409.1718	205.0895	392.1452	196.5763	391.1612	196.0842	3
12	1398.7991	***699.9032***	1381.7726	691.3899	1380.7886	690.8979	N	280.1292	140.5682	263.1026	132.0550			2
13							F	***166.0863***	83.5468					1

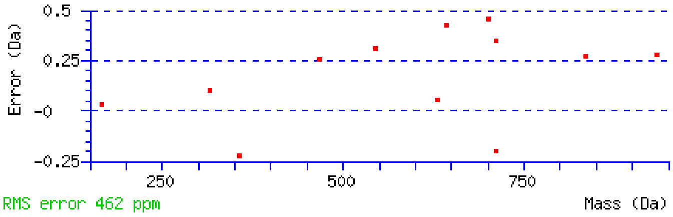

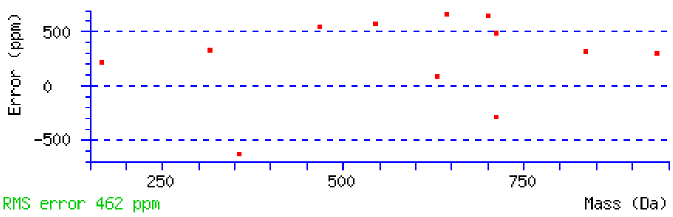

NCBI **BLAST** search of LFADKVPKTAENF
(Parameters: blastp, nr protein database, expect=20000, no filter, PAM30)
Other BLAST web gateways

All matches to this query

Score	Mr(calc)	Delta	Sequence
20.2	1562.8708	0.0051	LFADKVPKTAENF
19.6	1562.8549	0.0211	QGMLDDKGRVIED
18.7	1562.7952	0.0807	EPGANLDQPVTMIF
18.5	1562.8273	0.0487	AMDAKVGSLRRVM
17.2	1563.0657	-0.1897	SPAGPSHKGKKAVN
17.0	1562.7183	0.1577	LLAAFSVDCCLAYM
16.4	1562.7861	0.0898	EKKAEELTQTNM
15.7	1562.9355	-0.0595	LFAGSSLGKRGVNAD
15.4	1562.8966	-0.0207	LACLLTQYRVSLR
15.0	1562.7974	0.0786	LDDGSNMTELIRK

Mascot: http://www.matrixscience.com/

[redacted] Mascot Search Results

Peptide View

MS/MS Fragmentation of **LFADKVPKTAENFRAL**
Found in **PPIA_MOUSE** in **SwissProt**, Peptidyl-prolyl cis-trans isomerase A OS=Mus musculus GN=Ppia PE=1 SV=2

Match to Query 7801: 1915.174002 from(639.398610,3+) intensity(25260.8220) scans(6886) rawscans(sn6886) rtinseconds(2655.6959) index(4749)
Title: 4750: Scan 6886 (rt=2655.7) [C:\Users\Administrador\Desktop\AMOSTRAS NO ORBITRAP LEANDRO RODAR NOVAMENTE\CORTEX\cortex_01.raw]
Data file cortex_01.temp.mgf

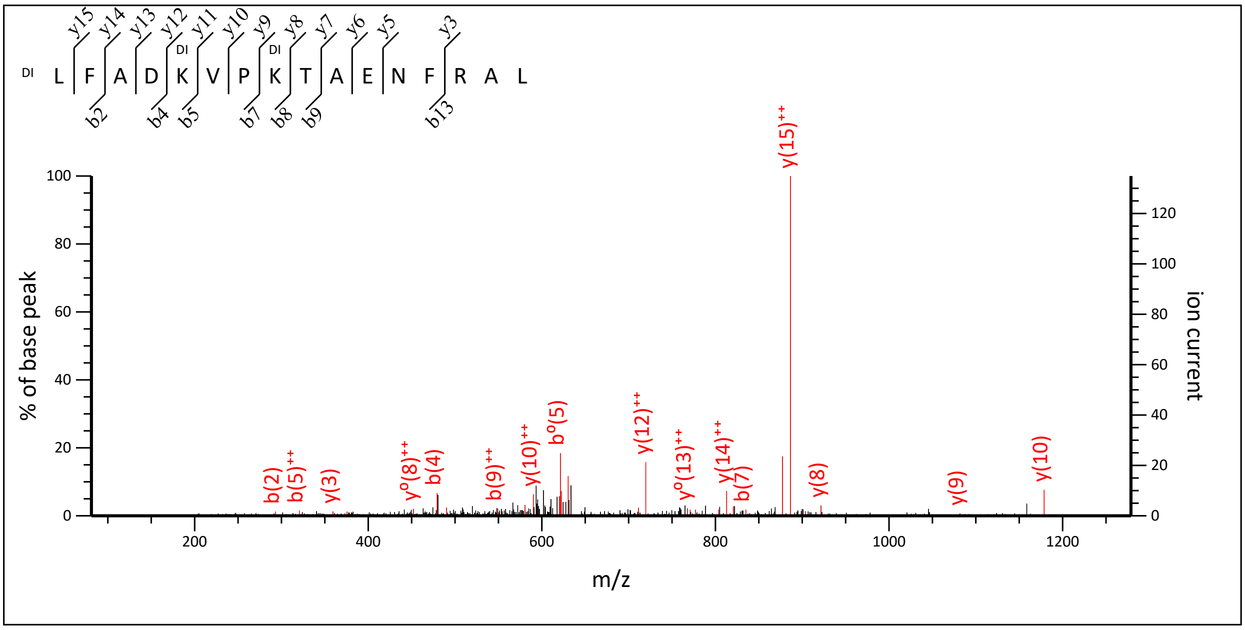

81.08 to 1278.73

Label all possible matches ○ Label matches used for scoring ◉

```
Monoisotopic mass of neutral peptide Mr(calc): 1915.1684
Variable modifications:
N-term : Dimethyl:2H(4) (N-term)
K5     : Dimethyl:2H(4) (K)
K8     : Dimethyl:2H(4) (K)
Ions Score: 19  Expect: 4.2e+002
Matches : 33/152 fragment ions using 97 most intense peaks   (help)
```

#	b	b^{++}	b*	b^{*++}	b^0	b^{0++}	Seq.	y	y^{++}	y*	y^{*++}	y^0	y^{0++}	#
1	146.1477	73.5775					L							16
2	**293.2162**	147.1117					F	1771.0352	***886.0213***	1754.0087	877.5080	1753.0247	877.0160	15
3	364.2533	182.6303					A	1623.9668	***812.4871***	1606.9403	803.9738	1605.9563	803.4818	14
4	**479.2802**	240.1437			461.2697	231.1385	D	1552.9297	***776.9685***	1535.9032	768.4552	1534.9192	767.9632	13
5	639.4316	320.2194	622.4050	311.7062	621.4210	311.2141	K	1437.9028	***719.4550***	1420.8762	710.9418	1419.8922	710.4497	12
6	738.5000	369.7536	721.4735	361.2404	720.4894	360.7484	V	1277.7514	639.3793	1260.7249	630.8661	1259.7408	630.3741	11
7	**835.5528**	418.2800	818.5262	409.7667	817.5422	409.2747	P	***1178.6830***	***589.8451***	1161.6564	581.3319	1160.6724	580.8399	10
8	995.7041	498.3557	978.6776	489.8424	977.6936	489.3504	K	***1081.6302***	541.3188	1064.6037	532.8055	1063.6197	532.3135	9
9	1096.7518	548.8795	1079.7253	540.3663	1078.7413	539.8743	T	***921.4789***	461.2431	904.4523	452.7298	903.4683	452.2378	8
10	1167.7889	584.3981	1150.7624	575.8848	1149.7784	575.3928	A	***820.4312***	410.7192	803.4046	402.2060	802.4206	401.7139	7
11	1296.8315	648.9194	1279.8050	640.4061	1278.8210	639.9141	E	749.3941	***375.2007***	732.3675	366.6874	731.3835	366.1954	6
12	1410.8744	705.9409	1393.8479	697.4276	1392.8639	696.9356	N	***620.3515***	310.6794	603.3249	302.1661			5
13	1557.9429	779.4751	1540.9163	770.9618	1539.9323	770.4698	F	506.3085	253.6579	489.2820	245.1446			4
14	1714.0440	857.5256	1697.0174	849.0124	1696.0334	848.5203	R	***359.2401***	180.1237	342.2136	171.6104			3
15	1785.0811	893.0442	1768.0545	884.5309	1767.0705	884.0389	A	203.1390	102.0731					2
16							L	132.1019	66.5546					1

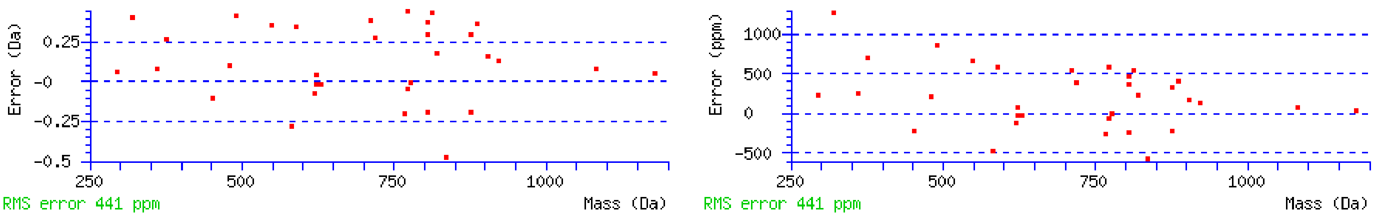

NCBI **BLAST** search of LFADKVPKTAENFRAL
(Parameters: blastp, nr protein database, expect=20000, no filter, PAM30)
Other BLAST web gateways

All matches to this query

Score	Mr(calc)	Delta	Sequence
18.9	1915.1684	0.0056	LFADKVPKTAENFRAL
16.1	1915.0903	0.0837	MRGTPVLVALGDFLGGPLA
14.8	1915.1146	0.0594	ADAFYKAKNEFAAKAT
14.1	1915.0477	0.1263	AEVLAARGYLTGMAGKW
13.3	1915.0940	0.0800	ADSTEMGDKATAGISKPL
11.7	1914.8892	0.2848	AFGEAALEQTLAEMCEL
11.4	1915.0430	0.1310	AIGYSETAVDPTLPKTF
11.2	1914.9823	0.1917	ATMTSHDLMKFVAPFN
11.2	1915.0470	0.1270	ITECSSLKRSLEQARM
11.2	1914.9547	0.2193	LTCESLEPVVVDLTHND

Mascot: http://www.matrixscience.com/

[redacted] Mascot Search Results

Peptide View

MS/MS Fragmentation of **FDITADDEPLGRVSF**
Found in **PPIA_MOUSE** in **SwissProt**, Peptidyl-prolyl cis-trans isomerase A OS=Mus musculus GN=Ppia PE=1 SV=2

Match to Query 6309: 1712.868048 from(857.441300,2+) intensity(15595.4550) scans(10691) rawscans(sn10691) rtinseconds(4046.8744) index(7877)
Title: 7878: Scan 10691 (rt=4046.87) [C:\Users\Administrador\Desktop\AMOSTRAS NO ORBITRAP LEANDRO RODAR NOVAMENTE\HIPOCAMPO\hipocampo_02.raw]
Data file hipocampo_02.temp.mgf

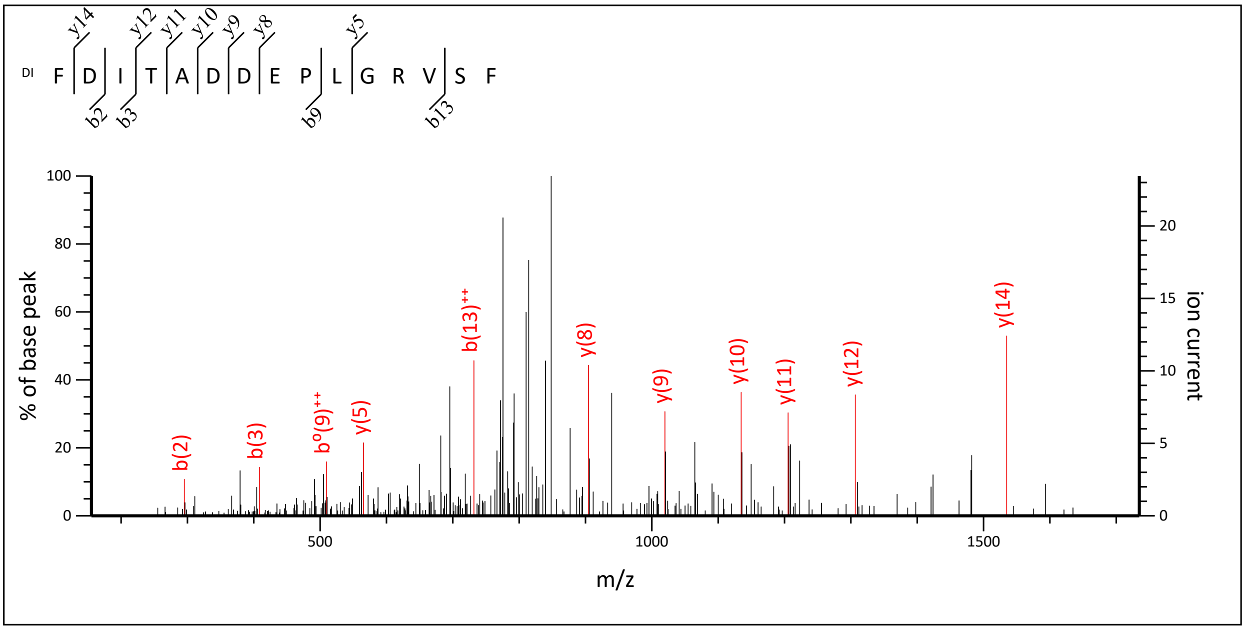

155.27 to 1734.77

Label all possible matches ○ Label matches used for scoring ◉

Monoisotopic mass of neutral peptide Mr(calc): 1712.8559
Variable modifications:
N-term : Dimethyl:2H(4) (N-term)
Ions Score: 49 **Expect:** 0.43
Matches : 12/136 fragment ions using 14 most intense peaks (help)

#	b	b^{++}	b^{*}	b^{*++}	b^{0}	b^{0++}	Seq.	y	y^{++}	y^{*}	y^{*++}	y^{0}	y^{0++}	#
1	180.1321	90.5697					F							15
2	**295.1590**	148.0832			277.1485	139.0779	D	***1534.7384***	767.8728	1517.7118	759.3596	1516.7278	758.8675	14
3	**408.2431**	204.6252			390.2325	195.6199	I	1419.7114	710.3594	1402.6849	701.8461	1401.7009	701.3541	13
4	**509.2908**	255.1490			491.2802	246.1437	T	***1306.6274***	653.8173	1289.6008	645.3040	1288.6168	644.8120	12
5	580.3279	290.6676			562.3173	281.6623	A	***1205.5797***	603.2935	1188.5531	594.7802	1187.5691	594.2882	11
6	695.3548	348.1811			677.3443	339.1758	D	***1134.5426***	567.7749	1117.5160	559.2617	1116.5320	558.7696	10
7	810.3818	405.6945			792.3712	396.6892	D	***1019.5156***	510.2615	1002.4891	501.7482	1001.5051	501.2562	9
8	939.4244	470.2158			921.4138	461.2105	E	***904.4887***	452.7480	887.4621	444.2347	886.4781	443.7427	8
9	1036.4771	518.7422			1018.4666	509.7369	P	775.4461	388.2267	758.4196	379.7134	757.4355	379.2214	7
10	1149.5612	575.2842			1131.5506	566.2790	L	678.3933	339.7003	661.3668	331.1870	660.3828	330.6950	6
11	1206.5827	603.7950			1188.5721	594.7897	G	***565.3093***	283.1583	548.2827	274.6450	547.2987	274.1530	5
12	1362.6838	681.8455	1345.6572	673.3323	1344.6732	672.8402	R	508.2878	254.6475	491.2613	246.1343	490.2772	245.6423	4
13	1461.7522	731.3797	1444.7256	722.8665	1443.7416	722.3745	V	352.1867	176.5970			334.1761	167.5917	3
14	1548.7842	774.8957	1531.7577	766.3825	1530.7737	765.8905	S	253.1183	127.0628			235.1077	118.0575	2
15							F	166.0863	83.5468					1

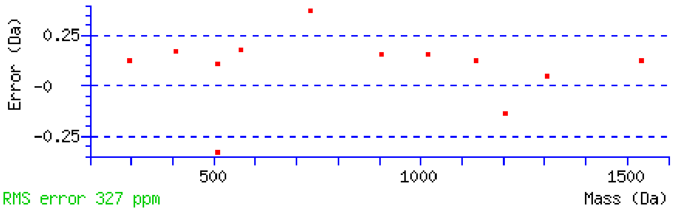

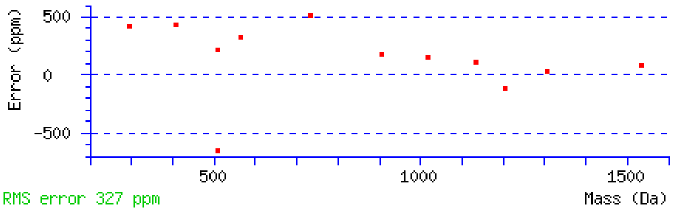

NCBI **BLAST** search of FDITADDEPLGRVSF
(Parameters: blastp, nr protein database, expect=20000, no filter, PAM30)
Other BLAST web gateways

All matches to this query

Score	Mr(calc)	Delta	Sequence
48.7	1712.8559	0.0121	FDITADDEPLGRVSF
25.4	1712.8052	0.0629	SLLWCAGVDSVTTNAC
22.4	1712.9083	-0.0403	TDLFEKGNADEFTI
21.1	1712.8791	-0.0111	MDITCYFLNKCGLL
20.6	1712.9473	-0.0793	SEDSSDDEPLIKKL
20.3	1712.8737	-0.0057	TELSWHSCRQLLY
19.8	1712.9382	-0.0701	PDSREFGCAKTLYI
17.8	1712.8255	0.0426	ASGGLSMAVEGPEGMER
17.6	1713.0400	-0.1719	FSHTAGVGLIVSQKTP
16.9	1712.8365	0.0316	SLFEAKGDVMATLEM

Mascot: http://www.matrixscience.com/

[redacted] Mascot Search Results

Peptide View

MS/MS Fragmentation of **VFDVELLKLE**
Found in **FKB1A_MOUSE** in **SwissProt**, Peptidyl-prolyl cis-trans isomerase FKBP1A OS=Mus musculus GN=Fkbp1a PE=1 SV=2

Match to Query 3517: 1259.744208 from(630.879380,2+) intensity(13627.6700) scans(11298) rawscans(sn11298) rtinseconds(4220.8133) index(8373)
Title: 8374: Scan 11298 (rt=4220.81) [C:\Users\Administrador\Desktop\AMOSTRAS NO ORBITRAP LEANDRO RODAR NOVAMENTE\HIPOCAMPO\hipocampo_01.raw]
Data file hipocampo_01.temp.mgf

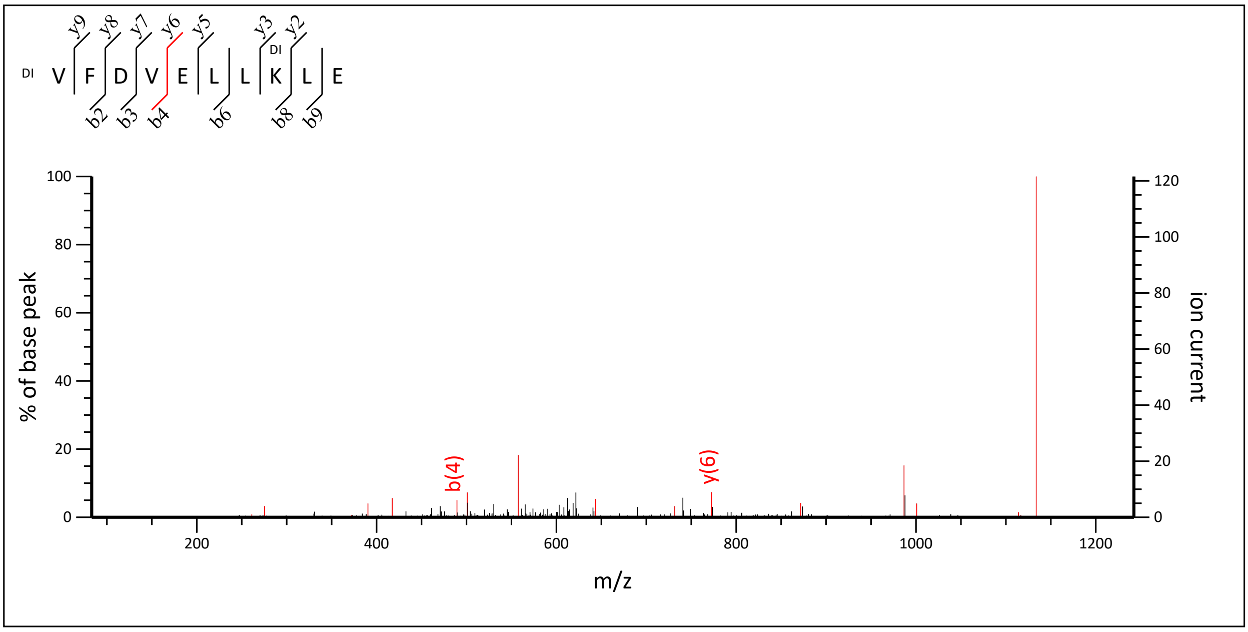

83.11 to 1242.39

Label all possible matches Label matches used for scoring

Monoisotopic mass of neutral peptide Mr(calc): 1259.7377
Variable modifications:
N-term : Dimethyl (N-term)
K8 : Dimethyl (K)
Ions Score: 53 **Expect:** 0.16
Matches : 17/86 fragment ions using 30 most intense peaks (help)

#	b	b^{++}	b*	b^{*++}	b^0	b^{0++}	Seq.	y	y^{++}	y*	y^{*++}	y^0	y^{0++}	#
1	128.1070	64.5571					V							10
2	275.1754	138.0913					F	1133.6453	567.3263	1116.6187	558.8130	1115.6347	558.3210	9
3	390.2023	195.6048			372.1918	186.5995	D	986.5768	493.7921	969.5503	485.2788	968.5663	484.7868	8
4	489.2708	245.1390			471.2602	236.1337	V	871.5499	436.2786	854.5233	427.7653	853.5393	427.2733	7
5	618.3134	309.6603			600.3028	300.6550	E	772.4815	386.7444	755.4549	378.2311	754.4709	377.7391	6
6	731.3974	366.2023			713.3869	357.1971	L	643.4389	322.2231	626.4123	313.7098	625.4283	313.2178	5
7	844.4815	422.7444			826.4709	413.7391	L	530.3548	265.6811	513.3283	257.1678	512.3443	256.6758	4
8	1000.6077	500.8075	983.5812	492.2942	982.5972	491.8022	K	417.2708	209.1390	400.2442	200.6257	399.2602	200.1337	3
9	1113.6918	557.3495	1096.6653	548.8363	1095.6812	548.3443	L	261.1445	131.0759			243.1339	122.0706	2
10							E	148.0604	74.5339			130.0499	65.5286	1

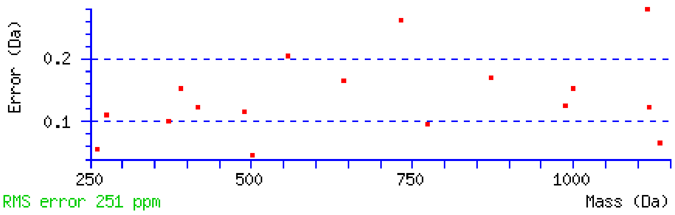

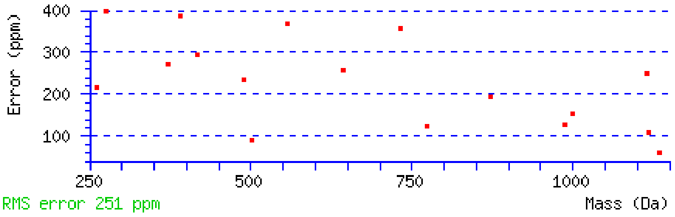

NCBI **BLAST** search of VFDVELLKLE
(Parameters: blastp, nr protein database, expect=20000, no filter, PAM30)
Other BLAST web gateways

All matches to this query

Score	Mr(calc)	Delta	Sequence
52.5	1259.7377	0.0065	VFDVELLKLE
25.2	1259.6319	0.1123	MQTVIEEVLE
24.5	1259.6985	0.0457	LEPLGTPMLIE
24.1	1259.6584	0.0858	VFPVQMSPIAQ
23.8	1259.5769	0.1673	GEDDATLEVIE
21.9	1259.6659	0.0783	ADRENIQVIE
21.7	1259.6790	0.0652	LLPEEAAEPQQ
21.7	1259.5704	0.1739	VMDVNDNAPEL
20.9	1259.6961	0.0481	IEKAHNNELE
20.1	1259.6192	0.1250	SFTCLRMELE

Mascot: http://www.matrixscience.com/

[redacted] Mascot Search Results

Peptide View

MS/MS Fragmentation of **AGVTVDELGKVLTPTQV**
Found in **PEBP1_MOUSE** in **SwissProt**, Phosphatidylethanolamine-binding protein 1 OS=Mus musculus GN=Pebp1 PE=1 SV=3

Match to Query 6578: 1790.066948 from(896.040750,2+) intensity(33433.7420) scans(10412) rawscans(sn10412) rtinseconds(3896.0992) index(7621)
Title: 7622: Scan 10412 (rt=3896.1) [C:\Users\Administrador\Desktop\AMOSTRAS NO ORBITRAP LEANDRO RODAR NOVAMENTE\HIPOCAMPO\hipocampo_01.raw]
Data file hipocampo_01.temp.mgf

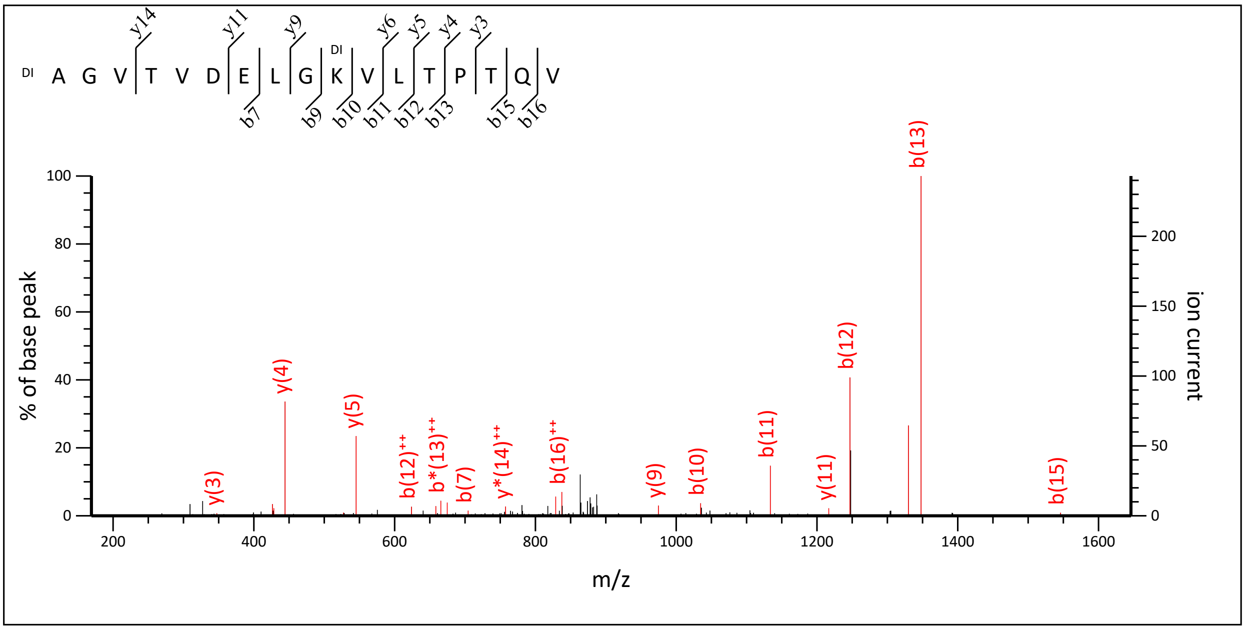

169.23 to 1646.06

Label all possible matches ○ Label matches used for scoring ◉

```
Monoisotopic mass of neutral peptide Mr(calc): 1790.0641
Variable modifications:
N-term : Dimethyl:2H(4) (N-term)
K10    : Dimethyl:2H(4) (K)
Ions Score: 46  Expect: 0.72
Matches : 26/162 fragment ions using 36 most intense peaks   (help)
```

#	b	b^{++}	b*	b^{*++}	b^0	b^{0++}	Seq.	y	y^{++}	y*	y^{*++}	y^0	y^{0++}	#
1	104.1008	52.5540					A							17
2	161.1223	81.0648					G	1687.9778	844.4926	1670.9513	835.9793	1669.9673	835.4873	16
3	260.1907	130.5990					V	1630.9564	815.9818	1613.9298	807.4686	1612.9458	806.9765	15
4	361.2384	181.1228			343.2278	172.1175	T	1531.8880	766.4476	1514.8614	757.9343	1513.8774	757.4423	14
5	460.3068	230.6570			442.2962	221.6517	V	1430.8403	715.9238	1413.8137	707.4105	1412.8297	706.9185	13
6	575.3337	288.1705			557.3231	279.1652	D	1331.7719	666.3896	1314.7453	657.8763	1313.7613	657.3843	12
7	***704.3763***	352.6918			686.3657	343.6865	E	***1216.7449***	608.8761	1199.7184	600.3628	1198.7344	599.8708	11
8	817.4604	409.2338			799.4498	400.2285	L	1087.7023	544.3548	1070.6758	535.8415	1069.6918	535.3495	10
9	874.4818	437.7446			856.4713	428.7393	G	***974.6183***	487.8128	957.5917	479.2995	956.6077	478.8075	9
10	***1034.6332***	517.8202	1017.6067	509.3070	1016.6226	508.8150	K	917.5968	459.3020	900.5703	450.7888	899.5862	450.2968	8
11	***1133.7016***	567.3544	1116.6751	558.8412	1115.6911	558.3492	V	***757.4454***	379.2264	740.4189	370.7131	739.4349	370.2211	7
12	***1246.7857***	**623.8965**	1229.7591	615.3832	1228.7751	614.8912	L	***658.3770***	329.6921	641.3505	321.1789	640.3665	320.6869	6
13	***1347.8334***	**674.4203**	1330.8068	665.9070	1329.8228	665.4150	T	***545.2930***	273.1501	528.2664	264.6368	527.2824	264.1448	5
14	1444.8861	722.9467	1427.8596	714.4334	1426.8756	713.9414	P	***444.2453***	222.6263	427.2187	214.1130	426.2347	213.6210	4
15	***1545.9338***	773.4705	1528.9073	764.9573	1527.9232	764.4653	T	***347.1925***	174.0999	330.1660	165.5866	329.1819	165.0946	3
16	1673.9924	**837.4998**	1656.9658	828.9866	1655.9818	828.4945	Q	246.1448	123.5761	229.1183	115.0628			2
17							V	118.0863	59.5468					1

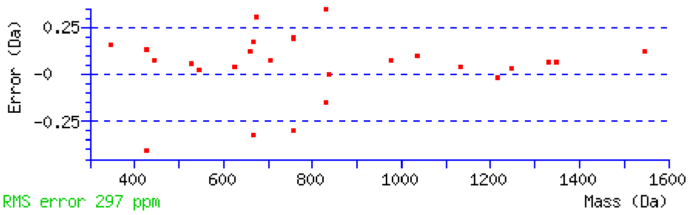

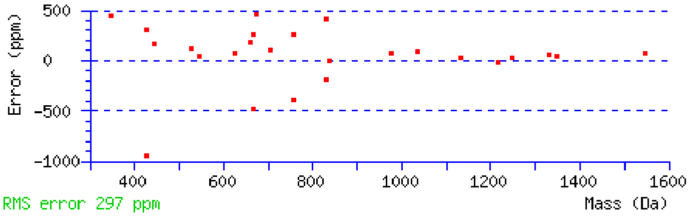

NCBI **BLAST** search of AGVTVDELGKVLTPTQV
(Parameters: blastp, nr protein database, expect=20000, no filter, PAM30)
Other BLAST web gateways

All matches to this query

Score	Mr(calc)	Delta	Sequence
46.4	1790.0641	0.0028	AGVTVDELGKVLTPTQV
31.2	1789.8743	0.1927	QLLSMGVFSRVTMYD
24.4	1789.9798	0.0872	ALANTLVALQMGVSVVD
24.3	1789.8954	0.1716	NGSLPCMESAVLTLAQI
22.7	1789.9911	0.0759	SNQHGIRRNLTAALGL
20.6	1789.7968	0.2701	EPETMETKSVTDYF
20.6	1790.0454	0.0215	LPYTFGGGTKLNIKR
20.6	1789.9032	0.1637	MPETITSRDAARFPI
20.6	1789.9761	0.0909	KCNTLFVVDVQTGRI
20.4	1790.0275	0.0395	REGLSRQHVLTRIGV

**Mascot:** http://www.matrixscience.com/

[redacted] Mascot Search Results

Peptide View

MS/MS Fragmentation of **DDYVPKLYEQLSGK**
Found in **PEBP1_MOUSE** in **SwissProt**, Phosphatidylethanolamine-binding protein 1 OS=Mus musculus GN=Pebp1 PE=1 SV=3

Match to Query 6409: 1750.006752 from(584.342860,3+) intensity(34890.2810) scans(8909) rawscans(sn8909) rtinseconds(3350.4485) index(6358)
Title: 6359: Scan 8909 (rt=3350.45) [C:\Users\Administrador\Desktop\AMOSTRAS NO ORBITRAP LEANDRO RODAR NOVAMENTE\HIPOCAMPO\hipocampo_01.raw]
Data file hipocampo_01.temp.mgf

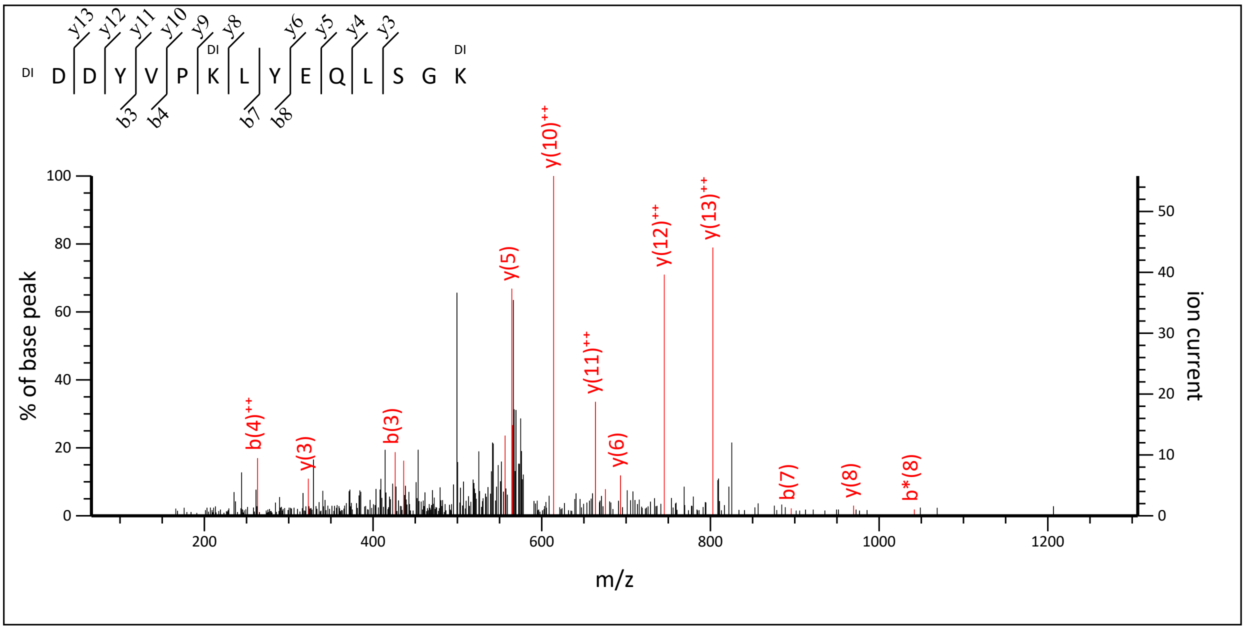

66.09 to 1306.64

Label all possible matches Label matches used for scoring

Monoisotopic mass of neutral peptide Mr(calc): 1749.9942
Variable modifications:
N-term : Dimethyl:2H(4) (N-term)
K6 : Dimethyl:2H(4) (K)
K14 : Dimethyl:2H(4) (K)
Ions Score: 28 **Expect:** 45
Matches : 18/142 fragment ions using 38 most intense peaks (help)

#	b	b++	b*	b*++	b0	b0++	Seq.	y	y++	y*	y*++	y0	y0++	#
1	148.0906	74.5490			130.0801	65.5437	D							14
2	**263.1176**	132.0624			245.1070	123.0571	D	1603.9182	***802.4627***	1586.8916	793.9494	1585.9076	793.4574	13
3	**426.1809**	213.5941			408.1703	204.5888	Y	1488.8912	***744.9492***	1471.8647	736.4360	1470.8806	735.9440	12
4	525.2493	263.1283			507.2387	254.1230	V	1325.8279	***663.4176***	1308.8013	654.9043	1307.8173	654.4123	11
5	622.3021	311.6547			604.2915	302.6494	P	1226.7595	***613.8834***	1209.7329	605.3701	1208.7489	604.8781	10
6	782.4534	391.7304	765.4269	383.2171	764.4429	382.7251	K	1129.7067	***565.3570***	1112.6802	556.8437	1111.6961	556.3517	9
7	**895.5375**	448.2724	878.5110	439.7591	877.5269	439.2671	L	***969.5553***	485.2813	952.5288	476.7680	951.5448	476.2760	8
8	1058.6008	529.8041	1041.5743	521.2908	1040.5903	520.7988	Y	856.4713	428.7393	839.4447	420.2260	838.4607	419.7340	7
9	1187.6434	594.3254	1170.6169	585.8121	1169.6329	585.3201	E	***693.4079***	347.2076	676.3814	338.6943	675.3974	338.2023	6
10	1315.7020	658.3546	1298.6755	649.8414	1297.6914	649.3494	Q	***564.3653***	282.6863	547.3388	274.1730	546.3548	273.6810	5
11	1428.7861	714.8967	1411.7595	706.3834	1410.7755	705.8914	L	***436.3068***	218.6570	419.2802	210.1437	418.2962	209.6517	4
12	1515.8181	758.4127	1498.7916	749.8994	1497.8075	749.4074	S	***323.2227***	162.1150	306.1962	153.6017	305.2121	153.1097	3
13	1572.8396	786.9234	1555.8130	778.4101	1554.8290	777.9181	G	236.1907	118.5990	219.1641	110.0857			2
14							K	179.1692	90.0882	162.1427	81.5750			1

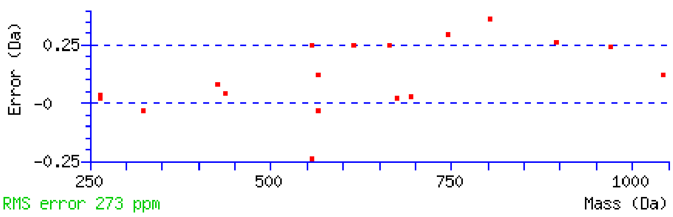

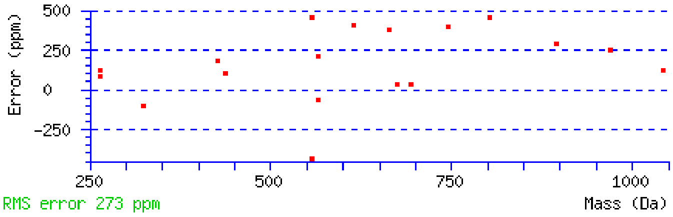

NCBI **BLAST** search of DDYVPKLYEQLSGK
(Parameters: blastp, nr protein database, expect=20000, no filter, PAM30)
Other BLAST web gateways

All matches to this query

Score	Mr(calc)	Delta	Sequence
28.5	1749.9942	0.0125	DDYVPKLYEQLSGK
22.5	1749.8537	0.1531	MLAKSAMPIEVMMNE
17.6	1749.9440	0.0627	VVLSYPELKVVYED
15.1	1749.9988	0.0079	PRKAVPGNSFTYVNE
14.9	1749.7377	0.2691	HPSSSVSSSPSQMDHH
14.4	1749.9245	0.0823	KSTTVQVPMMHQLE
14.1	1749.7433	0.2635	DDGAVFLAACSSGDTDE
13.3	1750.0866	-0.0799	FLKAGTVCRKEKNE
13.2	1749.6323	0.3745	MSGMMMSHMSQASMQ
13.2	1750.0792	-0.0725	AAHLGKKARGKEADE

Mascot: http://www.matrixscience.com/

[redacted] Mascot Search Results

Peptide View

MS/MS Fragmentation of **DGLDPGKLYTL**
Found in **PEBP1_MOUSE** in **SwissProt**, Phosphatidylethanolamine-binding protein 1 OS=Mus musculus GN=Pebp1 PE=1 SV=3

Match to Query 3369: 1246.687088 from(624.350820,2+) intensity(21610.6190) scans(9057) rawscans(sn9057) rtinseconds(3452.6934) index(6450)
Title: 6451: Scan 9057 (rt=3452.69) [C:\Users\Administrador\Desktop\AMOSTRAS NO ORBITRAP LEANDRO RODAR NOVAMENTE\HIPOCAMPO\hipocampo_02.raw]
Data file hipocampo_02.temp.mgf

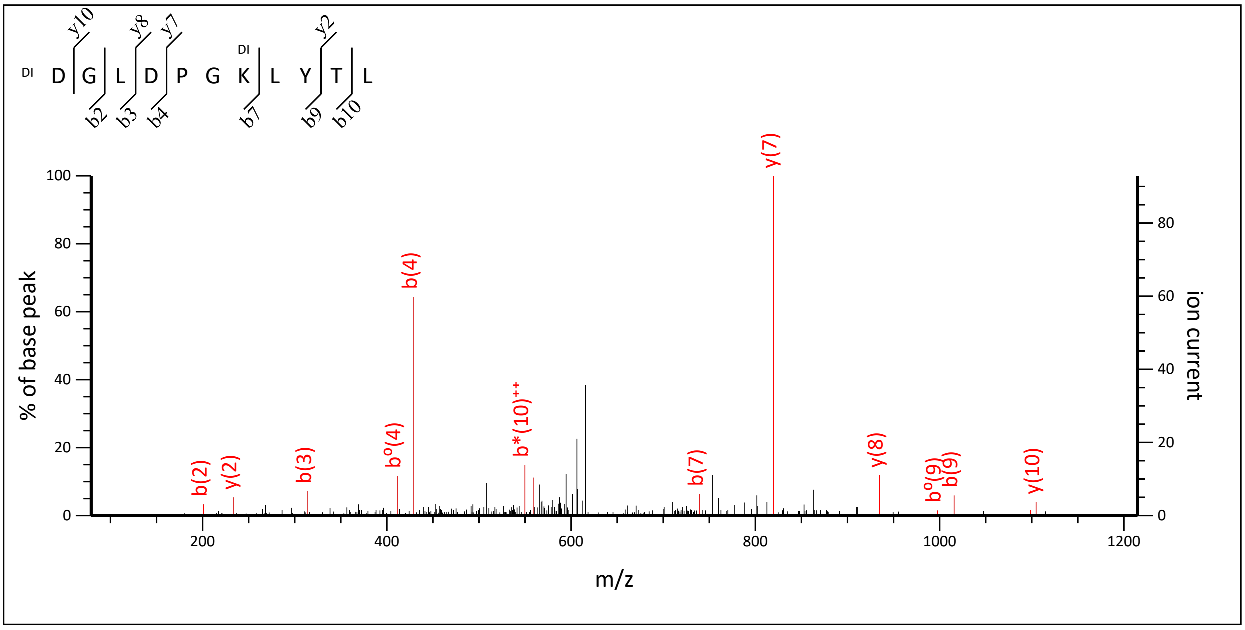

79.08 to 1214.8

Label all possible matches ○ Label matches used for scoring ◉

```
Monoisotopic mass of neutral peptide Mr(calc): 1246.6809
Variable modifications:
N-term : Dimethyl (N-term)
K7     : Dimethyl (K)
Ions Score: 34  Expect: 11
Matches : 15/98 fragment ions using 20 most intense peaks  (help)
```

#	b	b^{++}	b*	b^{*++}	b^0	b^{0++}	Seq.	y	y^{++}	y*	y^{*++}	y^0	y^{0++}	#
1	144.0655	72.5364			126.0550	63.5311	D							11
2	***201.0870***	101.0471			183.0764	92.0418	G	***1104.6299***	552.8186	1087.6034	544.3053	1086.6194	543.8133	10
3	***314.1710***	157.5892			296.1605	148.5839	L	1047.6085	524.3079	1030.5819	515.7946	1029.5979	515.3026	9
4	***429.1980***	215.1026			411.1874	206.0974	D	***934.5244***	467.7658	917.4979	459.2526	916.5138	458.7606	8
5	526.2508	263.6290			508.2402	254.6237	P	***819.4975***	410.2524	802.4709	401.7391	801.4869	401.2471	7
6	583.2722	292.1397			565.2617	283.1345	G	722.4447	361.7260	705.4182	353.2127	704.4341	352.7207	6
7	***739.3985***	370.2029	722.3719	361.6896	721.3879	361.1976	K	665.4232	333.2153	648.3967	324.7020	647.4127	324.2100	5
8	852.4825	426.7449	835.4560	418.2316	834.4720	417.7396	L	509.2970	255.1521			491.2864	246.1468	4
9	***1015.5459***	508.2766	998.5193	499.7633	997.5353	499.2713	Y	396.2129	198.6101			378.2023	189.6048	3
10	1116.5936	558.8004	1099.5670	550.2871	1098.5830	549.7951	T	***233.1496***	117.0784			215.1390	108.0731	2
11							L	132.1019	66.5546					1

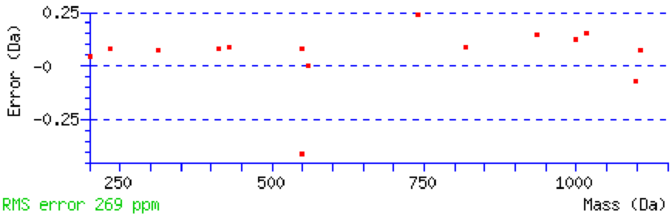

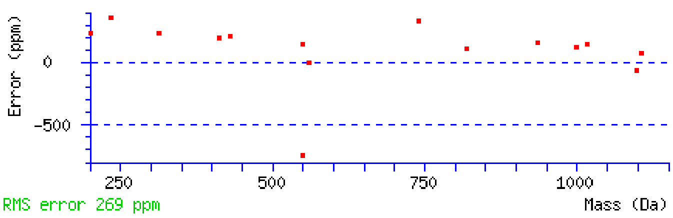

NCBI **BLAST** search of DGLDPGKLYTL
(Parameters: blastp, nr protein database, expect=20000, no filter, PAM30)
Other BLAST web gateways

All matches to this query

Score	Mr(calc)	Delta	Sequence
34.4	1246.6809	0.0062	DGLDPGKLYTL
28.9	1246.7529	-0.0658	AKDLRGEMTL
28.8	1246.6670	0.0201	RSDRPAYVTI
27.1	1246.7716	-0.0845	VPVMAIRDKM
25.1	1246.7781	-0.0910	KIDPSMGTLTI
23.7	1246.7166	-0.0295	AKDMGGQLGGLA
21.8	1246.6769	0.0102	ATINTIGTGASTI
21.5	1246.6115	0.0756	GDIPVSGMLLSD
19.5	1246.6838	0.0033	PVDVADVPEGTL
19.0	1246.5717	0.1153	DQDFPSSAPTL

Mascot: http://www.matrixscience.com/

[redacted] Mascot Search Results

Peptide View

MS/MS Fragmentation of **GLDPGKLYTL**
Found in **PEBP1_MOUSE** in **SwissProt**, Phosphatidylethanolamine-binding protein 1 OS=Mus musculus GN=Pebp1 PE=1 SV=3

Match to Query 2711: 1131.658828 from(566.836690,2+) intensity(49904.5080) scans(8571) rawscans(sn8571) rtinseconds(3230.7918) index(6097)
Title: 6098: Scan 8571 (rt=3230.79) [C:\Users\Administrador\Desktop\AMOSTRAS NO ORBITRAP LEANDRO RODAR NOVAMENTE\HIPOCAMPO\hipocampo_01.raw]
Data file hipocampo_01.temp.mgf

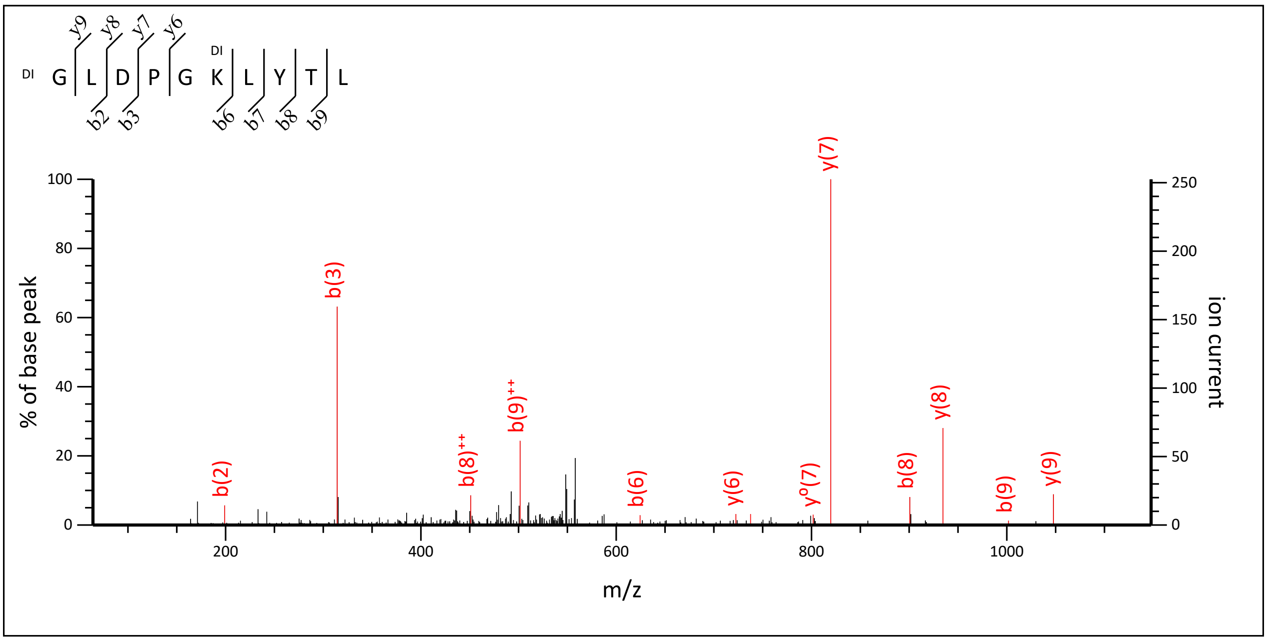

64.16 to 1147.76

Label all possible matches ○ Label matches used for scoring ◉

Monoisotopic mass of neutral peptide Mr(calc): 1131.6540
Variable modifications:
N-term : Dimethyl (N-term)
K6 : Dimethyl (K)
Ions Score: 52 **Expect:** 0.18
Matches : 14/84 fragment ions using 18 most intense peaks (help)

#	b	b^{++}	b*	b^{*++}	b^0	b^{0++}	Seq.	y	y^{++}	y*	y^{*++}	y^0	y^{0++}	#
1	86.0600	43.5337					G							10
2	***199.1441***	100.0757					L	***1047.6085***	524.3079	1030.5819	515.7946	1029.5979	515.3026	9
3	***314.1710***	157.5892			296.1605	148.5839	D	***934.5244***	467.7658	917.4979	459.2526	916.5138	458.7606	8
4	411.2238	206.1155			393.2132	197.1103	P	***819.4975***	410.2524	802.4709	401.7391	801.4869	401.2471	7
5	468.2453	234.6263			450.2347	225.6210	G	***722.4447***	361.7260	705.4182	353.2127	704.4341	352.7207	6
6	***624.3715***	312.6894	607.3450	304.1761	606.3610	303.6841	K	665.4232	333.2153	648.3967	324.7020	647.4127	324.2100	5
7	***737.4556***	369.2314	720.4291	360.7182	719.4450	360.2262	L	509.2970	255.1521			491.2864	246.1468	4
8	***900.5189***	**450.7631**	883.4924	442.2498	882.5084	441.7578	Y	396.2129	198.6101			378.2023	189.6048	3
9	***1001.5666***	**501.2869**	984.5401	492.7737	983.5560	492.2817	T	233.1496	117.0784			215.1390	108.0731	2
10							L	132.1019	66.5546					1

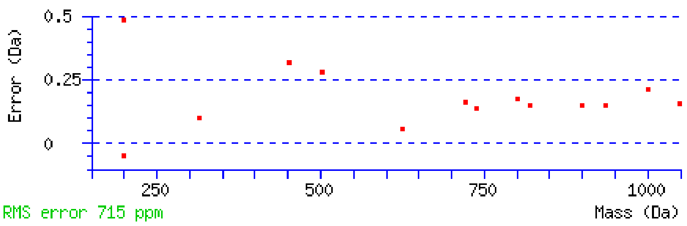

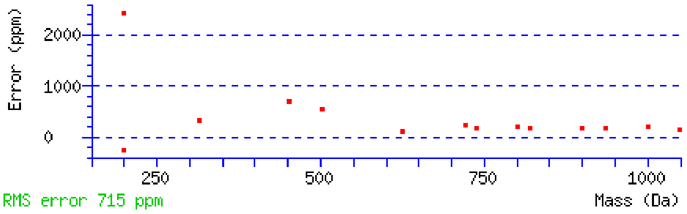

NCBI **BLAST** search of GLDPGKLYTL
(Parameters: blastp, nr protein database, expect=20000, no filter, PAM30)
Other BLAST web gateways

All matches to this query

Score	Mr(calc)	Delta	Sequence
52.2	1131.6540	0.0049	GLDPGKLYTL
33.0	1131.4794	0.1794	QDYMFETL
29.4	1131.5448	0.1140	QDFPSSAPTL
24.9	1131.5772	0.0816	ERVDGDVTTI
24.9	1131.6135	0.0453	GLDRSLETLT
24.4	1131.5875	0.0714	SMVSSGSSVTL
23.6	1131.6023	0.0565	EKVQEETTL
23.3	1131.5295	0.1293	QDSEGDVLSL
21.9	1131.7355	-0.0766	ALPSPIEKTI
21.8	1131.5560	0.1028	GIPDDRDGLF

Mascot: http://www.matrixscience.com/

[redacted] Mascot Search Results

Peptide View

MS/MS Fragmentation of **GVTVDELGKVLTPTQV**
Found in **PEBP1_MOUSE** in **SwissProt**, Phosphatidylethanolamine-binding protein 1 OS=Mus musculus GN=Pebp1 PE=1 SV=3

Match to Query 6673: 1710.997988 from(856.506270,2+) intensity(52410.6160) scans(10133-10239) rawscans(sn10133:sn10239) rtinseconds(3813.7829-3851.1647) index(7485)
Title: 7486: Sum of 2 scans in range 10133 (rt=3813.78) to 10239 (rt=3851.16) [C:\Users\Administrador\Desktop\AMOSTRAS NO ORBITRAP LEANDRO RODAR NOVAMENTE\CORTEX\cortex_01.raw]
Data file cortex_01.temp.mgf

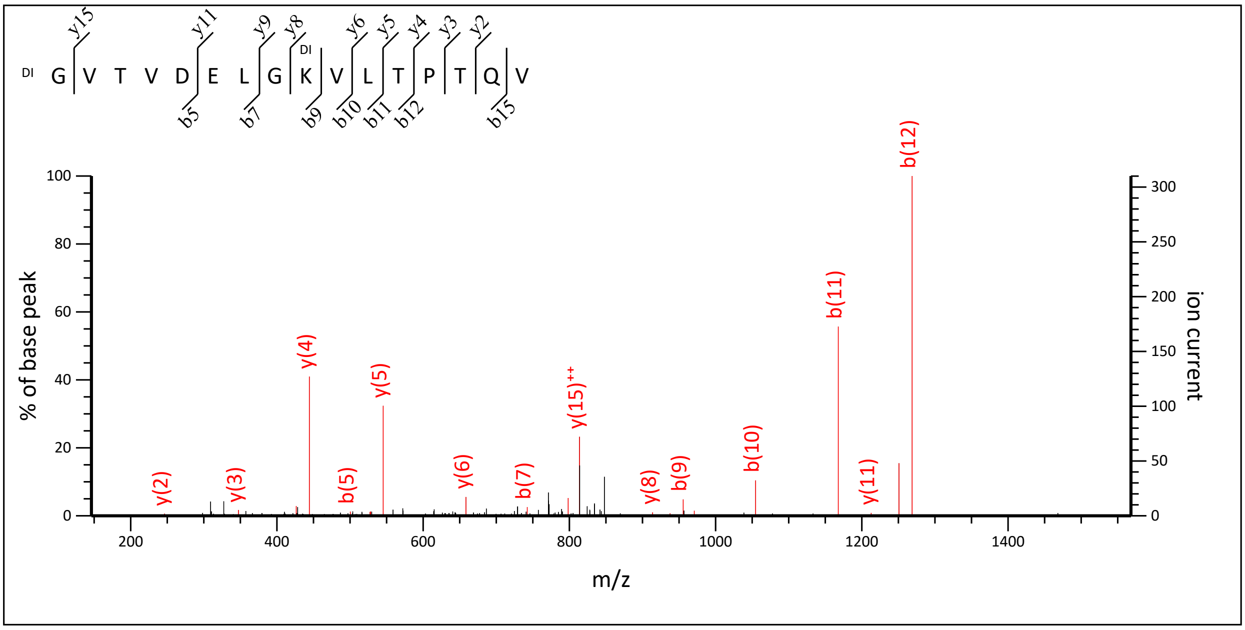

146.29 to 1568.09

Label all possible matches ○ Label matches used for scoring ◉

```
Monoisotopic mass of neutral peptide Mr(calc): 1710.9768
Variable modifications:
N-term : Dimethyl (N-term)
K9     : Dimethyl (K)
Ions Score: 34  Expect: 13
Matches : 21/154 fragment ions using 46 most intense peaks   (help)
```

#	b	b++	b*	b*++	b0	b0++	Seq.	y	y++	y*	y*++	y0	y0++	#
1	86.0600	43.5337					G							16
2	185.1285	93.0679					V	1626.9313	813.9693	1609.9047	805.4560	1608.9207	804.9640	15
3	286.1761	143.5917			268.1656	134.5864	T	1527.8629	764.4351	1510.8363	755.9218	1509.8523	755.4298	14
4	385.2445	193.1259			367.2340	184.1206	V	1426.8152	713.9112	1409.7886	705.3980	1408.8046	704.9059	13
5	**500.2715**	250.6394			482.2609	241.6341	D	1327.7468	664.3770	1310.7202	655.8637	1309.7362	655.3717	12
6	629.3141	315.1607			611.3035	306.1554	E	***1212.7198***	606.8635	1195.6933	598.3503	1194.7093	597.8583	11
7	**742.3981**	371.7027			724.3876	362.6974	L	1083.6772	542.3423	1066.6507	533.8290	1065.6667	533.3370	10
8	799.4196	400.2134			781.4090	391.2082	G	***970.5932***	485.8002	953.5666	477.2869	952.5826	476.7949	9
9	**955.5459**	478.2766	938.5193	469.7633	937.5353	469.2713	K	***913.5717***	457.2895	896.5451	448.7762	895.5611	448.2842	8
10	**1054.6143**	527.8108	1037.5877	519.2975	1036.6037	518.8055	V	757.4454	379.2264	740.4189	370.7131	739.4349	370.2211	7
11	**1167.6984**	584.3528	1150.6718	575.8395	1149.6878	575.3475	L	***658.3770***	329.6921	641.3505	321.1789	640.3665	320.6869	6
12	**1268.7460**	634.8767	1251.7195	626.3634	1250.7355	625.8714	T	***545.2930***	273.1501	528.2664	264.6368	527.2824	264.1448	5
13	1365.7988	683.4030	1348.7722	674.8898	1347.7882	674.3978	P	***444.2453***	222.6263	427.2187	214.1130	426.2347	213.6210	4
14	1466.8465	733.9269	1449.8199	725.4136	1448.8359	724.9216	T	***347.1925***	174.0999	330.1660	165.5866	329.1819	165.0946	3
15	1594.9051	797.9562	1577.8785	789.4429	1576.8945	788.9509	Q	***246.1448***	123.5761	229.1183	115.0628			2
16							V	118.0863	59.5468					1

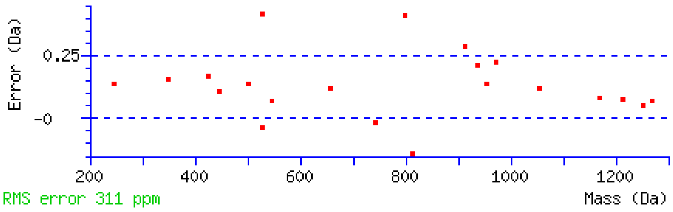

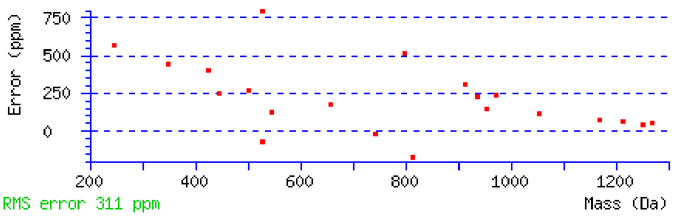

NCBI **BLAST** search of GVTVDELGKVLTPTQV
(Parameters: blastp, nr protein database, expect=20000, no filter, PAM30)
Other BLAST web gateways

All matches to this query

Score	Mr(calc)	Delta	Sequence
33.9	1710.9768	0.0212	GVTVDELGKVLTPTQV
29.5	1710.8788	0.1192	SAQSSPQQAVLTIPSQ
23.7	1710.9264	0.0716	QLSSTLKRYTESSR
22.0	1710.8611	0.1369	LLGCENELGPITPGRD
22.0	1711.0402	-0.0422	MRYHKAHLTRRL
21.8	1710.7957	0.2023	QLMQYDQCLTKGPD
21.8	1711.0433	-0.0454	QLGLVDSLVPLGILGSV
21.1	1710.9930	0.0050	GLGLATLVSETNRVLH
21.1	1710.8976	0.1003	ILMTLVLFFPDMLG
21.1	1711.0535	-0.0556	LIFTIVVDILGNLLV

Mascot: http://www.matrixscience.com/

Mascot Search Results

Peptide View

MS/MS Fragmentation of **KGNDISSGTVL**
Found in **PEBP1_MOUSE** in **SwissProt**, Phosphatidylethanolamine-binding protein 1 OS=Mus musculus GN=Pebp1 PE=1 SV=3

Match to Query 4090: 1145.645108 from(573.829830,2+) intensity(329543.7200) scans(6352) rawscans(sn6352) rtinseconds(2247.2864) index(2985)
Title: 2986: Scan 6352 (rt=2247.29) [C:\Users\Administrador\Desktop\AMOSTRAS NO ORBITRAP LEANDRO RODAR NOVAMENTE\HIPOCAMPO\hipocampo_04.raw]
Data file hipocampo_04.temp.mgf

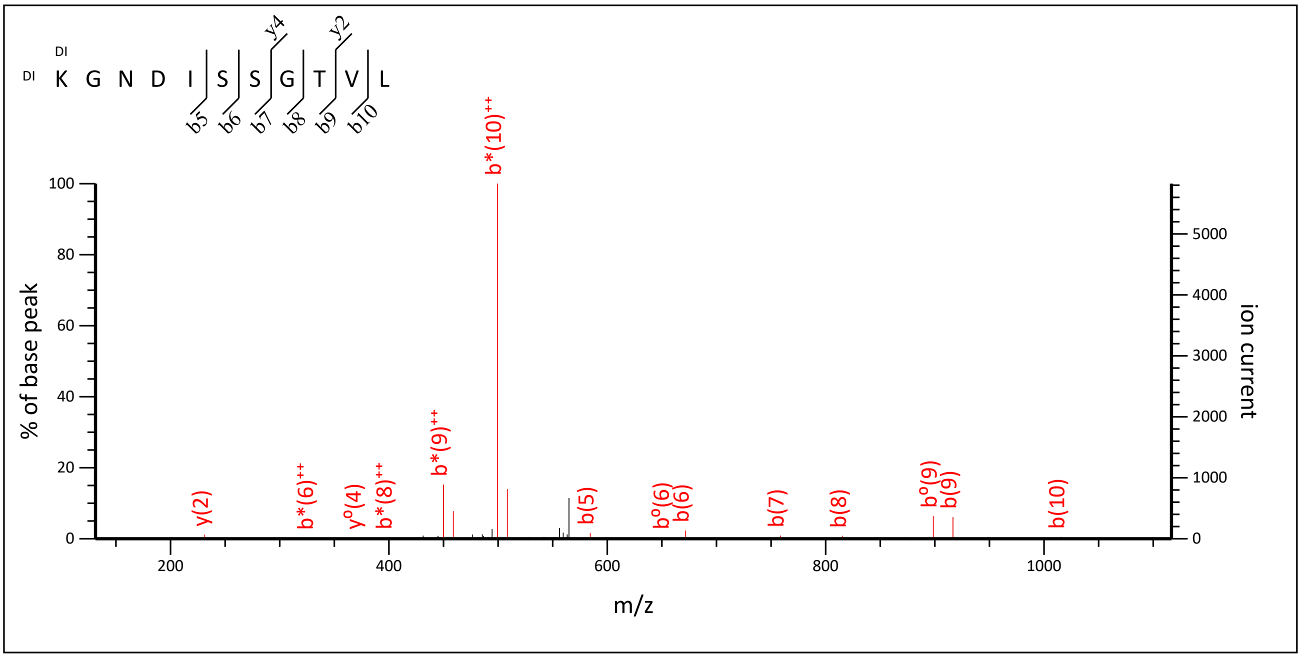

131.29 to 1116.72

Label all possible matches Label matches used for scoring

Monoisotopic mass of neutral peptide Mr(calc): 1145.6292
Variable modifications:
N-term : Dimethyl (N-term)
K1 : Dimethyl (K)
Ions Score: 38 **Expect:** 4.4
Matches : 22/94 fragment ions using 30 most intense peaks (help)

#	b	b^{++}	b*	b^{*++}	b^0	b^{0++}	Seq.	y	y^{++}	y*	y^{*++}	y^0	y^{0++}	#
1	185.1648	93.0861	168.1383	84.5728			K							11
2	242.1863	121.5968	225.1598	113.0835			G	962.4789	481.7431	945.4524	473.2298	944.4684	472.7378	10
3	356.2292	178.6183	339.2027	170.1050			N	905.4575	453.2324	888.4309	444.7191	887.4469	444.2271	9
4	471.2562	236.1317	454.2296	227.6185	453.2456	227.1264	D	791.4145	396.2109			773.4040	387.2056	8
5	***584.3402***	292.6738	567.3137	284.1605	566.3297	283.6685	I	676.3876	338.6974			658.3770	329.6921	7
6	***671.3723***	336.1898	654.3457	327.6765	653.3617	327.1845	S	563.3035	282.1554			545.2930	273.1501	6
7	***758.4043***	379.7058	741.3777	371.1925	740.3937	370.7005	S	476.2715	238.6394			458.2609	229.6341	5
8	***815.4258***	**408.2165**	798.3992	399.7032	797.4152	399.2112	G	389.2395	195.1234			371.2289	186.1181	4
9	***916.4734***	**458.7404**	899.4469	450.2271	898.4629	449.7351	T	332.2180	166.6126			314.2074	157.6074	3
10	***1015.5419***	**508.2746**	998.5153	499.7613	997.5313	499.2693	V	231.1703	116.0888					2
11							L	132.1019	66.5546					1

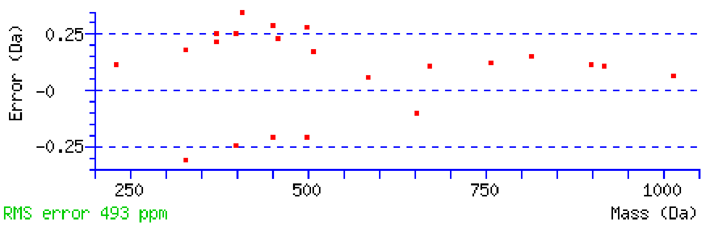

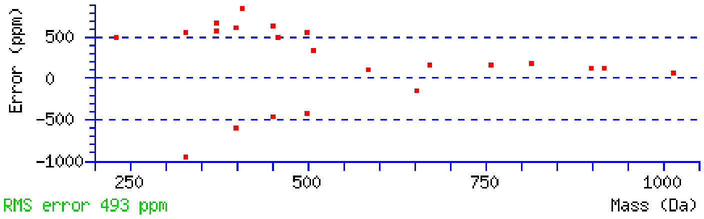

NCBI **BLAST** search of KGNDISSGTVL
(Parameters: blastp, nr protein database, expect=20000, no filter, PAM30)
Other BLAST web gateways

All matches to this query

Score	Mr(calc)	Delta	Sequence
49.1	1145.7383	-0.0932	YIHGKSGTVL
38.3	1145.6292	0.0159	KGNDISSGTVL
36.7	1145.5746	0.0705	GVPGSDGQPGTP
35.8	1145.6366	0.0085	DVLLCVSGTVI
35.7	1145.7048	-0.0597	RGFYKGTVL
33.6	1145.7008	-0.0557	HTQAKQGTVL
31.7	1145.6416	0.0035	VVSAAGGACPAVL
29.4	1145.7372	-0.0921	NPRGGKVTVL
28.1	1145.5564	0.0887	SGVAALGSSGTDP
27.9	1145.6747	-0.0295	VPFRPSSAVL

Mascot: http://www.matrixscience.com/

[redacted] Mascot Search Results

Peptide View

MS/MS Fragmentation of **KGNDISSGTVLSDYVGSGPPSGTGL**
Found in **PEBP1_MOUSE** in **SwissProt**, Phosphatidylethanolamine-binding protein 1 OS=Mus musculus GN=Pebp1 PE=1 SV=3

Match to Query 9731: 2420.222848 from(1211.118700,2+) intensity(5879.7676) scans(9689) rawscans(sn9689) rtinseconds(3685.0786) index(6733)
Title: 6734: Scan 9689 (rt=3685.08) [C:\Users\Administrador\Desktop\AMOSTRAS NO ORBITRAP LEANDRO RODAR NOVAMENTE\ESTRIADO\estriado_01.raw]
Data file estriado_01.temp.mgf

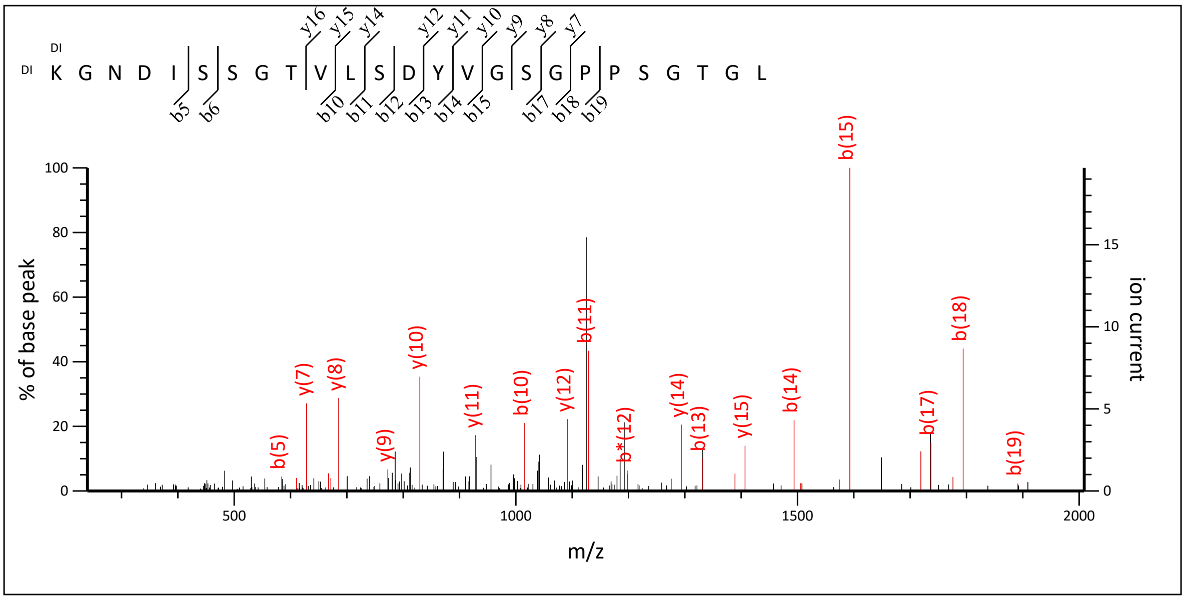

239.42 to 2009.02

Label all possible matches ○ Label matches used for scoring ◉

```
Monoisotopic mass of neutral peptide Mr(calc): 2420.2071
Variable modifications:
N-term : Dimethyl (N-term)
K1     : Dimethyl (K)
Ions Score: 43  Expect: 0.01
Matches : 27/234 fragment ions using 60 most intense peaks   (help)
```

#	b	b++	b*	b*++	b0	b0++	Seq.	y	y++	y*	y*++	y0	y0++	#
1	185.1648	93.0861	168.1383	84.5728			K							25
2	242.1863	121.5968	225.1598	113.0835			G	2237.0568	1119.0320	2220.0303	1110.5188	2219.0462	1110.0268	24
3	356.2292	178.6183	339.2027	170.1050			N	2180.0353	1090.5213	2163.0088	1082.0080	2162.0248	1081.5160	23
4	471.2562	236.1317	454.2296	227.6185	453.2456	227.1264	D	2065.9924	1033.4998			2047.9819	1024.4946	22
5	**584.3402**	292.6738	567.3137	284.1605	566.3297	283.6685	I	1950.9655	975.9864			1932.9549	966.9811	21
6	**671.3723**	336.1898	654.3457	327.6765	653.3617	327.1845	S	1837.8814	919.4443			1819.8709	910.4391	20
7	758.4043	379.7058	741.3777	371.1925	740.3937	370.7005	S	1750.8494	875.9283			1732.8388	866.9230	19
8	815.4258	408.2165	798.3992	399.7032	797.4152	399.2112	G	1663.8174	832.4123			1645.8068	823.4070	18
9	916.4734	458.7404	899.4469	450.2271	898.4629	449.7351	T	1606.7959	803.9016			1588.7853	794.8963	17
10	**1015.5419**	508.2746	998.5153	499.7613	997.5313	499.2693	V	**1505.7482**	753.3777			1487.7377	744.3725	16
11	**1128.6259**	564.8166	1111.5994	556.3033	1110.6153	555.8113	L	**1406.6798**	703.8435			1388.6692	694.8383	15
12	1215.6579	608.3326	1198.6314	599.8193	1197.6474	599.3273	S	**1293.5957**	647.3015			1275.5852	638.2962	14
13	**1330.6849**	665.8461	1313.6583	657.3328	1312.6743	656.8408	D	1206.5637	603.7855			1188.5531	594.7802	13
14	**1493.7482**	747.3777	1476.7217	738.8645	1475.7376	738.3725	Y	**1091.5368**	546.2720			1073.5262	537.2667	12
15	**1592.8166**	796.9120	1575.7901	788.3987	1574.8061	787.9067	V	**928.4734**	464.7404			910.4629	455.7351	11
16	1649.8381	825.4227	1632.8115	816.9094	1631.8275	816.4174	G	**829.4050**	415.2061			811.3945	406.2009	10
17	**1736.8701**	868.9387	1719.8436	860.4254	1718.8596	859.9334	S	**772.3836**	386.6954			754.3730	377.6901	9
18	**1793.8916**	897.4494	1776.8650	888.9362	1775.8810	888.4441	G	**685.3515**	343.1794			667.3410	334.1741	8
19	**1890.9443**	945.9758	1873.9178	937.4625	1872.9338	936.9705	P	**628.3301**	314.6687			610.3195	305.6634	7
20	1987.9971	994.5022	1970.9706	985.9889	1969.9865	985.4969	P	531.2773	266.1423			513.2667	257.1370	6
21	2075.0291	1038.0182	2058.0026	1029.5049	2057.0186	1029.0129	S	434.2245	217.6159			416.2140	208.6106	5
22	2132.0506	1066.5289	2115.0241	1058.0157	2114.0400	1057.5237	G	347.1925	174.0999			329.1819	165.0946	4

23	2233.0983	1117.0528	2216.0717	1108.5395	2215.0877	1108.0475	T	290.1710	145.5892			272.1605	136.5839	3
24	2290.1197	1145.5635	2273.0932	1137.0502	2272.1092	1136.5582	G	189.1234	95.0653					2
25							L	132.1019	66.5546					1

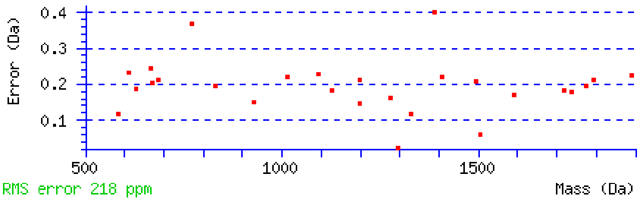

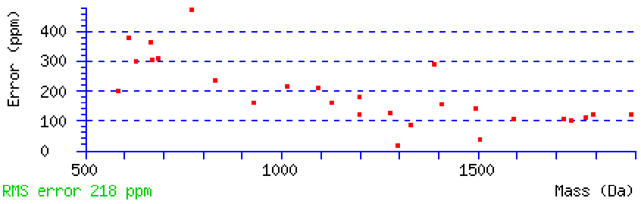

NCBI **BLAST** search of KGNDISSGTVLSDYVGSGPPSGTGL
(Parameters: blastp, nr protein database, expect=20000, no filter, PAM30)
Other BLAST web gateways

All matches to this query

Score	Mr(calc)	Delta	Sequence
43.3	2420.2071	0.0157	KGNDISSGTVLSDYVGSGPPSGTGL
21.4	2420.0914	0.1314	GTAMPPDDVTSLLHLGSGHDNDGA
20.5	2420.4215	-0.1987	SVFLLCPWVIVLSVTTESKVE
18.8	2420.3422	-0.1194	KSLVGASGGVYALMGGYFMNVIV
17.3	2420.0910	0.1319	CDTTHSNVSCLEMDVPIDLSK
16.8	2420.2760	-0.0532	LLSLSTSGVLSIWDIDIITAMS
16.2	2420.2226	0.0003	QGGLVHGGSVGGLAQTNFEREGGSA
16.0	2420.2329	-0.0101	RLANSTASVSRGSMQELDTLAGL
15.9	2420.1642	0.0587	KAMSSEESSNGSLSVEFRHLQ
15.7	2420.1244	0.0985	AFPCGSDHTPSPMVSCVPLEATP

Mascot: http://www.matrixscience.com/

[redacted] Mascot Search Results

Peptide View

MS/MS Fragmentation of **KGNDISSGTVLSDYVGSGPPSGTGLHRY**
Found in **PEBP1_MOUSE** in **SwissProt**, Phosphatidylethanolamine-binding protein 1 OS=Mus musculus GN=Pebp1 PE=1 SV=3

Match to Query 9997: 2876.458362 from(959.826730,3+) intensity(24883.7520) scans(7534) rawscans(sn7534) rtinseconds(2864.5721) index(5263)
Title: 5264: Scan 7534 (rt=2864.57) [C:\Users\Administrador\Desktop\AMOSTRAS NO ORBITRAP LEANDRO RODAR NOVAMENTE\HIPOCAMPO\hipocampo_01.raw]
Data file hipocampo_01.temp.mgf

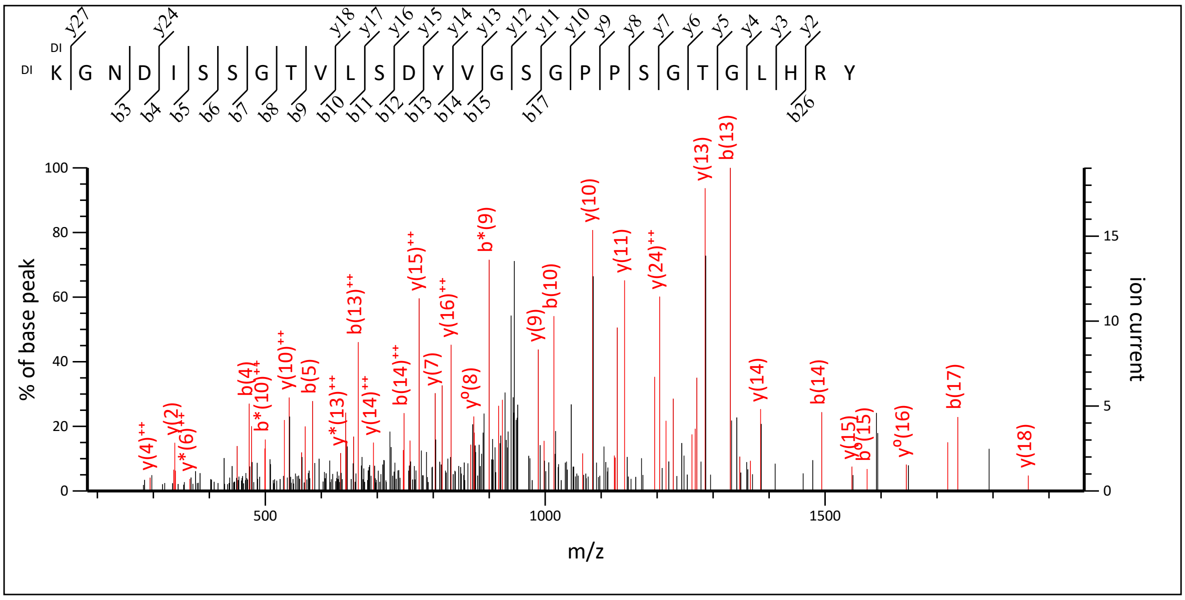

182.18 to 1962.96

Label all possible matches ○ Label matches used for scoring ◉

```
Monoisotopic mass of neutral peptide Mr(calc): 2876.4305
Variable modifications:
N-term : Dimethyl (N-term)
K1     : Dimethyl (K)
Ions Score: 81  Expect: 0.0003
Matches : 69/306 fragment ions using 105 most intense peaks   (help)
```

#	b	b^{++}	b*	b^{*++}	b^{0}	b^{0++}	Seq.	y	y^{++}	y*	y^{*++}	y^{0}	y^{0++}	#
1	185.1648	93.0861	168.1383	84.5728			K							28
2	242.1863	121.5968	225.1598	113.0835			G	2693.2802	1347.1437	2676.2536	1338.6304	2675.2696	1338.1384	27
3	356.2292	178.6183	339.2027	170.1050			N	2636.2587	1318.6330	2619.2322	1310.1197	2618.2481	1309.6277	26
4	471.2562	236.1317	454.2296	227.6185	453.2456	227.1264	D	2522.2158	1261.6115	2505.1892	1253.0983	2504.2052	1252.6062	25
5	584.3402	292.6738	567.3137	284.1605	566.3297	283.6685	I	2407.1888	1204.0981	2390.1623	1195.5848	2389.1783	1195.0928	24
6	671.3723	336.1898	654.3457	327.6765	653.3617	327.1845	S	2294.1048	1147.5560	2277.0782	1139.0427	2276.0942	1138.5507	23
7	758.4043	379.7058	741.3777	371.1925	740.3937	370.7005	S	2207.0727	1104.0400	2190.0462	1095.5267	2189.0622	1095.0347	22
8	815.4258	408.2165	798.3992	399.7032	797.4152	399.2112	G	2120.0407	1060.5240	2103.0142	1052.0107	2102.0301	1051.5187	21
9	916.4734	458.7404	899.4469	450.2271	898.4629	449.7351	T	2063.0192	1032.0133	2045.9927	1023.5000	2045.0087	1023.0080	20
10	1015.5419	508.2746	998.5153	499.7613	997.5313	499.2693	V	1961.9716	981.4894	1944.9450	972.9761	1943.9610	972.4841	19
11	1128.6259	564.8166	1111.5994	556.3033	1110.6153	555.8113	L	1862.9032	931.9552	1845.8766	923.4419	1844.8926	922.9499	18
12	1215.6579	608.3326	1198.6314	599.8193	1197.6474	599.3273	S	1749.8191	875.4132	1732.7925	866.8999	1731.8085	866.4079	17
13	1330.6849	665.8461	1313.6583	657.3328	1312.6743	656.8408	D	1662.7871	831.8972	1645.7605	823.3839	1644.7765	822.8919	16
14	1493.7482	747.3777	1476.7217	738.8645	1475.7376	738.3725	Y	1547.7601	774.3837	1530.7336	765.8704	1529.7496	765.3784	15
15	1592.8166	796.9120	1575.7901	788.3987	1574.8061	787.9067	V	1384.6968	692.8520	1367.6702	684.3388	1366.6862	683.8468	14
16	1649.8381	825.4227	1632.8115	816.9094	1631.8275	816.4174	G	1285.6284	643.3178	1268.6018	634.8046	1267.6178	634.3125	13
17	1736.8701	868.9387	1719.8436	860.4254	1718.8596	859.9334	S	1228.6069	614.8071	1211.5804	606.2938	1210.5963	605.8018	12
18	1793.8916	897.4494	1776.8650	888.9362	1775.8810	888.4441	G	1141.5749	571.2911	1124.5483	562.7778	1123.5643	562.2858	11
19	1890.9443	945.9758	1873.9178	937.4625	1872.9338	936.9705	P	1084.5534	542.7803	1067.5269	534.2671	1066.5429	533.7751	10
20	1987.9971	994.5022	1970.9706	985.9889	1969.9865	985.4969	P	987.5007	494.2540	970.4741	485.7407	969.4901	485.2487	9
21	2075.0291	1038.0182	2058.0026	1029.5049	2057.0186	1029.0129	S	890.4479	445.7276	873.4213	437.2143	872.4373	436.7223	8
22	2132.0506	1066.5289	2115.0241	1058.0157	2114.0400	1057.5237	G	803.4159	402.2116	786.3893	393.6983	785.4053	393.2063	7

**23**	2233.0983	1117.0528	2216.0717	1108.5395	2215.0877	1108.0475	**T**	***746.3944***	373.7008	729.3679	365.1876	728.3838	364.6956	**6**
**24**	2290.1197	1145.5635	2273.0932	1137.0502	2272.1092	1136.5582	**G**	***645.3467***	323.1770	628.3202	314.6637			**5**
**25**	2403.2038	1202.1055	2386.1773	1193.5923	2385.1932	1193.1003	**L**	588.3253	**294.6663**	571.2987	286.1530			**4**
**26**	2540.2627	**1270.6350**	2523.2362	1262.1217	2522.2522	1261.6297	**H**	***475.2412***	238.1242	458.2146	229.6110			**3**
**27**	2696.3638	1348.6856	2679.3373	1340.1723	2678.3533	1339.6803	**R**	***338.1823***	169.5948	321.1557	161.0815			**2**
**28**							**Y**	182.0812	91.5442					**1**

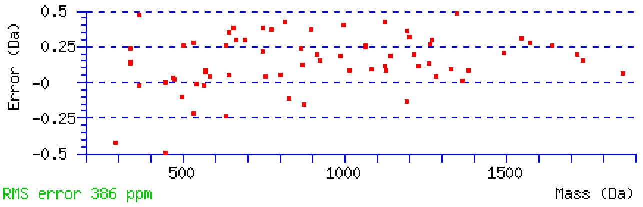

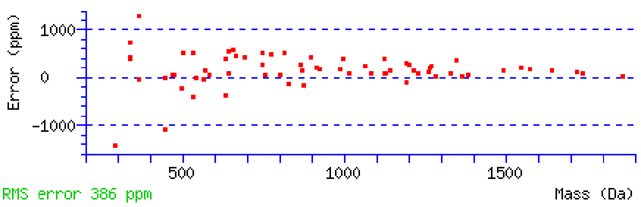

NCBI **BLAST** search of KGNDISSGTVLSDYVGSGPPSGTGLHRY
(Parameters: blastp, nr protein database, expect=20000, no filter, PAM30)
Other BLAST web gateways

All matches to this query

Score	Mr(calc)	Delta	Sequence
80.8	2876.4305	0.0279	KGNDISSGTVLSDYVGSGPPSGTGLHRY
19.8	2876.4789	-0.0205	VGMLRGVGAGMRYLSDLGYIHRDLAA
15.0	2876.4647	-0.0063	RSAQHPSSSDVVTYAPFTLFPSPVPSA
13.2	2876.3441	0.1143	DLQSLCHDLGAVGGCYPEPHKHCHM
13.2	2876.2574	0.2009	GFVMDGQQHMGIRAPGPMSGMGMNMGM
13.2	2876.2574	0.2009	GFVMDGQQHMGIRAPGPMSGMGMNMGM
11.5	2876.4010	0.0574	DVGYRHVDTAYAYQVEEEIGQAIQS
11.1	2876.6614	-0.2030	MLETYSNLVSVGYDIIKPDVIIKL
11.0	2876.4436	0.0148	TFEEEKMQLACDDLKTTEKLCES
10.4	2876.5607	-0.1023	KGRAGESLGRNWYVTPEITITDNDL

Mascot: http://www.matrixscience.com/

[redacted] Mascot Search Results

Peptide View

MS/MS Fragmentation of **SPQLEDEAKELQ**
Found in **PENK_MOUSE** in **SwissProt**, Proenkephalin-A OS=Mus musculus GN=Penk PE=1 SV=2

Match to Query 4648: 1441.734468 from(721.874510,2+) intensity(97246.6800) scans(5703) rawscans(sn5703) rtinseconds(2225.6315) index(3634)
Title: 3635: Scan 5703 (rt=2225.63) [C:\Users\Administrador\Desktop\AMOSTRAS NO ORBITRAP LEANDRO RODAR NOVAMENTE\HIPOCAMPO\hipocampo_01.raw]
Data file hipocampo_01.temp.mgf

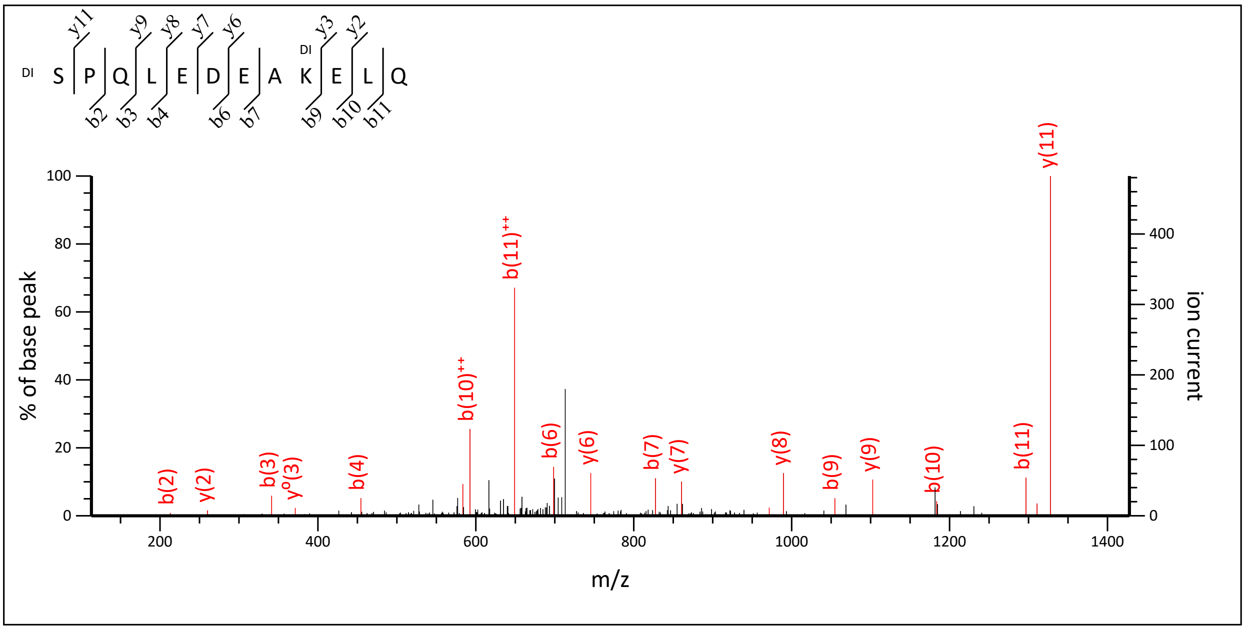

113.14 to 1427.77

Label all possible matches Label matches used for scoring

Monoisotopic mass of neutral peptide Mr(calc): 1441.7300
Variable modifications:
N-term : Dimethyl (N-term)
K9 : Dimethyl (K)
Ions Score: 91 **Expect:** 2.5e-005
Matches : 23/124 fragment ions using 23 most intense peaks (help)

#	b	b++	b*	b*++	b0	b0++	Seq.	y	y++	y*	y*++	y0	y0++	#
1	116.0706	58.5389			98.0600	49.5337	S							12
2	213.1234	107.0653			195.1128	98.0600	P	1327.6740	664.3406	1310.6474	655.8274	1309.6634	655.3353	11
3	341.1819	171.0946	324.1554	162.5813	323.1714	162.0893	Q	1230.6212	615.8142	1213.5947	607.3010	1212.6107	606.8090	10
4	454.2660	227.6366	437.2395	219.1234	436.2554	218.6314	L	1102.5626	551.7850	1085.5361	543.2717	1084.5521	542.7797	9
5	583.3086	292.1579	566.2821	283.6447	565.2980	283.1527	E	989.4786	495.2429	972.4520	486.7297	971.4680	486.2376	8
6	698.3355	349.6714	681.3090	341.1581	680.3250	340.6661	D	860.4360	430.7216	843.4094	422.2084	842.4254	421.7163	7
7	827.3781	414.1927	810.3516	405.6794	809.3676	405.1874	E	745.4090	373.2082	728.3825	364.6949	727.3985	364.2029	6
8	898.4153	449.7113	881.3887	441.1980	880.4047	440.7060	A	616.3665	308.6869	599.3399	300.1736	598.3559	299.6816	5
9	1054.5415	527.7744	1037.5150	519.2611	1036.5310	518.7691	K	545.3293	273.1683	528.3028	264.6550	527.3188	264.1630	4
10	1183.5841	592.2957	1166.5576	583.7824	1165.5735	583.2904	E	389.2031	195.1052	372.1765	186.5919	371.1925	186.0999	3
11	1296.6682	648.8377	1279.6416	640.3245	1278.6576	639.8324	L	260.1605	130.5839	243.1339	122.0706			2
12							Q	147.0764	74.0418	130.0499	65.5286			1

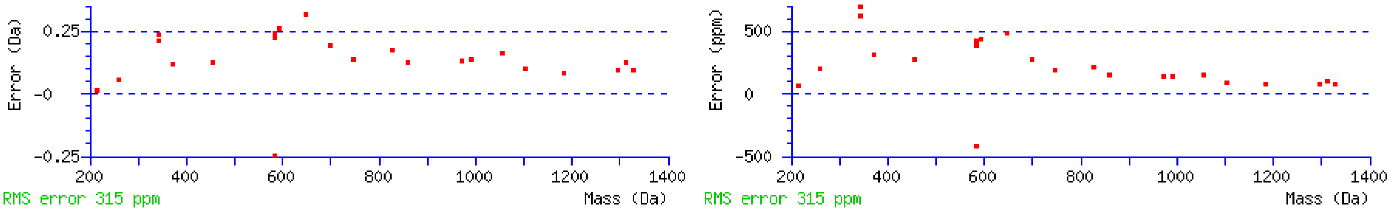

NCBI **BLAST** search of SPQLEDEAKELQ
(Parameters: blastp, nr protein database, expect=20000, no filter, PAM30)
Other BLAST web gateways

All matches to this query

Score	Mr(calc)	Delta	Sequence
90.9	1441.7300	0.0045	SPQLEDEAKELQ
33.4	1441.7966	-0.0621	INIPLDEVGEALQ
31.6	1441.7201	0.0143	SWSRSIEDLQPP
27.2	1441.7708	-0.0364	YVIPFSMGPLGSP
26.2	1441.6333	0.1012	YCNENEVGEAIQ
26.0	1441.8140	-0.0795	KAERDKSLELQ
26.0	1441.8392	-0.1047	KLEKLSAAEEIQ
26.0	1441.8252	-0.0908	RLKERSVEELQ
25.8	1441.7235	0.0110	NKLGTAMSHEIQ
25.7	1441.6543	0.0802	DYPEFPGSQNNP

Mascot: http://www.matrixscience.com/

[redacted] Mascot Search Results

Peptide View

MS/MS Fragmentation of **ASAPLVETSTPLRL**
Found in **PCSK1_MOUSE** in **SwissProt**, ProSAAS OS=Mus musculus GN=Pcsk1n PE=1 SV=2

Match to Query 5247: 1481.858568 from(741.936560,2+) intensity(17465.0230) scans(9155) rawscans(sn9155) rtinseconds(3469.1442) index(6640)
Title: 6641: Scan 9155 (rt=3469.14) [C:\Users\Administrador\Desktop\AMOSTRAS NO ORBITRAP LEANDRO RODAR NOVAMENTE\CORTEX\cortex_01.raw]
Data file cortex_01.temp.mgf

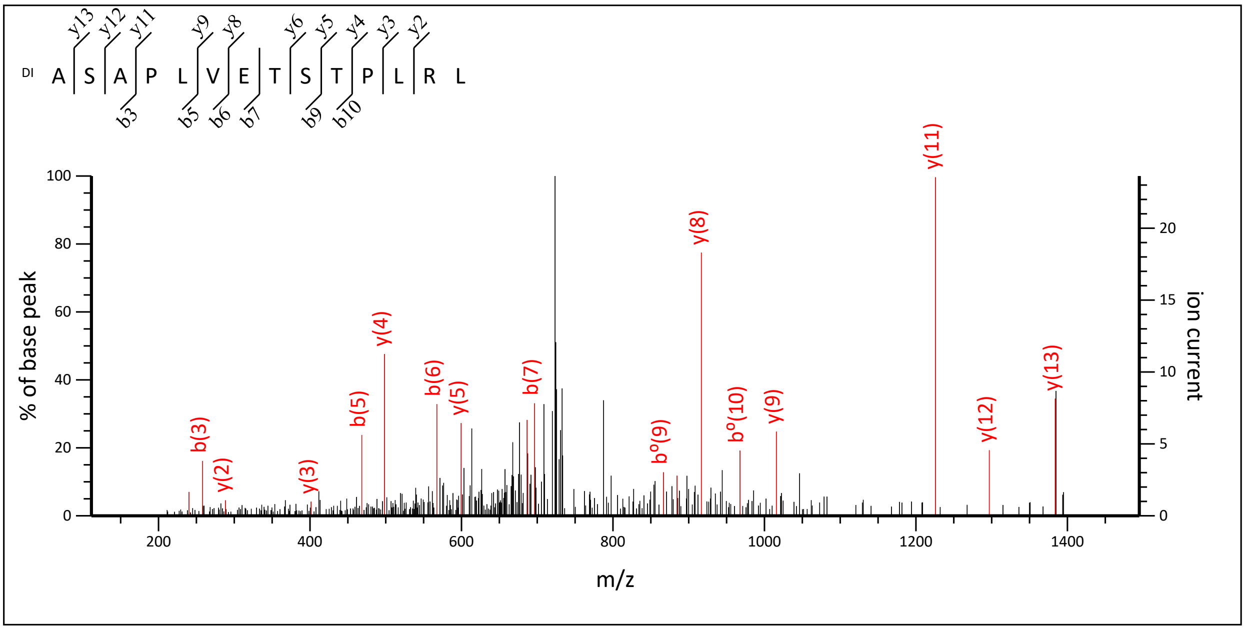

111.41 to 1494.92

Label all possible matches ○ Label matches used for scoring ◉

Monoisotopic mass of neutral peptide Mr(calc): 1481.8453
Variable modifications:
N-term : Dimethyl (N-term)
Ions Score: 67 **Expect:** 0.0056
Matches : 18/120 fragment ions using 36 most intense peaks (help)

#	b	b^{++}	b*	b^{*++}	b^{0}	b^{0++}	Seq.	y	y^{++}	y*	y^{*++}	y^{0}	y^{0++}	#
1	100.0757	50.5415					A							14
2	187.1077	94.0575			169.0972	85.0522	S	***1383.7842***	692.3957	1366.7577	683.8825	1365.7736	683.3905	13
3	**258.1448**	129.5761			240.1343	120.5708	A	***1296.7522***	648.8797	1279.7256	640.3665	1278.7416	639.8744	12
4	355.1976	178.1024			337.1870	169.0972	P	***1225.7151***	613.3612	1208.6885	604.8479	1207.7045	604.3559	11
5	**468.2817**	234.6445			450.2711	225.6392	L	1128.6623	564.8348	1111.6358	556.3215	1110.6517	555.8295	10
6	**567.3501**	284.1787			549.3395	275.1734	V	***1015.5782***	508.2928	998.5517	499.7795	997.5677	499.2875	9
7	**696.3927**	348.7000			678.3821	339.6947	E	***916.5098***	458.7585	899.4833	450.2453	898.4993	449.7533	8
8	797.4403	399.2238			779.4298	390.2185	T	787.4672	394.2373	770.4407	385.7240	769.4567	385.2320	7
9	**884.4724**	442.7398			866.4618	433.7345	S	***686.4196***	343.7134	669.3930	335.2001	668.4090	334.7081	6
10	985.5201	493.2637			967.5095	484.2584	T	***599.3875***	300.1974	582.3610	291.6841	581.3770	291.1921	5
11	1082.5728	541.7900			1064.5623	532.7848	P	***498.3398***	249.6736	481.3133	241.1603			4
12	1195.6569	598.3321			1177.6463	589.3268	L	***401.2871***	201.1472	384.2605	192.6339			3
13	1351.7580	676.3826	1334.7314	667.8694	1333.7474	667.3774	R	***288.2030***	144.6051	271.1765	136.0919			2
14							L	132.1019	66.5546					1

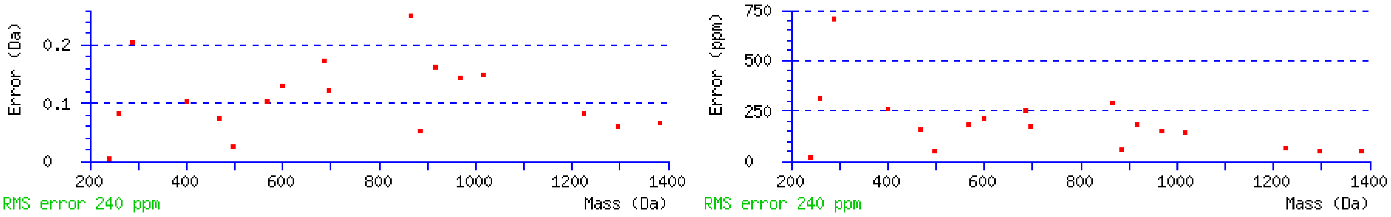

NCBI **BLAST** search of ASAPLVETSTPLRL
(Parameters: blastp, nr protein database, expect=20000, no filter, PAM30)
Other BLAST web gateways

All matches to this query

Score	Mr(calc)	Delta	Sequence
67.4	1481.8453	0.0132	ASAPLVETSTPLRL
17.4	1481.6998	0.1588	AGLPAEESDAQQAPA
16.7	1481.6646	0.1939	DGSLGSFTSTSNFM
16.6	1481.8311	0.0274	LPQVTWIVQYSA
14.8	1481.7613	0.0972	QVIEKTSGPEEPA
14.0	1481.9983	-0.1397	AVKIMEKVVKII
12.8	1481.6457	0.2129	QPCGSQGTPTSFSSA
12.8	1481.8541	0.0045	SKSVTEGLLTQQQ
12.3	1481.8326	0.0260	RACGLLSPLSAHLA
12.2	1481.7660	0.0926	ASAPLMELLHSRN

Mascot: http://www.matrixscience.com/

[redacted] Mascot Search Results

Peptide View

MS/MS Fragmentation of SLSAASAPLVETSTPLRL
Found in PCSK1_MOUSE in SwissProt, ProSAAS OS=Mus musculus GN=Pcsk1n PE=1 SV=2

Match to Query 6853: 1840.034728 from(921.024640,2+) intensity(51618.4840) scans(10414) rawscans(sn10414) rtinseconds(3896.7859) index(7623)
Title: 7624: Scan 10414 (rt=3896.79) [C:\Users\Administrador\Desktop\AMOSTRAS NO ORBITRAP LEANDRO RODAR NOVAMENTE\HIPOCAMPO\hipocampo_01.raw]
Data file hipocampo_01.temp.mgf

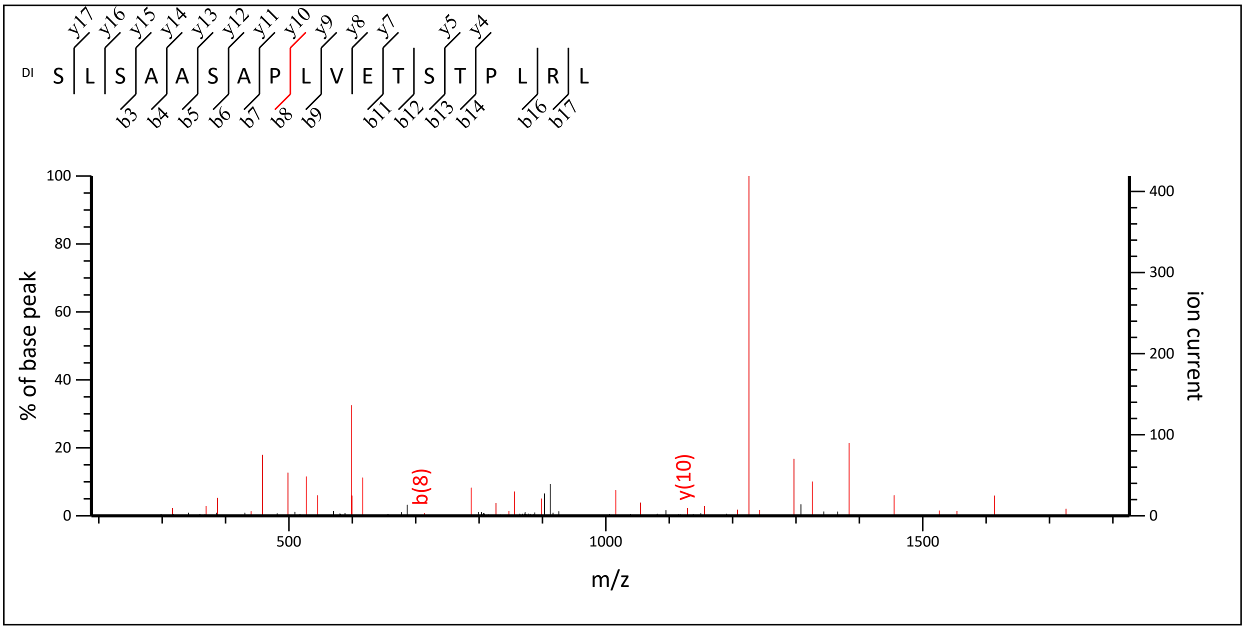

188.36 to 1826.08

Label all possible matches ○ Label matches used for scoring ◉

Monoisotopic mass of neutral peptide Mr(calc): 1840.0305
Variable modifications:
N-term : Dimethyl (N-term)
Ions Score: 125 **Expect:** 1e-008
Matches : 34/162 fragment ions using 38 most intense peaks (help)

#	b	b^{++}	b^*	b^{*++}	b^0	b^{0++}	Seq.	y	y^{++}	y^*	y^{*++}	y^0	y^{0++}	#
1	116.0706	58.5389			98.0600	49.5337	S							18
2	229.1547	115.0810			211.1441	106.0757	L	1725.9745	863.4909	1708.9480	854.9776	1707.9640	854.4856	17
3	316.1867	158.5970			298.1761	149.5917	S	1612.8905	806.9489	1595.8639	798.4356	1594.8799	797.9436	16
4	387.2238	194.1155			369.2132	185.1103	A	1525.8584	763.4329	1508.8319	754.9196	1507.8479	754.4276	15
5	458.2609	229.6341			440.2504	220.6288	A	1454.8213	727.9143	1437.7948	719.4010	1436.8108	718.9090	14
6	545.2930	273.1501			527.2824	264.1448	S	1383.7842	692.3957	1366.7577	683.8825	1365.7736	683.3905	13
7	616.3301	308.6687			598.3195	299.6634	A	1296.7522	648.8797	1279.7256	640.3665	1278.7416	639.8744	12
8	713.3828	357.1951			695.3723	348.1898	P	1225.7151	613.3612	1208.6885	604.8479	1207.7045	604.3559	11
9	826.4669	413.7371			808.4563	404.7318	L	1128.6623	564.8348	1111.6358	556.3215	1110.6517	555.8295	10
10	925.5353	463.2713			907.5247	454.2660	V	1015.5782	508.2928	998.5517	499.7795	997.5677	499.2875	9
11	1054.5779	527.7926			1036.5673	518.7873	E	916.5098	458.7585	899.4833	450.2453	898.4993	449.7533	8
12	1155.6256	578.3164			1137.6150	569.3111	T	787.4672	394.2373	770.4407	385.7240	769.4567	385.2320	7
13	1242.6576	621.8324			1224.6470	612.8272	S	686.4196	343.7134	669.3930	335.2001	668.4090	334.7081	6
14	1343.7053	672.3563			1325.6947	663.3510	T	599.3875	300.1974	582.3610	291.6841	581.3770	291.1921	5
15	1440.7581	720.8827			1422.7475	711.8774	P	498.3398	249.6736	481.3133	241.1603			4
16	1553.8421	777.4247			1535.8315	768.4194	L	401.2871	201.1472	384.2605	192.6339			3
17	1709.9432	855.4753	1692.9167	846.9620	1691.9327	846.4700	R	288.2030	144.6051	271.1765	136.0919			2
18							L	132.1019	66.5546					1

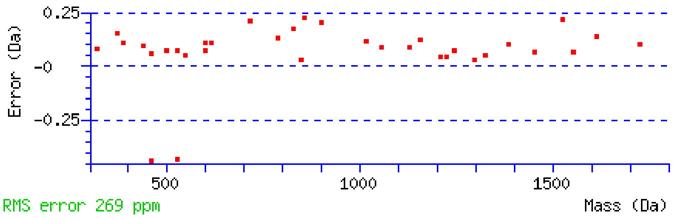

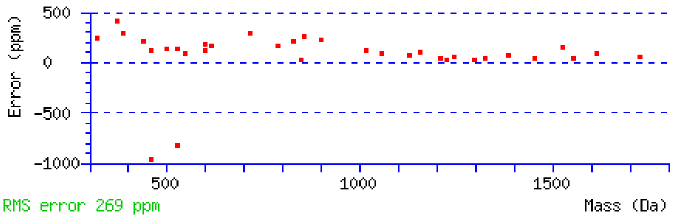

NCBI **BLAST** search of SLSAASAPLVETSTPLRL
(Parameters: blastp, nr protein database, expect=20000, no filter, PAM30)
Other BLAST web gateways

All matches to this query

Score	Mr(calc)	Delta	Sequence
124.9	1840.0305	0.0042	SLSAASAPLVETSTPLRL
30.1	1840.1246	-0.0899	PLGARRATKPLGTDRAP
29.4	1839.8879	0.1468	SSSSASSGPPGSSAASAPGIT
27.4	1840.0495	-0.0148	PPLSATVTLTIAVSDNIP
25.0	1840.1174	-0.0827	SFYVSPLKRQLVSRP
24.1	1839.8859	0.1488	QDEGPECIPPMQTVRI
23.4	1839.9563	0.0784	QLAASRGHGTCVQALTGP
23.4	1840.1497	-0.1149	ASSAKMGLVETKLAIIP
23.0	1839.9690	0.0657	EAELNTVRNQTVAASLP
22.4	1839.9880	0.0467	PGSSLPTSLTIPNPRET

Mascot: http://www.matrixscience.com/

Mascot Search Results

Peptide View

MS/MS Fragmentation of **LLDEGHYPVRESPIDTA**
Found in **SCG1_MOUSE** in **SwissProt**, Secretogranin-1 OS=Mus musculus GN=Chgb PE=1 SV=2

Match to Query 7937: 1938.985088 from(970.499820,2+) intensity(10820.4620) scans(6117) rawscans(sn6117) rtinseconds(2384.1047) index(4055)
Title: 4056: Scan 6117 (rt=2384.1) [C:\Users\Administrador\Desktop\AMOSTRAS NO ORBITRAP LEANDRO RODAR NOVAMENTE\CORTEX\cortex_01.raw]
Data file cortex_01.temp.mgf

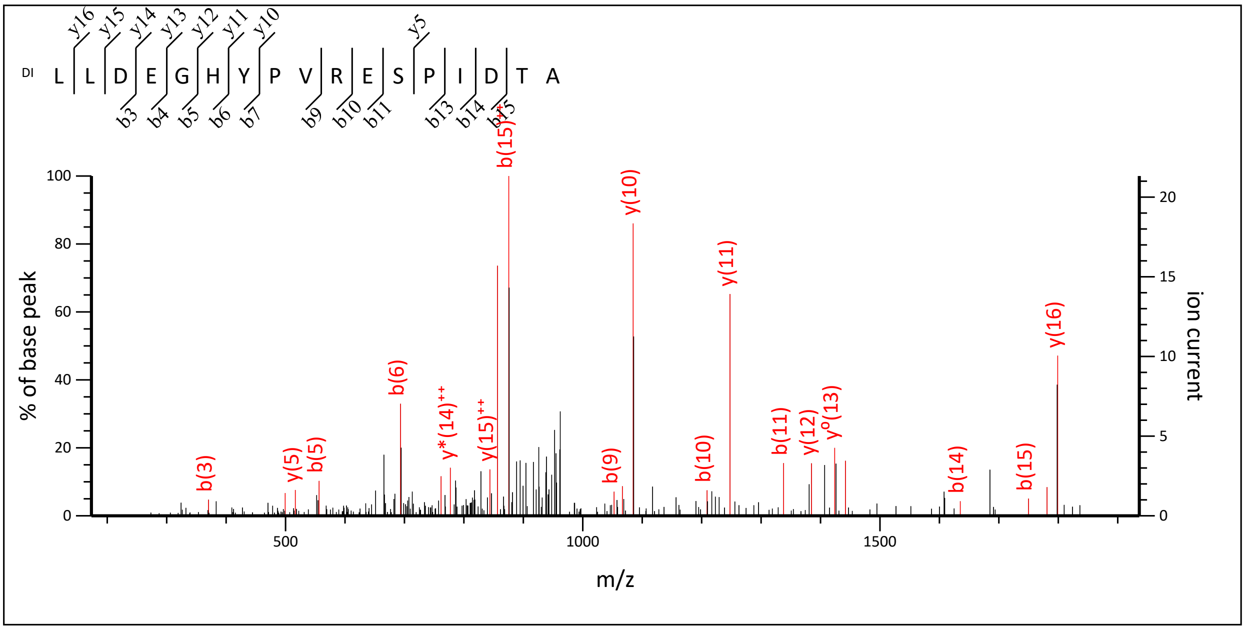

173.4 to 1936.29

Label all possible matches Label matches used for scoring

Monoisotopic mass of neutral peptide Mr(calc): 1938.9687
Variable modifications:
N-term : Dimethyl (N-term)
Ions Score: 46 **Expect:** 0.89
Matches : 24/154 fragment ions using 48 most intense peaks (help)

#	b	b^{++}	b^{*}	b^{*++}	b^{0}	b^{0++}	Seq.	y	y^{++}	y^{*}	y^{*++}	y^{0}	y^{0++}	#
1	142.1226	71.5650					L							17
2	255.2067	128.1070					L	**1798.8606**	899.9339	1781.8341	891.4207	1780.8501	890.9287	16
3	***370.2336***	185.6205			352.2231	176.6152	D	1685.7766	843.3919	1668.7500	834.8786	1667.7660	834.3866	15
4	***499.2762***	250.1418			481.2657	241.1365	E	1570.7496	785.8784	1553.7231	777.3652	1552.7390	776.8732	14
5	***556.2977***	278.6525			538.2871	269.6472	G	**1441.7070**	721.3571	1424.6805	712.8439	1423.6965	712.3519	13
6	***693.3566***	347.1819			675.3461	338.1767	H	**1384.6856**	692.8464	1367.6590	684.3331	1366.6750	683.8411	12
7	***856.4199***	428.7136			838.4094	419.7083	Y	**1247.6266**	624.3170	1230.6001	615.8037	1229.6161	615.3117	11
8	953.4727	477.2400			935.4621	468.2347	P	**1084.5633**	542.7853	1067.5368	534.2720	1066.5528	533.7800	10
9	***1052.5411***	526.7742			1034.5306	517.7689	V	987.5106	494.2589	970.4840	485.7456	969.5000	485.2536	9
10	***1208.6422***	604.8248	1191.6157	596.3115	1190.6317	595.8195	R	888.4421	444.7247	871.4156	436.2114	870.4316	435.7194	8
11	***1337.6848***	669.3461	1320.6583	660.8328	1319.6743	660.3408	E	732.3410	366.6742			714.3305	357.6689	7
12	1424.7169	712.8621	1407.6903	704.3488	1406.7063	703.8568	S	603.2984	302.1529			585.2879	293.1476	6
13	1521.7696	761.3884	1504.7431	752.8752	1503.7591	752.3832	P	**516.2664**	258.6368			498.2558	249.6316	5
14	***1634.8537***	817.9305	1617.8271	809.4172	1616.8431	808.9252	I	419.2136	210.1105			401.2031	201.1052	4
15	***1749.8806***	875.4440	1732.8541	866.9307	1731.8701	866.4387	D	306.1296	153.5684			288.1190	144.5631	3
16	1850.9283	925.9678	1833.9018	917.4545	1832.9177	916.9625	T	191.1026	96.0550			173.0921	87.0497	2
17							A	90.0550	45.5311					1

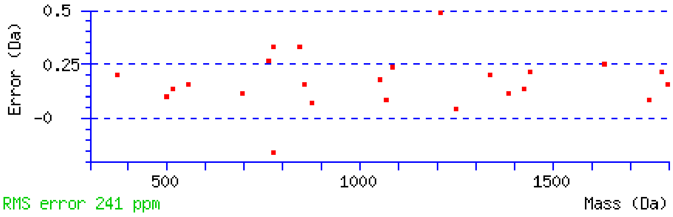

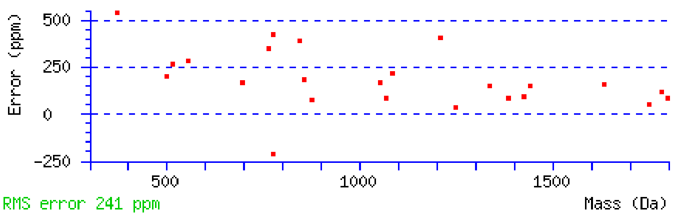

NCBI **BLAST** search of LLDEGHYPVRESPIDTA
(Parameters: blastp, nr protein database, expect=20000, no filter, PAM30)
Other BLAST web gateways

All matches to this query

Score	Mr(calc)	Delta	Sequence
45.6	1938.9687	0.0164	LLDEGHYPVRESPIDTA
10.7	1939.1393	-0.1542	TVPASAHKIDYSKRRD
10.7	1938.9662	0.0189	PPFMDGLQPGYRPVQPP
10.1	1938.9981	-0.0130	ATLSHLMMPKPPNLIM
9.9	1938.9850	0.0001	SAASPAQQLPTSHPGPGPHA
8.4	1938.8928	0.0923	QVIEVACGSHHSMALAADG
8.2	1938.9067	0.0784	LNGPFTVVVKESCDGMGD
7.8	1939.0163	-0.0312	QAPKASTGPHLANGYTTAV
7.6	1938.8853	0.0998	TMSGTHMVHMLCPSQVS
7.2	1939.1583	-0.1732	EEIPPAVAPSIPLLLPLP

Mascot: http://www.matrixscience.com/

[redacted] Mascot Search Results

Peptide View

MS/MS Fragmentation of SFARAPQLDL
Found in SCG1_MOUSE in SwissProt, Secretogranin-1 OS=Mus musculus GN=Chgb PE=1 SV=2

Match to Query 2758: 1148.654348 from(575.334450,2+) intensity(65289.3360) scans(7387) rawscans(sn7387) rtinseconds(2858.5866) index(5112)
Title: 5113: Scan 7387 (rt=2858.59) [C:\Users\Administrador\Desktop\AMOSTRAS NO ORBITRAP LEANDRO RODAR NOVAMENTE\HIPOCAMPO\hipocampo_02.raw]
Data file hipocampo_02.temp.mgf

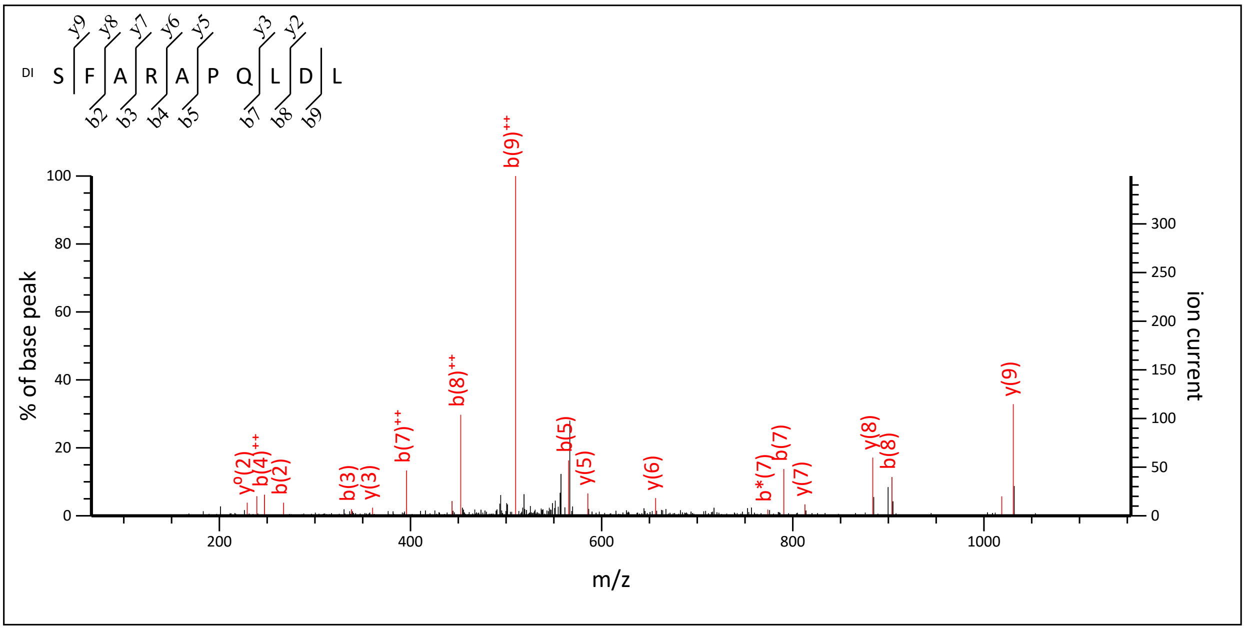

66.13 to 1153.82

Label all possible matches ○ Label matches used for scoring ◉

Monoisotopic mass of neutral peptide Mr(calc): 1148.6492
Variable modifications:
N-term : Dimethyl:2H(4) (N-term)
Ions Score: 62 **Expect:** 0.019
Matches : 22/94 fragment ions using 27 most intense peaks (help)

#	b	b^{++}	b*	b^{*++}	b^{0}	b^{0++}	Seq.	y	y^{++}	y*	y^{*++}	y^{0}	y^{0++}	#
1	120.0957	60.5515			102.0851	51.5462	S							10
2	267.1641	134.0857			249.1536	125.0804	F	1030.5680	515.7876	1013.5415	507.2744	1012.5574	506.7824	9
3	338.2012	169.6043			320.1907	160.5990	A	883.4996	442.2534	866.4730	433.7402	865.4890	433.2482	8
4	494.3023	247.6548	477.2758	239.1415	476.2918	238.6495	R	812.4625	406.7349	795.4359	398.2216	794.4519	397.7296	7
5	565.3395	283.1734	548.3129	274.6601	547.3289	274.1681	A	656.3614	328.6843	639.3348	320.1710	638.3508	319.6790	6
6	662.3922	331.6998	645.3657	323.1865	644.3817	322.6945	P	585.3243	293.1658	568.2977	284.6525	567.3137	284.1605	5
7	790.4508	395.7290	773.4243	387.2158	772.4402	386.7238	Q	488.2715	244.6394	471.2449	236.1261	470.2609	235.6341	4
8	903.5349	452.2711	886.5083	443.7578	885.5243	443.2658	L	360.2129	180.6101			342.2023	171.6048	3
9	1018.5618	509.7845	1001.5353	501.2713	1000.5512	500.7793	D	247.1288	124.0681			229.1183	115.0628	2
10							L	132.1019	66.5546					1

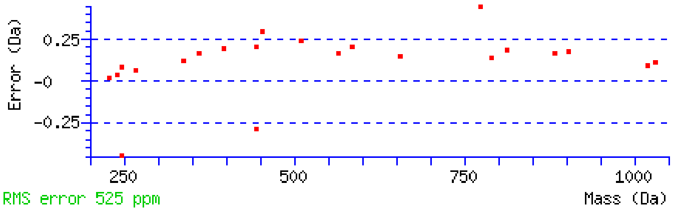

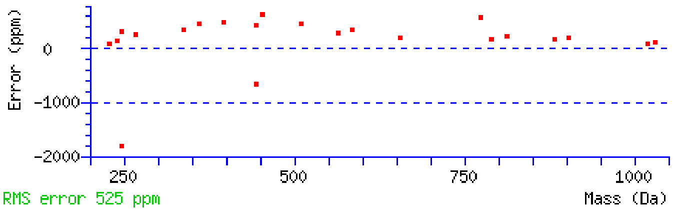

NCBI **BLAST** search of SFARAPQLDL
(Parameters: blastp, nr protein database, expect=20000, no filter, PAM30)
Other BLAST web gateways

All matches to this query

Score	Mr(calc)	Delta	Sequence
61.8	1148.6492	0.0052	SFARAPQLDL
28.6	1148.7117	-0.0574	RKQAQGGIDI
28.6	1148.6331	0.0213	RQNAQNLDL
28.3	1148.5601	0.0943	GEIYDAAIDL
28.3	1148.6077	0.0466	FGSSAIQALDL
27.4	1148.6023	0.0520	DDEDKDIDL
26.4	1148.6946	-0.0403	VDARLQALDL
25.9	1148.6569	-0.0025	VDLKEYVGY
25.9	1148.6106	0.0437	DSVDSHLLDL
25.8	1148.6892	-0.0349	KGGALAADIDI

Mascot: http://www.matrixscience.com/

[redacted] Mascot Search Results

Peptide View

MS/MS Fragmentation of ANSNPAMAPRE
Found in SMS_MOUSE in SwissProt, Somatostatin OS=Mus musculus GN=Sst PE=3 SV=1

Match to Query 4543: 1200.559268 from(601.286910,2+) intensity(22792.0880) scans(2542) rawscans(sn2542) rtinseconds(1322.4824) index(413)
Title: 414: Scan 2542 (rt=1322.48) [C:\Users\Administrador\Desktop\AMOSTRAS NO ORBITRAP LEANDRO RODAR NOVAMENTE\HIPOCAMPO\hipocampo_04.raw]
Data file hipocampo_04.temp.mgf

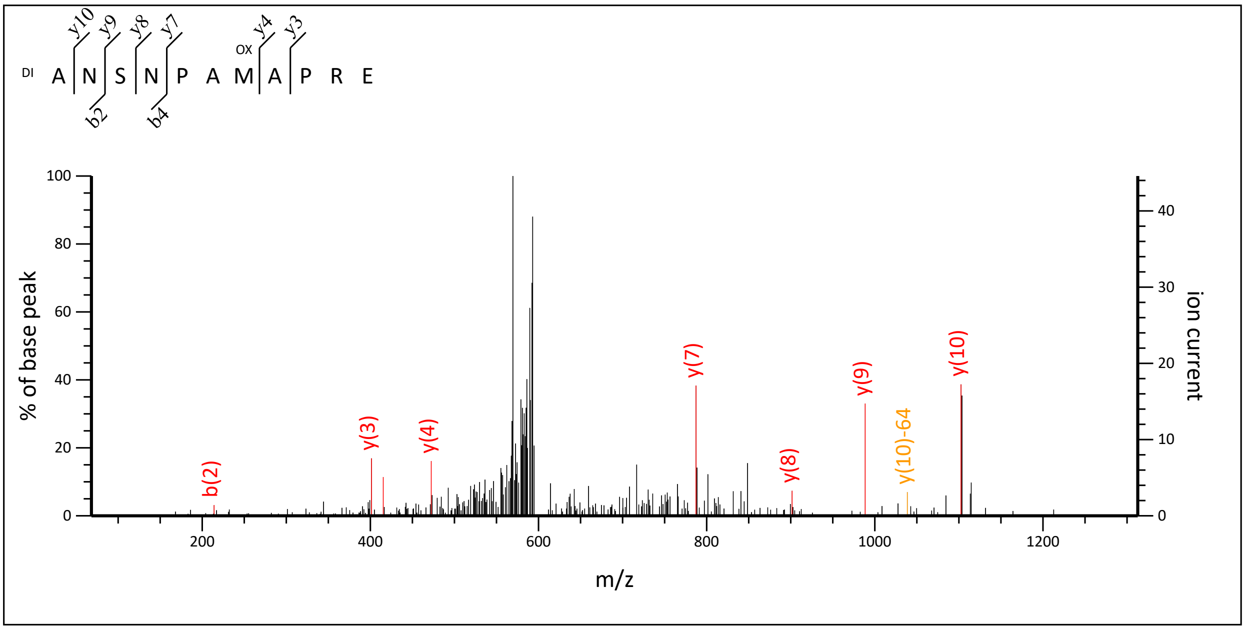

68.23 to 1312.74

Label all possible matches ○ Label matches used for scoring ◉

Monoisotopic mass of neutral peptide Mr(calc): 1200.5557
Variable modifications:
N-term : Dimethyl (N-term)
M7 : Oxidation (M), with neutral losses 0.0000(shown in table), 63.9983
Ions Score: 28 **Expect:** 48
Matches : 10/172 fragment ions using 20 most intense peaks (help)

#	b	b^{++}	b*	b^{*++}	b^{0}	b^{0++}	Seq.	y	y^{++}	y*	y^{*++}	y^{0}	y^{0++}	#
1	100.0757	50.5415					A							11
2	**214.1186**	107.5629	197.0921	99.0497			N	***1102.4946***	551.7509	1085.4680	543.2377	1084.4840	542.7456	10
3	301.1506	151.0790	284.1241	142.5657	283.1401	142.0737	S	***988.4517***	494.7295	971.4251	486.2162	970.4411	485.7242	9
4	**415.1936**	208.1004	398.1670	199.5871	397.1830	199.0951	N	***901.4196***	451.2135	884.3931	442.7002	883.4091	442.2082	8
5	512.2463	256.6268	495.2198	248.1135	494.2358	247.6215	P	***787.3767***	394.1920	770.3502	385.6787	769.3661	385.1867	7
6	583.2835	292.1454	566.2569	283.6321	565.2729	283.1401	A	690.3239	345.6656	673.2974	337.1523	672.3134	336.6603	6
7	730.3189	365.6631	713.2923	357.1498	712.3083	356.6578	M	619.2868	310.1470	602.2603	301.6338	601.2763	301.1418	5
8	801.3560	401.1816	784.3294	392.6683	783.3454	392.1763	A	***472.2514***	236.6293	455.2249	228.1161	454.2409	227.6241	4
9	898.4087	449.7080	881.3822	441.1947	880.3982	440.7027	P	***401.2143***	201.1108	384.1878	192.5975	383.2037	192.1055	3
10	1054.5098	527.7586	1037.4833	519.2453	1036.4993	518.7533	R	304.1615	152.5844	287.1350	144.0711	286.1510	143.5791	2
11							E	148.0604	74.5339			130.0499	65.5286	1

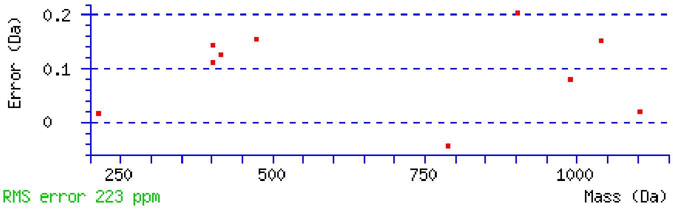

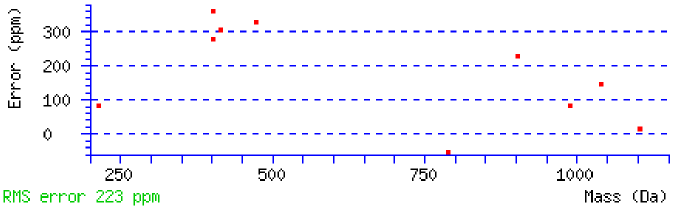

NCBI **BLAST** search of ANSNPAMAPRE
(Parameters: blastp, nr protein database, expect=20000, no filter, PAM30)
Other BLAST web gateways

All matches to this query

Score	Mr(calc)	Delta	Sequence
27.9	1200.5557	0.0036	ANSNPAMAPRE
20.5	1200.5643	-0.0050	MPGPAMPGPAMP
13.9	1200.7871	-0.2278	LLNAFTKKE
13.9	1200.5411	0.0181	PDGTQFANGPQ
13.6	1200.6208	-0.0615	PGAPPAFASPPD
13.1	1200.6176	-0.0583	PENGEEAVAVL
13.0	1200.6362	-0.0769	ILCTQPAGVPE
11.7	1200.5986	-0.0394	GAGSNPGDKTLE
11.2	1200.6462	-0.0870	ANSAGGRIVISE
10.6	1200.7566	-0.1973	LRGLCVAALVL

Mascot: http://www.matrixscience.com/

[redacted] Mascot Search Results

Peptide View

MS/MS Fragmentation of **SANSNPAMAPRE**
Found in **SMS_MOUSE** in **SwissProt**, Somatostatin OS=Mus musculus GN=Sst PE=3 SV=1

Match to Query 3640: 1275.620628 from(638.817590,2+) intensity(145209.3100) scans(2791) rawscans(sn2791) rtinseconds(1276.3717) index(1116)
Title: 1117: Scan 2791 (rt=1276.37) [C:\Users\Administrador\Desktop\AMOSTRAS NO ORBITRAP LEANDRO RODAR NOVAMENTE\HIPOCAMPO\hipocampo_01.raw]
Data file hipocampo_01.temp.mgf

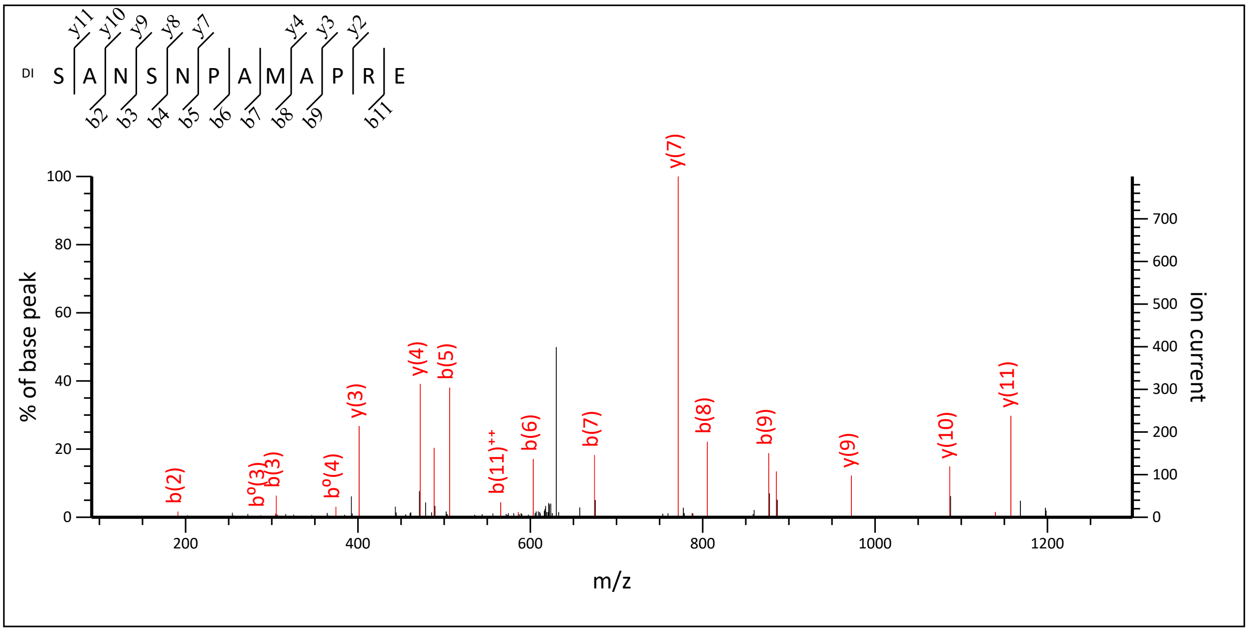

91.06 to 1298.52

Label all possible matches ○ Label matches used for scoring ◉

```
Monoisotopic mass of neutral peptide Mr(calc): 1275.6179
Variable modifications:
N-term : Dimethyl:2H(4) (N-term)
Ions Score: 77  Expect: 0.00057
Matches : 26/126 fragment ions using 36 most intense peaks   (help)
```

#	b	b^{++}	b*	b^{*++}	b^0	b^{0++}	Seq.	y	y^{++}	y*	y^{*++}	y^0	y^{0++}	#
1	120.0957	60.5515			102.0851	51.5462	S							12
2	**191.1328**	96.0701			173.1223	87.0648	A	***1157.5368***	579.2720	1140.5102	570.7588	1139.5262	570.2667	11
3	**305.1758**	153.0915	288.1492	144.5782	287.1652	144.0862	N	***1086.4997***	543.7535	1069.4731	535.2402	1068.4891	534.7482	10
4	392.2078	196.6075	375.1812	188.0943	374.1972	187.6022	S	***972.4567***	486.7320	955.4302	478.2187	954.4462	477.7267	9
5	**506.2507**	253.6290	489.2242	245.1157	488.2401	244.6237	N	***885.4247***	443.2160	868.3982	434.7027	867.4141	434.2107	8
6	**603.3035**	302.1554	586.2769	293.6421	585.2929	293.1501	P	***771.3818***	386.1945	754.3552	377.6813	753.3712	377.1892	7
7	**674.3406**	337.6739	657.3140	329.1607	656.3300	328.6686	A	***674.3290***	337.6681	657.3025	329.1549	656.3185	328.6629	6
8	**805.3811**	403.1942	788.3545	394.6809	787.3705	394.1889	M	***603.2919***	302.1496	586.2654	293.6363	585.2813	293.1443	5
9	**876.4182**	438.7127	859.3916	430.1995	858.4076	429.7074	A	***472.2514***	236.6293	455.2249	228.1161	454.2409	227.6241	4
10	973.4709	487.2391	956.4444	478.7258	955.4604	478.2338	P	***401.2143***	201.1108	384.1878	192.5975	383.2037	192.1055	3
11	1129.5721	565.2897	1112.5455	556.7764	1111.5615	556.2844	R	***304.1615***	152.5844	287.1350	144.0711	286.1510	143.5791	2
12							E	148.0604	74.5339			130.0499	65.5286	1

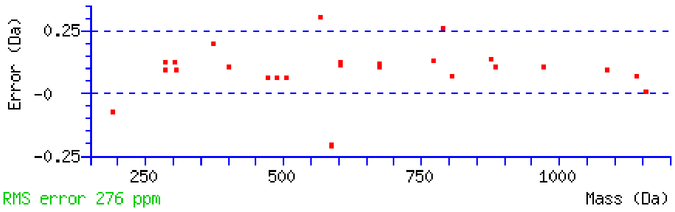

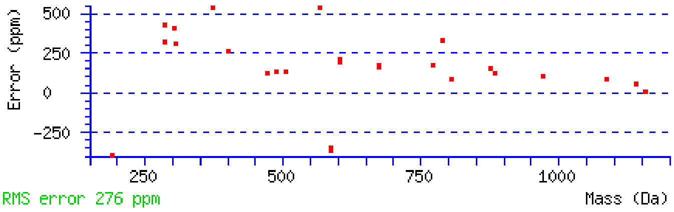

NCBI **BLAST** search of SANSNPAMAPRE
(Parameters: blastp, nr protein database, expect=20000, no filter, PAM30)
Other BLAST web gateways

All matches to this query

Score	Mr(calc)	Delta	Sequence
77.2	1275.6179	0.0027	SANSNPAMAPRE
35.8	1275.6245	-0.0038	SANTHDIDSLAT
29.6	1275.7237	-0.1031	SANSINWARLL
29.2	1275.7972	-0.1766	KSLLGSLNSNAT
24.4	1275.6319	-0.0113	DNLPPVCDTVAT
24.3	1275.6964	-0.0758	RLTGLDAGHSAT
23.8	1275.7150	-0.0944	ILNALGQNCPR
23.7	1275.5475	0.0731	CAAGVCVPGEEAT
23.7	1275.6132	0.0074	SAPSPLDASDGLD
23.7	1275.6327	-0.0121	SAIAMPAPMMQP

Mascot: http://www.matrixscience.com/

[redacted] Mascot Search Results

Peptide View

MS/MS Fragmentation of **SANSNPAMAPRE**
Found in **SMS_MOUSE** in **SwissProt**, Somatostatin OS=Mus musculus GN=Sst PE=3 SV=1

Match to Query 3751: 1291.617208 from(646.815880,2+) intensity(53846.9410) scans(2268) rawscans(sn2268) rtinseconds(1091.4888) index(642)
Title: 643: Scan 2268 (rt=1091.49) [C:\Users\Administrador\Desktop\AMOSTRAS NO ORBITRAP LEANDRO RODAR NOVAMENTE\HIPOCAMPO\hipocampo_01.raw]
Data file hipocampo_01.temp.mgf

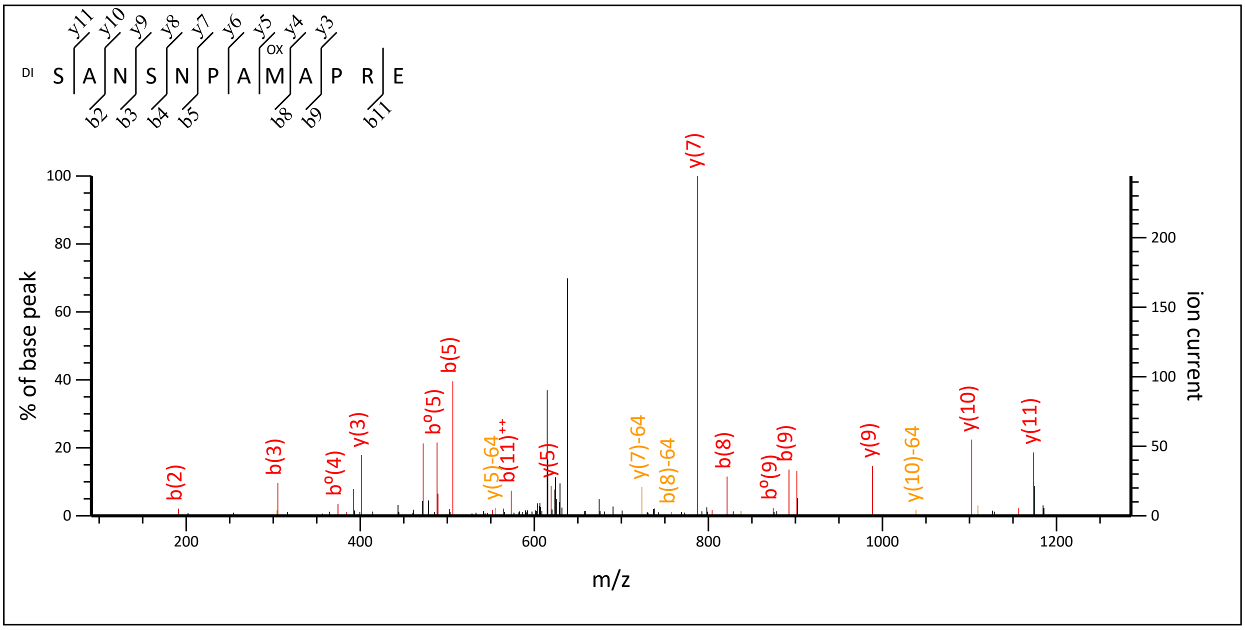

91.11 to 1285.53

Label all possible matches ○ Label matches used for scoring ◉

```
Monoisotopic mass of neutral peptide Mr(calc): 1291.6128
Variable modifications:
N-term : Dimethyl:2H(4) (N-term)
M8     : Oxidation (M), with neutral losses 0.0000(shown in table), 63.9983
Ions Score: 61  Expect: 0.022
Matches : 35/192 fragment ions using 50 most intense peaks   (help)
```

#	b	b^{++}	b*	b^{*++}	b^0	b^{0++}	Seq.	y	y^{++}	y*	y^{*++}	y^0	y^{0++}	#
1	120.0957	60.5515			102.0851	51.5462	S							12
2	**191.1328**	96.0701			173.1223	87.0648	A	***1173.5317***	587.2695	1156.5051	578.7562	1155.5211	578.2642	11
3	**305.1758**	153.0915	288.1492	144.5782	287.1652	144.0862	N	***1102.4946***	551.7509	1085.4680	543.2377	1084.4840	542.7456	10
4	**392.2078**	196.6075	375.1812	188.0943	374.1972	187.6022	S	***988.4517***	494.7295	971.4251	486.2162	970.4411	485.7242	9
5	**506.2507**	253.6290	489.2242	245.1157	488.2401	244.6237	N	***901.4196***	451.2135	884.3931	442.7002	883.4091	442.2082	8
6	603.3035	302.1554	586.2769	293.6421	585.2929	293.1501	P	***787.3767***	394.1920	770.3502	385.6787	769.3661	385.1867	7
7	674.3406	337.6739	657.3140	329.1607	656.3300	328.6686	A	690.3239	345.6656	673.2974	337.1523	672.3134	336.6603	6
8	**821.3760**	411.1916	804.3494	402.6784	803.3654	402.1863	M	***619.2868***	310.1470	602.2603	301.6338	601.2763	301.1418	5
9	**892.4131**	446.7102	875.3865	438.1969	874.4025	437.7049	A	***472.2514***	236.6293	455.2249	228.1161	454.2409	227.6241	4
10	989.4659	495.2366	972.4393	486.7233	971.4553	486.2313	P	***401.2143***	201.1108	384.1878	192.5975	383.2037	192.1055	3
11	1145.5670	573.2871	1128.5404	564.7739	1127.5564	564.2818	R	***304.1615***	152.5844	287.1350	144.0711	286.1510	143.5791	2
12							E	148.0604	74.5339			130.0499	65.5286	1

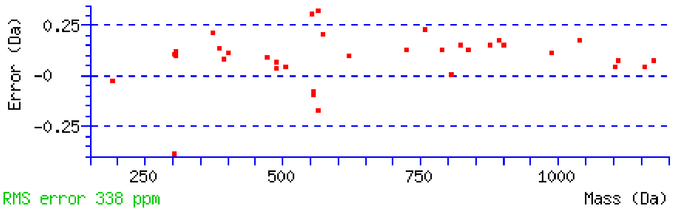

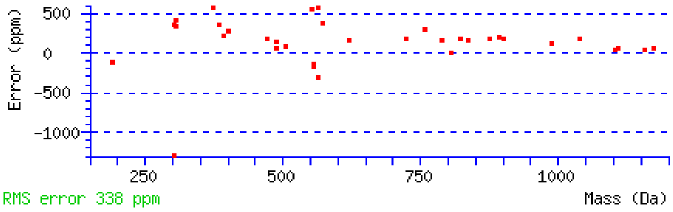

NCBI **BLAST** search of SANSNPAMAPRE
(Parameters: blastp, nr protein database, expect=20000, no filter, PAM30)
Other BLAST web gateways

All matches to this query

Score	Mr(calc)	Delta	Sequence
61.3	1291.6128	0.0044	SANSNPAMAPRE
22.3	1291.6997	-0.0825	VVRFTRSGGNAT
20.7	1291.5951	0.0221	SGICINCQHNTA
20.4	1291.5966	0.0207	SENLCGLSDLAT
20.0	1291.6053	0.0119	QMPPGMTSPRF
19.4	1291.4961	0.1211	MYHGMNPSNGD
18.9	1291.6521	-0.0349	FKDALRNSGGDG
18.9	1291.6158	0.0014	FSYYGVMALTA
18.9	1291.8860	-0.2688	KEKSKSNNTA
18.9	1291.6244	-0.0072	KGQTHENSNDG

Mascot: http://www.matrixscience.com/

Mascot Search Results

Peptide View

MS/MS Fragmentation of **AGGPPHPQLNKS**
Found in **SYN1_MOUSE** in **SwissProt**, Synapsin-1 OS=Mus musculus GN=Syn1 PE=1 SV=2

Match to Query 3599: 1257.689248 from(629.851900,2+) intensity(45609.9800) scans(2659) rawscans(sn2659) rtinseconds(1240.2501) index(1039)
Title: 1040: Scan 2659 (rt=1240.25) [C:\Users\Administrador\Desktop\AMOSTRAS NO ORBITRAP LEANDRO RODAR NOVAMENTE\CORTEX\cortex_01.raw]
Data file cortex_01.temp.mgf

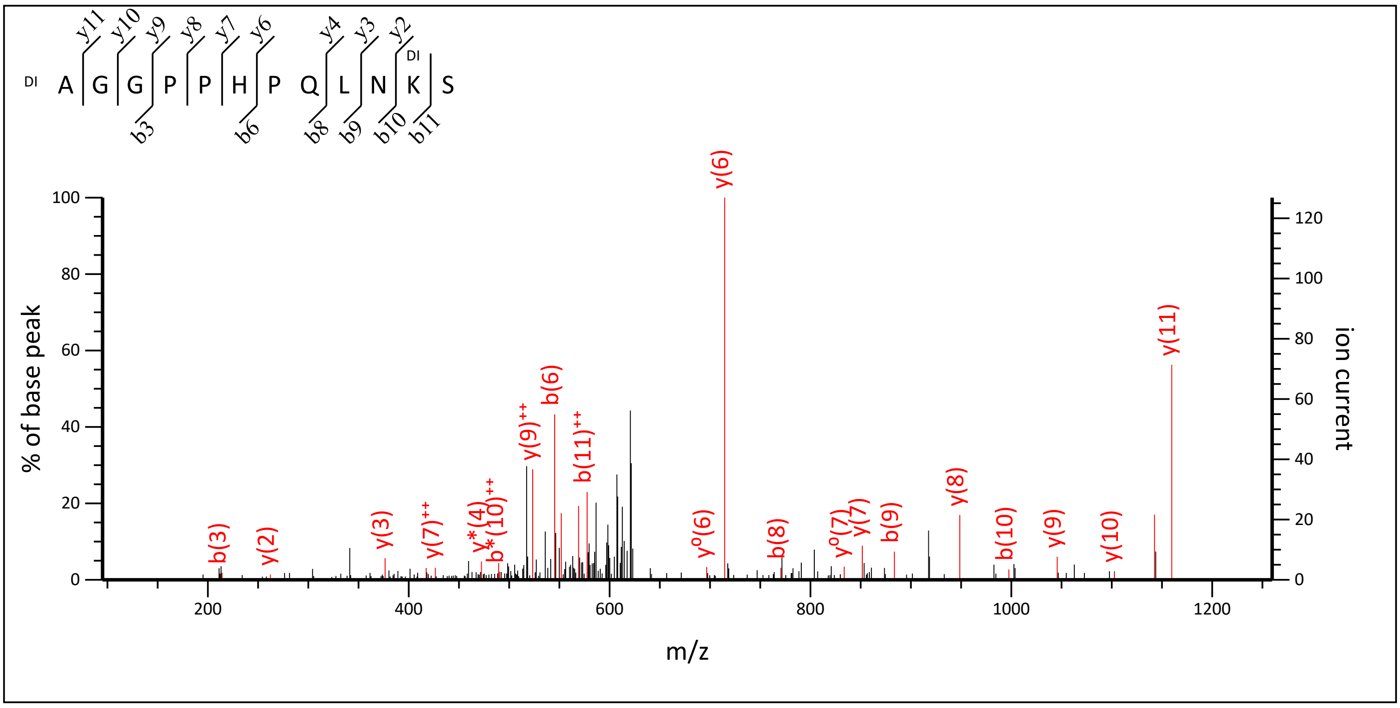

95.15 to 1259.69

Label all possible matches ○ Label matches used for scoring ◉

Monoisotopic mass of neutral peptide Mr(calc): 1257.6830
Variable modifications:
N-term : Dimethyl (N-term)
K11 : Dimethyl (K)
Ions Score: 42 **Expect:** 2
Matches : 26/94 fragment ions using 68 most intense peaks (help)

#	b	b^{++}	b*	b^{*++}	Seq.	y	y^{++}	y*	y^{*++}	y^0	y^{0++}	#
1	100.0757	50.5415			A							12
2	157.0972	79.0522			G	1159.6218	580.3146	1142.5953	571.8013	1141.6113	571.3093	11
3	214.1186	107.5629			G	1102.6004	551.8038	1085.5738	543.2905	1084.5898	542.7985	10
4	311.1714	156.0893			P	1045.5789	523.2931	1028.5524	514.7798	1027.5683	514.2878	9
5	408.2241	204.6157			P	948.5261	474.7667	931.4996	466.2534	930.5156	465.7614	8
6	545.2831	273.1452			H	851.4734	426.2403	834.4468	417.7271	833.4628	417.2350	7
7	642.3358	321.6715			P	714.4145	357.7109	697.3879	349.1976	696.4039	348.7056	6
8	770.3944	385.7008	753.3679	377.1876	Q	617.3617	309.1845	600.3352	300.6712	599.3511	300.1792	5
9	883.4785	442.2429	866.4519	433.7296	L	489.3031	245.1552	472.2766	236.6419	471.2926	236.1499	4
10	997.5214	499.2643	980.4948	490.7511	N	376.2191	188.6132	359.1925	180.0999	358.2085	179.6079	3
11	1153.6477	577.3275	1136.6211	568.8142	K	262.1761	131.5917	245.1496	123.0784	244.1656	122.5864	2
12					S	106.0499	53.5286			88.0393	44.5233	1

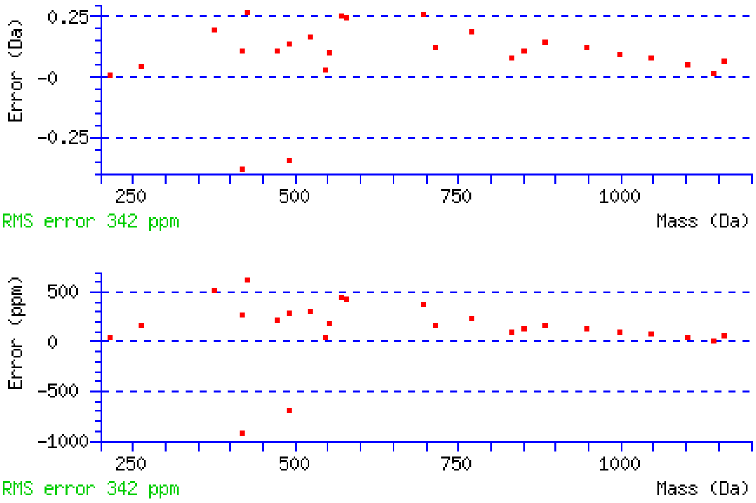

NCBI **BLAST** search of AGGPPHPQLNKS
(Parameters: blastp, nr protein database, expect=20000, no filter, PAM30)
Other BLAST web gateways

All matches to this query

Score	Mr(calc)	Delta	Sequence
41.9	1257.6830	0.0063	AGGPPHPQLNKS
18.9	1257.5949	0.0943	AGGQEAEGSAPTR
17.6	1257.6928	-0.0036	ALEESRKLADA
17.4	1257.5858	0.1035	PMQMNQALFM
16.6	1257.5520	0.1372	ANSRGLSDNCHG
13.8	1257.7921	-0.1029	ANPPRLGPKKP
13.7	1257.5877	0.1015	ALQDVPGEEWS
13.5	1257.6288	0.0604	APHGLPLCTGQH
13.0	1257.6354	0.0539	ANPPTTGAPPGPGP
12.6	1257.6201	0.0692	ALQDAASGLQEQ

Mascot: http://www.matrixscience.com/

[redacted] Mascot Search Results

Peptide View

MS/MS Fragmentation of SGPFGQIFRPDNF
Found in TBB4B_MOUSE in SwissProt, Tubulin beta-4B chain OS=Mus musculus GN=Tubb4b PE=1 SV=1

Match to Query 5040: 1508.746868 from(755.380710,2+) intensity(24802.6890) scans(10611) rawscans(sn10611) rtinseconds(3967.8521) index(7807)
Title: 7808: Scan 10611 (rt=3967.85) [C:\Users\Administrador\Desktop\AMOSTRAS NO ORBITRAP LEANDRO RODAR NOVAMENTE\HIPOCAMPO\hipocampo_01.raw]
Data file hipocampo_01.temp.mgf

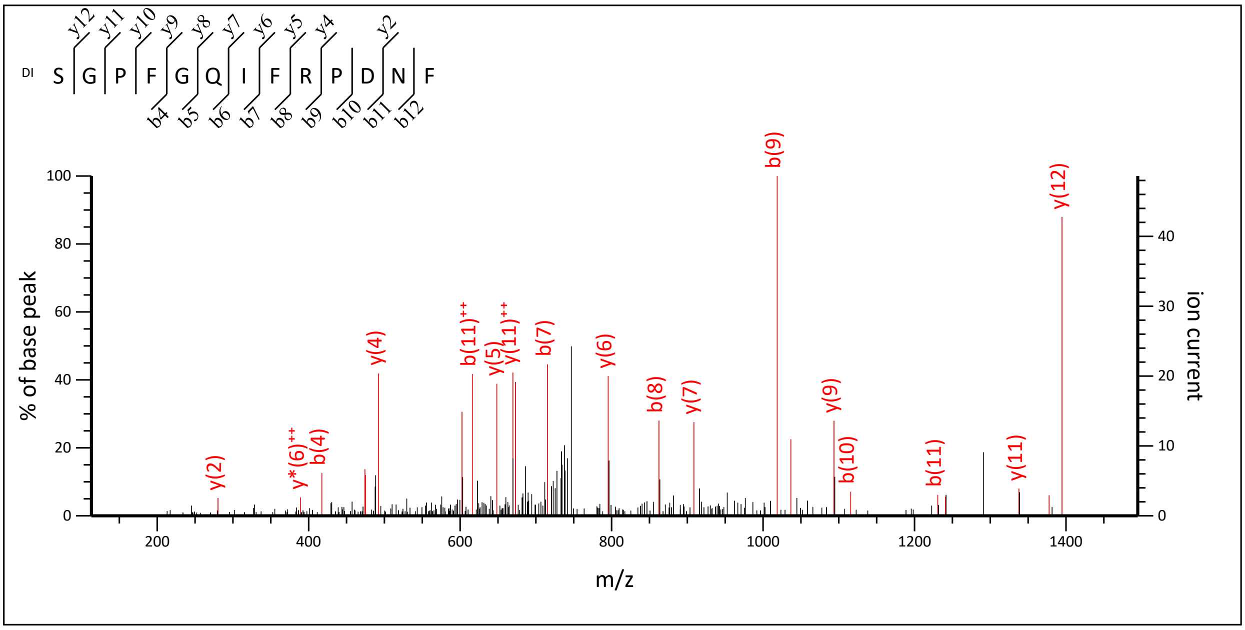

113.14 to 1494.76

Label all possible matches ○ Label matches used for scoring ◉

Monoisotopic mass of neutral peptide Mr(calc): 1508.7412
Variable modifications:
N-term : Dimethyl (N-term)
Ions Score: 57 **Expect:** 0.055
Matches : 27/128 fragment ions using 48 most intense peaks (help)

#	b	b^{++}	b*	b^{*++}	b^0	b^{0++}	Seq.	y	y^{++}	y*	y^{*++}	y^0	y^{0++}	#
1	116.0706	58.5389			98.0600	49.5337	S							13
2	173.0921	87.0497			155.0815	78.0444	G	1394.6852	697.8462	1377.6586	689.3329	1376.6746	688.8409	12
3	270.1448	135.5761			252.1343	126.5708	P	1337.6637	669.3355	1320.6371	660.8222	1319.6531	660.3302	11
4	417.2132	209.1103			399.2027	200.1050	F	1240.6109	620.8091	1223.5844	612.2958	1222.6004	611.8038	10
5	474.2347	237.6210			456.2241	228.6157	G	1093.5425	547.2749	1076.5160	538.7616	1075.5320	538.2696	9
6	602.2933	301.6503	585.2667	293.1370	584.2827	292.6450	Q	1036.5211	518.7642	1019.4945	510.2509	1018.5105	509.7589	8
7	715.3774	358.1923	698.3508	349.6790	697.3668	349.1870	I	908.4625	454.7349	891.4359	446.2216	890.4519	445.7296	7
8	862.4458	431.7265	845.4192	423.2132	844.4352	422.7212	F	795.3784	398.1928	778.3519	389.6796	777.3678	389.1876	6
9	1018.5469	509.7771	1001.5203	501.2638	1000.5363	500.7718	R	648.3100	324.6586	631.2835	316.1454	630.2994	315.6534	5
10	1115.5996	558.3035	1098.5731	549.7902	1097.5891	549.2982	P	492.2089	246.6081	475.1823	238.0948	474.1983	237.6028	4
11	1230.6266	615.8169	1213.6000	607.3037	1212.6160	606.8116	D	395.1561	198.0817	378.1296	189.5684	377.1456	189.0764	3
12	1344.6695	672.8384	1327.6430	664.3251	1326.6589	663.8331	N	280.1292	140.5682	263.1026	132.0550			2
13							F	166.0863	83.5468					1

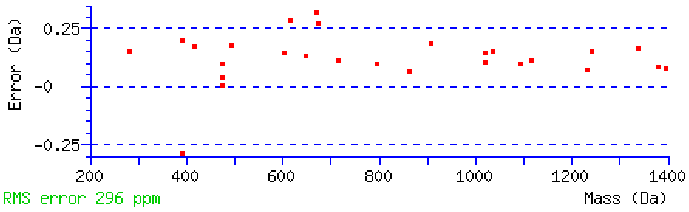

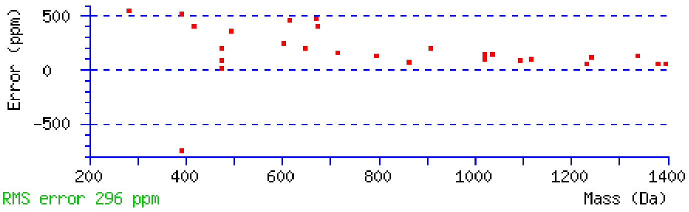

NCBI **BLAST** search of SGPFGQIFRPDNF
(Parameters: blastp, nr protein database, expect=20000, no filter, PAM30)
Other BLAST web gateways

All matches to this query

Score	Mr(calc)	Delta	Sequence
57.4	1508.7412	0.0056	SGPFGQIFRPDNF
57.4	1508.7412	0.0056	SGPFGQLFRPDNF
21.5	1508.7708	-0.0239	NNRGTIPGPPMGPGP
20.8	1508.7694	-0.0225	IVTAGQLSLCSEEQ
18.1	1508.9249	-0.1781	PRTTSRPSVPDKP
18.1	1508.6631	0.0838	TASSGYASVQGADEP
17.7	1508.7657	-0.0188	TPQHNMGLQLSVV
17.0	1508.8162	-0.0694	GRANGMGVIGQDGLL
16.8	1508.7318	0.0150	SSDSSRLVSASDDK
16.5	1508.7475	-0.0006	DPWGTVVARCSEGP

Mascot: http://www.matrixscience.com/

[redacted] Mascot Search Results

Peptide View

MS/MS Fragmentation of **DDEESEAQGPK**
Found in **TBB3_MOUSE** in **SwissProt**, Tubulin beta-3 chain OS=Mus musculus GN=Tubb3 PE=1 SV=1

Match to Query 3515: 1259.557768 from(630.786160,2+) intensity(12710.6270) scans(2116) rawscans(sn2116) rtinseconds(1035.1652) index(502)
Title: 503: Scan 2116 (rt=1035.17) [C:\Users\Administrador\Desktop\AMOSTRAS NO ORBITRAP LEANDRO RODAR NOVAMENTE\HIPOCAMPO\hipocampo_01.raw]
Data file hipocampo_01.temp.mgf

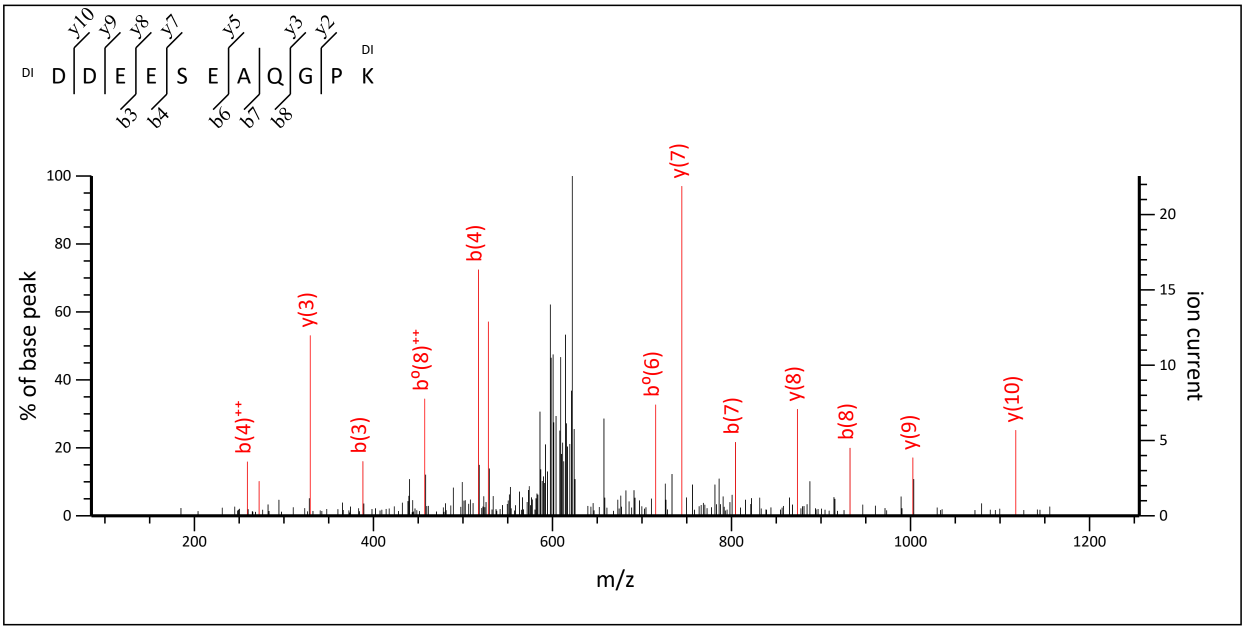

84.93 to 1255.58

Label all possible matches ○ Label matches used for scoring ◉

Monoisotopic mass of neutral peptide Mr(calc): 1259.5517
Variable modifications:
N-term : Dimethyl (N-term)
K11 : Dimethyl (K)
Ions Score: 64 **Expect:** 0.011
Matches : 17/96 fragment ions using 20 most intense peaks (help)

#	b	b^{++}	b*	b^{*++}	b^0	b^{0++}	Seq.	y	y^{++}	y*	y^{*++}	y^0	y^{0++}	#
1	144.0655	72.5364			126.0550	63.5311	D							11
2	**259.0925**	130.0499			241.0819	121.0446	D	***1117.5008***	559.2540	1100.4742	550.7408	1099.4902	550.2487	10
3	**388.1351**	194.5712			370.1245	185.5659	E	***1002.4738***	501.7406	985.4473	493.2273	984.4633	492.7353	9
4	**517.1776**	259.0925			499.1671	250.0872	E	***873.4312***	437.2193	856.4047	428.7060	855.4207	428.2140	8
5	604.2097	302.6085			586.1991	293.6032	S	***744.3886***	372.6980	727.3621	364.1847	726.3781	363.6927	7
6	733.2523	367.1298			715.2417	358.1245	E	657.3566	329.1819	640.3301	320.6687	639.3461	320.1767	6
7	**804.2894**	402.6483			786.2788	393.6430	A	***528.3140***	264.6607	511.2875	256.1474			5
8	**932.3480**	466.6776	915.3214	458.1643	914.3374	457.6723	Q	***457.2769***	229.1421	440.2504	220.6288			4
9	989.3694	495.1884	972.3429	486.6751	971.3589	486.1831	G	***329.2183***	165.1128	312.1918	156.5995			3
10	1086.4222	543.7147	1069.3956	535.2015	1068.4116	534.7095	P	***272.1969***	136.6021	255.1703	128.0888			2
11							K	175.1441	88.0757	158.1176	79.5624			1

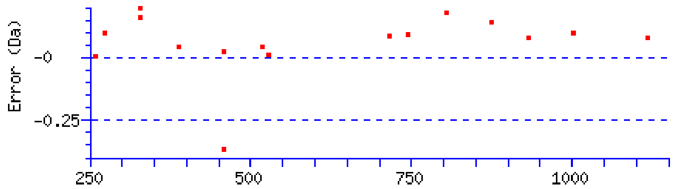

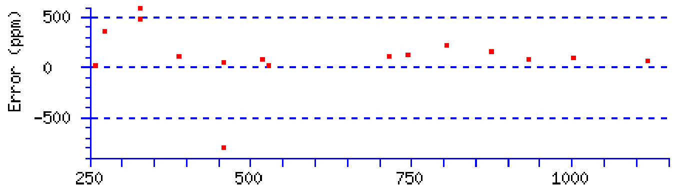

NCBI **BLAST** search of DDEESEAQGPK
(Parameters: blastp, nr protein database, expect=20000, no filter, PAM30)
Other BLAST web gateways

All matches to this query

Score	Mr(calc)	Delta	Sequence
64.3	1259.5517	0.0060	DDEESEAQGPK
37.5	1259.6183	-0.0605	SPEESTVQVPN
26.3	1259.7296	-0.1718	QKAGASEEQALG
25.7	1259.6795	-0.1218	LVSEALCSTKGP
25.3	1259.5266	0.0312	EGGGQAEEQAAEG
24.0	1259.5340	0.0238	DDEEESHKCL
23.8	1259.6357	-0.0780	DDEEEQKRK
21.5	1259.6544	-0.0966	DNAMGKAVITAN
21.5	1259.5266	0.0312	DDEESVDGNRP
21.3	1259.5380	0.0198	DDEEMKFQF

Mascot: http://www.matrixscience.com/

Diagram of Dimethyl labeling

Samples		Isotopic Labeling			Liquid cromatography and mass spectrometry
		H_2CO $NaBH_3CN$		D_2CO $NaBH_3CN$	
Cortex 1	=	WT1	+	KO1	LC/MS cortex run1
Cortex 2	=	WT2	+	KO2	LC/MS cortex run2
Cortex 3	=	KO3	+	WT3	LC/MS cortex run3
Hippocampus 1	=	WT1	+	KO1	LC/MS hippocampus run1
Hippocampus 2	=	KO2	+	WT2	LC/MS hippocampus run2
Hippocampus 3	=	KO3	+	WT3	LC/MS hippocampus run3
Striatum 1	=	WT1	+	KO1	LC/MS striatum run1
Striatum 2	=	KO2	+	WT2	LC/MS striatum run2
Striatum 3	=	KO3	+	WT3	LC/MS striatum run3

Dimethyl Isotopic labeling scheme used for relative quantification of peptides extracts from prefrontal cortex, striatum and hippocampus of THOP1$^{-/-}$**mice and C57BL/69 wild type mice.** For isotopic labeling with Formaldehyde tags were used two isotopic forms (H_2CO and D_2CO) plus $NaBH_3CN$ and the final product of these reactions adds 28 or 32 Da to the final mass of peptides at each available (lysine or N-terminal) labeling site. Peptides extracted from each brain area (KO and WT samples) were labeled and pooled as indicated in three different runs. In some runs the labeling scheme was reverse.

RPTLNELGISTPEELGLDKV 3+

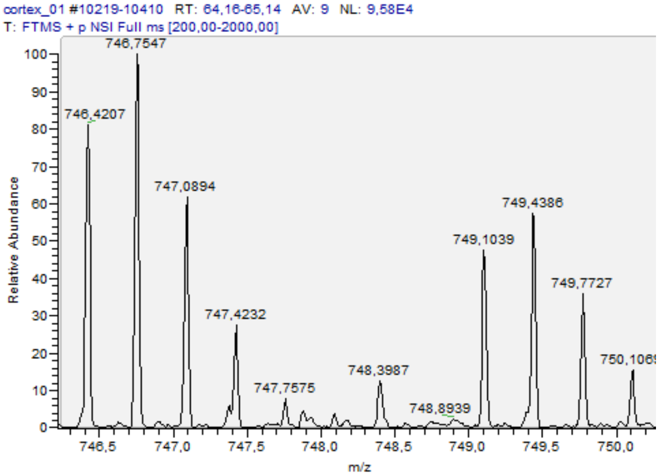

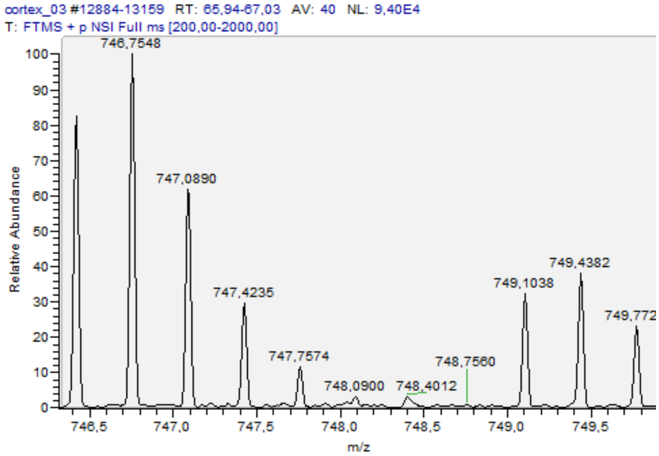

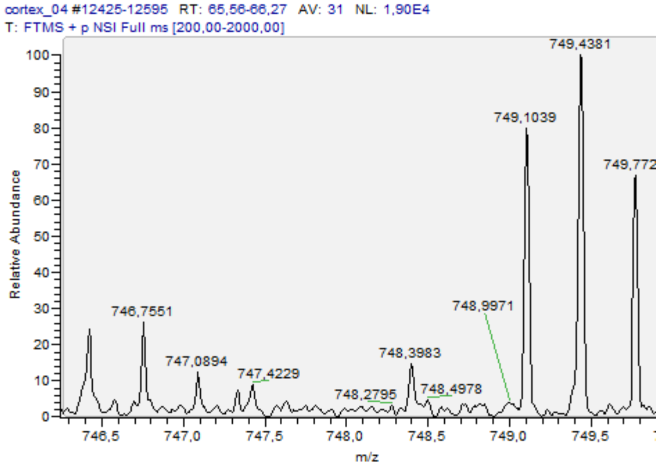

SGQAAARPLVATV 2+

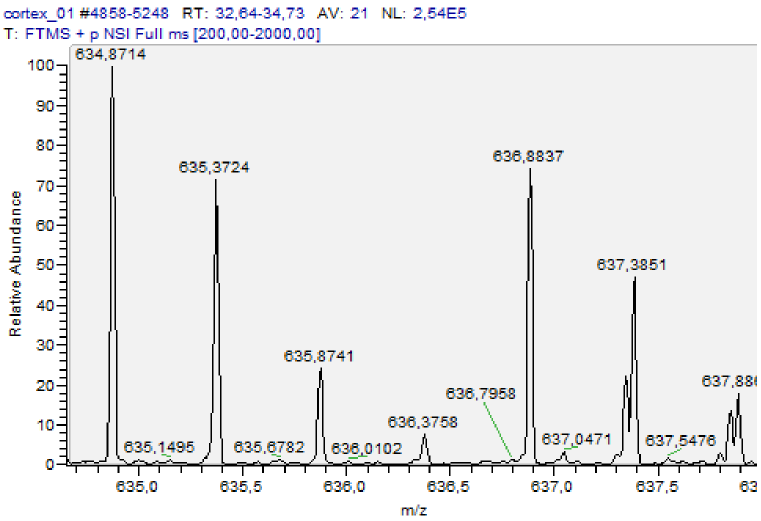

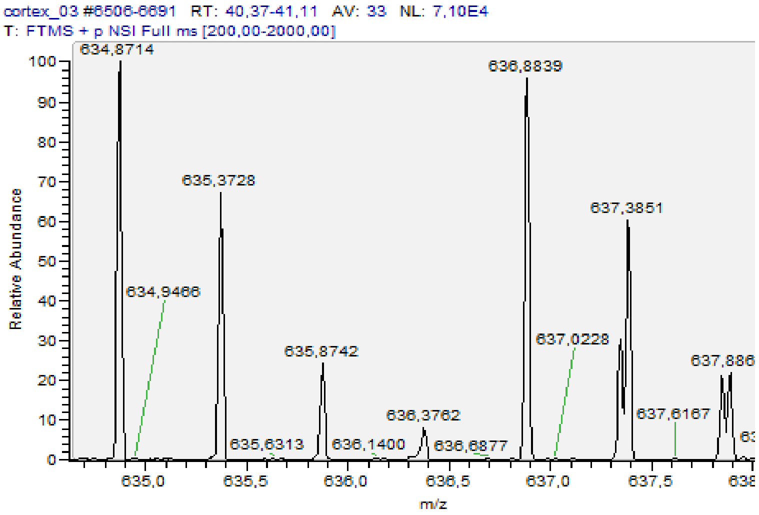

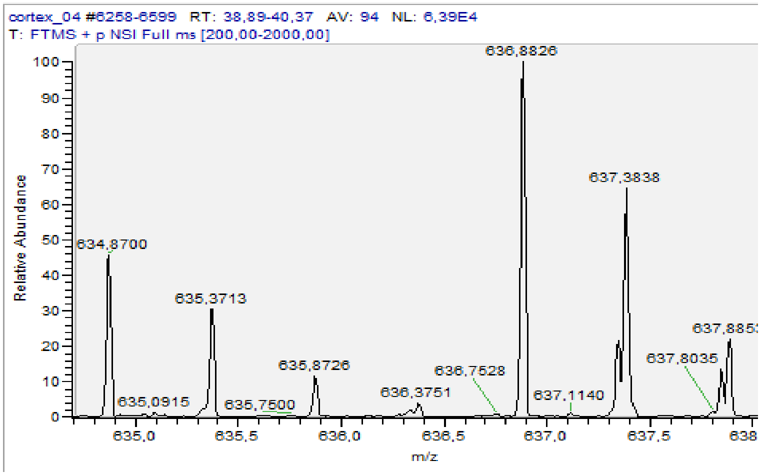

GLNVPASVRF 2+

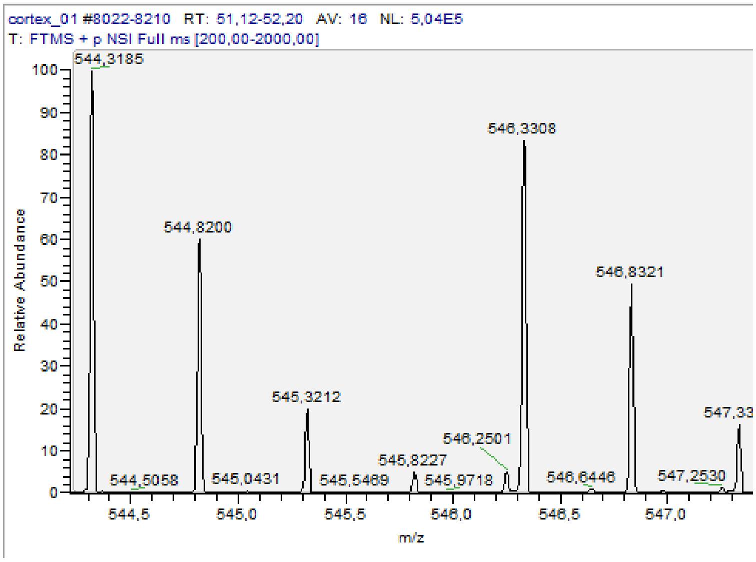

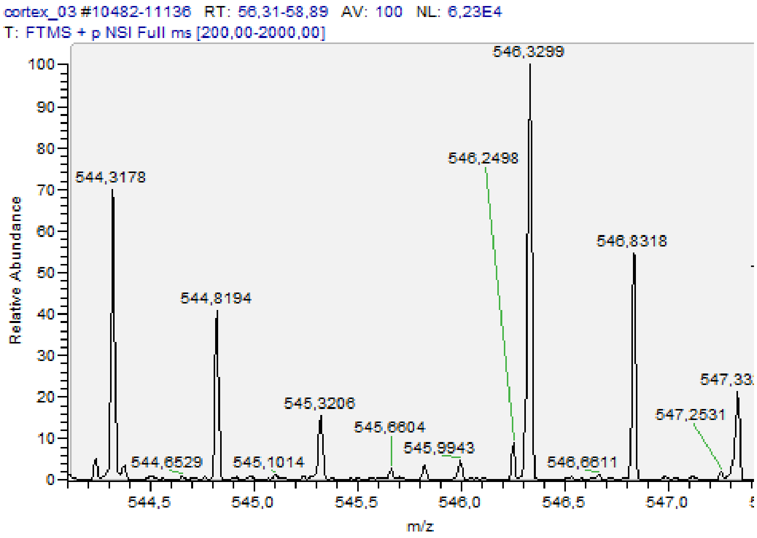

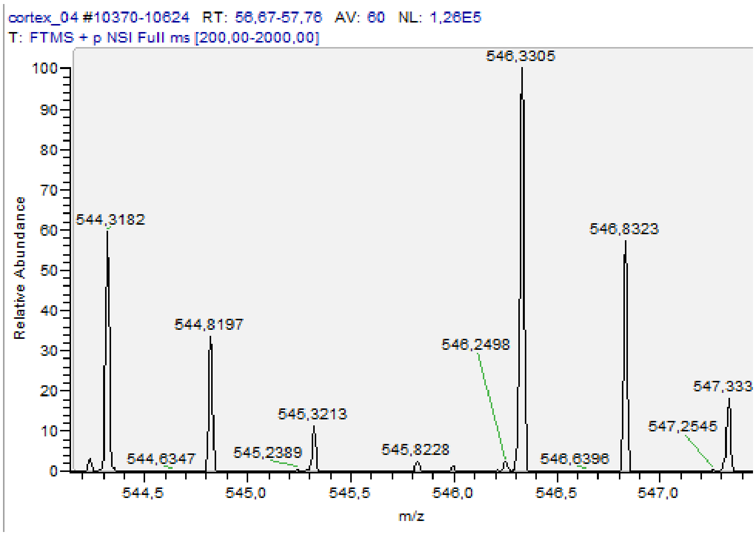

TVGLNVPASVRF 2+

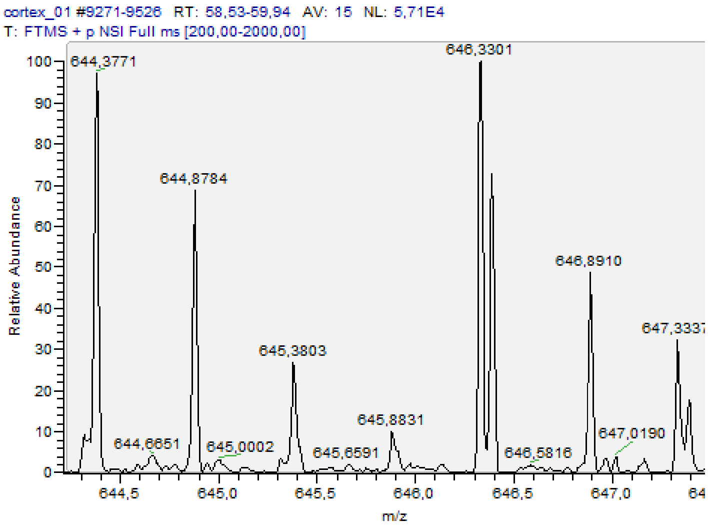

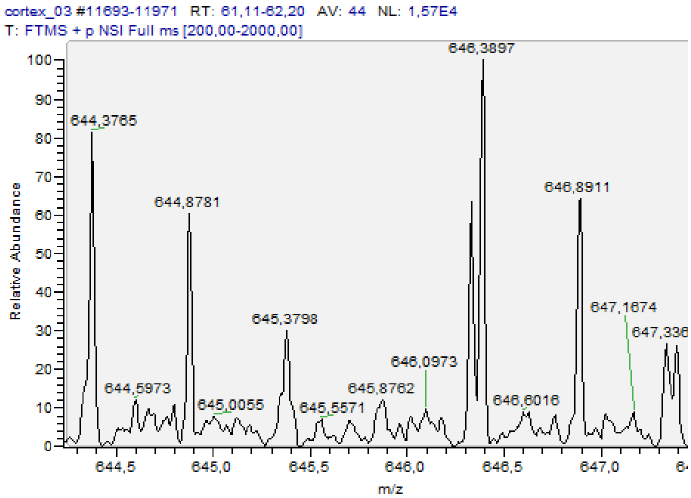

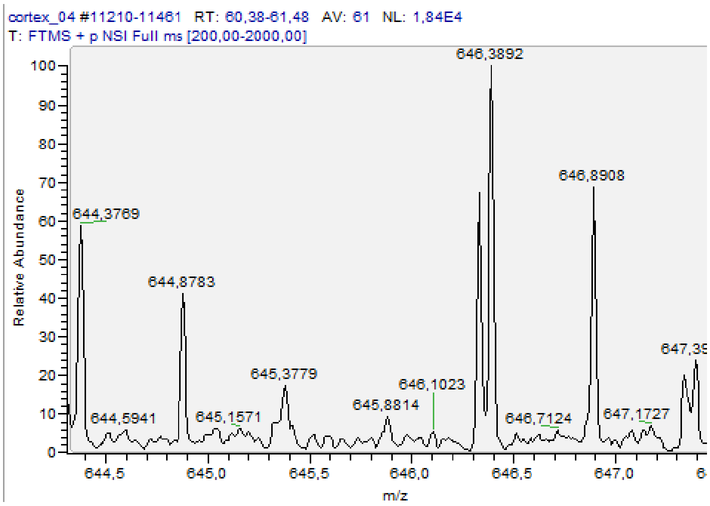

ARSGPFAPVLSAT 2+

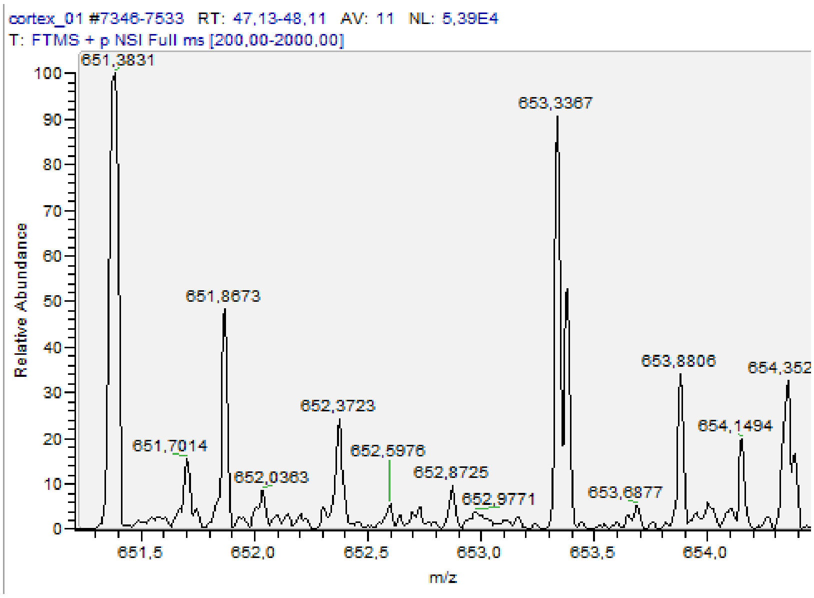

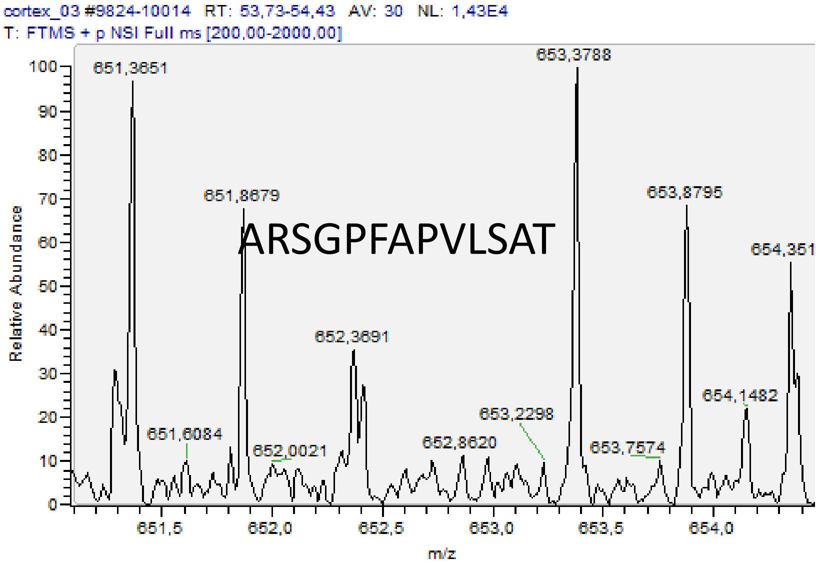

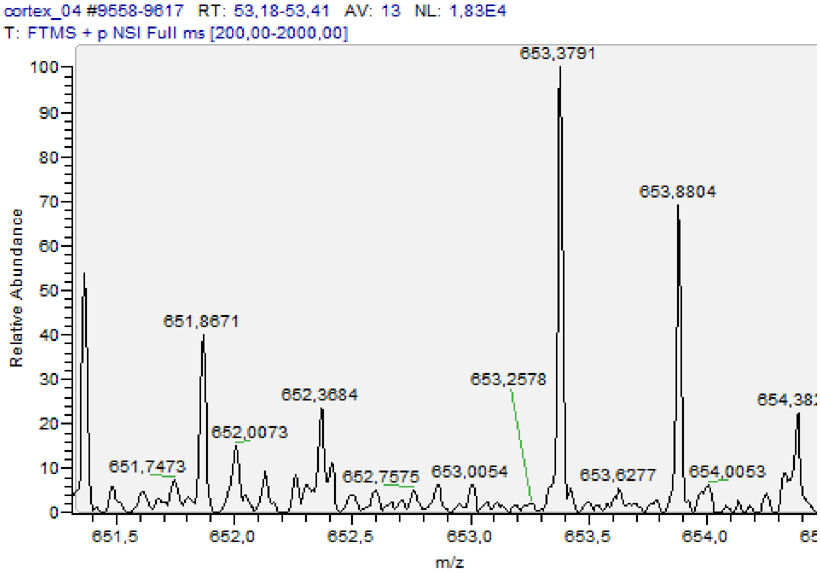

ATVGDVNTDRPGLLDL 2+

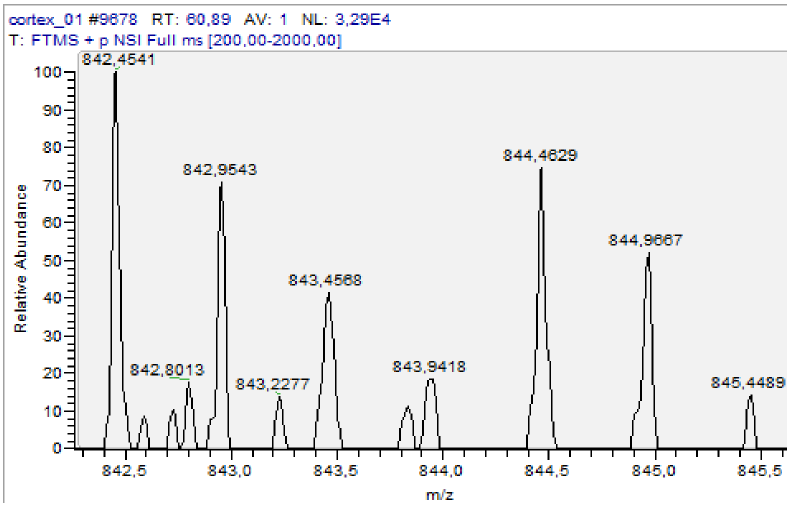

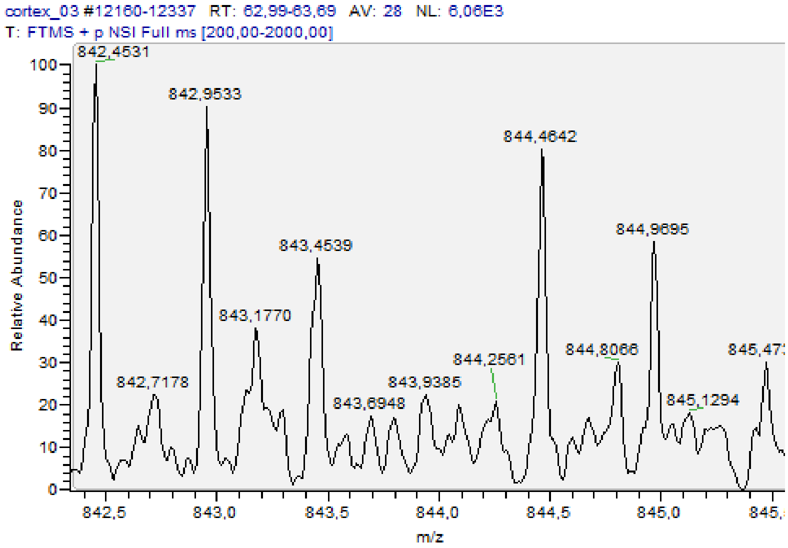

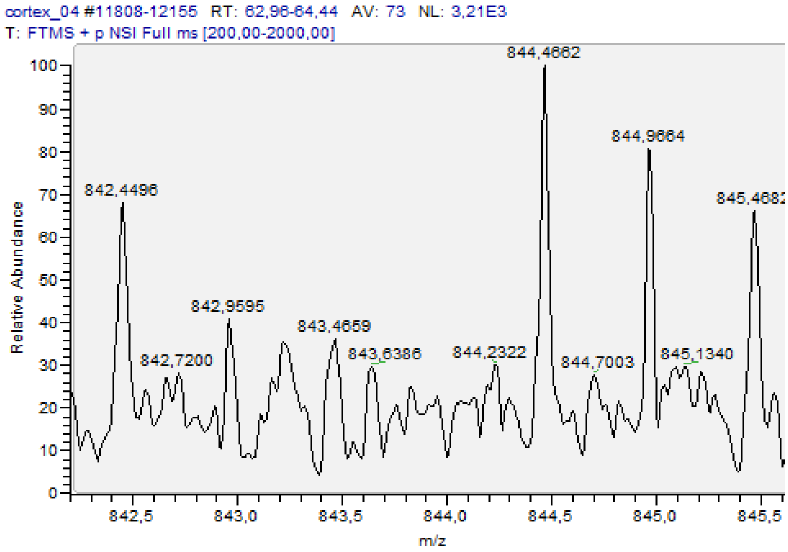

VEKVDELKKKYGI 2+

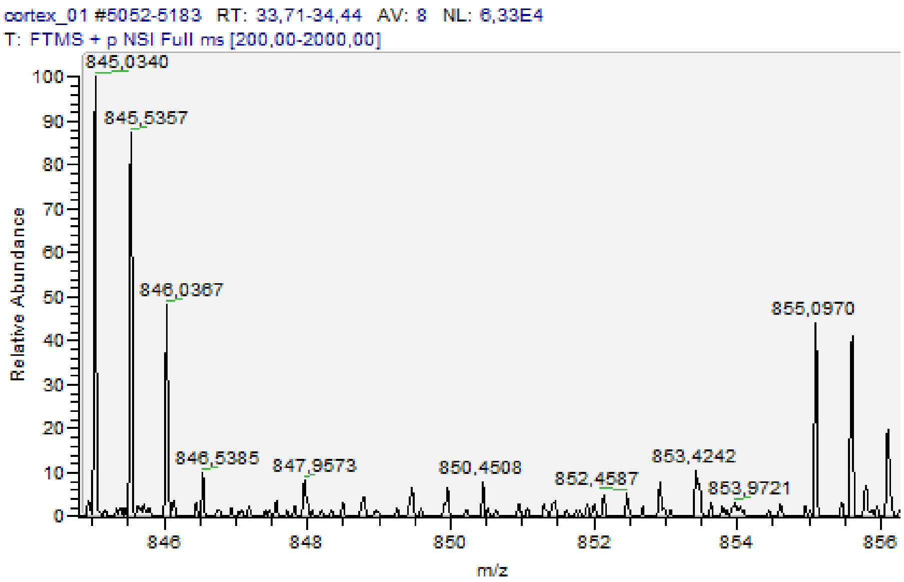

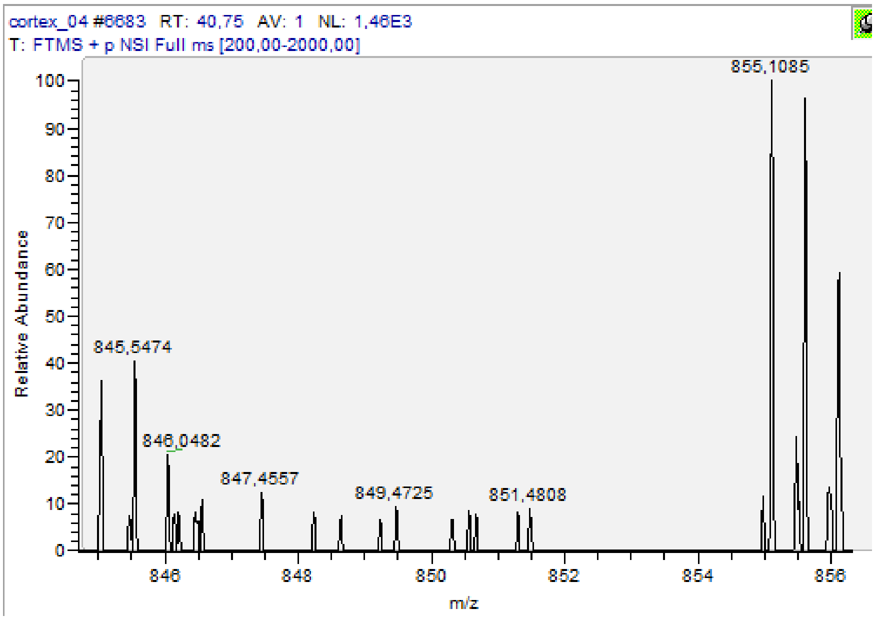

VEKVDELKKKYGI 3+

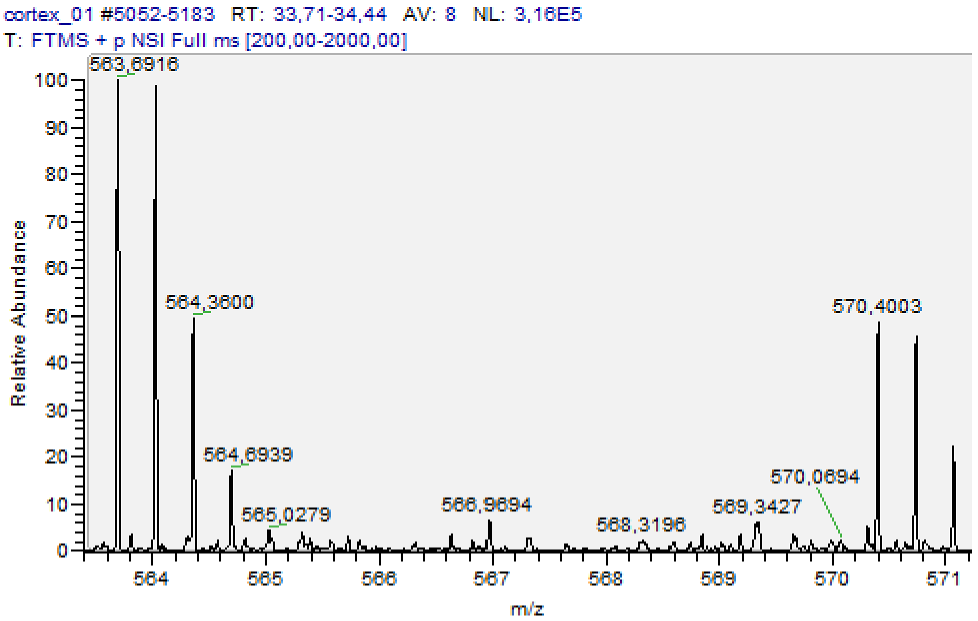

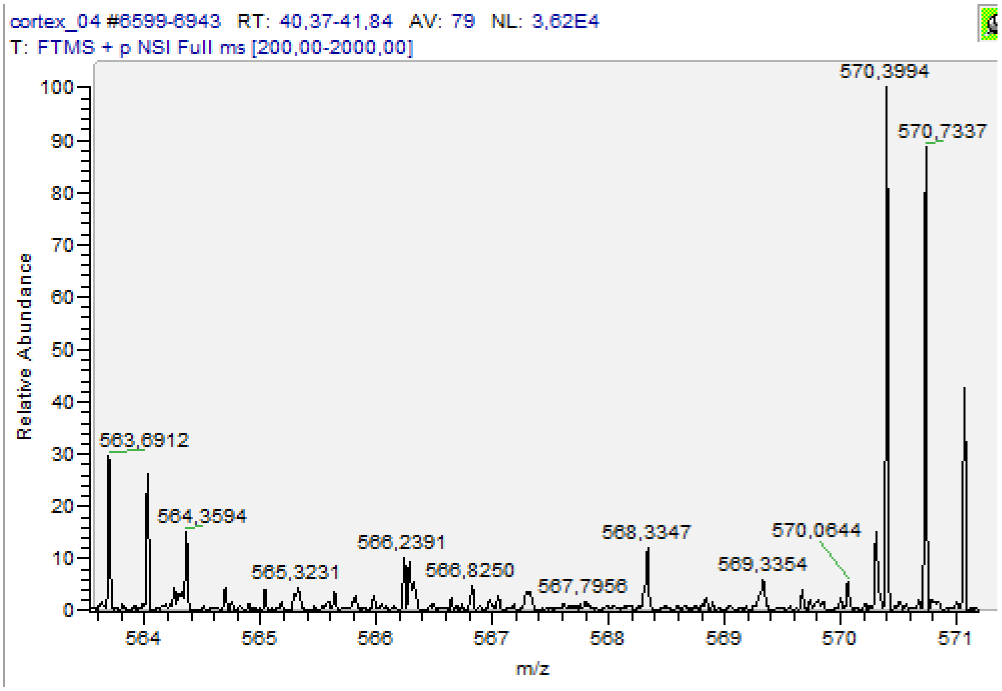

ASKGLGSDLDSSLASL 2+

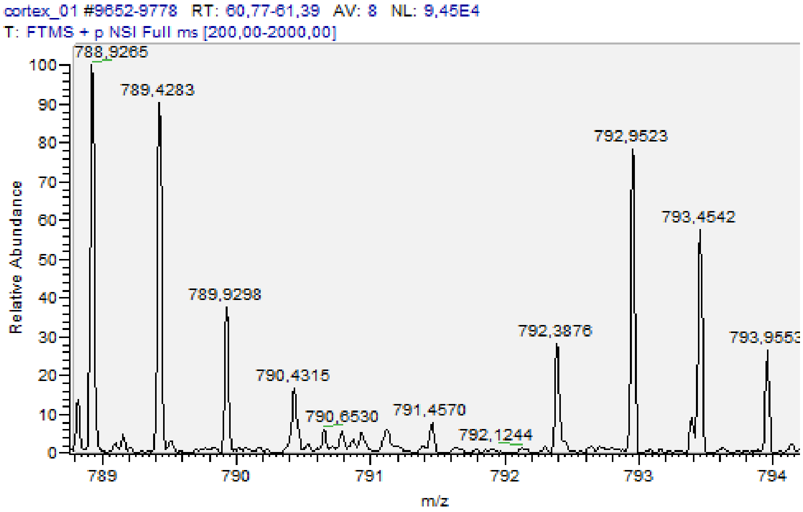

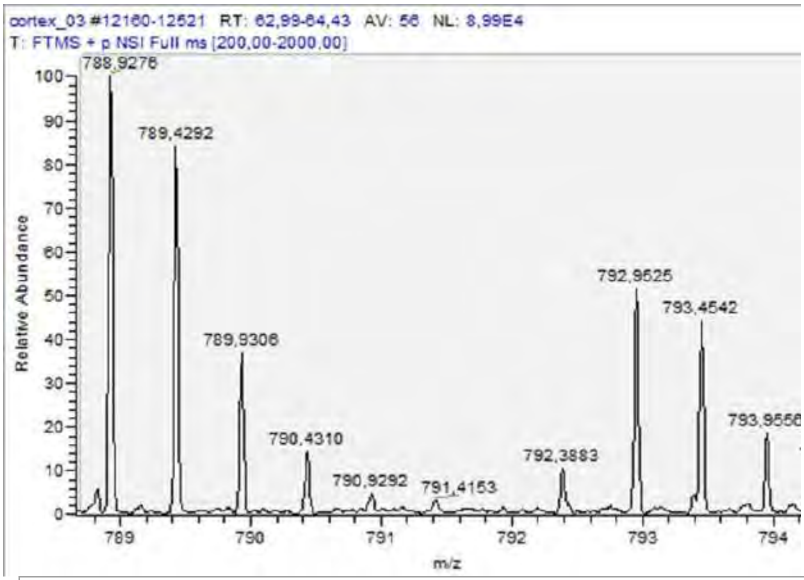

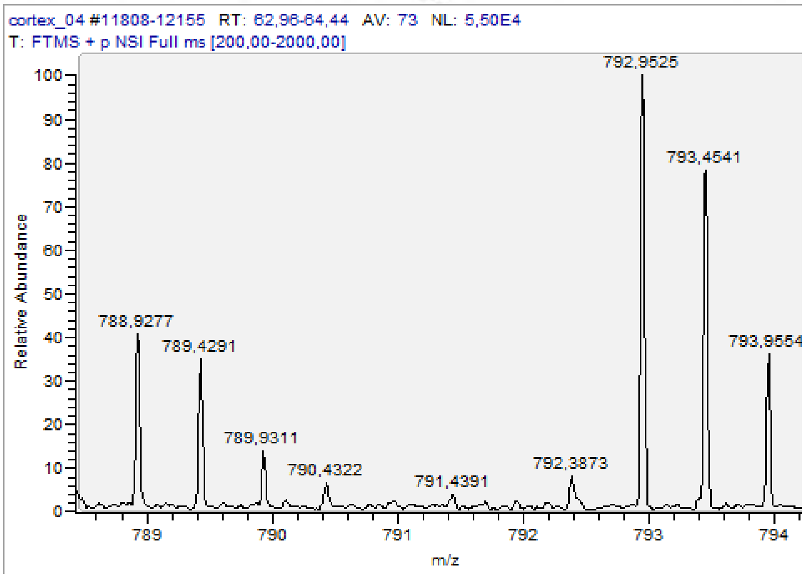

SKGLGSDLDSSLASL 2+

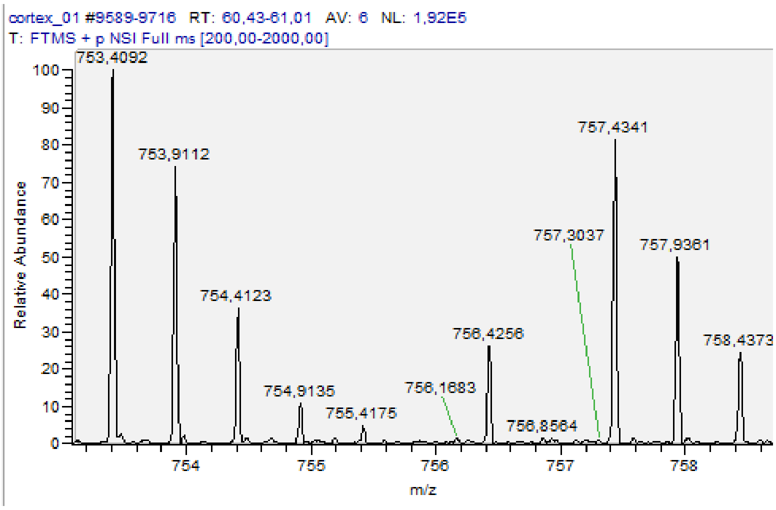

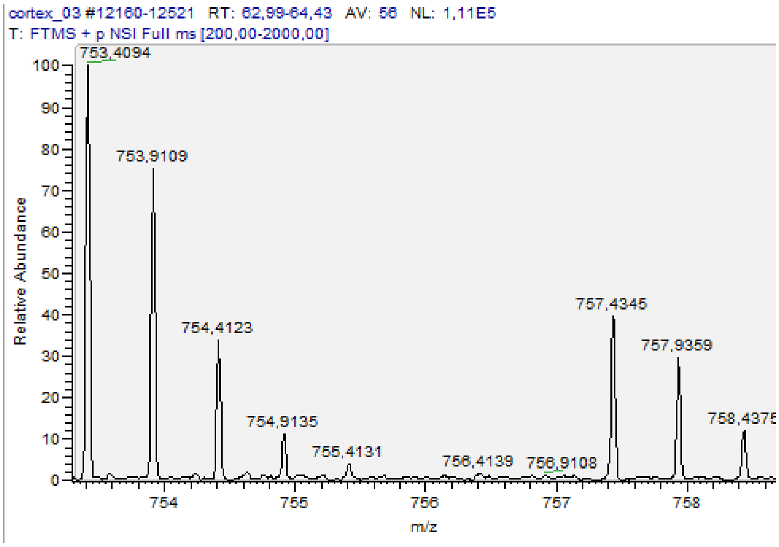

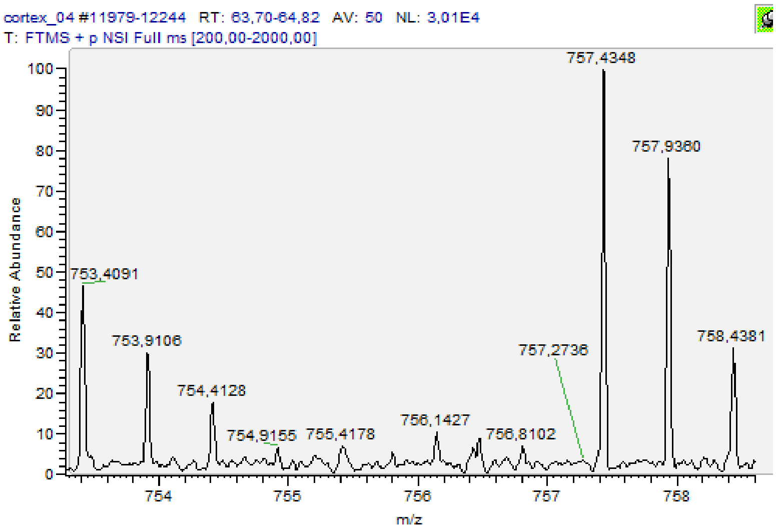

SPSPTPATQSPKKPPAKDPLADLNIKDFL 4+

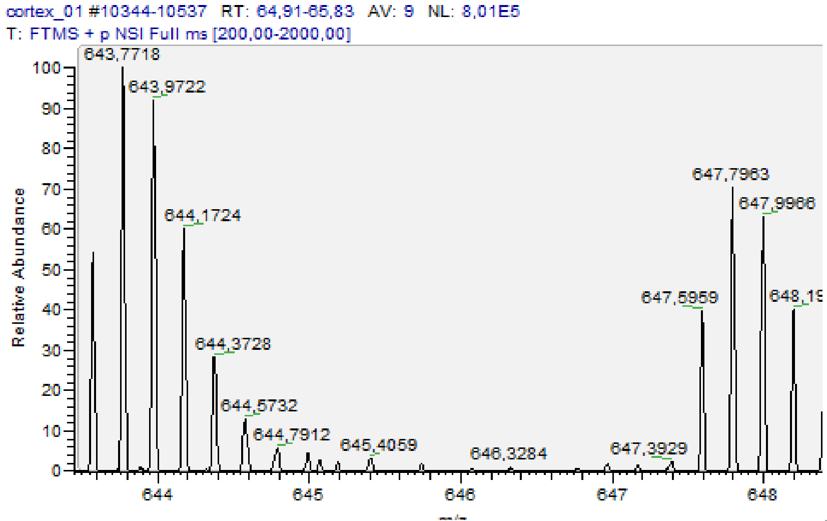

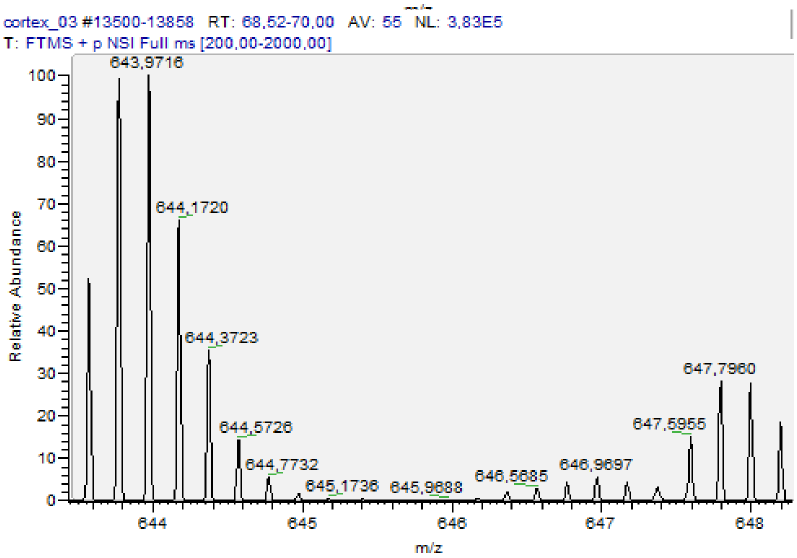

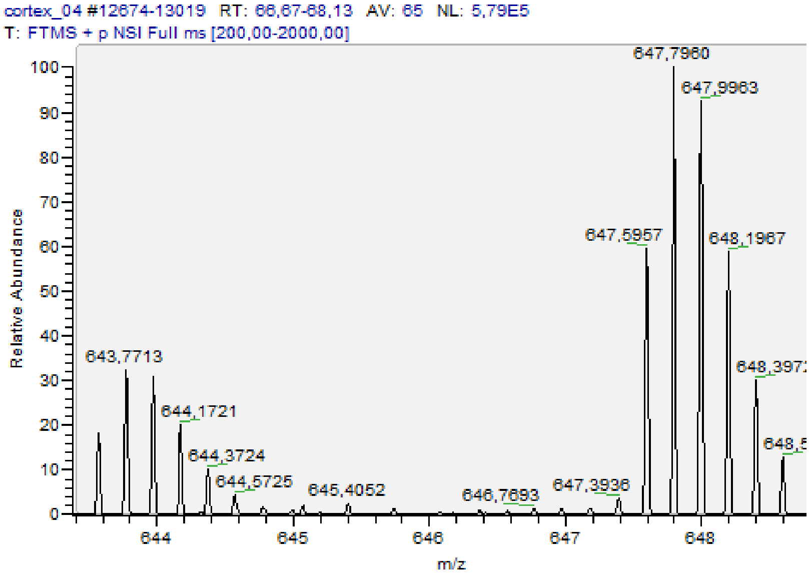

KGLGSDLDSSLASL 2+

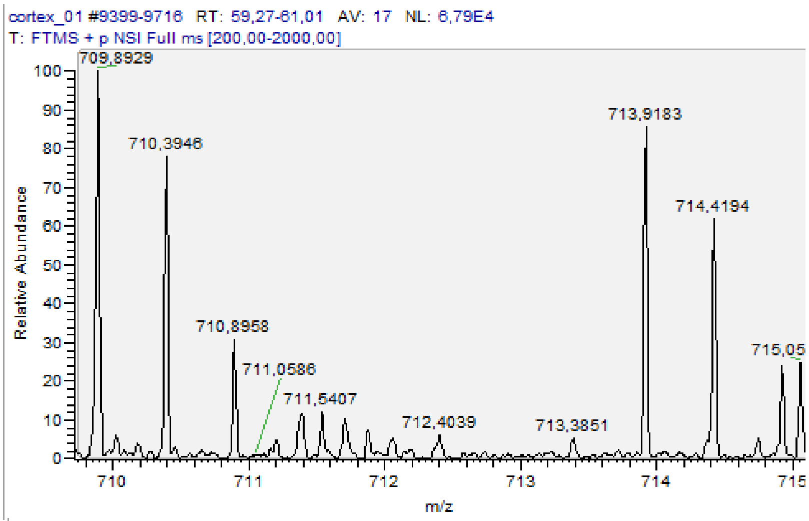

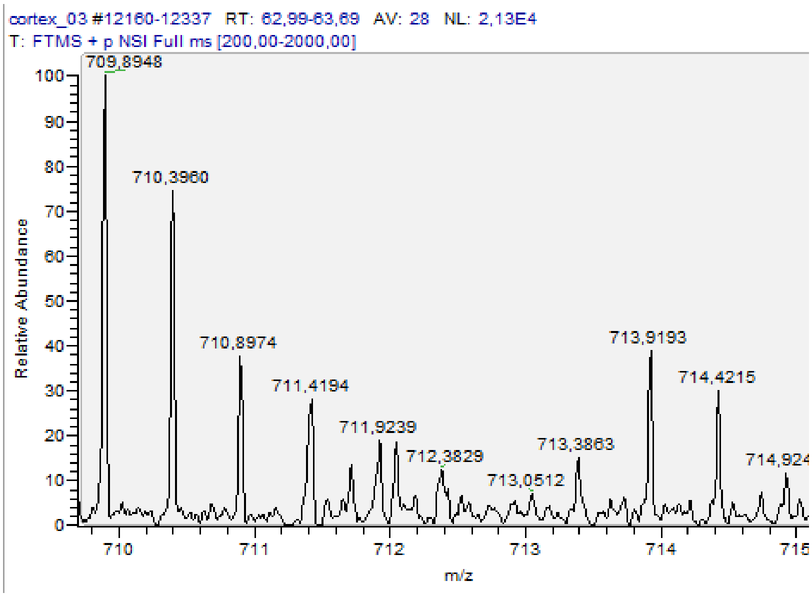

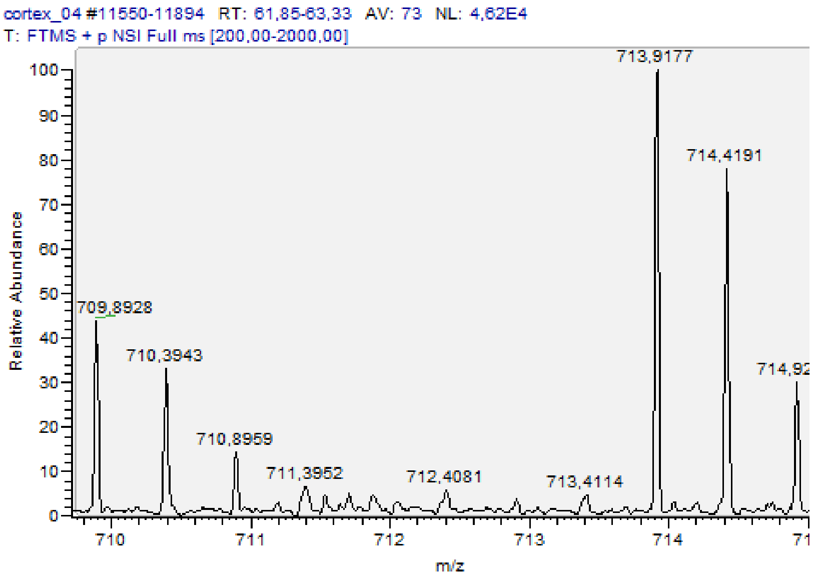

TQVVSDAAYKGVQPHVV 3+

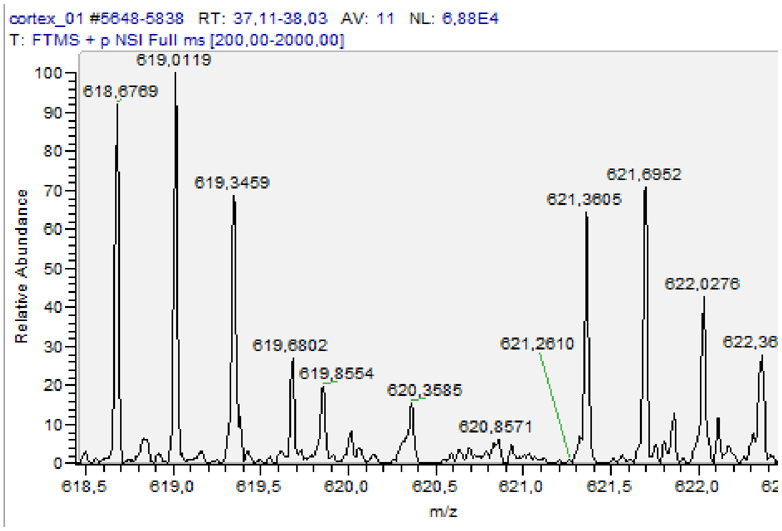

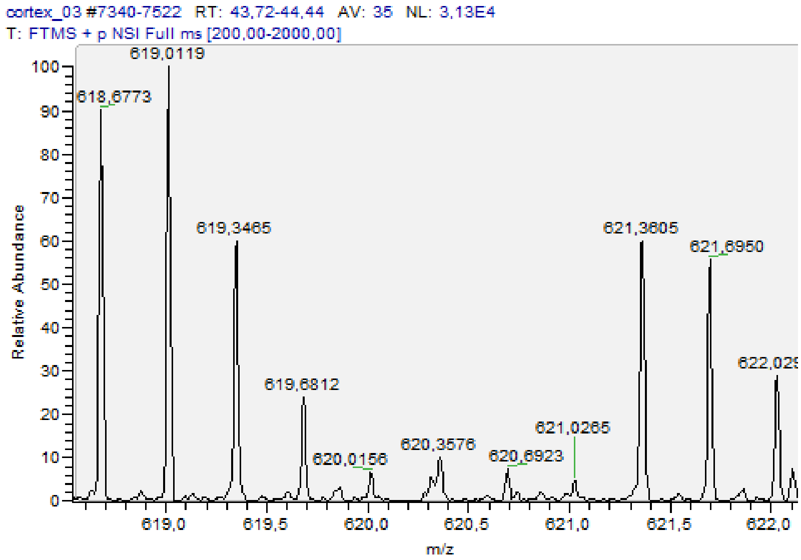

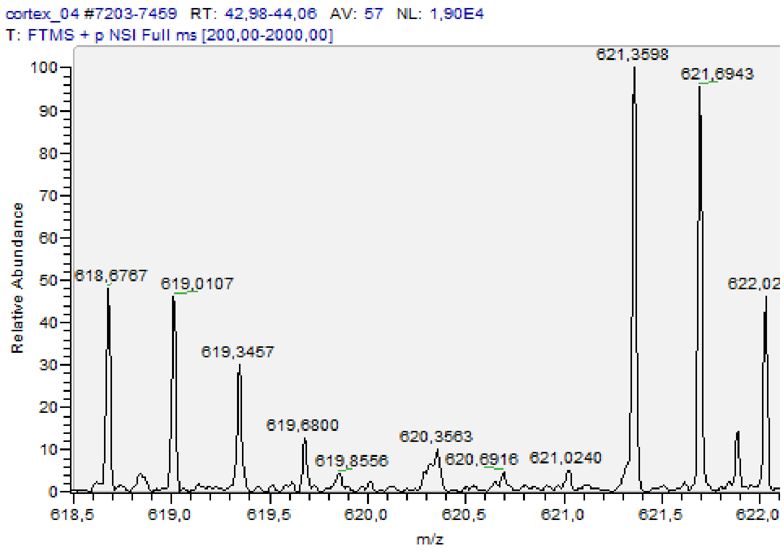

GRKGPGPGGPGGAGGARGGAGGGPSGD 3+

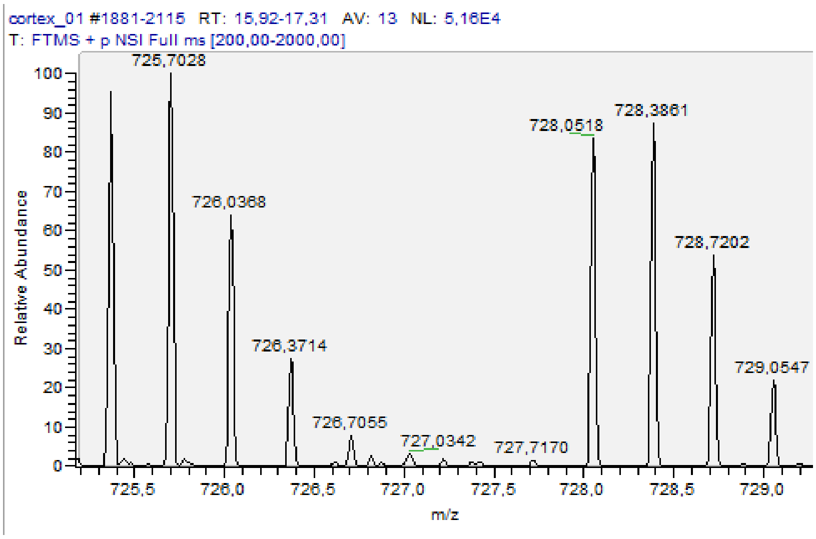

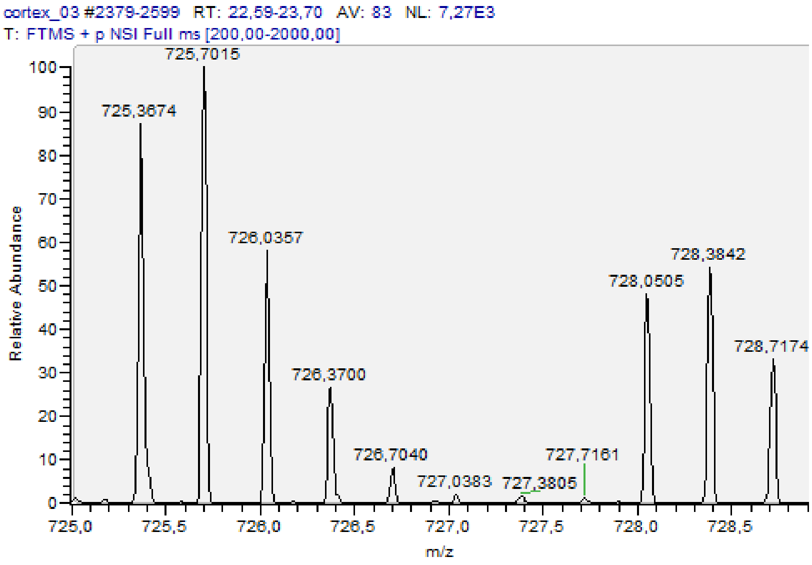

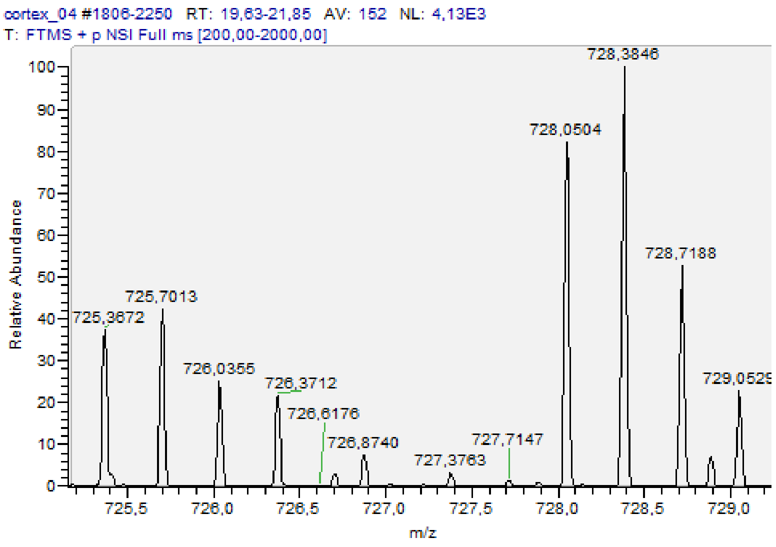

AQATGKPAQYIAVHVVPDQL 3+

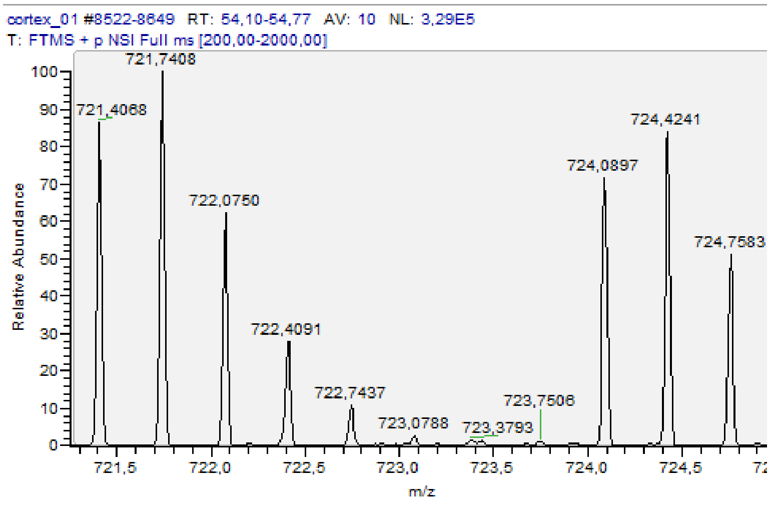

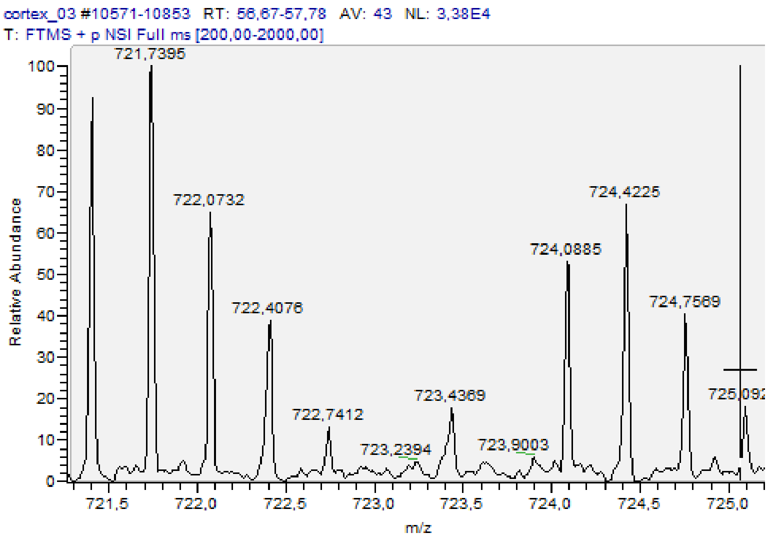

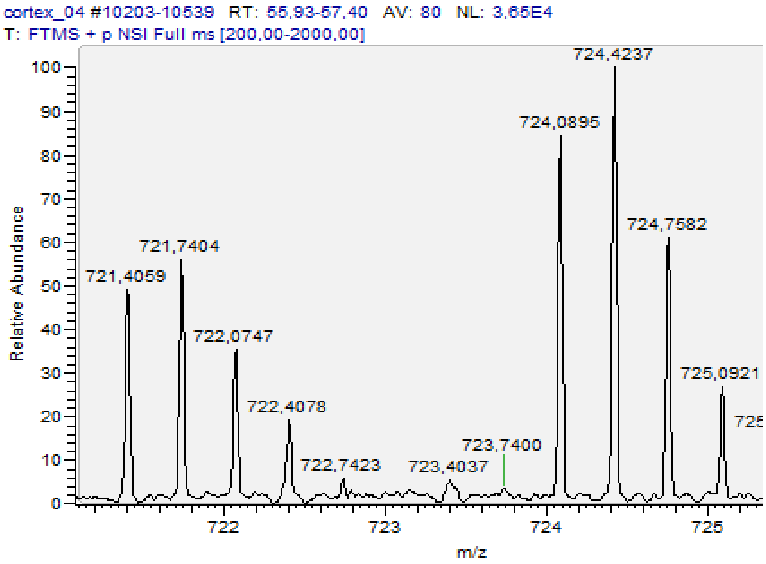

AQATGKPAQYIAVHVVPDQL 2+

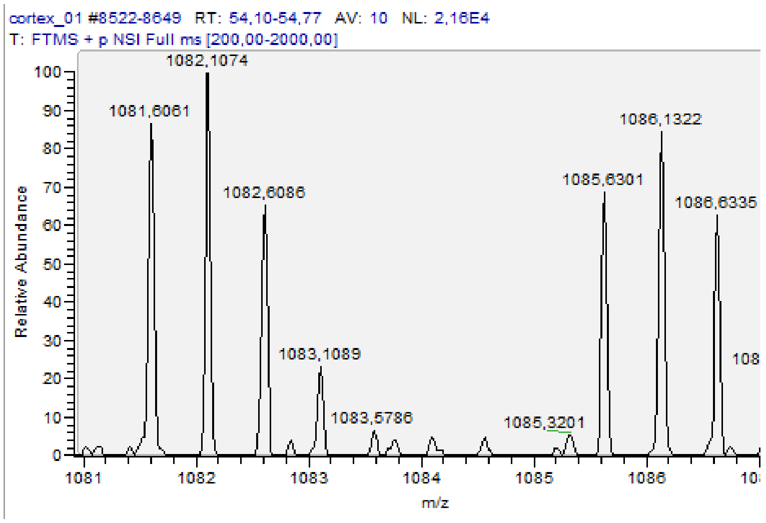

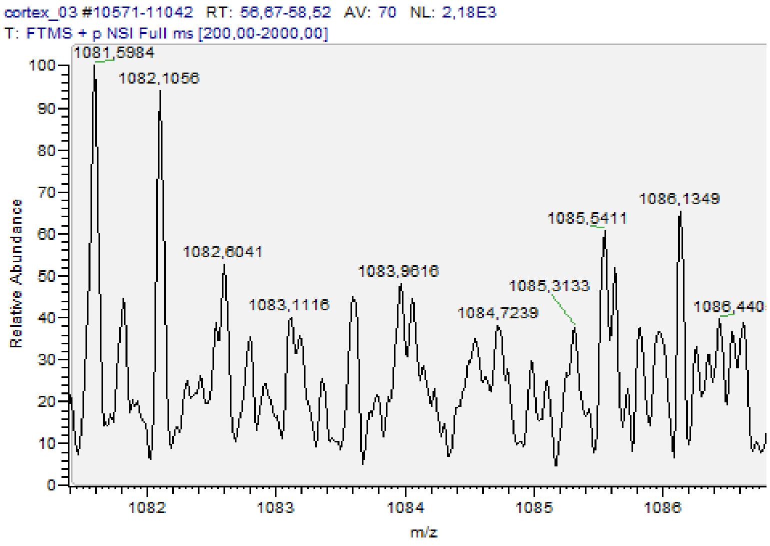

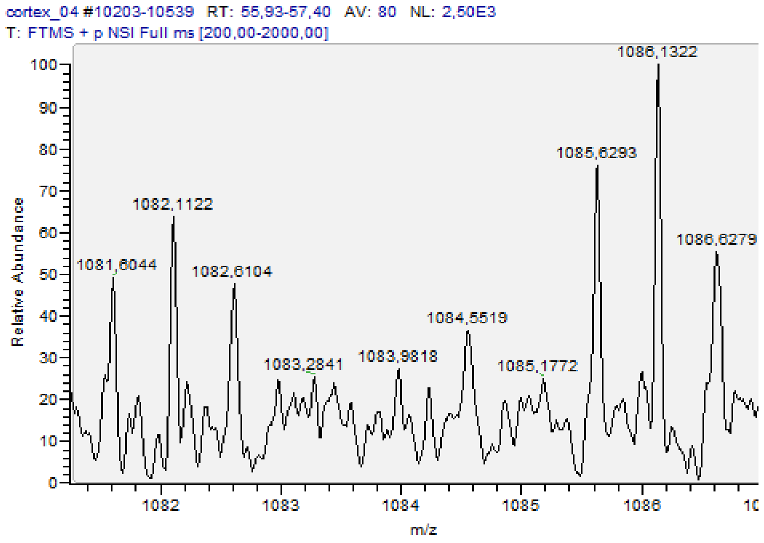

AQATGKPAQYIA 2+

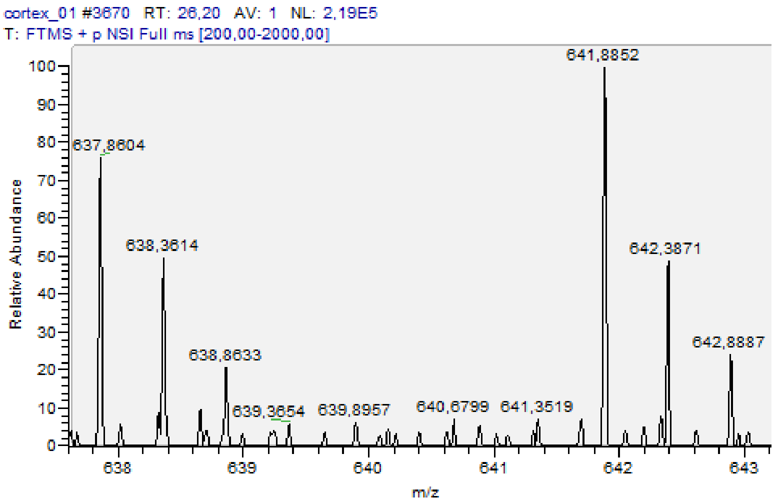

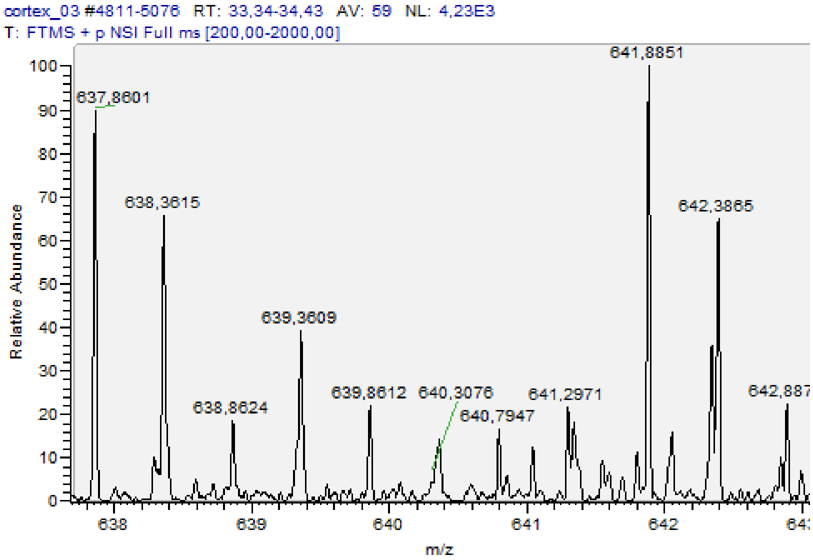

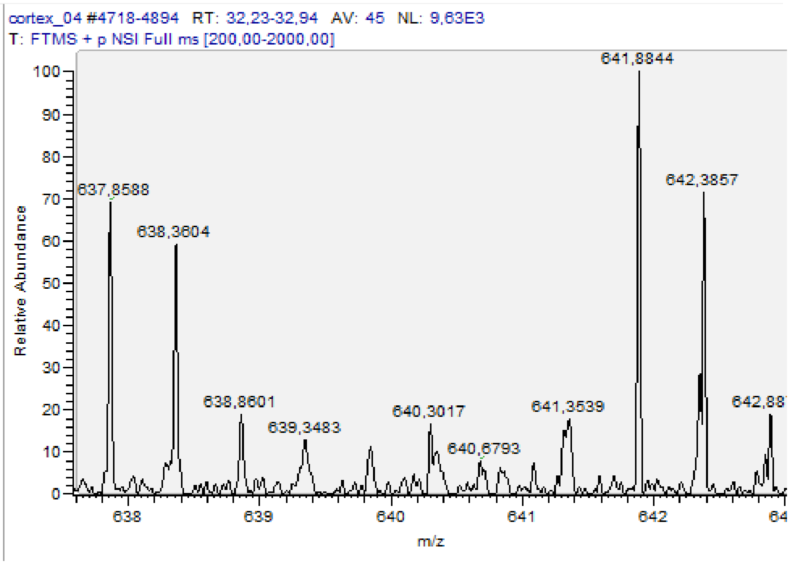

EDENFILKHTGPGILSM 2+

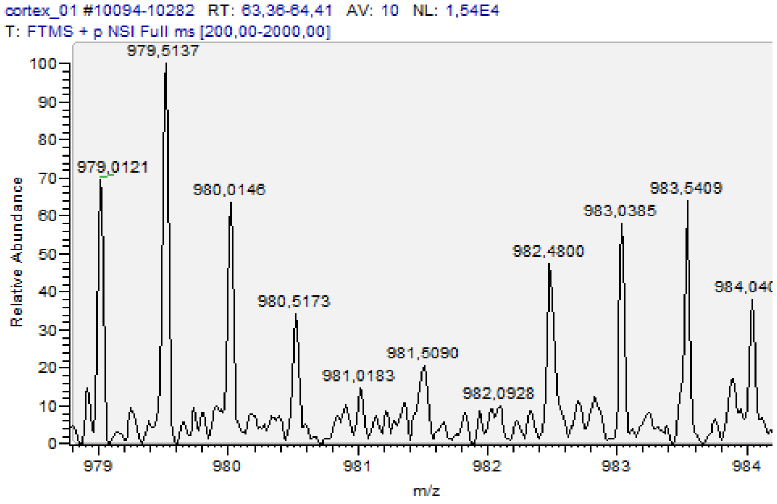

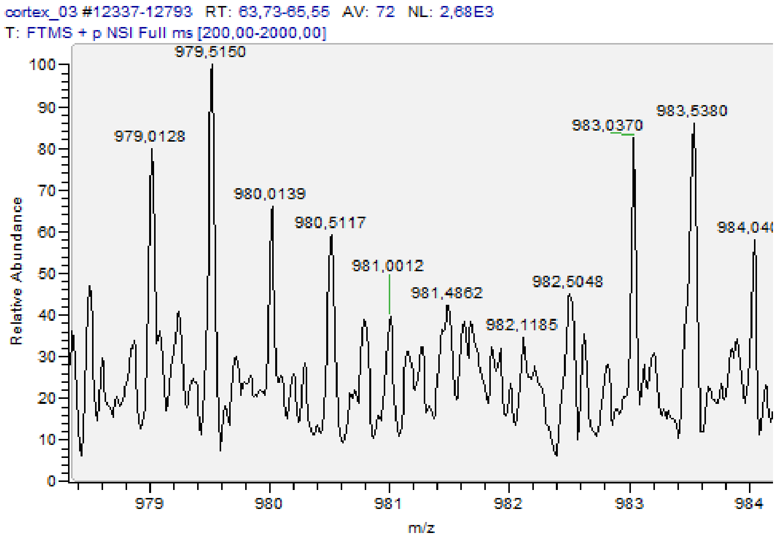

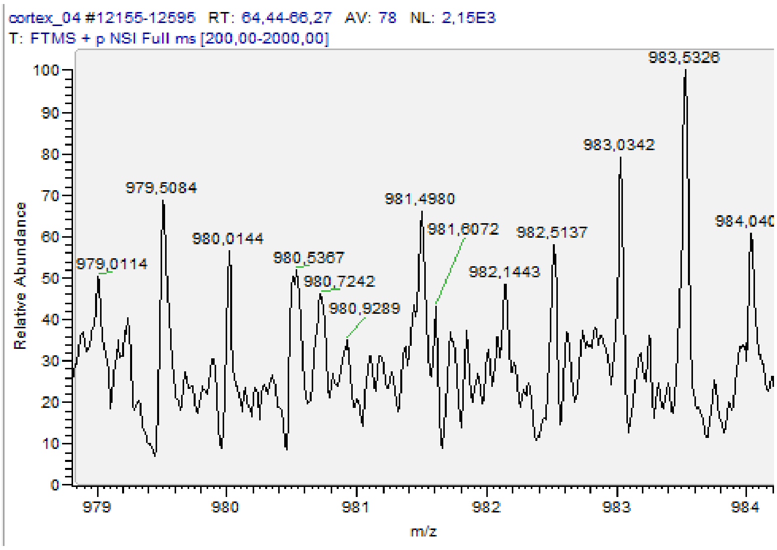

ELFADKVPKTAENFRAL 3+

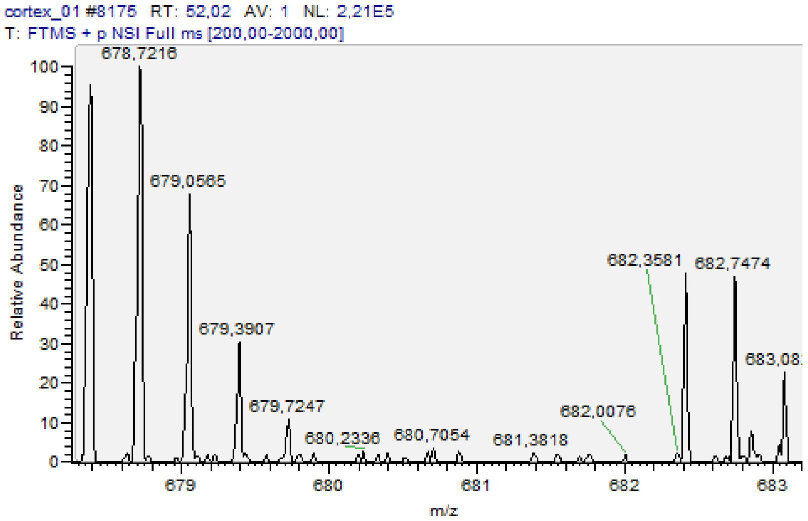

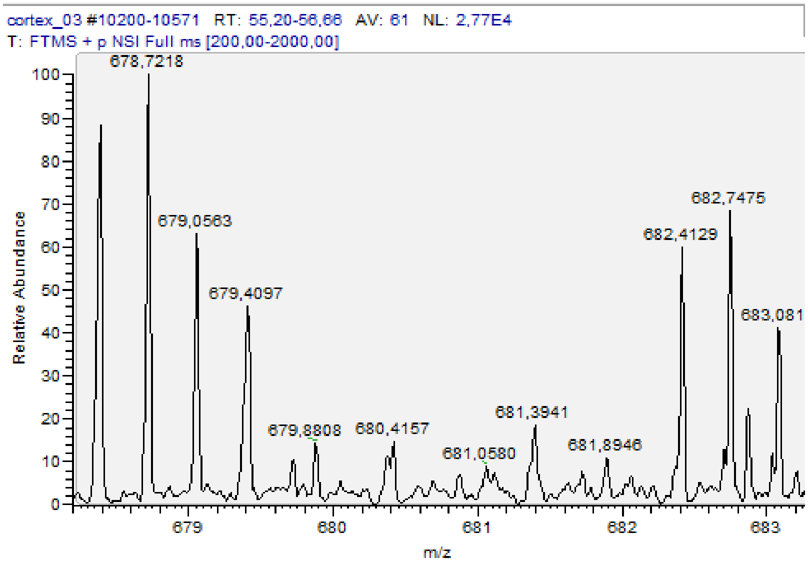

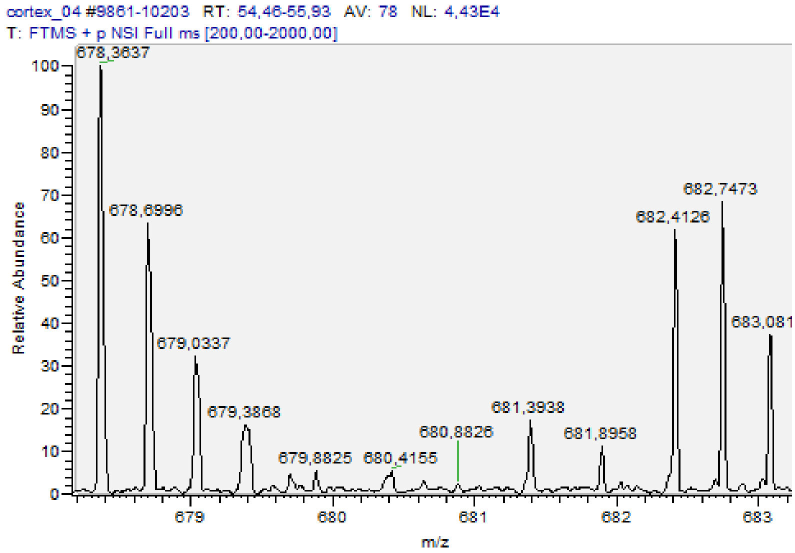

LFADKVPKTAENFRAL 3+

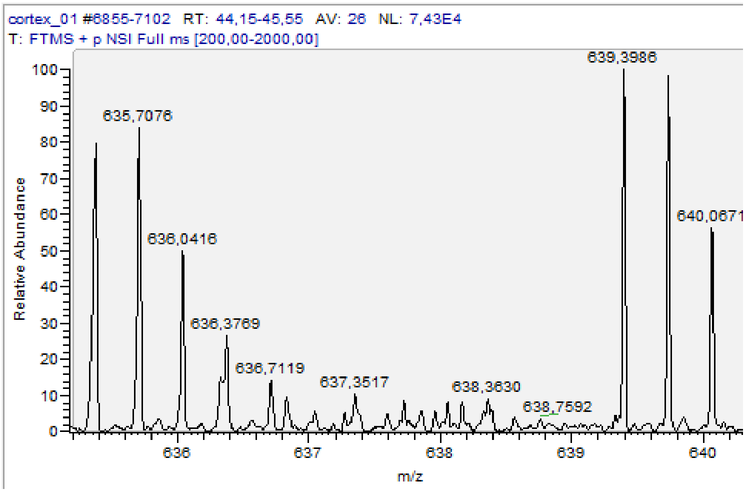

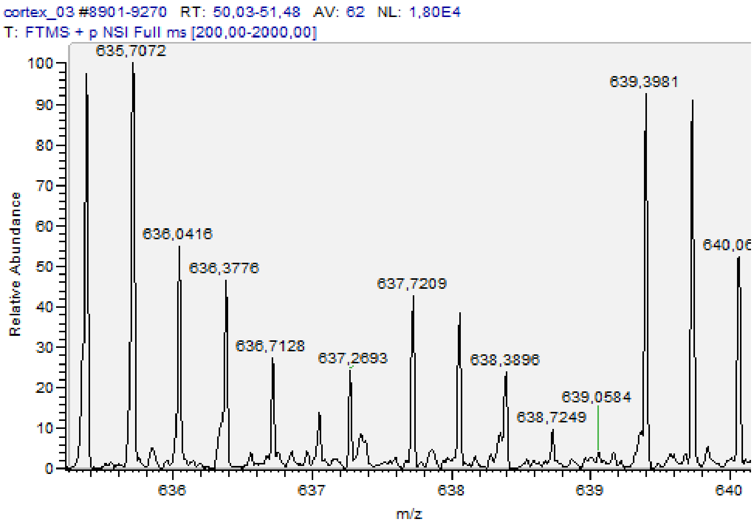

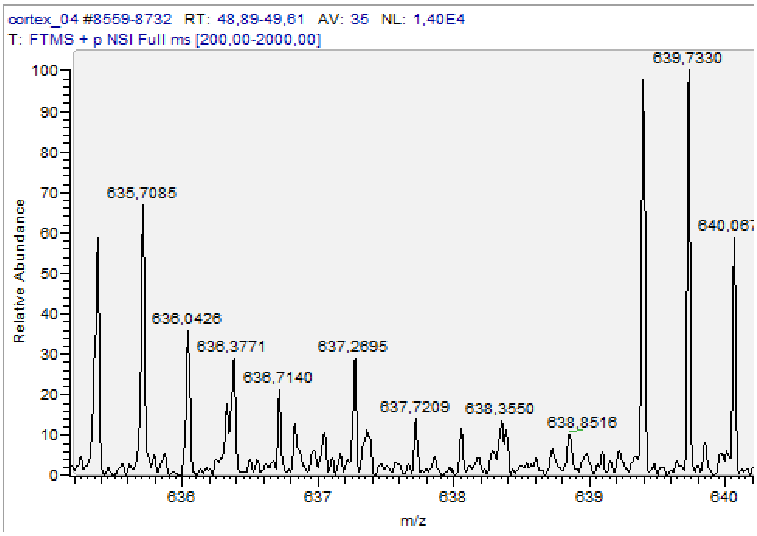

ADKVPKTAENFRAL 3+

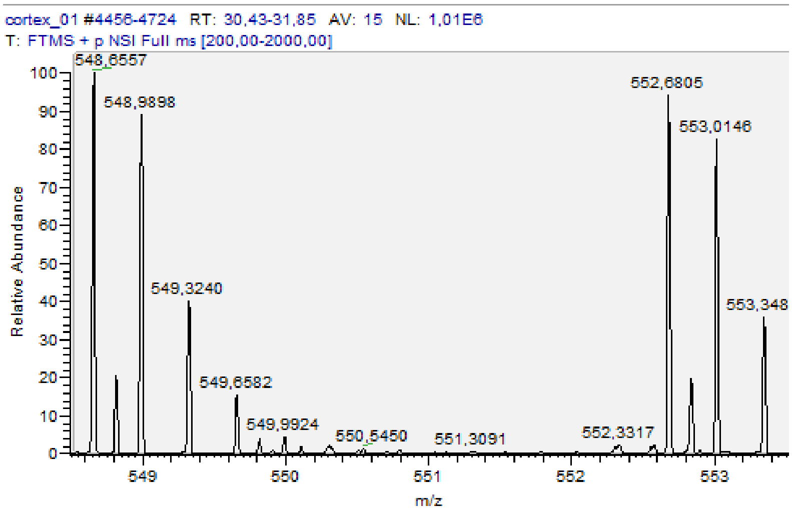

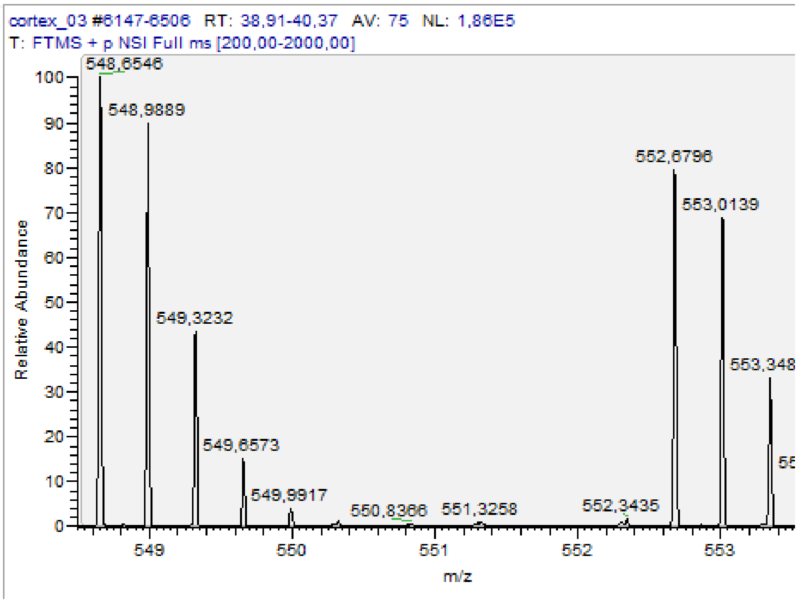

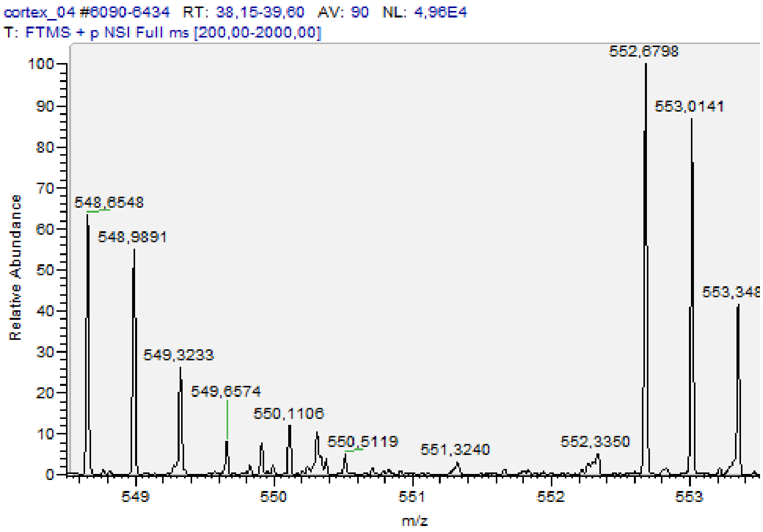

ITADDEPLGRVSF 2+

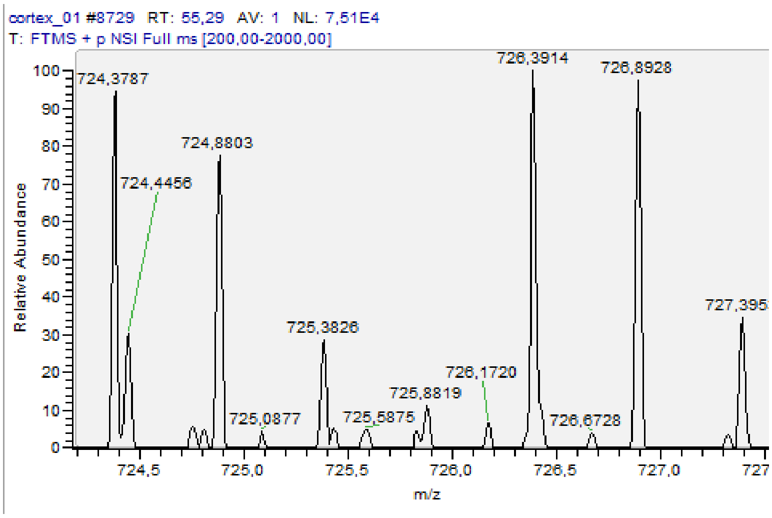

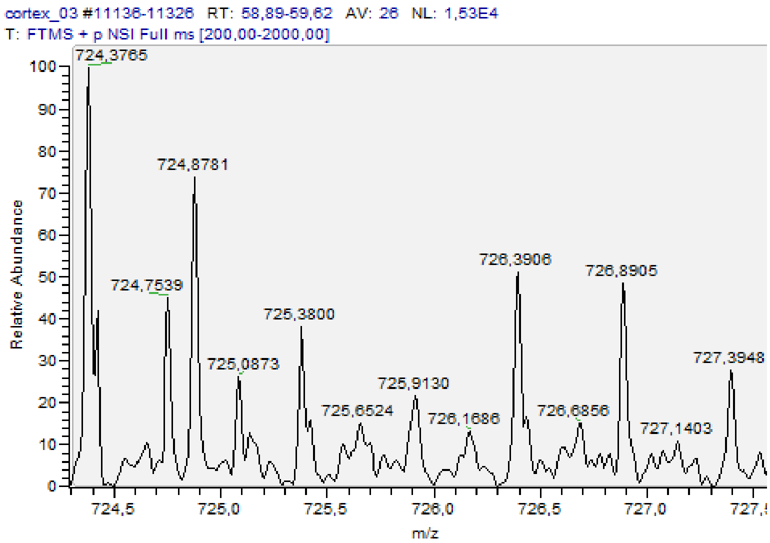

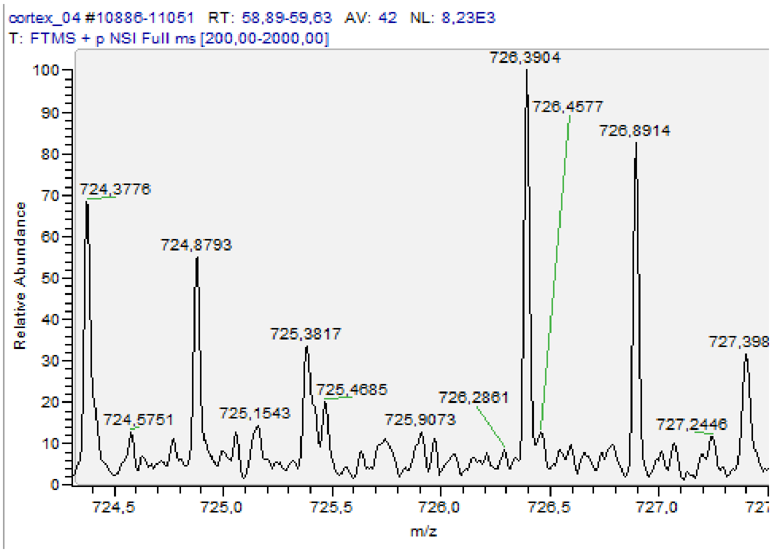

ADKVPKTAENF 2+

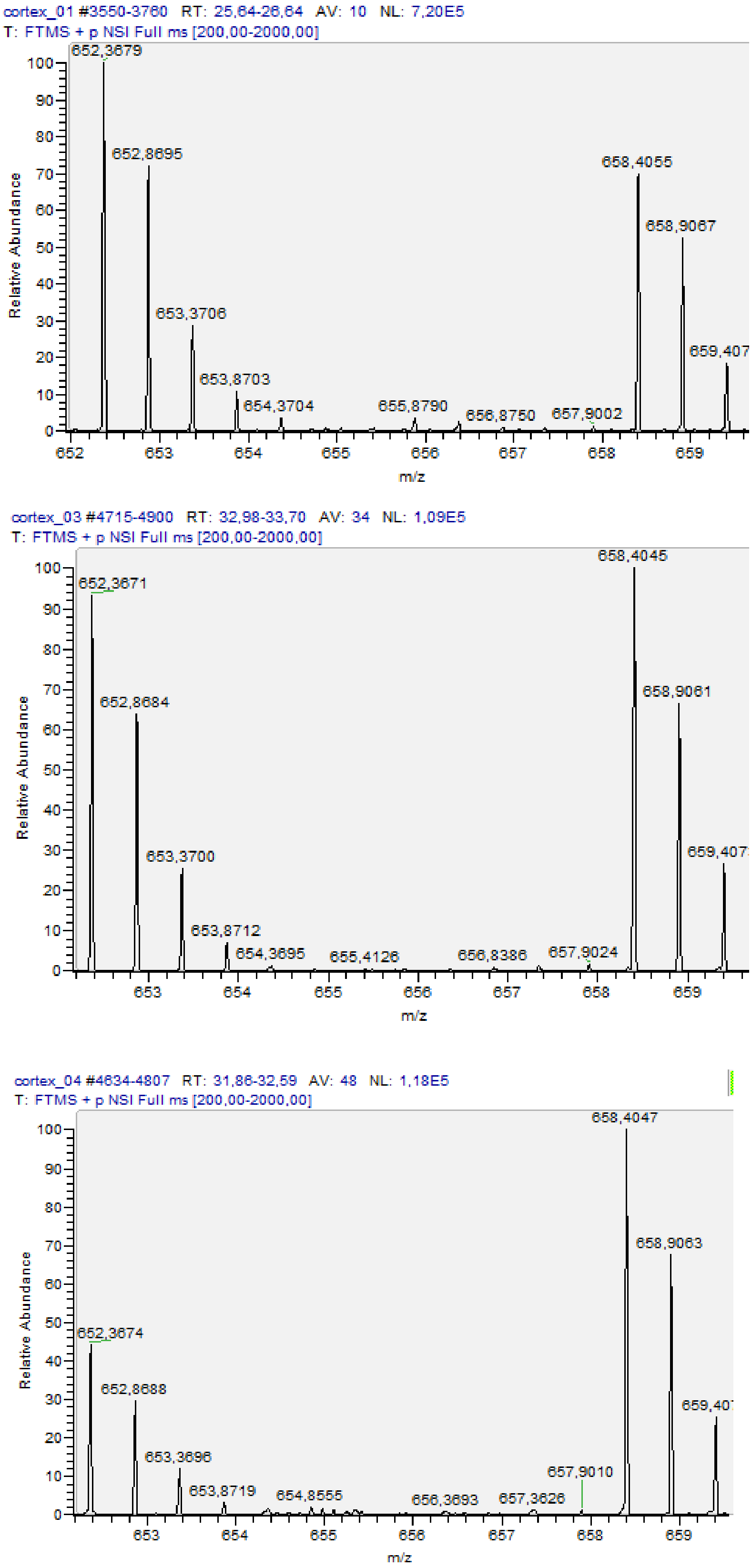

ADDEPLGRVSF 2+

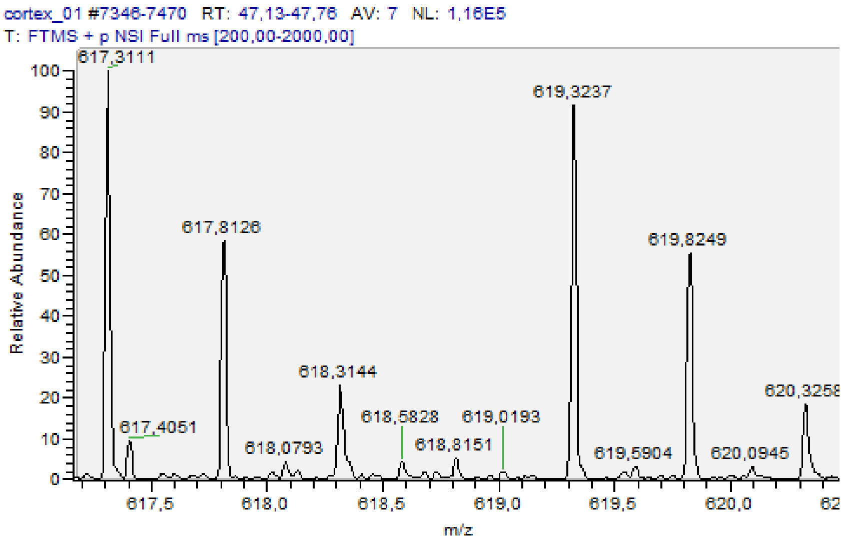

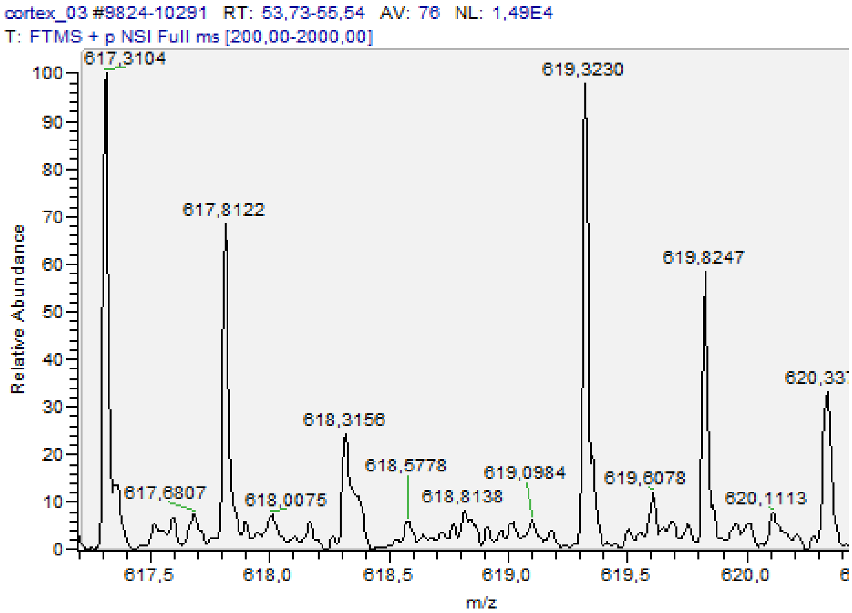

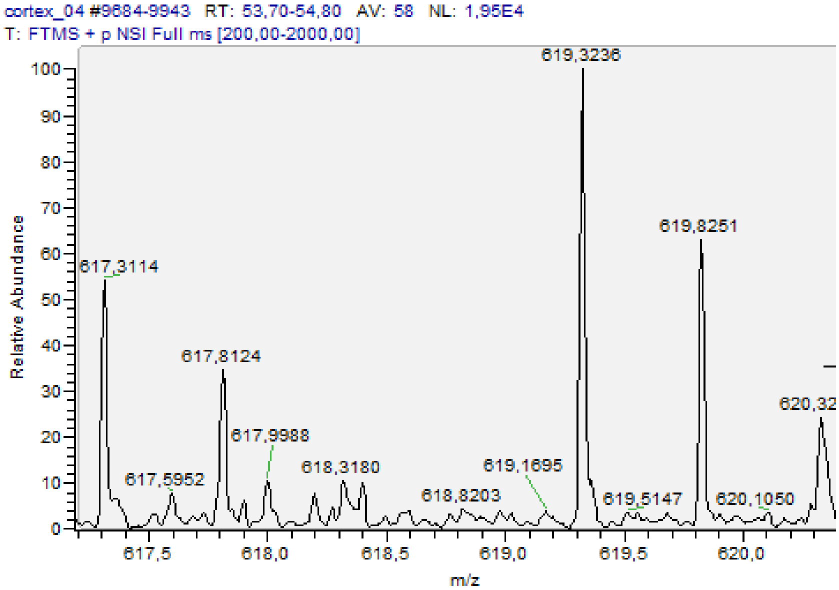

SGQAAARPLVA 2+

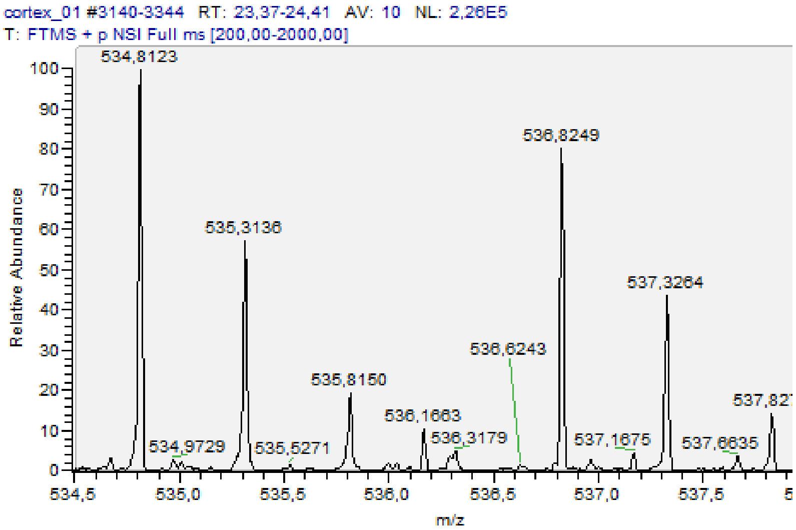

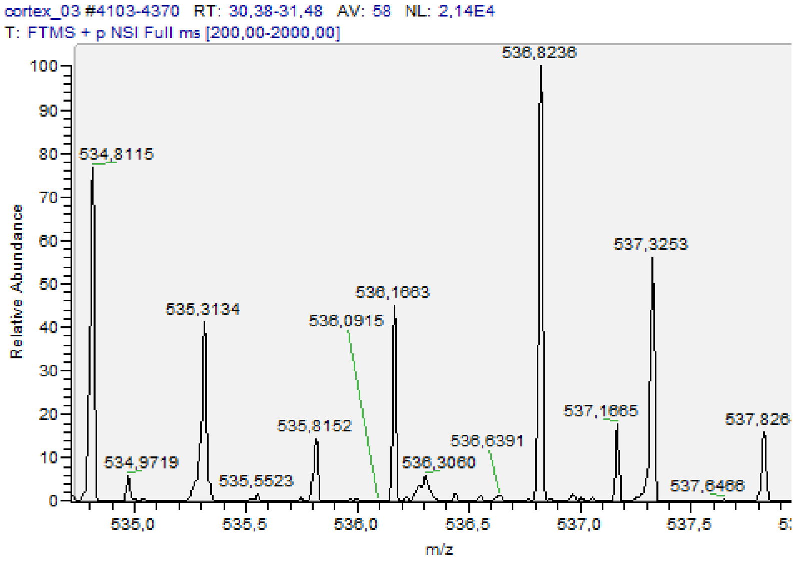

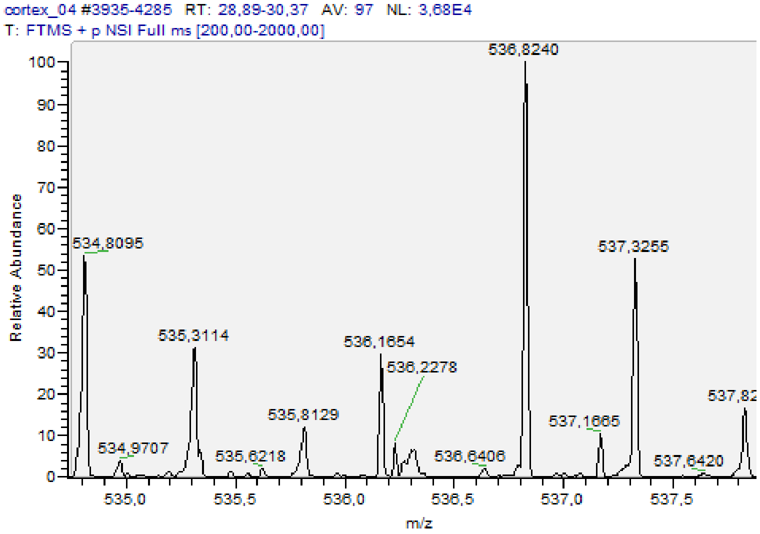

KGPGPGGPGGAGGARGGAGGGPSGD 2+

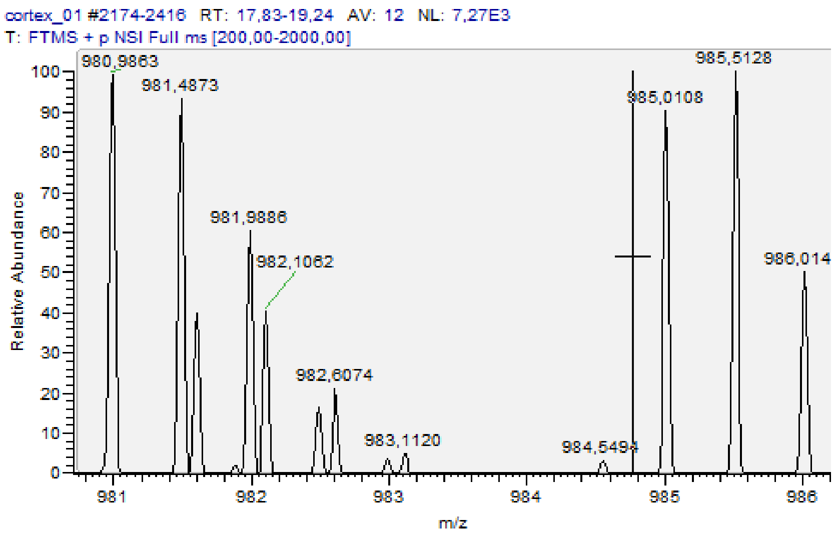

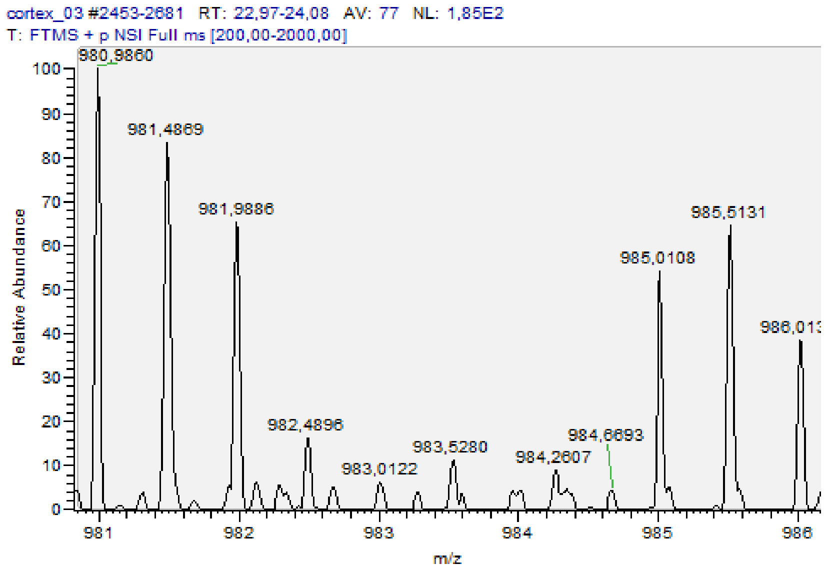

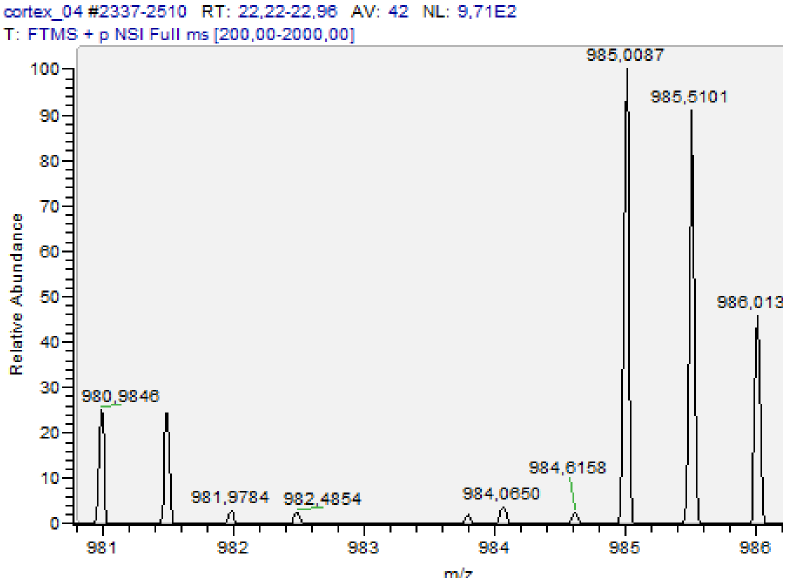

RKGPGPGGPGGAGGARGGAGGGPSGD 3+

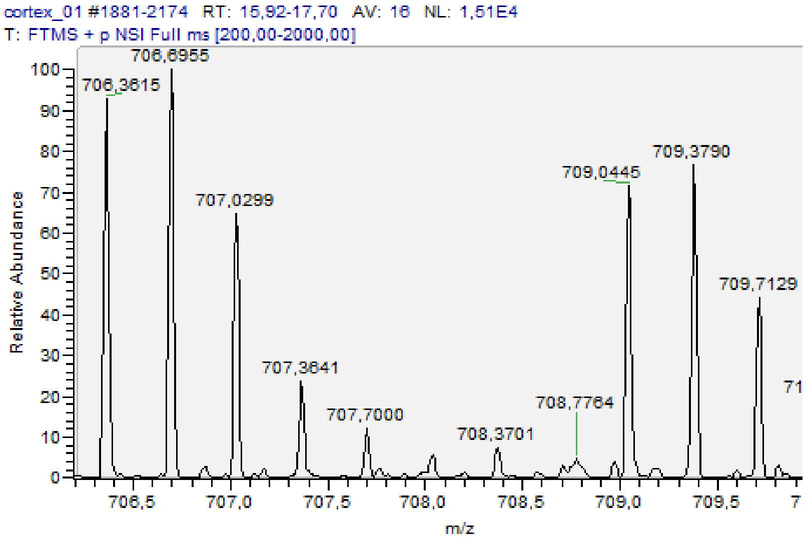

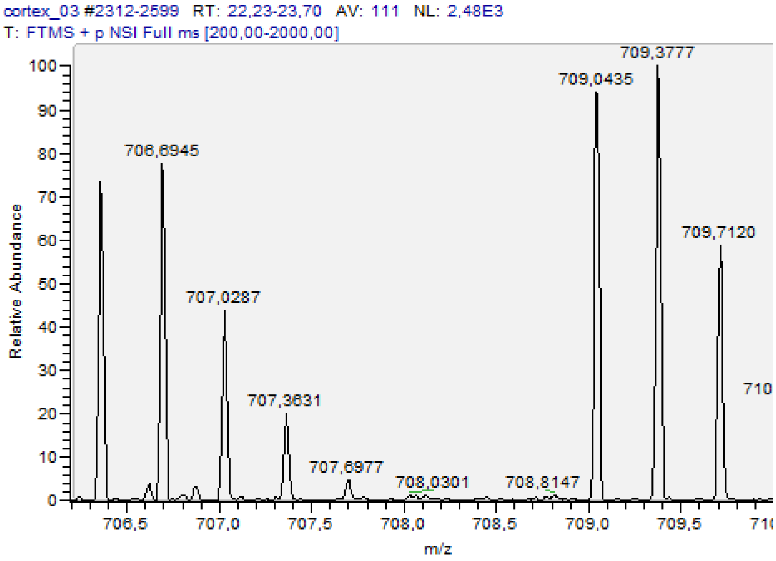

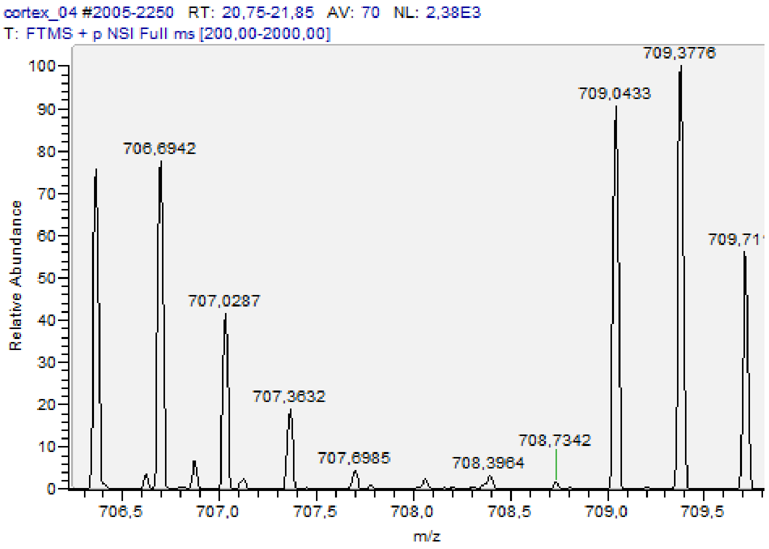

KQATVGDVNTDRPGLLDL 3+

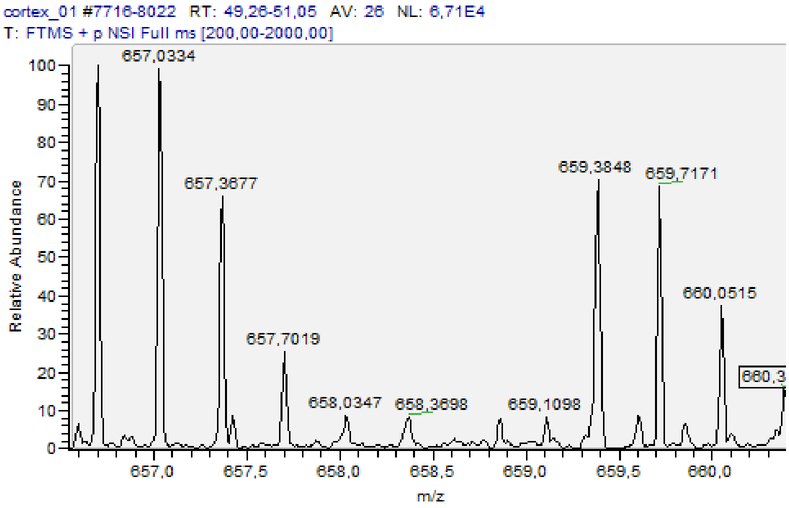

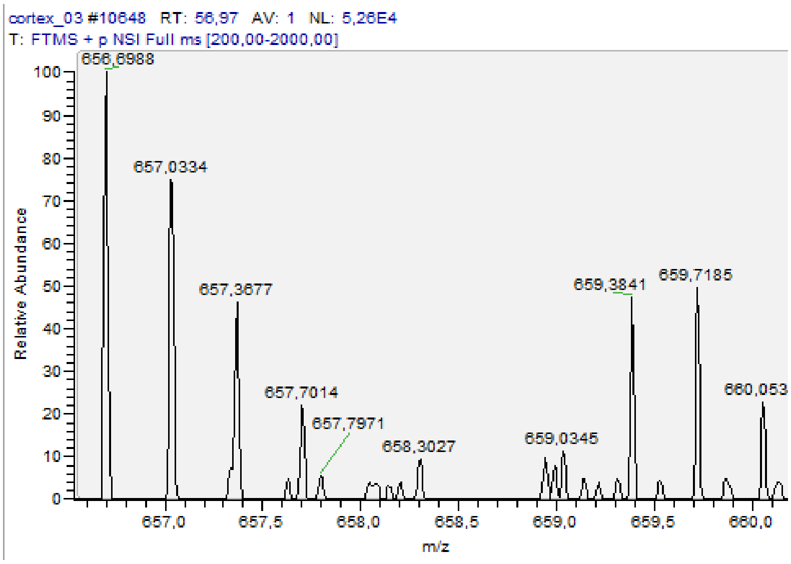

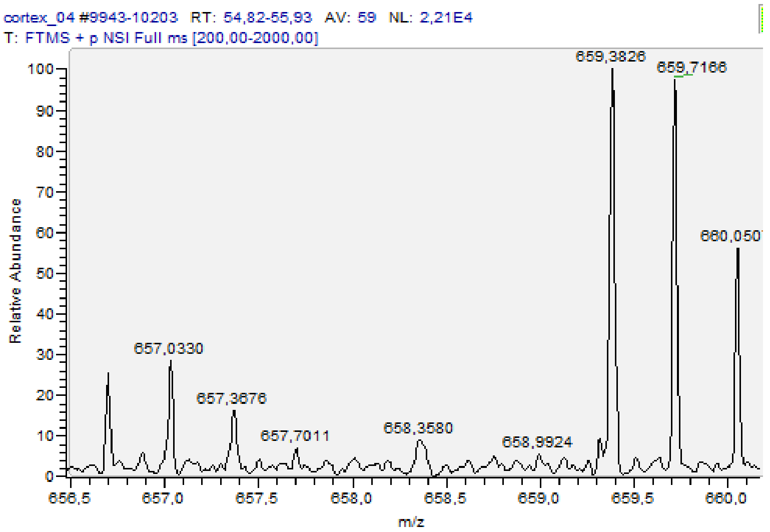

AANSKVAFSAVRSTN 2+

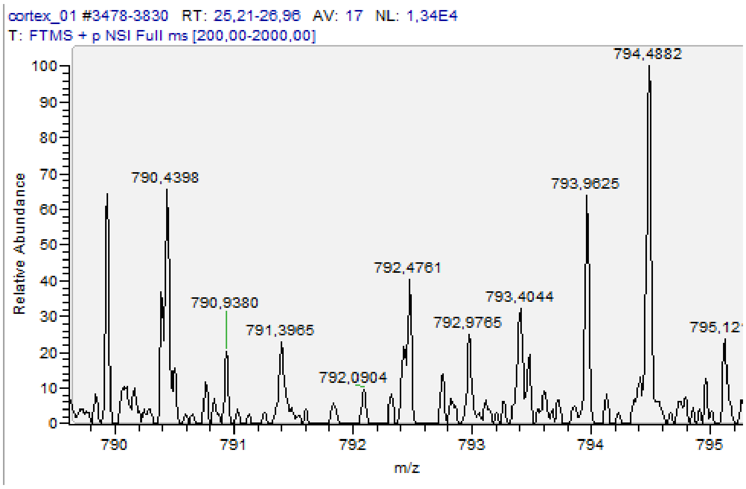

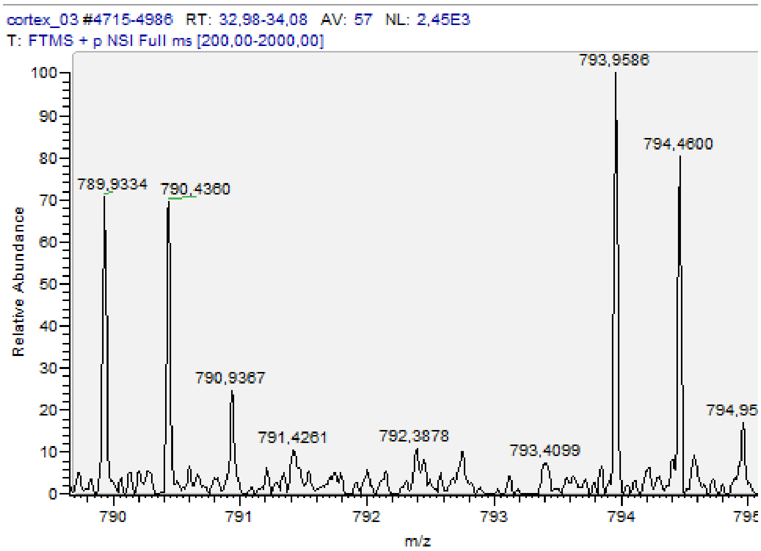

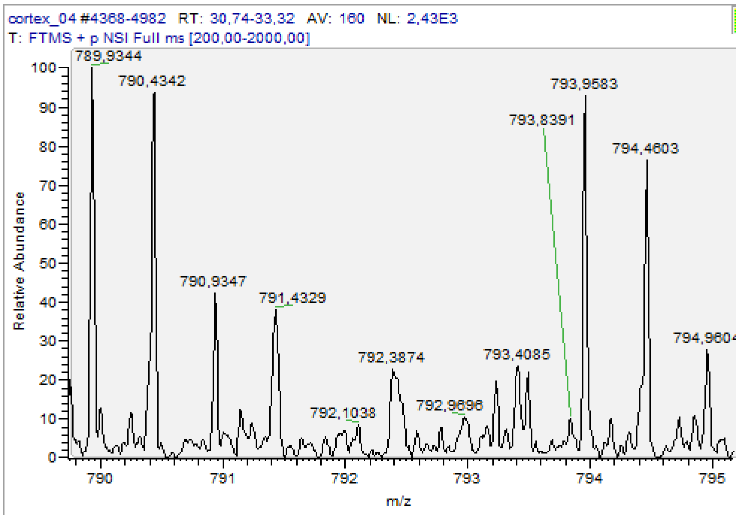

HNPHVNPLPTGYEDE 2+

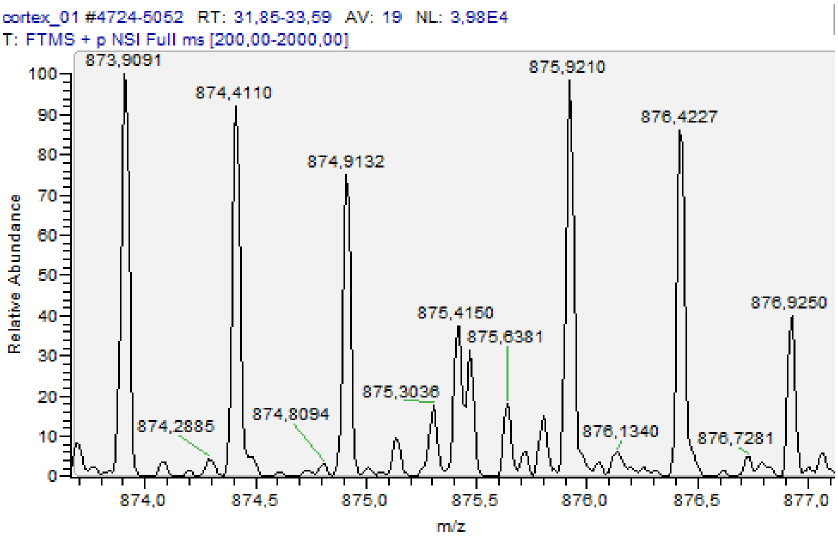

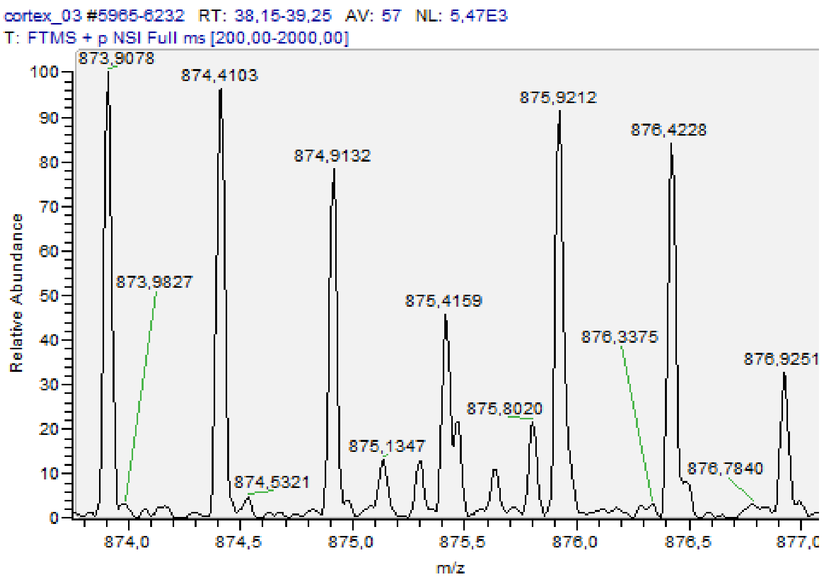

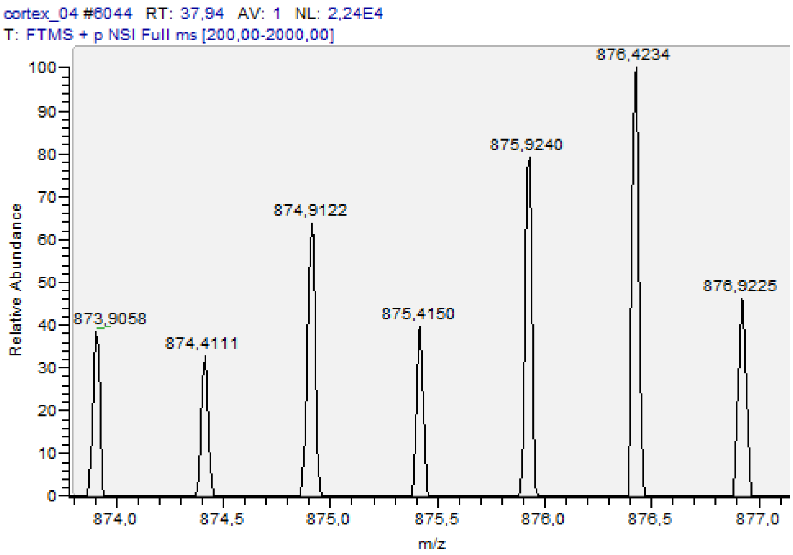

ADEVSASLAKQGL

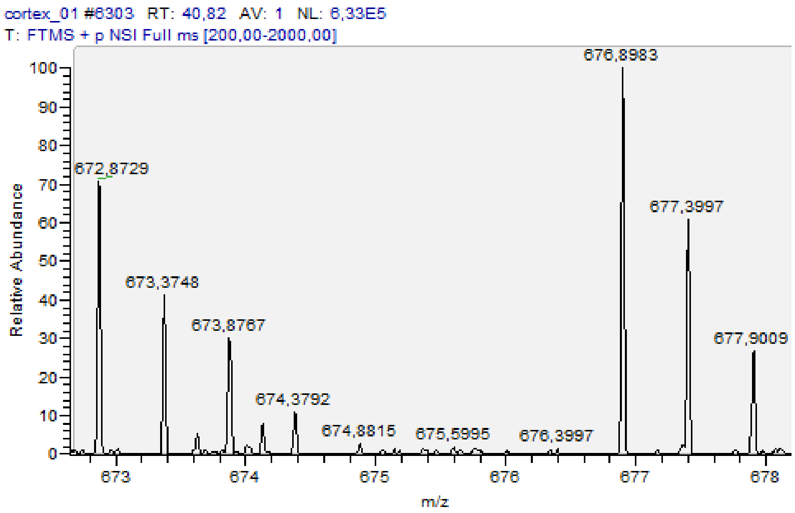

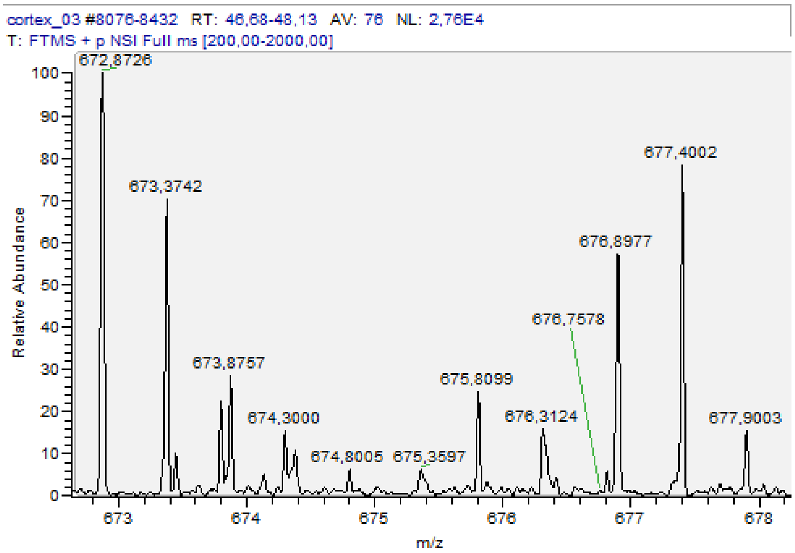

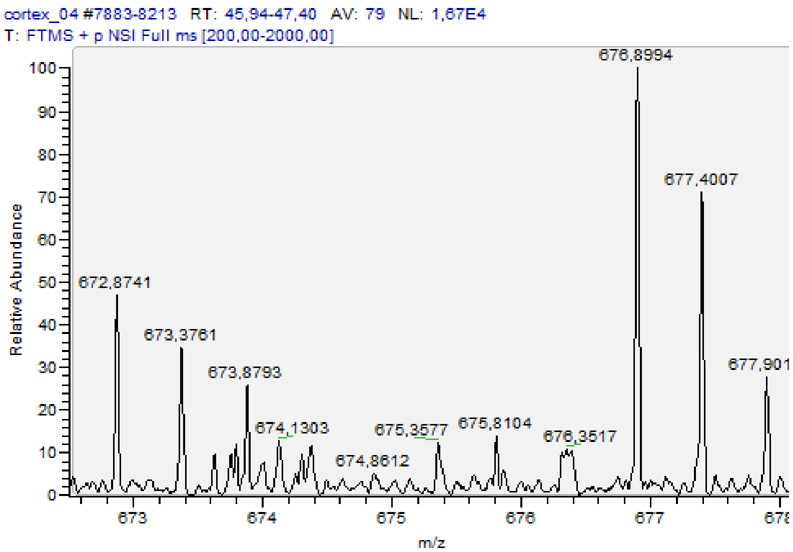

SANSNPAMAPRE 2+

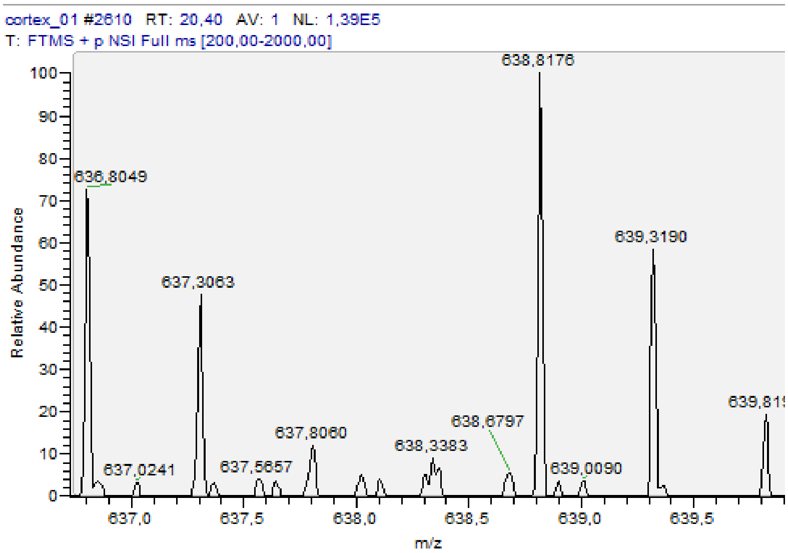

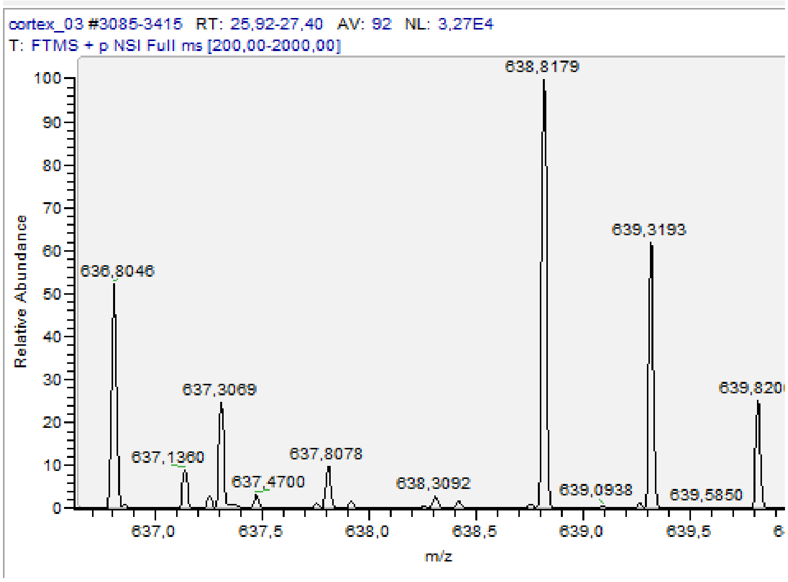

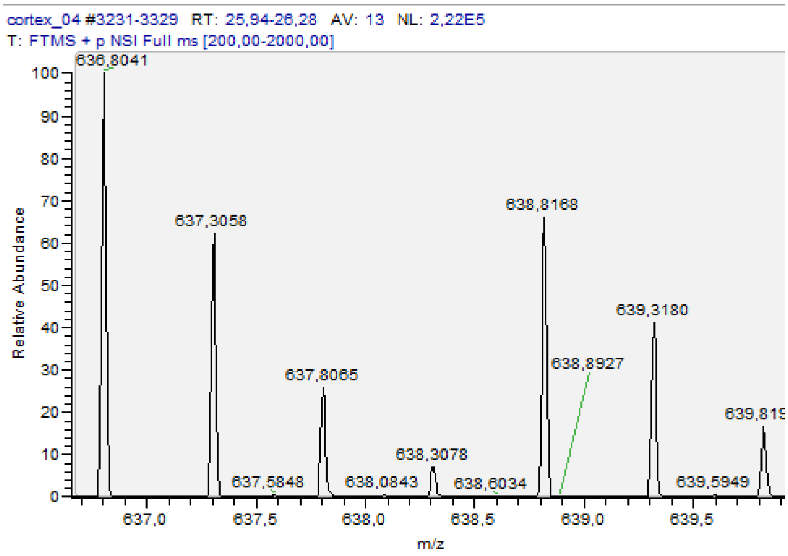

SANSNPAMAPRE 2+ (methionine oxidation)

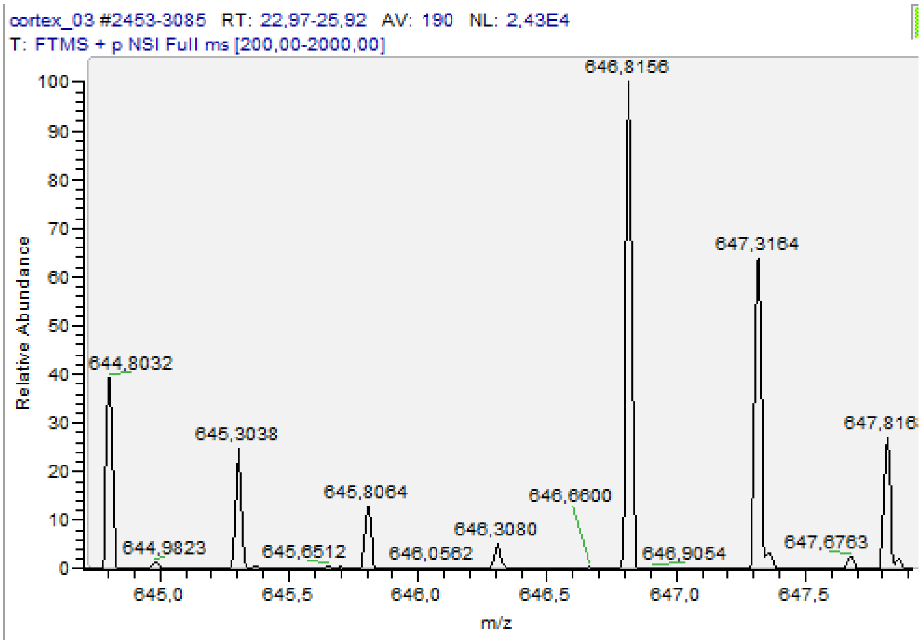

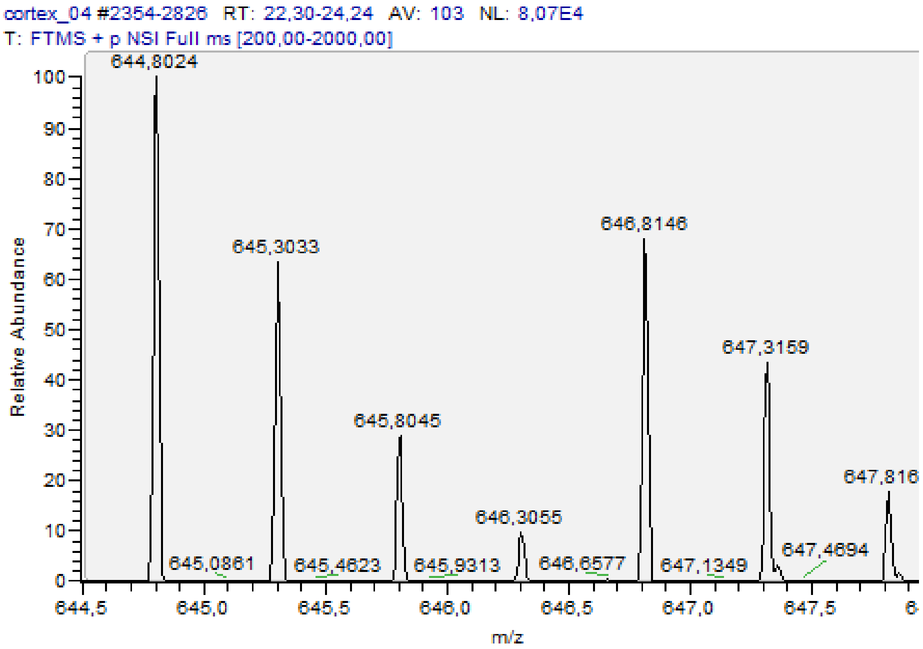

DDEESEAQGPK 2+

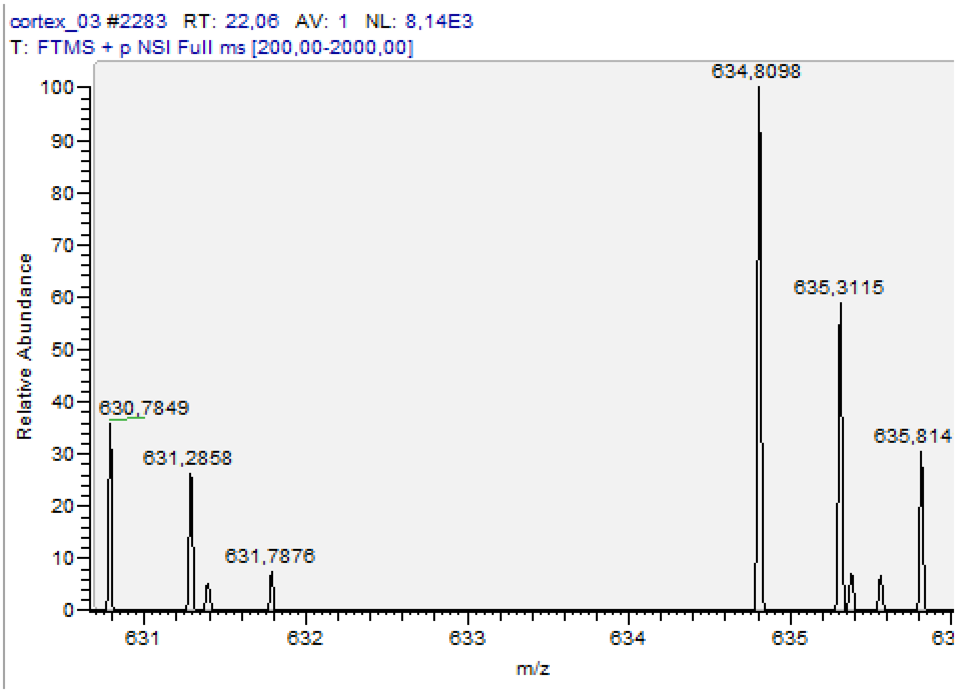

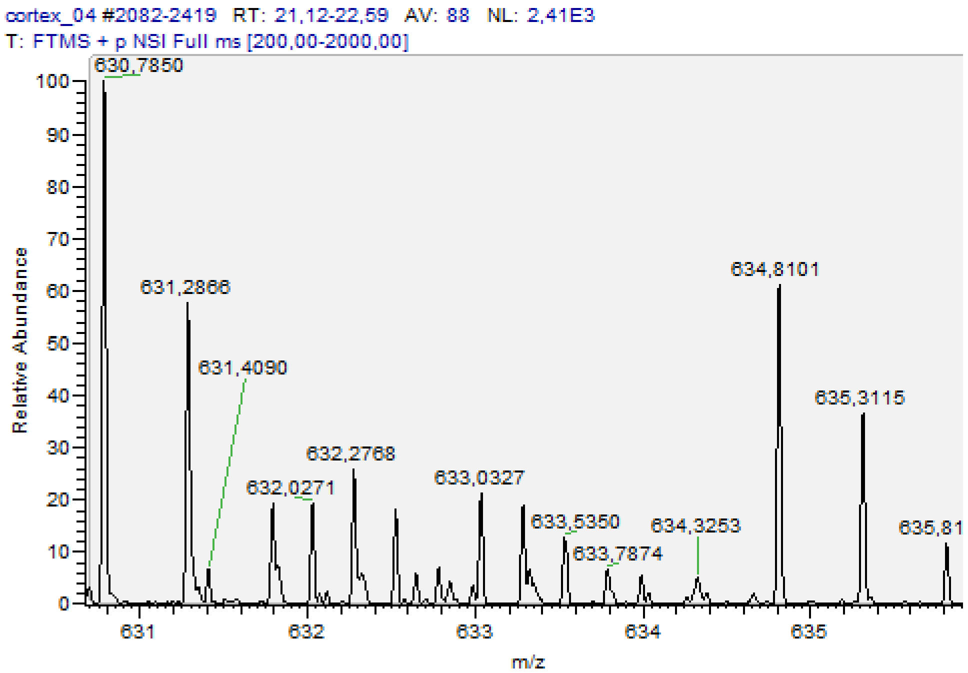

SLSAASAPLVETSTPLRL 2+

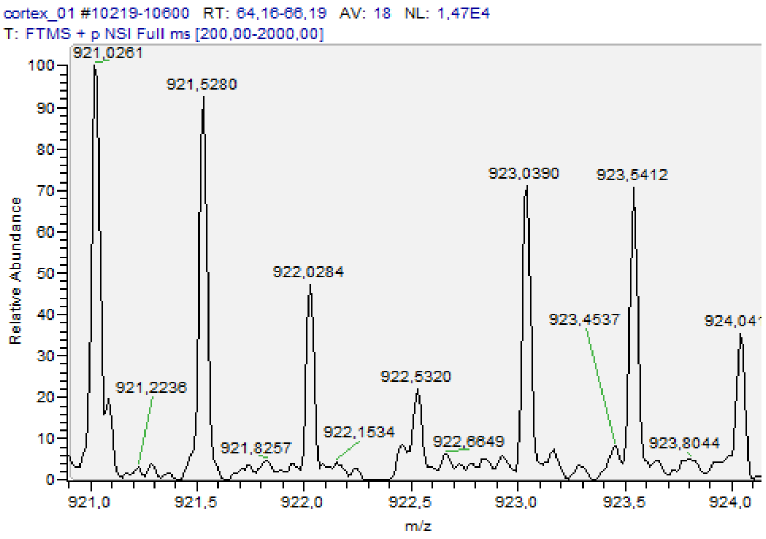

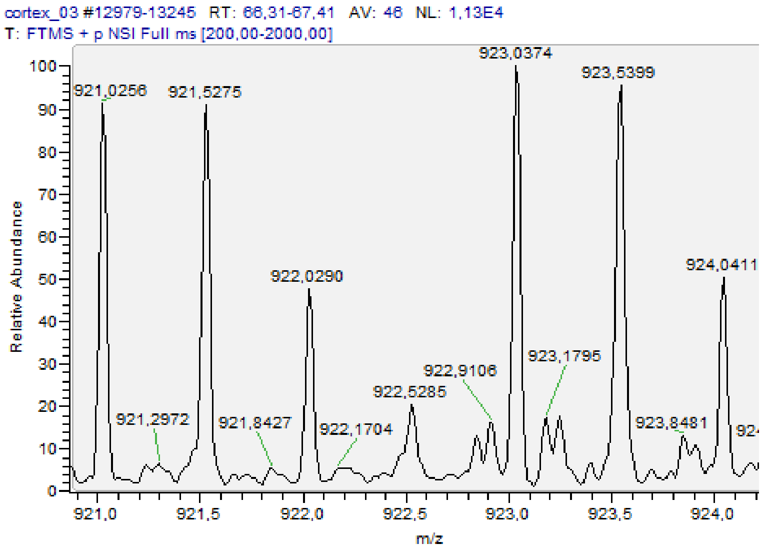

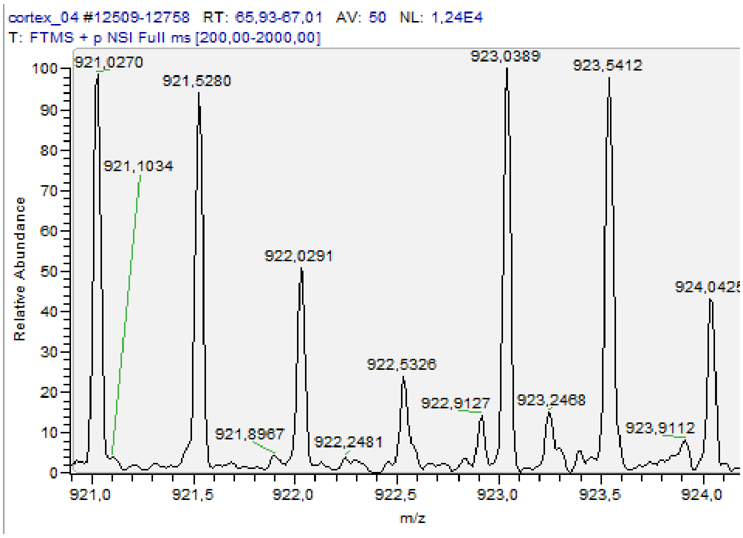

TVGDVNTDRPGLLDL 2+

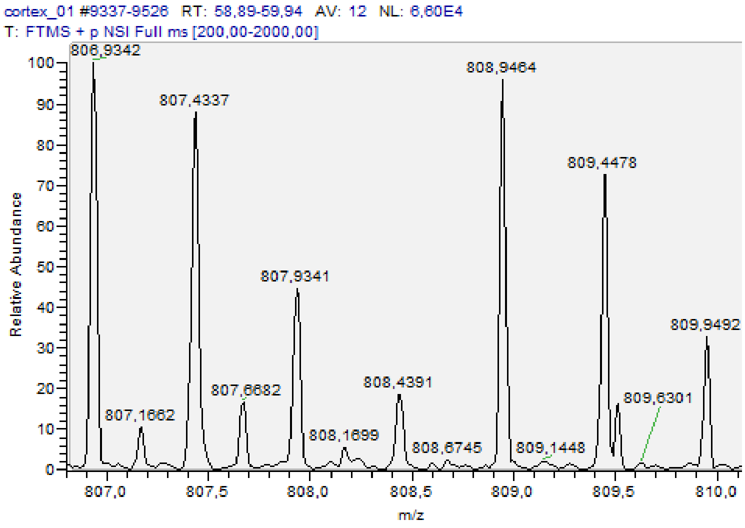

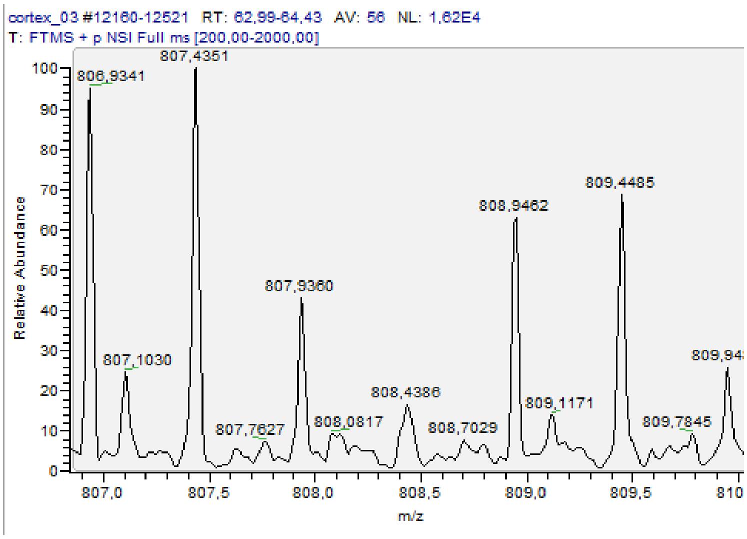

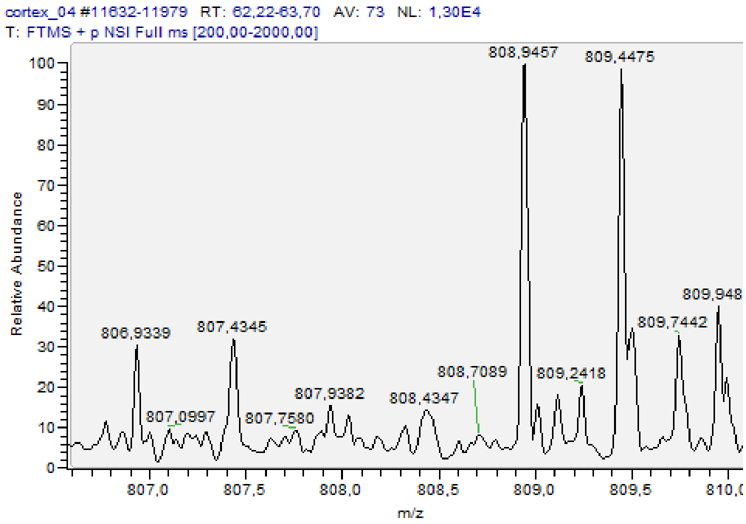

VFDVELLKLE 2+

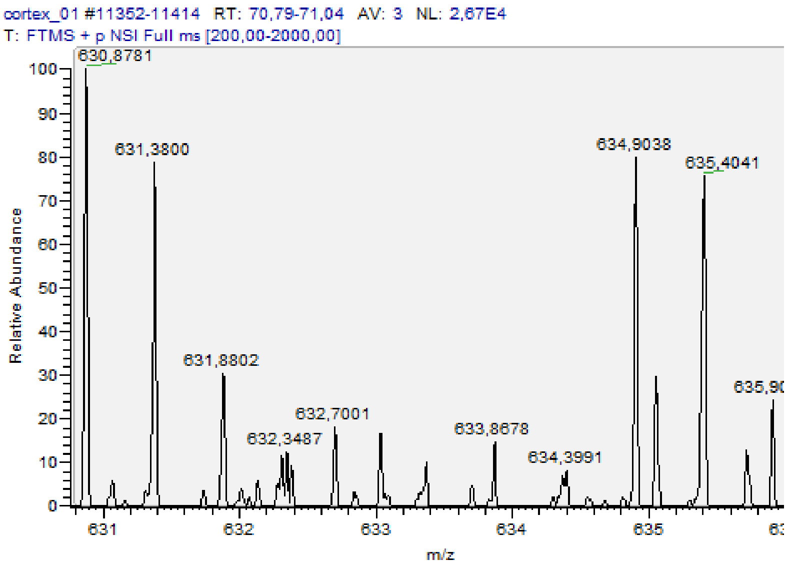

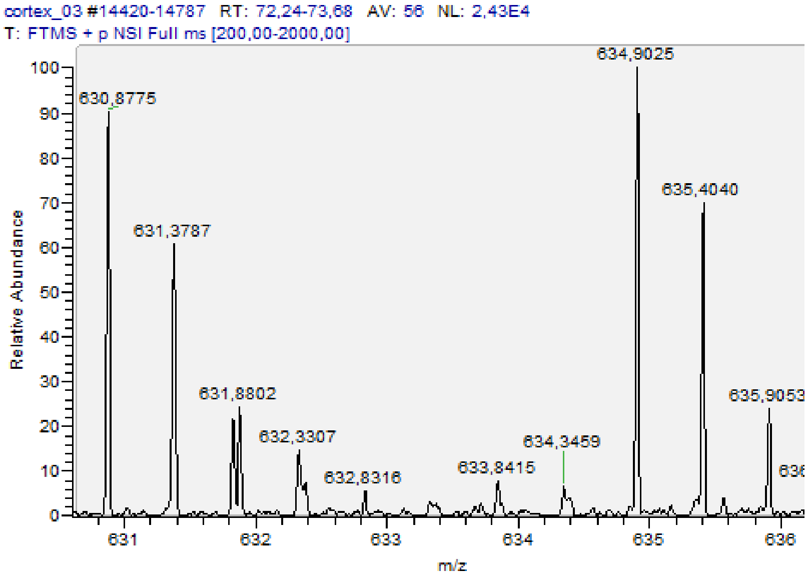

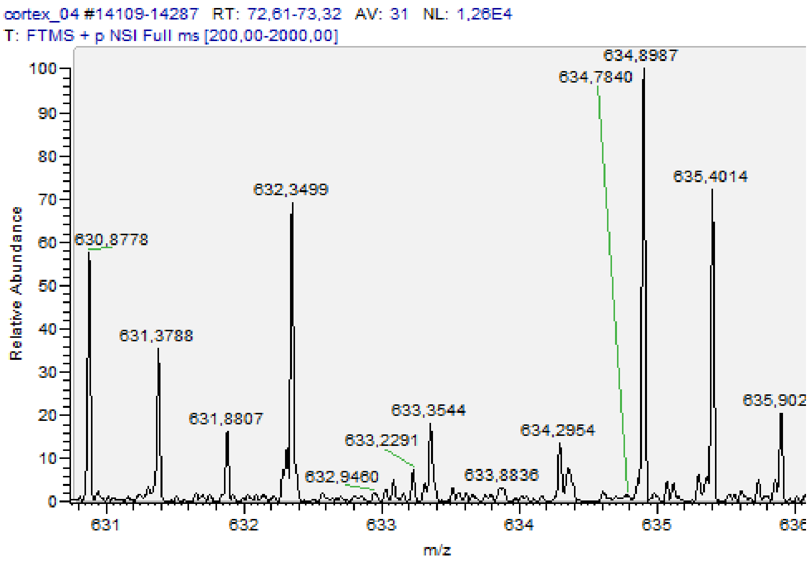

GISTPEELGLDKV 2+

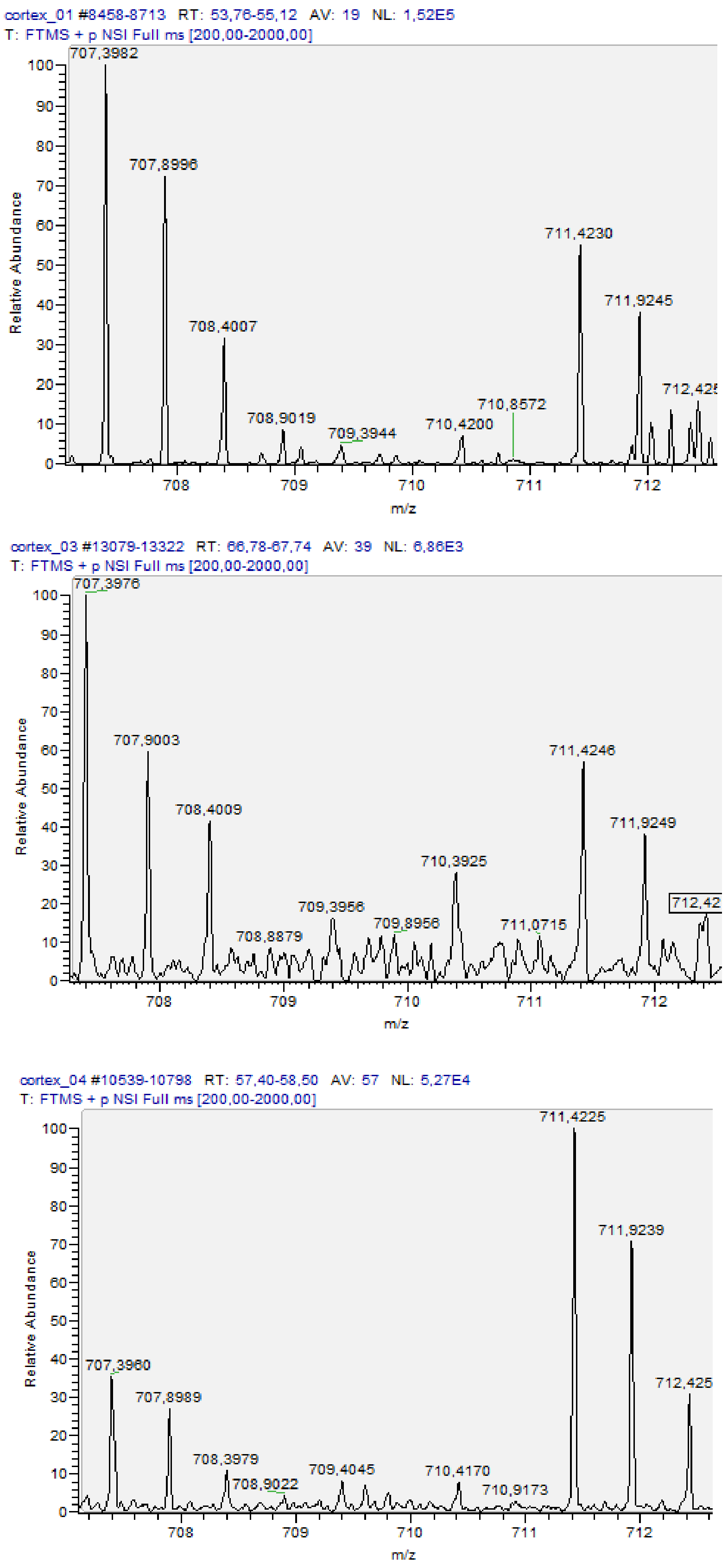

NDFASAVRILEV 2+

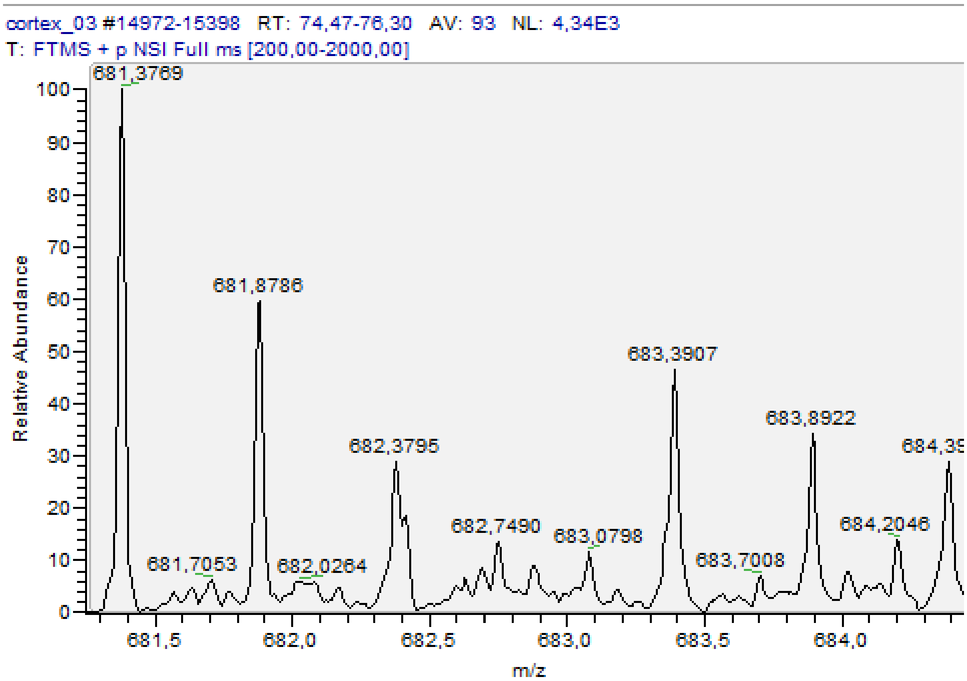

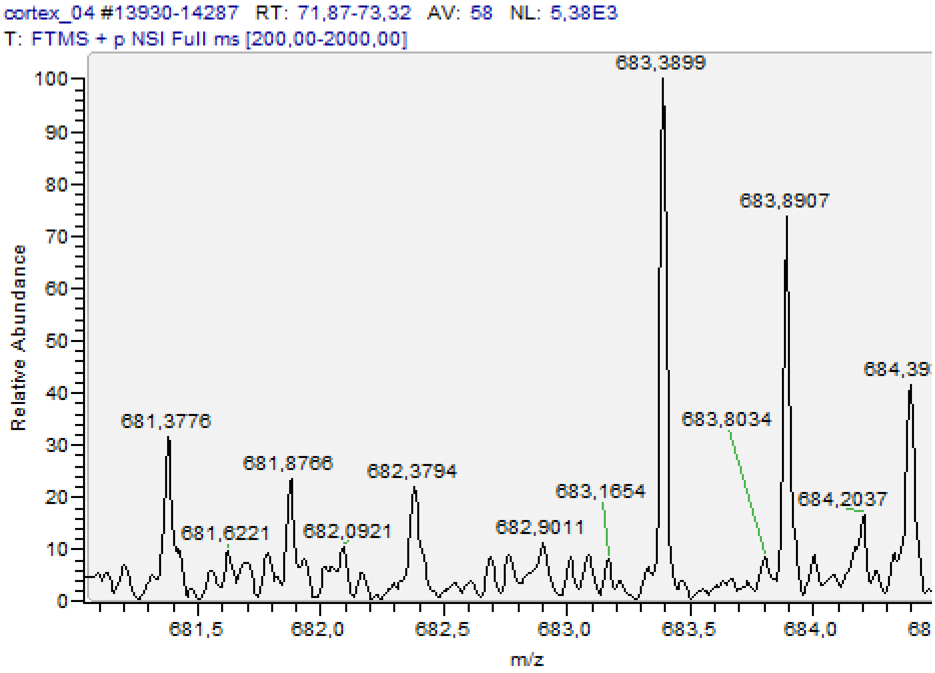

DGLDPGKLYTL 2+

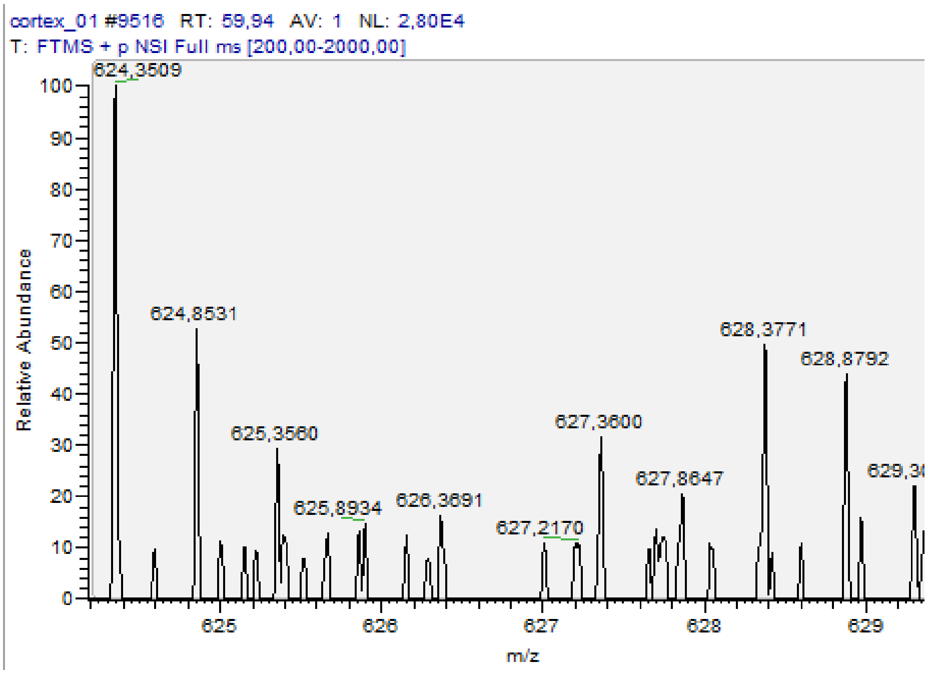

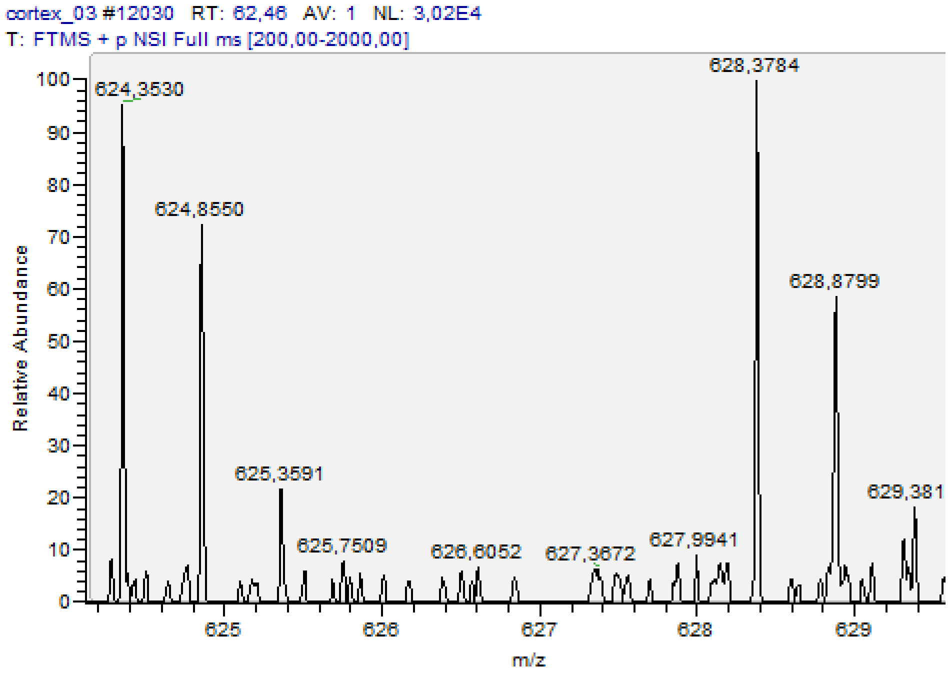

DDYVPKLYEQLSGK 2+

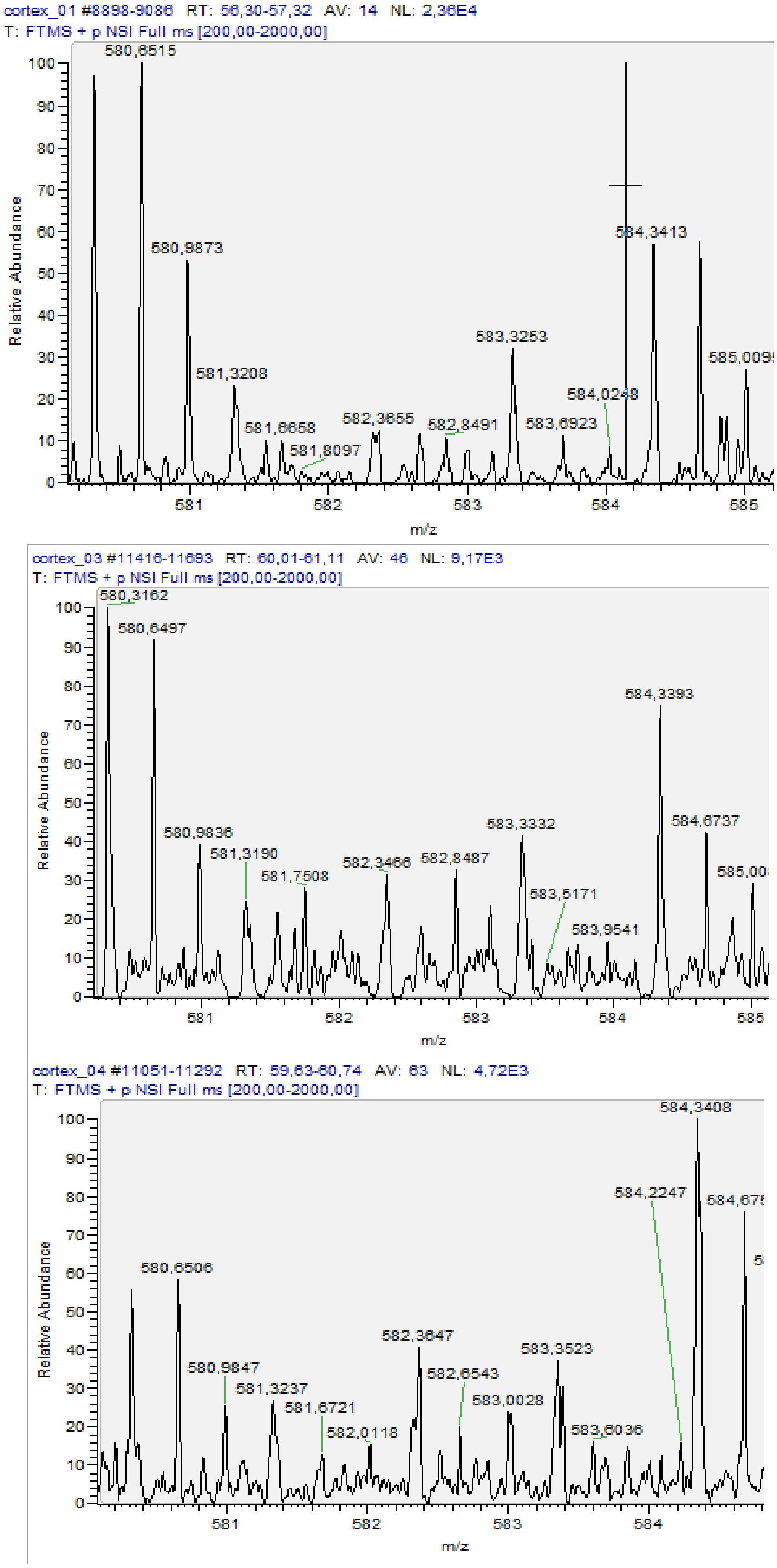

GRKGPGPGGPGGAGGARGGAGGGPSGD 4+

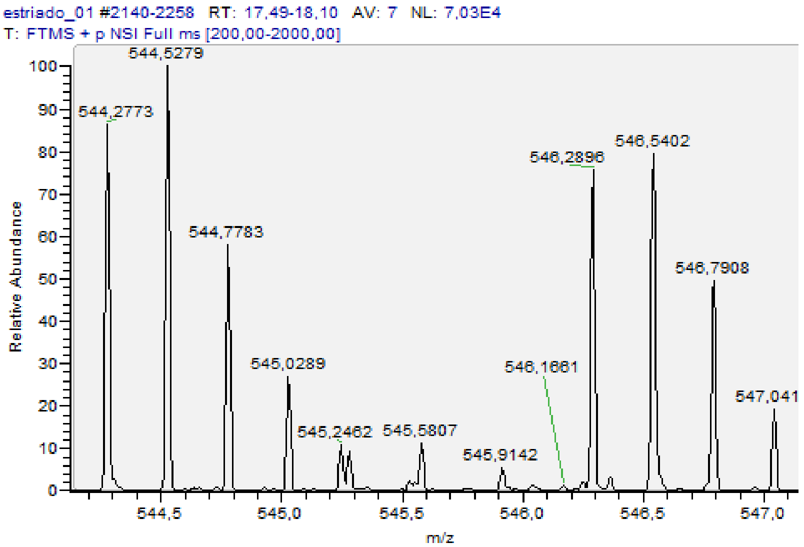

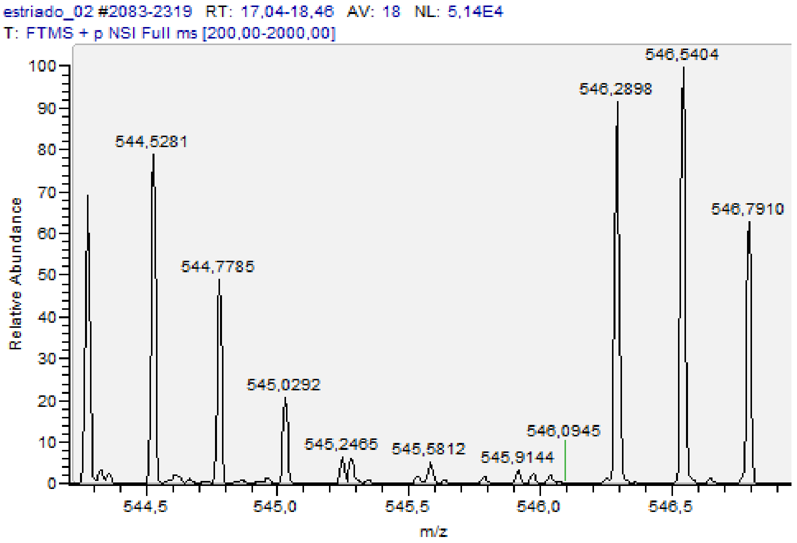

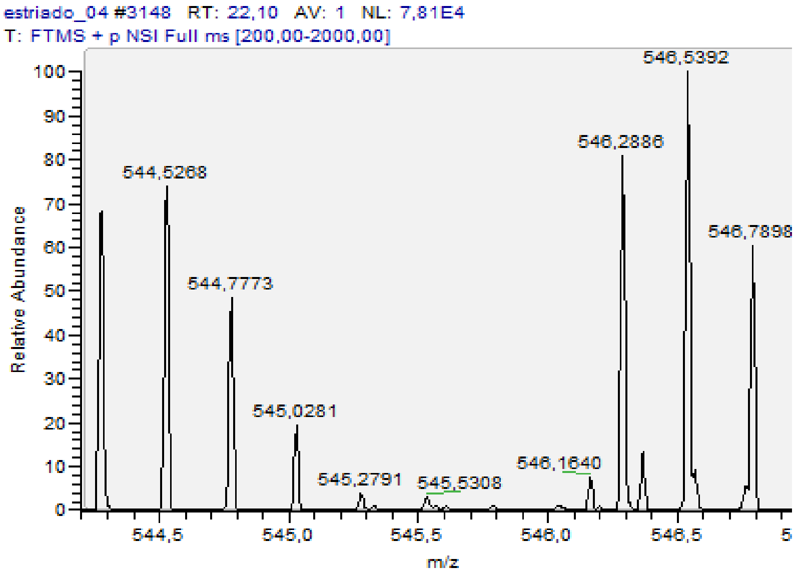

KGPGPGGPGGAGGARGGAGGGPSGD 3+

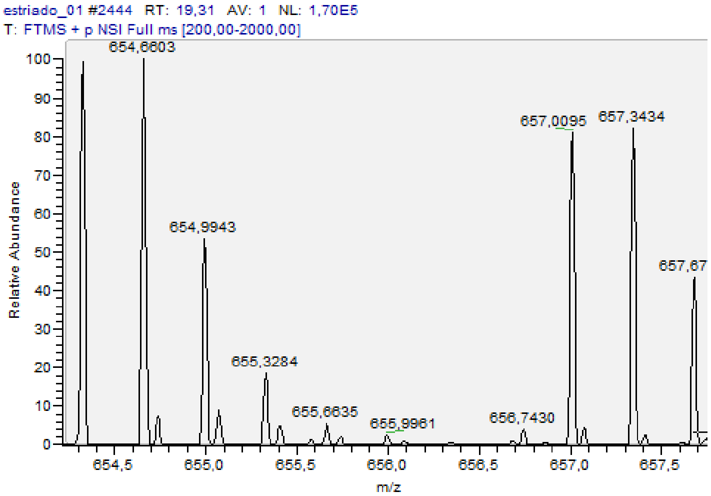

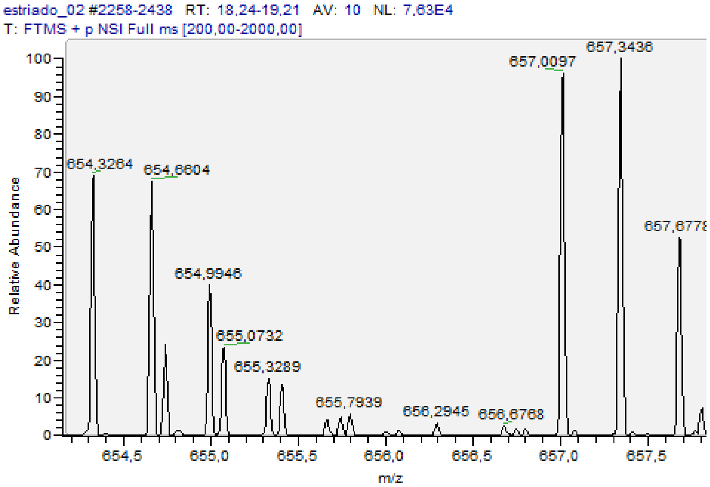

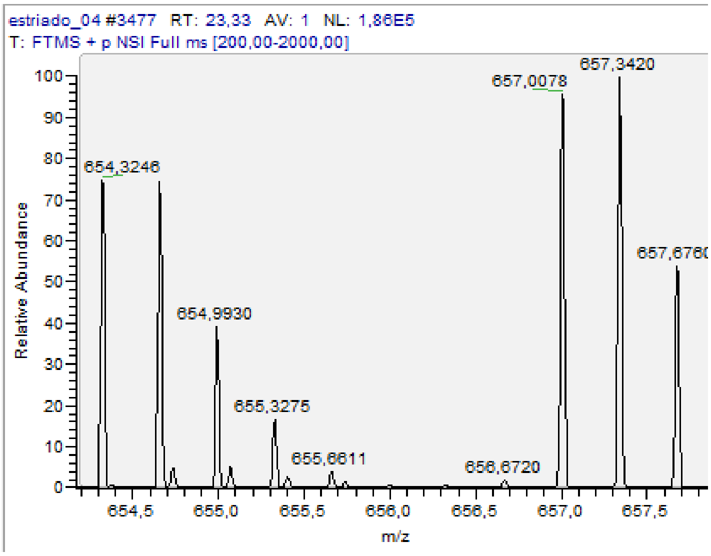

KGPGPGGPGGAGGARGGAGGGPSGD 2+

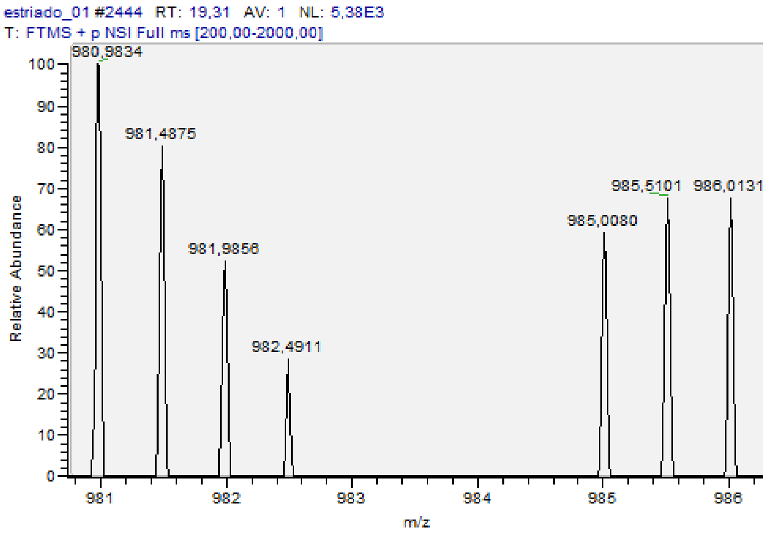

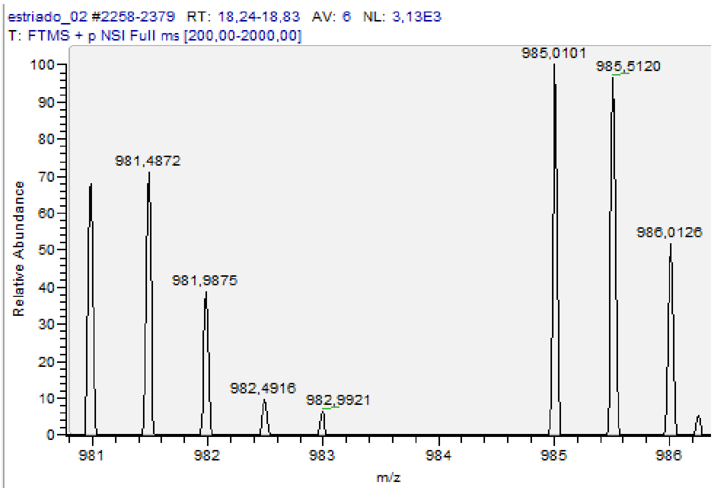

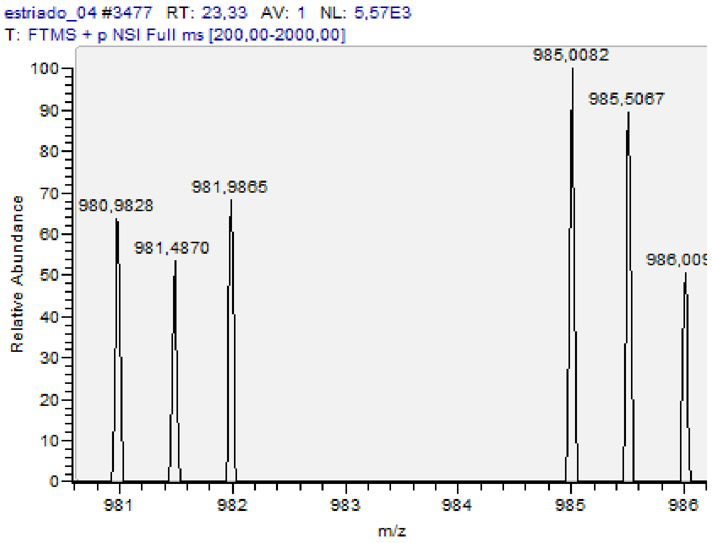

RKGPGPGGPGGAGGARGGAGGGPSGD 3+

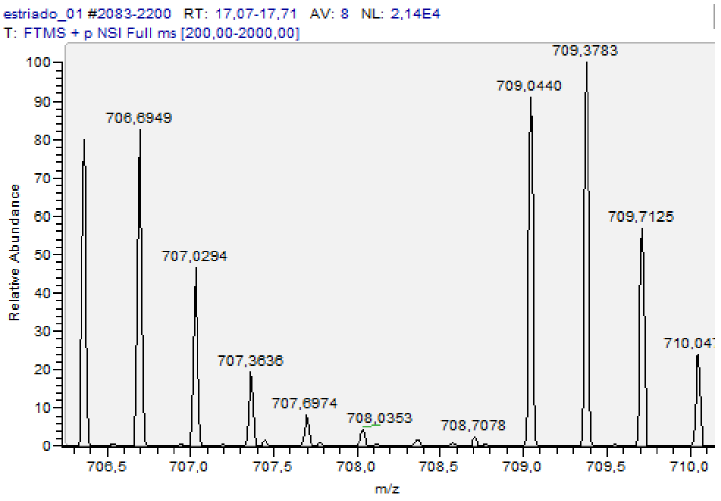

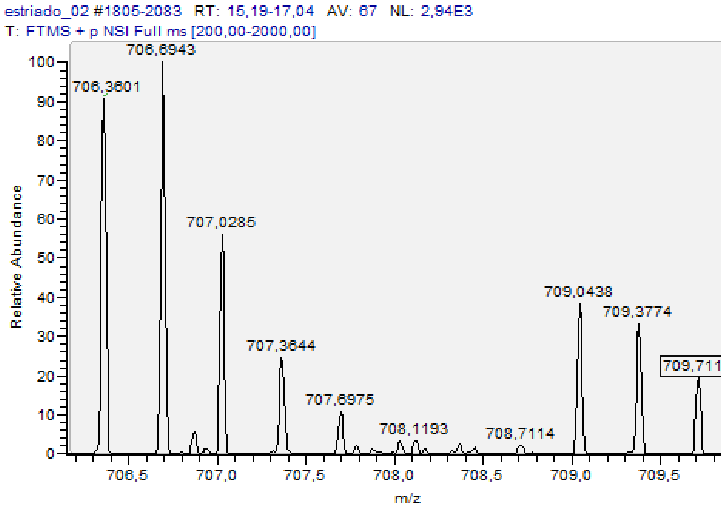

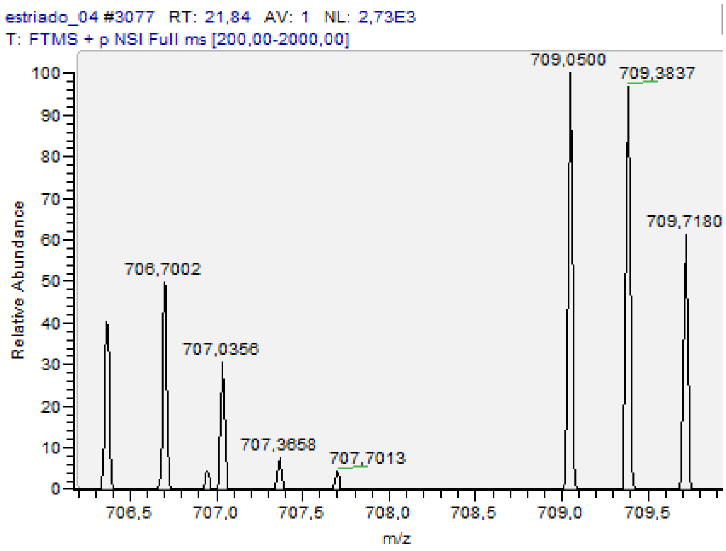

ATVGDVNTDRPGLLDL 2+

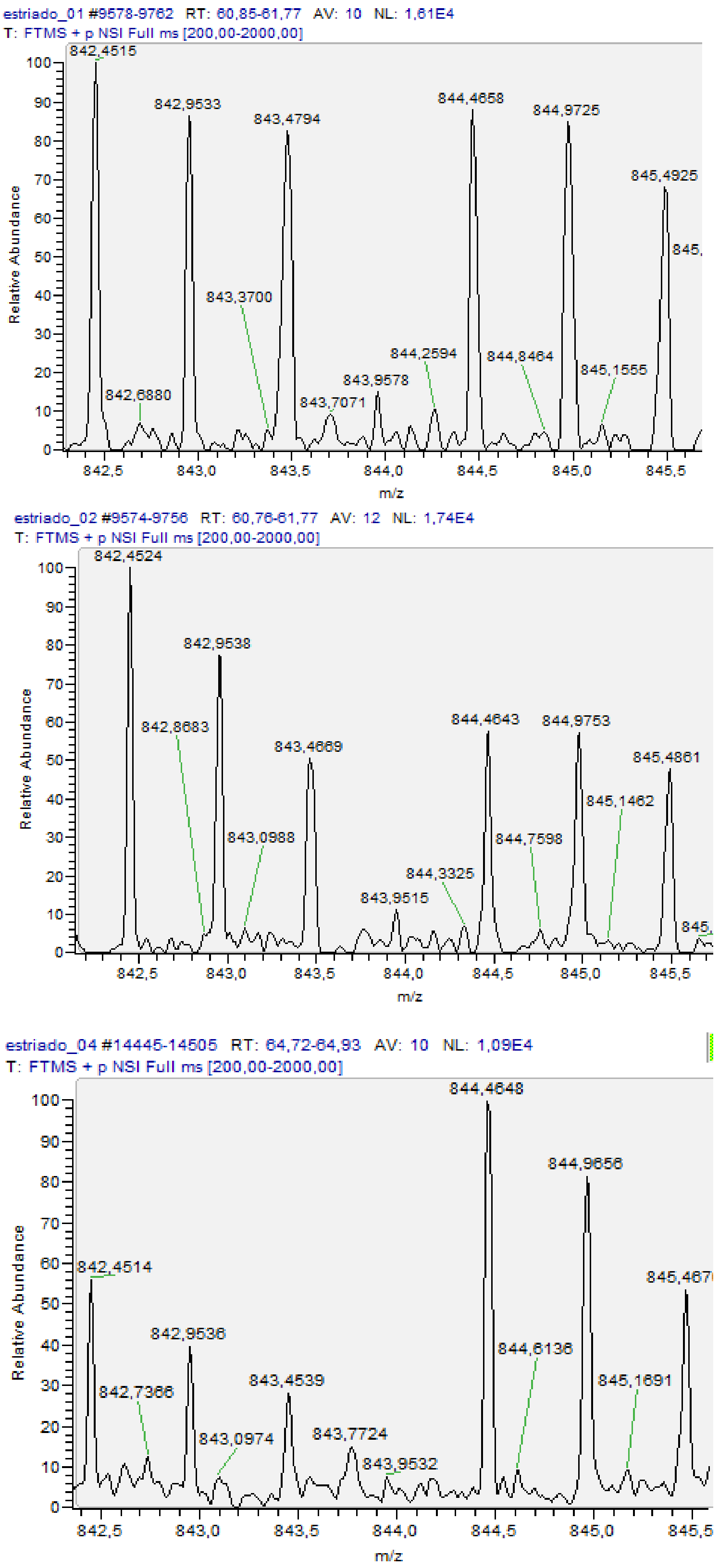

KQATVGDVNTDRPGLLDL 2+

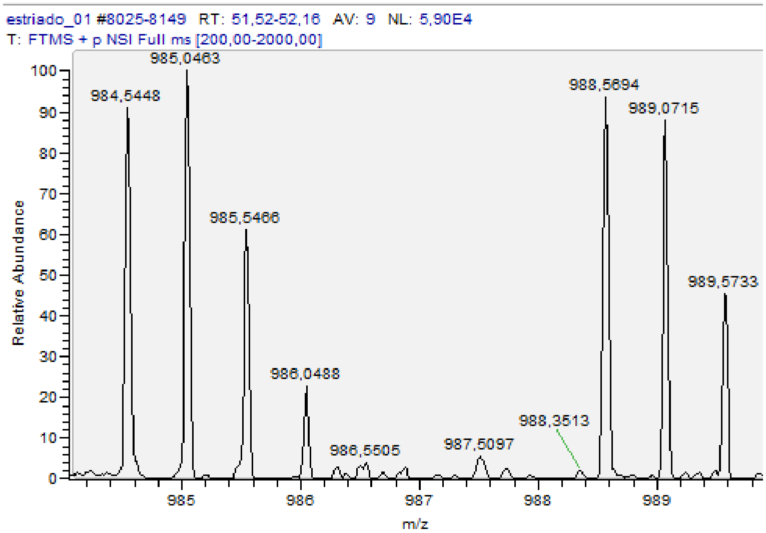

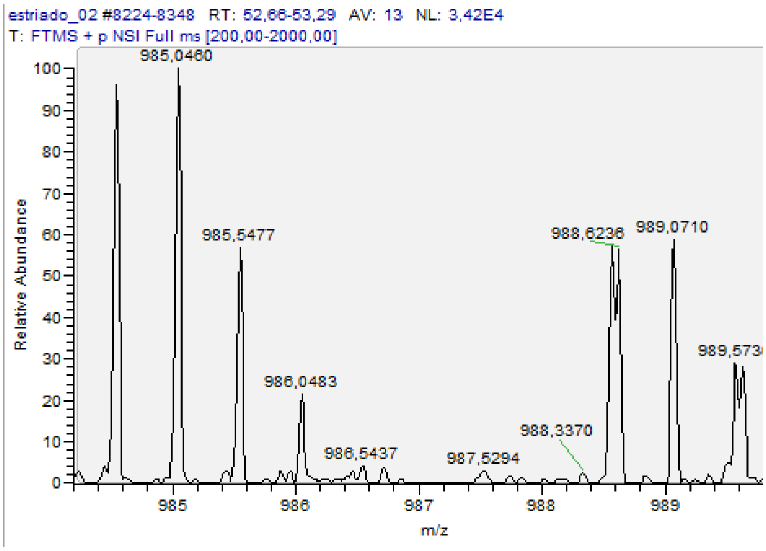

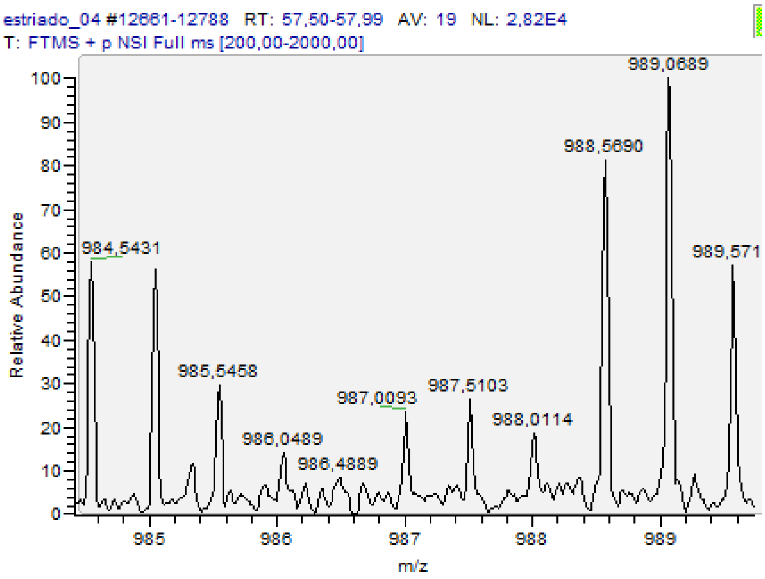

KQATVGDVNTDRPGLLDL 3+

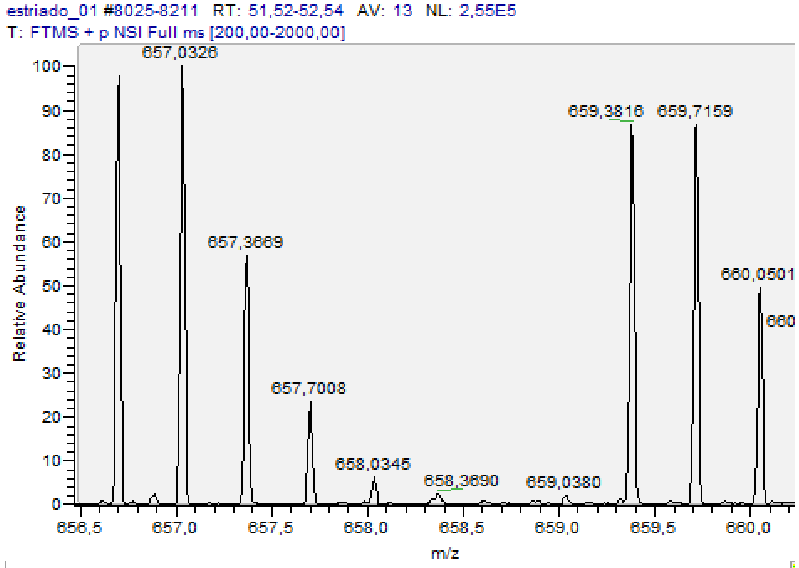

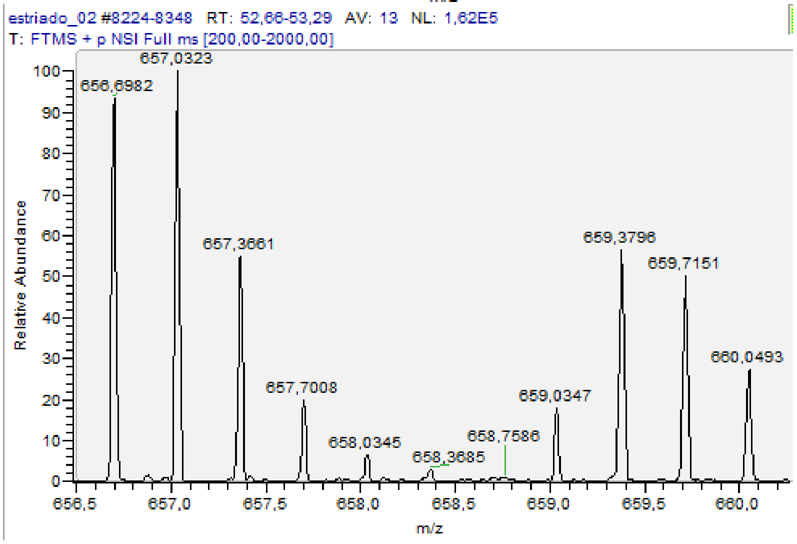

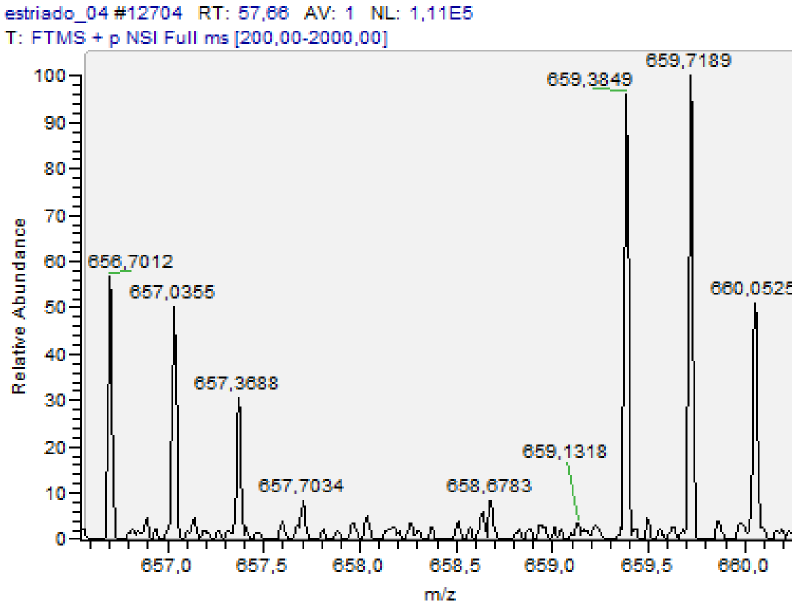

SHFKQATVGDVNTDRPGLLDL 2+

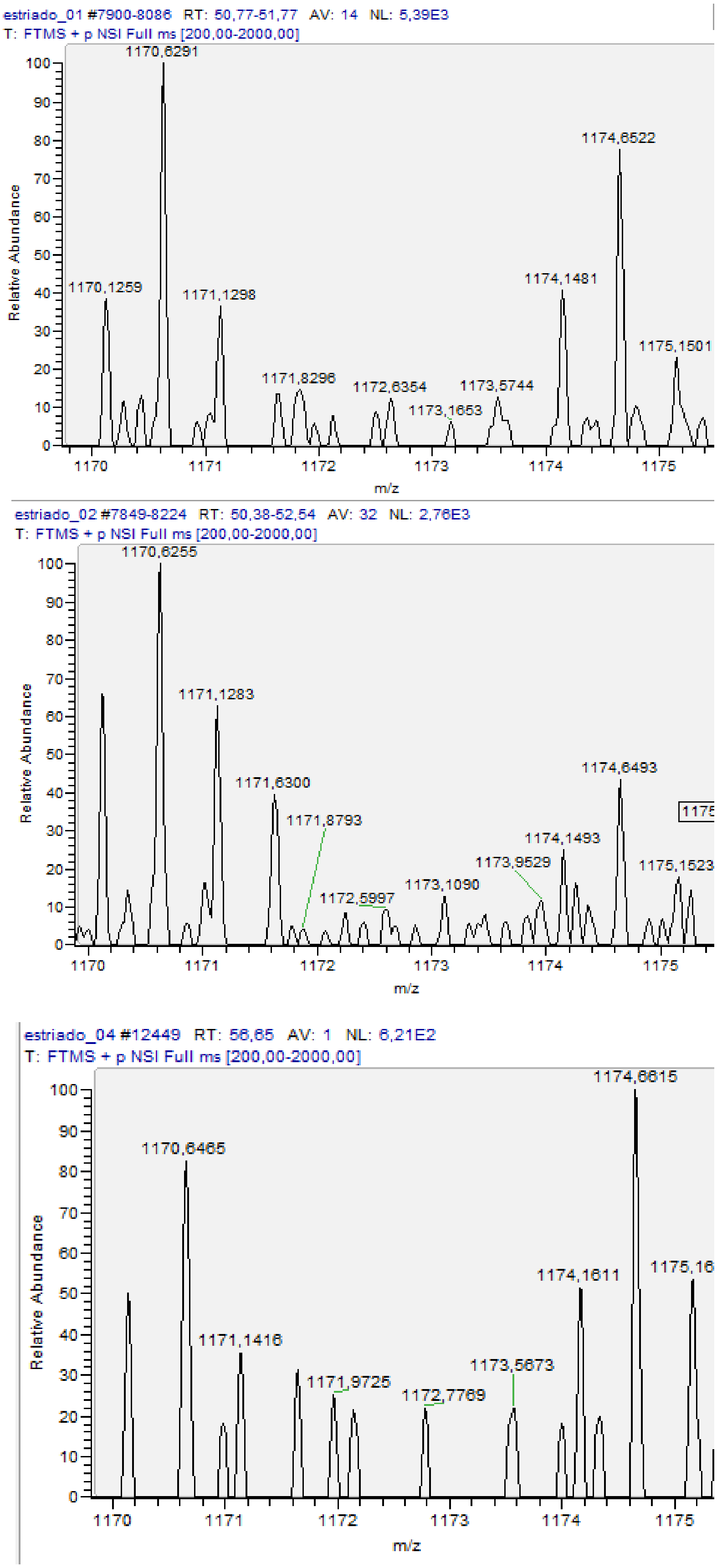

TVGDVNTDRPGLLDL 2+

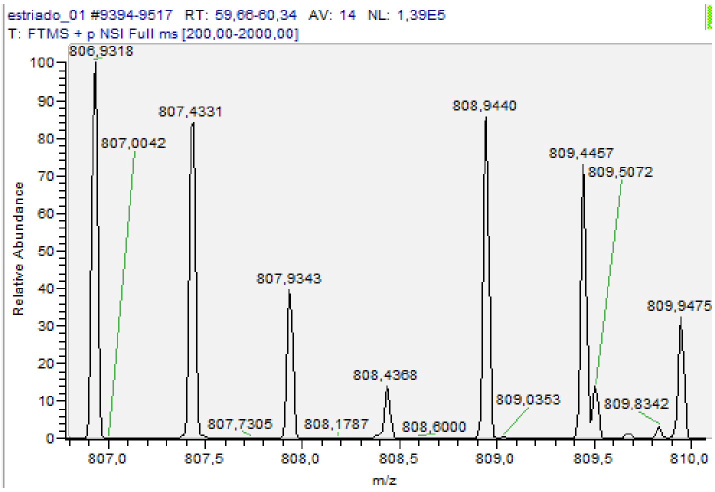

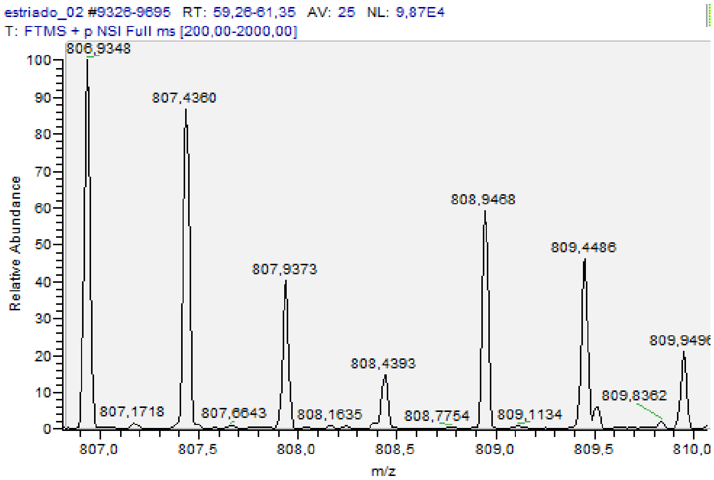

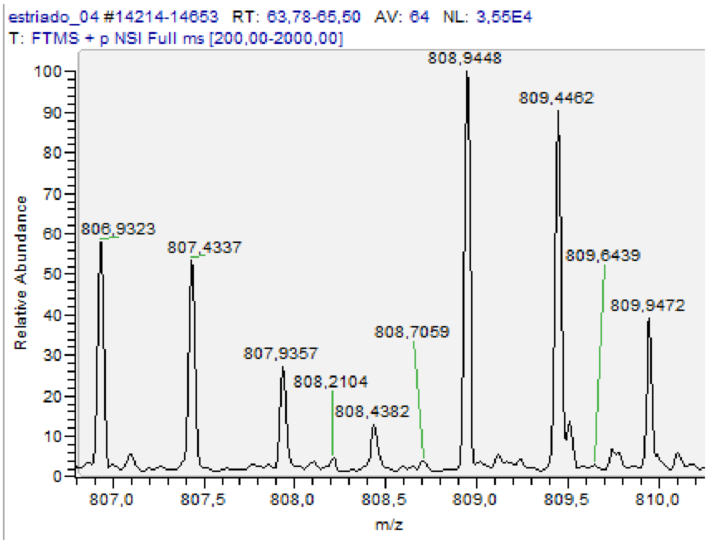

VEKVDELKKKYGI 3+

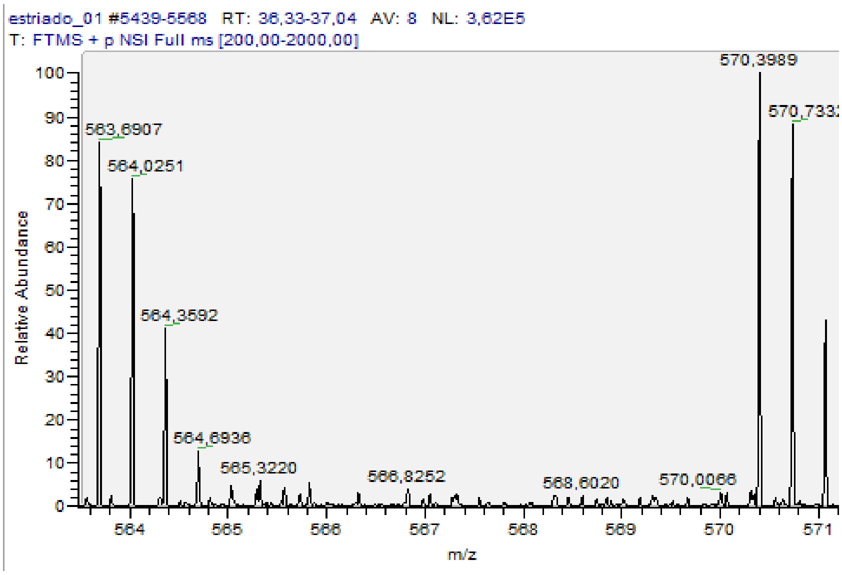

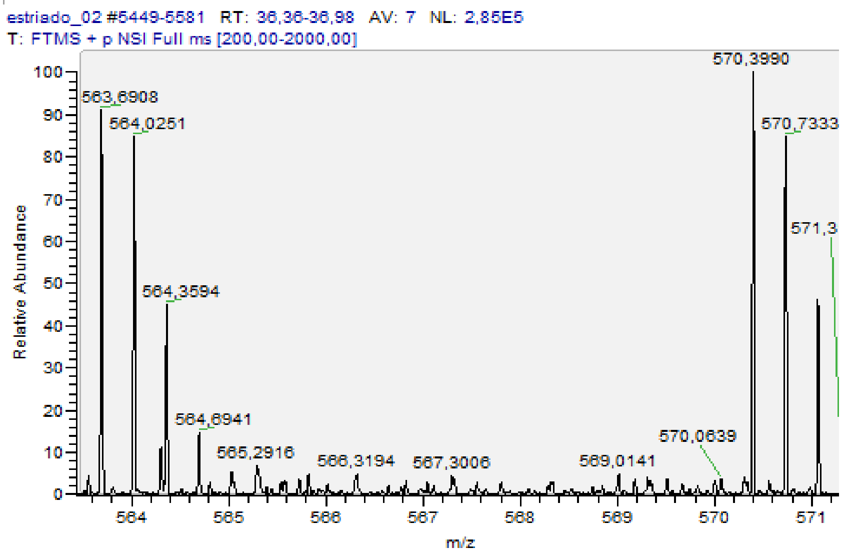

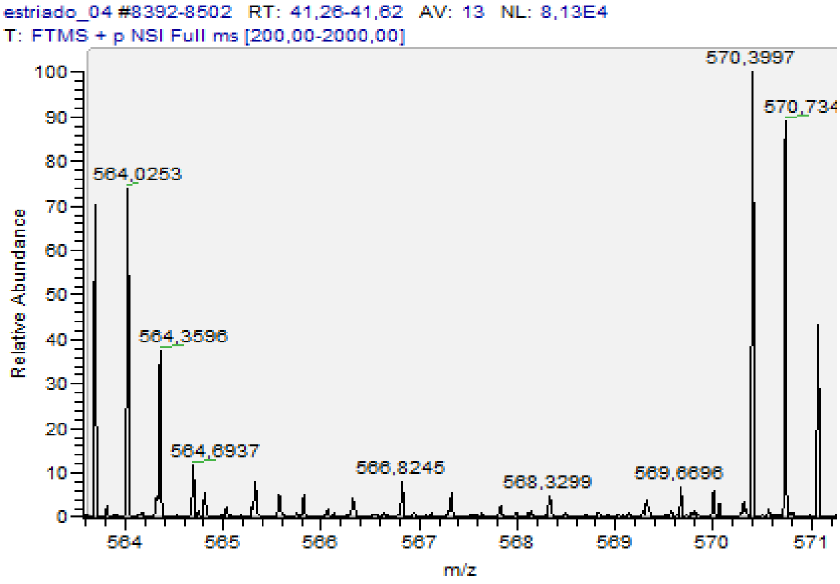

KGNDISSGTVLSDYVGSGPPSGTGL 2+

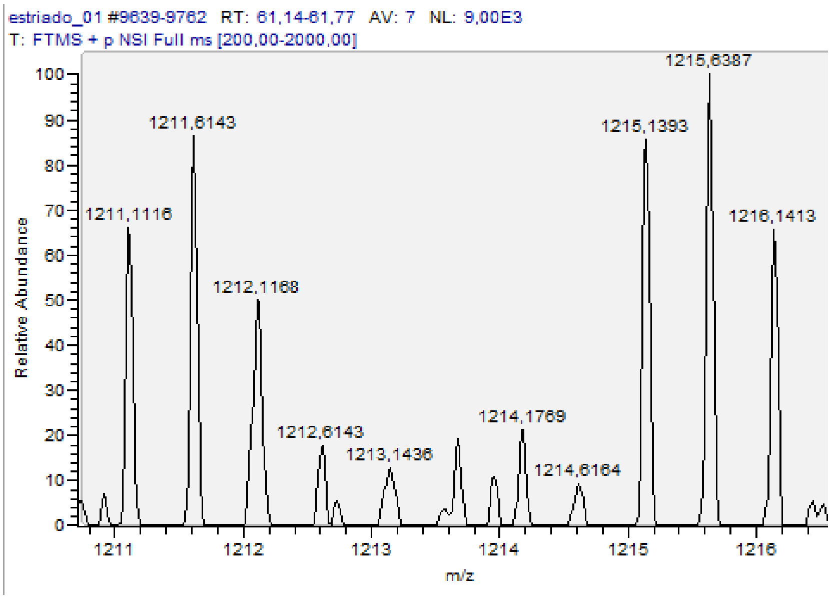

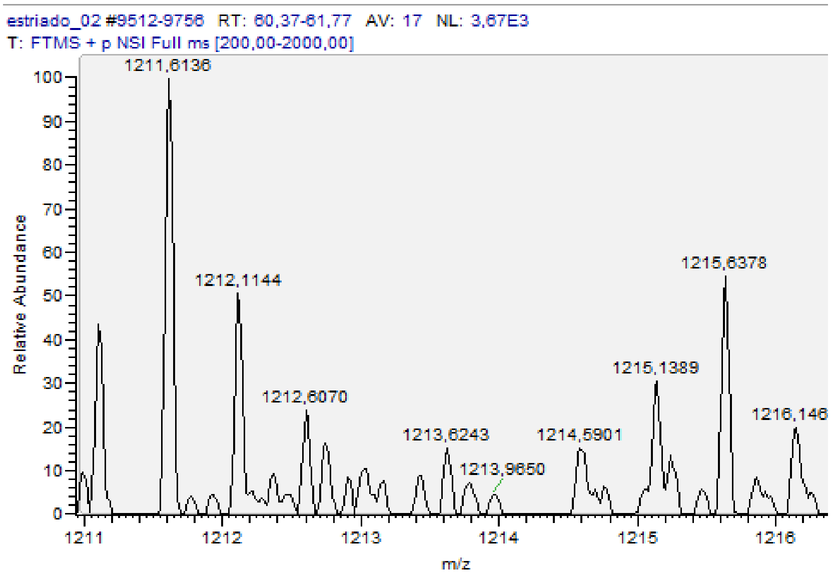

AGVTVDELGKVL 2+

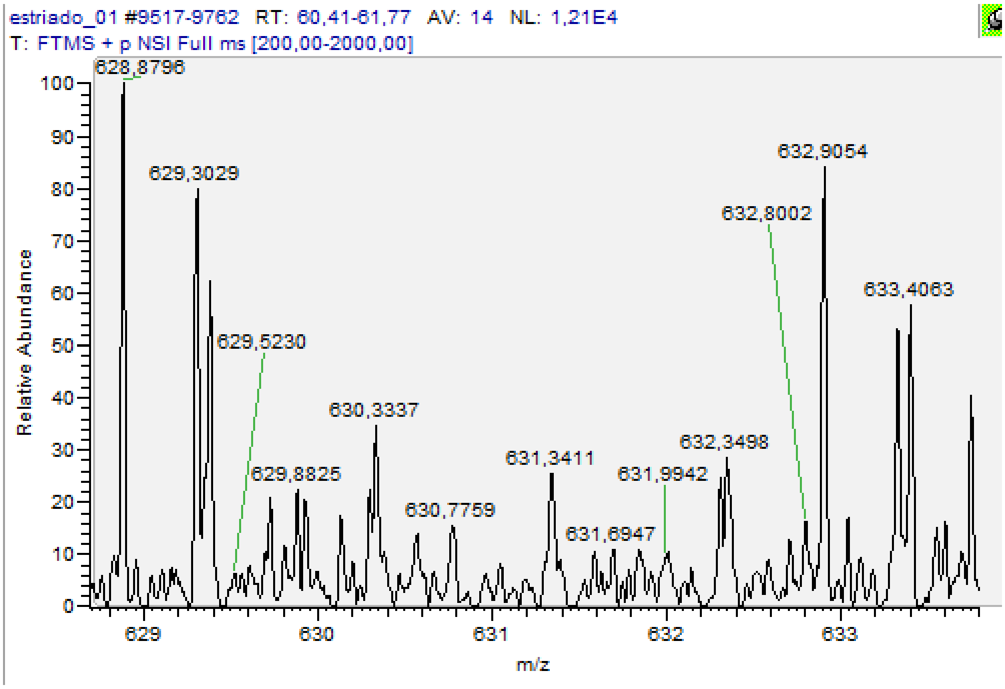

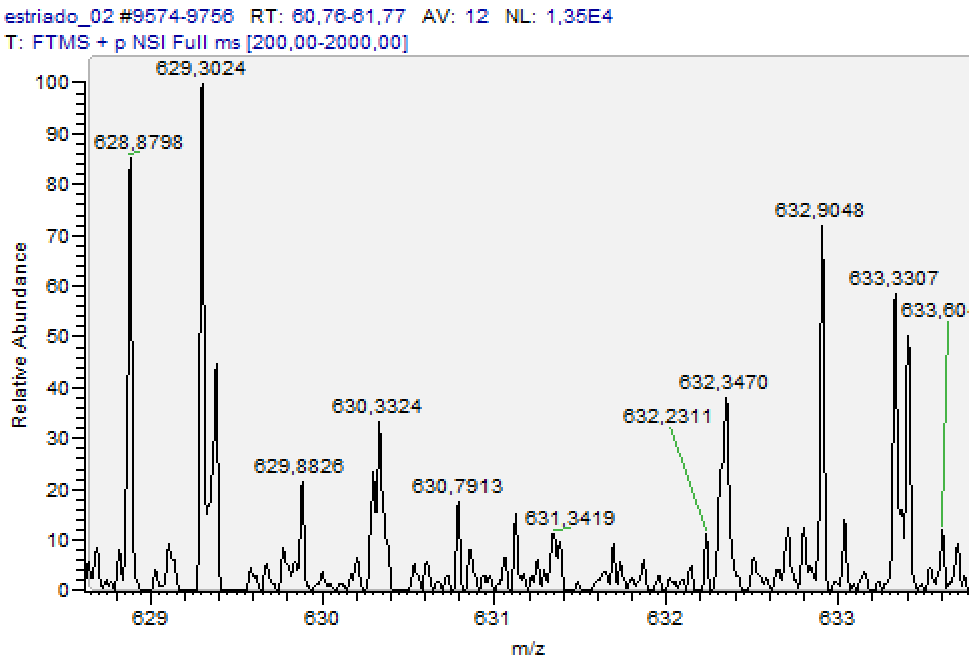

GVTVDELGKVLTPTQV 2+

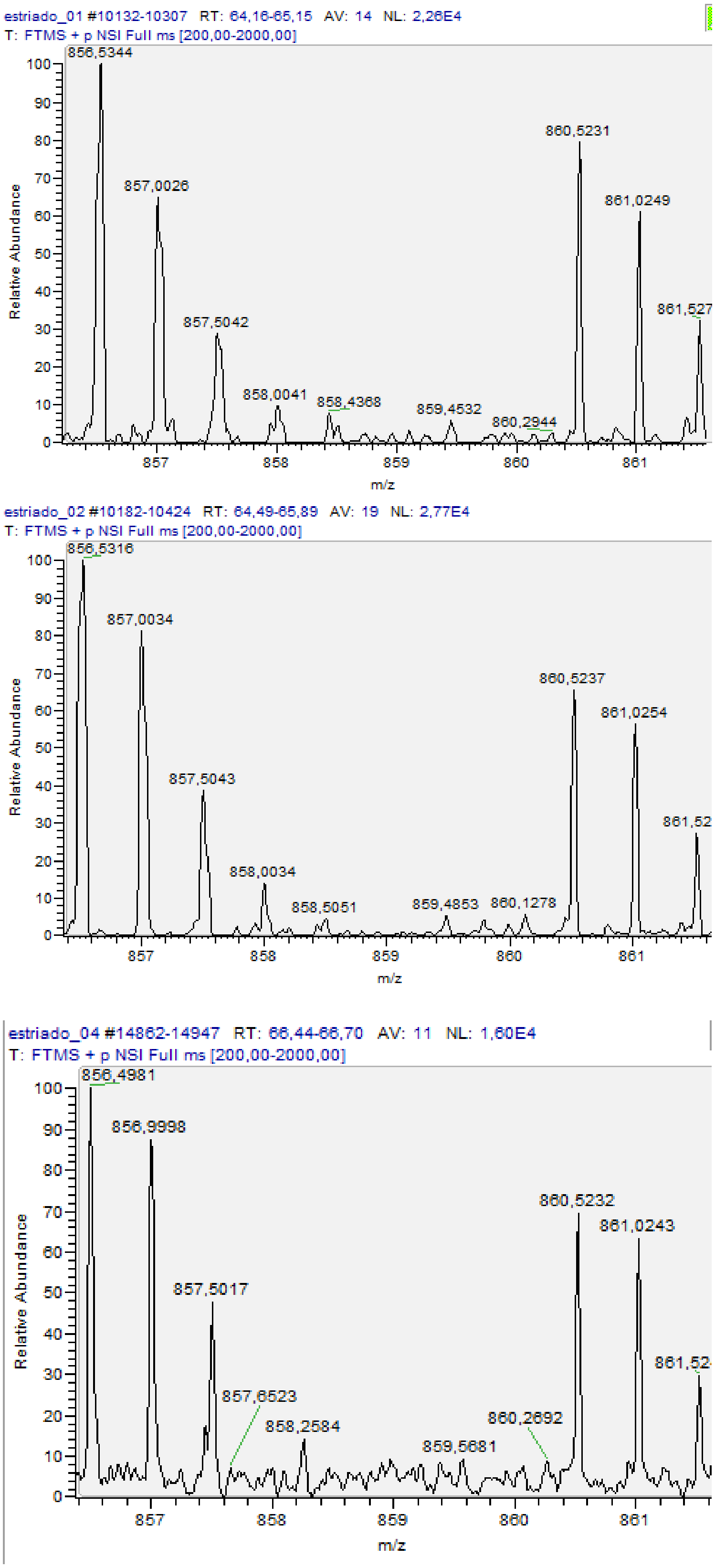

DDYVPKLYEQLSGK 2+

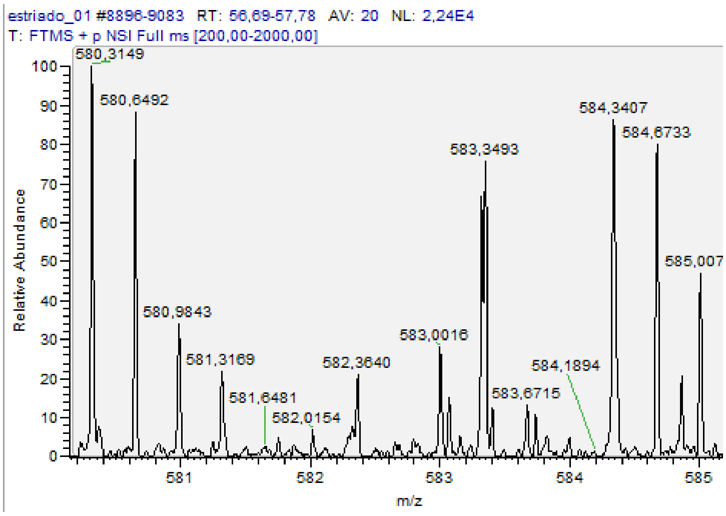

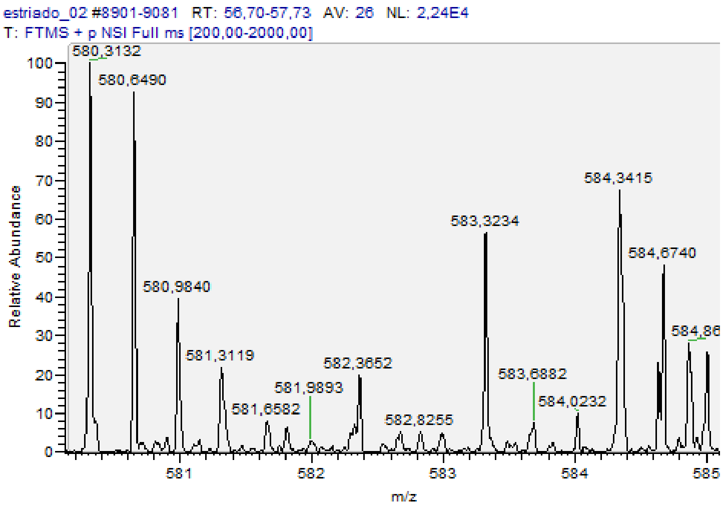

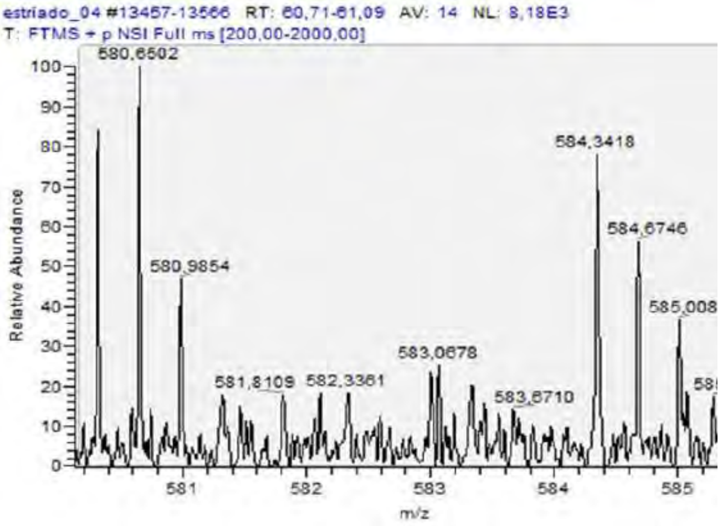

AGVTVDELGKVLTPTQV 2+

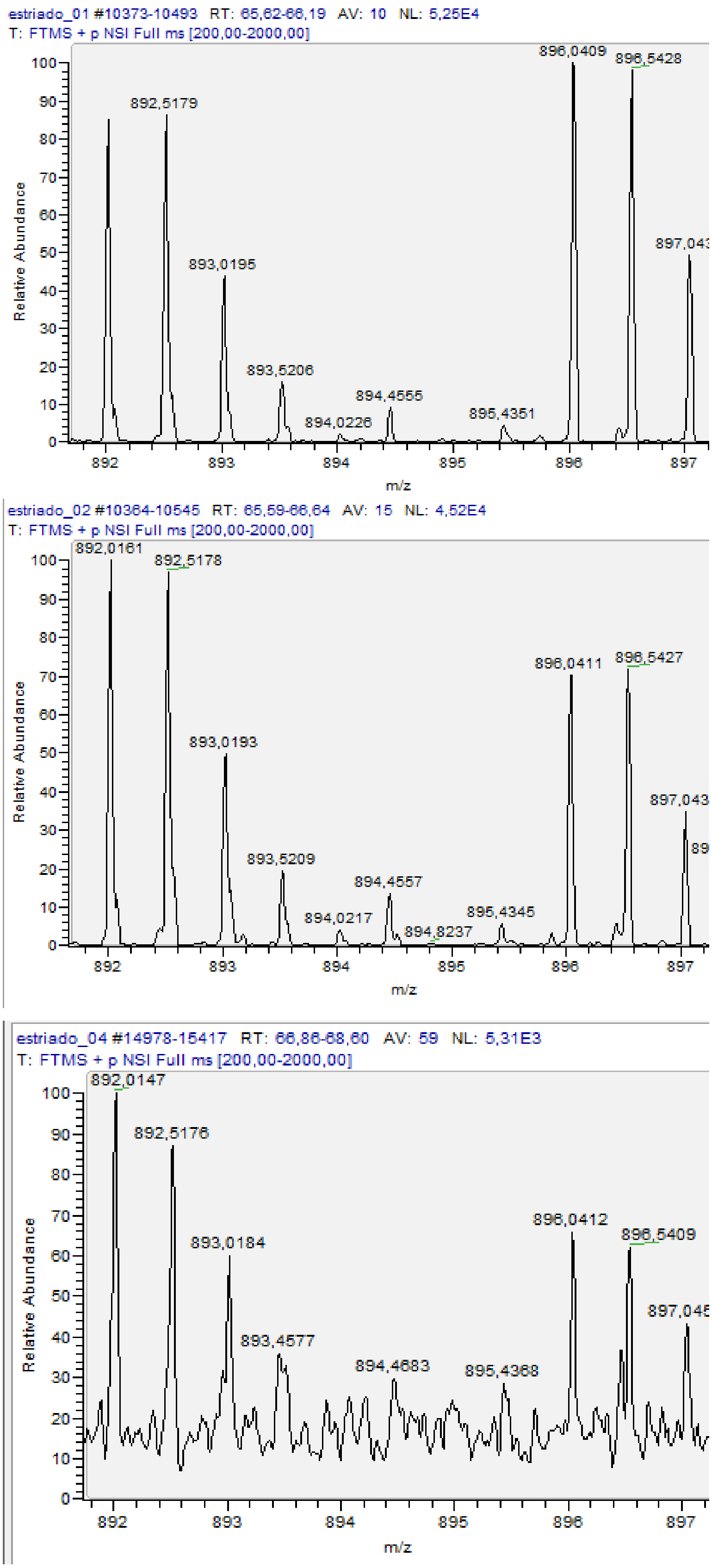

ADDEPLGRVSF 2+

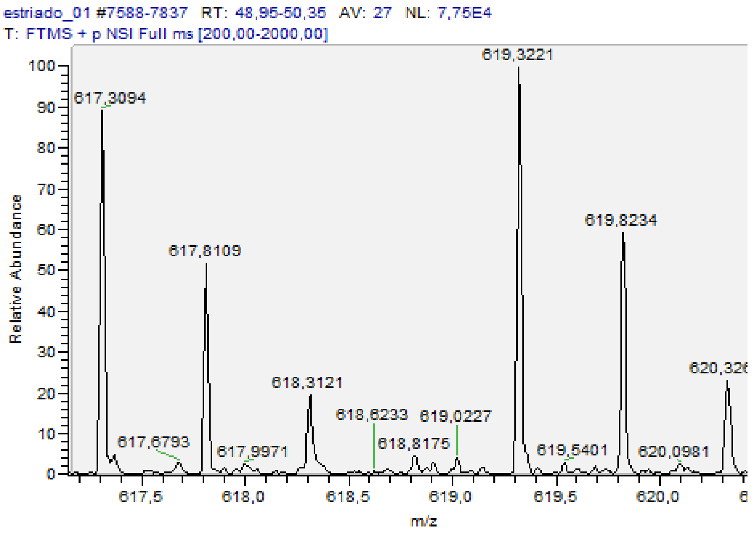

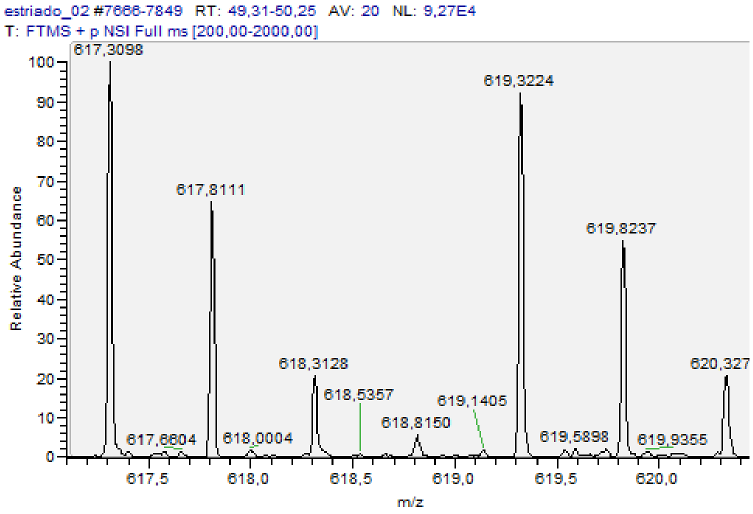

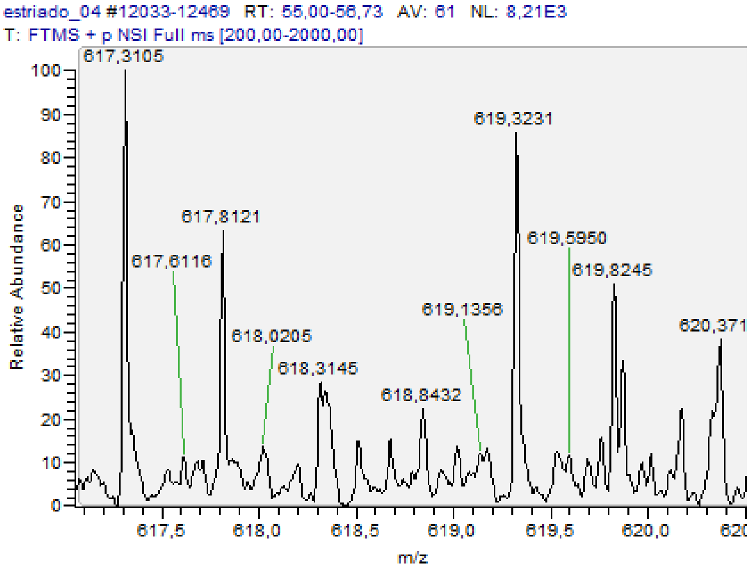

ADKVPKTAENFRAL 2+

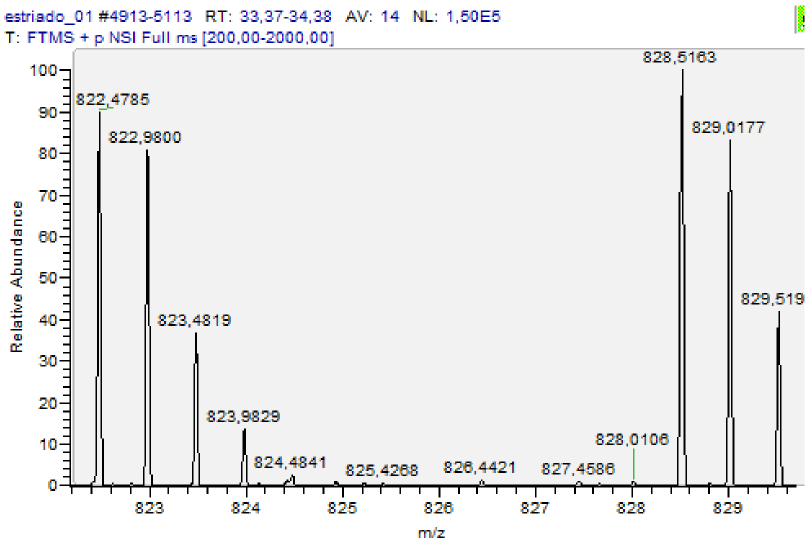

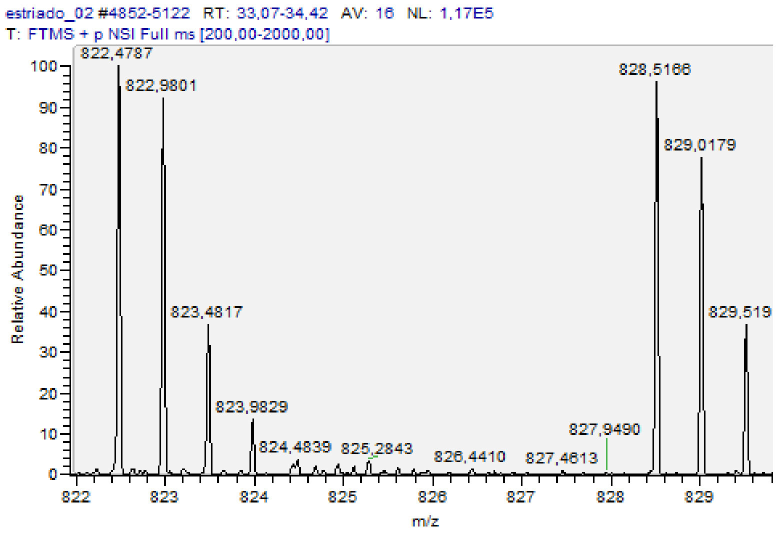

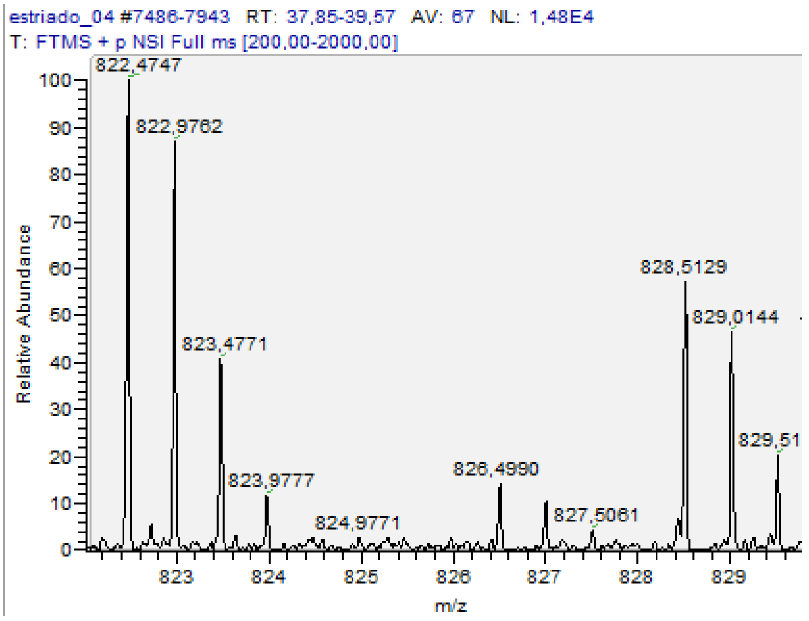

ADKVPKTAENFR 3+

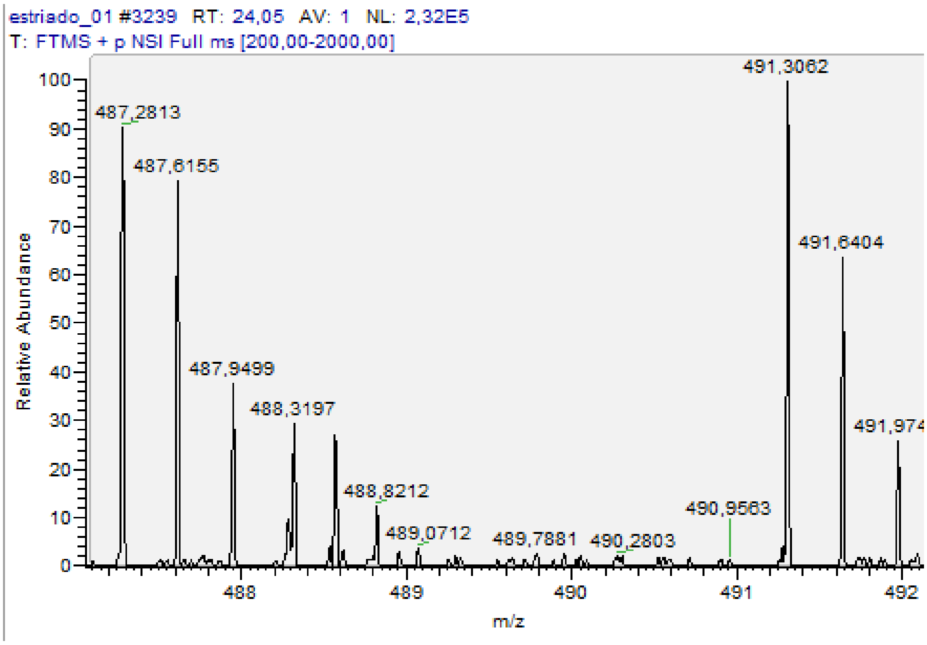

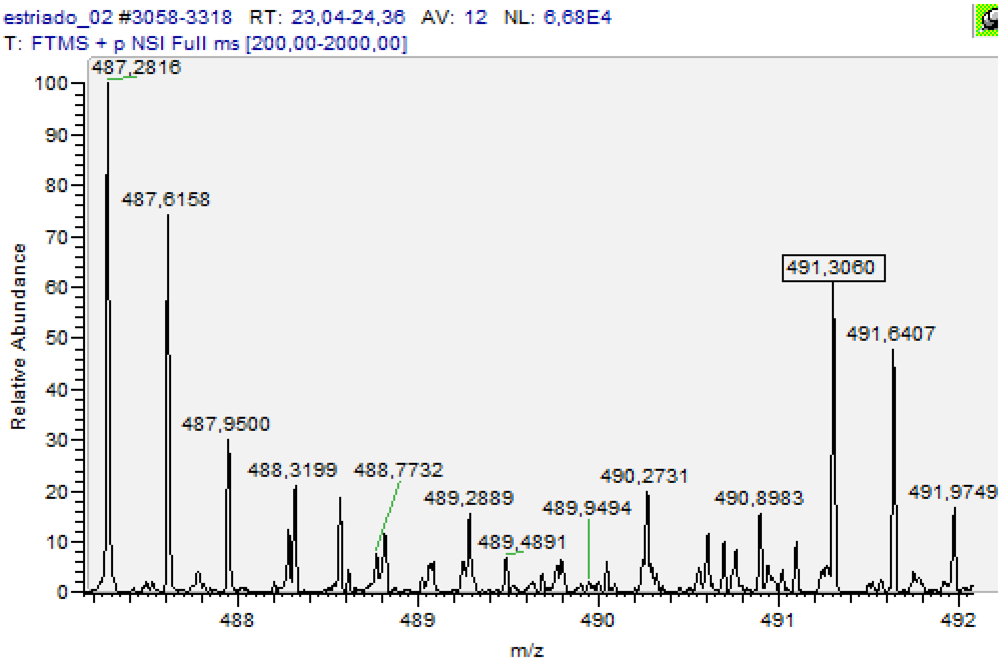

ELFADKVPKTAENF 2+

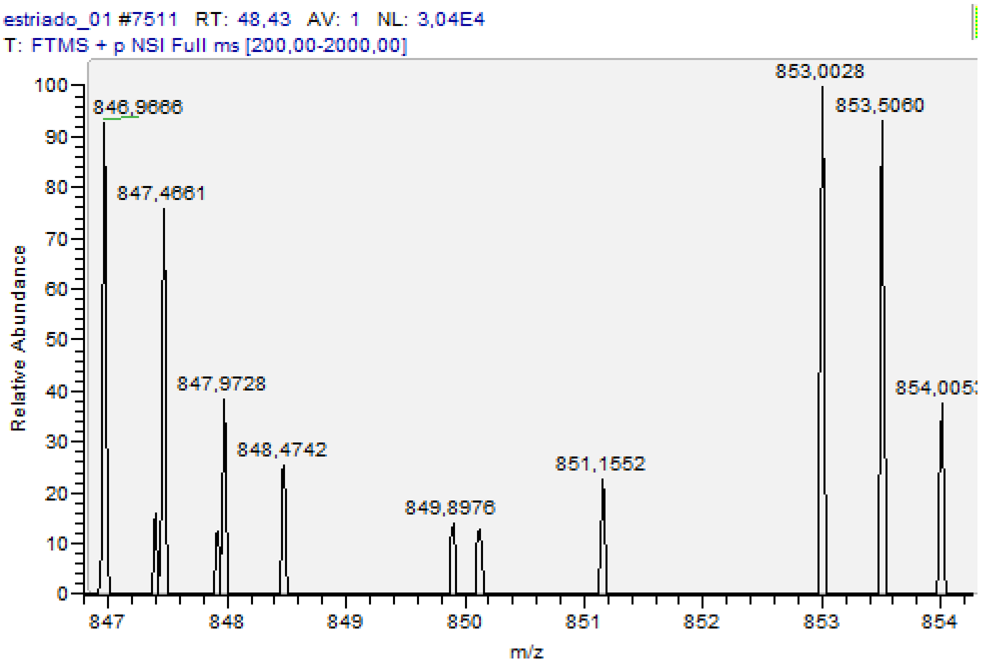

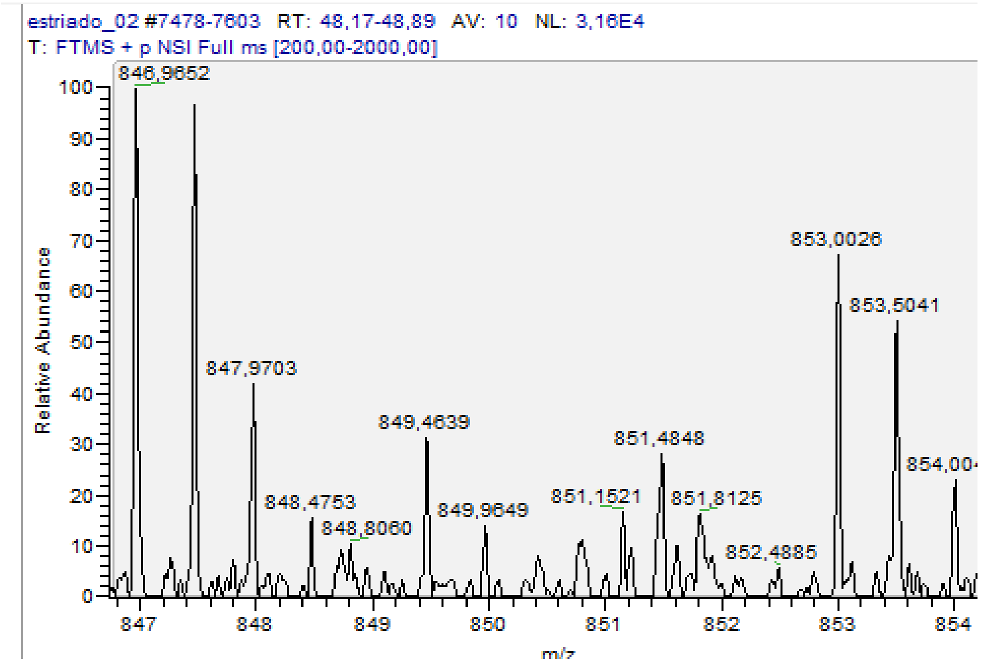

ASAPLVETSTPLRL 2+

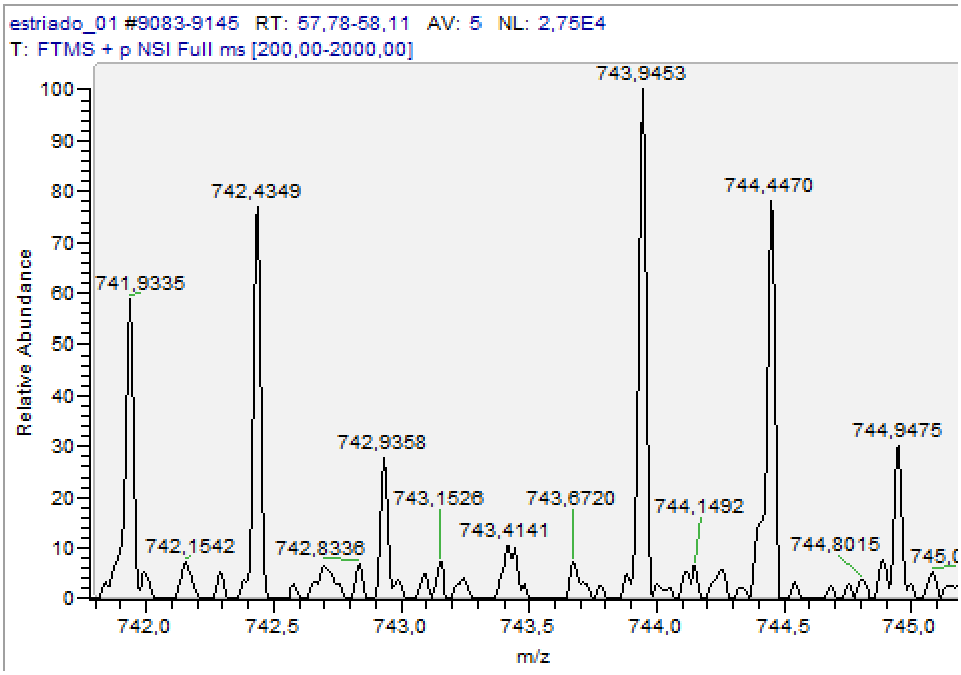

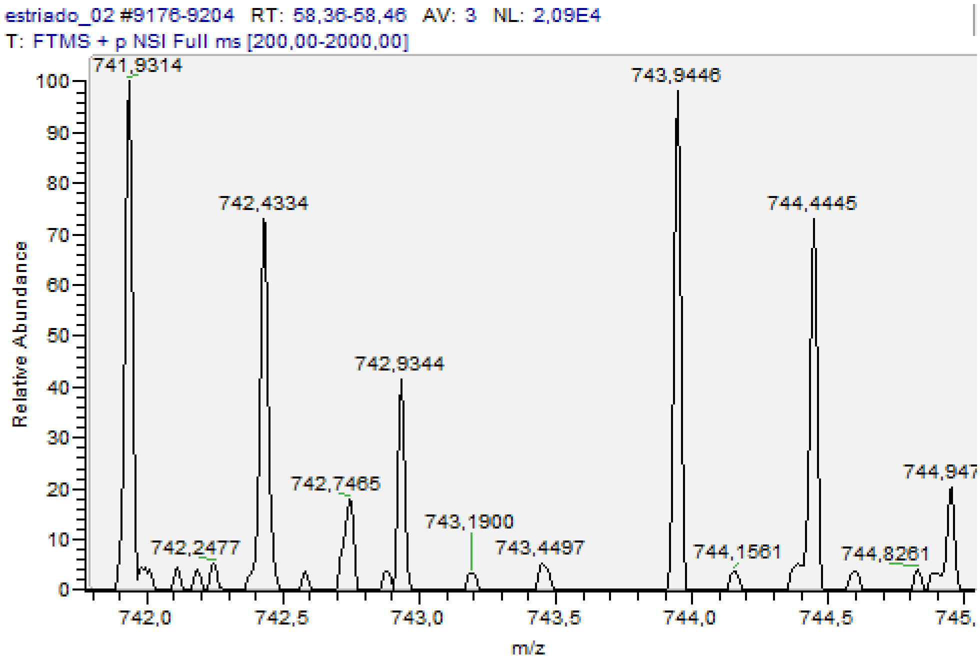

SLSAASAPLVETSTPLRL 2+

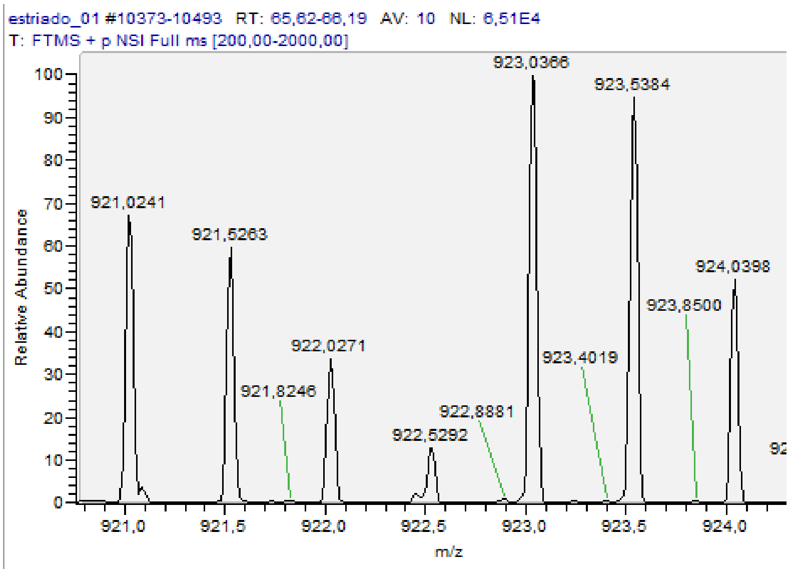

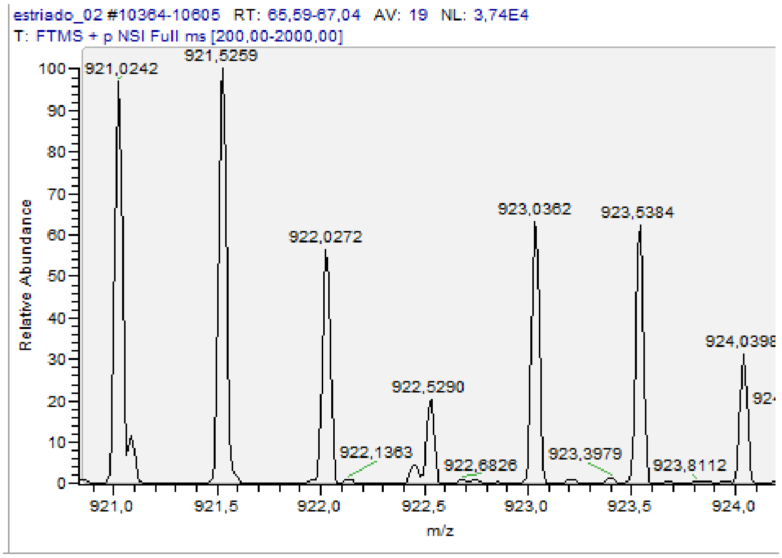

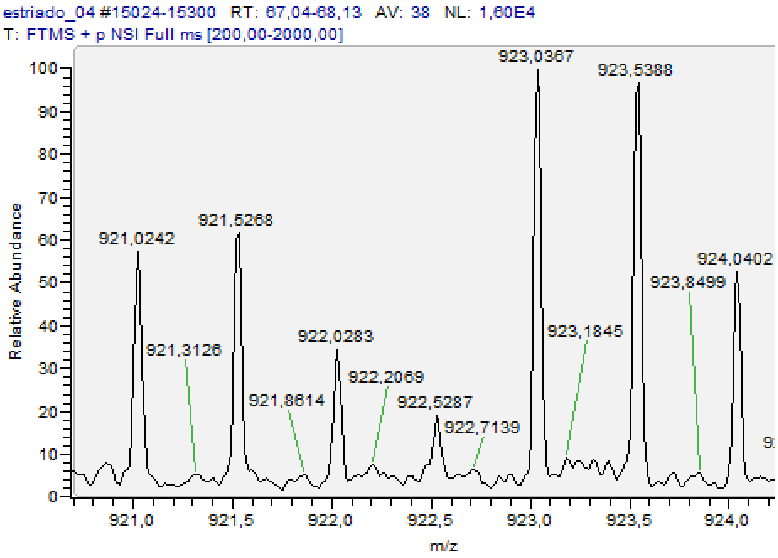

SANSNPAMAPRE 2+

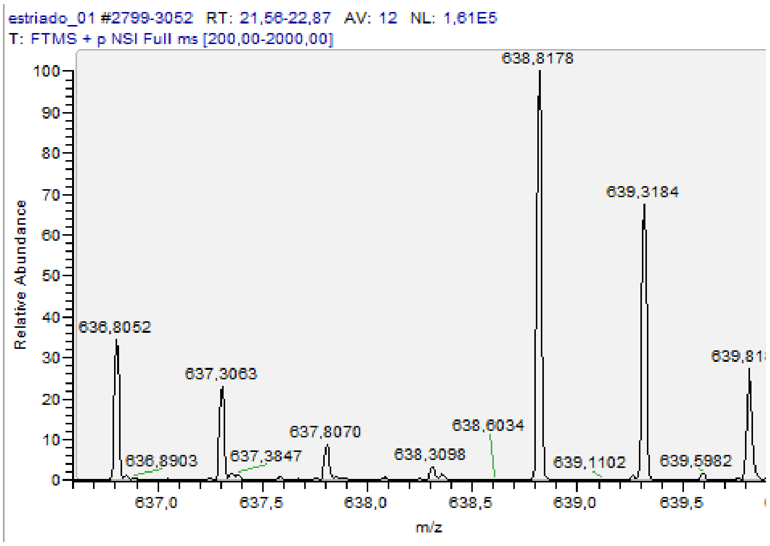

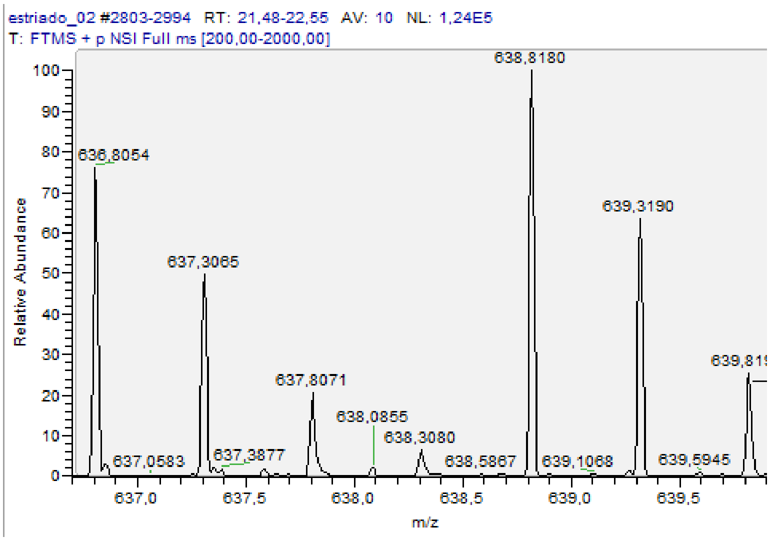

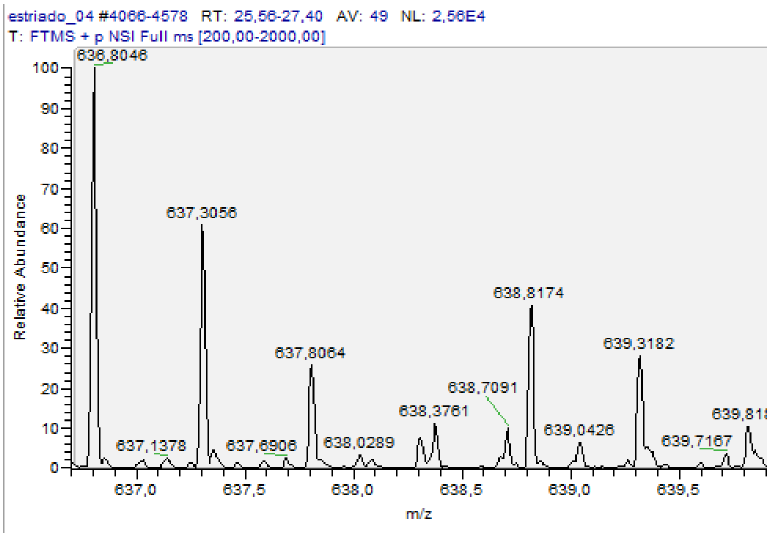

SANSNPA**M**APRE 2+
(methionine oxidation)

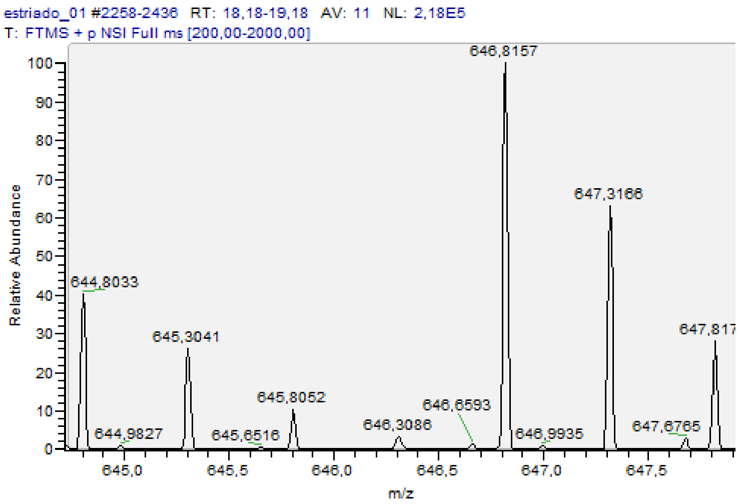

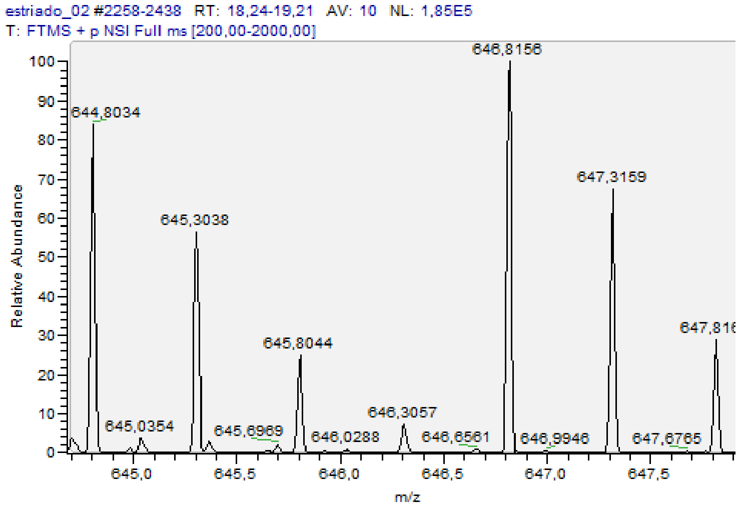

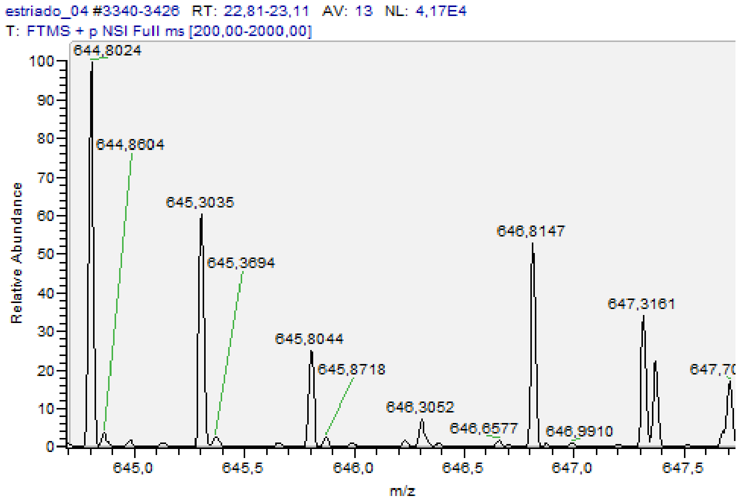

SFARAPQLDL 2+

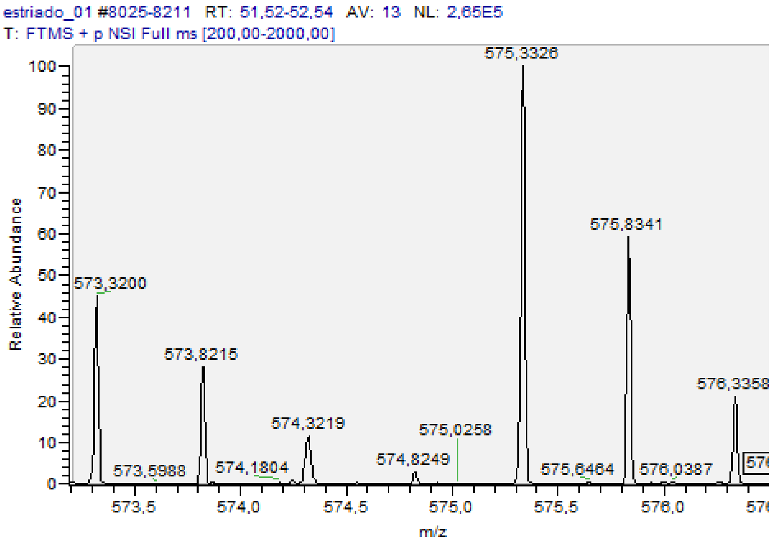

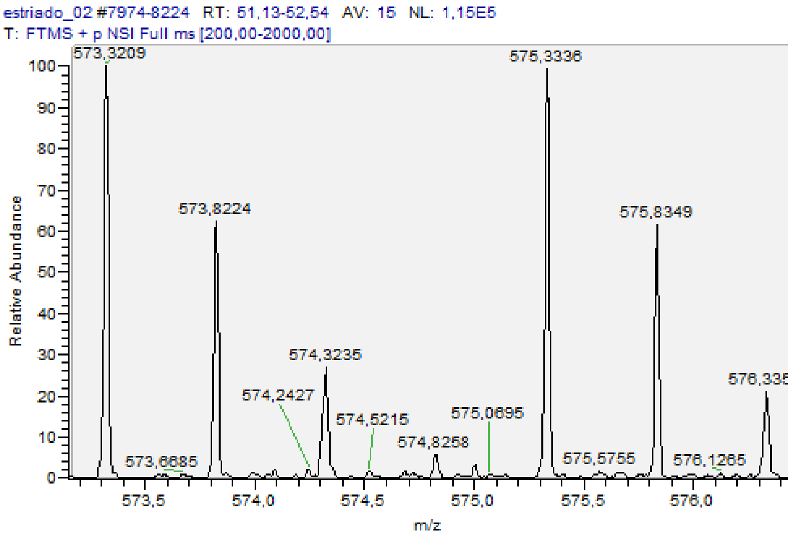

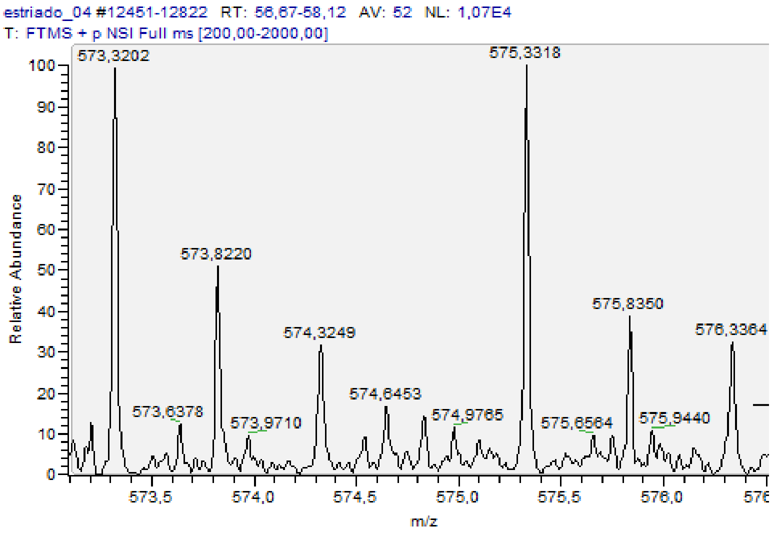

LLDEGHYPVRESPIDTA 2+

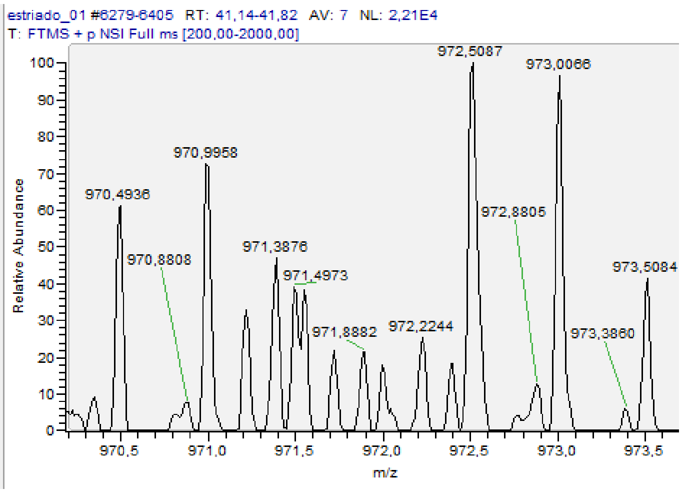

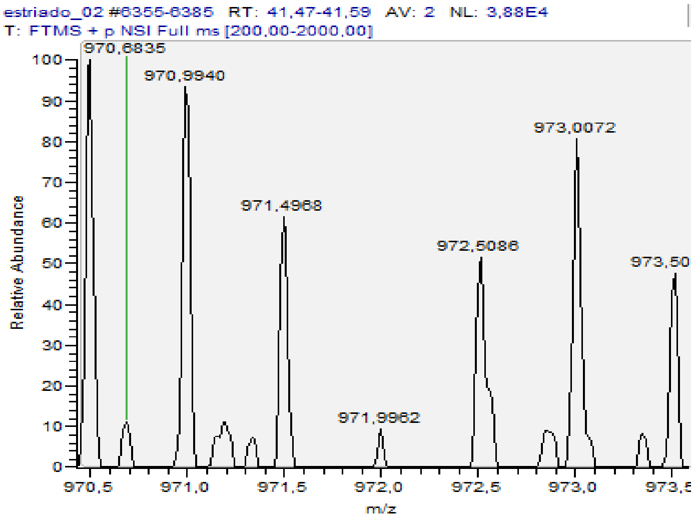

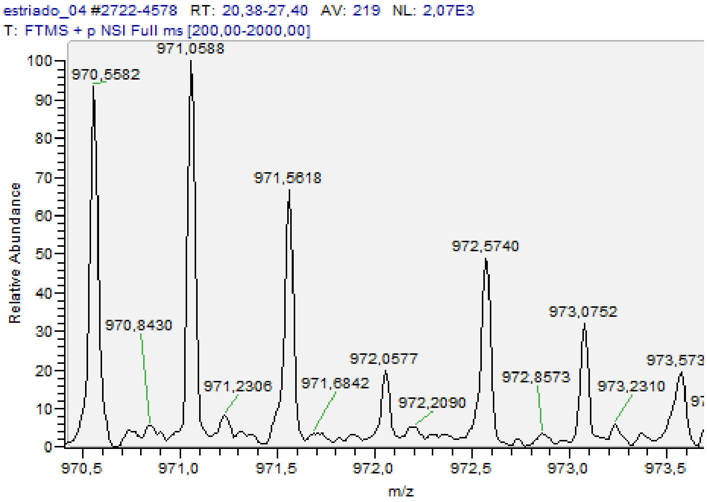

SPQLEDEAKELQ 2 +

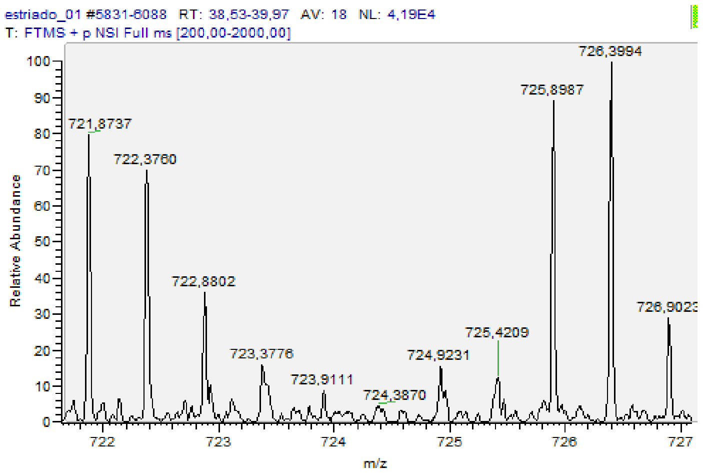

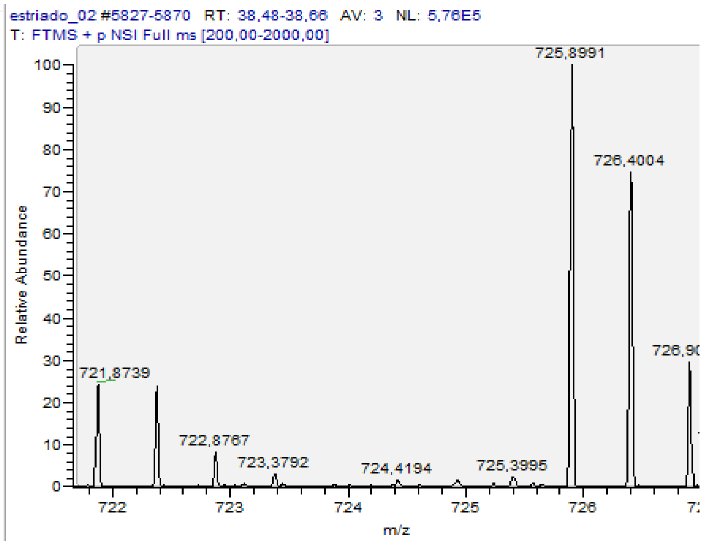

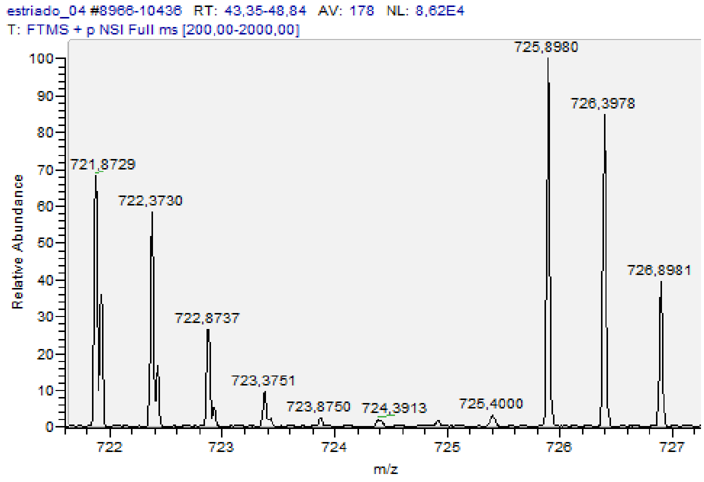

ADEVSASLAKQGL 2+

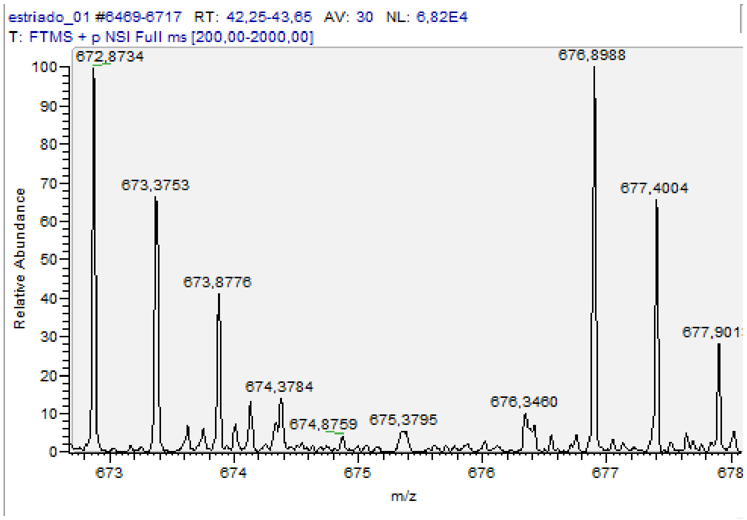

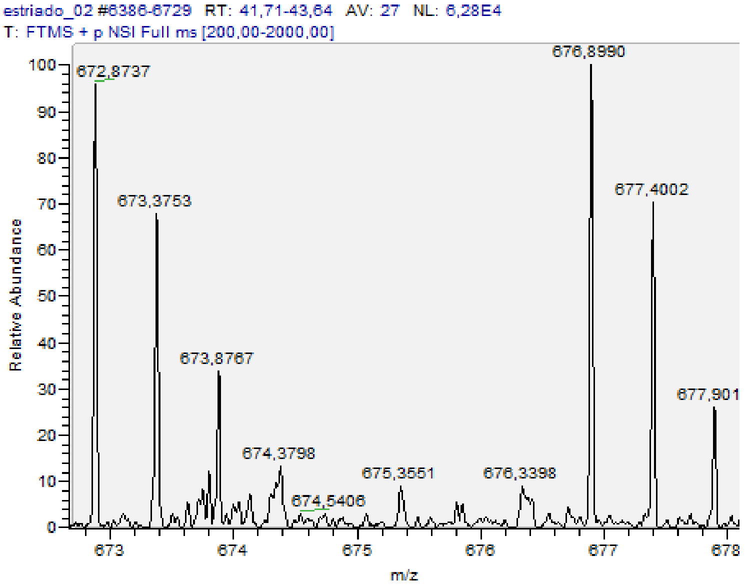

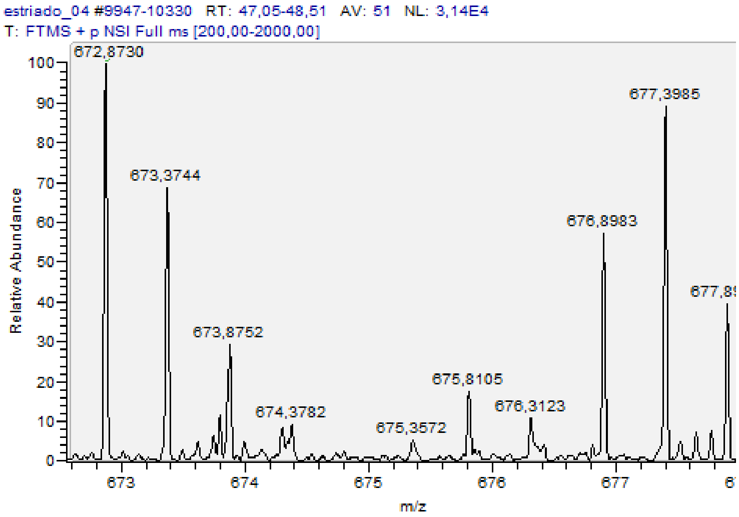

SGPFGQIFRPDNF 2+

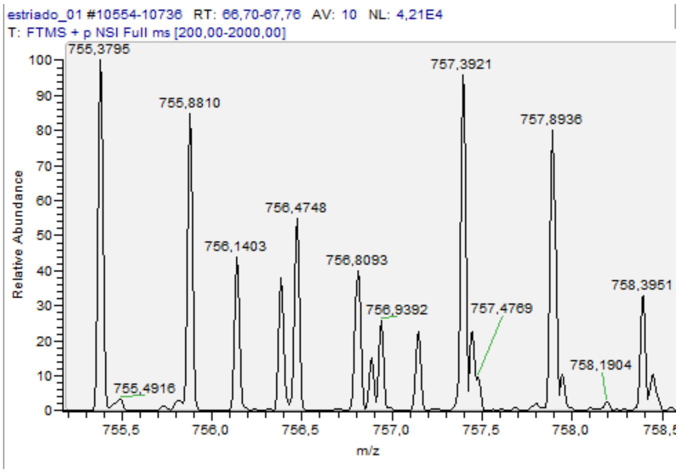

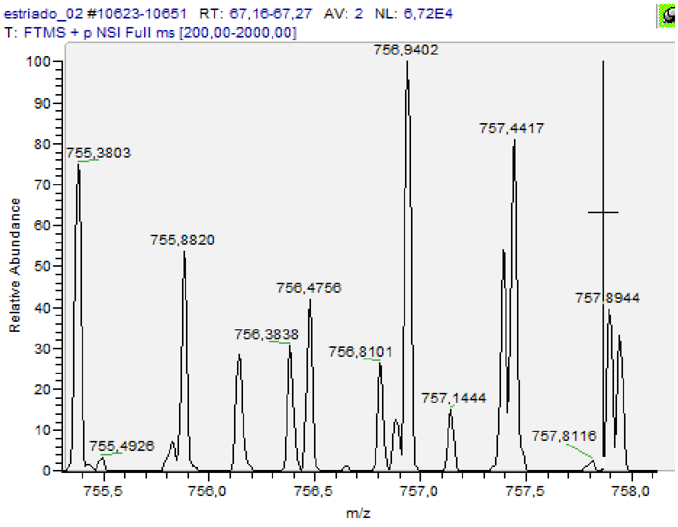

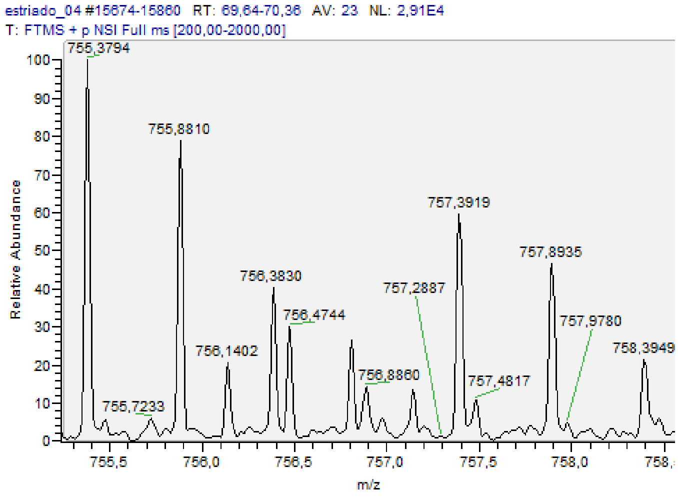

SGQAAARPLVATV 2+

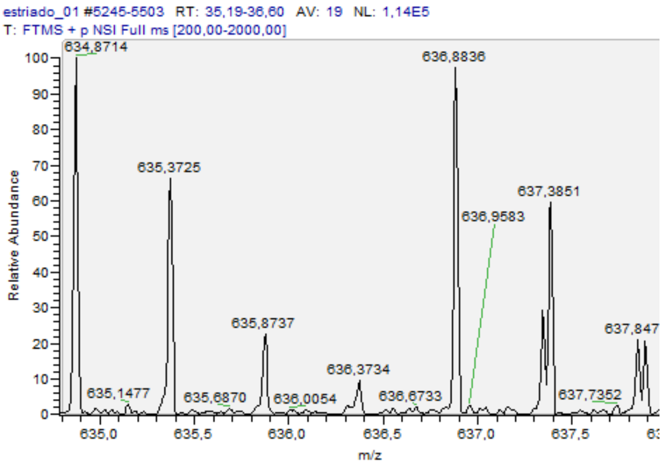

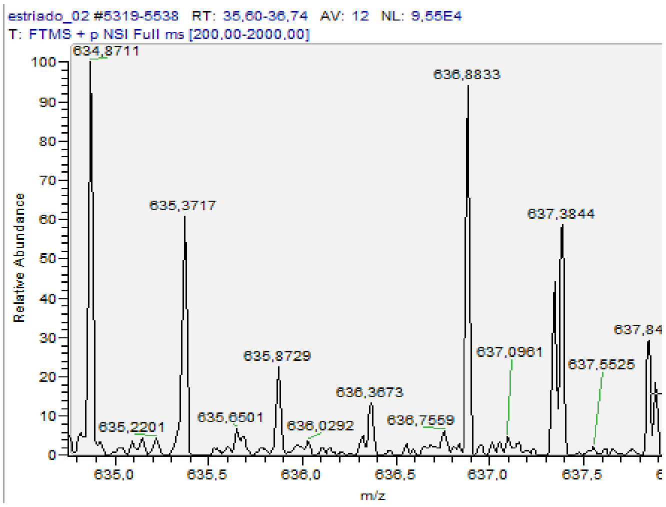

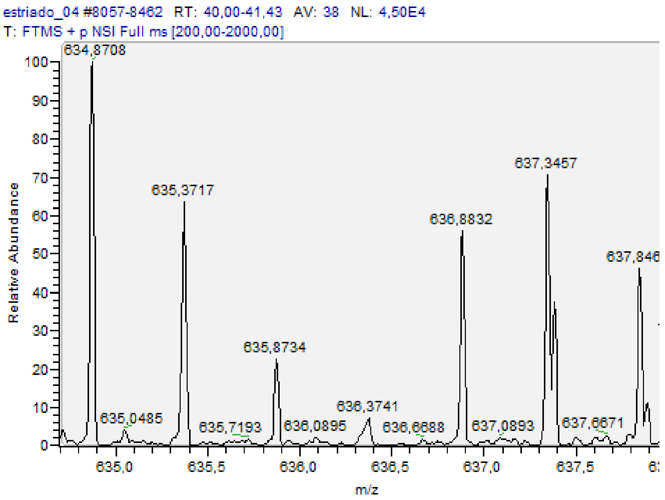

TVGLNVPASVRF 2+

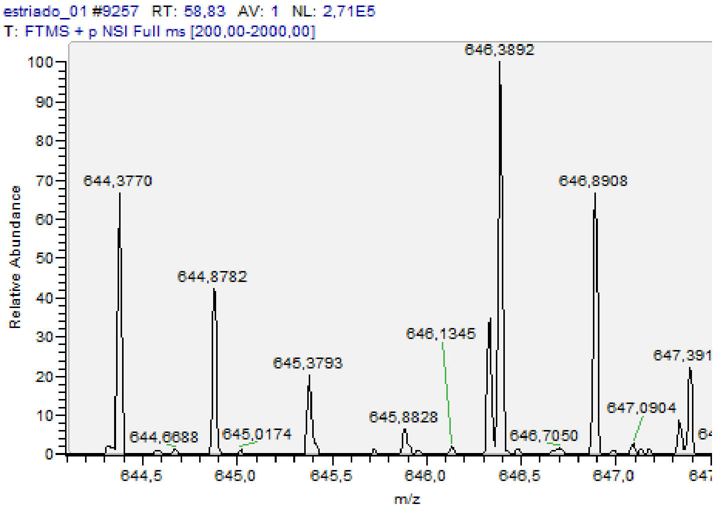

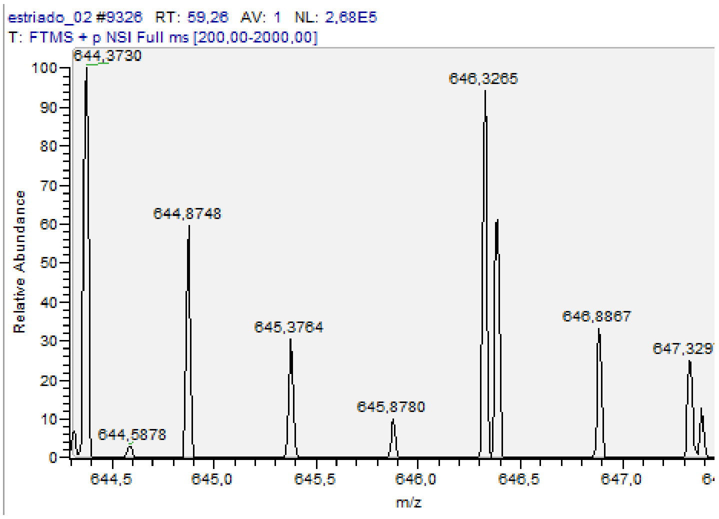

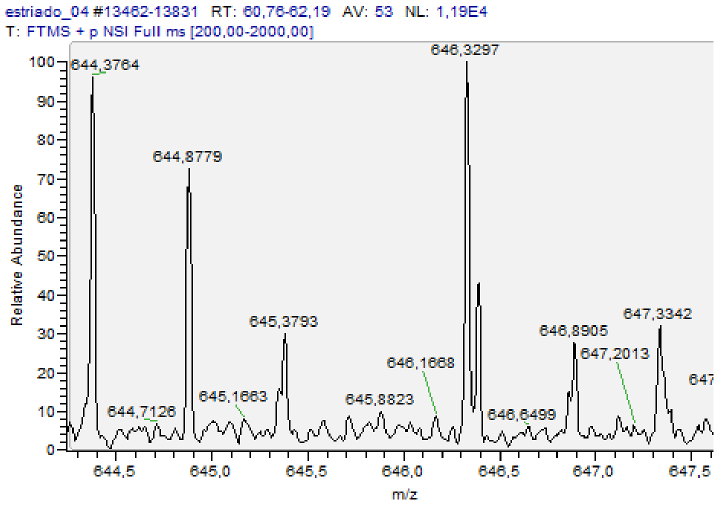

AQATGKPAQYIAVHVVPDQL

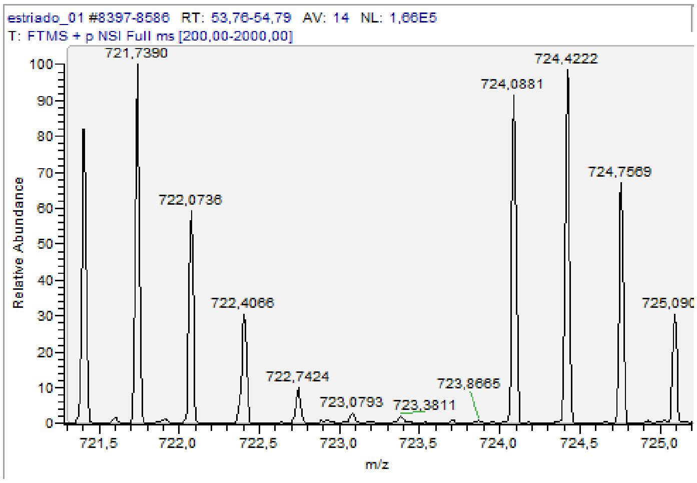

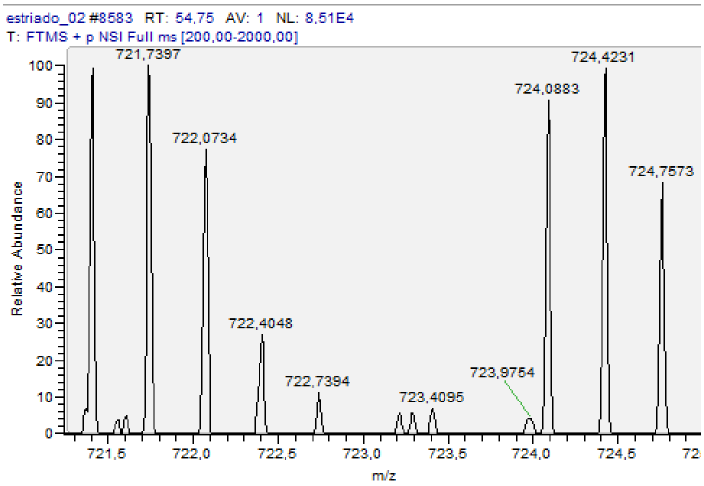

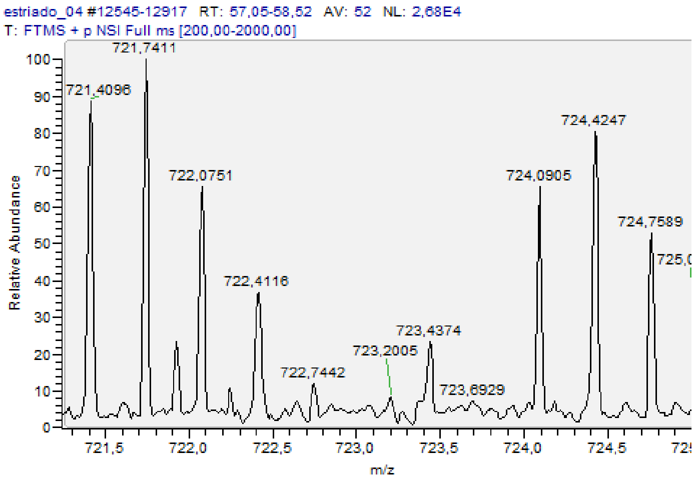

FHNPHVNPLPTGYEDE 2+

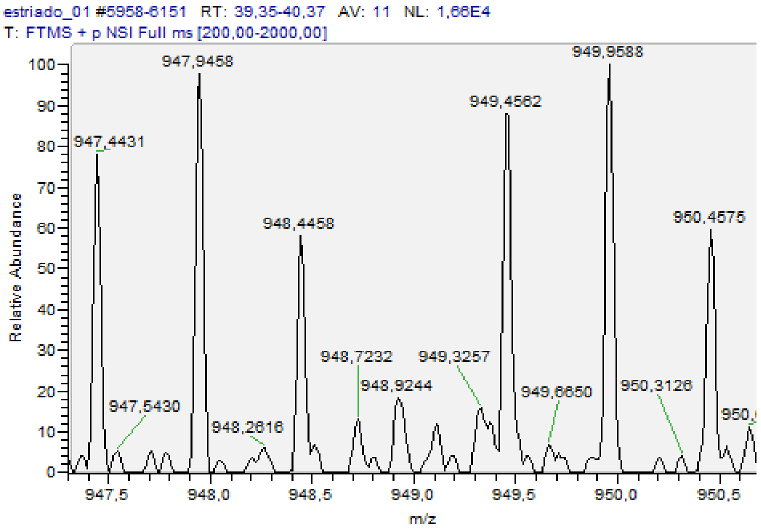

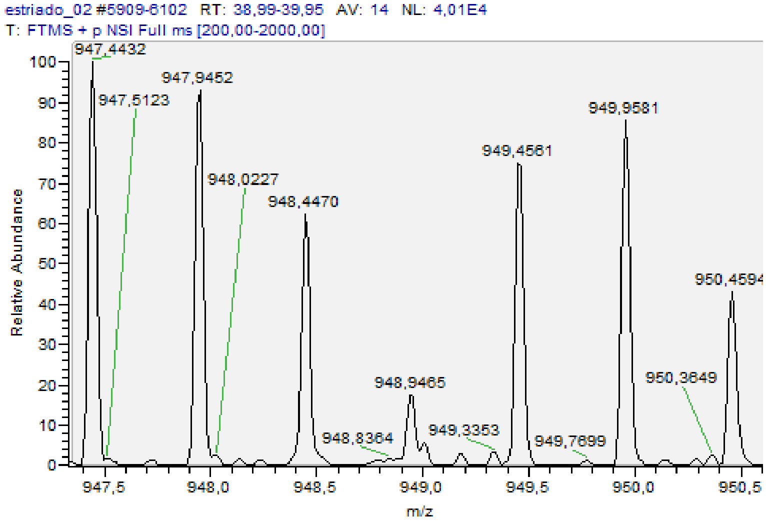

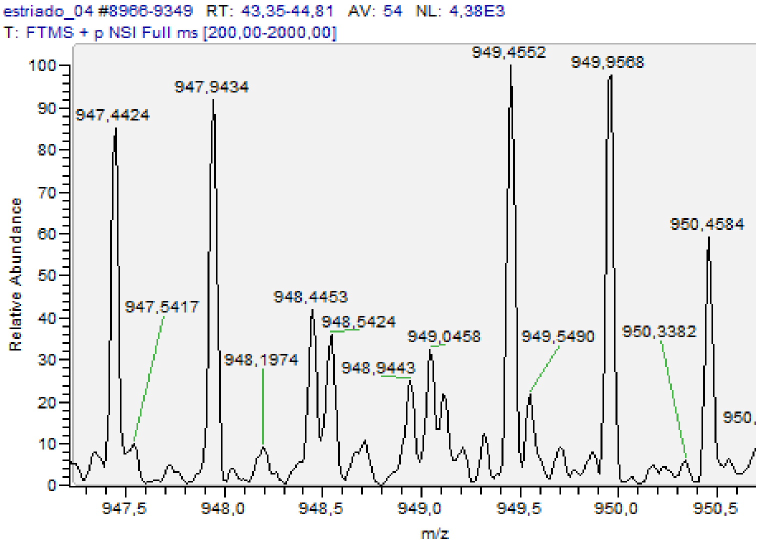

HNPHVNPLPTGYEDE 2+

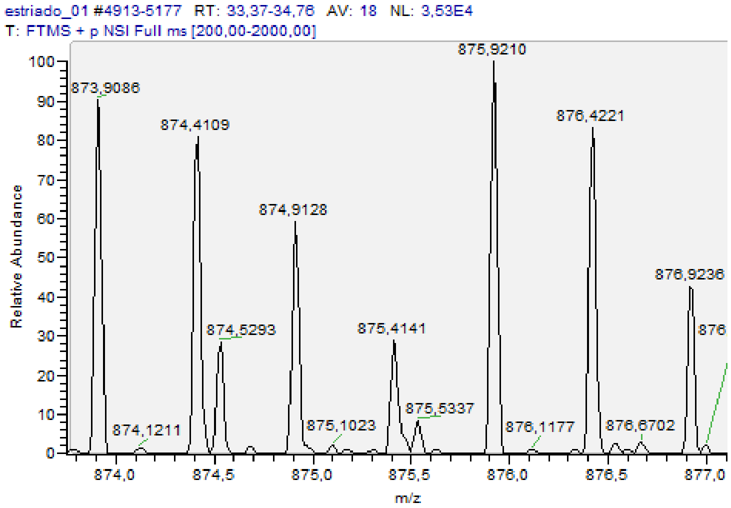

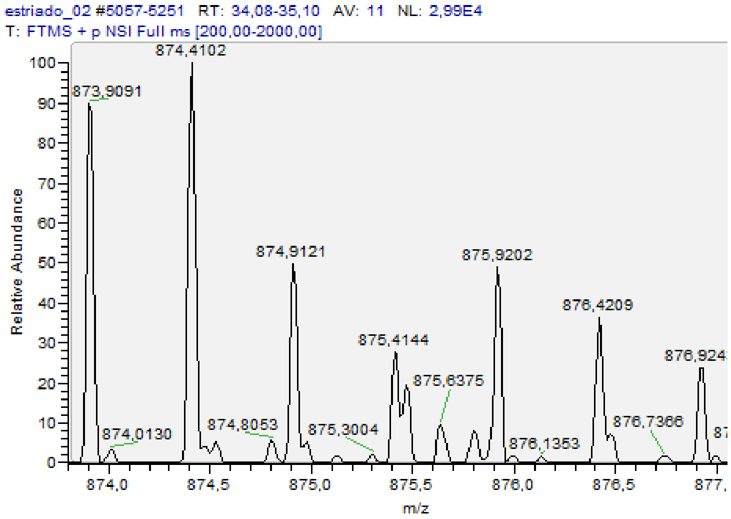

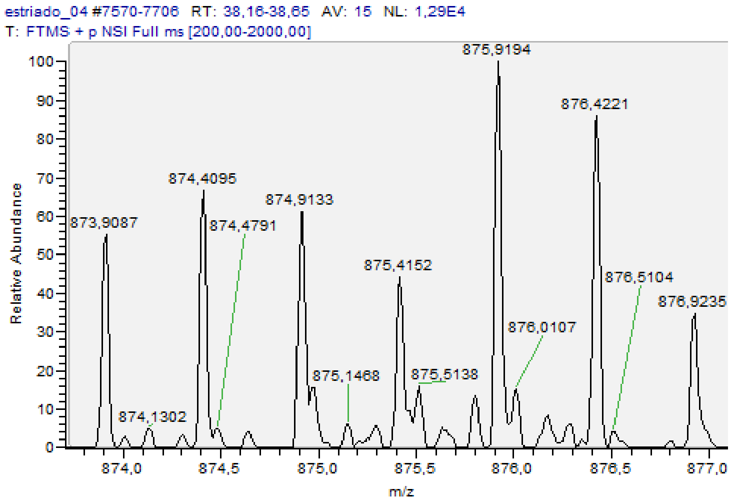

GISTPEELGLDKV 2+

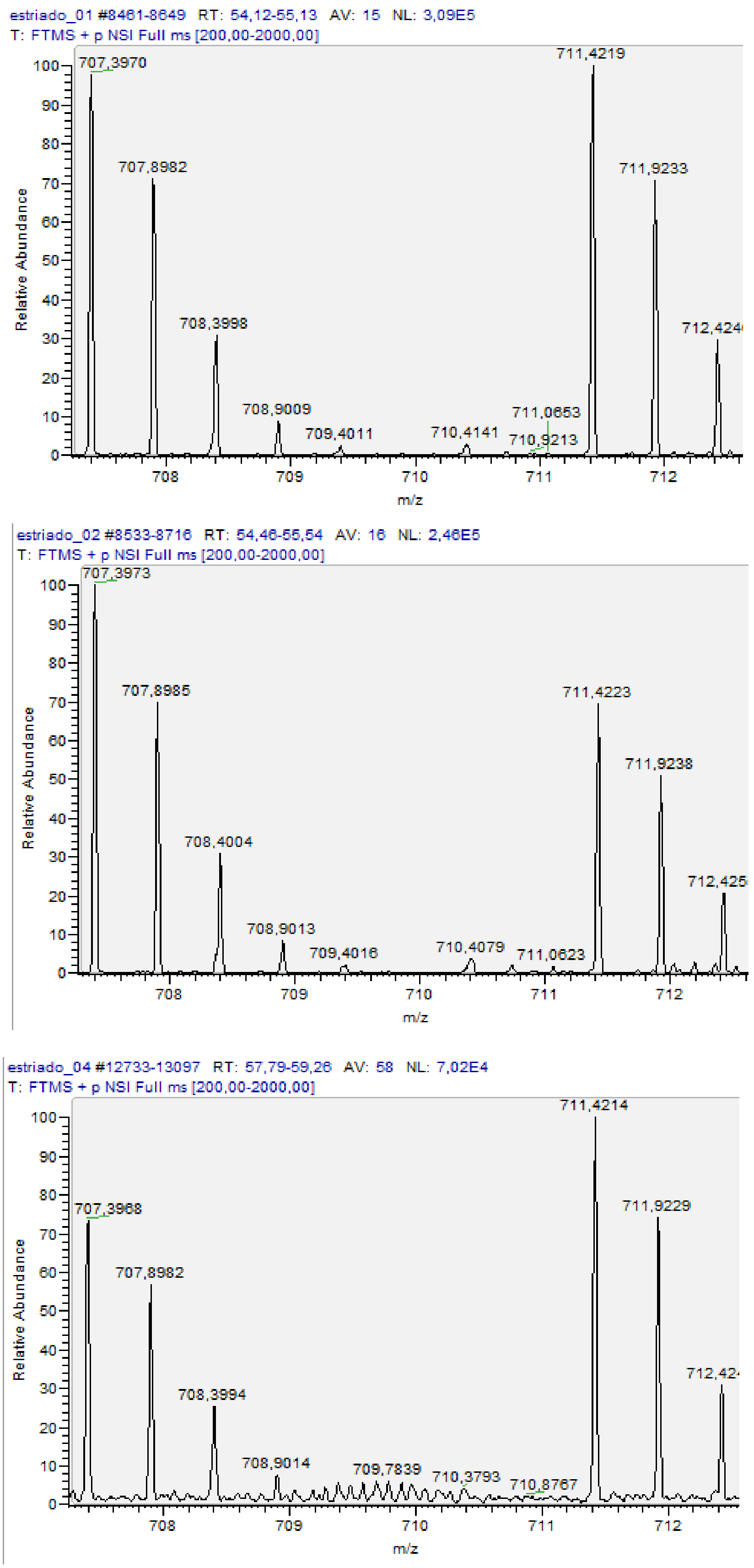

RPTLNELGISTPEELGLDKV 3+

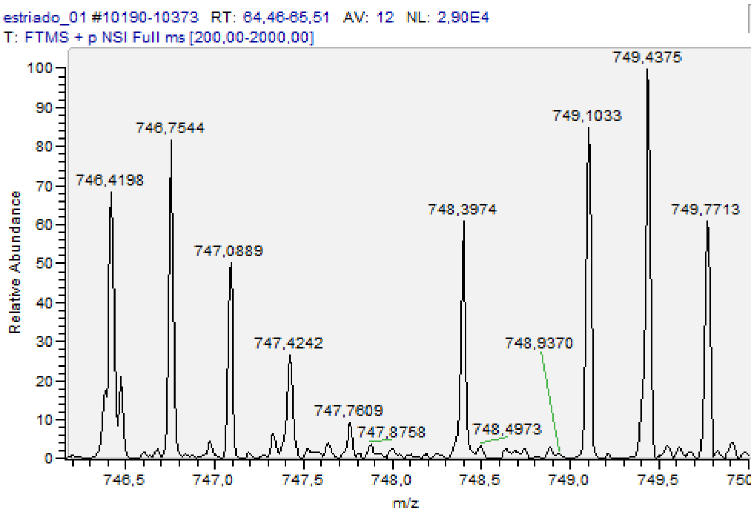

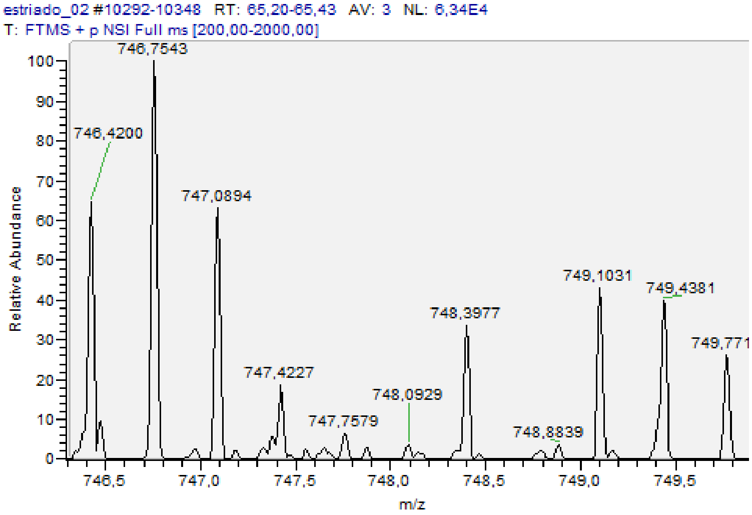

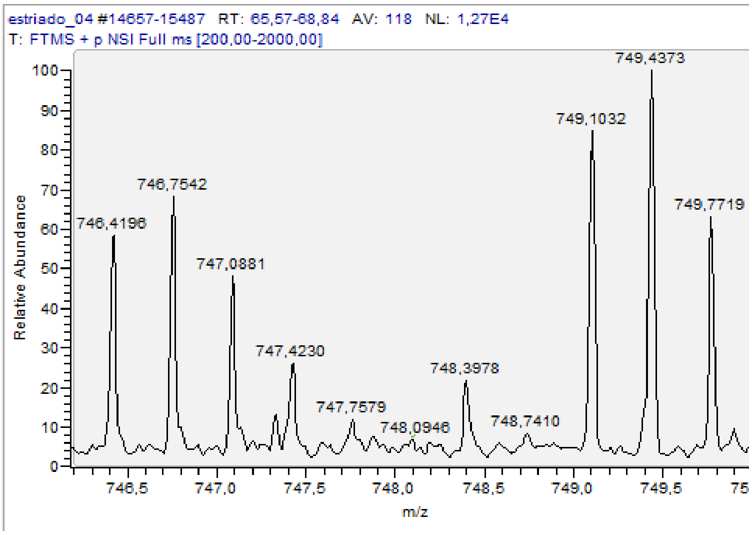

ASKGLGSDLDSSLASL 2+

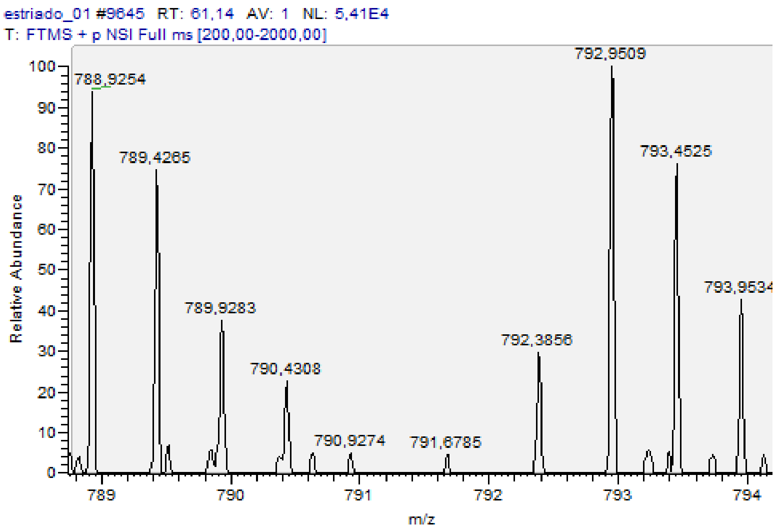

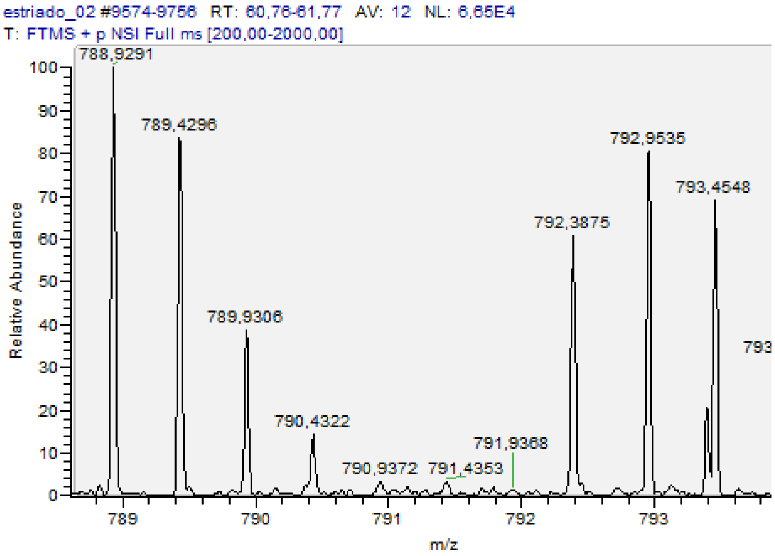

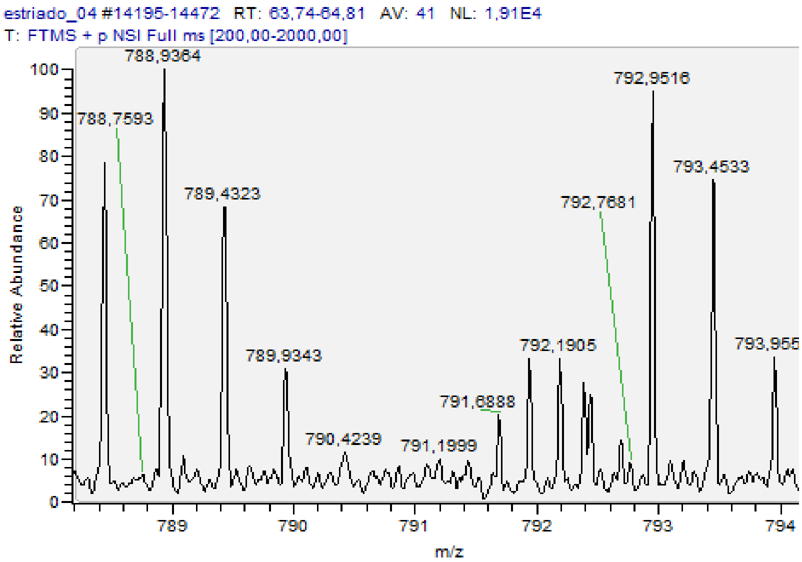

SPSPTPATQSPKKPPAKDPLADLNIKDFL 4+

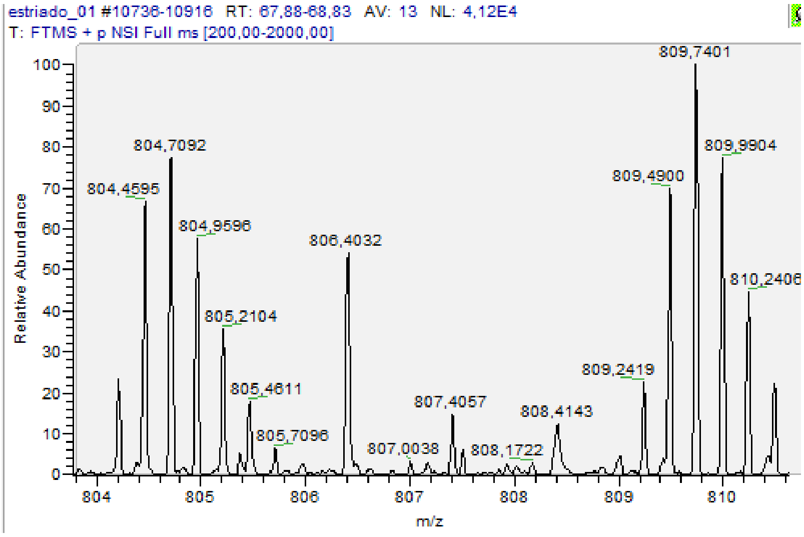

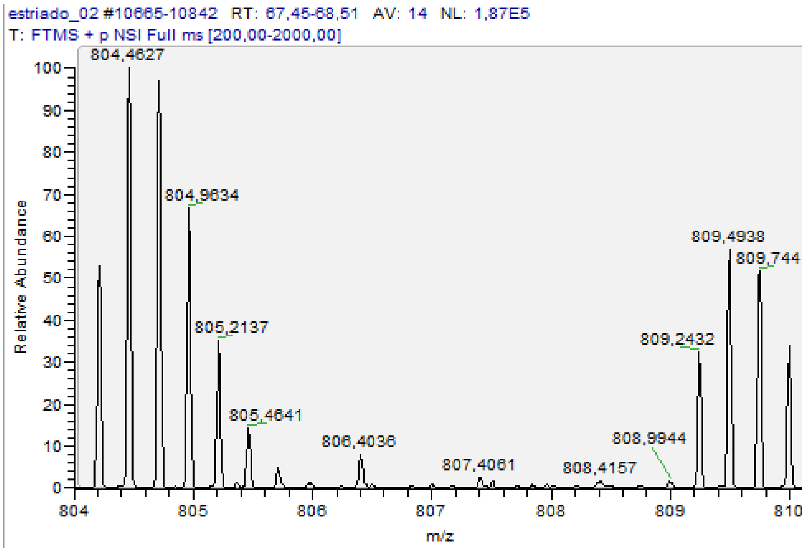

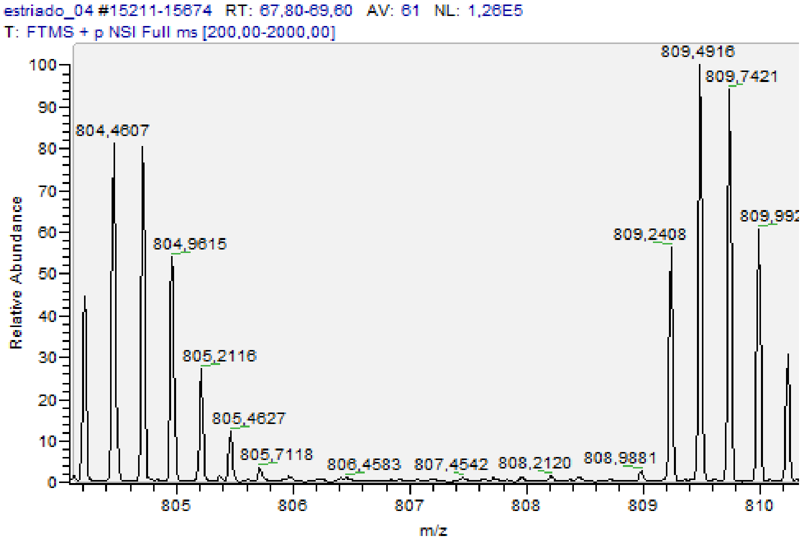

AVGSGGKGKSGEIEPVSV

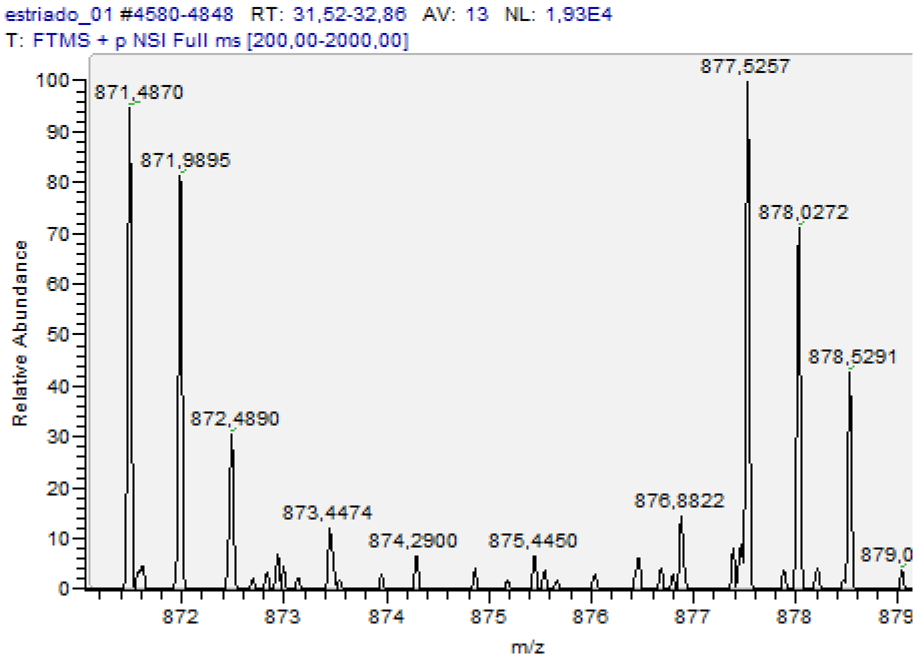

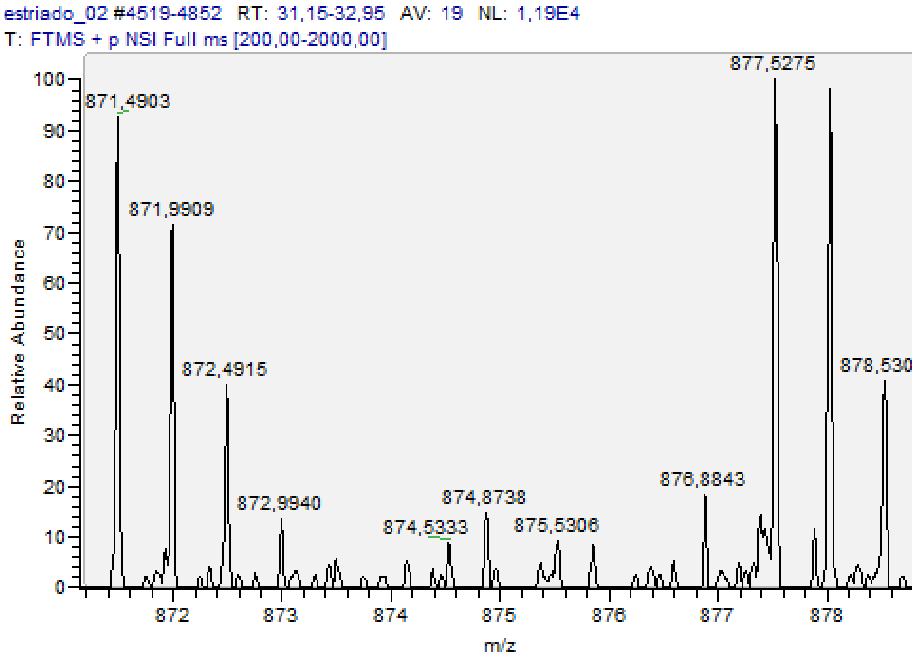

AGGPPHPQLNKS

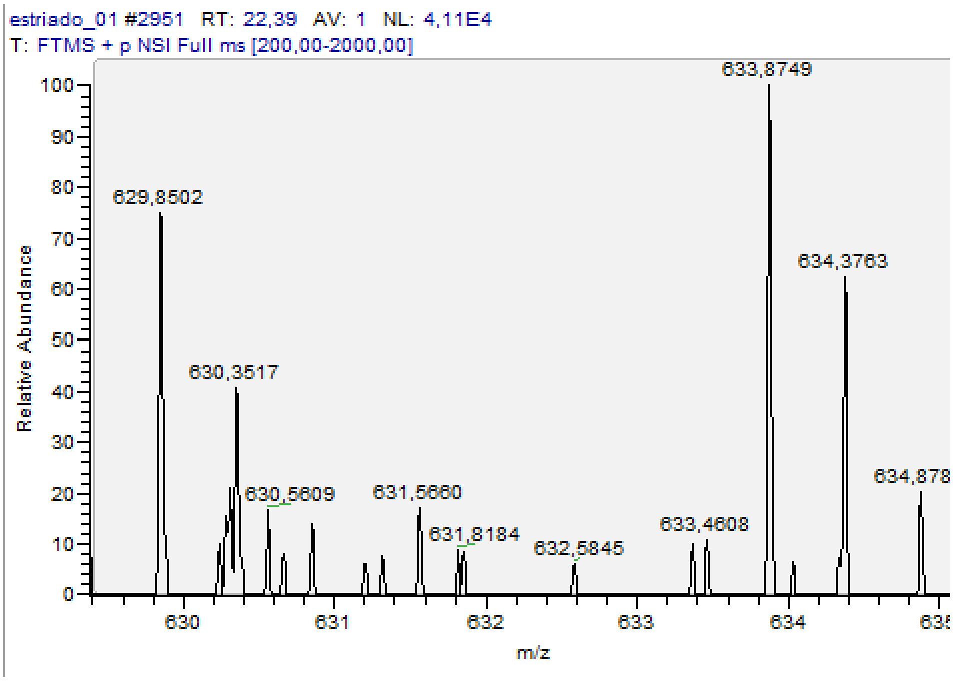

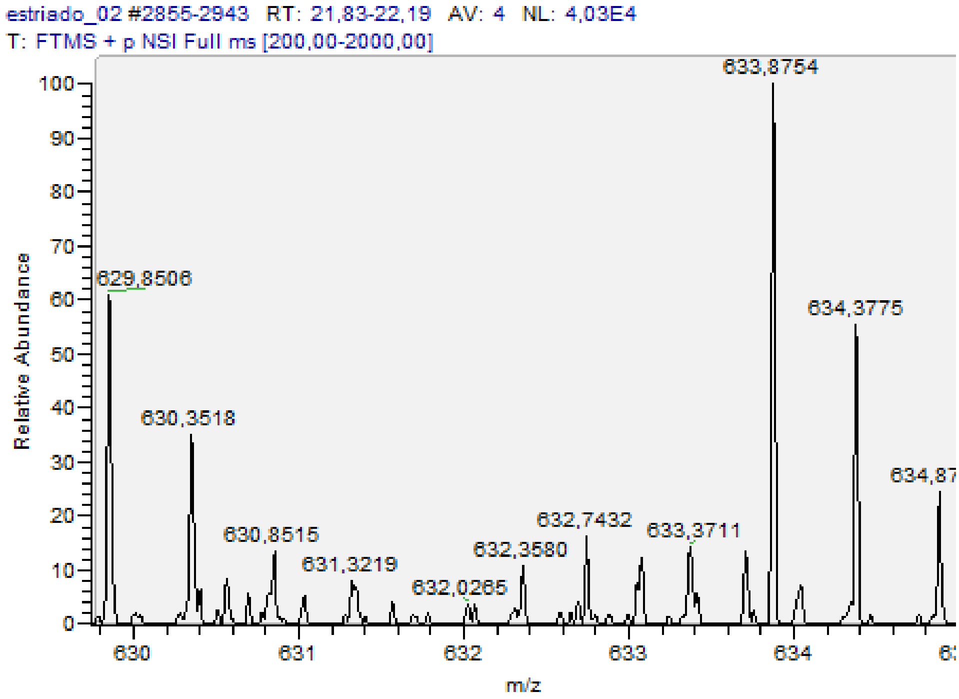

GQGAGALVHSE 2+

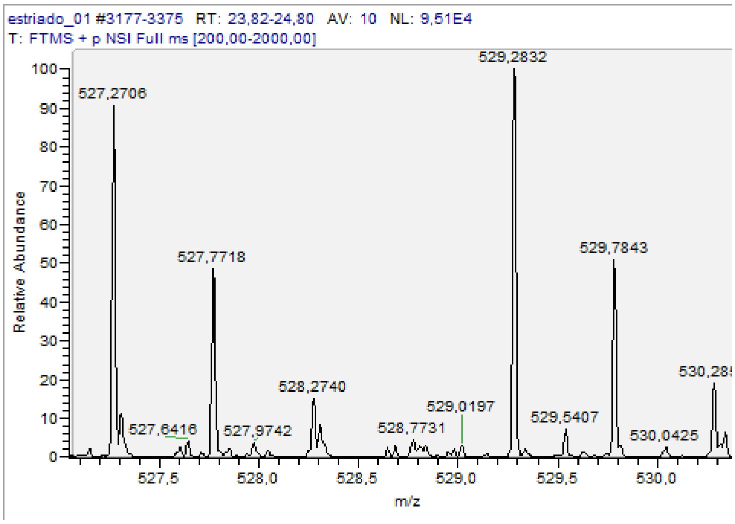

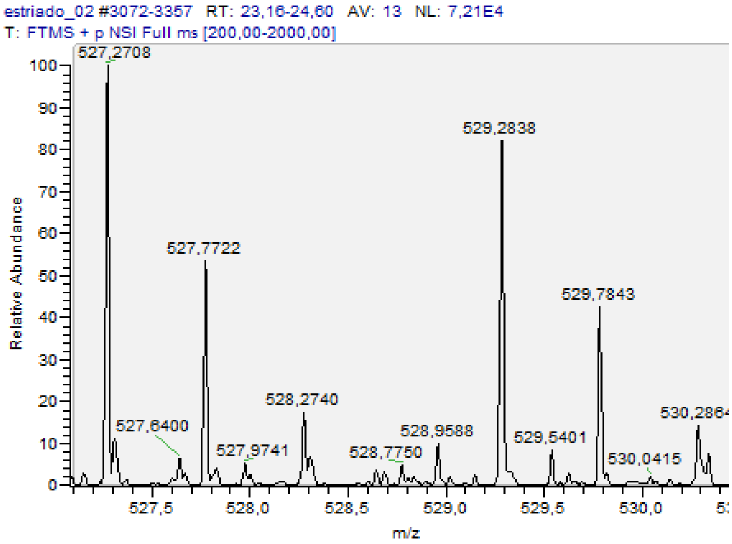

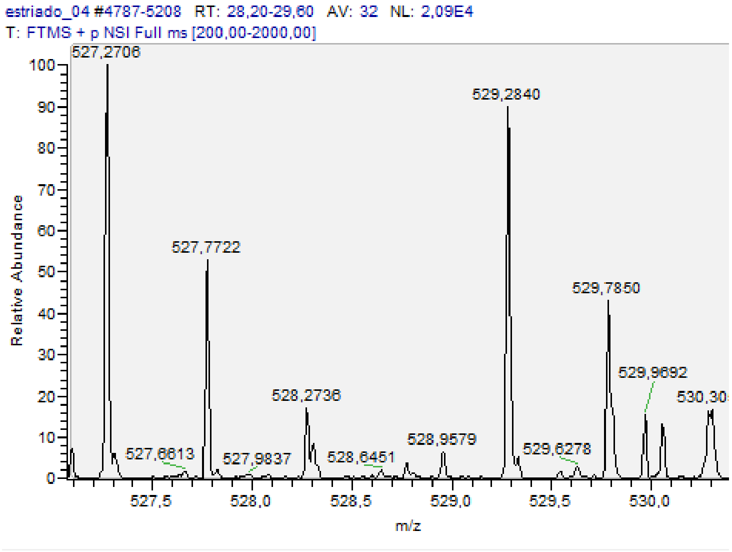

AFRVPTPNVSVVDL 2+

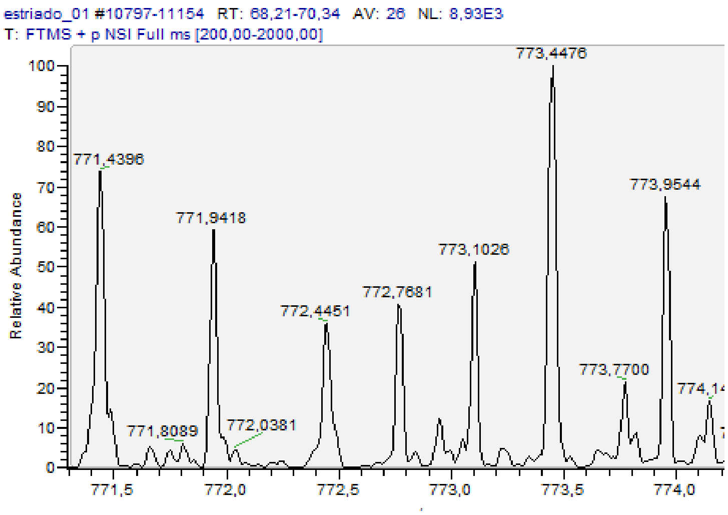

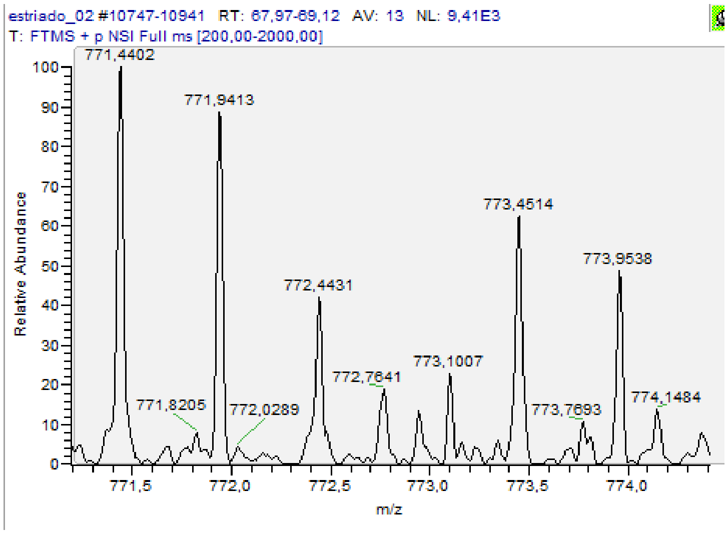

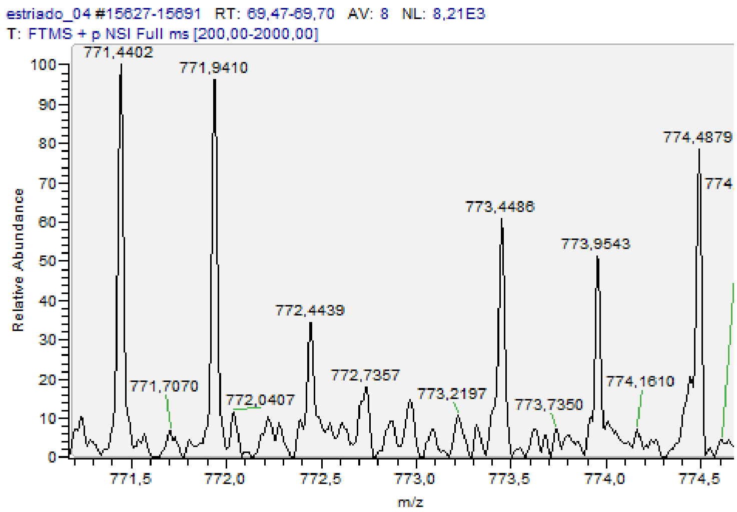

GRKGPGPGGPGGAGGARGGAGGGPSGD 3+

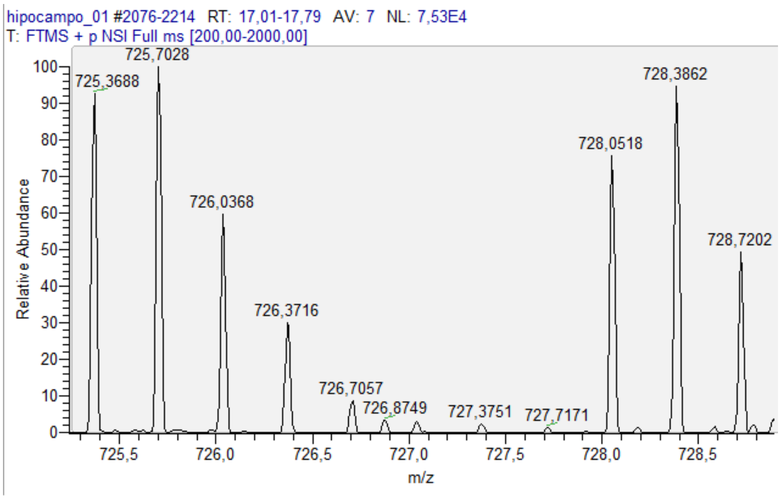

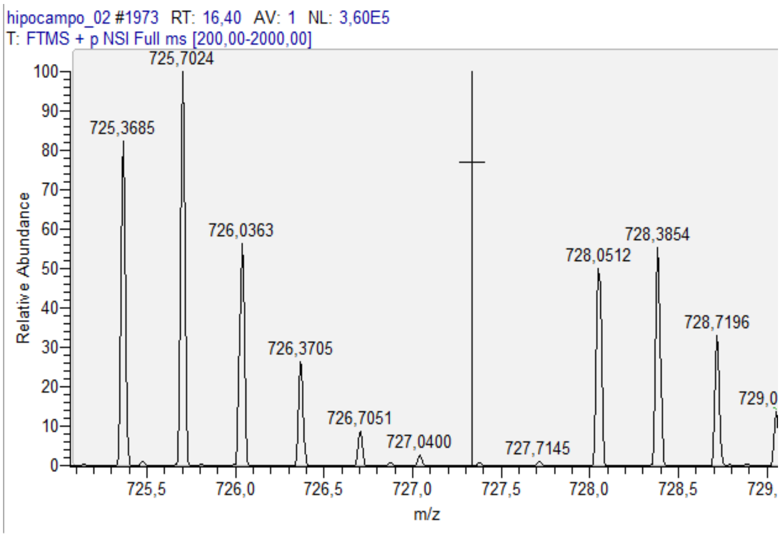

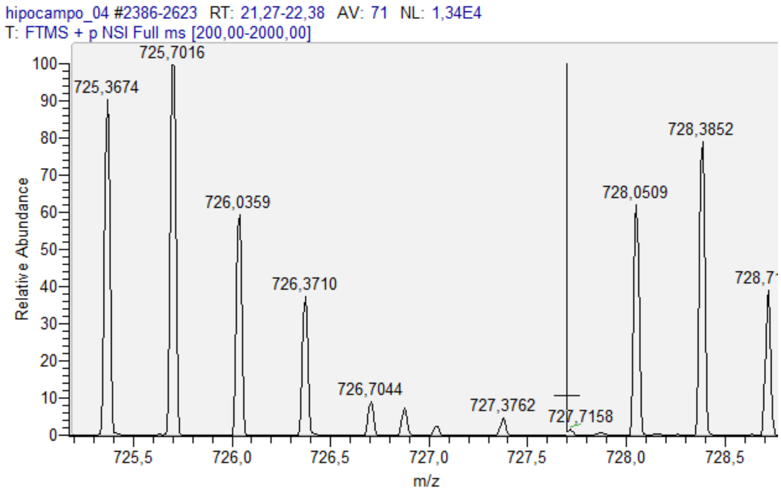

KGPGPGGPGGAGGARGGAGGGPSGD 2+

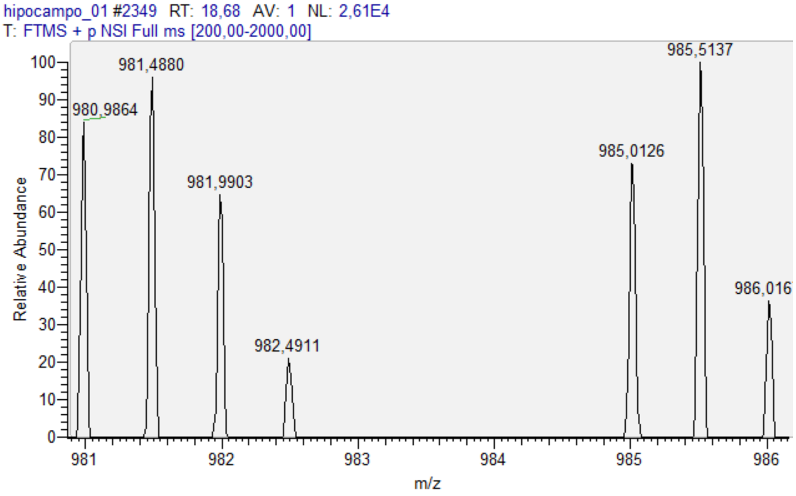

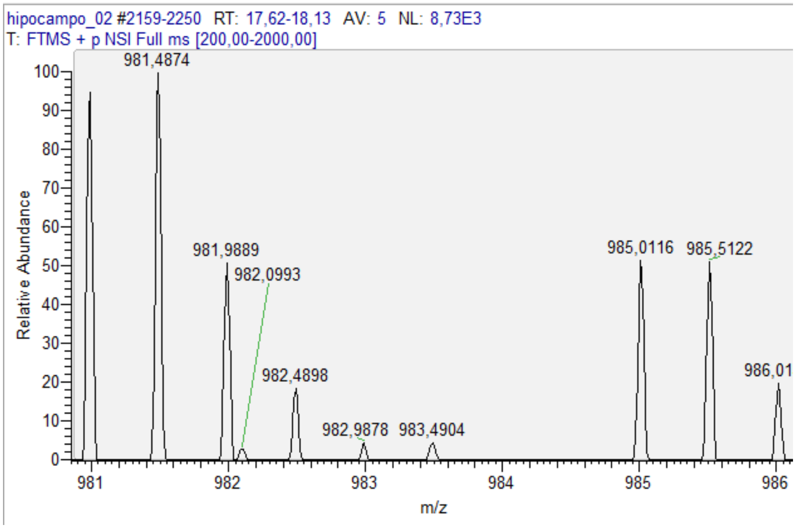

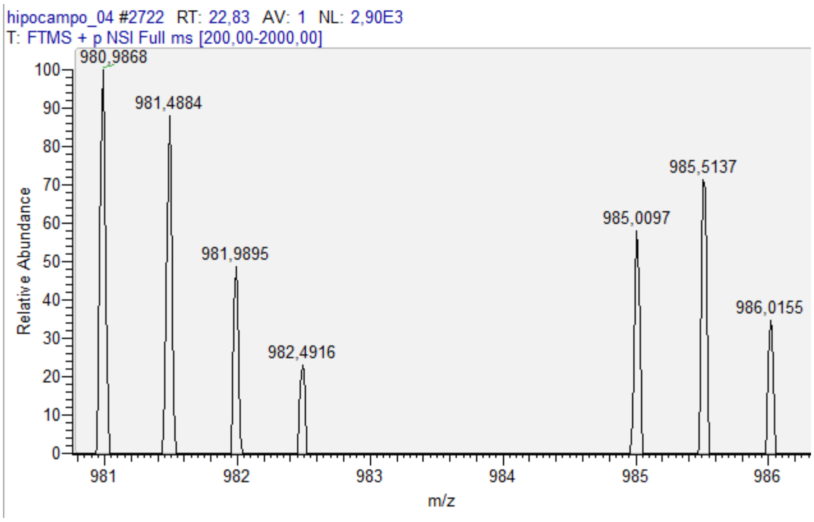

KGPGPGGPGGAGGARGGAGGGPSGD 3+

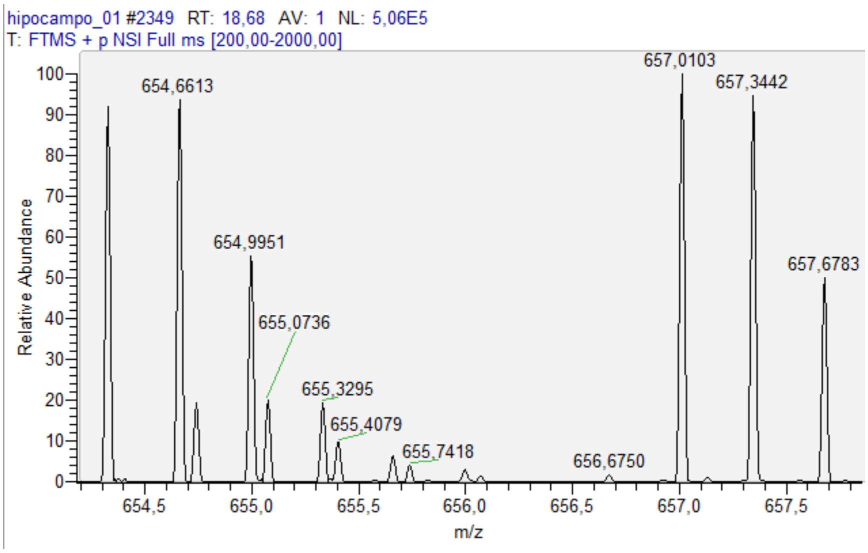

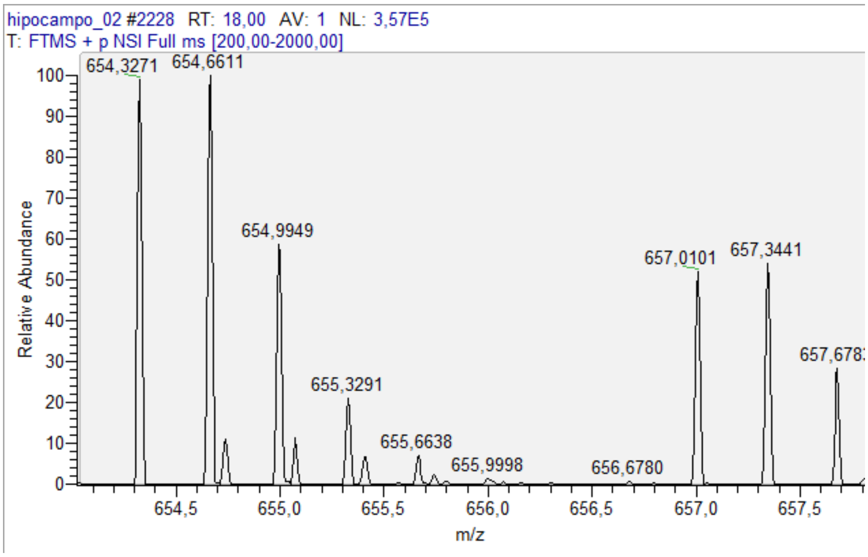

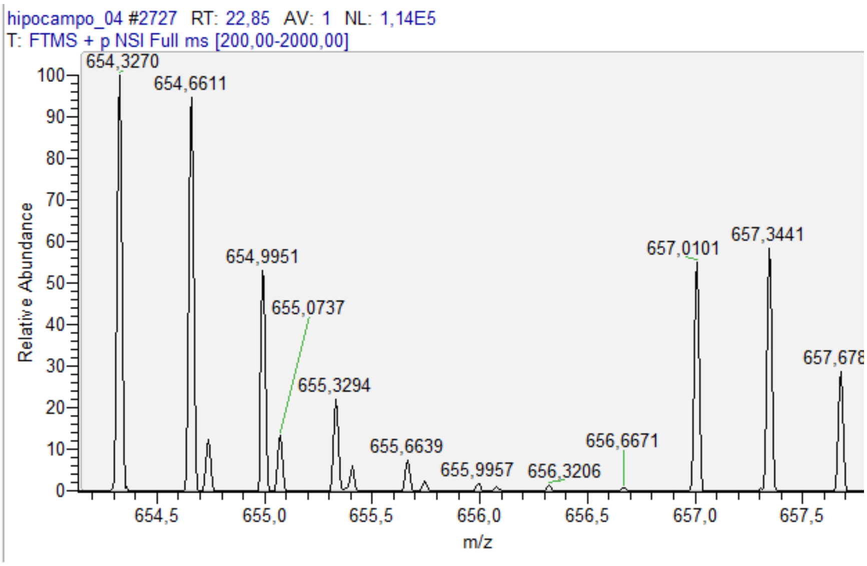

RKGPGPGGPGGAGGARGGAGGGPSGD 3+

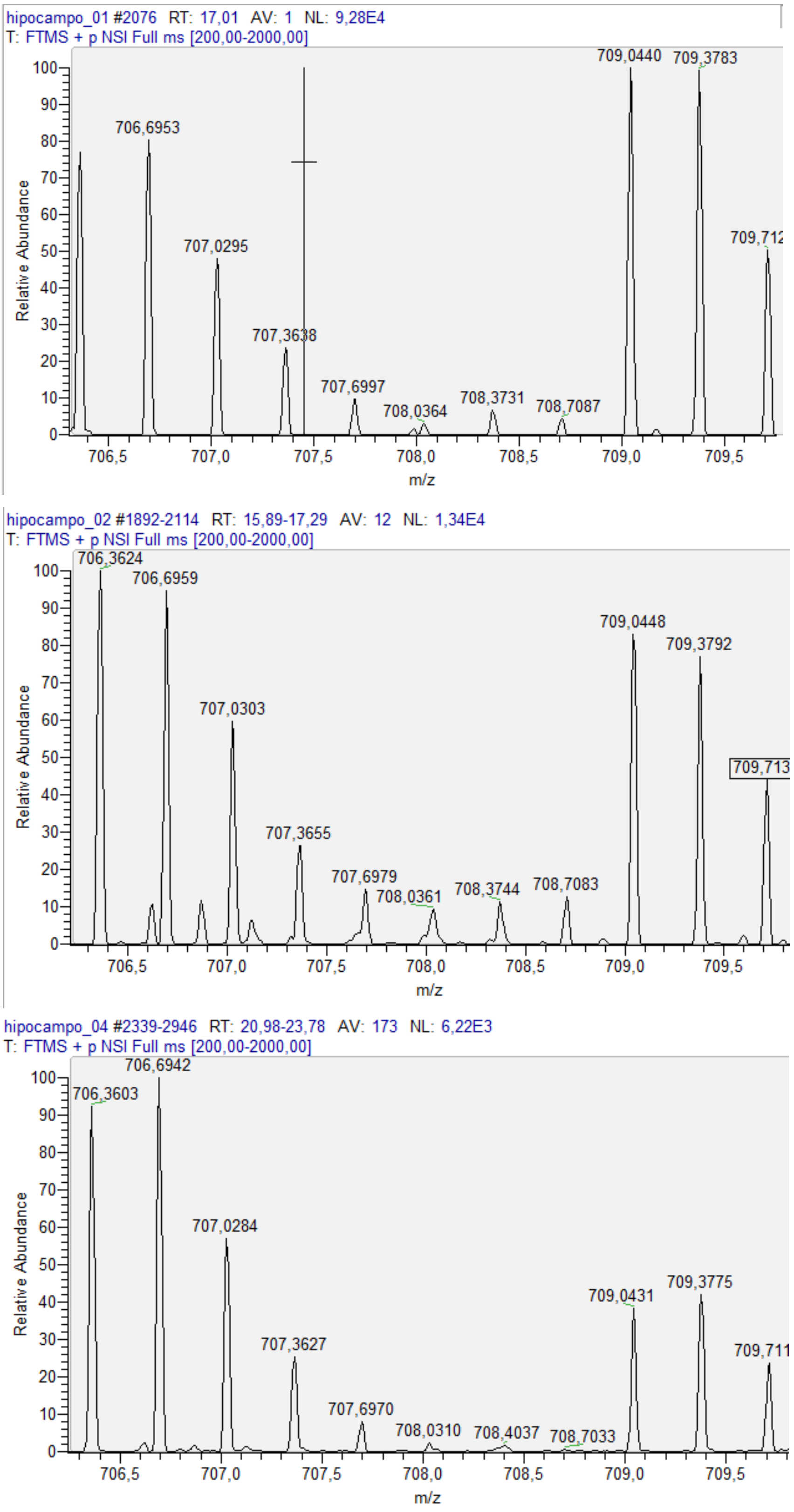

ATVGDVNTDRPGLLDL 2+

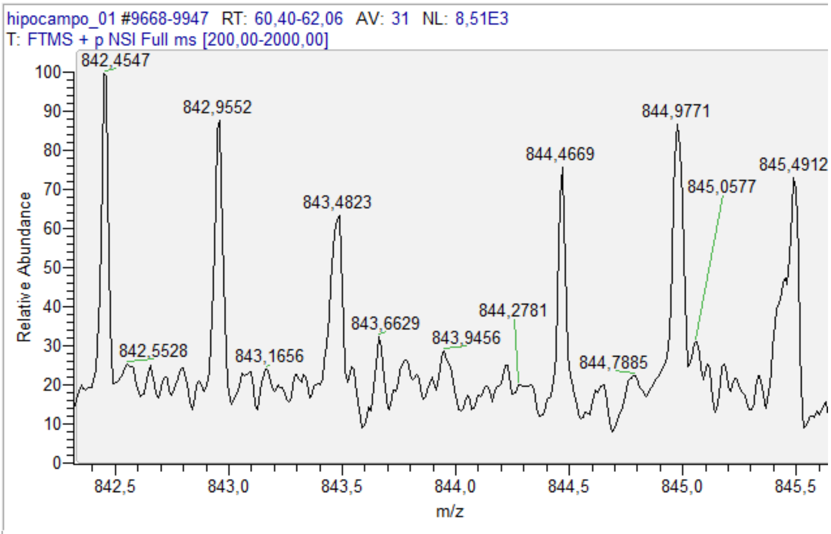

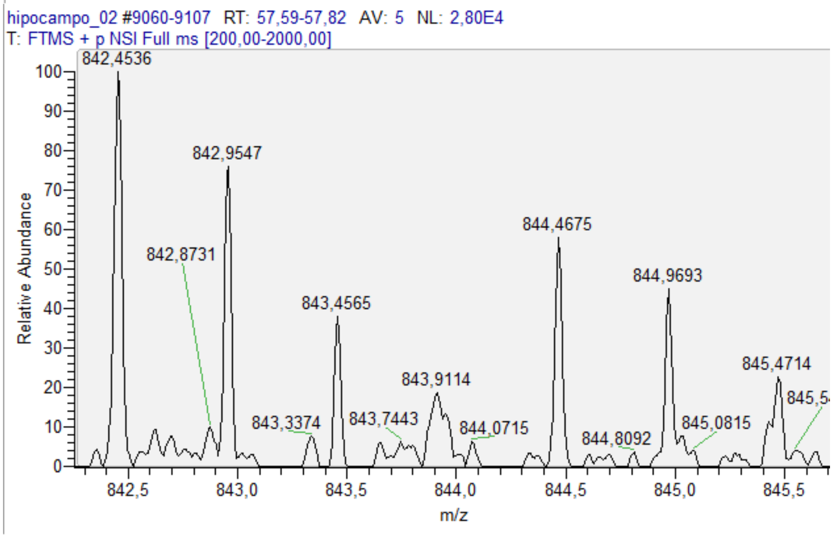

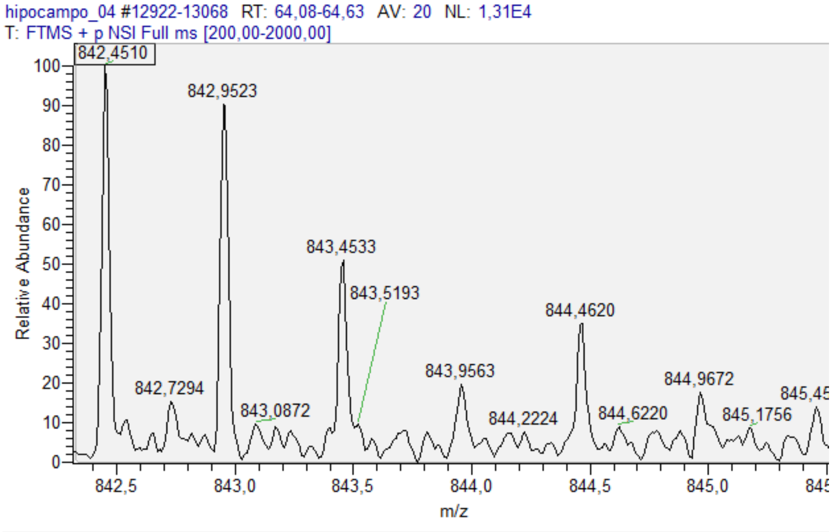

GDVNTDRPGLLDL 2+

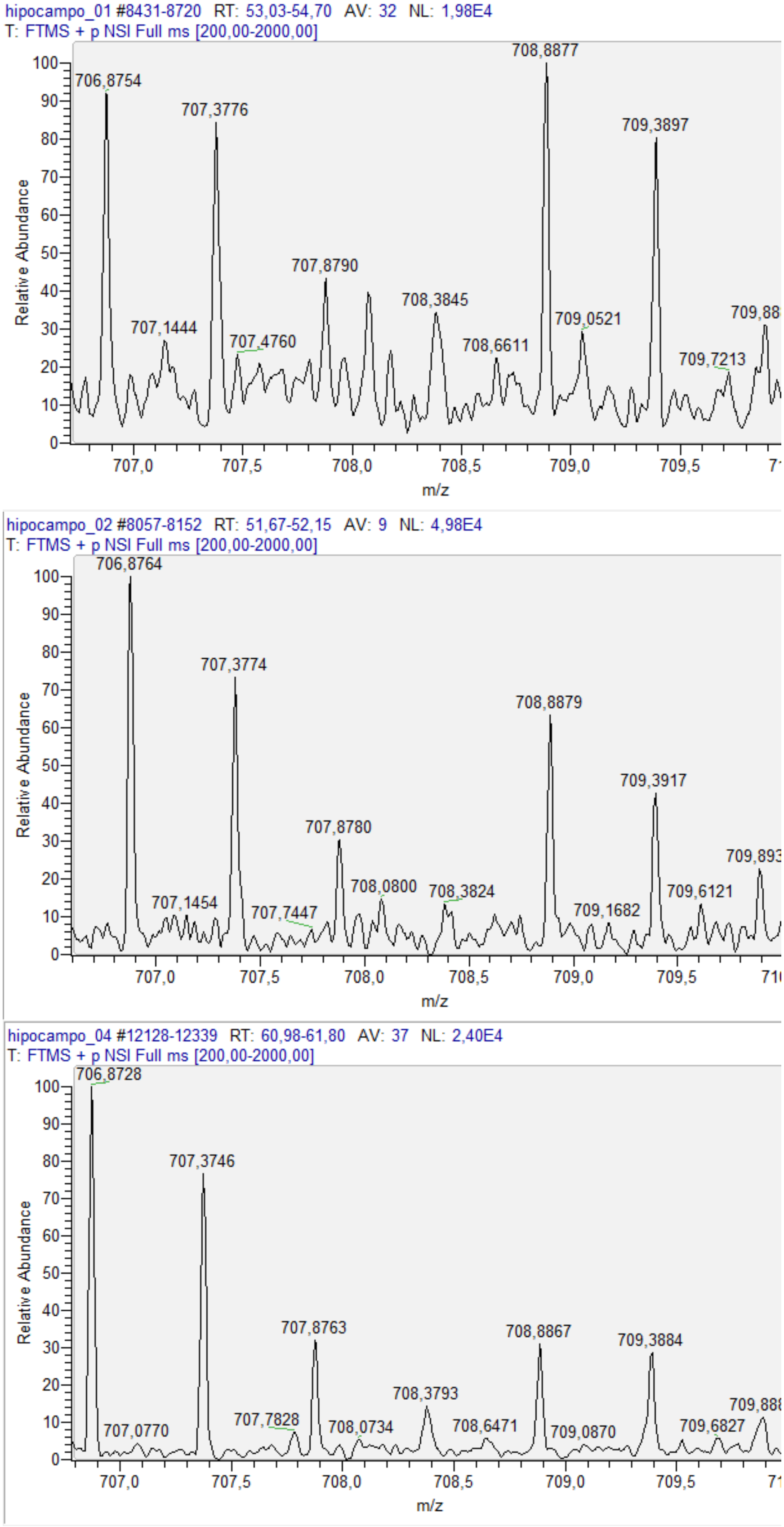

KQATVGDVNTDRPGLLDL 2+

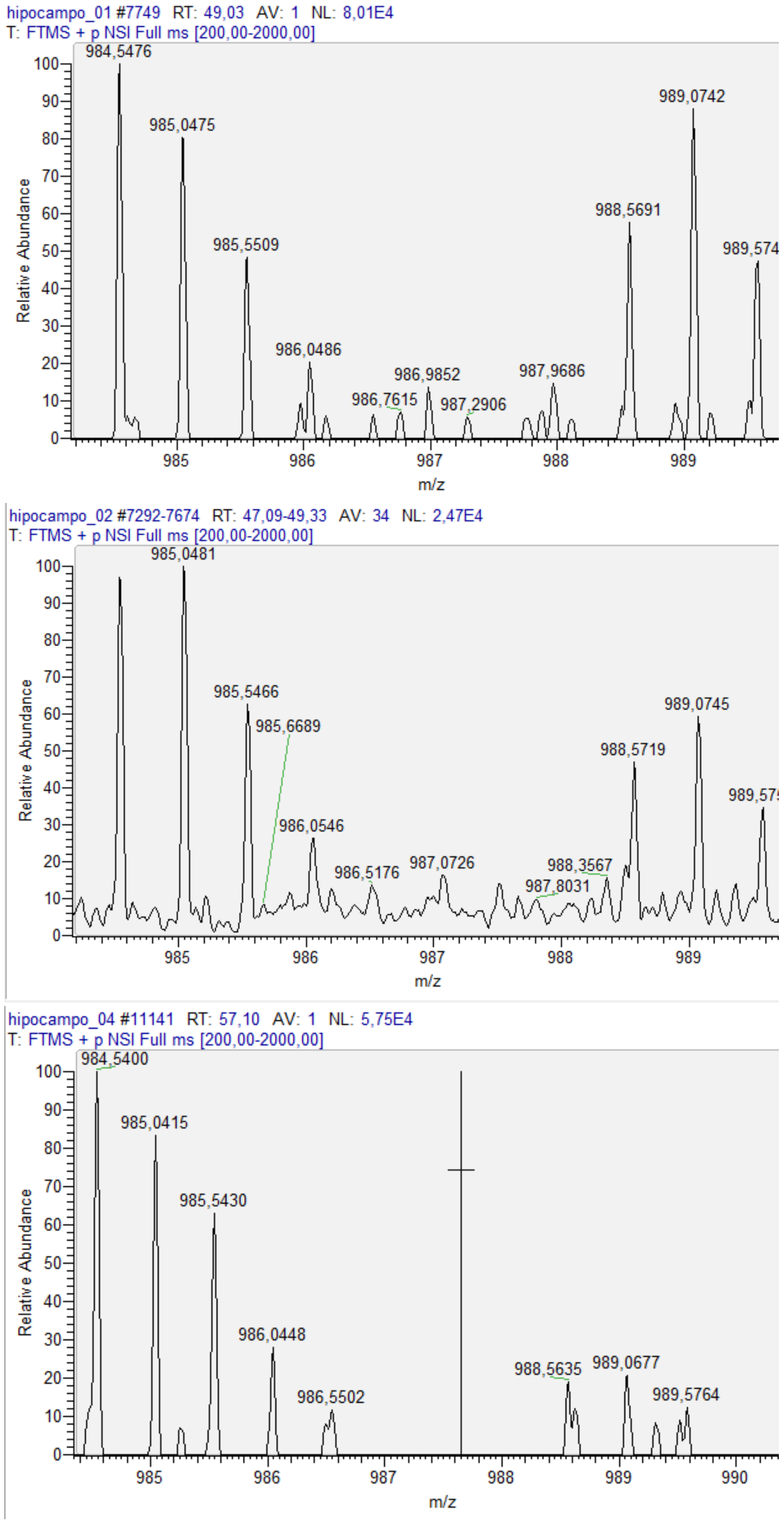

KQATVGDVNTDRPGLLDL 3+

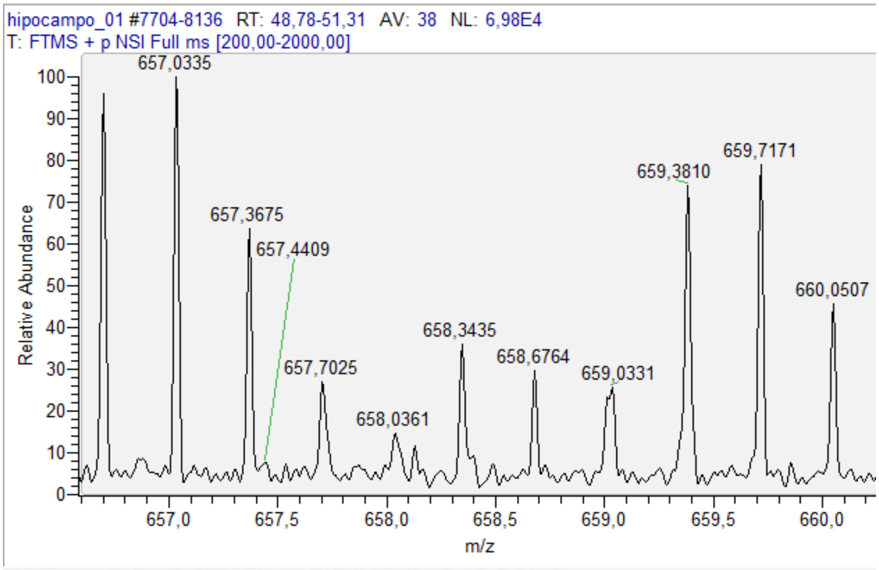

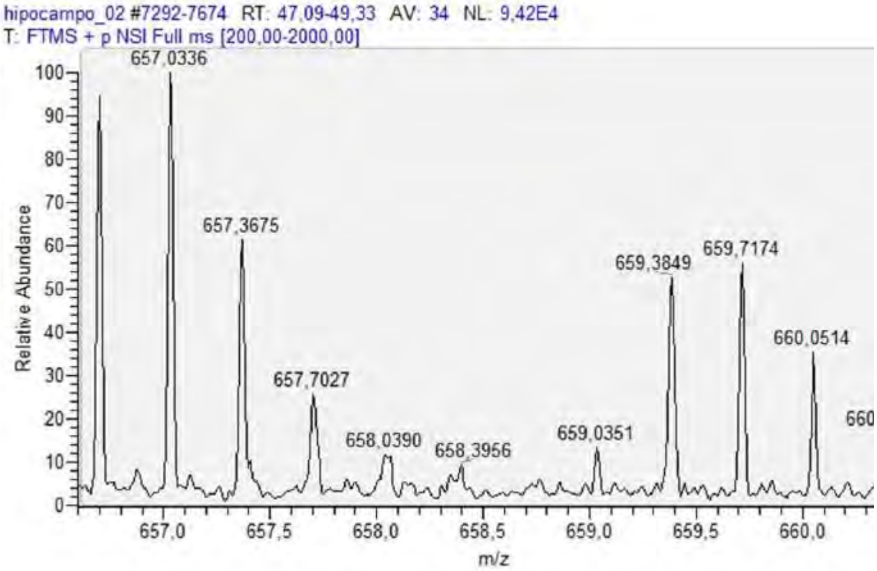

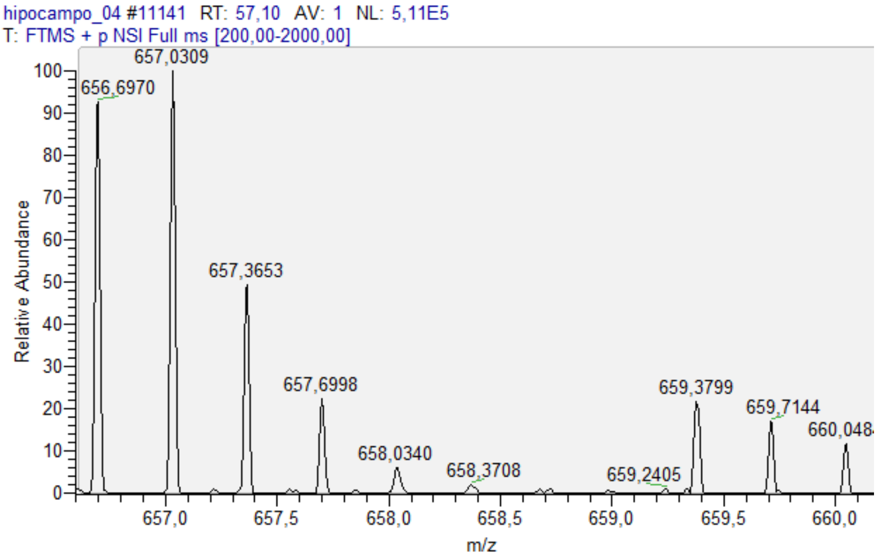

TVGDVNTDRPGLLDL 2+

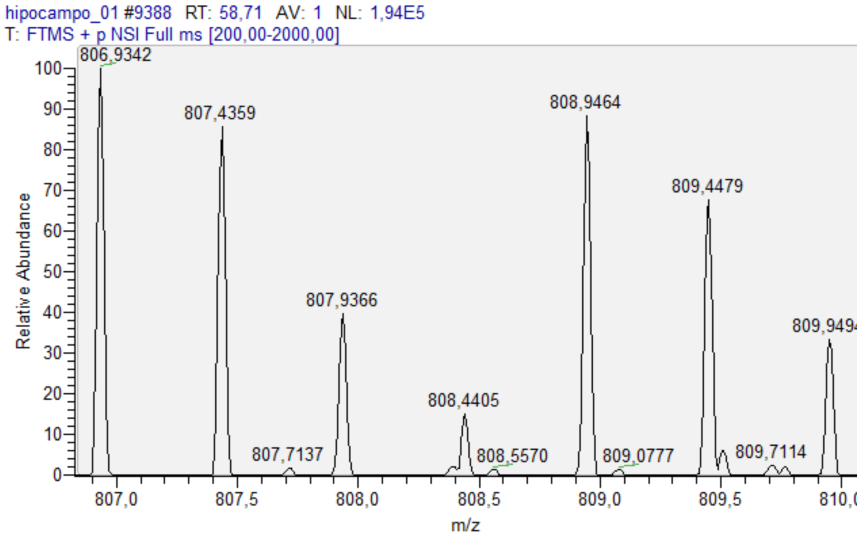

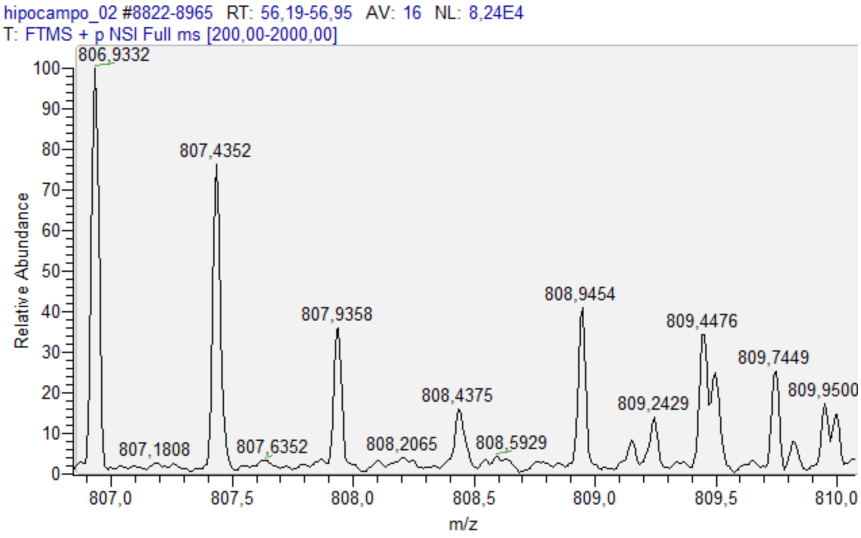

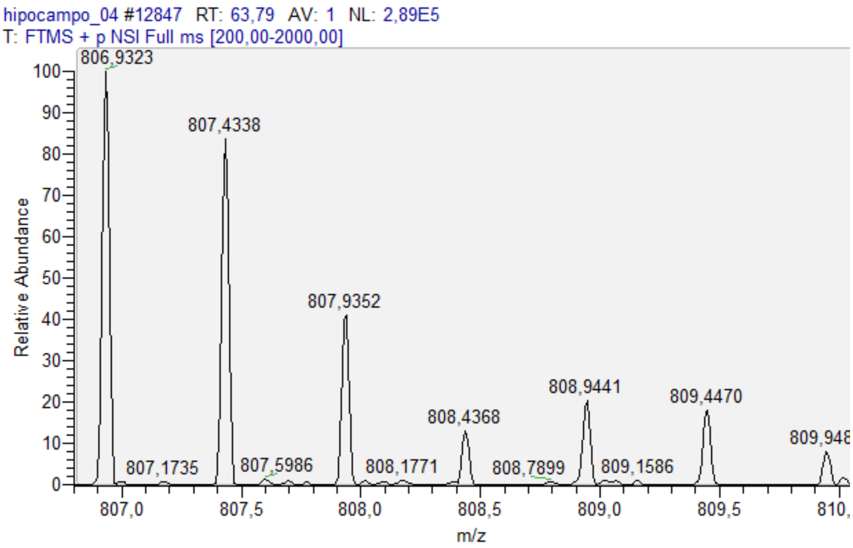

VEKVDELKKKYGI 2+

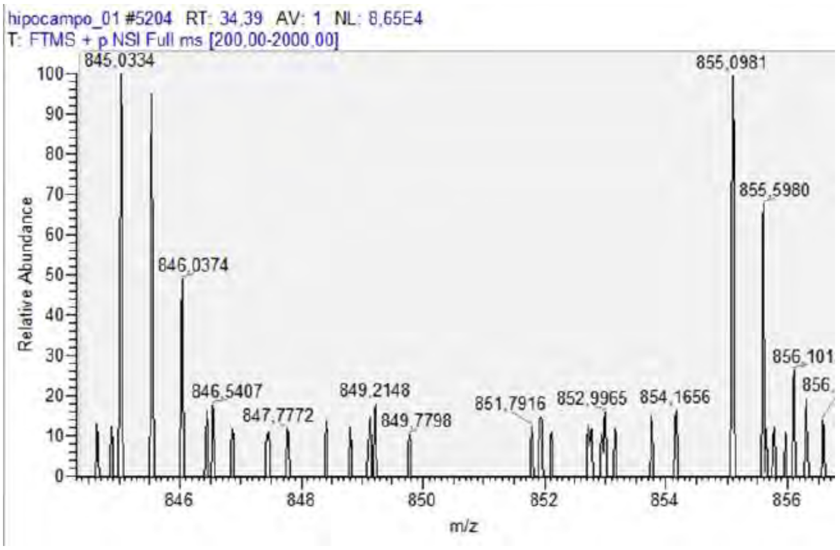

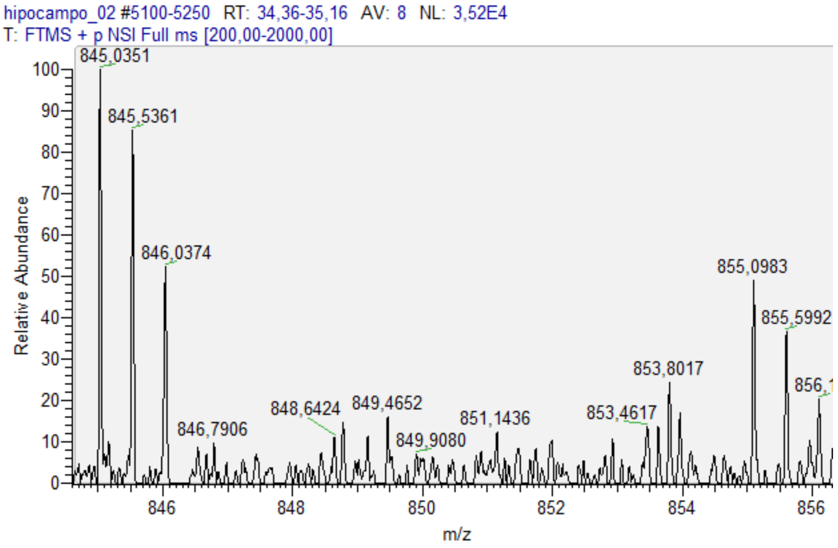

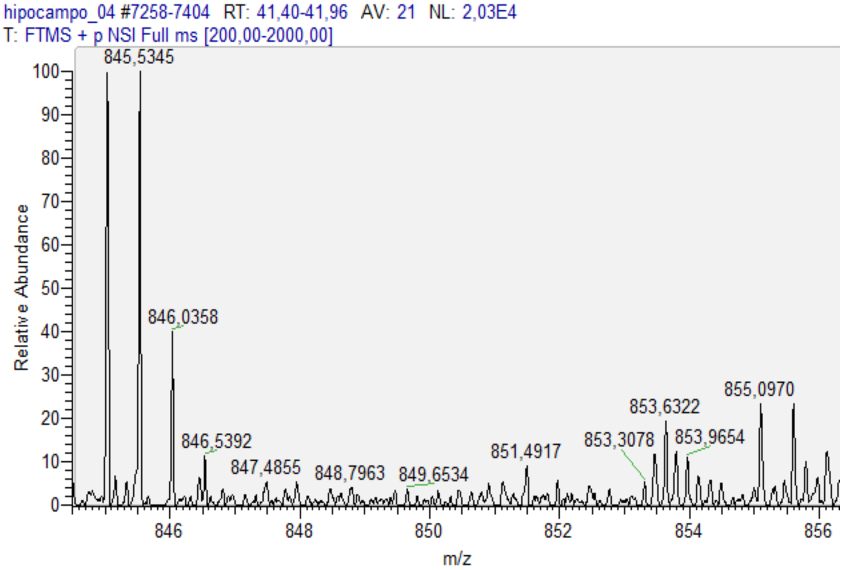

VEKVDELKKKYGI 3+

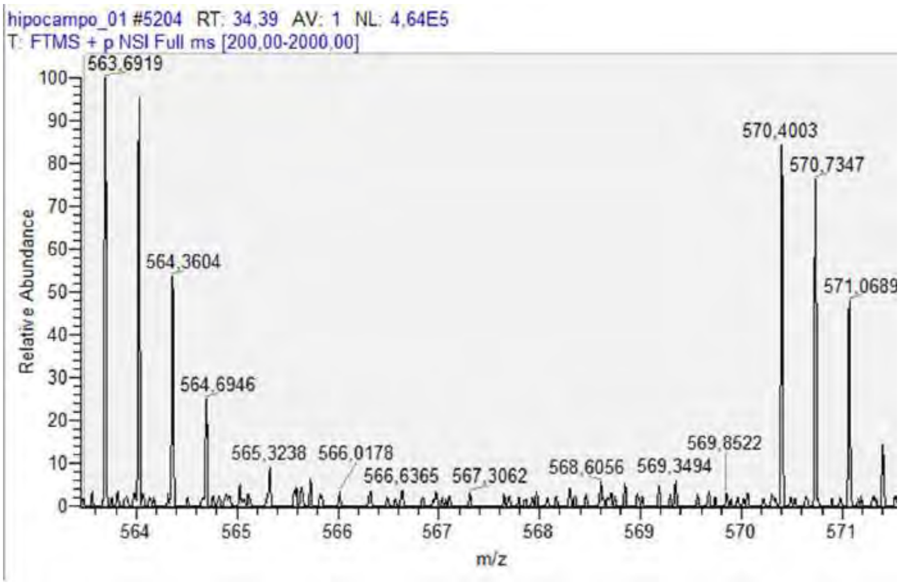

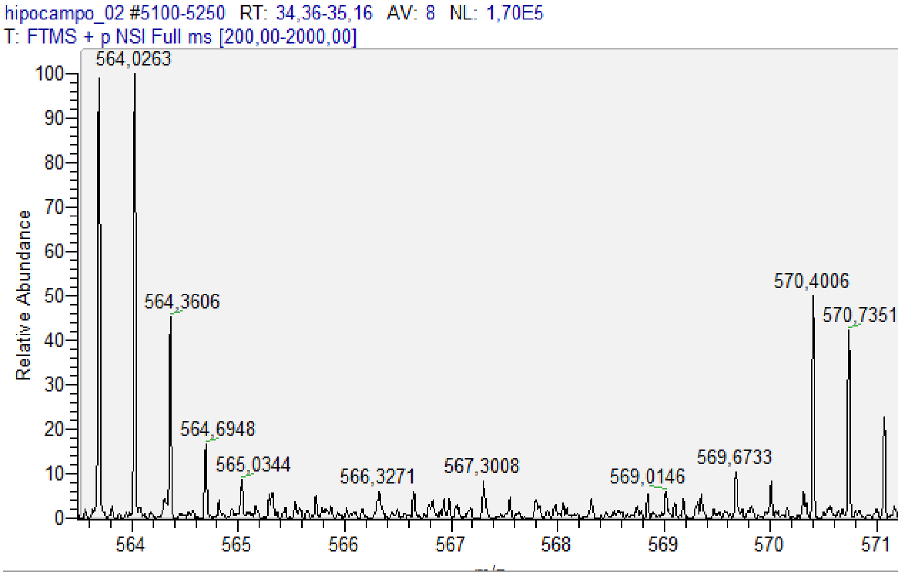

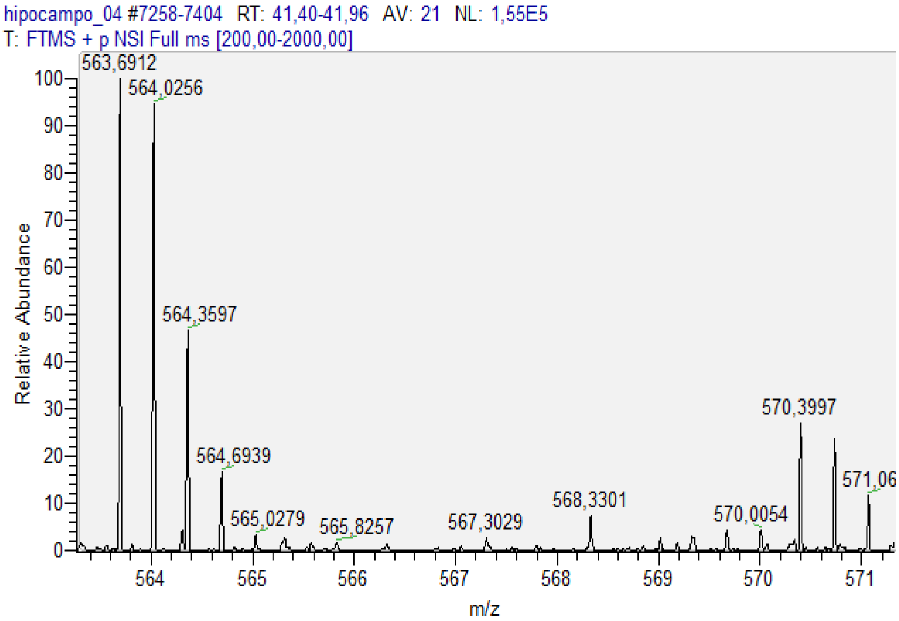

KGNDISSGTVL 2+

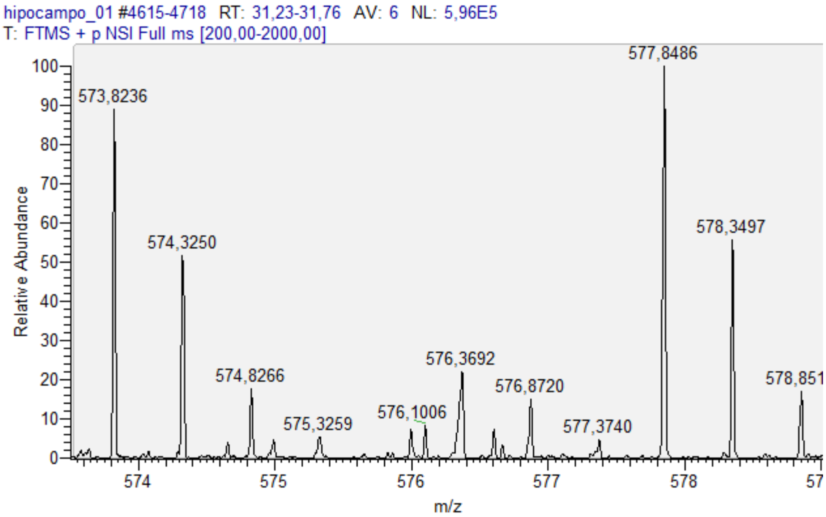

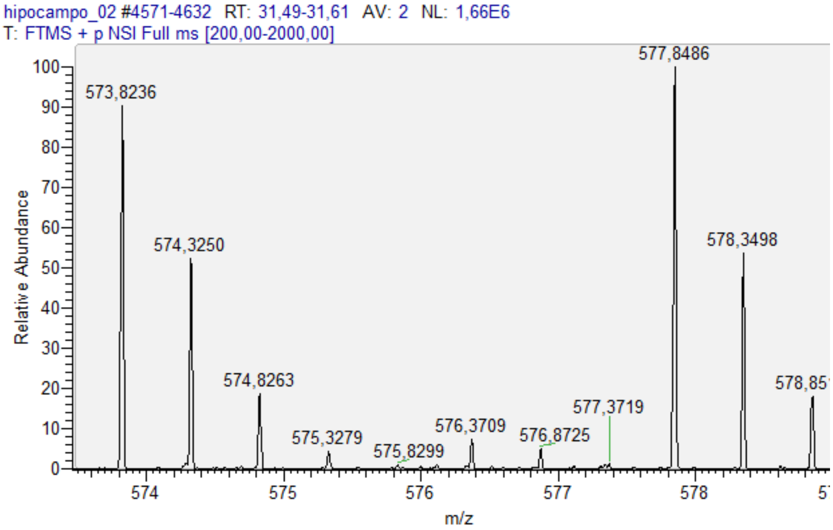

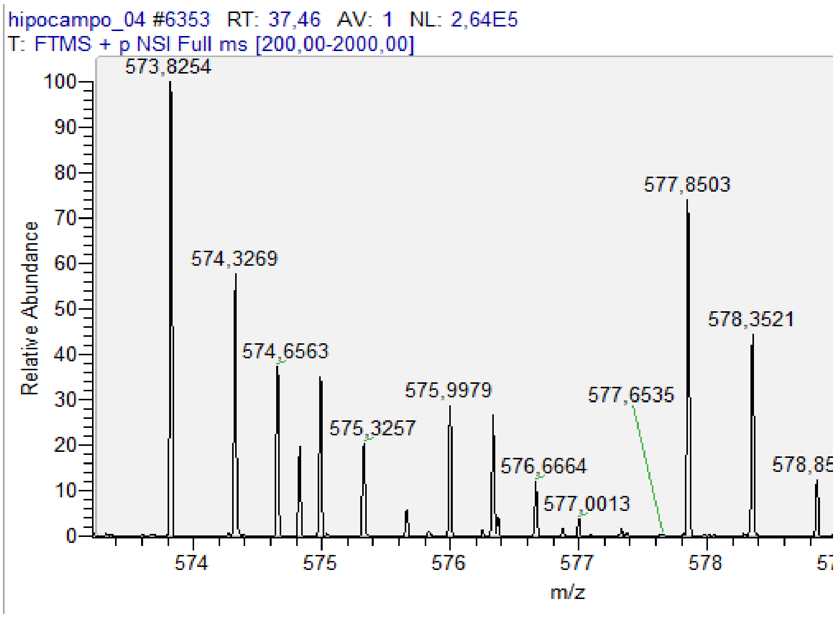

GLDPGKLYTL 2+

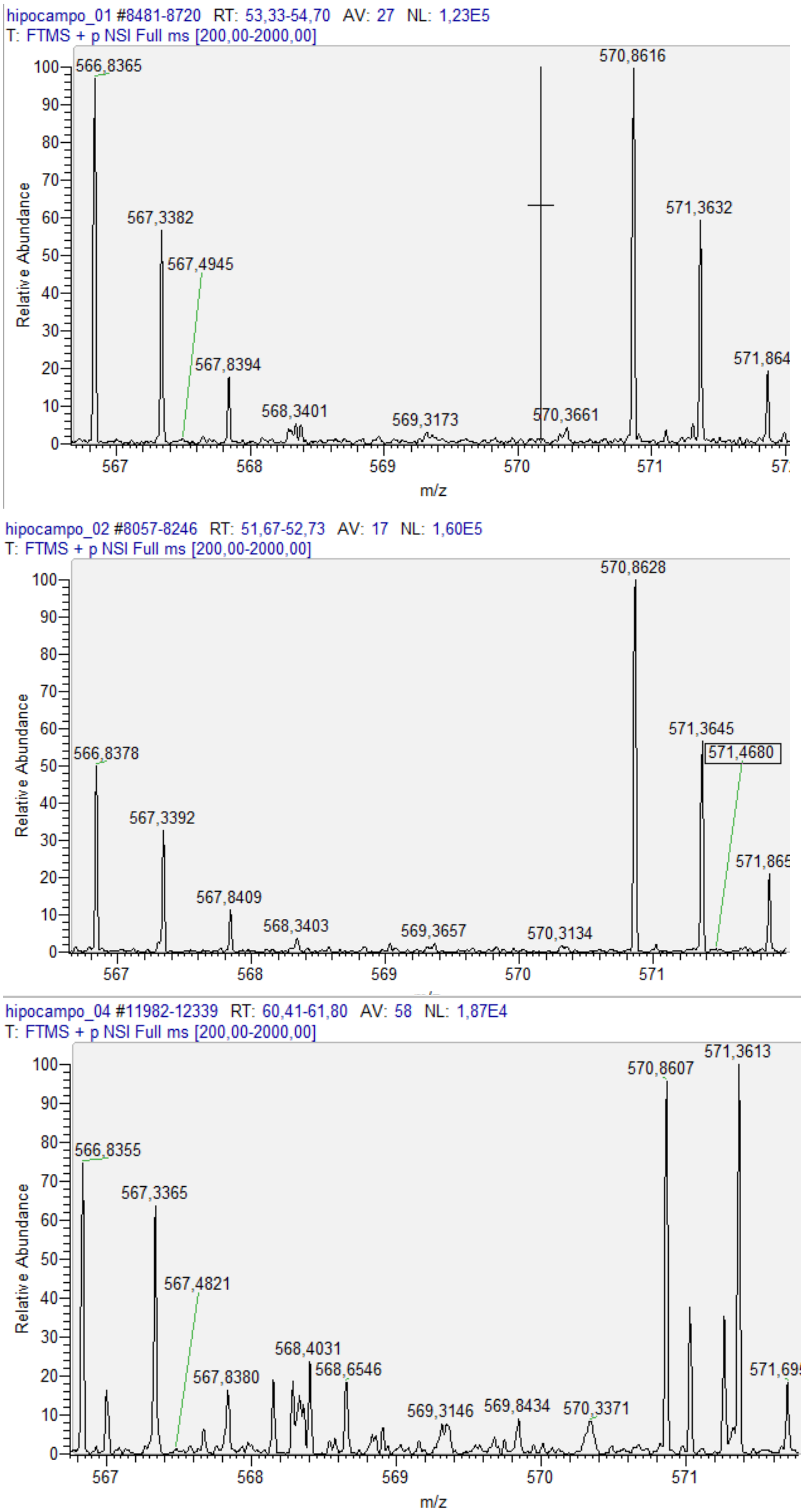

DGLDPGKLYTL 2+

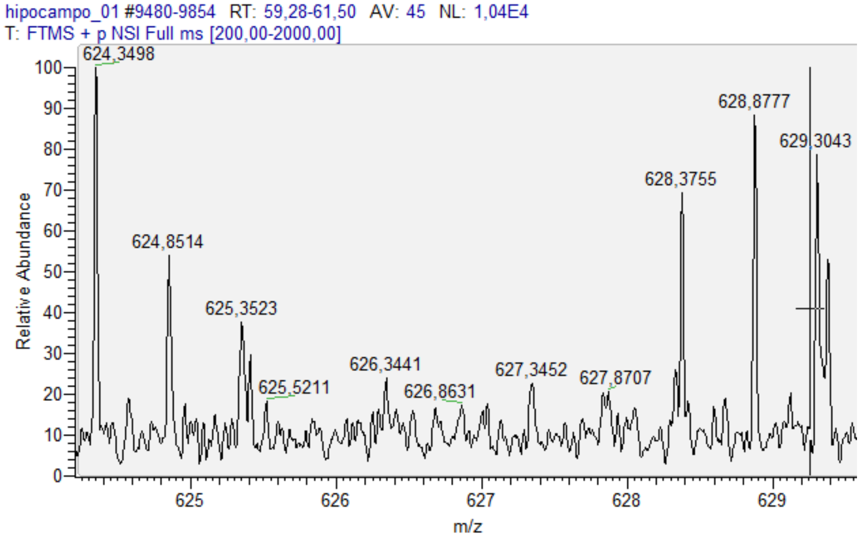

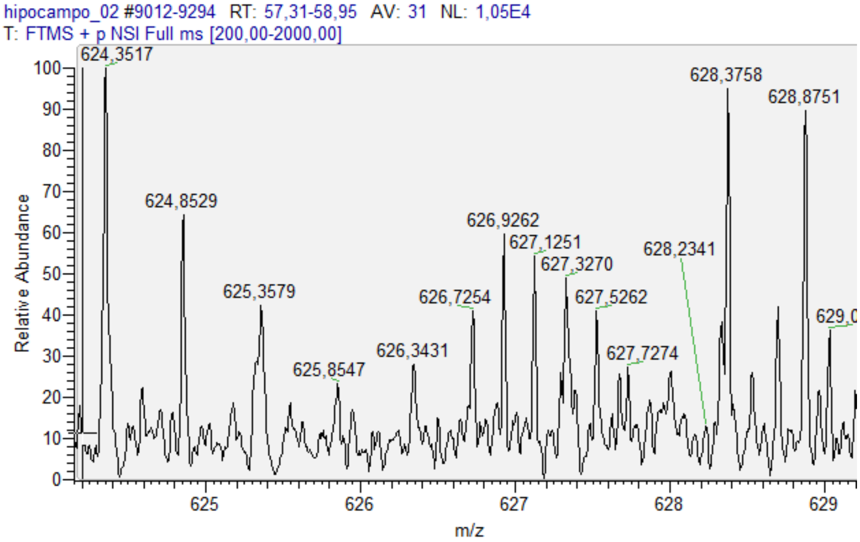

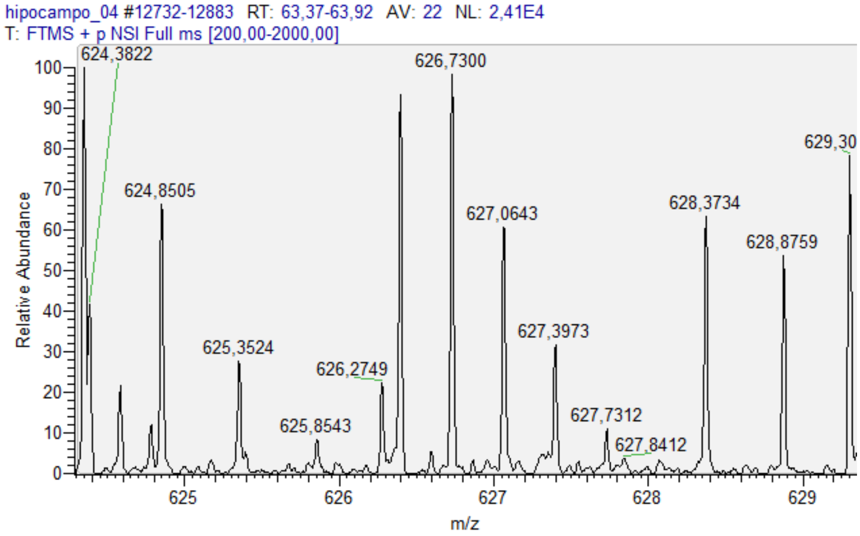

DDYVPKLYEQLSGK 3+

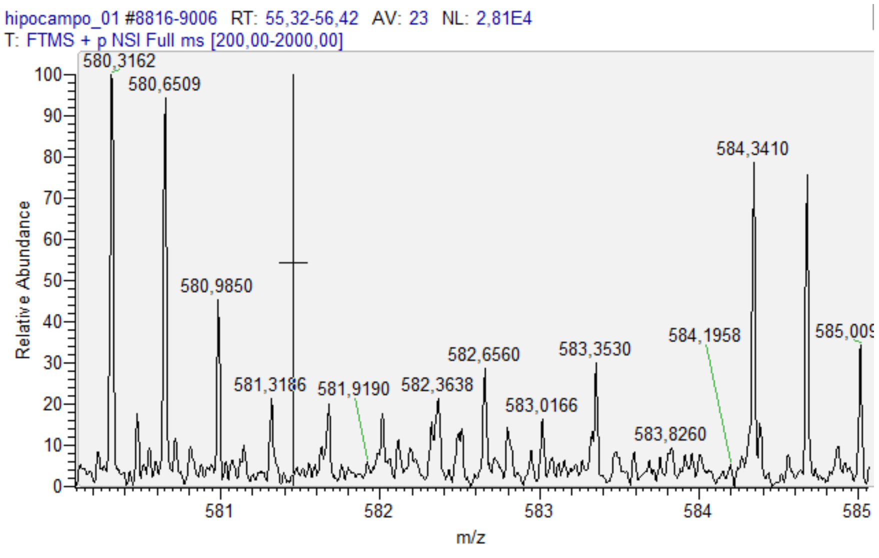

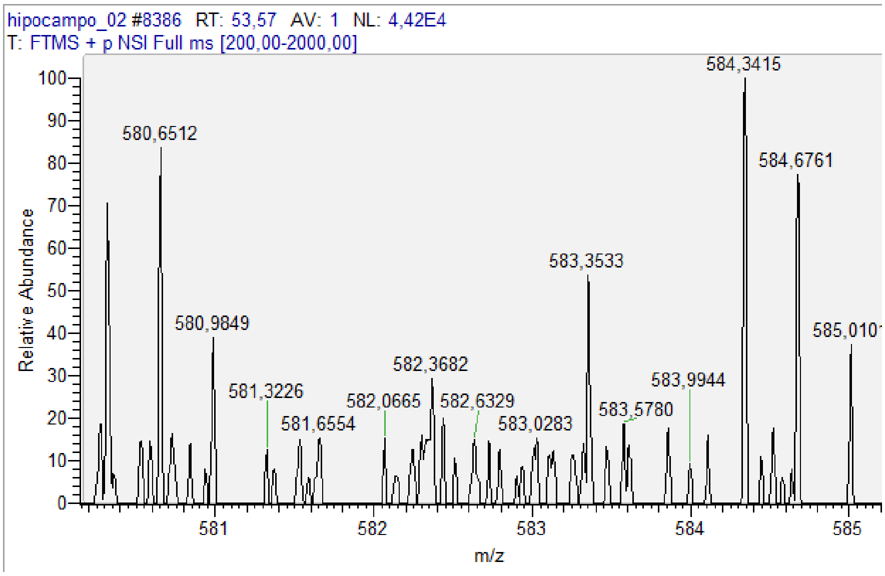

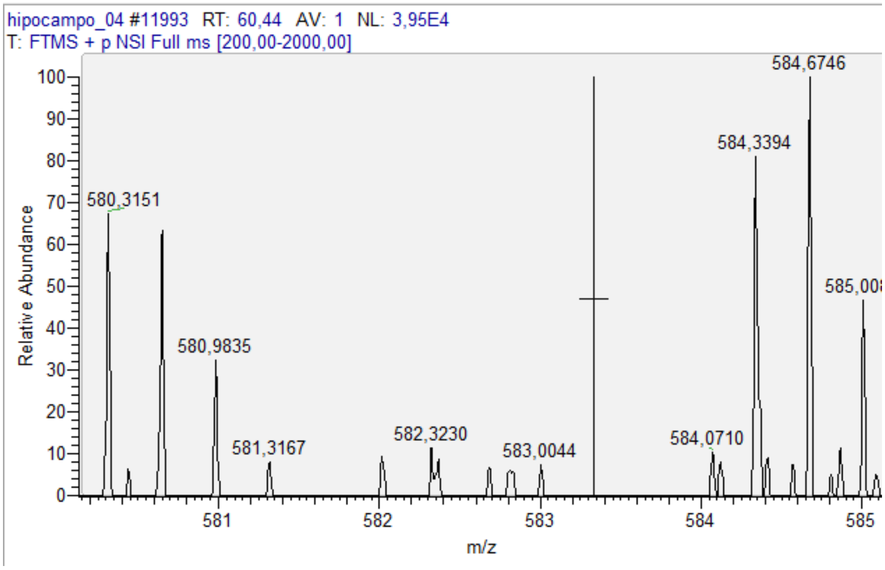

AGVTVDELGKVLTPTQV 2+

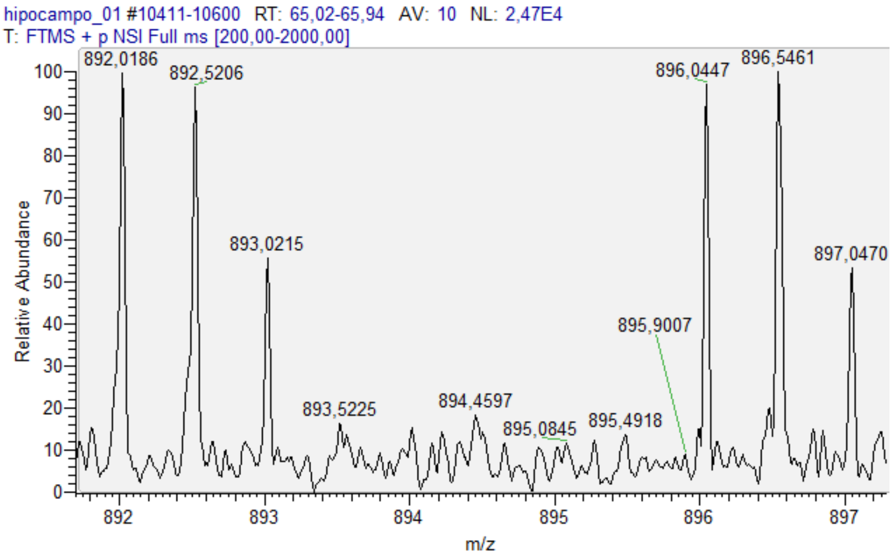

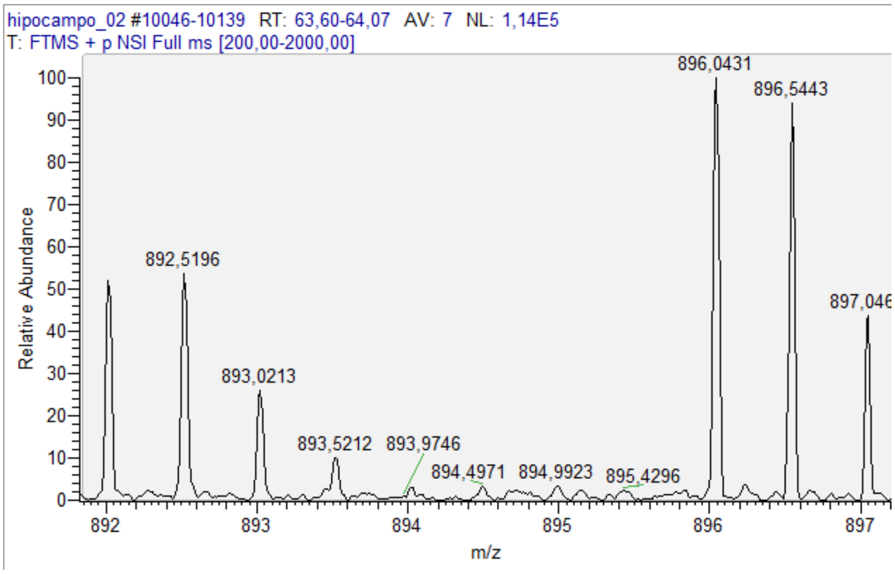

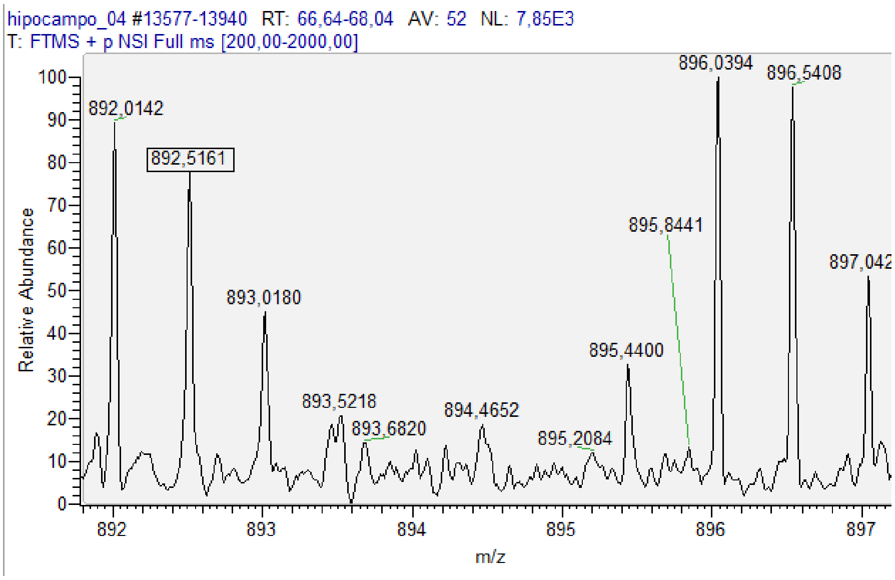

SGPFGQIFRPDNF 2+

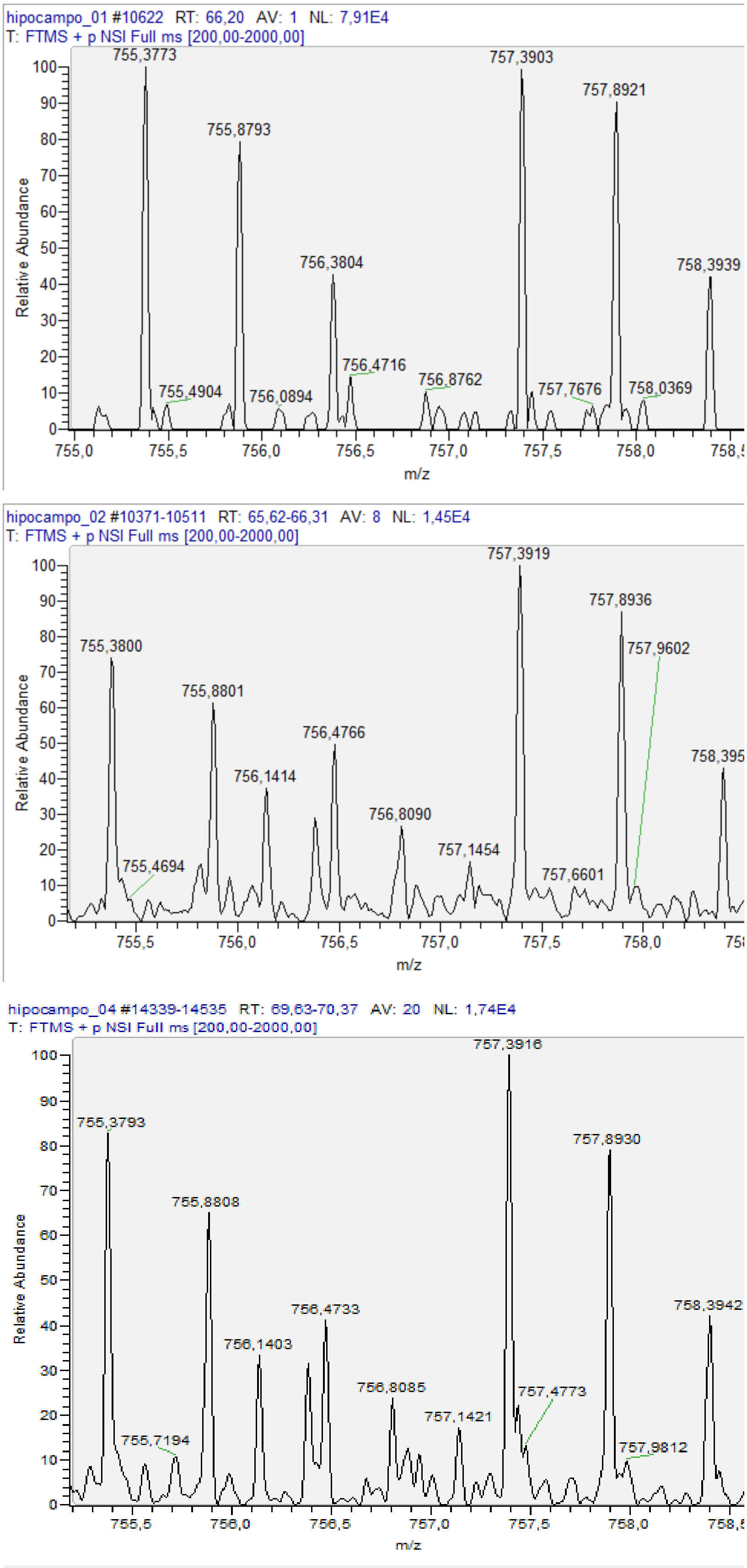

DDEESEAQGPK 2+

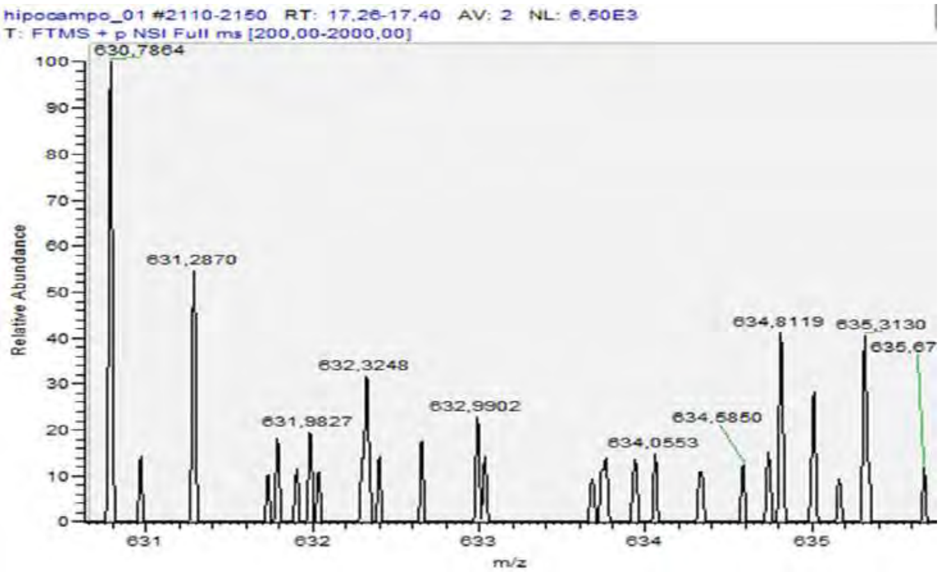

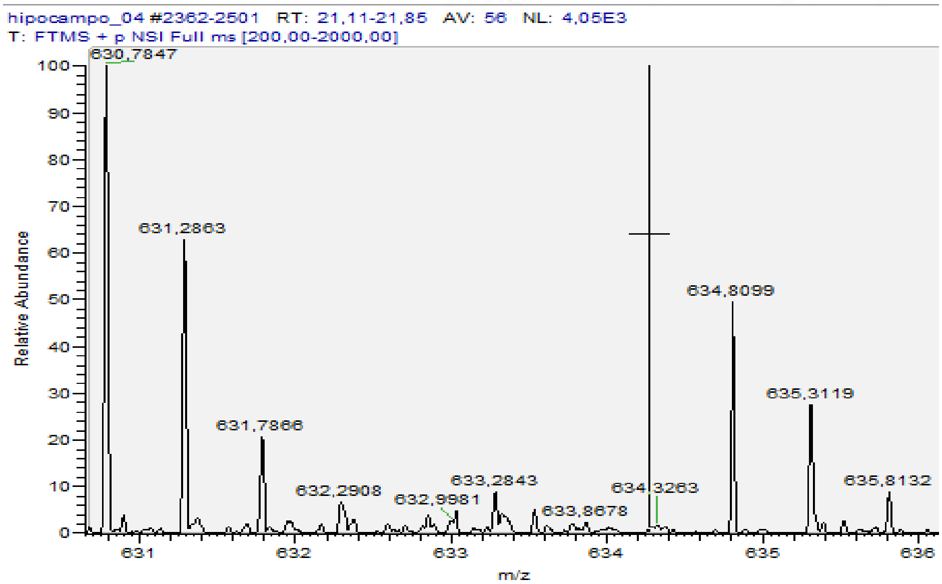

SPQLEDEAKELQ 2+

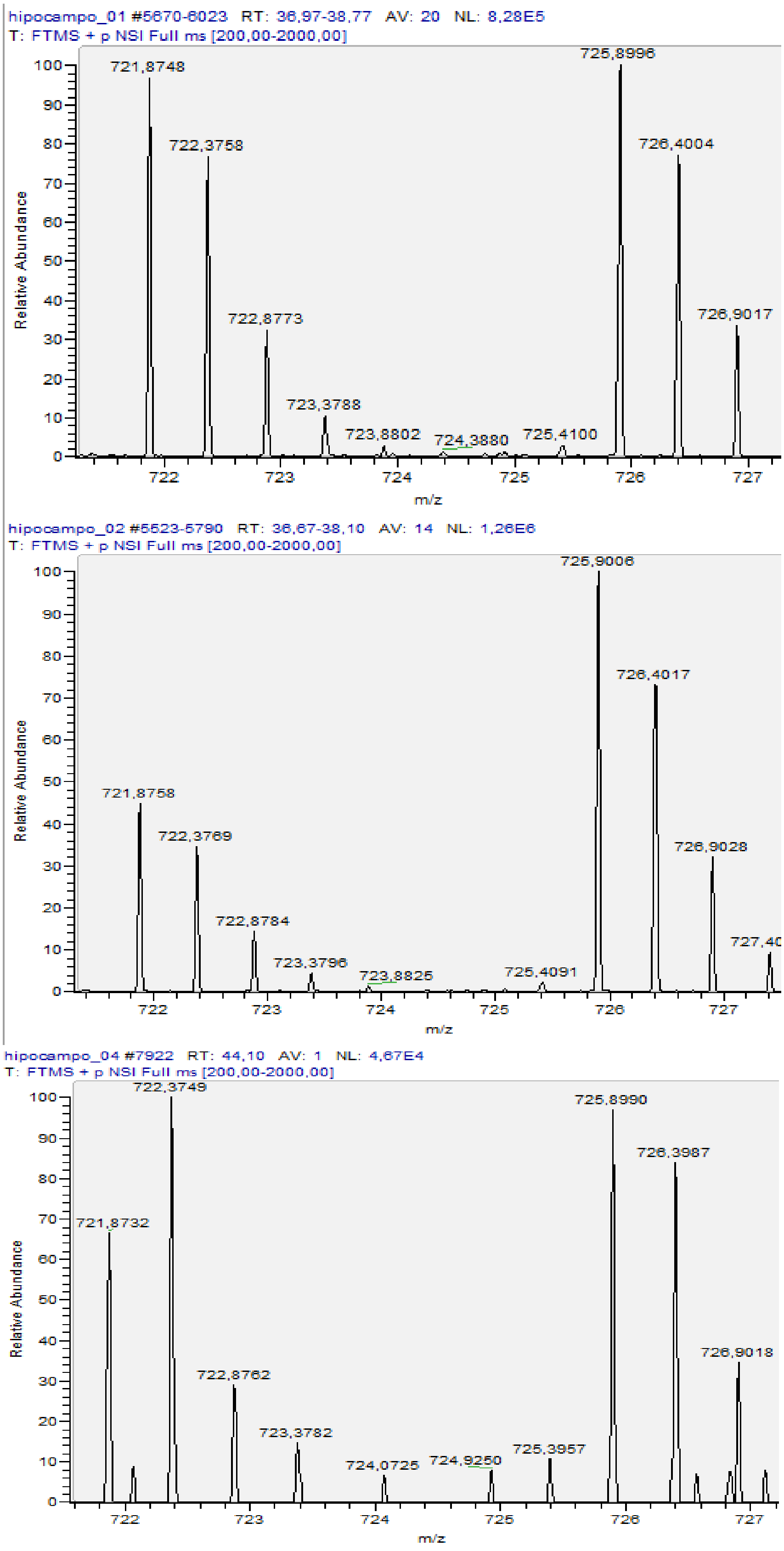

FDITADDEPLGRVSF 2+

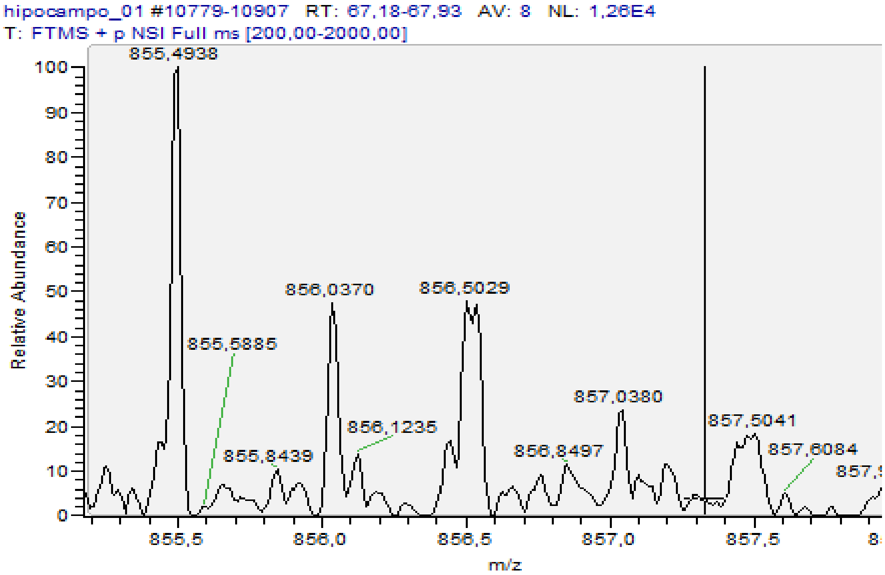

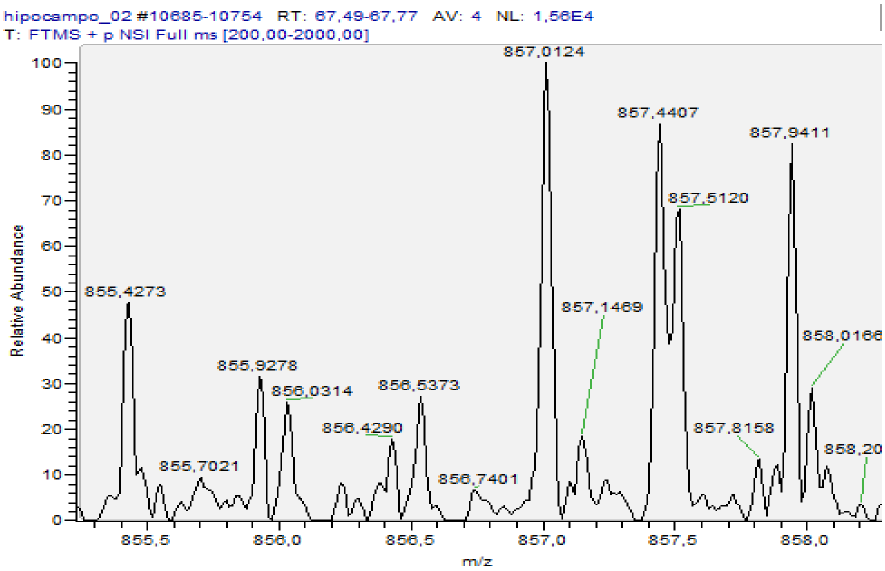

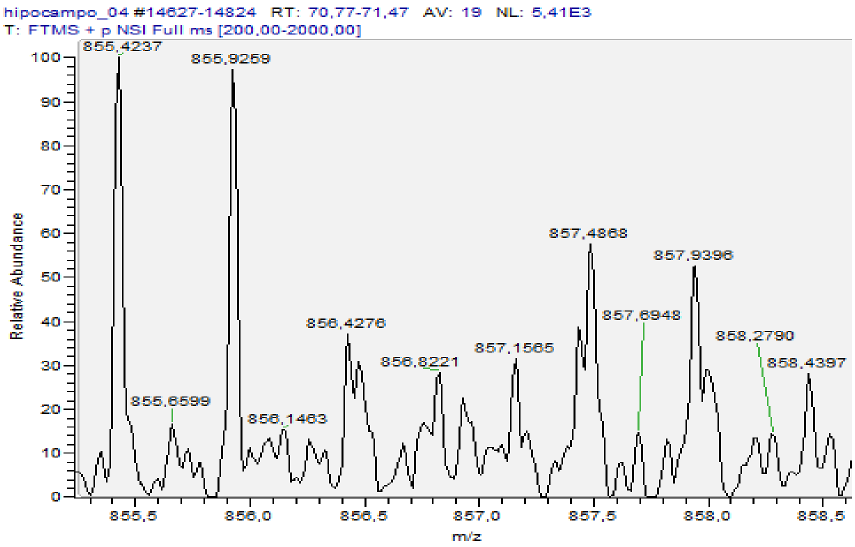

ITADDEPLGRVSF 2+

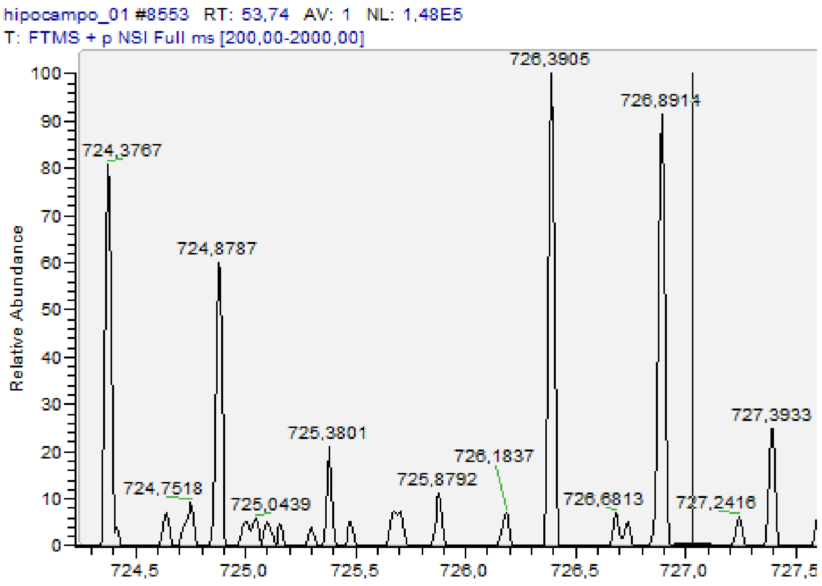

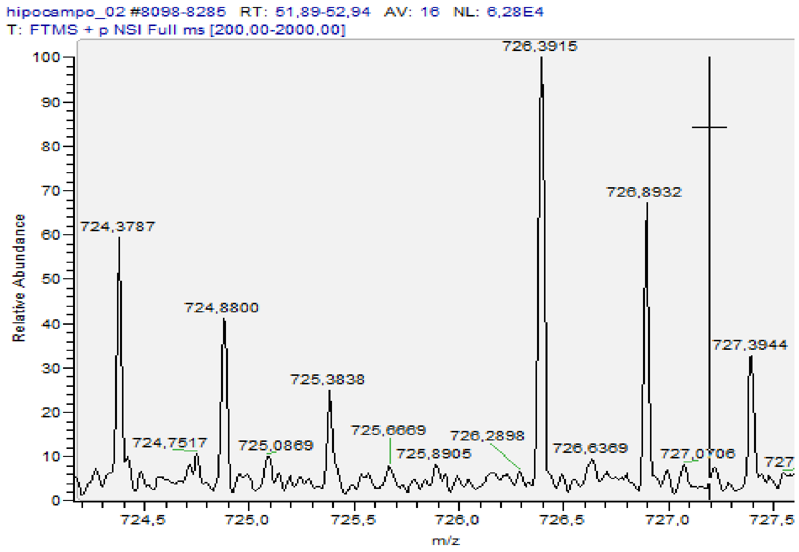

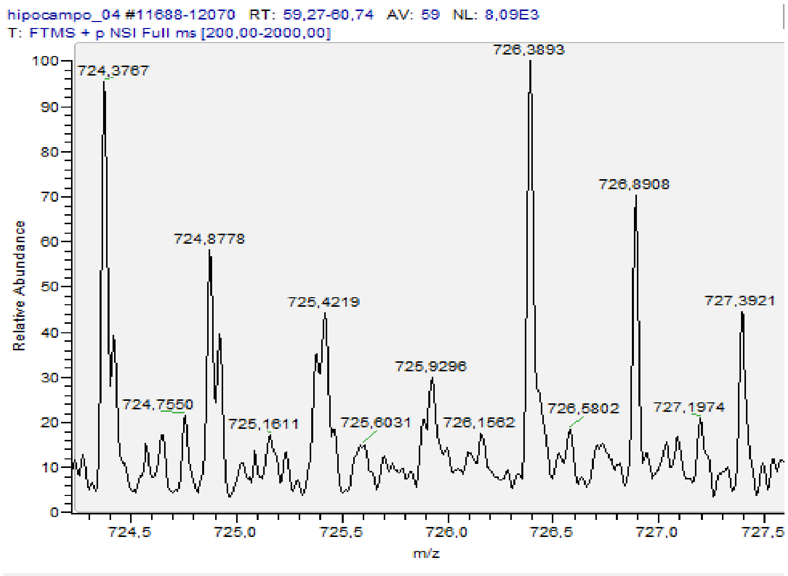

LFADKVPKTAENF 3+

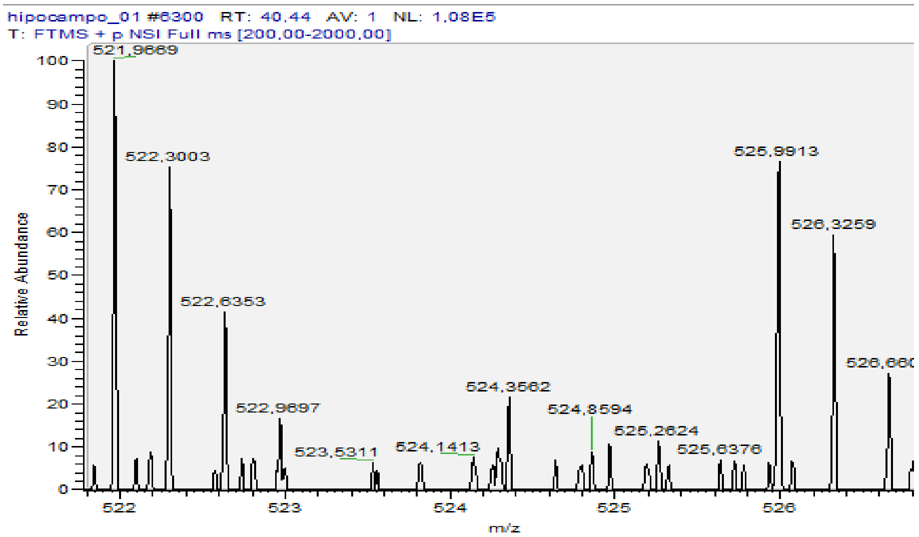

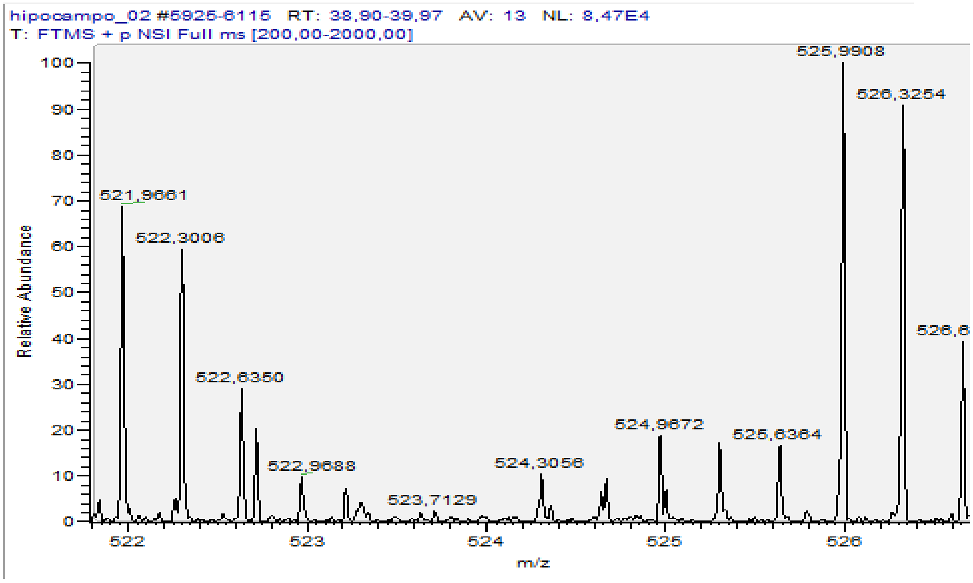

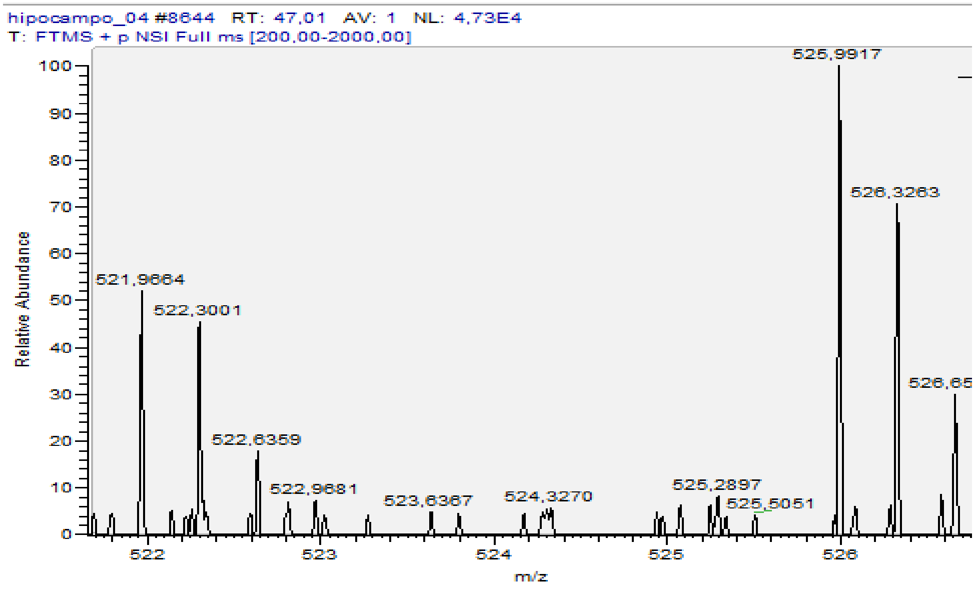

FHNPHVNPLPTGYEDE 2+

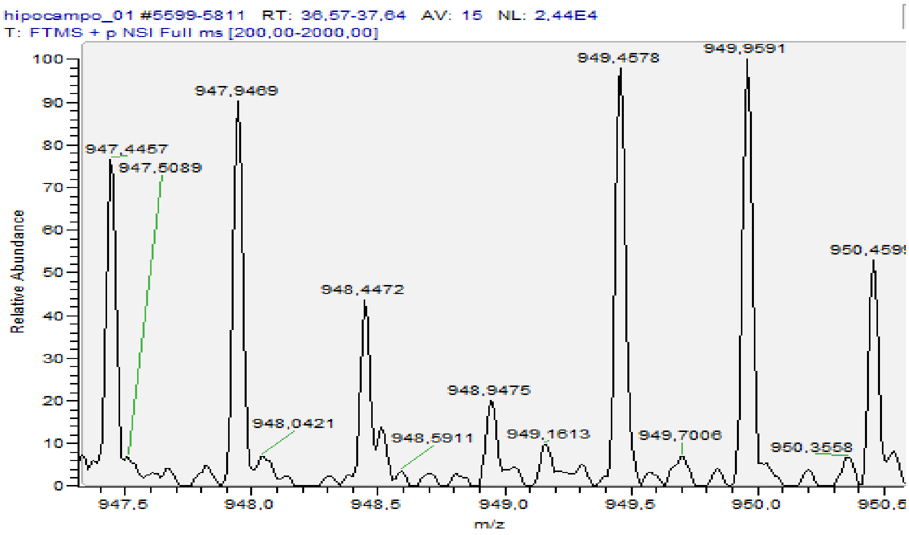

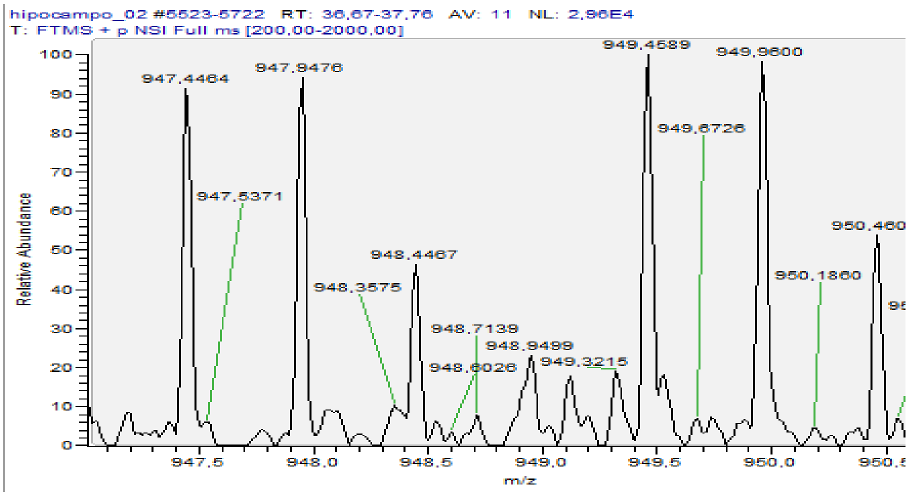

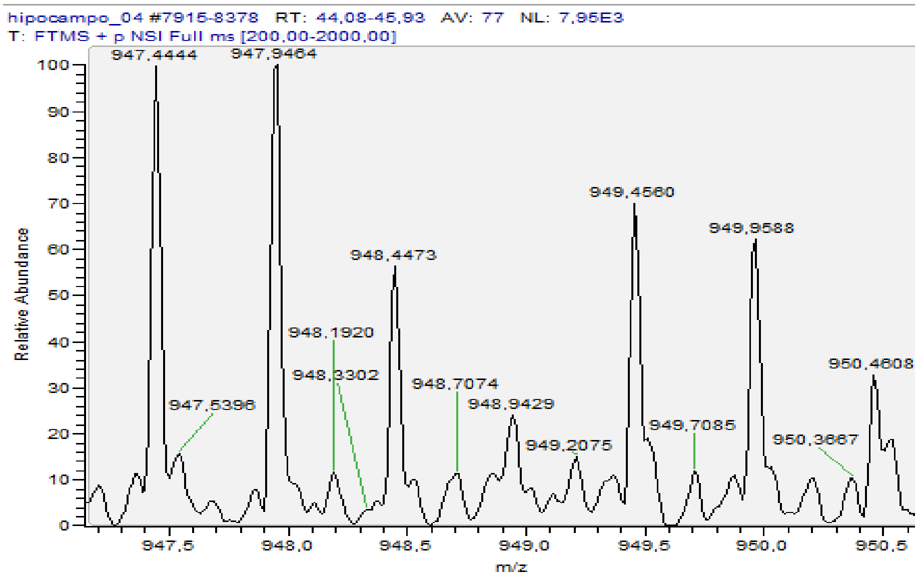

HNPHVNPLPTGYEDE 2+

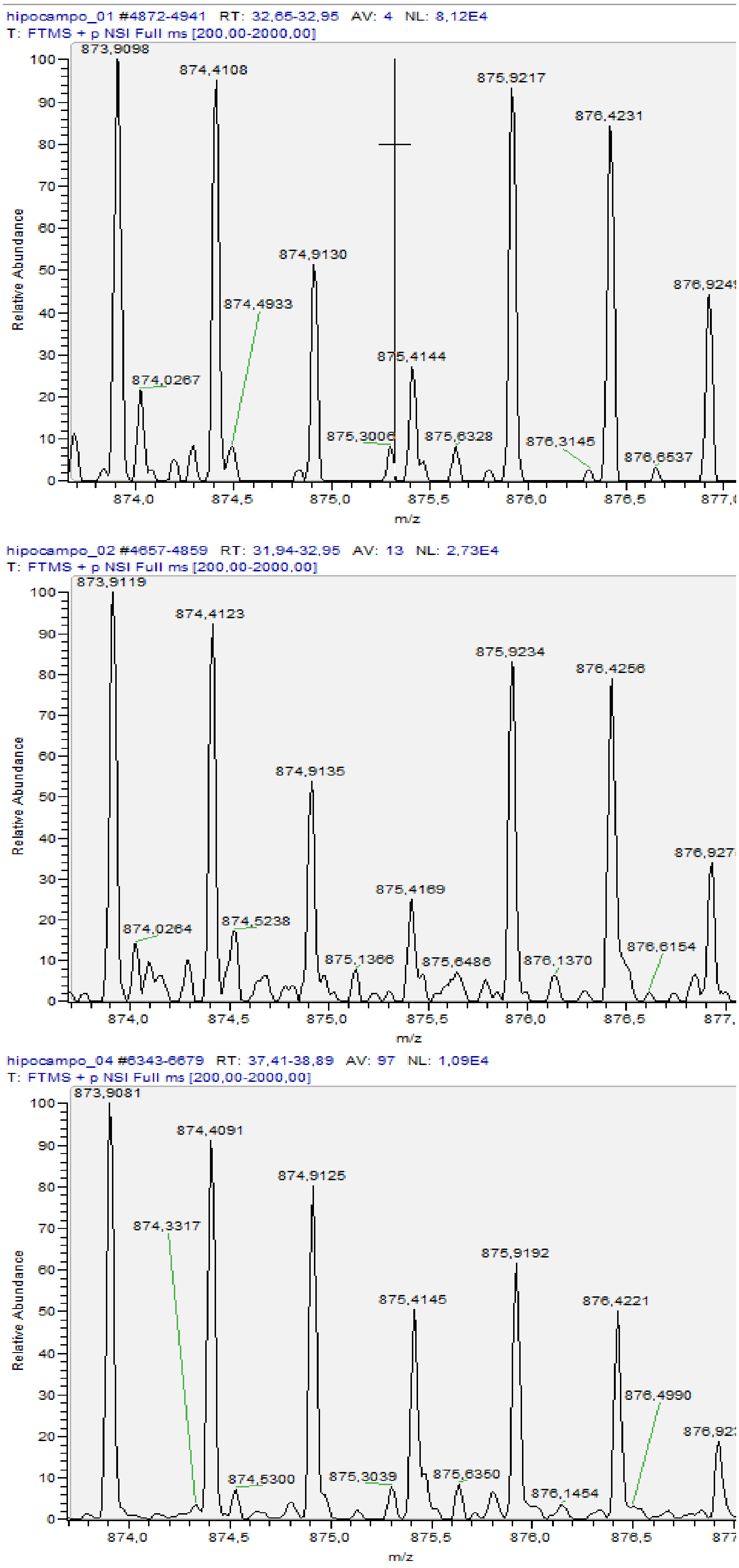

NPHVNPLPTGYEDE 2+

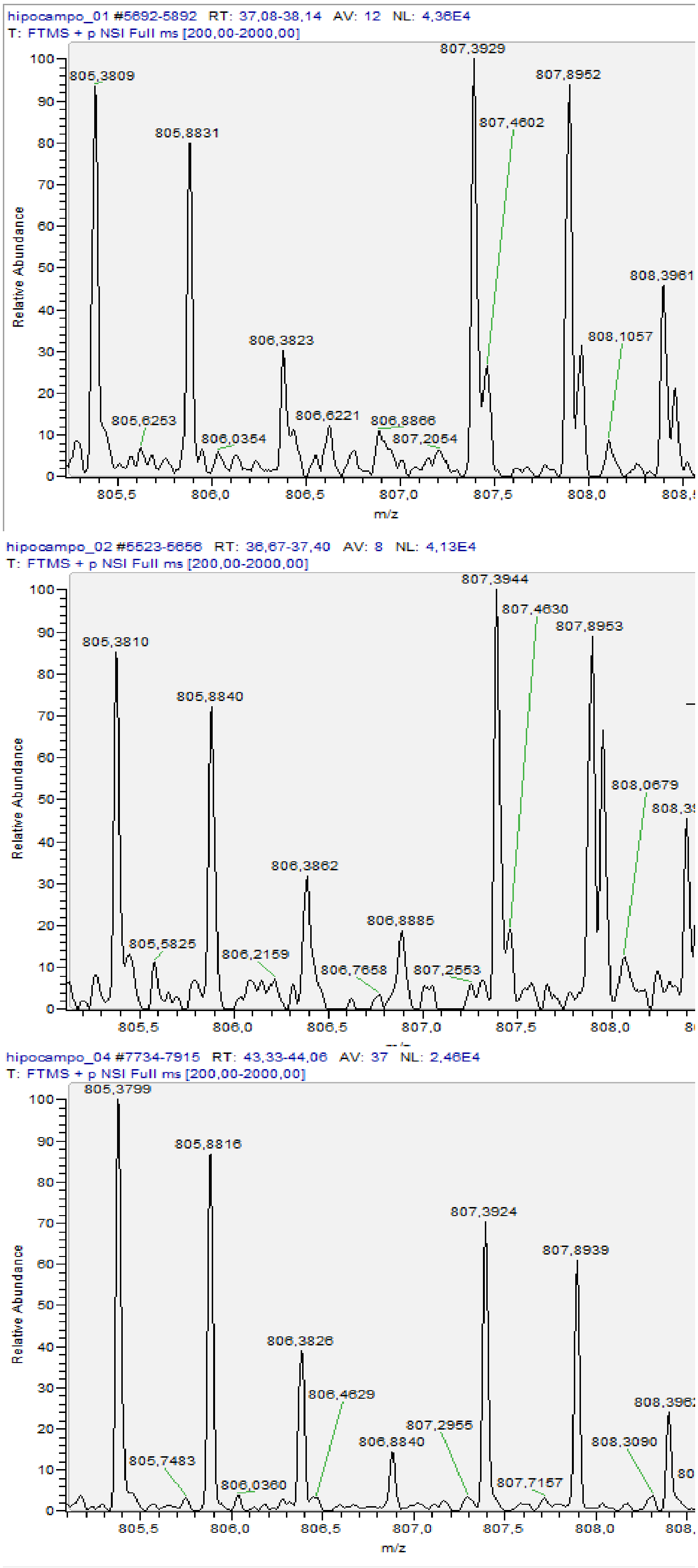

SLSAASAPLVETSTPLRL 2+

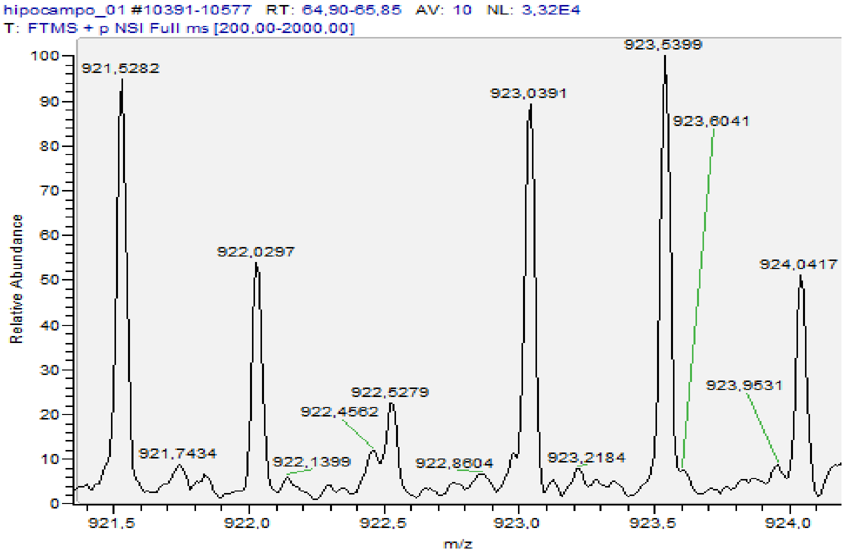

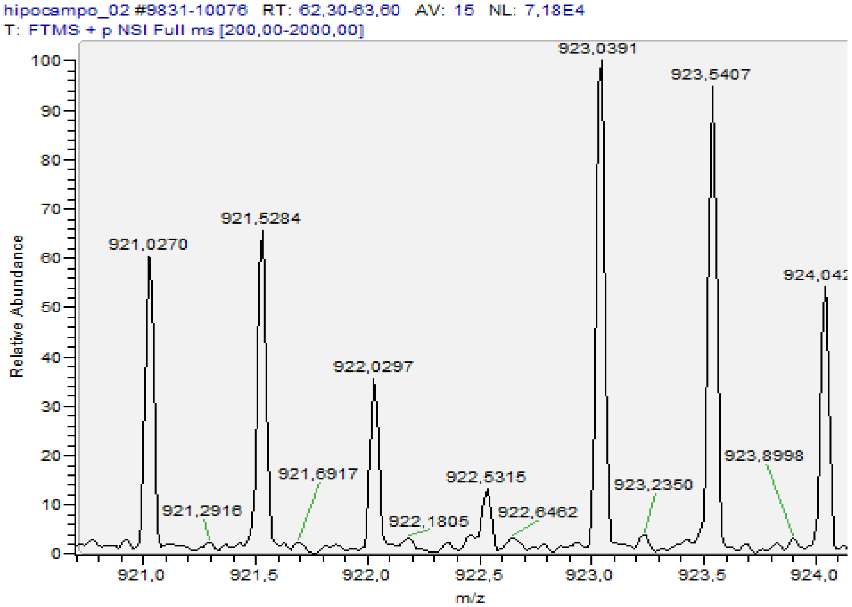

AQATGKPAQYIA 2+

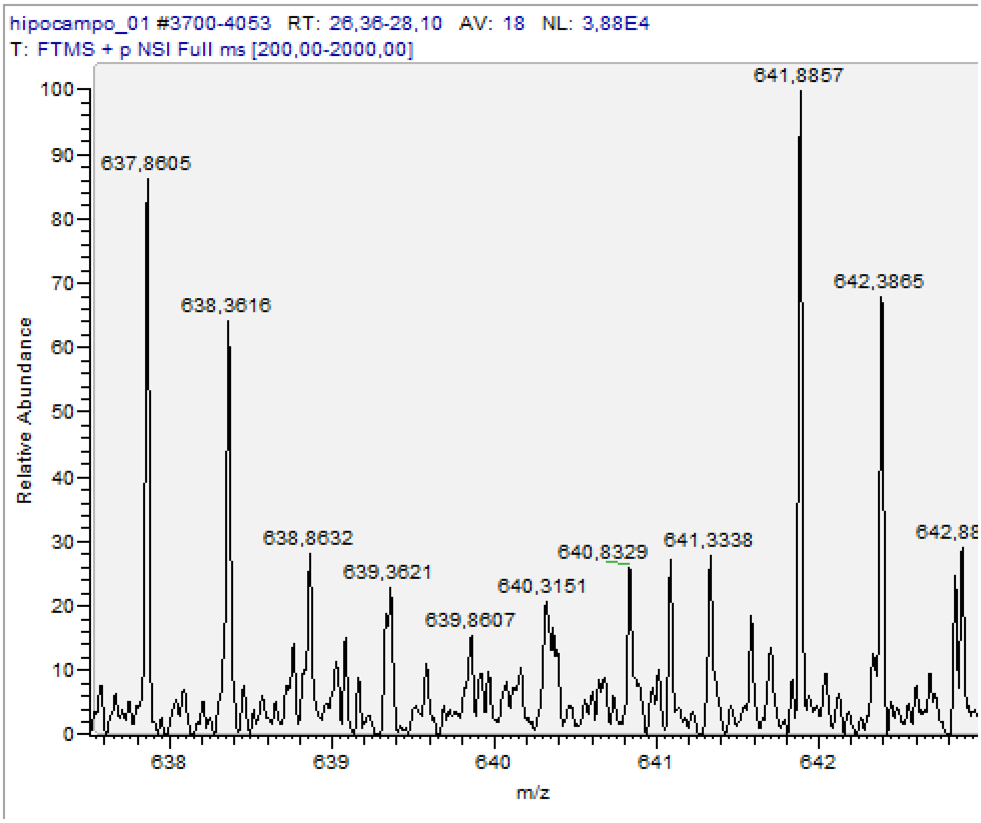

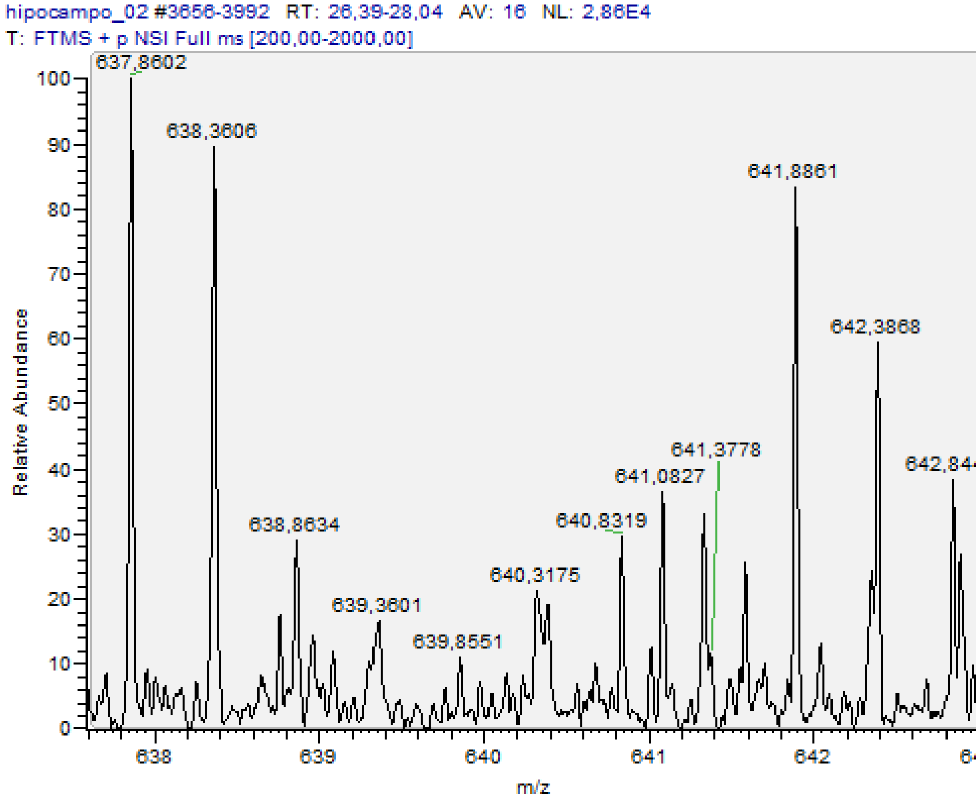

AQATGKPAQYIAVHVVPDQL 2+

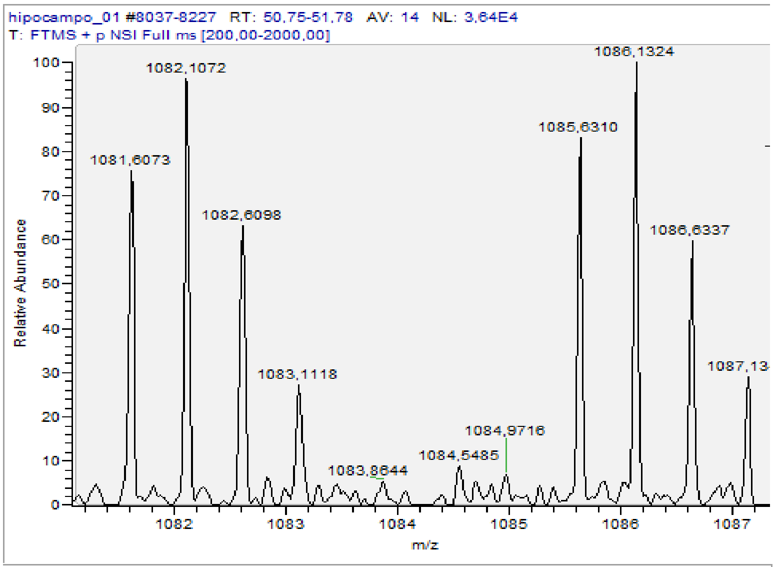

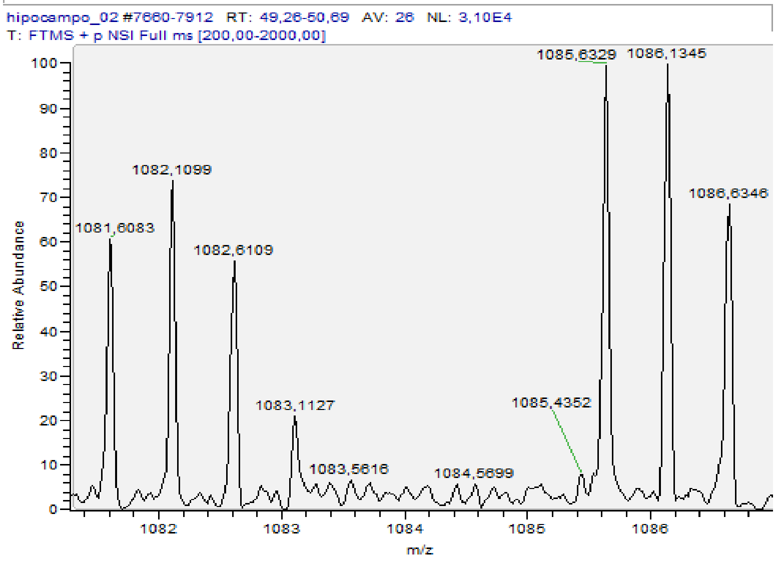

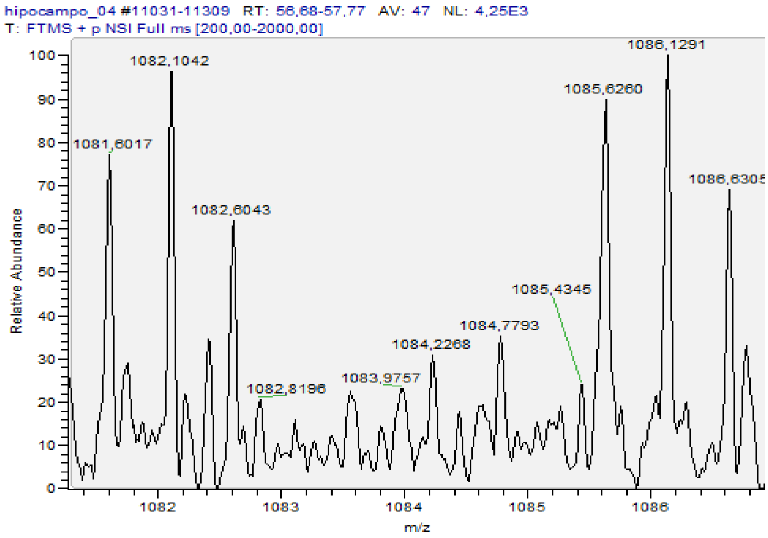

AQATGKPAQYIAVHVVPDQL 3+

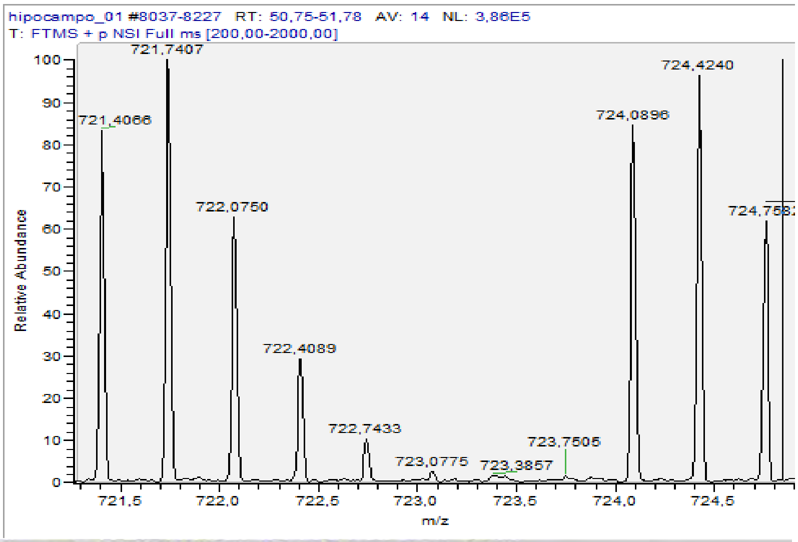

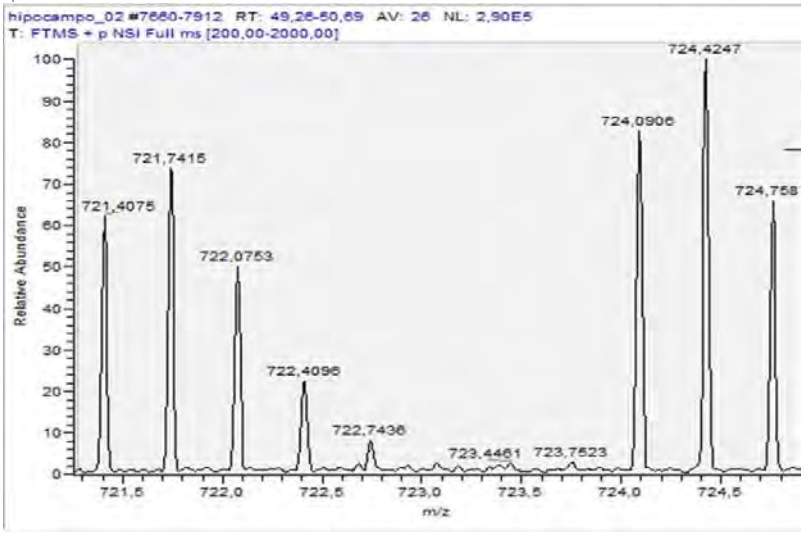

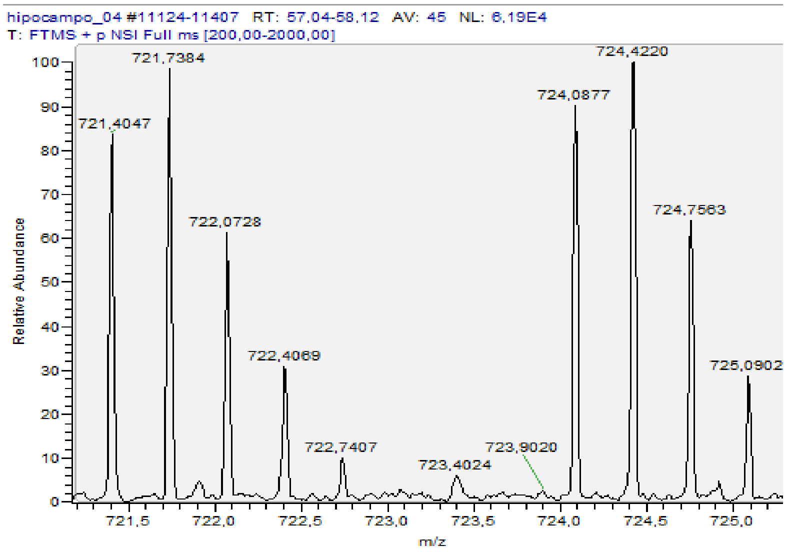

ASKGLGSDLDSSLASL 2+

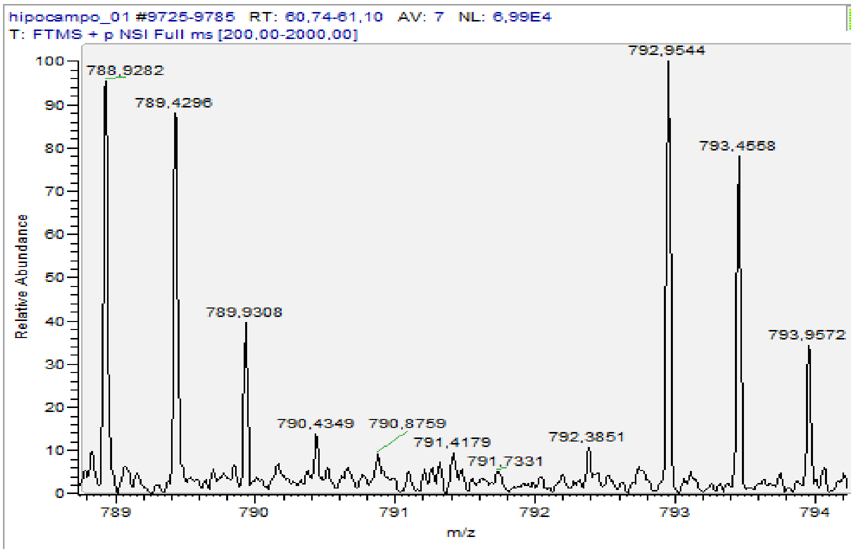

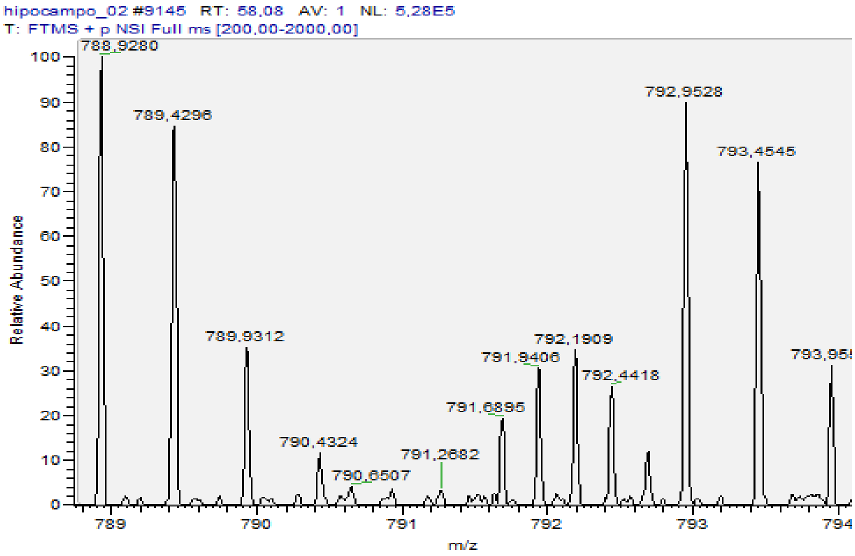

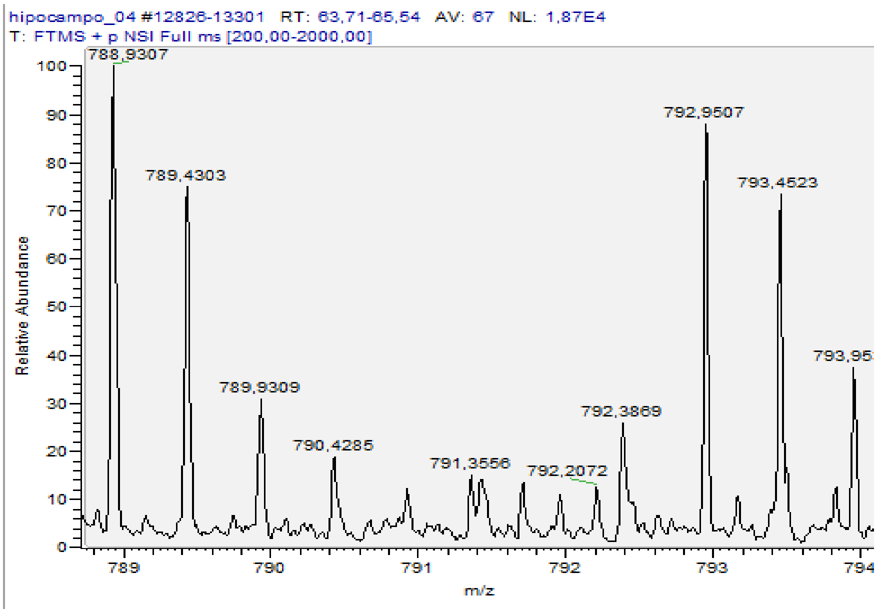

SPSPTPATQSPKKPPAKDPLADLNIKDFL 2+

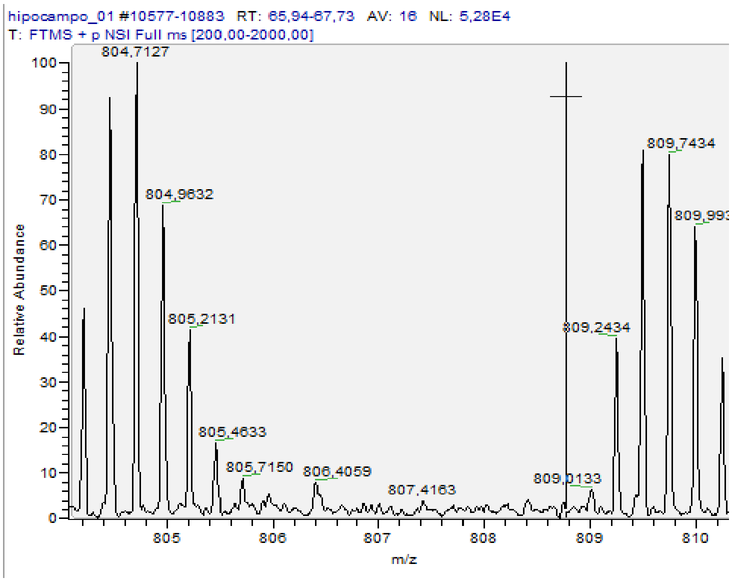

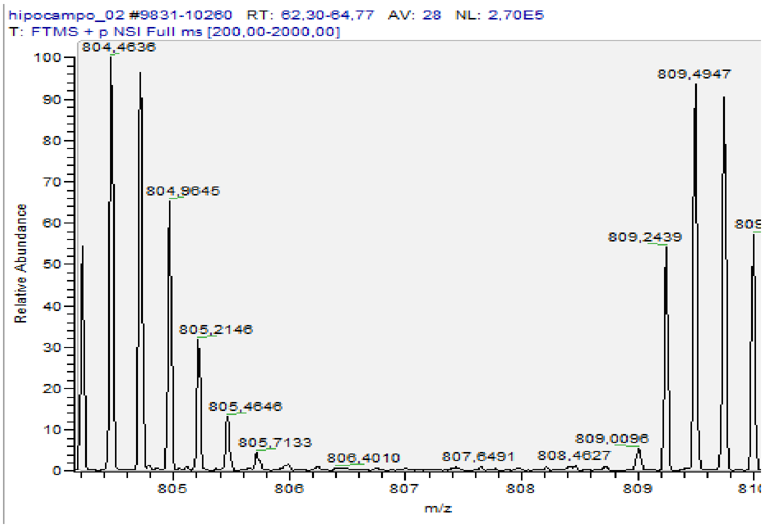

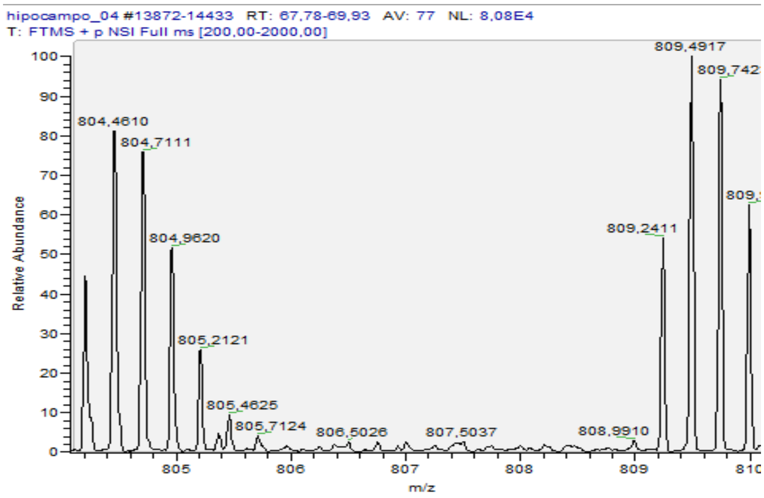

TQVVSDAAYKGVQPHVV 2+

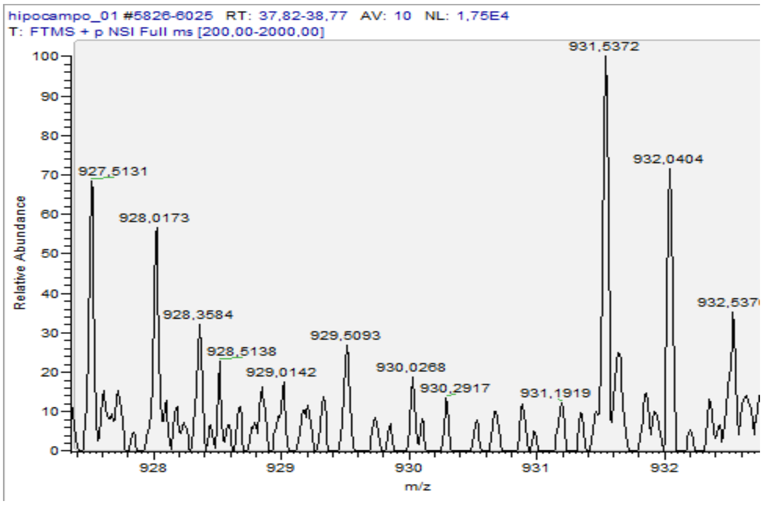

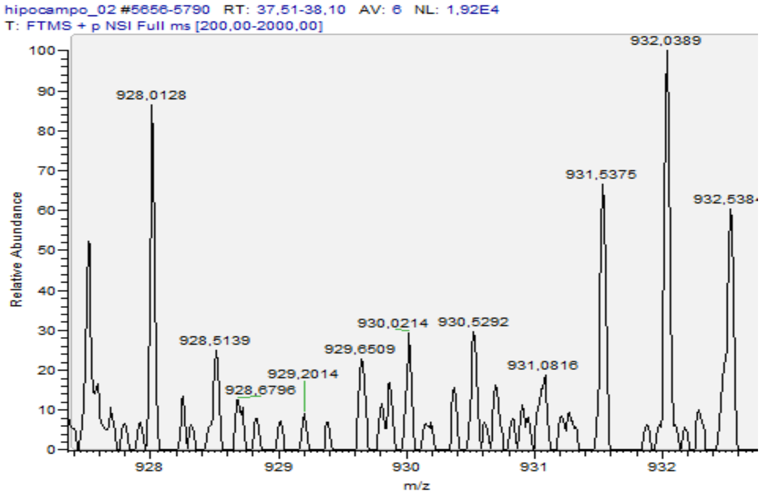

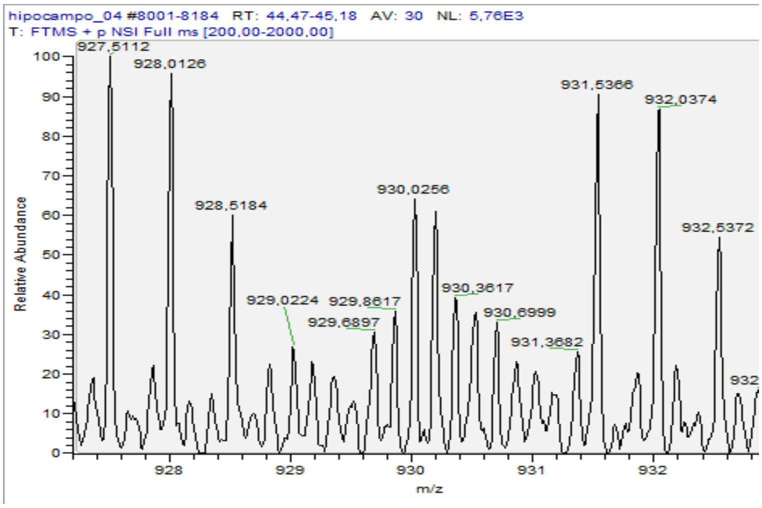

ADEVSASLAKQGL 2+

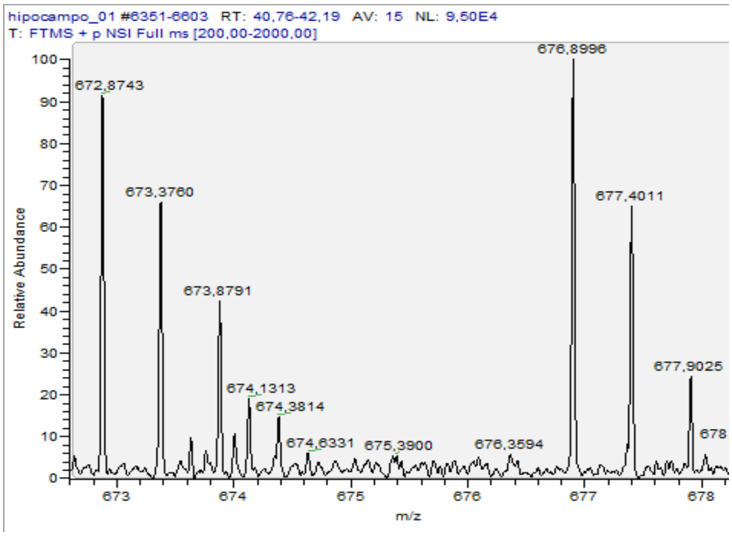

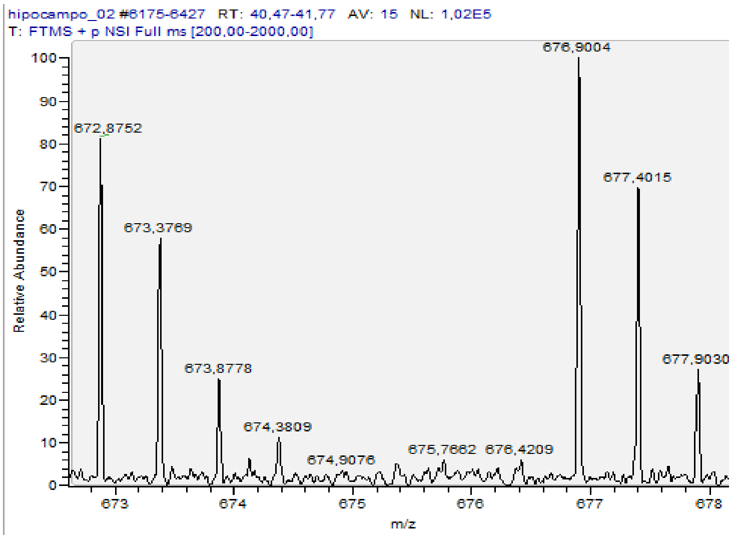

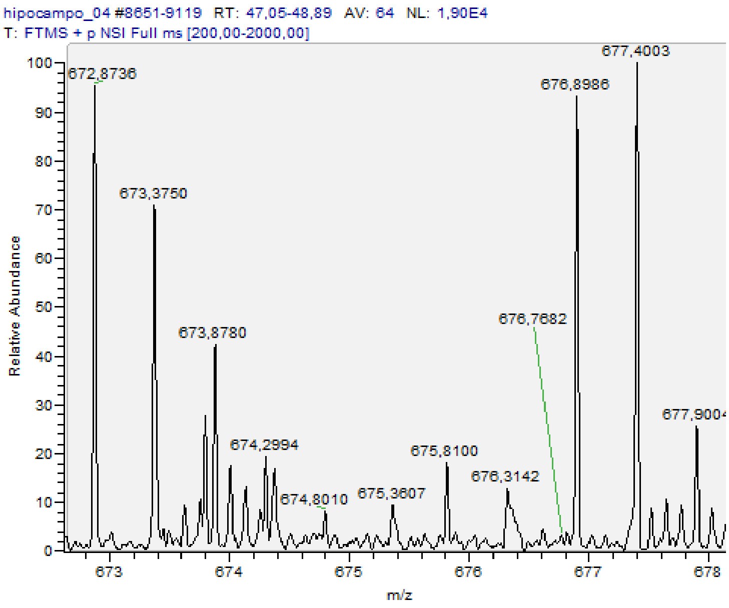

ANSNPAMAPRE 2 (METHIONINE OXIDATION)

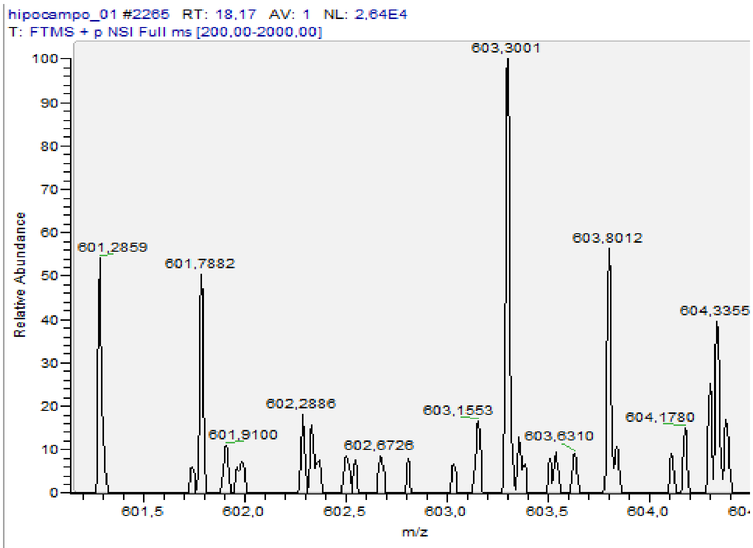

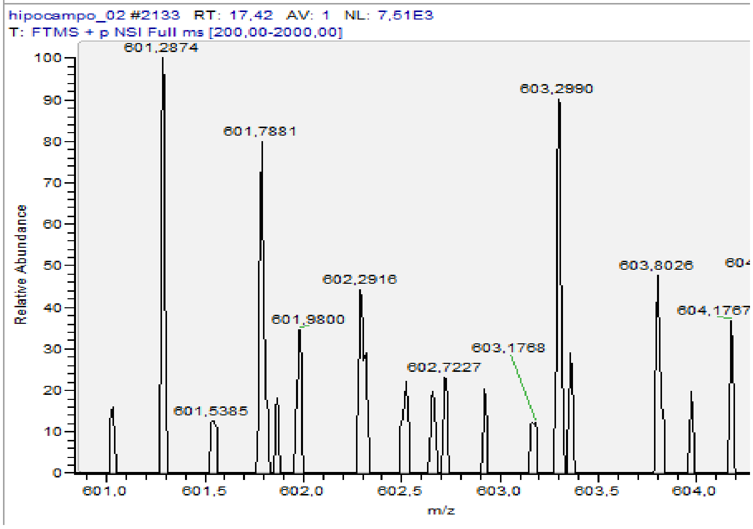

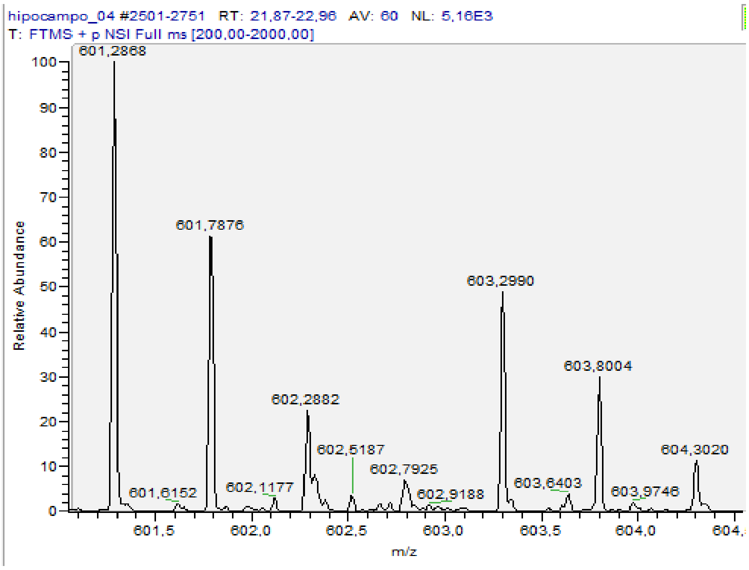

SANSNPAMAPRE 2+

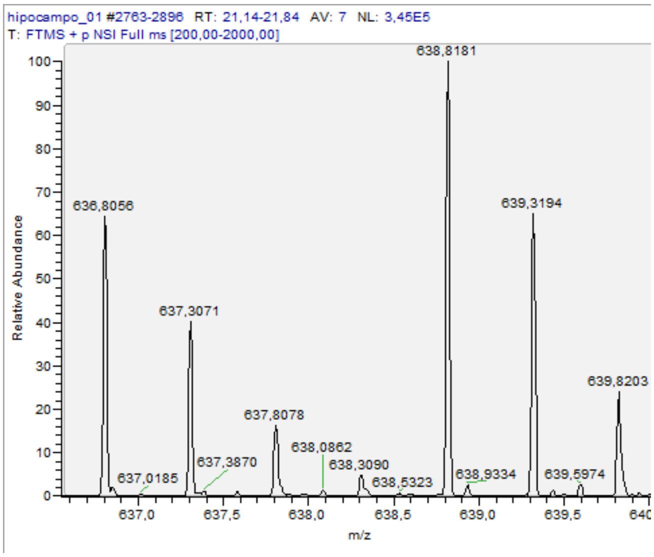

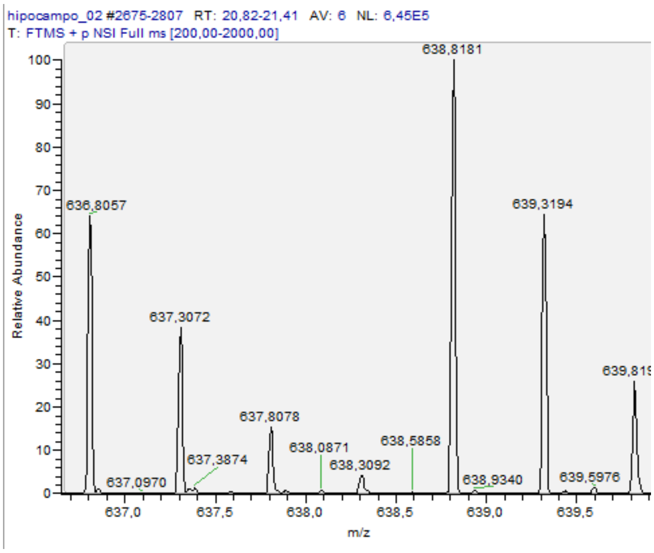

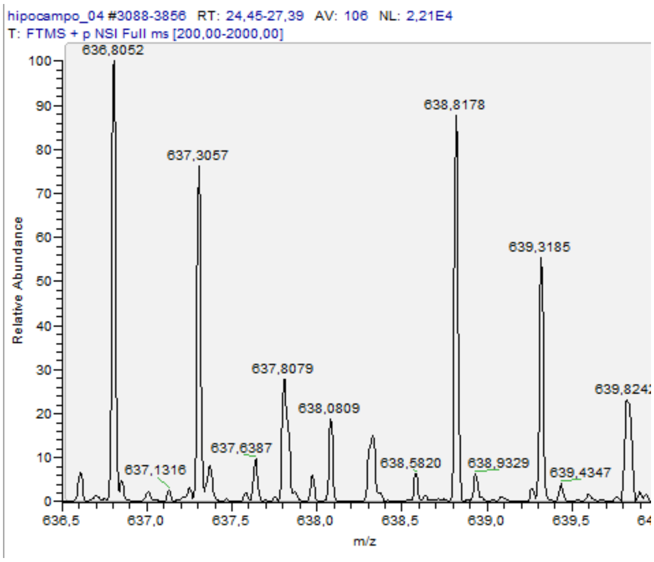

GISTPEELGLDKV 2+

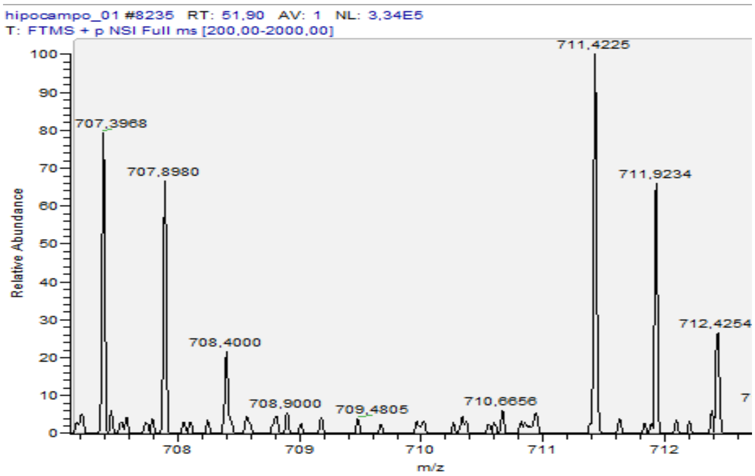

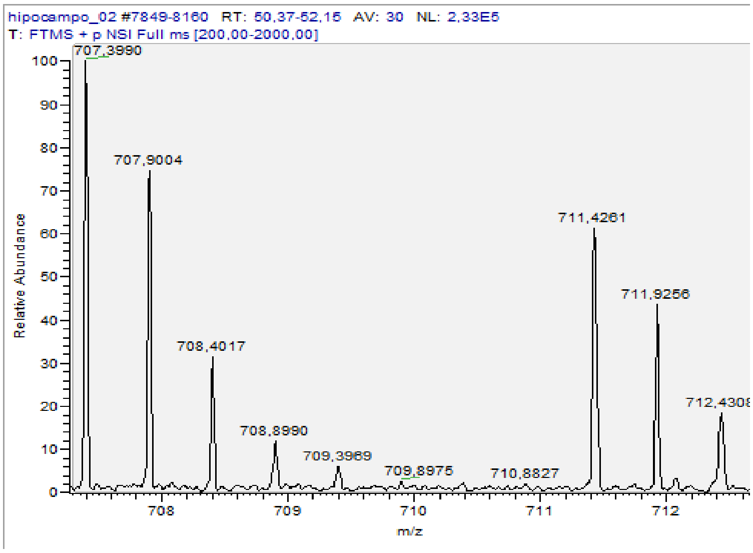

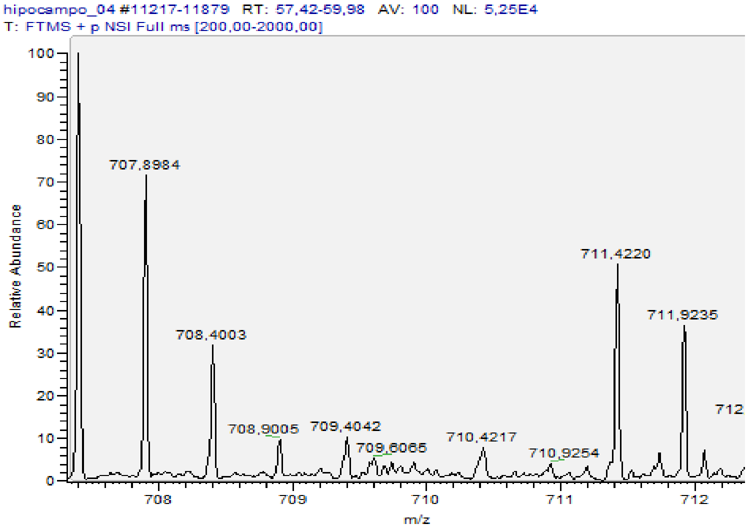

NDFASAVRILEV 2+

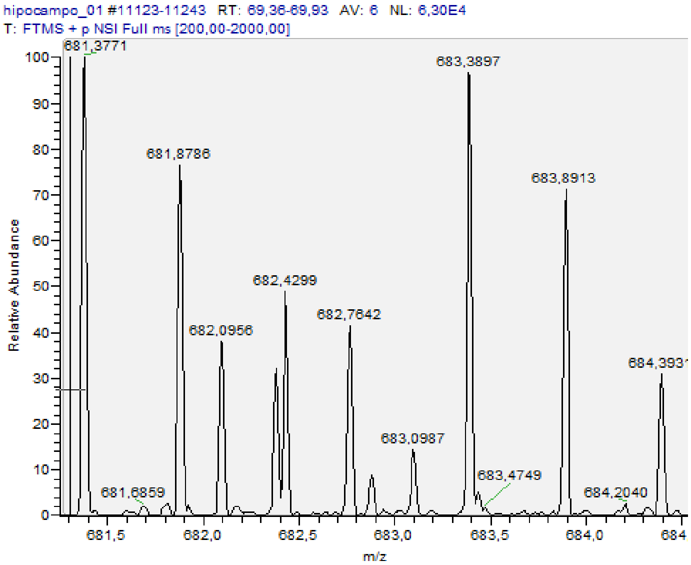

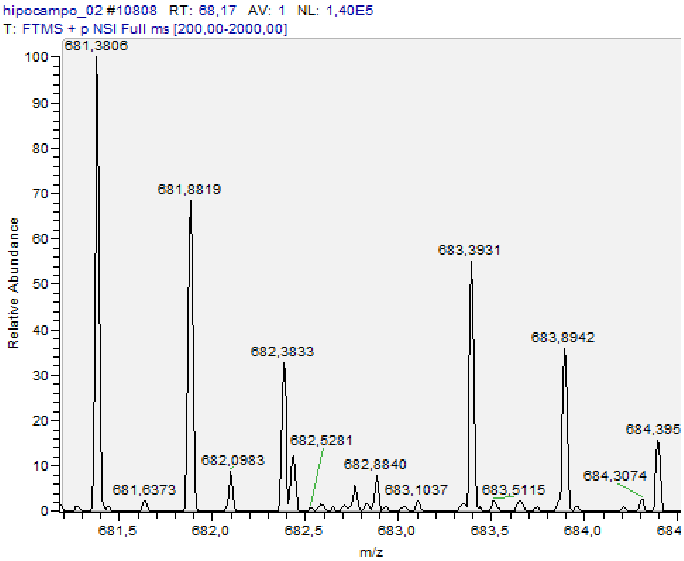

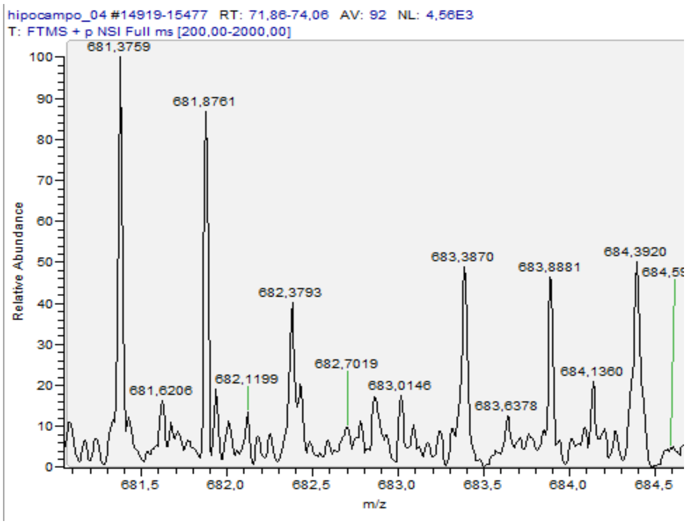

RPTLNELGISTPEELGLDKV 3+

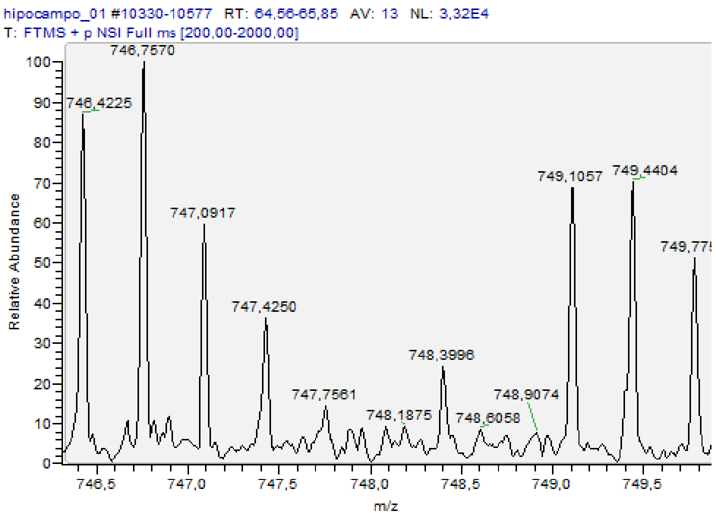

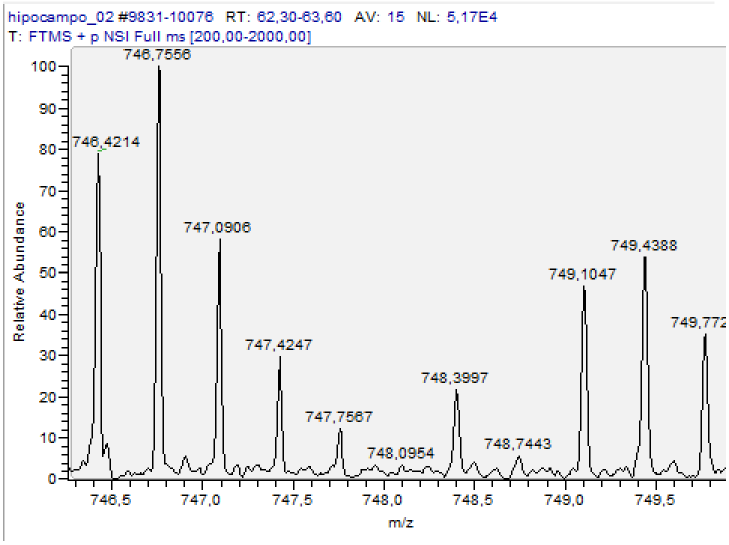

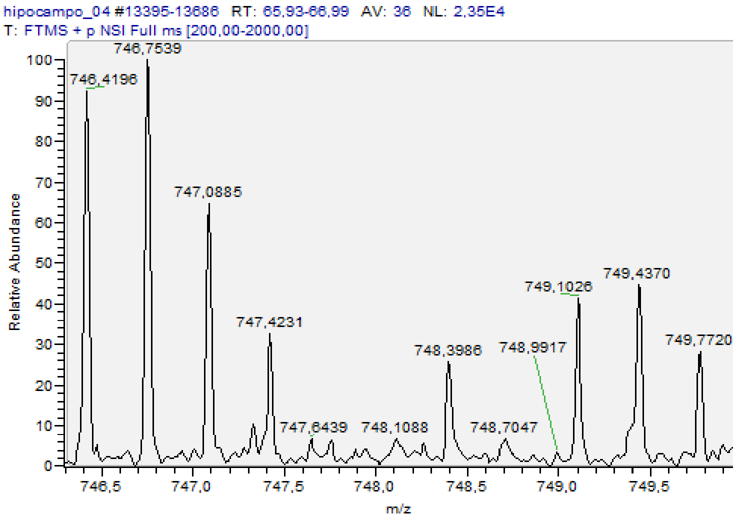

SGQAAARPLVA 2+

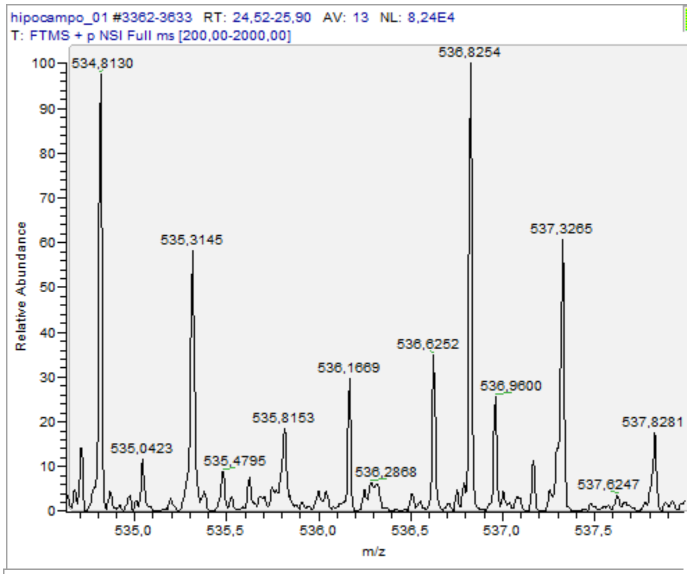

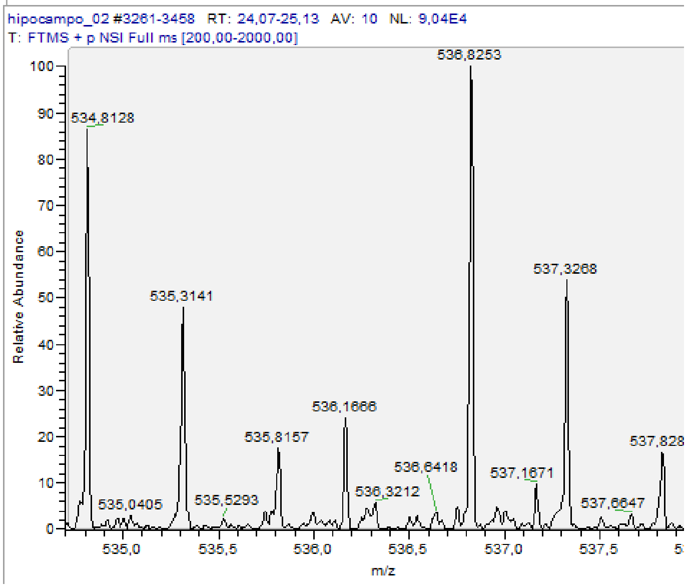

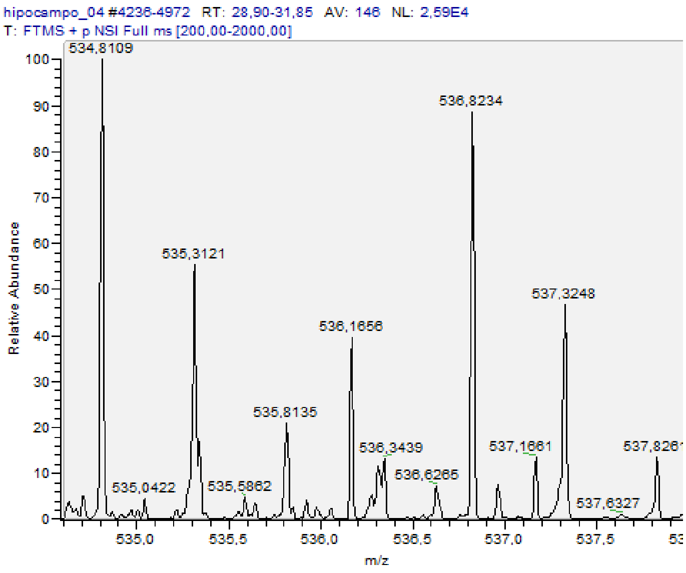

SGQAAARPLVATV 2+

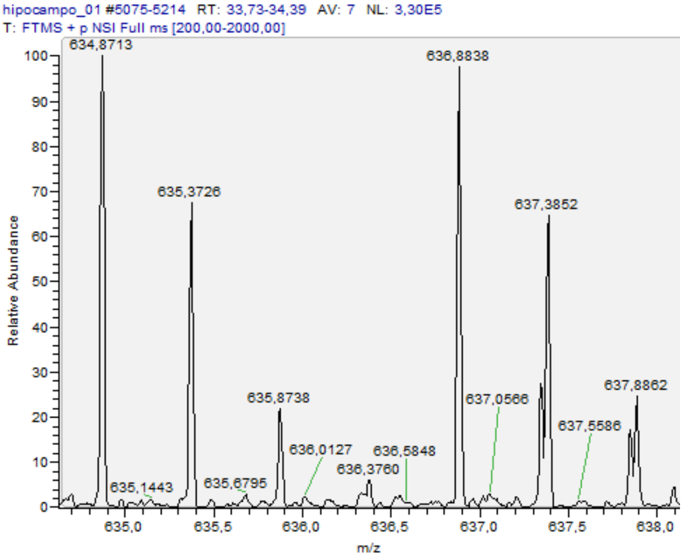

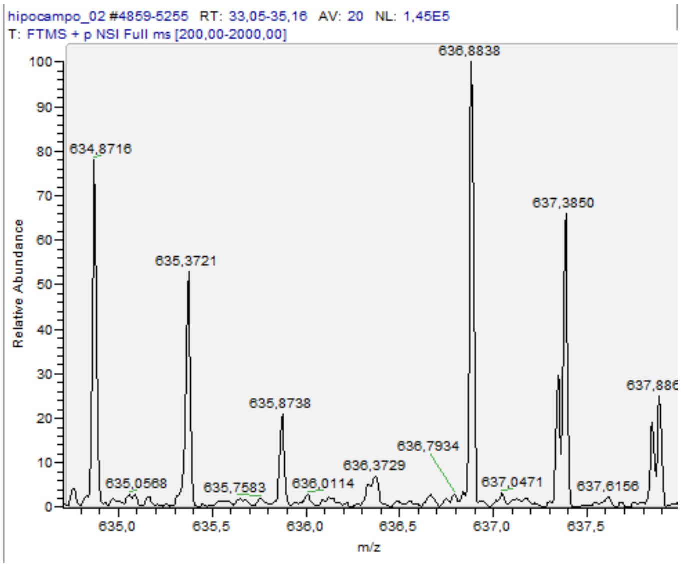

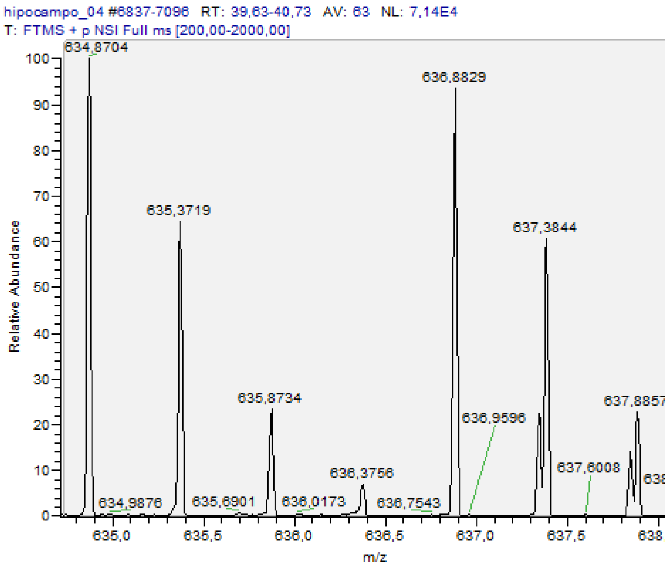

TVGLNVPASVRF 2+

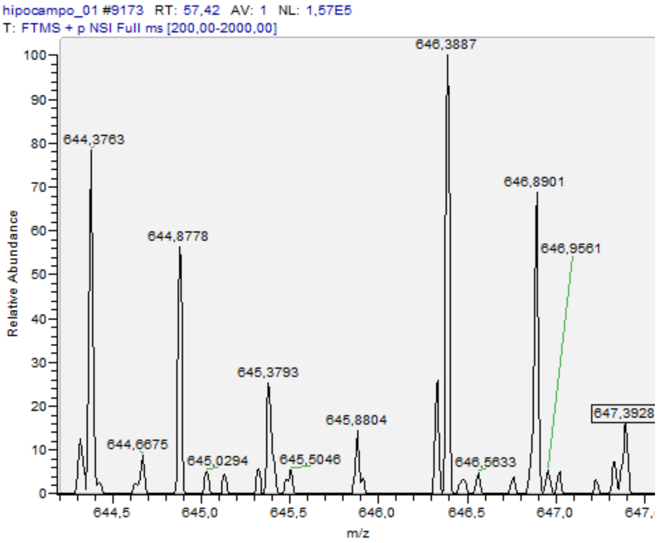

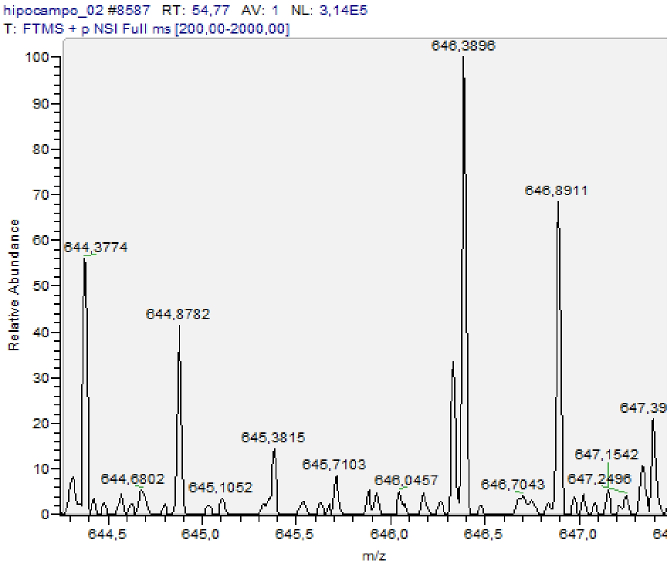

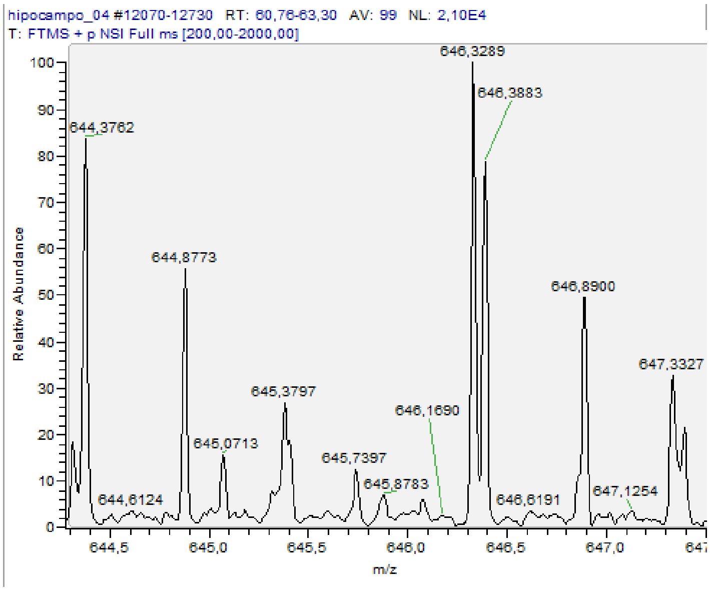

SFARAPQLDL 2+

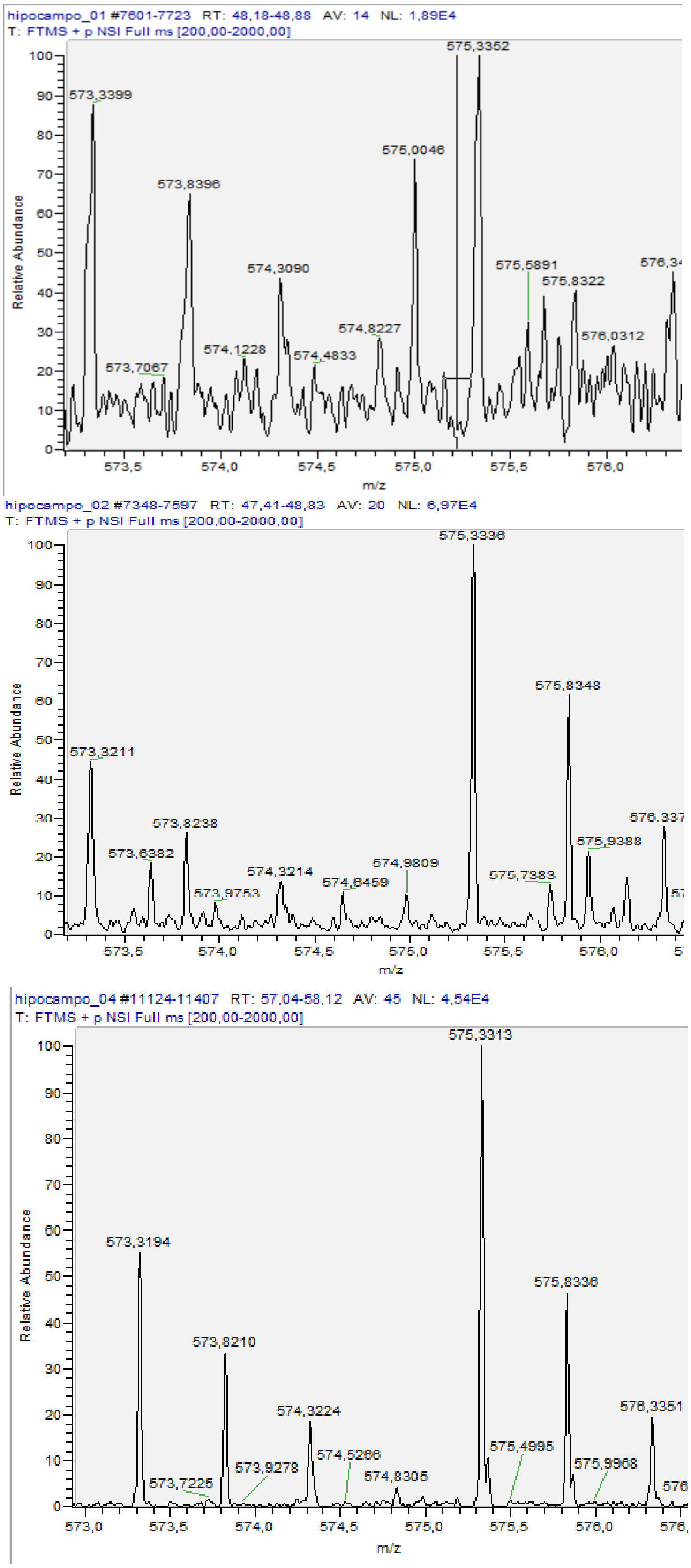

AGNSDLILPVPAFNVINGGSHAGNKL 3+

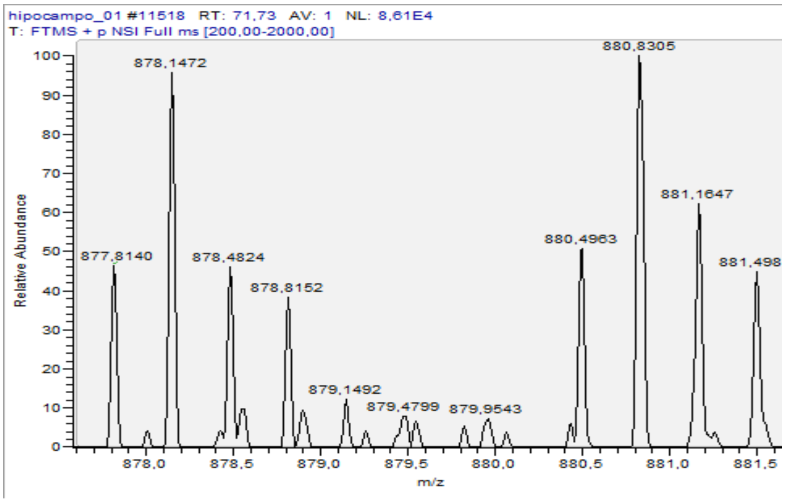

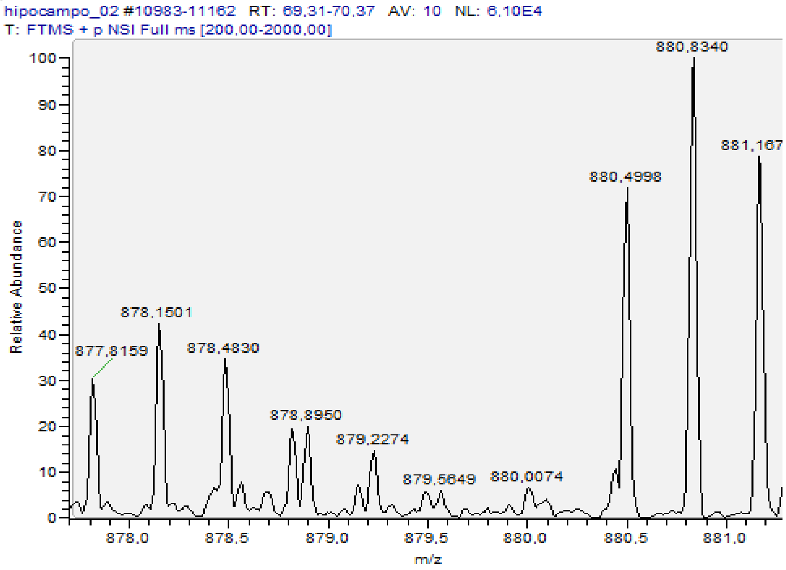

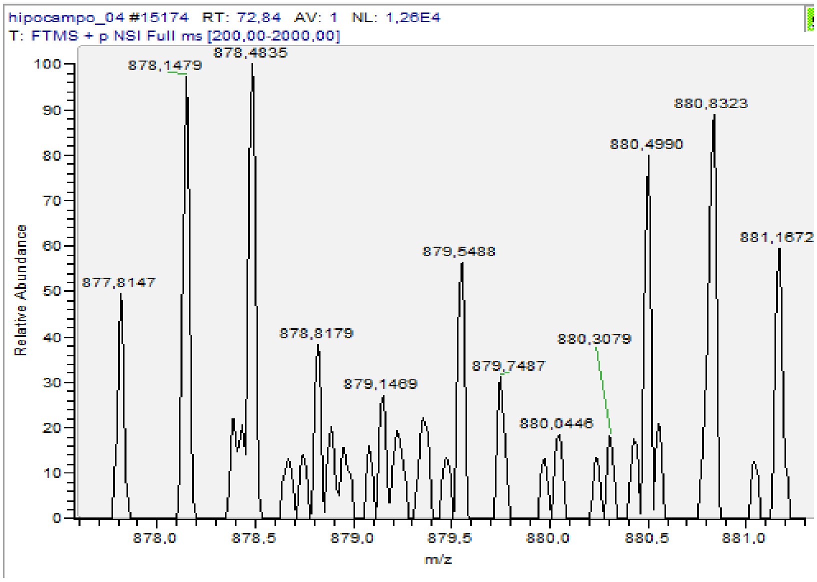

SKVAFSAVRSTN 2+

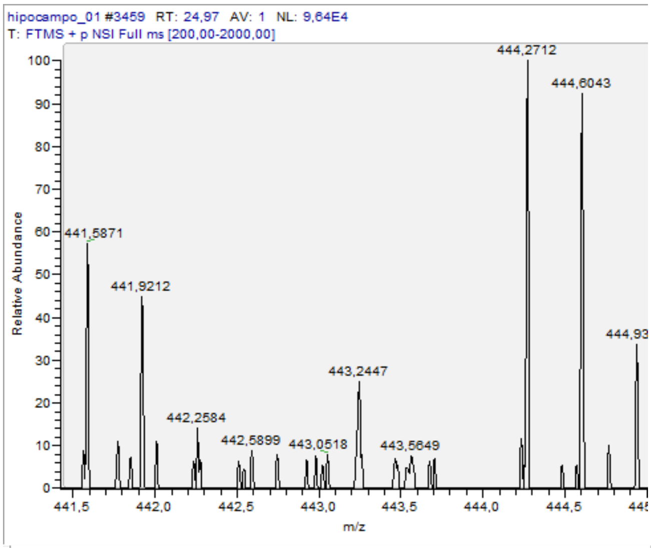

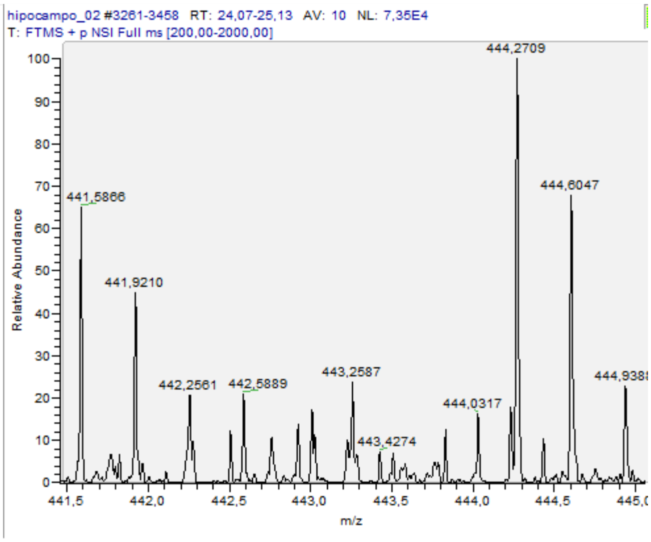

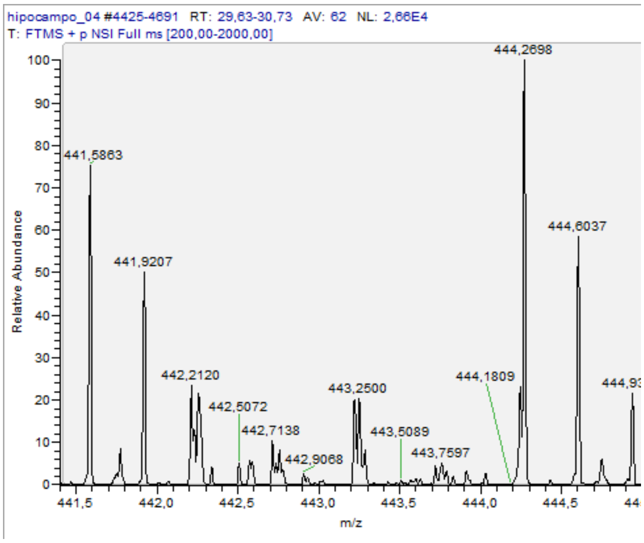

AEDVTAALAKQGL 2+

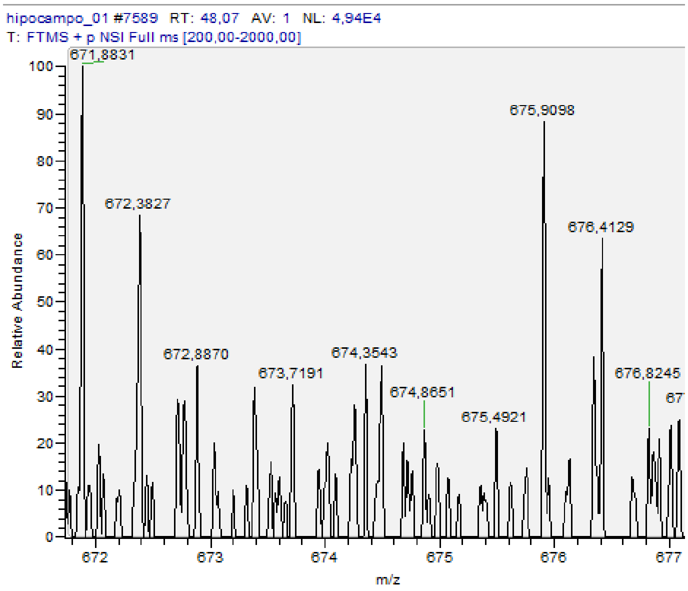

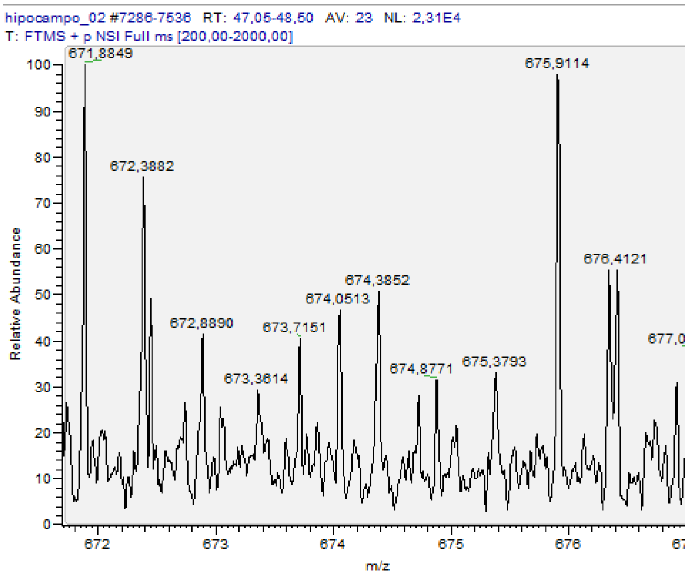

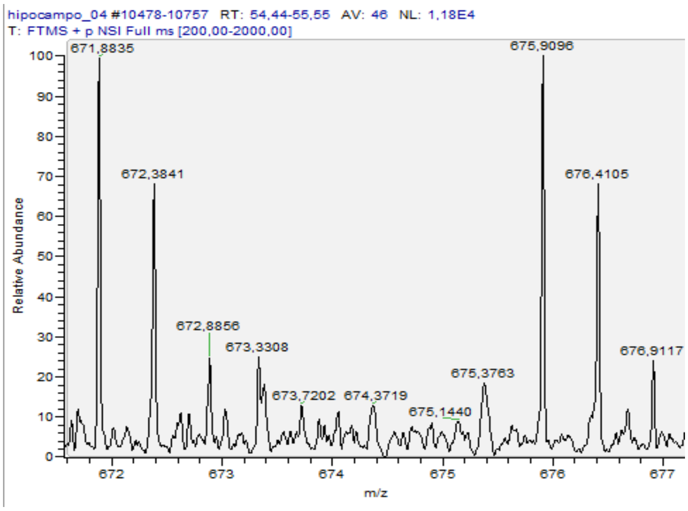

VFDVELLKLE 2+

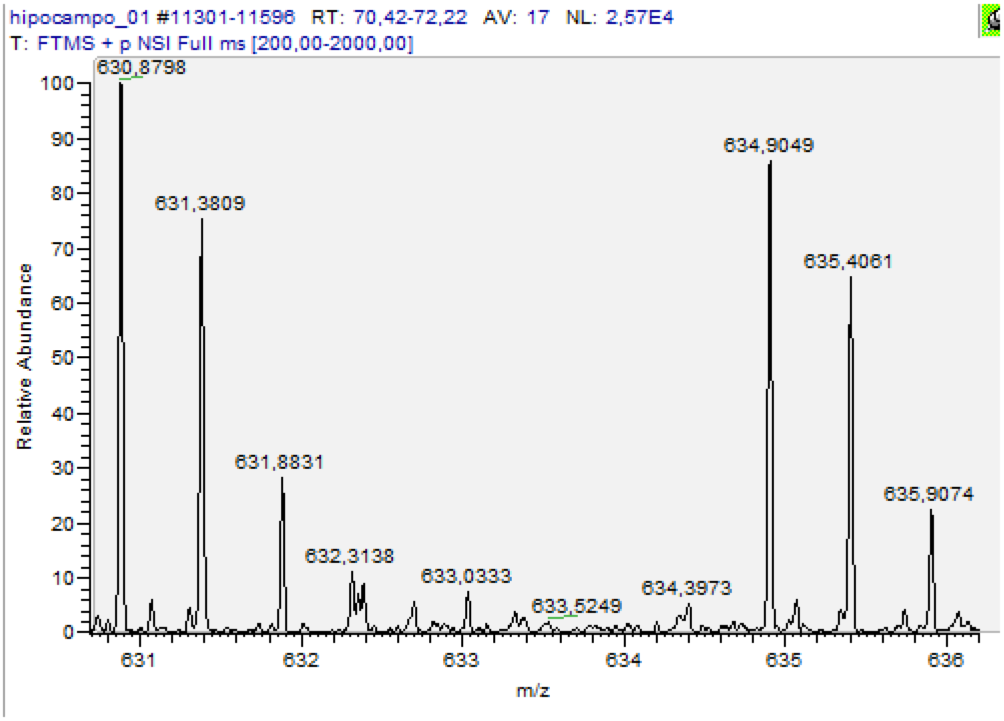

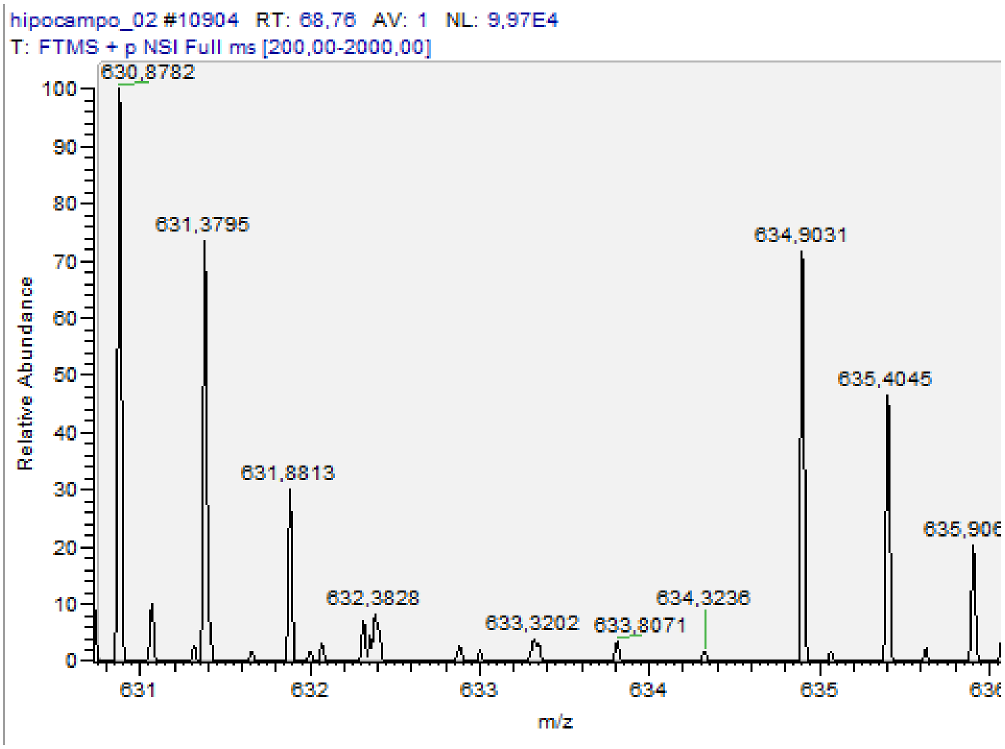

AFRVPTPNVSVVDL 2+

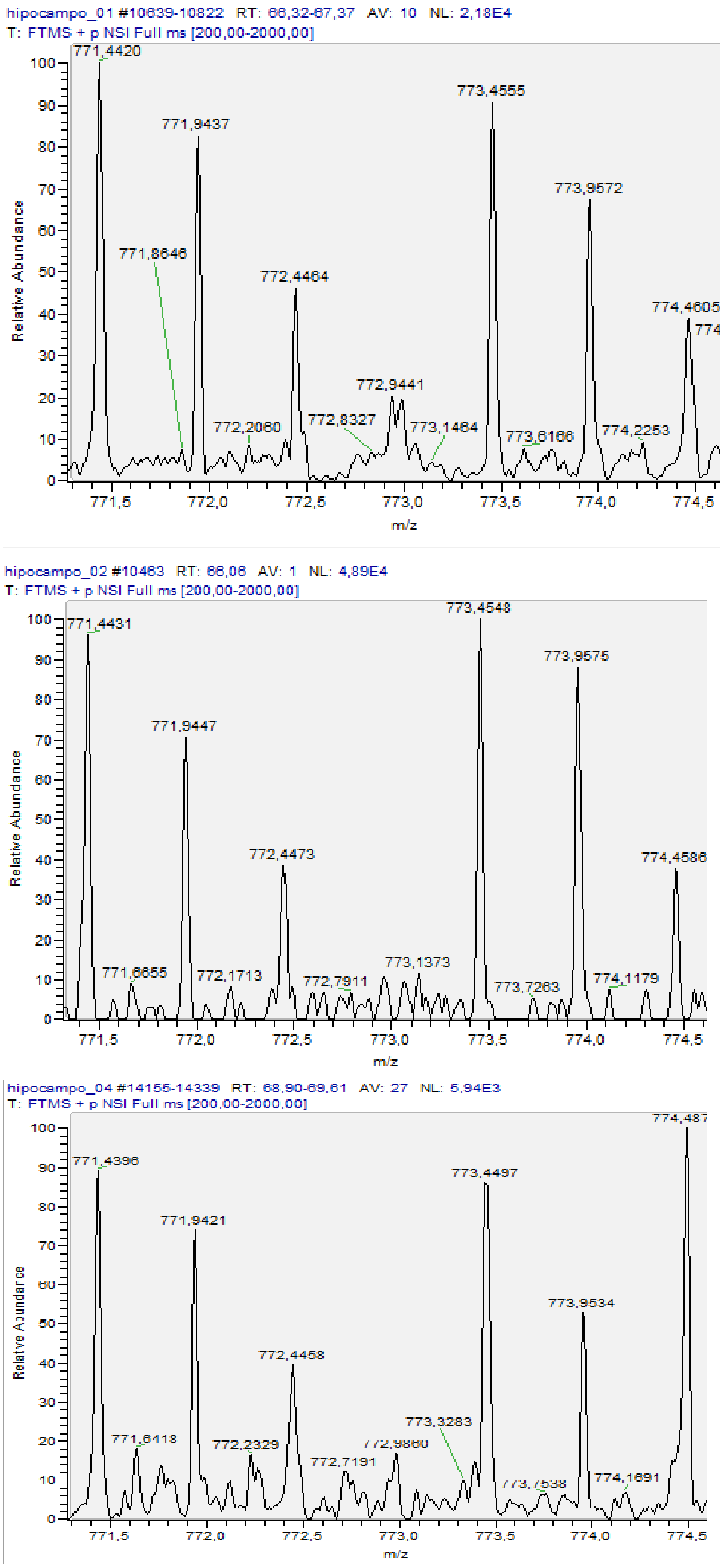

KFDDPKFEVIDKPQS 2+

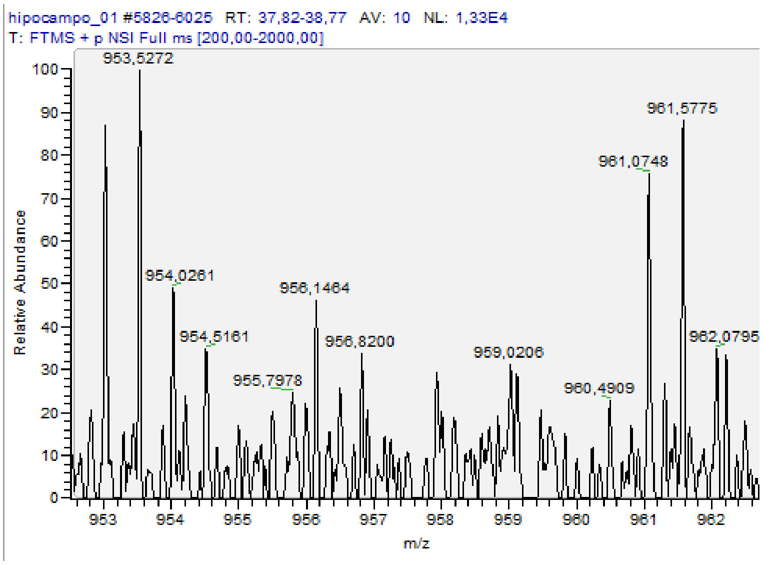

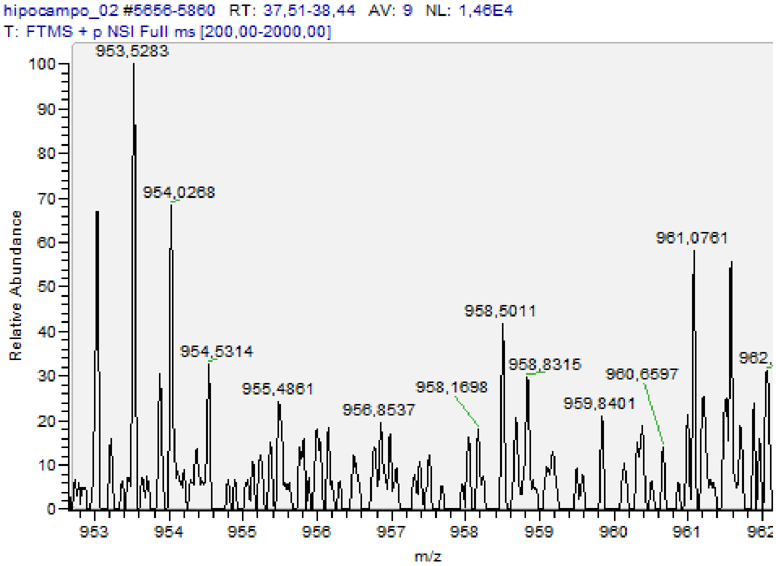

GQGAGALVHSE 2+

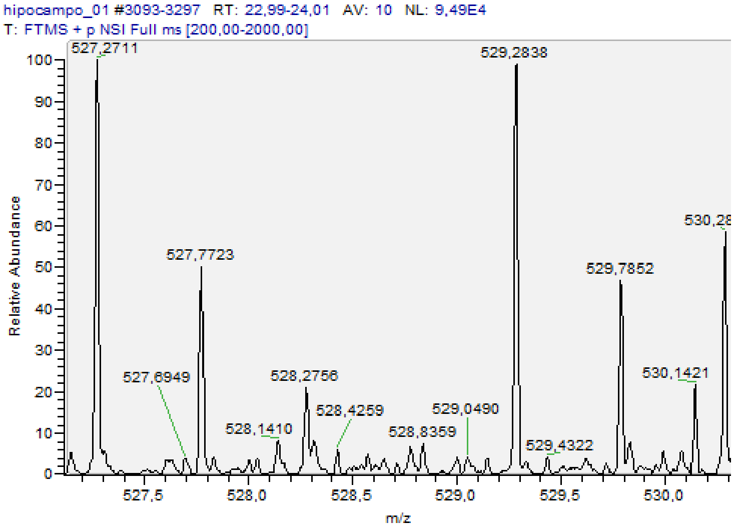

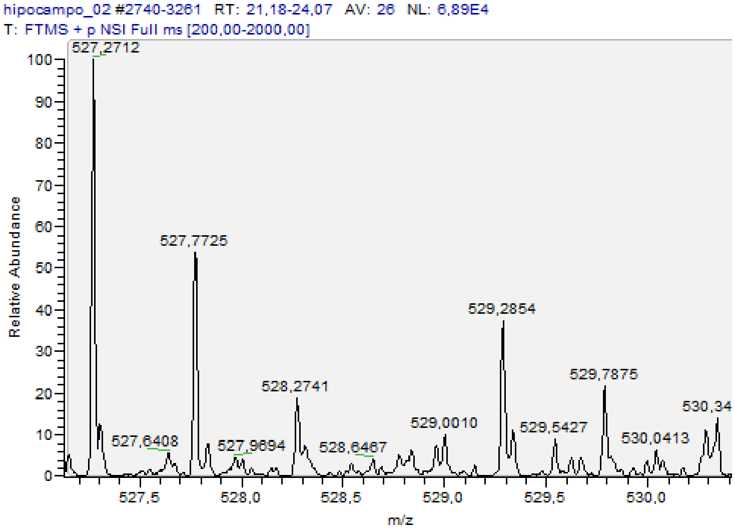

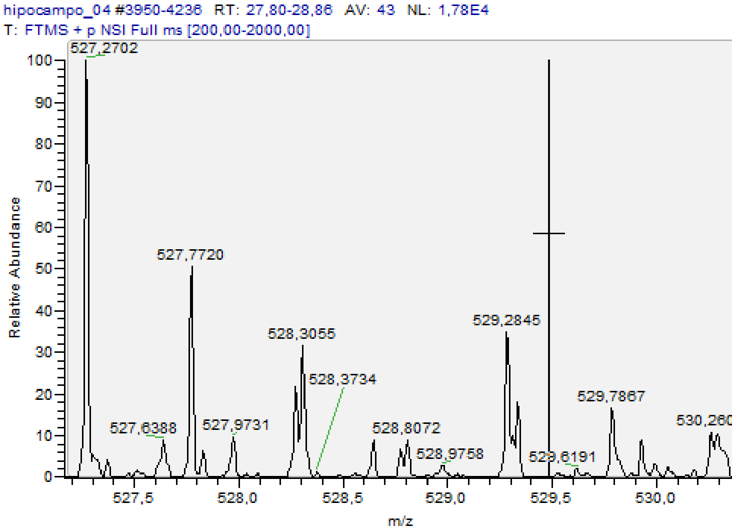

Supplement: Supplementary file 1 [file biomolecules-09-00382-s001.zip › Supp - Mascot MS-MS + labeling + quantitation.pdf]
