# Supplementary material for: Thimet Oligopeptidase (EC 3.4.24.15) Key Functions Suggested by Knockout Mice Phenotype Characterization
Source: Biomolecules. 2019 Aug 19;9(8):382. doi: 10.3390/biom9080382 (PMC6722639; doi:10.3390/biom9080382)
Supplement: Supplementary file 1 [file biomolecules-09-00382-s001.zip › Supplementary Figures 1-3 behavior studies.pdf]

Thimet oligopeptidase (EC 3.4.24.15) key functions suggested by knockout mice phenotype characterization

Dos Santos, N.B.[1#], Franco, R.D.[1#], Camarini, R.[1], Munhoz, C.D.[1], Eichler, R.A.S.[1], Gewehr, M.C.F. [1], Reckziegel, P.[1], Llanos, R.P.[1], Dale, C.S. [2], Oliveira, V.R. [2], Borges, V.F.[3], Lima, B.H.F.[3], Cunha, F.Q.[3], Visniauskas, B.[4], Chagas, J.R.[4], Tufik, S.[4]., Peres, F.F.[5], Abilio, V.C.[5], Florio, J.C.[6], Iwai, L.K.[7], Rioli, V.[7], Prezoto, B.C.[8], Guimaraes, A.O.[9], Pesquero, J.B.[9], Bader, M. [10,11,12,13,14], Castro, L.M.[15], and Ferro, E.S.[1*]

Department of [1]Pharmacology and [2]Anatomy, Biomedical Sciences Institute, and [6]Department of Pathology, Veterinarian Medical School, University of São Paulo (USP), São Paulo, 05508-000; [3]*Department of Pharmacology*, *Faculty* of *Medicine* of *Ribeirao Preto, University of São Paulo, Ribeirão Preto, SP,* 14049-900; Departments of [4]Psychobiology, [5]Pharmacology and [9]Biophysics, Federal University of São Paulo (UNIFESP), São Paulo, 04023-062; [7]Special Laboratory of Applied Toxinology (LETA), Center of Toxins, Immune Response and Cell Signaling (CETICS), [8]Pharmacology Department, Butantan Institute, 05503-000, São Paulo;[10]Max-Delbrück-Center for Molecular Medicine, D-13125, Berlin, Germany, [11]Charité - Universitätsmedizin Berlin, [12]Berlin Institute of Health (BIH), Berlin, Germany, [13]DZHK (German Center for Cardiovascular Research), Partner Site Berlin, Berlin, Germany, [14]Institute for Biology, University of Lübeck, Lübeck, Germany, [15]Biosciences Institute, São Paulo State University (UNESP), São Vicente, 11330-900; SP, Brazil.

Running Title: THOP1 knockout mice

***To whom correspondence should be addressed:** Emer S. Ferro, PhD, Professor, Av. Prof. Lineu Prestes 1524, Sala 317, São Paulo, SP, 05508-000, Brazil, Phone: +55-11-3091-7310, Fax: +55-11-3091-7322, e-mail: *eferro@usp.br*

#These two authors contributed equally

Keywords: THOP1; neurodegeneration; inflammation; sepsis; MHC-I; peptidome.

Supplementary Figure S1

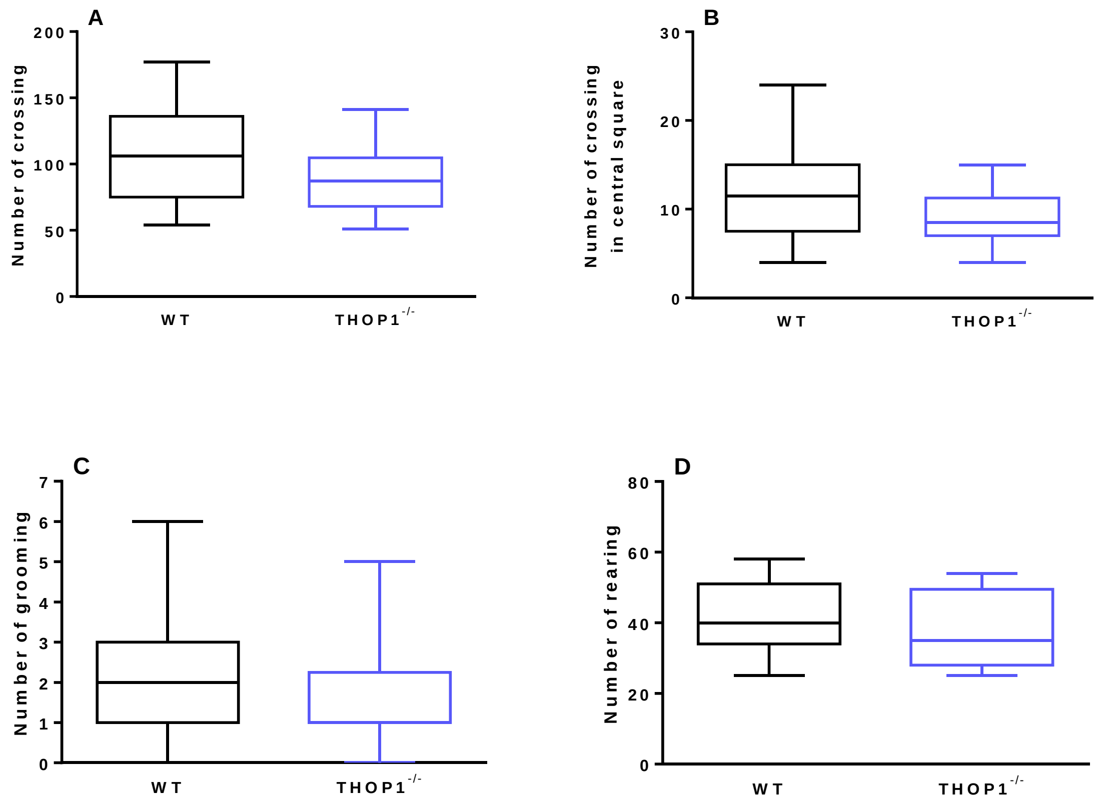

Supplementary Figure S1. Open field task. Representative images show typical examples of WT and THOP1-/- mice exploring behavior in the open field test. Each group of mice was placed in the open field apparatus and their number of crossing (A), number of crossing in the central square (B), number of grooming (C) and number of rearing (D) were measured. No difference was observed between the experimental groups. Data are presented as mean ± S.E.M. (Student's t test). WT n=23, THOP1-/- n=18.

Supplementary Figure S2

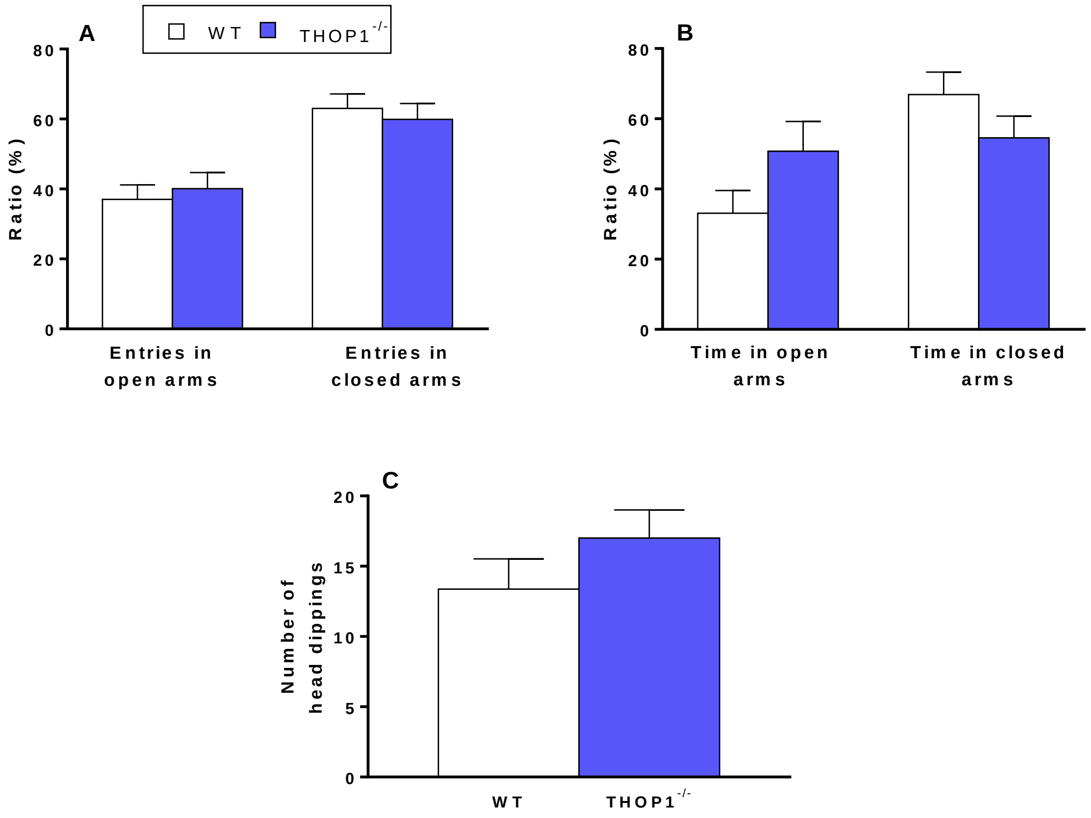

Supplementary Figure S2. Elevated plus maze task. (A) Percentage ratio of entries in open or closed arms/ (entries in both arms). (B) Percentage ratio of time spent in open or closed arms/ (time spent in both arms). (C) Number of head dippings during the test. No difference was observed between the experimental groups. Data are presented as mean ± S.E.M. (Student's *t* test). WT n=08, THOP1$^{-/-}$ n=09.

Supplementary Figure S3

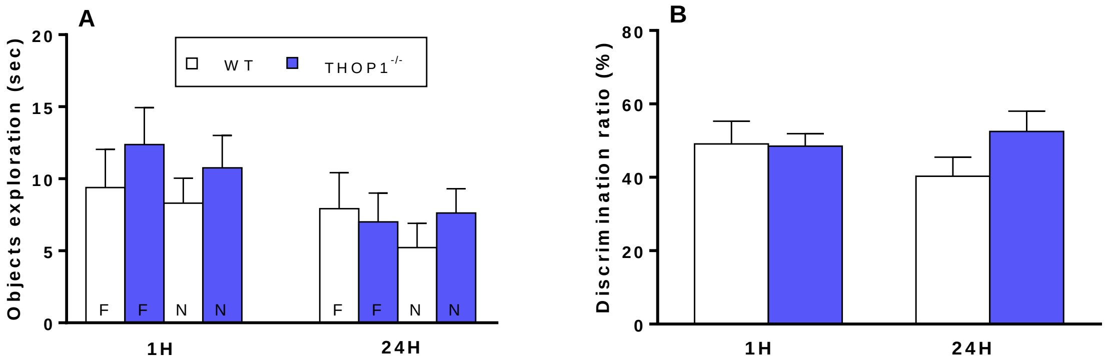

Supplementary Figure S3. Novel object recognition task. (A) The graph shows the time of exploration in familiar (F) and novel (N) objects after 1 and 24h after the training. No difference was observed among groups. **(B)** The ratio of time spent exploring the novel object by the total time spent exploring both objects was described as discrimination rate. No significance between groups. Data are presented as mean ± S.E.M. (One-way ANOVA test, Sidak's post hoc). WT n=13, THOP1$^{-/-}$ n=08.
