# Supplementary material for: Thimet Oligopeptidase (EC 3.4.24.15) Key Functions Suggested by Knockout Mice Phenotype Characterization
Source: Biomolecules. 2019 Aug 19;9(8):382. doi: 10.3390/biom9080382 (PMC6722639; doi:10.3390/biom9080382)

TNF Western blots

	TNF	B ACTINA	TNF/BACT	%Control
	Adj. Vol. (Int)	Adj. Vol. (Int)		
WT1	1.167.461,67	6.920.164,32	0,168704	0,723361
THOP1-1	2.787.342,00	6.092.992,07	0,457467	1,961501
WT2	1.013.855,33	4.471.864,00	0,226719	0,972112
THOP1-2	1.840.642,00	6.593.621,78	0,279155	1,196945
WT3	1.799.854,33	5.712.120,95	0,315094	1,351042
THOP1-3	2.417.030,33	6.702.637,46	0,360609	1,546198
WT4	798.855,67	6.139.185,54	0,130124	0,557939
THOP1-4	2.580.707,33	5.901.906,12	0,437267	1,874888
WT5	2.179.940,33	5.731.892,00	0,380318	1,630705
THOP1-5	1.843.964,67	7.554.004,07	0,244104	1,046657
WT6	1.338.808,00	7.505.435,39	0,178378	0,764841
THOP1-6	3.239.711,67	6.636.660,44	0,488154	2,093079
Average Control	0,233222867			

Hippocampus

TNF

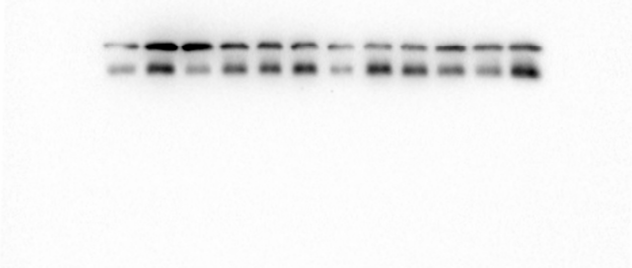

Beta actin (BACT)

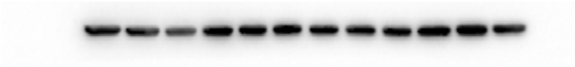

TNF Western blots

	TNF	B ACTINA	TNF/BACT	%Control
	Adj. Vol. (Int)	Adj. Vol. (Int)		
WT1	2.064.798,93	4.970.143,40	0,41544051	0,54378866
THOP1-1	6.181.236,88	6.011.798,14	1,02818437	1,34583648
WT2	4.727.962,84	6.493.477,53	0,72810953	0,9530551
THOP1-2	2.495.862,67	5.017.280,79	0,49745326	0,6511388
WT3	3.405.637,53	4.699.611,40	0,72466365	0,94854464
THOP1-3	4.730.476,58	6.307.893,55	0,74992968	0,98161648
WT4	7.023.078,84	6.937.872,10	1,01228139	1,32502036
THOP1-4	8.556.587,35	6.318.050,93	1,35430807	1,77271437
WT5	4.222.308,26	5.626.075,58	0,75048908	0,9823487
THOP1-5	7.064.656,05	5.500.711,56	1,28431676	1,68109962
WT6	5.857.025,00	6.146.777,08	0,95286114	1,24724254
THOP1-6	9.911.958,84	6.654.739,83	1,48945851	1,94961882

Average Contr 0,76

Medula (spinal cord)

TNF

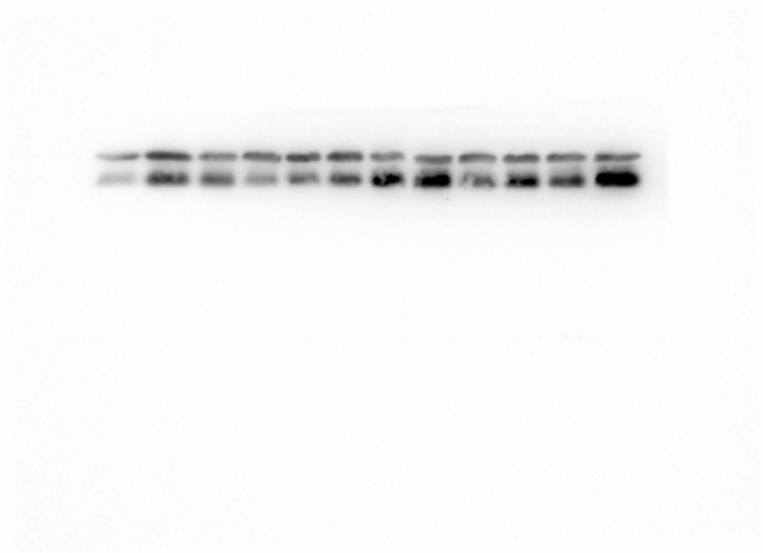

Beta actin (BACT)

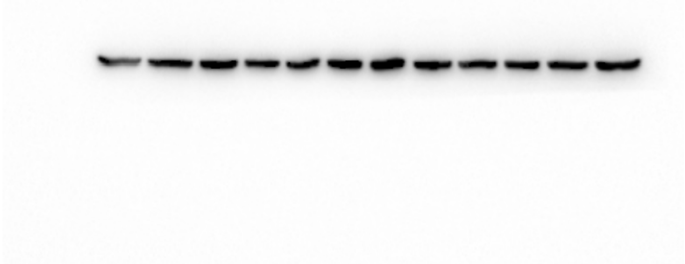

Supplement: Supplementary file 1 [file biomolecules-09-00382-s001.zip › TNF Western blots crude data and membranes.pdf]
