# Supplementary material for: Thimet Oligopeptidase (EC 3.4.24.15) Key Functions Suggested by Knockout Mice Phenotype Characterization
Source: Biomolecules. 2019 Aug 19;9(8):382. doi: 10.3390/biom9080382 (PMC6722639; doi:10.3390/biom9080382)
Supplement: Supplementary file 1 [file biomolecules-09-00382-s001.zip › Trap THOP1 Knockout generation Supp.pdf]

Thimet oligopeptidase (EC 3.4.24.15) key functions suggested by knockout mice phenotype characterization

Dos Santos, N.B.[1#], Franco, R.D.[1#], Camarini, R.[1], Munhoz, C.D.[1], Eichler, R.A.S.[1], Gewehr, M.C.F. [1], Reckziegel, P.[1], Llanos, R.P.[1], Dale, C.S. [2], Oliveira, V.R. [2], Borges, V.F.[3], Lima, B.H.F.[3], Cunha, F.Q.[3], Visniauskas, B.[4], Chagas, J.R.[4], Tufik, S.[4]., Peres, F.F.[5], Abilio, V.C.[5], Florio, J.C.[6], Iwai, L.K.[7], Rioli, V.[7], Prezoto, B.C.[8], Guimaraes, A.O.[9], Pesquero, J.B.[9], Bader, M. [10,11,12,13,14], Castro, L.M.[15], and Ferro, E.S.[1*]

Department of [1]Pharmacology and [2]Anatomy, Biomedical Sciences Institute, and [6]Department of Pathology, Veterinarian Medical School, University of São Paulo (USP), São Paulo, 05508-000; [3]*Department of Pharmacology*, *Faculty* of *Medicine* of *Ribeirao Preto, University of São Paulo, Ribeirão Preto, SP,* 14049-900; Departments of [4]Psychobiology, [5]Pharmacology and [9]Biophysics, Federal University of São Paulo (UNIFESP), São Paulo, 04023-062; [7]Special Laboratory of Applied Toxinology (LETA), Center of Toxins, Immune Response and Cell Signaling (CETICS), [8]Pharmacology Department, Butantan Institute, 05503-000, São Paulo;[10]Max-Delbrück-Center for Molecular Medicine, D-13125, Berlin, Germany, [11]Charité - Universitätsmedizin Berlin, [12]Berlin Institute of Health (BIH), Berlin, Germany, [13]DZHK (German Center for Cardiovascular Research), Partner Site Berlin, Berlin, Germany, [14]Institute for Biology, University of Lübeck, Lübeck, Germany, [15]Biosciences Institute, São Paulo State University (UNESP), São Vicente, 11330-900; SP, Brazil.

Running Title: THOP1 knockout mice

***To whom correspondence should be addressed:** Emer S. Ferro, PhD, Professor, Av. Prof. Lineu Prestes 1524, Sala 317, São Paulo, SP, 05508-000, Brazil, Phone: +55-11-3091-7310, Fax: +55-11-3091-7322, e-mail: eferro@usp.br

#These two authors contributed equally

Keywords: THOP1; neurodegeneration; inflammation; sepsis; MHC-I; peptidome.

Supplementary information for Trap THOP1 knockout generation

Introduction

Thimet Oligopeptidase (THOP1) gene trap knockout mouse strain CSG163 was generated by C57Bl/6 blastocist micro-injection of genetically modified Embryonic Stem Cells (129ola) obtained from Baygenomics through the International Gene Trap Consortium (IGTC, http://www.genetrap.org/).

Results

The genetically modified INTRON allele of CSG163 strain have the gene trap vector pGT0Lxf inserted at THOP1 intron 5 (Figure 1), as evidenced by sequencing 5'RACE cDNA from the original CSG163 ES cell line (DX812807), used to generate this mice strain (The detailed reports for this ES Cell line can be found at the following web link: http://www.genetrap.org/cgi-bin/annotation.py?cellline=CSG163&type=detail)

Based in this ES cell line 5'RACE cDNA sequences, the gene trap vector sequence and the genomic and mRNA sequences for the THOP1 gene we designed primers and performed RT-PCR, PCR and sequencing experiments to confirm the expected transgenic mRNA and to map the genomic site of the gene trap vector integration in the THOP1 genomic *locus*, as described below.

We also collected tissues from wild type and homozygous gene trap mutant CSG163 mice and verified by western blot, with a THOP1 catalytic site commercial antibody, the absence of the 78.8 KDa predicted THOP1 protein only in the studied tissues of homologous CSG163 mutants.

Taken together, these results present strong evidence that the CSG163 mice strain is a correct THOP1 gene trap knockout strain and can be very useful in studies of this gene biological function.

Confirming by RT- PCR the hybrid mRNA formed by the 5' region of THOP1 mRNA fused to vector derived Beta-Geo mRNA sequence

In order to check the presence of the THOP1 wild type (WT) mRNA and the expected Transgenic mRNA resulting from the Gene trap vector insertion in the THOP1 *locus,* hereafter referred as THOP1-Beta-Geo, we designed primers (additional information 1) and performed RT-PCRs on total RNA obtained from tail of seven CSG163 mice .

We were able to PCR amplify from the cDNA of some of the samples the expected 200bp and 449bp fragments in two different reactions for the transgenic THOP1-Beta-Geo mRNA (Figure 1). These amplicons were purified and confirmed by sequencing (additional information 2). Therefore, we concluded that the CSG163 mice strain have the described gene trap insertion in the THOP1 *locus, and* the THOP1-Beta-Geo mRNA was expressed in the tail tissues.

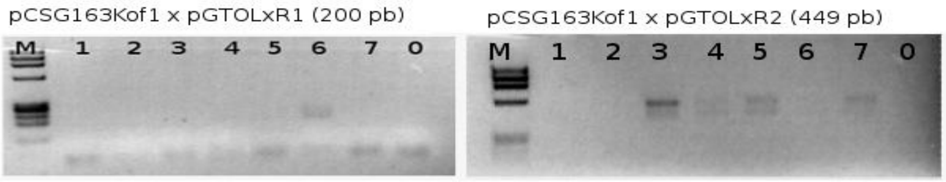

Figure 1. RT-PCR analysis of CGS163 mice tail total RNA. Negative picture of 2% agarose gels stained with ethidium bromide and visualized under UV light after electrophoresis of PCRs performed with the indicated primers on 1/10 dilution of cDNAs obtained from 3 micro-gram of Tail Total RNA using the reverse transcriptase M-MLV (Invitrogen) of several animals, Lanes 1 to 7. Lanes M are the DNA marker X174 RF DNA/Hae III Fragments (Invitrogen).

Mapping the genomic gene trap insertion position on the modified THOP1 allele of the CSG163 mice strain

We were able to PCR amplify from CSG163 heterozygotes mouse tail genomic DNA (gDNA) the expected a DNA fragment of about 2.6 kb using the forward primer pCSG163KOf1 with the reverse primer pGT0LxR2 (Figure 2).

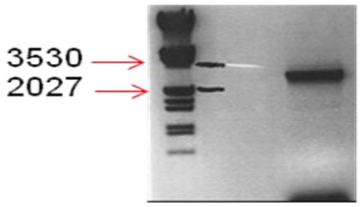

Figure 2. Product of the long-range PCR. This reaction was done using forward prime pCSG163KoF1, that anneals on the exon 5 of mouse THOP1 gene, and reverse prime pGTOLxR2, that anneals on gene trap vector inserted. Negative picture of 0.7% agarose gel stained with ethidium bromide and visualized under UV light after electrophoresis stained with bromide ethidium. Lane 1: Lambda DNA/EcoRI+HindIII Marker, 3 (Fermentas); lane 2- 2,600 pb amplicon of CSG gDNA gene trap insertion region.

Sequencing this fragment showed that the gene trap vector inserted 438 nucleotides downstream of the THOP1 exon 5/intron 5 boundary (Additional information 3). This information allowed us to design primers for gDNA based PCR genotyping of the mice from the CSG163 strain (Figure 3), and define the best protocol for genotyping that is summarized at Table 1.

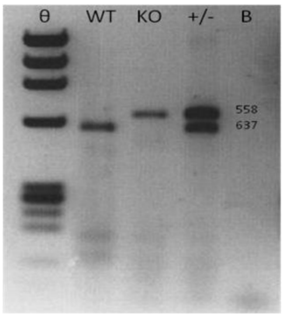

Figure 3. Product of PCR using tree primes and gDNA extract from tails. Negative picture of 2% agarose gel stained with ethidium bromide and visualized under UV light after electrophoresis stained with bromide ethidium. Lane 1: □X174 RF DNA/Hae III Fragments (Invitrogen); lane 2: WT 558 bp amplicon; lane 3: KO 637 bp amplicon; lane 4 Heterozygous amplicons and lane 5 PCR control without template.

Table 1: Protocol for gDNA genotyping

Animal			*strain name*	*THOP1 CSG163 -/-*
X	*mouse*		*backgroud*	*129/C57BL/6*

Primers	*name*	*sequence*	*stock-conz*	*dilution*	*working*
**fw**	pCSG163KOf1	5'-TCGACTTCAACAAGAACCTC-3'	**100 ng/uL**	10 x	10 ng/uL
**rev 1**	pCSG163wtr1	5'-TGCCTTCTCCAGAGAGCTC-3'	**100 ng/uL**	10 x	10 ng/uL
**rev 2**	CSGKOR2	5'-GACGGTCACAGCTTGTCTG-3'	**100 ng/uL**	10 x	10 ng/uL

PCR-protocol to genotype:

	**x1 µl**	**25 µl**
**10x PCR Buffer**	**2,50**	68,75
**dNTP (10 or 5 mM)**	**1,25**	34,38
**MgCl2**	**0,75**	20,63
**Primer fw**	**0,50**	13,75
**Primer rev1**	**0,10**	2,75
**Primer rev2**	**0,50**	13,75
**Taq polimerase**	**0,20**	5,50
**H20**	**18,20**	500,50
**DNA**	**1,00**	
**Master Mix**	**24,00**	660,00
**Total Vol.**	**25,00**	

PCR-program

95 oC	5 min	
**95 oC**	**15 sec**	
**60 oC**	**15 sec**	**x 45**
**72 oC**	**30 sec**	
72 oC	3 min	
10 oC	for ever	

Size of Product:

WT amplicon 558 bp

KO amplicon 637 bp

Agarose Gel: 2%

The resulting DNA fragments were once more sequenced and confirmed (additional information 4).

Furthermore, the sequencing results confirmed that there was a recombination of gene trap vector sequences that could have occurred even before ES cell transfection. The gene trap vector pGT0Lxf sequence just upstream of the Beta-Geo gene is common to the antisense vector sequence of part of the ampicilin resistance gene, which probable facilitated this event.

Additional information 1: nucleotide sequences of the cDNA region of the THOP1 and THOP1-Beta-Geo transgenic mRNAs used to design RT-PCR primers.

PRIMERS sequences:

pGT0LxR1: 5' CTCTAGAGTCCAGATCTGCG 3'
pGT0LxR2: 5'TGTAGATGGGCGCATCGTA 3'
pCSG163KOf1: 5'TCGACTTCAACAAGAACCTC 3'
pCSG163wtr1: 5'TGCCTTCTCCAGAGAGCTC 3'

The cDNA nucleotide sequence of the 5' region of the wild type THOP1 mRNA (NM 022653.4):

5'ATGAAGCCCCCCGCAGCCTGTGCTGGGGACGTGGTGGATGCAGCATCCCCAGCCTCCACCGTGAATCATC
TTCGCTGGGATCTGAGCGCACAGCAGATCAGGGCTCTAACCACGCAGCTCATCGAGCAGACCAAGTGTGTGT
ACGACCGAGTGGGTGCTCAGAACTTCGAGGATGTGTCGTATGAGAGCACACTGAAGGCACTGGCTGACGTGG
AGGTCACCTACACAGTGCAGAGGAACATTCTCGACTTCCCCCAACACGTGTCCCCGTGCAAGGACATCCGTG
CAGCCAGCACAGAGGCTGACAAGAAGCTCTCGGAGTTTGATGTAGAGATGAGCATGAGACAGGACGTGTACC
AGAGGGTCGTGTGGCTGCAGGAGAAAACCCCGAAAGATTCCCTGAAGCCTGAAGCAGCTCGCTACCTGGAGC
GGCTCATCAAGCTGGGCCGGAGAAACGGCCTCCACTTACCTCAGGACACACAAGAGAAGATCAAGAACATCA
AGAAGAGGCTGAGCCTGCTGTGCATCGACTTCAACAAGAACCTCAACGAGGACACCACCTTCCTGCCCTTCA
CAAGAGAGGAGCTGGGTGGGCTCCCGGAGGACTTTCTGAGCTCTCTGGAGAAGGCAGAGGACGGCAAGCTGA
AGGTCACCCTCAAGTACCCTCACTACTTCCCGCTGTTGAAGAAGTGCCATGTGCCGGAGACACGACGCCTGT
TGGAGGAGGCCTTCAACTGTCGCTGCAAGGAGGAGAACTGTGCCA 3'

Legend:
Highlighted in green – the sequence of forward primer pGSG163Kof1,
Highlighted in blue – the sequence that reverse primer pGSG163WTR1 aneals.

The cDNA nucleotide sequence of the 5' region of the expected THOP1-Beta-Geo transgenic mRNA (CSGri), defined on the basis of the gene trap vector pGT0Lxf and the 5'RACE gene trap cDNA (DX812807) sequences:

CSGri:
**GCGTGAGACCAGAGGAGGGCCGTCGCCGCCTCGCGCAGCCGACCGCCATGAAGCCCCCCGCAGCCTGTGC
TGGGGACGTGGTGGATGCAGCATCCCCAGCCTCCACCGTGAATCATCTTCGCTGGGATCTGAGCGCACAGCA
GATCAGGGCTCTAACCACGCAGCTCATCGAGCAGACCAAGTGTGTGTACGACCGAGTGGGTGCTCAGAACTT
CGAGGATGTGTCGTATGAGAGCACACTGAAGGCACTGGCTGACGTGGAGGTCACCTACACAGTGCAGAGGAA
CATTCTCGACTTCCCCCAACACGTGTCCCCGTGCAAGGACATCCGTGCAGCCAGCACAGAGGCTGACAAGAA
GCTCTCGGAGTTTGATGTAGAGATGAGCATGAGACAGGACGTGTACCAGAGGGTCGTGTGGCTGCAGGAGAA
AACCCCGAAAGATTCCCTGAAGCCTGAAGCAGCTCGCTACCTGGAGCGGCTCATCAAGCTGGGCCGGAGAAA
CGGCCTCCACTTACCTCAGGACACACAAGAGAAGATCAAGAACATCAAGAAGAGGCTGAGCCTGCTGTGCAT
CGACTTCAACAAGAACCTCAACGAGGACACCACCTTCCTGCCCTTCACAAGAGAGGAGCTGG**AAACGCTGGT
GAAAGTAAAAGATGCTGAAGATCAGTTGGGTGCACGAGTGGGTTACATCGAACTGGATCTCAACAGC**GGTCC
CAGGTCCCGAAAACCAAAGAAGAAGAAC**GCAGATCGCATCGATAACTTCGTATAGCATACATTATACGAAGT
TATCGCAGATCTGGACTCTAGAGGATCCCGTCGTTTTACAACGTCGTGACTGGGAAAACCCTGGCGTTACCC
AACTTAATCGCCTTGCAGCACATCCCCCTTTCGCCAGCTGGCGTAATAGCGAAGAGGCCCGCACCGATCGCC
CTTCCCAACAGTTGCGCAGCCTGAATGGCGAATGGCGCTTTGCCTGGTTTCCGGCACCAGAAGCGGTGCCGG
AAAGCTGGCTGGAGTGCGATCTTCCTGAGGCCGATACTGTCGTCGTCCCCTCAAACTGGCAG**ATGCACGGTT
ACGATGCGCCCATCTACA**

Legend:
Blue bold letters - sequence corresponding to the 5'region of THOP1 mRNA up to the end of exon 5,
hightlighted in green – the sequence of forward primer pGSG163Kof1

yellow highlighted black letters - the 7897 to 7977 nucleotide sequence of the gene trap vector pGTOLxf in the antisense orientation that resulted from a non documented recombination event,
In green letters - gene Trap vector sequence corresponding to the end of the intronic region, splice acceptor, the initial exon 2 nucleotides of the mouse engrailed 2 gene,
In black letters – pGT0Lxf vector sequences, were: underlined is a Lox site, in bold is the initial coding sequence of Beta-Geo
Highlighted in blue – the sequence that reverse primer pGTLxR1 aneals,
Highlighted in dark blue – the sequence that reverse primer pGTLxR2 aneals.

Additional information 2: Sequence analysis of the mice CSG163 strain Tail RT-PCR obtained DNA fragments

Comparison of the nucleotide sequences obtained by RT-PCR and the same cDNA region of the THOP1 and THOP1-Beta-Geo transgenic mRNAs used to design RT-PCR primers as shown in additional file 1.

Sequencing results for the transgenic 200bp cDNA Fragment (KoG1) obtained by RT-PCR with primers: pCSG163KOf1 x pGT0LxR1 (Figure 2, PCR3 sample 6).

Seq01: KoG1 sequenced with pGT0LxR1 (files: F11_ba036-g1-pr1_002.seq)

```
5'AGTCTCTTGGTTTTCGGACTGGGTaCCGCTGTTGATGTTTACGATGTATAGATTCGTGCACCCAACTGAT
CTTAGCACTTTTACTTTTGAGCGTTTCCAGCTCCTCTCTTGTGAAGGGCAGGAAGGTGGTGTCCTCGTTGAG
GTTCTTGTTGAAGTTGAA
GT 3'
```

Seq02: KoG1 sequenced with pCSG163KOf1 (files: E11_ba036-g1-pg_001.seq)

```
5'AAGGGCACCCCTTCCTGCCCTTCCAAGAGAGAGCTGGAAAAGCAGGTGAAAGTAAAAGATGCTGAAGATC
AGTTGGGTGCAAAGAGTGGGTTACATCGAACTGGATCTCAACAGCGGTCCCAGGTCCCGAAAACCAAAGAAG
AAGAACGCAGATCTGGAC
TCTAGAG 3'
```

Obs.: the sequencing stopped a little before the initial nucleotides of the Lox site located just upstream of the reverse primer pGT0LxR1.

Blast comparison of Seq01 with the transgenic 200bp cDNA Fragment (KoG1):
Score = 170 bits (188), Expect = 6e-47 Identities = 139/162 (85%), Gaps = 9/162 (5%) Strand=Plus/Minus

```
CSGri   576   TCGACTTCAACAAGAACCTCAACGAGGACACCACCTTCCTGCCCTTCACAAGAGAGGAGC
635
              || |||||||||||||||||||||||||||||||||||||||||||||||||||||||||
seq01   159   TCAACTTCAACAAGAACCTCAACGAGGACACCACCTTCCTGCCCTTCACAAGAGAGGAGC
100

CSGri   636   TGGAAACGCTGGTGAAAGTAAAAGATGCTGAAGATCAGTTGGGTGCACGAGTGGGTTACA
695
              ||||||||||    |||||||||| |||| |||||||||||||||||||| |    ||||
seq01   99    TGGAAACGCT--CAAAAGTAAAAG-TGCT-AAGATCAGTTGGGTGCACGAAT-CTATACA
45

CSGri  696  TCGAACTGGATCTCAACAGCGGTCCCAGGTCCCGAAAACCAA  737
            |||   |  |  ||||||||||| ||  || |||||||||||
seq01  44   TCG---TAAACATCAACAGCGGTACCCAGT-CCGAAAACCAA  7
```

Blast comparison of Seq02 with the transgenic 200bp cDNA Fragment (KoG1):
Score = 233 bits (258), Expect = 6e-66 Identities = 149/157 (94%), Gaps = 4/157 (2%) Strand=Plus/Plus

```
CSGri   600   AGGACACCACCTTCCTGCCCTTCACAAGAGAGGAGCTGGAAACGCTGGTGAAAGTAAAAG
659
              ||| |||| |||||||||||||| |||||||| ||||||||| || ||||||||||||||
seq02   2     AGGGCACC-CCTTCCTGCCCTTC-CAAGAGAG-AGCTGGAAAAGCAGGTGAAAGTAAAAG
58

CSGri   660   ATGCTGAAGATCAGTTGGGTGCAC-GAGTGGGTTACATCGAACTGGATCTCAACAGCGGT
718
              |||||||||||||||||||||||  |||||||||||||||||||||||||||||||||||
```

```
seq02   59    ATGCTGAAGATCAGTTGGGTGCAAAGAGTGGGTTACATCGAACTGGATCTCAACAGCGGT
118

CSGri  719  CCCAGGTCCCGAAAACCAAAGAAGAAGAACGCAGATC  755
             |||||||||||||||||||||||||||||||||||||
seq02  119  CCCAGGTCCCGAAAACCAAAGAAGAAGAACGCAGATC  155
```

Additional information 2, continuation:

Sequencing results for the transgenic 449bp cDNA Fragment (KoG2) obtained by RT-PCR with primers: pCSG163KOf1 x pGT0LxR2 (Figure 2, PCR2 samples 3, 4, 5 and 7 top band).

Seq03 = Fragment KoG2 sequenced with pGT0LxR2(file: A02_ba036-g2-pr2_001.seq)

5'ATGTCTGAGTTTGAGGGCGACGACAGTATCGGCCTCGGAAGATCGCACTTAGCCGCTTTCCGGCACCGCT
TCTGGTGCCGGAAACCAGGCAAAGCGCCATTCGCCATTCAGGCTGCGCAACTGTTGGGAAGGGCGATCGGTG
CGGGCCTCTTCGCTATTACGCCAGCTGGCGAAAGGGGGATGTGCTGCAAGGCGATTAAGTTGGGTAACGCCA
GGGTTTTCCCAGTCACGACGTTGTAAAACGACGGGATCCTCTAGAGTCCAGATCTGCGATAACTTCGTATAA
TGTATGCTATACGAAGTTATCGATCTGCGATCTGCGTTCTTCTTCTTTGGTTTTCGGGACCTGGGACCGCTG
TTGAGATCCAGTTCGATGTAACCCACTCGTGCACCCAACTGATCTTCAGCATCTTTTACTTTCACCAGCGTT
TCCAGCTCCTCTCTTGTGAAGGGCAGGAAGGTGGTGTCCTCGTTGAGGTTCTTGTTGAAGTCGAA 3'

Seq04 = Fragment KoG2 sequenced with pCSG163KOf1(file: E12_ba036-g2-pg_001.seq)

5'AGGACCCCTTCCTGCCCTTCCAAGAGAGGAGCTGGAAACGCTGGTGAAAGTAAAAGATGCTGAAGATCAG
TTGGGTGCACGAGTGGGTTACATCGAACTGGATCTCAACAGCGGTCCCAGGTCCCGAAAACCAAAGAAGAAG
AACGCAGATCGCAGATCGATAACTTCGTATAGCATACATTATACGAAGTTATCGCAGATCTGGACTCTAGAG
GATCCCGTCGTTTTACAACGTCGTGACTGGGAAAACCCTGGCGTTACCCAACTTAATCGCCTTGCAGCACAT
CCCCCTTTCGCCAGCTGGCGTAATAGCGAAGAGGCCCGCACCGATCGCCCTTCCCAACAGTTGCGCAGCCTG
AATGGCGAATGGCGCTTTGCCTGGTTTCCGGCACCAGAAGCGGTGCCGGAAAGCTGGCTGGAGTGCGATCTT
CCTGAGGCCGATACTGTCGTCGTCCCCTCAAACTGGCAGATGCACGGTTACGATGCGCCCATCTACAA 3'

Blast comparison of Seq03 with the transgenic 449bp cDNA Fragment (KoG2):
Score = 836 bits (926), Expect = 0.0 Identities = 484/492 (98%), Gaps = 7/492 (1%) Strand=Plus/Minus

```
CSGri 576   TCGACTTCAACAAGAACCTCAACGAGGACACCACCTTCCTGCCCTTCACAAGAGAGGAGC
 635
            ||||||||||||||||||||||||||||||||||||||||||||||||||||||||||||
seq03  494  TCGACTTCAACAAGAACCTCAACGAGGACACCACCTTCCTGCCCTTCACAAGAGAGGAGC
  435

CSGri  636  TGGAAACGCTGGTGAAAGTAAAAGATGCTGAAGATCAGTTGGGTGCACGAGTGGGTTACA
695
            ||||||||||||||||||||||||||||||||||||||||||||||||||||||||||||
seq03  434  TGGAAACGCTGGTGAAAGTAAAAGATGCTGAAGATCAGTTGGGTGCACGAGTGGGTTACA
  375

CSGri  696 TCGAACTGGATCTCAACAGCGGTCCCAGGTCCCGAAAACCAAAGAAGAAGAACGCAGATC
  755
           ||||||||||||||||||||||||||||||||||||||||||||||||||||||||||||
seq03  374 TCGAACTGGATCTCAACAGCGGTCCCAGGTCCCGAAAACCAAAGAAGAAGAACGCAGATC
  315

CSGri  756   GCA--TCGATAACTTCGTATAGCATACATTATACGAAGTTATCGCAGATCTGGACTCTAG
813
             |||  |||||||||||||||||||||||||||||||||||||||||||||||||||||||
seq03  314   GCAGATCGATAACTTCGTATAGCATACATTATACGAAGTTATCGCAGATCTGGACTCTAG
255

CSGri  814  AGGATCCCGTCGTTTTACAACGTCGTGACTGGGAAAACCCTGGCGTTACCCAACTTAATC
873
            ||||||||||||||||||||||||||||||||||||||||||||||||||||||||||||
```

```
seq03   254   AGGATCCCGTCGTTTTACAACGTCGTGACTGGGAAAACCCTGGCGTTACCCAACTTAATC
195

CSGri  874  GCCTTGCAGCACATCCCCCTTTCGCCAGCTGGCGTAATAGCGAAGAGGCCCGCACCGATC
933
            ||||||||||||||||||||||||||||||||||||||||||||||||||||||||||||
seq03  194  GCCTTGCAGCACATCCCCCTTTCGCCAGCTGGCGTAATAGCGAAGAGGCCCGCACCGATC
135

CSGri  934  GCCCTTCCCAACAGTTGCGCAGCCTGAATGGCGAATGGCGCTTTGCCTGGTTTCCGGCAC
  993
            ||||||||||||||||||||||||||||||||||||||||||||||||||||||||||||
seq03  134  GCCCTTCCCAACAGTTGCGCAGCCTGAATGGCGAATGGCGCTTTGCCTGGTTTCCGGCAC
  75

CSGri  994   CAGAAGCGGTGCCGGAAAGCTGGCTGGAGTGCGATCTTCCTGAGGCCGATACTGTCGTCG
1053
             |||||||||||||||||||| ||||  ||||||||||||| |||||||||||||||||||
seq03  74    CAGAAGCGGTGCCGGAAAGC-GGCT-AAGTGCGATCTTCC-GAGGCCGATACTGTCGTCG
18

CSGri  1054  TCCCCTCAAACT  1065
               ||||||||||
seq03  17    --CCCTCAAACT  8
```

Blast comparison of Seq04 with the transgenic 200bp cDNA Fragment (KoG1):

Score = 868 bits (962), Expect = 0.0 Identities = 489/492 (99%), Gaps = 3/492 (0%) Strand=Plus/Plus

```
CSGri  609   CCTTCCTGCCCTTCACAAGAGAGGAGCTGGAAACGCTGGTGAAAGTAAAAGATGCTGAAG
668
             |||||||||||||| |||||||||||||||||||||||||||||||||||||||||||||
seq04  7     CCTTCCTGCCCTTC-CAAGAGAGGAGCTGGAAACGCTGGTGAAAGTAAAAGATGCTGAAG
65

CSGri  669  ATCAGTTGGGTGCACGAGTGGGTTACATCGAACTGGATCTCAACAGCGGTCCCAGGTCCC
728
            ||||||||||||||||||||||||||||||||||||||||||||||||||||||||||||
seq04  66   ATCAGTTGGGTGCACGAGTGGGTTACATCGAACTGGATCTCAACAGCGGTCCCAGGTCCC
125

CSGri  729   GAAAACCAAAGAAGAAGAACGCAGATCGCA--TCGATAACTTCGTATAGCATACATTATA
786
             ||||||||||||||||||||||||||||||  ||||||||||||||||||||||||||||
seq04  126   GAAAACCAAAGAAGAAGAACGCAGATCGCAGATCGATAACTTCGTATAGCATACATTATA
185

CSGri  787  CGAAGTTATCGCAGATCTGGACTCTAGAGGATCCCGTCGTTTTACAACGTCGTGACTGGG
846
            ||||||||||||||||||||||||||||||||||||||||||||||||||||||||||||
seq04  186  CGAAGTTATCGCAGATCTGGACTCTAGAGGATCCCGTCGTTTTACAACGTCGTGACTGGG
  245

CSGri  847  AAAACCCTGGCGTTACCCAACTTAATCGCCTTGCAGCACATCCCCCTTTCGCCAGCTGGC
  906
            ||||||||||||||||||||||||||||||||||||||||||||||||||||||||||||
seq04  246  AAAACCCTGGCGTTACCCAACTTAATCGCCTTGCAGCACATCCCCCTTTCGCCAGCTGGC
```

```
  305

CSGri  907  GTAATAGCGAAGAGGCCCGCACCGATCGCCCTTCCCAACAGTTGCGCAGCCTGAATGGCG
966
            ||||||||||||||||||||||||||||||||||||||||||||||||||||||||||||
seq04  306  GTAATAGCGAAGAGGCCCGCACCGATCGCCCTTCCCAACAGTTGCGCAGCCTGAATGGCG
  365

CSGri  967  AATGGCGCTTTGCCTGGTTTCCGGCACCAGAAGCGGTGCCGGAAAGCTGGCTGGAGTGCG
 1026
            ||||||||||||||||||||||||||||||||||||||||||||||||||||||||||||
seq04  366  AATGGCGCTTTGCCTGGTTTCCGGCACCAGAAGCGGTGCCGGAAAGCTGGCTGGAGTGCG
425

CSGri  1027 ATCTTCCTGAGGCCGATACTGTCGTCGTCCCCTCAAACTGGCAGATGCACGGTTACGATG
1086
            ||||||||||||||||||||||||||||||||||||||||||||||||||||||||||||
seq04  426  ATCTTCCTGAGGCCGATACTGTCGTCGTCCCCTCAAACTGGCAGATGCACGGTTACGATG
  485

CSGri  1087  CGCCCATCTACA  1098
             ||||||||||||
seq04  486   CGCCCATCTACA  497
```

Additional information 3: Sequence analysis of the PCR DNA fragments obtained from mice CSG163 strain Tail genomic DNA.

Comparison of the nucleotide sequences obtained by long range PCR, the gene trap vector pGT0Lxf and the THOP1 exon 5 to 6 gDNA region. This comparisions were used to define partially the expected sequence for and THOP1-Beta-Geo transgenic gDNA (gCSG1) in orther to design gDNA based genotyping primers.

Wild type genomic DNA nucleotide sequence of THOP1 exon 5 to exon 6 region (gWT):

5'**AAGATCAAGAACATCAAGAAGAGGCTGAGCCTGCTGTGCATCGACTTCAACAAGAACCTCAACGAGGACA**
CCACCTTCCTGCCCTTCACAAGAGAGGAGCTGGgtgagtacacggaatgatgttCgcacagctcccaaggac
actgcagacagagctgggctctgctgacccagatccctgagctggggcagccgtgttgtccttgccgttttc
tgtgagcaagtgtctggggccacaggcacccagccacactgtggggtttagtttaggagtaggggtggtgag
tctcagcccggagtcgggaccttggagcatcagcacggctgtgtgctgtgtgggccttcgctctatggctgt
ggtaccttcactctctggtctgaggccccagcatctctggcgaggcccattccctgctcagcactcatcctc
acttgctgactggggaTatcctgccaaggtcacctacagccctGggaggttcccttcaggttccaagctcag
agctgctggcctgacttcaaggctcCgggctgagcagcgtccgctgcatccacag**GTGGGCTCCCGGAGGAC**
TTTCTGAGCTCTCTGGAGAAGGCAGAGGACGGCAAGCTGAAGGTCACCCTCAAGTACCCTCACTACTTCCCG
CTGTTGAAGAAGTGCCATGTGCCGGAGACACGACGCCTGTTGGAGGAGGCCTTCAACTGTCGCTGCAAGGAG
3'

Legend:
Bold and blue letters – exon 5 and 6 sequeces
Highlighted in green – the sequence of forward primer pGSG163Kof1,
Highlighted in blue – the sequence that reverse primer pGSG163WTR1 aneals.

Sequencing results for the transgenic DNA Fragment (gCSG1) obtained by long range PCR with primers: pCSG163KOf1 x pGT0LxR2 (Figure 3).

Seq05: Fragment gCSG2 sequenced with pCSG163kof1 (C05_ba049-gk-gf1_003.seq)
5'AAGAGACCTTCCTGCCCTTCCAAGCGAGAGCTGGGTGAGTACACGGAATGATGTTCGCACAGCTCCCAAG
GACACTGCAGACAGAGCTGGGCTCTGCTGACCCAGATCCCTGAGCTGGGGCAGCCGTGTTGTCCTTGCCGTT
TTCTGTGAGCAAGTGTCTGGGGCCACAGGCACCCAGCCACACTGTGGGGTTTAGTTTAGGAGTAGGGGTGGT
GAGTCTCAGCCCGGAGTCGGGACCTTGGAGCATCAGCACGGCTGTGTGCTGTGTGGGCCTTCGCTCTATGGC
TGTGGTACCTTCACTCTCTGGTCTGAGGCCCCAGCATCTCTGGCGAGGCCCATTCCCTGCTCAGCACTCATC
CTCACTTGCTGACTGGGGATATCCTGCCAAGGTCACCTACAGCCCTGGGAGGTTCCCTTCAGGTTCCAAGCT
CAGAGCTGCTGGCCTGACTTCAAGGCTCCGGGCTGAGCAGCGCATATGGTGCACTCTCAGTACAATCTGCTC
TGATGCCGCATAGTTAAGCCAGCCCCGACACCCGCCAACACCCGCTGACGCGCCCTGACGGGCTTGTCTGCT
CCCGGCATCCGCTTACAGACAAGCTGTGACCGTCTCCGGGAGCTGCATGTGTCAGAGGTTT 3'

Seq06: Fragment gCSG2 sequenced with pGT0LxR2 (D05_ba049-gk-r2_004.seq)
5'CCGGATCTGCAGTTTGAGGGAGACGACAGTATCGGCCTCAGGAAGATCGCACTCCAGCCAGCTTTCCGGC
ACCGCTTCTGGTGCCGGAAACCAGGCAAAGCGCCATTCGCCATTCAGGCTGCGCAACTGTTGGGAAGGGCGA
TCGGTGCGGGCCTCTTCGCTATTACGCCAGCTGGCGAAAGGGGGATGTGCTGCAAGGCGATTAAGTTGGGTA
ACGCCAGGGTTTTCCCAGTCACGACGTTGTAAAACGACGGGATCCTCTAGAGTCCAGATCTGCGATAACTTC
GTATAATGTATGCTATACGAAGTTATCGATCTGCGATCTGCGTTCTTCTTCTTTGGTTTTCGGGACCTGG 3'

Blast comparison of Seq05 with the pGTOLxf gene trap vector:
Score = 298 bits (330), Expect = 6e-84 Identities = 167/168 (99%), Gaps = 0/168 (0%) Strand=Plus/Minus

```
Vect.  8276 AAACCTCTGACACATGCAGCTCCCGGAGACGGTCACAGCTTGTCTGTAAGCGGATGCCGG
  8335
            ||||||||||||||||||||||||||||||||||||||||||||||||||||||||||||
```

```
seq05  635  AAACCTCTGACACATGCAGCTCCCGGAGACGGTCACAGCTTGTCTGTAAGCGGATGCCGG
  576

Vect.  8336 GAGCAGACAAGCCCGTCAGGGCGCGTCAGCGGGTGTTGGCGGGTGTCGGGGCTGGCTTAA
  8395
            ||||||||||||||||||||||||||||||||||||||||||||||||||||||||||||
seq05  575  GAGCAGACAAGCCCGTCAGGGCGCGTCAGCGGGTGTTGGCGGGTGTCGGGGCTGGCTTAA
  516

Vect.  8396  CTATGCGGCATCAGAGCAGATTGTACTGAGAGTGCACCATATGCGGTG  8443
             |||||||||||||||||||||||||||||||||||||||||||||| ||
seq05  515   CTATGCGGCATCAGAGCAGATTGTACTGAGAGTGCACCATATGCGCTG  468
```

Blast comparison of Seq06 with the pGTOLxf gene trap vector :
Score = 605 bits (670), Expect = 2e-176 Identities = 349/355 (98%), Gaps = 5/355 (1%) Strand=Plus/Minus

```
Vect.  1535  CCAGGTCCCGAAAACCAAAGAAGAAGAACGCAGATCGCA--TCGATAACTTCGTATAGCA
1592
             ||||||||||||||||||||||||||||||||||||||  ||||||||||||||||||||
Seq06  356   CCAGGTCCCGAAAACCAAAGAAGAAGAACGCAGATCGCAGATCGATAACTTCGTATAGCA
297

Vect.  1593 TACATTATACGAAGTTATCGCAGATCTGGACTCTAGAGGATCCCGTCGTTTTACAACGTC
  1652
            ||||||||||||||||||||||||||||||||||||||||||||||||||||||||||||
Seq06  296  TACATTATACGAAGTTATCGCAGATCTGGACTCTAGAGGATCCCGTCGTTTTACAACGTC
  237

Vect.  1653 GTGACTGGGAAAACCCTGGCGTTACCCAACTTAATCGCCTTGCAGCACATCCCCCTTTCG
  1712
            ||||||||||||||||||||||||||||||||||||||||||||||||||||||||||||
Seq06  236  GTGACTGGGAAAACCCTGGCGTTACCCAACTTAATCGCCTTGCAGCACATCCCCCTTTCG
  177

Vect.  1713 CCAGCTGGCGTAATAGCGAAGAGGCCCGCACCGATCGCCCTTCCCAACAGTTGCGCAGCC
  1772
            ||||||||||||||||||||||||||||||||||||||||||||||||||||||||||||
Seq06  176  CCAGCTGGCGTAATAGCGAAGAGGCCCGCACCGATCGCCCTTCCCAACAGTTGCGCAGCC
  117

Vect.  1773 TGAATGGCGAATGGCGCTTTGCCTGGTTTCCGGCACCAGAAGCGGTGCCGGAAAGCTGGC
  1832
            ||||||||||||||||||||||||||||||||||||||||||||||||||||||||||||
Seq06  116  TGAATGGCGAATGGCGCTTTGCCTGGTTTCCGGCACCAGAAGCGGTGCCGGAAAGCTGGC
  57

Vect.  1833  TGGAGTGCGATCTTCCTGAGGCCGATACTGTCGTCGTCCCCTCAAACTGGCAGAT  1887
             ||||||||||||||||||||||||||||||||||   |||||||||| ||||||
Seq06  56    TGGAGTGCGATCTTCCTGAGGCCGATACTGTCGTC--TCCCTCAAACT-GCAGAT  5
```

Score = 280 bits (310), Expect = 9e-79 Identities = 155/155 (100%), Gaps = 0/155 (0%) Strand=Plus/Plus

```
Vect.  8486  GCGCCATTCGCCATTCAGGCTGCGCAACTGTTGGGAAGGGCGATCGGTGCGGGCCTCTTC
  8545
            ||||||||||||||||||||||||||||||||||||||||||||||||||||||||||||
```

```
Seq06  100  GCGCCATTCGCCATTCAGGCTGCGCAACTGTTGGGAAGGGCGATCGGTGCGGGCCTCTTC
  159

Vect.  8546 GCTATTACGCCAGCTGGCGAAAGGGGGATGTGCTGCAAGGCGATTAGTTGGGTAACGCC
  860
            ||||||||||||||||||||||||||||||||||||||||||||||||||||||||||||
Seq06  160  GCTATTACGCCAGCTGGCGAAAGGGGGATGTGCTGCAAGGCGATTAAGTTGGGTAACGCC
  219

Vect.  8606  AGGGTTTTCCCAGTCACGACGTTGTAAAACGACGG  8640
             |||||||||||||||||||||||||||||||||||
Seq06  220   AGGGTTTTCCCAGTCACGACGTTGTAAAACGACGG  254
```

Additional information 3, continuation:

Blast comparison of Seq05 with the THOP1 exon 5 to 6 gDNA region (gWT):

Score = 825 bits (914), Expect = 0.0 Identities = 466/469 (99%), Gaps = 2/469 (0%) Strand=Plus/Plus

```
gWT    73   ACCTTCCTGCCCTTCACAAGAGAGGAGCTGGGTGAGTACACGGAATGATGTTCGCACAGC
132
            ||||||||||||||| |||| ||| |||||||||||||||||||||||||||||||||||
Seq05  6    ACCTTCCTGCCCTTC-CAAGCGAG-AGCTGGGTGAGTACACGGAATGATGTTCGCACAGC
63

gWT    133  TCCCAAGGACACTGCAGACAGAGCTGGGCTCTGCTGACCCAGATCCCTGAGCTGGGGCAG
192
            ||||||||||||||||||||||||||||||||||||||||||||||||||||||||||||
Seq05  64   TCCCAAGGACACTGCAGACAGAGCTGGGCTCTGCTGACCCAGATCCCTGAGCTGGGGCAG
123

gWT    193  CCGTGTTGTCCTTGCCGTTTTCTGTGAGCAAGTGTCTGGGGCCACAGGCACCCAGCCACA
252
            ||||||||||||||||||||||||||||||||||||||||||||||||||||||||||||
Seq05  124  CCGTGTTGTCCTTGCCGTTTTCTGTGAGCAAGTGTCTGGGGCCACAGGCACCCAGCCACA
183

gWT    253  CTGTGGGGTTTAGTTTAGGAGTAGGGGTGGTGAGTCTCAGCCCGGAGTCGGGACCTTGGA
312
            ||||||||||||||||||||||||||||||||||||||||||||||||||||||||||||
Seq05  184  CTGTGGGGTTTAGTTTAGGAGTAGGGGTGGTGAGTCTCAGCCCGGAGTCGGGACCTTGGA
243

gWT    313  GCATCAGCACGGCTGTGTGCTGTGTGGGCCTTCGCTCTATGGCTGTGGTACCTTCACTCT
372
            ||||||||||||||||||||||||||||||||||||||||||||||||||||||||||||
Seq05  244  GCATCAGCACGGCTGTGTGCTGTGTGGGCCTTCGCTCTATGGCTGTGGTACCTTCACTCT
303

gWT    373  CTGGTCTGAGGCCCCAGCATCTCTGGCGAGGCCCATTCCCTGCTCAGCACTCATCCTCAC
432
            ||||||||||||||||||||||||||||||||||||||||||||||||||||||||||||
Seq05  304  CTGGTCTGAGGCCCCAGCATCTCTGGCGAGGCCCATTCCCTGCTCAGCACTCATCCTCAC
363

gWT    433  TTGCTGACTGGGGATATCCTGCCAAGGTCACCTACAGCCCTGGGAGGTTCCCTTCAGGTT
492
```

```
              ||||||||||||||||||||||||||||||||||||||||||||||||||||||||||||
Seq05   364   TTGCTGACTGGGGATATCCTGCCAAGGTCACCTACAGCCCTGGGAGGTTCCCTTCAGGTT
423

gWT     493   CCAAGCTCAGAGCTGCTGGCCTGACTTCAAGGCTCCGGGCTGAGCAGCG   541
              |||||||||||||||||||||||||||||||||||||||||||||||||
Seq05   424   CCAAGCTCAGAGCTGCTGGCCTGACTTCAAGGCTCCGGGCTGAGCAGCG   472
```

The expected sequence for and THOP1-Beta-Geo transgenic gDNA (gCSG1) based on the sequencing results and *in silico* informaiton:

5'**TCGACTTCAACAAGAACCTCAACGAGGACACCACCTTCCTGCCCTTCACAAGAGAGGAGCTGG**gtgagta
cacggaatgatgttcgcacagctcccaaggacactgcagacagagctgggctctgctgacccagatccctga
gctggggcagccgtgttgtccttgccgttttctgtgagcaagtgtctggggccacaggcacccagccacact
gtggggtttagtttaggagtaggggtggtgagtctcagcccggagtcgggaccttggagcatcagcacggct
gtgtgctgtgtgggccttcgctctatggctgtggtaccttcactctctggtctgaggccccagcatctctgg
cgaggcccattccctgctcagcactcatcctcacttgctgactggggatatcctgccaaggtcacctacagc
cctgggaggttcccttcaggttccaagctcagagctgctggcctgacttcaaggctccgggctgagcagcg***C***
ATATGGTGCACTCTCAGTACAATCTGCTCTGATGCCGCATAGTTAAGCCAGCCCCGACACCCGCCAACACCC
GCTGACGCGCCCTGACGGGCTTGTCTGCTCCCGGCATCCGCTTACAGACAAGCTGTgACCGTCAGGGGC...
.
AAACGCTGGTGAAAGTAAAAGATGCTGAAGATCAGTTGGGTGCACGAGTGGGTTACATCGAACTGGATCTCA
ACAGCGGTCCCAGGTCCCGAAAACCAAAGAAGAAGAACGCAGATCGCATCGATAACTTCGTATAGCATACAT
TATACGAAGTTATCGCAGATCTGGACTCTAGAGGATCCCGTCGTTTTACAACGTCGTGACTGGGAAAACCCT
GGCGTTACCCAACTTAATCGCCTTGCAGCACATCCCCCTTTCGCCAGCTGGCGTAATAGCGAAGAGGCCCGC
ACCGATCGCCCTTCCCAACAGTTGCGCAGCCTGAATGGCGAATGGCGCTTTGCCTGGTTTCCGGCACCAGAA
GCGGTGCCGGAAAGCTGGCTGGAGTGCGATCTTCCTGAGGCCGATACTGTCGTCGTCCCCTCAAACTGGCAG
ATGCACGGTTACGATGCGCCCATCTACA 3'

Legend:
blue letters – 3'end of THOP1 exon 5 (in bold) and initinal intron 5 sequence,
hightlighted in green – the sequence of forward primer pGSG163Kof1
In bold and red letters – pGT0Lxf vector sequences in the antisense orientation that resulted from a non documented recombination event,
yellow highlighted red letters – the sequence that gDNa genotyping reverse primer CSGkoR2 aneals,
In green letters – gene Trap vector sequence corresponding to the end of the intronic region, splice acceptor, the initial exon 2 nucleotides of the mouse engrailed 2 gene,
In black letters – pGT0Lxf vector sequences, were: underlined is a Lox site, in bold is the initial coding sequence of Beta-Geo,
Highlighted in light blue – the sequence that reverse primer pGTLxR1 aneals,
Highlighted in dark blue – the sequence that reverse primer pGTLxR2 aneals.

Additional information 4: Sequence analysis of the PCR DNA fragments obtained from mice CSG163 strain genomic Tail DNA genotyping.
Sequencing results for the wild type Fragment obtained with primers pCSG163KOf1 and pCSG163WTr1 (figure 4).

Seq07: gDNA wild type Fragment obtained with primers pCSG163KOf1 and pCSG163WTr1 sequenced with pCSG163KOf1 (E04_ba092-gw-f1_001.seq)

```
5'AAGGCCCACCCTTCCTGCCCTTCAATGAGAGGAGCTGGGTGTGTACACGGAATGATGTTCGCACTGCTCC
CAAGGACACTGCAGACAGAGCTGGGCTCTGCTGACCCAGATCCCTGAGCTGGGGCAGCCGTGTTGTCCTTGC
CGTTTTCTGTGAGCAAGTGTCTGGGGCCACAGGCACCCAGCCACACTGTGGGGTTTAGTTTAGGAGTAGGGG
TGGTGAGTCTCAGCCCGGAGTCGGGACCTTGGAGCATCAGCACGGCTGTGTGCTGTGTGGGCCTTCGCTCTA
TGGCTGTGGTACCTTCACTCTCTGGTCTGAGGCCCCAGCATCTCTGGCGAGGCCCATTCCCTGCTCAGCACT
CATCCTCACTTGCTGACTGGGGATATCCTGCCAAGGTCACCTACAGCCCTGGGAGGTTCCCTTCAGGTTCCA
AGCTCAGAGCTGCTGGCCTGACTTCAAGGCTCCGGGCTGAGCAGCGTCCGCTGCATCCACAGGTGGGCTCCC
GGAGGACTTTCTGAGCTCTCTGGAAAAAAGGCA 3'
```

Seq08: gDNA wild type Fragment obtained with primers pCSG163KOf1 and pCSG163WTr1 sequenced with pCSG163WTr1 (F04_ba092-gw-r1_002.seq)

```
5'TAGTTGAAGCCTTGCCCACCCGTAACGCAGGGGGCGGGGTGCGCCCCGAAATGATGGTACGCAACTCCCA
AGGACACTGCAAAACTGGCTGGACTCTGCAGACCTGTATGCGAGATCTGGGGGATATCCGTTGTCCTTGGTG
AGGATGAGTGCAAGTGTCTGGGGCCACCTGCACCCAGATGCTCTGTGCGGTTAAGTTTAAGAGTAGGTACTG
GTGAGTTTCAGCCAAGGCTCACACCCTTGCAGCATCAGCTCAGCTGTGTGGTGTGTGCTCCTTCCTTCTACT
GATGACCTACCCTCATAATCTGGTCTGAGGCCGTGGCTGGGTGCCTGGGCCCATTCCACTTGATCACTCAAA
ACTCACTTGCTGACTGGGCATATCCTGCCAAGGTCACCTACTCACCTGAGACGATCTCTTTATGTTCCGAGC
TTAGAAGTGCTGCCAAGATTTTTACGCTTCCTGCTGAGCATCCTCTCTTGCATCCACAGGTGGGCTCCCGGA
GGACTTTCTGTTCTTGTTGAAAAAAGGCA 3'
```

Blast comparison of Seq07 with Seq08:
Score = 289 bits (320), Expect = 2e-82 Identities = 366/492 (74%), Gaps = 11/492 (2%) Strand=Plus/Plus

```
Seq07  48   CGGAATGATGTT-CGCACTGCTCCCAAGGACACTGCAGACAGAGCTGGGCTCTGCTGACC  106
            || ||||||| | ||||   ||||||||||||||||| |    ||||| |||||| ||||
Seq08  47   CGAAATGATGGTACGCAA--CTCCCAAGGACACTGCAAAACTGGCTGGACTCTGCAGACC  104

Seq07  107  CAGATCCCTGAGCTGGGGCAGCCGTGTTGTCCTTGCCGTTTTCTGTGAGCAAGTGTCTGG  166
               || |  || ||||||| |     ||||||||||  |     || | ||||||||||||
Seq08  105  TGTATGCGAGATCTGGGGGATATCCGTTGTCCTTGGTGAGGA-TGAGTGCAAGTGTCTGG  163

Seq07  167  GGCCACAGGCACCCAGCCACACTGTGGGGTTTAGTTTAGGAGTAGGGG-TGGTGAGTCTC  225
            ||||||  ||||||||   | ||||| |||| |||||| |||||||   |||||||| ||
Seq08  164  GGCCACCTGCACCCAGATGCTCTGTGCGGTTAAGTTTAAGAGTAGGTACTGGTGAGTTTC  223

Seq07  226  AGCCCGGAGTCGGGACCTTGGAGCATCAGCACGGCTGTGTGCTGTGTGGGCCTTCGCTCT  285
            ||||  |  ||    ||||| ||||||||| | |||||||| ||||||  |||||  |||
Seq08  224  AGCCAAGGCTCACACCCTTGCAGCATCAGCTCAGCTGTGTGGTGTGTGCTCCTTCCTTCT  283

Seq07  286  ATGGCTGTGGTACCTTCACTCTCTGGTCTGAGGCCCCAGCATCTCTGGCGAGGCCCATTC  345
            |  | ||   |||| |||   ||||||||||||||   || |   || |  |||||||||
```

```
Seq08   284   ACTGATGACCTACCCTCATAATCTGGTCTGAGGCCGTGGC-TGGGTGCCTGGGCCCATTC
342

Seq07   346   C-CTGCTCAGCACTCATC-CTCACTTGCTGACTGGGGATATCCTGCCAAGGTCACCTACA
403
              | ||  | | ||||||   ||||||||||||||||| ||||||||||||||||||||||
Seq08   343   CACT--TGATCACTCAAAACTCACTTGCTGACTGGGCATATCCTGCCAAGGTCACCTACT
400

Seq07   404   GCCCTGGGAGGTTCCCTTCAGGTTCCAAGCTCAGAGCTGCTGGCCTGACTTCAAGGCTCC
463
                |||| || | || ||| | ||||| |||| |||  ||||| |  || ||  | ||| |
Seq08   401   CACCTGAGACGATCTCTTTATGTTCCGAGCTTAGAAGTGCTGCCAAGATTTTACGCTTC
460

Seq07   464   GGGCTGAGCAGCGTCCGCTGCATCCACAGGTGGGCTCCCGGAGGACTTTCTGAGCTCTCT
523
                |||||||| | ||   ||||||||||||||||||||||||||||||||||  || | |
Seq08   461   CTGCTGAGCATCCTCTCTTGCATCCACAGGTGGGCTCCCGGAGGACTTTCTGTTCT-TGT
519

Seq07  524  GGAAAAAAGGCA  535
             |||||||||||
Seq08  520  TGAAAAAAGGCA  531
```

Blast comparison of Seq07 with the THOP1 exon 5 to 6 gDNA region (gWT):

```
 Score =  913 bits (1012),  Expect = 0.0  Identities = 516/521 (99%), Gaps
= 1/521 (0%)  Strand=Plus/Plus

gWT      34   CCTTCCTGCCCTTCACAAGAGAGGAGCTGGGTGAGTACACGGAATGATGTTCGCACAGCT
93
              ||||||||||||||| | ||||||||||||||| |||||||||||||||||||||| |||
Seq07   10    CCTTCCTGCCCTTCA-ATGAGAGGAGCTGGGTGTGTACACGGAATGATGTTCGCACTGCT
68

gWT     94   CCCAAGGACACTGCAGACAGAGCTGGGCTCTGCTGACCCAGATCCCTGAGCTGGGGCAGC
  153
             ||||||||||||||||||||||||||||||||||||||||||||||||||||||||||||
Seq07   69   CCCAAGGACACTGCAGACAGAGCTGGGCTCTGCTGACCCAGATCCCTGAGCTGGGGCAGC
  128

gWT     154  CGTGTTGTCCTTGCCGTTTTCTGTGAGCAAGTGTCTGGGGCCACAGGCACCCAGCCACAC
  213
             ||||||||||||||||||||||||||||||||||||||||||||||||||||||||||||
Seq07   129  CGTGTTGTCCTTGCCGTTTTCTGTGAGCAAGTGTCTGGGGCCACAGGCACCCAGCCACAC
  188

gWT     214  TGTGGGGTTTAGTTTAGGAGTAGGGGTGGTGAGTCTCAGCCCGGAGTCGGGACCTTGGAG
  273
             ||||||||||||||||||||||||||||||||||||||||||||||||||||||||||||
Seq07   189  TGTGGGGTTTAGTTTAGGAGTAGGGGTGGTGAGTCTCAGCCCGGAGTCGGGACCTTGGAG
  248

gWT     274  CATCAGCACGGCTGTGTGCTGTGTGGGCCTTCGCTCTATGGCTGTGGTACCTTCACTCTC
  333
             ||||||||||||||||||||||||||||||||||||||||||||||||||||||||||||
Seq07   249  CATCAGCACGGCTGTGTGCTGTGTGGGCCTTCGCTCTATGGCTGTGGTACCTTCACTCTC
```

```
  308

gWT    334  TGGTCTGAGGCCCCAGCATCTCTGGCGAGGCCCATTCCCTGCTCAGCACTCATCCTCACT
  393
            ||||||||||||||||||||||||||||||||||||||||||||||||||||||||||||
Seq07  309  TGGTCTGAGGCCCCAGCATCTCTGGCGAGGCCCATTCCCTGCTCAGCACTCATCCTCACT
  368

gWT    394  TGCTGACTGGGGATATCCTGCCAAGGTCACCTACAGCCCTGGGAGGTTCCCTTCAGGTTC
  453
            ||||||||||||||||||||||||||||||||||||||||||||||||||||||||||||
Seq07  369  TGCTGACTGGGGATATCCTGCCAAGGTCACCTACAGCCCTGGGAGGTTCCCTTCAGGTTC
  428

gWT    454  CAAGCTCAGAGCTGCTGGCCTGACTTCAAGGCTCCGGGCTGAGCAGCGTCCGCTGCATCC
  513
            ||||||||||||||||||||||||||||||||||||||||||||||||||||||||||||
Seq07  429  CAAGCTCAGAGCTGCTGGCCTGACTTCAAGGCTCCGGGCTGAGCAGCGTCCGCTGCATCC
  488

gWT    514  ACAGGTGGGCTCCCGGAGGACTTTCTGAGCTCTCTGGAGAA  554
            |||||||||||||||||||||||||||||||||||||| ||
Seq07  489  ACAGGTGGGCTCCCGGAGGACTTTCTGAGCTCTCTGGAAAA  529
```
Additional information 4, continuation:

Blast comparison of Seq08 with the THOP1 exon 5 to 6 gDNA region (gWT):

Score = 280 bits (310), Expect = 8e-80 Identities = 350/471 (74%), Gaps = 8/471 (1%) Strand=Plus/Plus

```
gWT     73    CGGAATGATGTTCGCACAGCTCCCAAGGACACTGCAGACAGAGCTGGGCTCTGCTGACCC
132
              || ||||||| |  | || ||||||||||||||||| |    ||||| |||||| ||||
Seq08   47    CGAAATGATGGTA-CGCAACTCCCAAGGACACTGCAAAACTGGCTGGACTCTGCAGACCT
105

gWT     133   AGATCCCTGAGCTGGGGCAGCCGTGTTGTCCTTGCCGTTTTCTGTGAGCAAGTGTCTGGG
192
                || |  || |||||| |     ||||||||||  |     || | |||||||||||||
Seq08   106   GTATGCGAGATCTGGGGGATATCCGTTGTCCTTGGTGAGGA-TGAGTGCAAGTGTCTGGG
164

gWT     193   GCCACAGGCACCCAGCCACACTGTGGGGTTTAGTTTAGGAGTAGGGG-TGGTGAGTCTCA
251
              |||||  ||||||||   | ||||| |||| |||||| |||||||   |||||||| |||
Seq08   165   GCCACCTGCACCCAGATGCTCTGTGCGGTTAAGTTTAAGAGTAGGTACTGGTGAGTTTCA
224

gWT     252   GCCCGGAGTCGGGACCTTGGAGCATCAGCACGGCTGTGTGCTGTGTGGGCCTTCGCTCTA
311
              |||  |  ||    ||||| ||||||||| | |||||||| ||||||  |||||  ||||
Seq08   225   GCCAAGGCTCACACCCTTGCAGCATCAGCTCAGCTGTGTGGTGTGTGCTCCTTCCTTCTA
284

gWT     312   TGGCTGTGGTACCTTCACTCTCTGGTCTGAGGCCCCAGCATCTCTGGCGAGGCCCATTCC
371
                | ||   |||| |||   ||||||||||||||   || |   || |  ||||||||||
Seq08   285   CTGATGACCTACCCTCATAATCTGGTCTGAGGCCGTGGC-TGGGTGCCTGGGCCCATTCC
343
```

```
gWT     372   -CTGCTCAGCACTCATC-CTCACTTGCTGACTGGGGATATCCTGCCAAGGTCACCTACAG
429
               ||  | | ||||||   ||||||||||||||||| ||||||||||||||||||||||
Seq08   344   ACT--TGATCACTCAAAACTCACTTGCTGACTGGGCATATCCTGCCAAGGTCACCTACTC
401

gWT     430   CCCTGGGAGGTTCCCTTCAGGTTCCAAGCTCAGAGCTGCTGGCCTGACTTCAAGGCTCCG
489
               |||| || | || ||| | ||||| |||| |||  ||||| |  || ||  | ||| |
Seq08   402   ACCTGAGACGATCTCTTTATGTTCCGAGCTTAGAAGTGCTGCCAAGATTTTTACGCTTCC
461

gWT    490  GGCTGAGCAGCGTCCGCTGCATCCACAGGTGGGCTCCCGGAGGACTTTCTG  540
             |||||||| | ||   ||||||||||||||||||||||||||||||||||
Seq08  462  TGCTGAGCATCCTCTCTTGCATCCACAGGTGGGCTCCCGGAGGACTTTCTG  512
```

Analysis of the transgenic DNA Fragment obtained with primers pCSG163KOf1 and CSG163R2 from genotyping

Sequencing results for the transgenic DNA Fragment (gCSGk) obtained by genotyping PCR with primers: pCSG163KOf1 x CSG163R2 (Figure 4).

Seq09 Fragment gCSGk sequenced with pCSG163KOf1(G04_ba092-gk-f1_003.seq)

```
5'AAGGACTCCCTTGTCCTGCCTTTACAAGGAAAGGCGCTGGGTGCGTTCACGGAATGATGTTCGACAGCTC
CCAAGGACACTGCAGACAGAGCTGGGCTCTGCTGACCCAGATCCCTGAGCTGGGGCAGCCGTGTTGTCCTTG
CCGTTTTCTGTGAGCAAGTGTCTGGGGCCACAGGCACCCAGCCACACTGTGGGGTTTAGTTTAGGAGTAGGG
GTGGTGAGTCTCAGCCCGGAGTCGGGACCTTGGAGCATCAGCACGGCTGTGTGCTGTGTGGGCCTTCGCTCT
ATGGCTGTGGTACCTTCACTCTCTGGTCTGAGGCCCCAGCATCTCTGGCGAGGCCCATTCCCTGCTCAGCAC
TCATCCTCACTTGCTGACTGGGGATATCCTGCCAAGGTCACCTACAGCCCTGGGAGGTTCCCTTCAGGTTCC
AAGCTCAGAGCTGCTGGCCTGACTTCAAGGCTCCGGGCTGAGCAGCG 3'
```

Seq10 Fragment gCSGk sequenced with CSGKOR2 (H04_ba092-gk-r2_004.seq)

```
5'CTGCGATGCTGGTGCATGCTGCCCATTTGTAAACGACTCGGGAGGTGTCTGCTGCTGGGGCCGGCTTAAC
AATGCCTATTCTATGAAATTGCACTGAGTGTGCACCATATGCACTGCGTATCCCGGAGCCTTGAAGTGGGGC
CTTTGTCTCTGAGCTTGGAACCTGAAGGGAACCTAACAGGGCTGTAGGTGACCTTGGCCAGATATCCCCAGT
CATGAAGTGAGGATCAGTGCTTTGCAGGGAATGGGACTAATTATACATGCTGGCCCCTATCACCGAAGAGTG
AAGGTACCACACTCCTCTATAAGAAGGCCCACACTGTGGACAGATGTGCTGTTGCTTCAAGGTCTTAAACTT
GAGCTTTGCTCACCACCATAACTCCTAAACTAAACCCCACAGTGTGGCTGGTTGCCTGTGGCCCCACACACT
TGCTCACAGAAAACATGCTGGACAACACCGCTGCCCCAGCTTAAGGTTCTGGGTCAACAAACCCCAGCTCTT
TCTGGTGTGTCCTTGGGAGCTGTGCTAACATCATTCCGTGTACTCACCCAGCTCCTCTCTTGTGAAGGGCAC
CATGGTGGTGTCAACAAAATTGTTCTTGTTTAACATCTTAAAGTCAGGCATGGTGATTCCCCCAGAGGTTCT
TGTTGAAGTCAAAAT 3'
```

Blast comparison of Seq01 with the THOP1 exon 5 to 6 gDNA region (gWT):

Score = 794 bits (880), Expect = 0.0 Identities = 458/466 (98%), Gaps = 3/466 (0%) Strand=Plus/Plus

```
gWT     37    TCCTGCCCTTCACAAG-AGAGGAGCTGGGTGAGTACACGGAATGATGTTCGCACAGCTCC
95
              ||||||| || ||||| | ||| |||||||| || |||||||||||||||| ||||||||
Seq09   14    TCCTGCCTTT-ACAAGGAAAGGCGCTGGGTGCGTTCACGGAATGATGTTCG-ACAGCTCC
71
```

```
gWT    96   CAAGGACACTGCAGACAGAGCTGGGCTCTGCTGACCCAGATCCCTGAGCTGGGGCAGCCG
 155
            ||||||||||||||||||||||||||||||||||||||||||||||||||||||||||||
Seq09  72   CAAGGACACTGCAGACAGAGCTGGGCTCTGCTGACCCAGATCCCTGAGCTGGGGCAGCCG
 131

gWT    156  TGTTGTCCTTGCCGTTTTCTGTGAGCAAGTGTCTGGGGCCACAGGCACCCAGCCACACTG
 215
            ||||||||||||||||||||||||||||||||||||||||||||||||||||||||||||
Seq09  132  TGTTGTCCTTGCCGTTTTCTGTGAGCAAGTGTCTGGGGCCACAGGCACCCAGCCACACTG
 191

gWT    216  TGGGGTTTAGTTTAGGAGTAGGGGTGGTGAGTCTCAGCCCGGAGTCGGGACCTTGGAGCA
 275
            ||||||||||||||||||||||||||||||||||||||||||||||||||||||||||||
Seq09  192  TGGGGTTTAGTTTAGGAGTAGGGGTGGTGAGTCTCAGCCCGGAGTCGGGACCTTGGAGCA
 251

gWT    276  TCAGCACGGCTGTGTGCTGTGTGGGCCTTCGCTCTATGGCTGTGGTACCTTCACTCTCTG
 335
            ||||||||||||||||||||||||||||||||||||||||||||||||||||||||||||
Seq09  252  TCAGCACGGCTGTGTGCTGTGTGGGCCTTCGCTCTATGGCTGTGGTACCTTCACTCTCTG
 311

gWT    336  GTCTGAGGCCCCAGCATCTCTGGCGAGGCCCATTCCCTGCTCAGCACTCATCCTCACTTG
 395
            ||||||||||||||||||||||||||||||||||||||||||||||||||||||||||||
Seq09  312  GTCTGAGGCCCCAGCATCTCTGGCGAGGCCCATTCCCTGCTCAGCACTCATCCTCACTTG
 371

gWT    396  CTGACTGGGGATATCCTGCCAAGGTCACCTACAGCCCTGGGAGGTTCCCTTCAGGTTCCA
 455
            ||||||||||||||||||||||||||||||||||||||||||||||||||||||||||||
Seq09  372  CTGACTGGGGATATCCTGCCAAGGTCACCTACAGCCCTGGGAGGTTCCCTTCAGGTTCCA
 431

gWT    456  AGCTCAGAGCTGCTGGCCTGACTTCAAGGCTCCGGGCTGAGCAGCG  501
            ||||||||||||||||||||||||||||||||||||||||||||||
Seq09  432  AGCTCAGAGCTGCTGGCCTGACTTCAAGGCTCCGGGCTGAGCAGCG  477
```

Additional information 4, continuation:

Blast comparison of Seq09 with Seq10:

```
 Score =  455 bits (504),  Expect = 3e-132  Identities = 426/535 (79%),
Gaps = 5/535 (0%)  Strand=Plus/Minus

Seq09   19    CCTTTACAAGGAAAGGCGCTGGGTGCGTTCACGGAATGATGTTCG-ACAGCTCCCAAGGA
77
              |||| ||||| | ||| |||||||| || |||||||||||||| | ||||||||||||||
Seq10   570   CCTTCACAAG-AGAGGAGCTGGGTGAGTACACGGAATGATGTTAGCACAGCTCCCAAGGA
512

Seq09   78    CACTGCAGACAGAGCTGGGCTCTGCTGACCCAGATCCCTGAGCTGGGGCAGCCGTGTTGT
137
              |||  |||| ||||||||| | || ||||||||| || | |||||||||||| |||||||
Seq10   511   CACACCAGAAAGAGCTGGGGTTTGTTGACCCAGAACCTTAAGCTGGGGCAGCGGTGTTGT
452

Seq09   138   CCTTGCCGTTTTCTGTGAGCAAGTGTCTGGGGCCACAGGCACCCAGCCACACTGTGGGGT
197
              ||     ||||||||||||||||||| |||||||||||||| ||||||||||||||||||
Seq10   451   CCAGCATGTTTTCTGTGAGCAAGTGTGTGGGGCCACAGGCAACCAGCCACACTGTGGGGT
392

Seq09   198   TTAGTTTAGGAGTAGGGGTGGTGAGTCTCAGCCCGGAGTCGGGACCTTGGAGCATCAGCA
257
              |||||||||||||   ||||||||| |  ||| |    |    ||||||| |||| |||||
Seq10   391   TTAGTTTAGGAGTTATGGTGGTGAG-CAAAGCTCAAGTTTAAGACCTTGAAGCAACAGCA
333

Seq09   258   CGGCTGTGTGCTGTGTGGGCCTTCGC-TCTATGGCTGTGGTACCTTCACTCTCTGGTCTG
316
              |  ||||   | ||||||||||||   |  | |  |||||||||||||||||  |||
Seq10   332   CATCTGTCCACAGTGTGGGCCTTCTTATAGAGGAGTGTGGTACCTTCACTCTTCGGTGAT
273

Seq09   317   AGGCCCCAGCATCTCTGGCGAGGCCCATTCCCTGCTCAGCACTCATCCTCACTTGCTGAC
376
              |||  ||||||| | |    || ||||||||||||  |||||| ||||||||||  ||||
Seq10   272   AGGGGCCAGCATGTATAATTAGTCCCATTCCCTGCAAAGCACTGATCCTCACTTCATGAC
213

Seq09   377   TGGGGATATCCTGCCAAGGTCACCTACAGCCCTGGGAGGTTCCCTTCAGGTTCCAAGCTC
436
              ||||||||||  ||||||||||||||||||||||  ||||||||||||||||||||||||
Seq10   212   TGGGGATATCTGGCCAAGGTCACCTACAGCCCTGTTAGGTTCCCTTCAGGTTCCAAGCTC
153

Seq09   437   AGAGCTGCTGGCCTGACTTCAAGGCTCCGGGCTGAGCAGCGCATATGGTGCACTCTCAGT
496
              ||||     ||||  |||||||||||||||| |  |||| ||||||||||||| ||||||
Seq10   152   AGAGACAAAGGCCCCACTTCAAGGCTCCGGGATACGCAGTGCATATGGTGCACACTCAGT
93

Seq09  497  ACAATCTGCTCTGATGCCGCATAGTTAAGCCAGCCCCGACACCCGCCAACACCCG  551
             |||| | |   ||    |||| |||||||| |||||  || | | || | ||||
Seq10  92   GCAAT-TTCATAGAATAGGCATTGTTAAGCCGGCCCCAGCAGCAGACACCTCCCG  39
```

Blast comparison of Seq09 with the pGTOLxf gene trap vector:

```
 Score =  237 bits (262),  Expect = 2e-65  Identities = 133/134 (99%),
Gaps = 0/134 (0%)  Strand=Plus/Minus

vect.  8310 ACAGCTTGTCTGTAAGCGGATGCCGGGAGCAGACAAGCCCGTCAGGGCGCGTCAGCGGGT
  8369
            ||||||||||||||||||||||||||||||||||||||||||||||||||||||||||||
Seq09  606  ACAGCTTGTCTGTAAGCGGATGCCGGGAGCAGACAAGCCCGTCAGGGCGCGTCAGCGGGT
  547

vect.  8370 GTTGGCGGGTGTCGGGGCTGGCTTAACTATGCGGCATCAGAGCAGATTGTACTGAGAGTG
  8429
            ||||||||||||||||||||||||||||||||||||||||||||||||||||||||||||
Seq09  546  GTTGGCGGGTGTCGGGGCTGGCTTAACTATGCGGCATCAGAGCAGATTGTACTGAGAGTG
  487

vect.  8430  CACCATATGCGGTG  8443
             ||||||||||| ||
Seq09  486   CACCATATGCGCTG  473
```

Blast comparison of Seq10 with the pGTOLxf gene trap vector:

```
 Score =  605 bits (670),  Expect = 2e-176  Identities = 349/355 (98%),
Gaps = 5/355 (1%)  Strand=Plus/Minus

Vect.  1535  CCAGGTCCCGAAAACCAAAGAAGAAGAACGCAGATCGCA--TCGATAACTTCGTATAGCA
1592
             |||||||||||||||||||||||||||||||||||||||  |||||||||||||||||||
Seq10  356   CCAGGTCCCGAAAACCAAAGAAGAAGAACGCAGATCGCAGATCGATAACTTCGTATAGCA
297

Vect.  1593 TACATTATACGAAGTTATCGCAGATCTGGACTCTAGAGGATCCCGTCGTTTTACAACGTC
  1652
            ||||||||||||||||||||||||||||||||||||||||||||||||||||||||||||
Seq10  296  TACATTATACGAAGTTATCGCAGATCTGGACTCTAGAGGATCCCGTCGTTTTACAACGTC
  237

Vect.  1653 GTGACTGGGAAAACCCTGGCGTTACCCAACTTAATCGCCTTGCAGCACATCCCCCTTTCG
  1712
            ||||||||||||||||||||||||||||||||||||||||||||||||||||||||||||
Seq10  236  GTGACTGGGAAAACCCTGGCGTTACCCAACTTAATCGCCTTGCAGCACATCCCCCTTTCG
  177

Vect.  1713 CCAGCTGGCGTAATAGCGAAGAGGCCCGCACCGATCGCCCTTCCCAACAGTTGCGCAGCC
  1772
            ||||||||||||||||||||||||||||||||||||||||||||||||||||||||||||
Seq10  176  CCAGCTGGCGTAATAGCGAAGAGGCCCGCACCGATCGCCCTTCCCAACAGTTGCGCAGCC
  117

Vect.  1773 TGAATGGCGAATGGCGCTTTGCCTGGTTTCCGGCACCAGAAGCGGTGCCGGAAAGCTGGC
  1832
            ||||||||||||||||||||||||||||||||||||||||||||||||||||||||||||
Seq10  116  TGAATGGCGAATGGCGCTTTGCCTGGTTTCCGGCACCAGAAGCGGTGCCGGAAAGCTGGC
  57

Vect.  1833  TGGAGTGCGATCTTCCTGAGGCCGATACTGTCGTCGTCCCCTCAAACTGGCAGAT  1887
             |||||||||||||||||||||||||||||||||||   |||||||||| ||||||
Seq10  56    TGGAGTGCGATCTTCCTGAGGCCGATACTGTCGTC--TCCCTCAAACT-GCAGAT  5
```

```
 Score =  280 bits (310),  Expect = 9e-79 Identities = 155/155 (100%),
Gaps = 0/155 (0%) Strand=Plus/Plus

Vect.  8486 GCGCCATTCGCCATTCAGGCTGCGCAACTGTTGGGAAGGGCGATCGGTGCGGGCCTCTTC
  8545
            ||||||||||||||||||||||||||||||||||||||||||||||||||||||||||||
Seq10  100  GCGCCATTCGCCATTCAGGCTGCGCAACTGTTGGGAAGGGCGATCGGTGCGGGCCTCTTC
  159

Vect.  8546 GCTATTACGCCAGCTGGCGAAAGGGGGATGTGCTGCAAGGCGATTAAGTTGGGTAACGCC
  8605
            ||||||||||||||||||||||||||||||||||||||||||||||||||||||||||||
Seq10  160  GCTATTACGCCAGCTGGCGAAAGGGGGATGTGCTGCAAGGCGATTAAGTTGGGTAACGCC
  219

Vect.  8606  AGGGTTTTCCCAGTCACGACGTTGTAAAACGACGG  8640
             |||||||||||||||||||||||||||||||||||
Seq10  220   AGGGTTTTCCCAGTCACGACGTTGTAAAACGACGG  254
```
